# Convergent reduction of *V1R* genes in subterranean rodents

**DOI:** 10.1186/s12862-019-1502-4

**Published:** 2019-08-30

**Authors:** Hengwu Jiao, Wei Hong, Eviatar Nevo, Kexin Li, Huabin Zhao

**Affiliations:** 1 Department of Ecology, Hubei Key Laboratory of Cell Homeostasis, College of Life Sciences, Wuhan University, 299 Bayi Road, Wuhan, 430072 Hubei China; 2 Institute of Evolution, University of Haifa, Mount Carmel, 31905 Haifa, Israel

**Keywords:** *V1R*, Pheromonal olfaction, Subterranean rodents, Gene family

## Abstract

**Background:**

Vomeronasal type 1 receptor genes (*V1R*s) are expected to detect intraspecific pheromones. It is believed that rodents rely heavily on pheromonal communication mediated by *V1R*s, but pheromonal signals are thought to be confined in subterranean rodents that live in underground burrows. Thus, subterranean rodents may show a contrasting mode of *V1R* evolution compared with their superterranean relatives.

**Results:**

We examined the *V1R* evolution in subterranean rodents by analyzing currently available genomes of 24 rodents, including 19 superterranean and 5 subterranean species from three independent lineages. We identified a lower number of putatively functional *V1R* genes in each subterranean rodent (a range of 22–40) compared with superterranean species (a range of 63–221). After correcting phylogenetic inertia, the positive correlation remains significant between the small *V1R* repertoire size and the subterranean lifestyle. To test whether *V1R*s have been relaxed from functional constraints in subterranean rodents, we sequenced 22 intact *V1R*s in 29 individuals of one subterranean rodent (*Spalax galili*) from two soil populations, which have been proposed to undergo incipient speciation. We found 12 of the 22 *V1R*s to show significant genetic differentiations between the two natural populations, indicative of diversifying selection.

**Conclusion:**

Our study demonstrates convergent reduction of *V1R*s in subterranean rodents from three independent lineages. Meanwhile, it is noteworthy that most *V1Rs* in the two *Spalax* populations are under diversifying selection rather than relaxed selection, suggesting that functional constraints on these genes may have retained in some subterranean species.

**Electronic supplementary material:**

The online version of this article (10.1186/s12862-019-1502-4) contains supplementary material, which is available to authorized users.

## Background

Olfaction has been widely believed to trigger essential animal behaviors such as mate choice, food location and predator avoidance [1]. In most tetrapod vertebrates, two olfactory systems are present: the main olfactory system (MOS) and the vomeronasal system (VNS) [2]. Through distinct signal transduction pathways, the two olfactory systems act in concert to detect odorants [3]. The intraspecific pheromones are species-specific odor molecules that trigger sexual and social behaviors in conspecific individuals [4], which are more likely to evoke the VNS chemoreceptors that show an evolutionary pattern with a species-specific manner; By contrast, the MOS chemoreceptors show an evolutionary pattern with a relatively conserved manner [3, 5]. There are at least three families of receptor genes that are expressed in the VNS, including vomeronasal type 1 receptor genes (*V1R*s), vomeronasal type 2 receptor genes (*V2R*s), and formyl peptide receptor genes (*FPR*s) [6–8]. Of the three gene families, *V1R*s are of particular interest for evolutionary analysis within a phylogenetic framework, because the *V1R* gene family in mammals shows dramatic among-species variation in size, from 0 in two bat species, dolphin and rhesus macaque to 283 in platypus [9–12]. Earlier studies have reported that five surface-dwelling rodents (superterranean rodents) have an expanded *V1R* repertoire and suggested that *V1R*s play an important role in pheromonal communication in these animals [12, 13]. Indeed, mutant mice lacking 16 intact *V1R* genes showed a reduced level of maternal aggression and male sexual drive [14]. To our knowledge, however, all studies published so far were focused on *V1R* gene repertoires in superterranean rodents, little is known about evolution of *V1R* repertoires in subterranean rodents.

Subterranean rodents live in underground burrows and spend most of their life below the soil surface [15]. Due to the limited space in burrows, chemical communication was thought to be confined in subterranean rodents [15]. However, there was evidence supporting that subterranean rodents may use pheromonal communication [16]. To study the evolution of *V1R*s in subterranean rodents, we examined *V1R* evolution by analyzing currently available genomes of 24 rodents. We next sequenced 22 intact *V1R* genes in 29 individuals of a subterranean rodent species (*Spalax galili*) from two soil populations, with the aim of testing functional importance of *V1R*s in natural populations of subterranean rodents.

## Results

### Genome mining

**Fig. 1:**
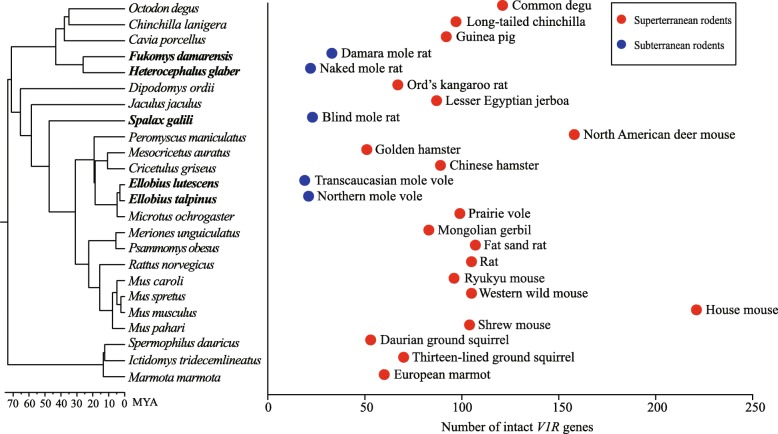
Repertoire sizes of intact *V1R*s in 24 rodents. The phylogenetic relationship of 24 rodents was obtained from multiple sources (Additional file 1: Table S2). Based on their lifestyles, these rodents are divided into two groups: superterranean rodents (red) and subterranean rodents (blue)

We identified *V1R* genes in 24 currently available rodent genome sequences, including five subterranean and 19 superterranean species (Fig. 1). A total of 4763 *V1R* genes were identified in the present study, including 2244 putatively functional *V1R* genes and 2519 pseudogenes (Additional file 1: Table S1). We divided putatively functional genes into two categories: intact genes and partial genes. The former contain an intact open reading frame (ORF) and a complete coding region, whereas the latter retain an intact ORF and a partial coding region due to incomplete sequencing [17–21]. These 2244 putatively functional *V1R* genes include 1983 intact genes (mean 83, median 88) and 261 partial genes (mean 11, median 9) (Additional file 1: Table S1). The total number of putatively functional *V1R*s in each species varied from 22 in the naked mole rat (*Heterocephalus glaber*) to 221 in the mouse (*Mus musculus*) (Additional file 1: Table S1). Notably, subterranean rodents (mean 24, median 22) tend to have a lower number of intact *V1R* genes than their superterranean relatives (mean 98, median 96) (Fig. 1). The same tendency was also observed when both intact and partial *V1R*s were considered (Additional file 1: Table S1). The reduction of *V1R* genes has occurred repeatedly in five subterranean rodents from three different lineages (Fig. 1), suggestive of convergent reduction. In addition, we constructed a gene tree (Additional file 2: Figure S1) using all intact *V1R*s from the 24 species of rodents. Our phylogenetic analyses revealed that different subterranean lineages with similar sizes of small gene repertoires tend to retain different gene groups (Additional file 2: Figure S1).

### Phylogenetically independent contrast (PIC) analysis

**Fig. 2:**
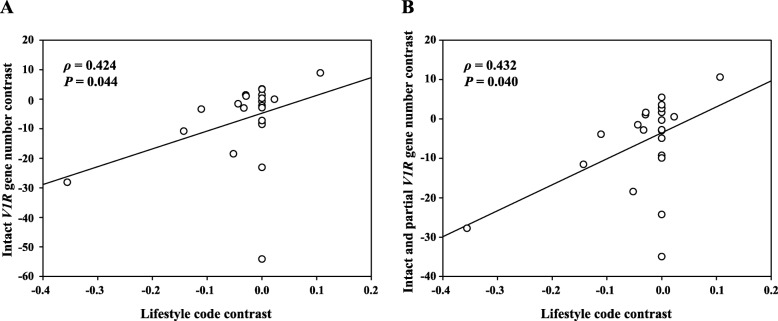
Convergent reduction of functional *V1R* genes in subterranean rodents. **a** Phylogenetically independent contrast (PIC) in intact *V1R* gene number is positively correlated with that in lifestyle code. **b** PIC in intact and partial *V1R* gene number remains positively correlated with that in lifestyle code. The lifestyle in each animal was coded as 0 (subterranean rodent) or 1 (superterranean rodent). The Spearman’s rank correlation coefficient (ρ) with a two-tailed *P* value was used to evaluate the association

To determine whether the reduction of *V1R* genes has resulted from the subterranean lifestyle shared by the five species, we performed phylogenetically independent contrast (PIC) analysis based on the *V1R* genes identified from 24 rodent species (Additional file 1: Figure S2, Table S2). Due to the tendency of fewer *V1R* genes in subterranean rodents, we coded the lifestyle of a rodent as 0 (subterranean species) or 1 (superterranean species). We converted the lifestyle codes and the *V1R* gene numbers into phylogenetically independent contrasts (PICs) [22] and subsequently conducted a regression analysis between lifestyle code contrasts and gene number contrasts. This analysis revealed a significant positive correlation between the PICs of the intact *V1R* gene numbers and those of lifestyle codes (Spearman’s ρ = 0.424, *P* = 0.044; Fig. 2a). The same trend was identified when the PICs of the total numbers of putatively functional *V1R*s (intact and partial genes) were correlated with the PICs of lifestyle codes (Spearman’s ρ = 0.432, *P* = 0.040; Fig. 2b). We also performed PIC analysis based on the *V1R* genes identified from 18 species that were sequenced on the Illumina platform and obtained similar results (Additional file 1: Figure S3). These findings strongly suggest that the lifestyle impacts the repertoire size of functional *V1R*s, with a smaller size in subterranean rodents relative to their superterranean relatives (Fig. 1, Additional file 1: Table S1).

### Population genetic differentiation of *V1R*s in *Spalax galili*

**Fig. 3:**
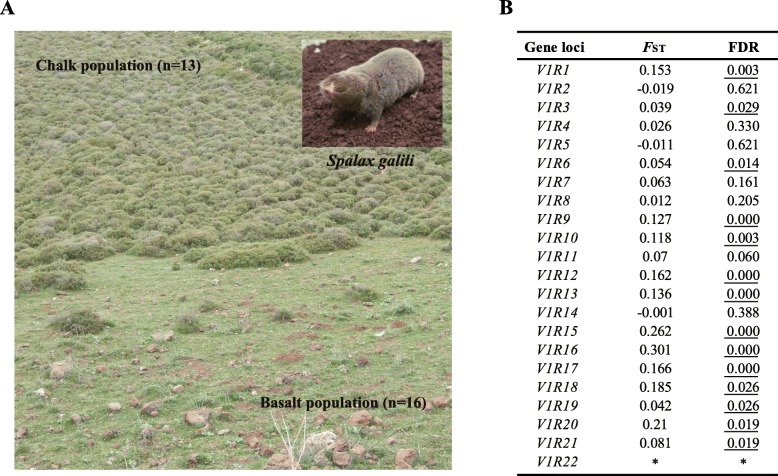
Study subject and population differentiation of *V1R*s. (A) The blind mole rat *Spalax galili* inhabiting the chalk and basalt areas; Thirteen and 16 animals were sampled from the chalk and basalt soils, respectively. The images of **a** were adapted from our previous study [23]. **b** Genetic differentiation of 22 *V1R*s between the two soil populations. *F*_ST,_ fixation index; FDR, *P*-value adjusted by false discovery rate (FDR). Significant *P*-values were underlined. *, *F*_ST_ was not able to be estimated because there is no polymorphism site in *V1R22*. Gene loci with *P*-values less than 0.05 are significantly differentiated between the two populations

To characterize the evolution of those survival *V1R*s in subterranean rodents, we performed population genetic analyses of *V1R*s between two natural populations of one subterranean rodent species (the blind mole rat, *S. galili*) (Fig. 3a). Our samples were collected in a recent study [23], including 29 individuals of *S. galili* from two soil populations (16 from the basalt and 13 from the chalk) (Fig. 3a). From the publicly available genome of *S. galili* (GenBank assembly GCF_000622305.1) [24], we identified 23 putatively functional *V1R* genes with complete coding regions and intact ORFs. Twenty-two of the 23 genes were successfully amplified and sequenced in the 29 individuals of *S. galili* (Fig. 3b, Additional file 1: Table S3). To test whether these *V1R* genes have undergone relaxed selection, we compared *V1R* genes with neutrally evolved sequences, which were taken from a recent study [23]. These sequences that were assumed to be under neutral evolution were 18 randomly selected noncoding regions, which were sequenced in the same 29 mol rats [23]. We identified single nucleotide polymorphisms (SNPs) in each locus from each animal but did not observe any SNPs that are fixed within either population, suggesting that the two populations diverged very recently. In line with this finding, genomic evidence has estimated a divergence time of 0.2–0.4 million years ago between the two populations [23].

The intra-population genetic variations of 22 *V1R* genes and 18 noncoding regions are presented in Additional file 1 Table S4 and Table S5, respectively. We found the average number of nucleotide differences per site (π) at the 18 noncoding regions to be same in both populations (0.0014) (Additional file 1: Table S5). By contrast, *V1R*s showed an overall higher π in the chalk population (0.0018) than in the basalt population (0.0014) (Additional file 1: Table S4), although the difference is not statistically significant (*P* = 0.24, two-tailed paired *t*-test). After comparing with the noncoding regions, we found *V1R*s to have a same π (0.0014) in the basalt population but an overall higher π (0.0018) in the chalk population (*P* = 0.45, two tailed Mann-Whitney U test) (Additional file 1: Tables S4, S5). We also calculated the Watterson’s polymorphism per site (θ) in *V1R*s and noncoding regions, and obtained similar results to the estimates of π (Additional file 1: Tables S4, S5). Tajima’s neutrality test [25] was used to assess whether a *V1R* gene or a noncoding region fit the neutral theory model. The rejection of the null hypothesis of neutrality suggests a non-neutral process of sequence evolution, including purifying selection, positive selection, and demographic changes [26]. We found zero *V1R* and four noncoding regions that showed significantly positive or negative Tajima’s *D* (Additional file 1: Table S4, S5), suggesting all *V1R*s fit the neutral theory model and the majority of noncoding regions also fit. In the basalt population, one noncoding region showed significantly positive *D*, whereas two noncoding regions showed significantly negative *D* (Additional file 1: Table S5). In the chalk population, only one noncoding region showed a significantly negative *D* (Additional file 1: Table S5). However, we did not identify a significant difference between the basalt (3/18 = 16.7%) and chalk (1/18 = 5.6%) populations in the fraction of noncoding regions with significantly positive or negative *D* (*P* > 0.6, Fisher’s exact test). In either population, the fraction of loci with significantly positive or negative *D* in *V1R*s (Additional file 1: Table S4) is not significantly different from that in noncoding regions (Additional file 1: Table S5) (*P* = 0.23 for the basalt and *P* = 1 for the chalk population, Fisher’s exact test). We also performed Fu and Li’s neutrality test [27] and found similar results to Tajima’s test. Neither population shows a significant difference between noncoding regions and *V1R*s in the fraction of loci with a significantly different Fu and Li’s *D* (Additional file 1: Tables S4, S5) (*P* = 0.16 for the basalt and *P* = 0.65 for the chalk population, Fisher’s exact test).

The genetic differentiations between the chalk and basalt populations were measured as the fixation index (termed *F*_ST_). Pairwise *F*_ST_ statistics between the two populations are provided in Fig. 3b. We found that 14 *V1R*s are significantly differentiated between populations (*P* < 0.05 after false discovery rate (FDR) adjustment) (Fig. 3b). Only one noncoding region was found to be significantly differentiated between populations (*P* < 0.05 after FDR adjustment) [23]. The percentage of significantly differentiated loci is statistically greater for *V1R*s (14/22 = 63.6%) than for noncoding regions (1/18 = 5.6%) (*P* < 0.001, Fisher’s exact test), suggesting that positive selection may have shaped the *V1R* evolution. We also compared the frequency of each nonsynonymous SNP in the chalk population with that in the basalt population, with the aim to detect significantly differentiated nonsynonymous SNPs that may be of functional importance. Briefly, we used Fisher’s exact test to examine the difference of the frequency of each nonsynonymous SNP between the two populations. Nonsynonymous SNPs with adjusted *P*-values less than 0.05 were considered significantly differentiated nonsynonymous SNPs (Additional file 1: Table S6). We found 12 of 22 *V1R*s to have at least 1 significantly differentiated nonsynonymous SNP, with the number ranging from 1 to 6 (Additional file 1: Figure S4). With one exception, the 12 *V1R*s in this analysis could also be identified by the *F*_ST_ analysis (Fig. 3b). The exception is *V1R6*, which showed overall genetic differentiation between populations (Fig. 3b) but did not have nonsynonymous SNPs (Additional file 1: Figure S4). We further examined the distributions of these significantly differentiated nonsynonymous SNPs on each *V1R* gene by predicting V1R transmembrane protein topologies (Additional file 1: Table S6). Our prediction revealed that most of these SNPs occurred in putative ligand binding sites [28–30]. These findings unambiguously demonstrate nonsynonymous substitutions contribute to the genetic and functional divergence of *V1R*s in the two natural populations of the subterranean rodent species.

## Discussion

Our data show that the number of functional *V1Rs* was markedly reduced in 5 subterranean rodents from 3 different lineages. A previous study has identified a positive correlation between the number of intact *V1R*s and the complexity of vomeronasal organ (VNO) morphology across mammals [11]. Our results are consistent with the known information about VNO morphology of studied rodents. For example, *Heterocephalus glaber*, one of the five subterranean rodents in this study, has a small and growth-deficient VNO [31], while other studied superterranean rodents, like *Mus musculus*, *Microtus ochrogaster*, *Rattus norvegicus* and *Chinchilla laniger*, have a well-developed VNO [32–35]. Combined our genetic data with previous morphological evidence, we concluded that pheromonal olfaction mediated by *V1Rs* is commonly reduced in subterranean rodents. In addition, the small *V1R* repertoire in subterranean rodents can not be compensated by a large *V2R* repertoire, because the majority of the *V2R* repertoires in *Spalax galili* and *H. glaber* are characterized by pseudogenes [36]. Thus, we speculate that the function of VNS in subterranean rodents is less important than their superterranean relatives. By contrast, the MOS, which mainly expresses olfactory receptors (ORs), was expected to play a more important role in African mole-rats. Specifically, the naked mole rat possesses 1401 functional *OR*s, which are around three times more than its superterranean relative, the guinea pig [37]. Thus, *OR*s seem to have shown an evolutionary pattern distinct from *V1R*s in rodents.

The observation of reduction or complete loss of *V1R* repertoire in mammals may have resulted from a number of ecological factors, such as a sensory tradeoff between vision and pheromonal olfaction in hominids and Old World monkeys [38], and an aquatic lifestyle in dolphins [12]. Why did the reduction of *V1R* repertoire occur in subterranean rodents? Previous studies suggested that chemical signals are relatively less important than vibrational signals in these animals [15]. In other words, living in the underground burrow confined the dispersal of chemical signals, whereas vibrational signals can spread beyond the confines of underground burrow [15]. We thus infer that selective pressures acting on *V1R* repertoire may have been relaxed because of ineffective chemical communication in subterranean rodents. In addition, we also identified the *V1R* gene repertoires in four species of soricomorphans, including one subterranean species (*Condylura cristata*, star-nosed mole) and three superterranean relatives (*Erinaceus europaeus*, *Solenodon paradoxus*, *Sorex araneus*). We found that the star-nosed mole possesses the fewer intact *V1R* genes (*n* = 15) compared with its superterranean relatives (*n* = 56 in *E. europaeus* and *n* = 54 in *S.araneus*), which is consistent with the overall pattern in rodents (Fig. 1), although the Hispaniolan solenodon (*S. paradoxus*) carries the fewest number of intact *V1R*s (*n* = 6) (Additional file 1: Table S1).

The substantial reduction of functional *V1R*s does not represent nonfunctional pheromonal olfaction in subterranean rodents, because considerable evidence has suggested that pheromone cues are used in subterranean rodents such as *Spalax*, *Heterocephalus*, ctenomyids, and bathyergids [39]. Indeed, subterranean rodents have been assumed to rely on chemical cues, because life underground deprives them of visual cues, which otherwise represent an important type of sensory stimuli in mammals [15]. In addition, pheromones are relatively easy to synthesize and are also long-lasting cues [40], which may provide an inexpensive mode of communication for subterranean rodents coping with multiple stressors such as darkness, energetics, hypoxia, and hypercapnia [24]. Our population genetic data include intra-population polymorphisms and inter-population variations. The intra-population polymorphisms in *V1R*s are similar to those in noncoding regions, suggesting that *V1R* evolution is largely neutral within either population of the blind mole rats. In accordance with our study, earlier studies in humans [41], mice [26, 42], and other mammals [12] also revealed that genetic drift plays an important role in *V1R* evolution. By contrast, our inter-population analysis (*F*_ST_) showed significantly greater genetic differentiation of *V1R*s than neutrally evolving noncoding regions, with the fraction of significantly differentiated loci much higher for *V1R*s (14/22) than for noncoding regions (1/18). Furthermore, a number of significantly differentiated nonsynonymous SNPs were observed in the significantly differentiated *V1R*s between populations, implying that diversifying selection may also play an important role in *V1R* functional changes. Thus, the microevolution of *V1R*s in *S. galili* is at least in part shaped by natural selection, supporting the hypothesis that *V1R*s may be involved in reproductive isolation of *S. galili*. In addition, several studies have suggested that pheromonal olfaction mediated by the VNS is involved in *Spalax* reproductive isolation: *S. galili* exhibits mate choice by choosing mates with similar genetically determined odors [43]; A behavioral test demonstrated that reproductive isolation associated with olfaction plays a major role in *Spalax* speciation across Israel [44]; Olfactory discrimination was observed to serve as a reproductive isolating mechanism in *Spalax* speciation [45]; Pheromones identified from the urine of male mole rats were found to be involved in sexual attractance [46]. Together, our study shows that functional *V1R*s in subterranean rodents are significantly reduced in number and may remain important in some species of subterranean rodents.

## Conclusion

Our study suggests that the subterranean or superterranean lifestyle shaped the evolution of *V1R*s in rodents. Subterranean rodents lived in underground burrows have a smaller *V1R* repertoire than their superterranean relatives. However, we noticed that the small *V1R* repertoire of *Spalax galili* are under diversifying selection between two populations, indicative of functional significance of these genes. It would be interesting to test whether *V1R*s remain significant genetic differentiation in other populations or species of subterranean rodents.

## Methods

### Identification of *V1R* repertoires

A total of 24 rodent genome sequences were retrieved from the National Center for Biotechnology Information database (http://www.ncbi.nlm.nih.gov/). Of them, five species are subterranean, while the remaining 19 species are superterranean (Additional file 1: Table S1). The species names and genome accession numbers are given in Additional file 1: Table S1. Because vertebrate *V1R*s are single-exon genes encoding vomeronasal type 1 receptors with seven transmembrane domains [6], we generally followed two previous studies [11, 20] to identify the *V1R* gene repertoires in the 24 rodents. Briefly, TblastN searches [47] were conducted on the genome sequences using phylogenetically diverse *V1R*s that were obtained from previous studies, including full-length *V1R* genes from the mouse, rat, cow, dog, two fishes, and one *ancV1R* of *Hippopotamus amphibius* [11, 12, 48–50] as queries, with a cutoff e-value of 1e-5. The blast hits shorter than 300 bp were discarded, and the remaining blast hits were extended in both directions to acquire the start or stop codons. All full-length candidate genes were confirmed for the presence of seven-transmembrane domains using the TMHMM [51] or HMMTOP method [52]. A putative gene was considered to be real when the BLASTN searches against the non-redundant database of GenBank yielded the best hit being the known *V1R* gene. All candidate genes were classified into three categories: intact genes, partial genes and pseudogenes. Intact genes contain at least 270 codons, a putative start and stop codon, and an intact ORF; Partial genes contain more than 300 nucleotides, a putative start or a stop codon, and a truncated ORF due to incomplete sequencing or poor genomic assembly. Pseudogenes contain more than 300 nucleotides and an interrupted ORF because of nonsense or frameshift mutations. We conducted BLASTP searches [47] against non-redundant database of GenBank, and chose the best hit as the basis of nomenclature of rodent *V1R*s. By contrast, we named the 22 *V1R* genes from *S. galili* numerically with the order in which they were identified from the genome, because 16 of them were annotated as *V1R4* in GenBank and 4 of them as *V1R1* (Additional file 1: Table S3). For simplicity and convenience, we used *V1R1-V1R22* to name the 22 *V1R* genes in *Spalax galili*. All intact *V1R* genes newly identified in this study were provided in Additional file 3. The information about gene location and orientation on the scaffolds for each *V1R* was provided in the Additional file 4. The phylogenetic tree (Additional file 2: Figure S1) of all intact *V1R* sequences was reconstructed using the Bayesian method [53] with 6 million generations, and *hTAS2R1* (GenBank accession number: NM_019599.2) was used as an outgroup.

### Phylogenetically independent contrast analysis

To assess the potential impact of the lifestyle categories (subterranean or superterranean lifestyle) on the evolution of *V1R*s in rodents, we conducted a regression analysis of *V1R* gene number against the lifestyle category following a recent study [20]. Briefly, we coded each rodent as 0 (subterranean species) and 1 (superterranean species) by assuming that subterranean rodents may contain fewer *V1R*s than their superterranean relatives. We conducted a phylogenetically independent contrast (PIC) analysis using the package APE (Analyses of Phylogenetics and Evolution) [54]. The PIC method was used to quantify the correlation between *V1R* gene number and the lifestyle after eliminating the confounding effects of phylogenetic inertia. The input tree (Additional file 1: Figure S2) is the established species tree [55], and the branch lengths of the input tree were estimated by divergence times (Additional file 1: Table S2). The regression analysis was performed after converting the lifestyle codes and *V1R* gene numbers into PICs. The correlation between the lifestyle and *V1R* gene number was assessed by the nonparametric Spearman’s rank correlation coefficient (ρ).

### DNA sequencing and population genetic analysis

By searching the published genome sequence of the blind mole rat *Spalax galili* (GenBank assembly: GCF_000622305.1) [24], we identified 23 full-length *V1R*s with an intact open reading frame, suggesting that they are putatively functional. A suite of primers (Additional file 1: Table S3) were designed to amplify all *V1R* genes except for the *ancV1R* based on the corresponding flanking sequences. A total of 29 individuals of *S. galili*, 16 from the basalt soil and 13 from the chalk soil, were captured alive near Rehaniya, Upper Galilee Mountains in northern Israel (33°02.5′N, 35°29.2′E) in a previous study [23]. Animals were euthanized by injecting Ketaset CIII at 5 mg/kg of body weight. Muscle tissues were sampled and then stored in 95% (vol/vol) ethanol to be kept at − 80 °C until DNA extraction. Genomic DNAs were isolated from the muscle tissue using the DNeasy Blood and Tissue Kit (Qiagen). PCRs (polymerase chain reactions) were processed with high-fidelity KOD-Plus-Neo DNA polymerase (Toyobo), and details of PCR amplication were described previously [17–19, 21, 56]. PCR products were purified by the QIAquick PCR Purification Kit (Qiagen) and sequenced directly from both strands. A total of 1276 sequences that were newly obtained were deposited in GenBank with accession numbers MF118921-MF120196, and we included both alleles for each gene in the data deposition. All sequences were edited and aligned by MEGA6 [57]. Nucleotide diversity π, a measure of genetic variation, is defined as the average number of nucleotide differences per site [58]. Another important parameter of genetic diversity is Watterson’s θ, which was estimated by the number of segregating sites [59]. Tajima’s D and Fu and Li’s D* [25, 27] are classical statistical tests to assess whether DNA sequences evolve neutrally. The significance of Tajima’s D test or Fu and Li’s D* test was conducted by 10,000 replicates of coalescent simulation. The population genetic differentiation of each gene between the basalt and chalk populations was measured by the fixation index (termed *F*_ST_) [60]. The nearest-neighbor statistic(Snn) test [61] was conducted to evaluate the significance level of the genetic differentiation between the two populations. The estimates of significance level were corrected for multiple comparisons using the FDR adjustment. All these parameters and statistical tests of population genetic differentiation were computed and performed using DnaSP v5.10 [62].

## Additional files

Additional file 1: **Table S1.** Numbers of *V1R* genes identified from the genome assemblies of 28 mammals. Twenty-four species of rodents and four species of soricomorphans (shaded) were included. The subterranean species were shown in bold. **Table S2.** Node ages in the species tree depicted in the Additional file 1: Figure S2. **Table S3.** Primers used for amplification**. Table S4.** Intra-population genetic variations of 22 intact *V1R* genes in the two soil populations of *Spalax galili*. **Table S5.** Intra-population genetic variations of the 18 noncoding regions in the two soil populations of *Spalax galili*. **Table S6.** All significantly differentiated nonsynonymous SNPs between populations in each of the 22 *V1R* genes. Ten *V1R*s were left blank and shown in gray due to the lack of significantly differentiated nonsynonymous SNPs. **Figure S2.** The species tree of 24 rodents in this study. The letters (A-W) indicate the nodes of the tree. Branch lengths were estimated from the divergence times among species (Table S2). **Figure S3**. PIC analysis based on the *V1R*s identified from 18 species that were sequenced on the Illumina platform, with the aim to avoid biases resulting from different sequencing platforms. **Figure S4.** Number of significantly differentiated nonsynonymous SNPs between populations in each of the 12 *V1R*s. (DOCX 545 kb)

Additional file 2: **Figure S1.** The Bayesian tree based on the alignment of 1983 intact *V1R*s. (PDF 3369 kb)

Additional file 3: Nucleotide sequences of intact *V1R* genes identified from 28 mammalian genomes in the present study. These genes were named following the best hits of BLASTP searches. (DOCX 683 kb)

Additional file 4: Genomic locations for all annotated *V1R *genes studied. (TXT 579 kb)

## Data Availability

All DNA sequences of *Spalax V1R*s generated in this study can be found under the GenBank accession numbers: MF118921-MF120196. The accession numbers for all other genetic data used in this study has been included in the manuscript and its additional files.

## Abbreviations

FDR: False discovery rate
FPR: Formyl peptide receptor gene
MOS: Main olfactory system
OR: Olfactory receptor
ORF: Open reading frame
PCR: Polymerase chain reaction
PIC: Phylogenetically independent contrast
Snn test: Nearest-neighbor statistic test
SNP: Single nucleotide polymorphism
V1R: Vomeronasal type 1 receptor
V2R: Vomeronasal type 2 receptor
VNO: Vomeronasal organ
VNS: Vomeronasal system
