# Supplementary material for: Convergent reduction of V1R genes in subterranean rodents
Source: BMC Evol Biol. 2019 Aug 30;19:176. doi: 10.1186/s12862-019-1502-4 (PMC6717356; doi:10.1186/s12862-019-1502-4)
Supplement: Supplementary file 1 — Table S1. Numbers of V1R genes identified from the genome assemblies of 28 mammals. Twenty-four species of rodents and four species of soricomorphans (shaded) were included. The subterranean species were shown in bold. Table S2. Node ages in the species tree depicted in the Additional file 1: Figure S2. Table S3. Primers used for amplification. Table S4. Intra-population genetic variations of 22 intact V1R genes in the two soil populations of Spalax galili. Table S5. Intra-population genetic variations of the 18 noncoding regions in the two soil populations of Spalax galili. Table S6. All significantly differentiated nonsynonymous SNPs between populations in each of the 22 V1R genes. Ten V1Rs were left blank and shown in gray due to the lack of significantly differentiated nonsynonymous SNPs. Figure S2. The species tree of 24 rodents in this study. The letters (A-W) indicate the nodes of the tree. Branch lengths were estimated from the divergence times among species (Table S2). Figure S3. PIC analysis based on the V1Rs identified from 18 species that were sequenced on the Illumina platform, with the aim to avoid biases resulting from different sequencing platforms. Figure S4. Number of significantly differentiated nonsynonymous SNPs between populations in each of the 12 V1Rs. (DOCX 545 kb) [file 12862_2019_1502_MOESM1_ESM.docx]

**Additional file 1**

**Table S1. Numbers of *V1R* genes identified from the genome assemblies of 28 mammals.** Twenty-four species of rodents and four species of soricomorphans (shaded) were included. The subterranean species were shown in bold.

| **Species name** | **Assembly version** | **Contig N50 (bp)** | **Intact** | **Partial** | **Pseudogene** | **Functional** | **Total** |
| --- | --- | --- | --- | --- | --- | --- | --- |
| *Cavia porcellus* | GCF_000151735.1_Cavpor3.0 | 80583 | 92 | 2 | 160 | 94 | 254 |
| *Chinchilla lanigera* | GCF_000276665.1_ChiLan1.0 | 61105 | 97 | 8 | 64 | 105 | 169 |
| *Cricetulus griseus* | GCF_000223135.1_CriGri_1.0 | 39361 | 89 | 19 | 94 | 108 | 202 |
| *Dipodomys ordii* | GCF_000151885.1_Dord_2.0 | 48087 | 67 | 4 | 157 | 71 | 228 |
| ***Ellobius lutescens*** | GCA_001685075.1_ASM168507v1 | 11648 | 19 | 9 | 53 | 28 | 81 |
| ***Ellobius talpinus*** | GCA_001685095.1_ETalpinus_0.1 | 8259 | 21 | 19 | 47 | 40 | 87 |
| ***Fukomys damarensis*** | GCF_000743615.1_DMR_v1.0 | 44830 | 33 | 1 | 46 | 34 | 80 |
| ***Heterocephalus glaber*** | GCF_000247695.1_HetGla_female_1.0 | 47778 | 22 | 0 | 36 | 22 | 58 |
| *Ictidomys tridecemlineatus* | GCF_000236235.1_SpeTri2.0 | 44137 | 70 | 14 | 147 | 84 | 231 |
| *Jaculus jaculus* | GCF_000280705.1_JacJac1.0 | 15675 | 87 | 9 | 58 | 96 | 154 |
| *Marmota marmota* | GCF_001458135.1_marMar2.1 | 66492 | 60 | 3 | 112 | 63 | 175 |
| *Meriones unguiculatus* | GCF_002204375.1_MunDraft-v1.0 | 46546 | 83 | 28 | 102 | 111 | 213 |
| *Mesocricetus auratus* | GCF_000349665.1_MesAur1.0 | 22511 | 51 | 14 | 72 | 65 | 137 |
| *Microtus ochrogaster* | GCF_000317375.1_MicOch1.0 | 21250 | 99 | 13 | 65 | 112 | 177 |
| *Mus caroli* | GCF_900094665.1_CAROLI_EIJ_v1.1 | 30916 | 96 | 17 | 136 | 113 | 249 |
| *Mus musculus* | GCF_000001635.25_GRCm38.p5 | 32273079 | 221 | 0 | 212 | 221 | 433 |
| *Mus pahari* | GCF_900095145.1_PAHARI_EIJ_v1.1 | 29465 | 104 | 14 | 121 | 118 | 239 |
| *Mus spretus* | GCA_001624865.1_SPRET_EiJ_v1 | 17887 | 105 | 41 | 178 | 146 | 324 |
| *Octodon degus* | GCF_000260255.1_OctDeg1.0 | 19847 | 121 | 7 | 71 | 128 | 199 |
| *Peromyscus maniculatus* | GCF_000500345.1_Pman_1.0 | 36367 | 158 | 15 | 141 | 173 | 314 |
| *Psammomys obesus* | GCA_002215935.1_ASM221593v1 | 76398 | 107 | 5 | 98 | 112 | 210 |
| *Rattus norvegicus* | GCF_000001895.5_Rnor_6.0 | 100461 | 105 | 6 | 114 | 111 | 225 |
| ***Spalax galili*** | GCF_000622305.1_S.galili_v1.0 | 30353 | 23 | 0 | 75 | 23 | 98 |
| *Spermophilus dauricus* | GCA_002406435.1_ASM240643v1 | 34849 | 53 | 13 | 160 | 66 | 226 |
| ***Condylura cristata*** | GCF_000260355.1_ConCri1.0 | 46163 | 15 | 0 | 11 | 15 | 26 |
| *Erinaceus europaeus* | GCF_000296755.1_EriEur2.0 | 21359 | 56 | 6 | 48 | 62 | 110 |
| *Solenodon paradoxus* | GCA_002901085.1_ASM290108v1 | 89121 | 6 | 0 | 46 | 6 | 52 |
| *Sorex araneus* | GCF_000181275.1_SorAra2.0 | 22623 | 54 | 6 | 40 | 60 | 100 |

**Table S2. Node ages in the species tree depicted in the Figure S2.**

| **Node** | **Divergence times millions of years ago, MYA** | **References** |
| --- | --- | --- |
| A | 73.4 | Fang et al. 2014a |
| B | 71.1 | Fang et al. 2014a |
| C | 43.3 | Voloch et al. 2013 |
| D | 37.9 | Voloch et al. 2013 |
| E | 35 | Voloch et al. 2013 |
| F | 26 | Fang et al. 2014b |
| G | 65.4 | Fang et al. 2014a |
| H | 58.51 | Pisano et al. 2015 |
| I | 47.4 | Fang et al. 2014a |
| J | 31 | Fang et al. 2014a |
| K | 19.2 | Steppan et al. 2004 |
| L | 18.5 | Steppan et al. 2004 |
| M | 10.8 | Neumann et al. 2006 |
| N | 4.7 | Abramson et al. 2009 |
| O | 3 | Mulugeta et al. 2016 |
| P | 22.5 | Steppan et al. 2004 |
| Q | 5.51 | Chevret and Dobigny 2005 |
| R | 15.6 | Fang et al. 2014a |
| S | 7.6 | Veyrunes et al. 2005 |
| T | 4.8 | Veyrunes et al. 2005 |
| U | 2.3 | Veyrunes et al. 2005 |
| V | 13.4 | Harrison et al. 2003 |
| W | 12.5 | Harrison et al. 2003 |

Abramson NI, Lebedev VS, Bannikova AA, Tesakov AS. Radiation events in the subfamily Arvicolinae (Rodentia): evidence from nuclear genes. Doklady Biological Sciences. 2009; 428: 458–461.

Chevret P, Dobigny G. Systematics and evolution of the subfamily Gerbillinae (Mammalia, Rodentia, Muridae). Molecular Phylogenetics and Evolution. 2005; 35:674-688.

Fang X, Nevo E, Han LJ, Levanon EY, Zhao J, Avivi A, Larkin D, Jiang XT, Feranchuk S, Zhu YB et al. Genome-wide adaptive complexes to underground stresses in blind mole rats *Spalax*. Nature Communcations. 2014a; 5:3966.

Fang X, Seim I, Huang ZY, Gerashchenko MV, Xiong ZQ, Turanov AA, Zhu YB, Lobanov AV, Fan DD, Yim SH et al. Adaptations to a subterranean environment and longevity revealed by the analysis of mole rat genomes. Cell Reports. 2014b; 8:1354-1364.

Harrison RG, Bogdanowicz SM, Hoffmann RS, Yensen E, Sherman PW. Phylogeny and evolutionary history of the ground squirrels (Rodentia: Marmotinae). Journal of Mammalian Evolution. 2003; 10:249-276.

Mulugeta E, Wassenaar E, Sleddens-Linkels E, van Ijcken WFJ, Heard E, Grootegoed JA, Just W, Gribnau J, Baarends WM. Genomes of *Ellobius* species provide insight into the evolutionary dynamics of mammalian sex chromosomes. Genome Research. 2016; 26:1202-1210.

Neumann K, Michaux J, Lebedev V, Yigit N, Colak E, Ivanova N, Poltoraus A, Surov A, Markov G, Maak S et al. Molecular phylogeny of the Cricetinae subfamily based on the mitochondrial cytochrome b and 12S rRNA genes and the nuclear vWF gene. Molecular Phylogenetics and Evolution. 2006; 39:135-148.

Pisano J, Condamine FL, Lebedev V, Bannikova A, Quere JP, Shenbrot GI, Pages M, Michaux JR. Out of Himalaya: the impact of past Asian environmental changes on the evolutionary and biogeographical history of Dipodoidea (Rodentia). Journal of Biogeography. 2015; 42:856-870.

Steppan SJ, Adkins RM, Anderson J. Phylogeny and divergence-date estimates of rapid radiations in muroid rodents based on multiple nuclear genes. Systematic Biology. 2004; 53:533-553.

Veyrunes F, Britton-Davidian J, Robinson TJ, Calvet E, Denys C, Chevret P. Molecular phylogeny of the African pygmy mice, subgenus Nannomys (Rodentia, Murinae, Mus): Implications for chromosomal evolution. Molecular Phylogenetics and Evolution. 2005; 36:358-369.

Voloch CM, Vilela JF, Loss-Oliveira L, Schrago CG. Phylogeny and chronology of the major lineages of New World hystricognath rodents: insights on the biogeography of the Eocene/Oligocene arrival of mammals in South America. BMC Research Notes. 2013; 6:160.

**Table S3. Primers used for amplification.**

| Gene | GenBank annotation | Forward primer (5’-3’) | Reverse primer (5’-3’) |
| --- | --- | --- | --- |
| *V1R1* | *V1R4* | TGGACATACGGACCAGTGAA | AGGATGGAGGAAGTGTTCACA |
| *V1R2* | *V1R54* | TGGGATGCATCAACGTGTTC | TCACAAACCCCACACCCTTA |
| *V1R3* | *V1R4* | AGGTCCCCATGTAATGTGCA | GCTCTGTTCACCTCCGAGAT |
| *V1R4* | *V1R4* | CTCAGTGCTTCTCCTGTCCC | GTAGCTTCCTCCAGTCACCT |
| *V1R5* | *V1R4* | GCAGCTATGAACCGTCCTTA | TGCGTCTCATGCTGTAATTGT |
| *V1R6* | *V1R4* | CAATTTGGTTCACACTGGCTTT | ACCTTCCTCACACTTCTCTGT |
| *V1R7* | *V1R4* | CAAAACCTGCTCTTGACGCT | ACAAGTCCCAATGTGACAACC |
| *V1R8* | *V1R4* | GCTCACCAACCTTCTCTCCT | TGAAGTCTAACTGGGGCTGG |
| *V1R9* | *V1R4* | CTCTCCAGGCCCTGCTTTAT | TGGGTCCTCTGCACAAGTAG |
| *V1R10* | *V1R4* | AAAGAGCCCTCCTTGACTGG | GCGTGATGGGTAAGCAACAA |
| *V1R11* | *V1R4* | CACAGTGCCAAGACACTTTGA | GGGTCTGGATTTGAGTGGGA |
| *V1R12* | *V1R1* | ACTGAAACTGAAGGCCATACA | TGAGAAATGCTAGCAAAACAGC |
| *V1R13* | *V1R4* | GGCCTTGAACACATGAGCTC | TGGACACAATGGACTCACGA |
| *V1R14* | *V1R1* | AGATCTTCATGGGGCTGTGT | CAGTTGTTCTTGTTTTCGATGGT |
| *V1R15* | *V1R1* | CCTGTGCGTTTTCAAAGTCTC | CCTGCCTCTGTCCAAATCAC |
| *V1R16* | *V1R4* | TGCACACACATAAGCACATACA | ACACAAACCTCTAGACTTGCA |
| *V1R17* | *V1R1* | ACCCCATTTCAATATTTCCCGG | ACAGGCTGGTAGTTCTATGGG |
| *V1R18* | *V1R90* | CCGTTTCTGTTGTAATGTCAATGA | TGGTCTACATAGCAAGGCAC |
| *V1R19* | *V1R4* | GAAGTGTCACCTCTGAGCCT | TGGTACGGCTTGTTTCCTCA |
| *V1R20* | *V1R4* | GGGTCTCAAGTGCTCCTGAA | GGGTCATATCAGATATTCAAGTTCC |
| *V1R21* | *V1R4* | GCTCAGATTGCAACCGTCAT | ACGTTTTCCACCCCTACAGT |
| *V1R22* | *V1R4* | TCACTTCTCTTTCTCTCCAAACA | ACCCTTGAACTATCCTTAGGCT |

**Table S4.** Intra-population genetic variations of 22 intact *V1R* genes in the two soil populations of *Spalax galili*.

| **Gene Name**^1^ | **L(nt)** | **Basalt Population** | | | | |  | **Chalk Population** | | | | |
| --- | --- | --- | --- | --- | --- | --- | --- | --- | --- | --- | --- | --- |
|  |  | **S**^2^ | **π (%)**^3^ | **θ (%)**^4^ | **Tajima’s D**^5^ | **Fu and Li D***^5^ |  | **S**^2^ | **π (%)**^3^ | **θ (%)**^4^ | **Tajima’s D**^5^ | **Fu and Li D***^5^ |
| *V1R1* | 918 | 1 | 0.06 | 0.03 | 1.60 | 0.59 |  | 5 | 0.20 | 0.14 | 1.16 | 1.16 |
| *V1R2* | 924 | 1 | 0.01 | 0.03 | -0.78 | 0.59 |  | 1 | 0.02 | 0.03 | -0.31 | 0.61 |
| *V1R3* | 921 | 7 | 0.18 | 0.19 | -0.17 | 0.56 |  | 8 | 0.23 | 0.23 | -0.03 | 0.11 |
| *V1R4* | 921 | 8 | 0.28 | 0.22 | 0.82 | 0.68 |  | 8 | 0.28 | 0.23 | 0.68 | 0.72 |
| *V1R5* | 942 | 3 | 0.04 | 0.08 | -1.03 | -0.28 |  | 3 | 0.03 | 0.08 | -1.51 | -1.40 |
| *V1R6* | 1032 | 16 | 0.22 | 0.39 | -1.49 | -1.87 |  | 6 | 0.13 | 0.15 | -0.38 | -1.02 |
| *V1R7* | 906 | 2 | 0.07 | 0.06 | 0.47 | 0.80 |  | 4 | 0.11 | 0.12 | -0.05 | 1.07 |
| *V1R8* | 948 | 5 | 0.10 | 0.13 | -0.59 | 1.14 |  | 5 | 0.11 | 0.14 | -0.61 | 0.31 |
| *V1R9* | 927 | 8 | 0.25 | 0.21 | 0.53 | **1.32** |  | 8 | 0.11 | 0.23 | -1.56 | **-2.33** |
| *V1R10* | 927 | 5 | 0.11 | 0.13 | -0.50 | 1.14 |  | 6 | 0.20 | 0.17 | 0.53 | 1.23 |
| *V1R11* | 900 | 7 | 0.22 | 0.19 | 0.34 | 1.27 |  | 8 | 0.24 | 0.23 | 0.03 | 0.11 |
| *V1R12* | 924 | 1 | 0.05 | 0.03 | 1.45 | 0.59 |  | 3 | 0.15 | 0.09 | 1.87 | 0.97 |
| *V1R13* | 831 | 6 | 0.13 | 0.18 | -0.74 | 1.21 |  | 6 | 0.19 | 0.19 | -0.03 | 1.23 |
| *V1R14* | 930 | 1 | 0.03 | 0.03 | 0.15 | 0.59 |  | 3 | 0.06 | 0.09 | -0.87 | 0.97 |
| *V1R15* | 945 | 7 | 0.21 | 0.18 | 0.35 | 1.27 |  | 21 | 0.60 | 0.58 | 0.08 | 0.49 |
| *V1R16* | 915 | 2 | 0.11 | 0.05 | 1.96 | 0.80 |  | 2 | 0.05 | 0.06 | -0.32 | -0.69 |
| *V1R17* | 1017 | 6 | 0.12 | 0.15 | -0.51 | -1.15 |  | 10 | 0.26 | 0.26 | 0.06 | 0.37 |
| *V1R18* | 939 | 18 | 0.41 | 0.48 | -0.50 | 0.91 |  | 19 | 0.61 | 0.53 | 0.51 | 0.36 |
| *V1R19* | 945 | 1 | 0.05 | 0.03 | 1.60 | 0.59 |  | 3 | 0.10 | 0.08 | 0.56 | -0.22 |
| *V1R20* | 948 | 9 | 0.37 | 0.24 | 1.68 | 0.77 |  | 8 | 0.11 | 0.22 | -1.59 | **-2.94** |
| *V1R21* | 903 | 5 | 0.16 | 0.14 | 0.42 | 0.25 |  | 3 | 0.11 | 0.09 | 0.53 | -0.22 |
| *V1R22* | 903 | 0 | 0.00 | 0.00 | 0.00 | 0.00 |  | 0 | 0.00 | 0.00 | 0.00 | 0.00 |
| Mean | 930.3 | 5.4 | 0.14 | 0.14 | 0.23 | 0.54 |  | 6.4 | 0.18 | 0.18 | -0.06 | 0.04 |

Note – 1.The 22 *V1R* genes were numbered with the order in which they were identified.

2. Number of polymorphic sites.

3. The average number of nucleotide differences per site.

4. Watterson’s polymorphism per site.

5. Values significantly different from 0 at the 5% level are shown in bold and underlined. Significance is determined by 10,000 coalescent simulations. Significant values of Tajima’s *D* and Fu and Li’s *D** indicate the rejection of neutral theory model, and can result from demographic changes and selective pressures.

**Table S5: Intra-population genetic variations of the 18 noncoding regions in the two soil populations of *Spalax galili*.**

| **Region name**^1^ | **L(nt)** | **Basalt Population** | | | | |  | **Chalk Population** | | | | |
| --- | --- | --- | --- | --- | --- | --- | --- | --- | --- | --- | --- | --- |
|  |  | **S**^2^ | **π(%)**^3^ | **θ(%)**^4^ | **Tajima’s D**^5^ | **Fu and Li D***^5^ |  | **S**^2^ | **π(%)**^3^ | **θ(%)**^4^ | **Tajima’s D**^5^ | **Fu and Li D***^5^ |
| Noncoding region 1 | 953 | 9 | 0.18 | 0.23 | -0.71 | -1.59 |  | 8 | 0.19 | 0.22 | -0.40 | 0.11 |
| Noncoding region 2 | 946 | 4 | 0.08 | 0.11 | -0.64 | 1.05 |  | 2 | 0.04 | 0.06 | -0.67 | 0.83 |
| Noncoding region 3 | 1053 | 7 | 0.14 | 0.17 | -0.41 | 0.56 |  | 6 | 0.23 | 0.15 | 1.53 | 1.23 |
| Noncoding region 4 | 812 | 3 | 0.08 | 0.09 | -0.22 | -0.28 |  | 3 | 0.08 | 0.10 | -0.40 | -0.22 |
| Noncoding region 5 | 1066 | 10 | 0.35 | 0.23 | 1.60 | 0.86 |  | 11 | 0.28 | 0.27 | 0.14 | 0.00 |
| Noncoding region 6 | 927 | 4 | 0.05 | 0.11 | -1.36 | **-2.02** |  | 3 | 0.05 | 0.09 | -1.07 | 0.97 |
| Noncoding region 7 | 1003 | 3 | 0.10 | 0.07 | 0.75 | 0.94 |  | 3 | 0.08 | 0.08 | 0.14 | 0.97 |
| Noncoding region 8 | 1094 | 6 | 0.14 | 0.14 | 0.08 | 1.21 |  | 7 | 0.19 | 0.17 | 0.49 | 0.61 |
| Noncoding region 9 | 1085 | 1 | 0.02 | 0.02 | -0.14 | 0.59 |  | 3 | 0.02 | 0.07 | **-1.73** | **-2.58** |
| Noncoding region 10 | 1001 | 1 | 0.03 | 0.03 | 0.15 | 0.59 |  | 1 | 0.04 | 0.03 | 0.92 | 0.61 |
| Noncoding region 11 | 1045 | 1 | 0.01 | 0.02 | -0.78 | 0.59 |  | 1 | 0.01 | 0.03 | -0.71 | 0.61 |
| Noncoding region 12 | 1062 | 15 | 0.59 | 0.35 | **2.22** | **1.55** |  | 14 | 0.39 | 0.35 | 0.40 | -0.47 |
| Noncoding region 13 | 1022 | 11 | 0.19 | 0.27 | -0.95 | -0.09 |  | 11 | 0.21 | 0.29 | -0.91 | -0.48 |
| Noncoding region 14 | 1126 | 11 | 0.10 | 0.24 | **-1.84** | **-3.16** |  | 7 | 0.14 | 0.16 | -0.36 | **1.28** |
| Noncoding region 15 | 1150 | 3 | 0.04 | 0.07 | -1.04 | -0.28 |  | 1 | 0.01 | 0.02 | -1.16 | -1.63 |
| Noncoding region 16 | 1079 | 14 | 0.25 | 0.32 | -0.73 | -1.45 |  | 13 | 0.32 | 0.32 | 0.05 | **1.49** |
| Noncoding region 17 | 949 | 3 | 0.12 | 0.08 | 1.07 | 0.94 |  | 5 | 0.12 | 0.14 | -0.36 | -1.39 |
| Noncoding region 18 | 1069 | 5 | 0.04 | 0.12 | **-1.76** | **-2.41** |  | 4 | 0.07 | 0.10 | -0.90 | 0.09 |
| Mean | 1025 | 6.2 | 0.14 | 0.15 | -0.26 | -0.13 |  | 5.7 | 0.14 | 0.15 | -0.28 | 0.11 |

**Note –** 1.The 18 noncoding regions were numbered with the order in which they were identified.

2. Number of polymorphic sites.

3. The average number of nucleotide differences per site.

4. Watterson’s polymorphism per site.

5. Values significantly different from 0 at the 5% level are shown in bold and underlined. Significance is determined by 10,000 coalescent simulations. Significant values of Tajima’s *D* and Fu and Li’s *D** indicate the rejection of neutral theory model, and can result from demographic changes and selective pressures.

**Table S6. All significantly differentiated nonsynonymous SNPs between populations in each of the 22 *V1R* genes.** Ten *V1R*s were left blank and shown in gray due to the lack of significantly differentiated nonsynonymous SNPs.

| ***V1R* genes** | **Position^1^** | **SNP** | **Basalt^2^** | **Chalk^2^** | ***P*-value^3^** | **Adjusted *P*-value^4^** | **Domain^5^** | **Method^6^** |
| --- | --- | --- | --- | --- | --- | --- | --- | --- |
| *V1R1* | 437 | G-A | 0/32 | 6/26 | 5.69E-03 | 2.62E-02 | TM4 | TMHMM |
|  | 519 | G-T | 0/32 | 6/26 | 5.69E-03 | 2.62E-02 | EC2 |  |
|  | 637 | C-T | 0/32 | 6/26 | 5.69E-03 | 2.62E-02 | IC3 |  |
| *V1R2* |  |  |  |  |  |  |  |  |
| *V1R3* |  |  |  |  |  |  |  |  |
| *V1R4* |  |  |  |  |  |  |  |  |
| *V1R5* |  |  |  |  |  |  |  |  |
| *V1R6* |  |  |  |  |  |  |  |  |
| *V1R7* |  |  |  |  |  |  |  |  |
| *V1R8* |  |  |  |  |  |  |  |  |
| *V1R9* | 218 | C-G | 16/32 | 4/26 | 1.14E-02 | 4.83E-02 | EC1 | HMMTOP |
|  | 337 | A-G | 0/32 | 5/26 | 1.44E-02 | 4.92E-02 | IC2 |  |
| *V1R10* | 163 | C-T | 9/32 | 17/26 | 7.59E-03 | 3.35E-02 | TM2 | HMMTOP |
| *V1R11* |  |  |  |  |  |  |  |  |
| *V1R12* | 149 | T-C | 0/32 | 8/26 | 8.15E-04 | 1.36E-02 | TM2 | HMMTOP |
| *V1R13* | 518 | A-G | 3/32 | 13/26 | 9.00E-04 | 1.36E-02 | EC2 | HMMTOP |
| *V1R14* |  |  |  |  |  |  |  |  |
| *V1R15* | 100 | C-A | 2/32 | 23/26 | 0.00E+00 | 0.00E+00 | IC1 | HMMTOP |
|  | 152 | G-A | 0/32 | 5/26 | 1.44E-02 | 4.92E-02 | TM2 |  |
|  | 581 | T-C | 0/32 | 7/26 | 2.19E-03 | 2.49E-02 | TM5 |  |
|  | 587 | A-T | 0/32 | 11/26 | 3.40E-05 | 1.80E-03 | TM5 |  |
|  | 632 | A-G | 0/32 | 5/26 | 1.44E-02 | 4.92E-02 | IC3 |  |
| *V1R16* | 301 | G-A | 13/32 | 0/26 | 2.11E-04 | 7.46E-03 | TM3 | HMMTOP |
|  | 470 | G-T | 0/32 | 6/26 | 5.69E-03 | 2.62E-02 | EC2 |  |
|  | 892 | C-T | 12/32 | 1/26 | 3.29E-03 | 2.49E-02 | C-terminus |  |
| *V1R17* | 653 | A-C | 0/32 | 5/26 | 1.44E-02 | 4.92E-02 | IC3 | HMMTOP |
| *V1R18* | 47 | T-C | 3/32 | 11/26 | 5.22E-03 | 2.62E-02 | N-terminus | HMMTOP |
|  | 146 | G-T | 3/32 | 11/26 | 5.22E-03 | 2.62E-02 | TM2 |  |
|  | 409 | T-C | 3/32 | 11/26 | 5.22E-03 | 2.62E-02 | TM4 |  |
|  | 523 | C-T | 3/32 | 14/26 | 3.45E-04 | 8.31E-03 | EC2 |  |
|  | 644 | G-A | 3/32 | 11/26 | 5.22E-03 | 2.62E-02 | IC3 |  |
|  | 652 | T--C | 5/32 | 16/26 | 3.92E-04 | 8.31E-03 | IC3 |  |
| *V1R19* | 53 | T-A | 0/32 | 6/26 | 5.69E-03 | 2.62E-02 | TM1 | HMMTOP |
| *V1R20* | 395 | T-C | 12/32 | 1/26 | 3.29E-03 | 2.49E-02 | TM4 | HMMTOP |
|  | 458 | C-G | 12/32 | 1/26 | 3.29E-03 | 2.49E-02 | EC2 |  |
|  | 557 | C-T | 12/32 | 1/26 | 3.29E-03 | 2.49E-02 | TM5 |  |
|  | 590 | T-C | 12/32 | 1/26 | 3.29E-03 | 2.49E-02 | TM5 |  |
|  | 686 | T-C | 12/32 | 1/26 | 3.29E-03 | 2.49E-02 | IC3 |  |
| *V1R21* | 661 | C-T | 7/32 | 0/26 | 1.34E-02 | 4.92E-02 | IC3 | HMMTOP |
|  | 704 | C-G | 7/32 | 0/26 | 1.34E-02 | 4.92E-02 | IC3 |  |
| *V1R22* |  |  |  |  |  |  |  |  |

Note-1. Nucleotide position of SNP in each *V1R* gene.

2. Frequency of SNPs in the basalt or the chalk population.

3. *P*-value was calculated by Fisher’s exact test.

4. *P*-values were adjusted by false discovery rate (FDR).

5. Each *V1R* gene has 15 domains, including N-terminus, TM1, IC1, TM2, EC1, TM3, IC2, TM4, EC2, TM5, IC3, TM6, EC3, TM7, C-terminus. TM, IC, EC indicate transmembrane, intracellular, and extracellular domains, respectively.

6. The topologies of all *V1R* proteins were predicted by the TMHMM or HMMTOP server.

**Figure S2.** The species tree of 24 rodents in this study. The letters (A-W) indicate the nodes of the tree. Branch lengths were estimated from the divergence times among species (Table S2).


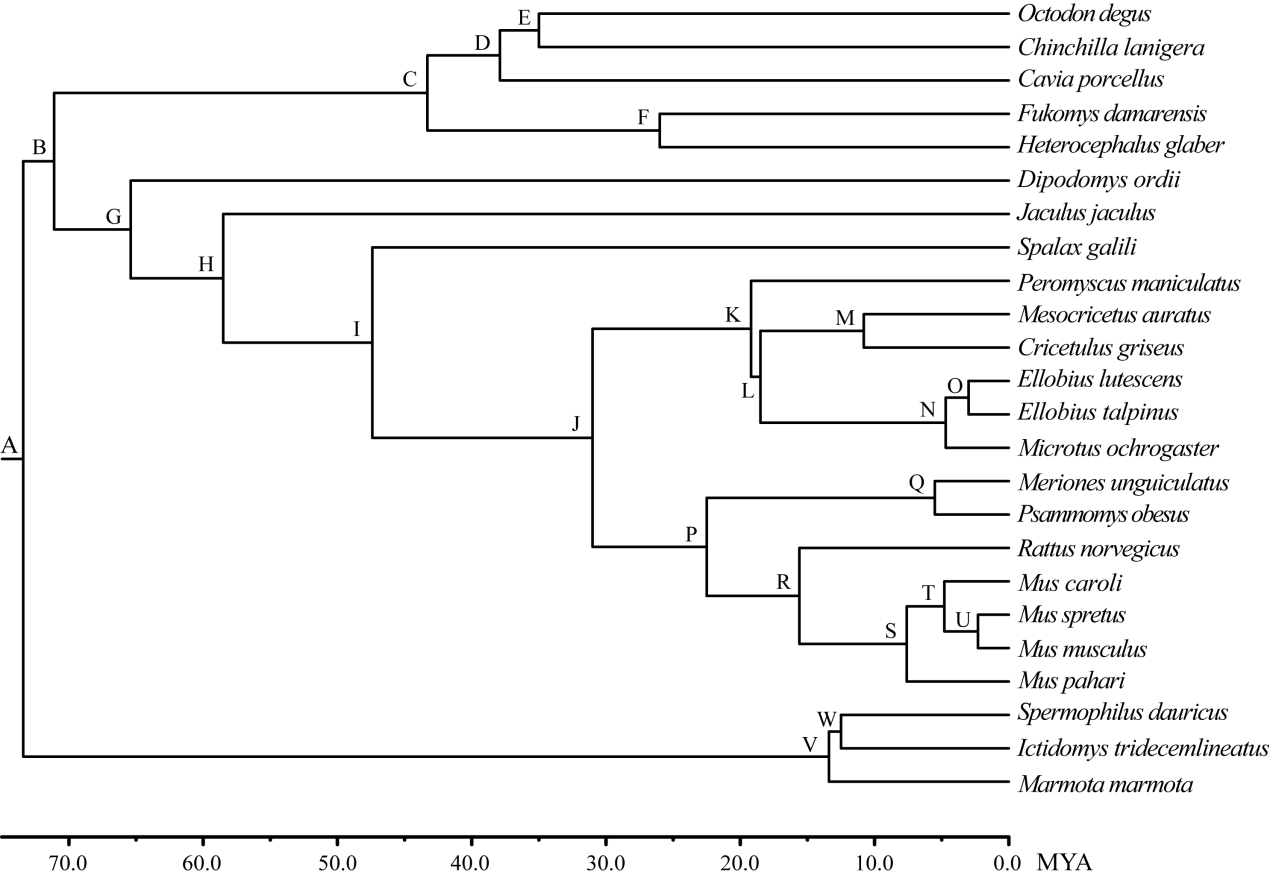


**Figure S3.** **PIC analysis based on the *V1R*s identified from 18 species that were sequenced on the Illumina platform, with the aim to avoid biases resulting from different sequencing platforms.** (A) Phylogenetically independent contrast (PIC) in intact *V1R* gene number is positively correlated with that in lifestyle code. **(B)** PIC in functional *V1R* gene number remains positively correlated with that in lifestyle code. The lifestyle in each animal was coded as 0 (subterranean rodent) or 1 (superterranean rodent). The Spearman’s rank correlation coefficient (ρ) with a two-tailed *P* value was used to evaluate the association.


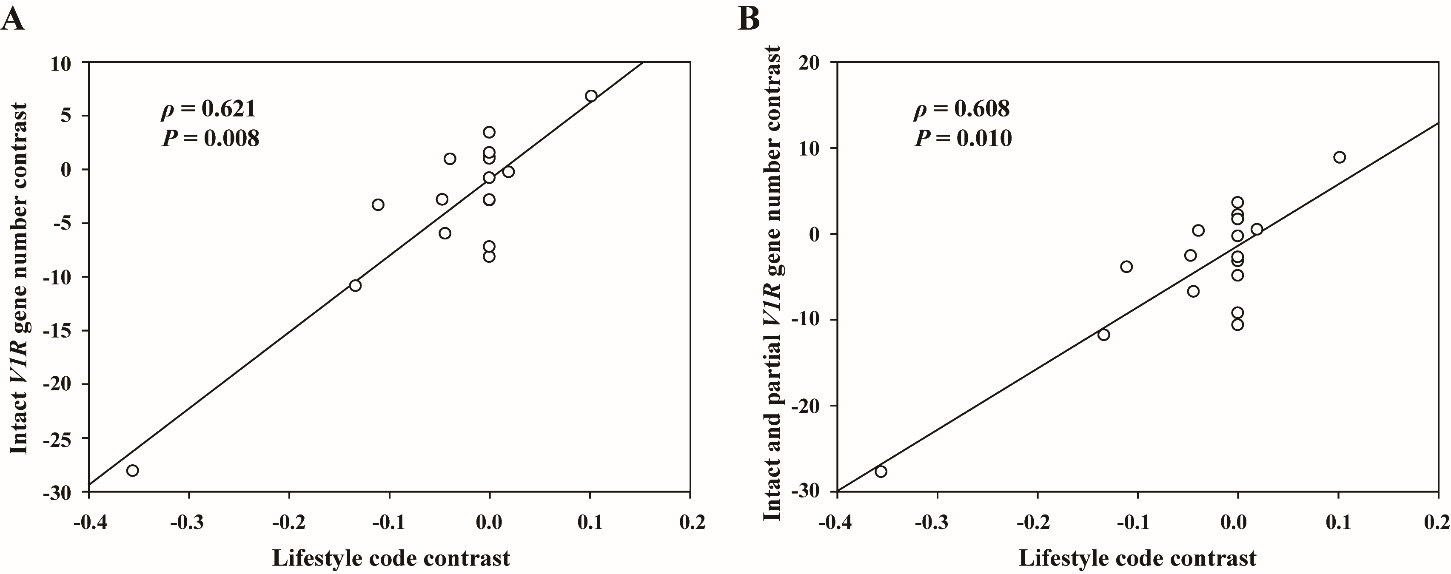


**Figure S4.** Number of significantly differentiated nonsynonymous SNPs between
populations in each of the 12 V1Rs.

**
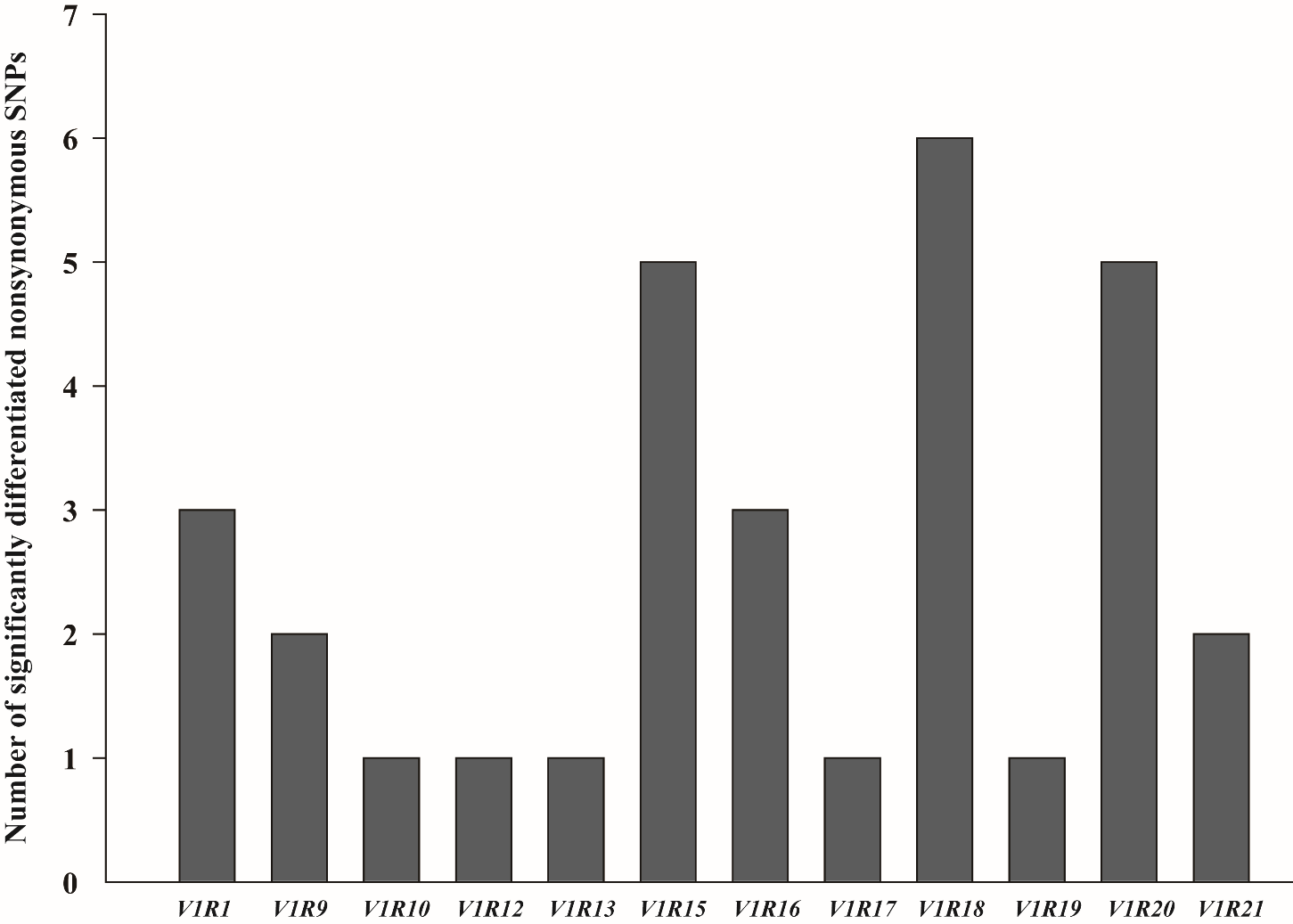
**
