# Supplementary material for: Convergent reduction of V1R genes in subterranean rodents
Source: BMC Evol Biol. 2019 Aug 30;19:176. doi: 10.1186/s12862-019-1502-4 (PMC6717356; doi:10.1186/s12862-019-1502-4)
Supplement: Supplementary file 3 — Nucleotide sequences of intact V1R genes identified from 28 mammalian genomes in the present study. These genes were named following the best hits of BLASTP searches. (DOCX 683 kb) [file 12862_2019_1502_MOESM3_ESM.docx]

**Additional file 3**

Nucleotide sequences of intact *V1R* genes identified from 28 mammalian genomes in the present study. These genes were named following the best hits of BLASTP searches.

>Cavia_porcellus_intact_ancV1R

ATGAAGCTCTCGGAAGACCTGCTTGAGATCATCTCTTGTGCCATCCTAATCTTTGTGAGCTTTGTAGGAAATGTATGTTTATTTTATTCTACAAGGAAGTGCATCGCTGGACGCTTACAGACATCATTTCTTCTGATTTTCAGCCTTGTGTTCGTCCACCTTATTAAGAACTTTGTGGTAAATGTCATGAAAATTGTTTATTCTTCTGGTATCACGTTGGATTCAGCCGGTTGCAAAGTTCTGCACTTCACAGCAGCCCTGACAACGTCACTGGCCATCTGGTTCACGTTGCACTTAGCACTGTTCTACCACCTGAAACTTTACCAAGTTGTCTACCCCTCGGATAATGCTGCAAGCCTAGACCAACAGAAACATTCTTTGAAGGGGATTTCTGCTCTTTGGGTGGCTGCCGTGGCTGTGTACGTACCAGTTTTAATTTATACTACAAAACCAGAATACCTGAAAGCAGGAAATGATACAGATGCCTTGTCTACGAAGAGAATTTACAAAGATTGCTTAATTATCTTCAGAAACAAGCAGGTAGAGTTTTACTATGGGAAAATCTTTTTAATGCTGATTGATATTCTTCCCTTAGCCATCTTAGTCTTTGTCTGTTTCTGGATGTCTTCCCTTCTTTCAGCAAAAAAGAAGATGACATATGGTGACATCTGGATTGGAGATGATGATTCAGAAATTGAAGTCCTGAGAGGGGCCAAGTGCAGCATAGTATTAACGCTGCTGATCACTCTGCTCTGGGTGTCCCACTCTGTCTTGGTCTATTTCTGGAGCAGCGTGGCAGCCTGTGTCTTCCTCCCCGCTGTCCTCACAGCACTGTCCTCCGGCTTCTCTGCTCTCAGCCCTTTCCTGCTGATGCTGGTTAATTACAGAGTGAAGTTGGTATCACTTTGTGGTGCCAAAGAGGAAAACTCCATACCACAGGCGACAAATACTATTTTTTCTCCATATGCTTAA

>Cavia_porcellus_intact_V1R4_1

ATGAGCCGCGTTACTCAAAGGAATGATTGTCTTAGCAATTTAAATGAAATTTTAAGAGTAAATGACAAAATAACTTCCAAAGATTTGGCAGTAGGAATAATCATCTTGTCACAGACTATCATTGGTATCATGGGCAATTTCTTTGTTCTTTACCATTATATCTTCATTGTCCACACTAGAAGGAGGTTAAGGTCCACAGACGTGATTCTGAAGCACCTTTTCATAGCAAACTGCTTAGTTCTTCTTTCTGGAGGAATTCTTCAAACAATATCAGTGTTTGGGTTGAAACTTATCTTCAGTAATGCGGTGTGTAAATTTCTCTTGTACGTTGAGAGAGTGTGCAGAGGTGTGTCAATTAGTGCCATCTGCCTCTTGAGTGTCTTTCAAAACATCATCATTAGCCCCATAAATTCCTGTTCAAAAAGTGTTAAAGGTAAAGTTCAAAAATATATTGGCTTCTCTACTCGATTCTGCTGGATCCTAAACATGGTGGTTGGCTTAATTATTCCTTTATATGCATGGTATTCATCTGTAAAAGGTTATGACAGAATCATCAAAAAGAAAATAAATCTAGGATACTGTTGTCTTGTGGATTATGGGGTAGTTATTCGTTCTATCTATACAGCATTAGTAATTTTCCCTGAAGTTTGCTTTGCTCTGCTTACTGTCTGGGCCAGTGGCTCTATGATTTTGGTCCTGTACAGACACAAGCACCGGGTCCAACACATTCACACCACTAATGTTTCCTTAAGATCCCCTGAGTCCAGAGCCACCCAGAGCATTCTCCTACTGGTGATCACCTTCATATCTTGTTACACTGTCTCCACCATTATTCGAATTCCTATTGCTTTAGCTCAGAATCTCAGTTCATGGATAGTGAGTGCTTCTGATCTAATATCAGGGTGTTTTCCAATGATTAGCCCCTTTCTGATTGTAAGCCAACGCTACTCTATATTTAGGCACTGTTTTGCCTGCATAAGAAACCTAAAAGCTTTGTAA

>Cavia_porcellus_intact_V1R4_2

ATGTCATCTAGCAACTTGGCAATAGGAATGACTTTTCTGACTCAGACAGTCATTGGCACCTTAGCTAACACTGTCCTTCTGTTCCATTATCTCTCTCTGTACTTCACTGGATACAAGTTCAGGCCCACTGATCTAATAGCTGAGCACTTGACTGTAGCAAACACACTGATCATGCTCTCTAAGGGAGTCTCACAGACATTACAAGCTTTTGGAATAAAGTATTTCTCTCCGGGTAATAGATGCATATTTCTTTTGTATATCTACAAATTGGCAAGGGGTGTATCATTTAGTACTACCTGCCTGCTGAGTATCTTCCAGACCATTAAGAGGAGCCCCATGAACTCCTTTCGGAAGGAGCTTAAAGCTAAACTTCCCAAGCATATTGGATTCTCGATGTTCCTCAGTTGGATTTTCTGTATGTTGATAAATTGTAATTTTCCATTTTATAATATCATGAAATATAGCAATAAAAATATCAAAAAGAGAAAATGTTTTGGGTACTGCTATGCTGGATTTCAAGACAAAATCATAGATGCATTGTATGCAGCATCTGTGTTATTCCCTGAAGTTTCATGTTCTGGTCTCATGATGTGGTCCAGTGGCTCCATGATTTTCATTTTGCACAGGCACAAGCAGCAGGTCCAACACATCCTTAGGACAAATGATTCCCACAAAACTACCCCTGAGATCAGGGCCACCTACAGCATCTGCATTCTTATAGGCACCTTTGTGTCCTTTTACACCCTCTCCTCATTCTTCTATGCTTGTATTGTGAATTTCAGTATTCCTAGTTGGGTGGCTCATAAACACCTCTGCCCTGATTTCCACCTGCTTCCCCACTATCAGCCCTTTTGTTCTAATGAGCTGTAA

>Cavia_porcellus_intact_V1R601

ATGAATGTGAACAAGGAATTGTCCAATAATATTAACATCCGAATCACATTTTATTTTCTGTTTATCATTGGGGTCTCAGGCAATGCCATCCTTCTTCTCTTCCACATCGTTGTGTTTGTTGTAGGGCACAGGCCCAGACTCACTGACCTGCCCATTGGCCTCTTGGCCCTAAACCACCTAATGACAATGCTAACCACAGGATTCATAACTGGAGACATTTTTACCTCGCAAGGGGGGTTTTGGAATGATATTACATGTAAATCAGTTATCTACTTGAACAGGTTGATGAGGGGGCTCTCCATGTGTATCACCTGCCATCTGAGTGTCCTTCAGGCTATCACGCTCAGCCCCAGAAGCTCTTGTTTGGCAAAGTTAAAACACCAATCCTCATGTCACAGCCTGTGTTCCCTTCTTTTCTTTTGGCTGTTATATATATCCATTAGCAGTCACCTTTTTATCTCCATCGCTGCTAGTACCAATTTAACTTCAGAAAATATTTTGTATGTCAGTAAGTCCTGTTCTCTTTTACCCATGAACTATTTACTCCGGCACATAGTTTTCATATTATTGACCATCAGAGATATGTTCTTTATAGGAATTGTGTCCCTCTCAGGTGGATACATAGTGATTCTCTTGTGCAGGCATAAGAAAAAGTCCCAGCATCTTCACAGGAACAAAGTTTCTCCAAAAGCATCCCCAGAACAAAGGGCCACTCGGACCATTCTGCTGCTTATGTGTCTCTTTGTAGTTATCACCAGTTTGGACATTATATCTTCCTCAAGAATTGTGTTGAATAATAATCCAGTAATATATTGTATTCAAATTTTTGTGGCCCATGGCTATGCCACAGTCAGTCCTTTAGTATTCATTACTACTGAAAAACGTATCATTAATATTTTGAGACTCATGTGTGGAAGAAAATAA

>Cavia_porcellus_intact_V1R602

ATGAATGTGAACAAGGAATTGTCCAATAATATTAACATCAGAATCATATTTTATTTTCAGTTTATGATTGGGGTCTCAGGCAATGTCATCCTTCTTCTCTTCCACATCGTTGTGTTTGTTGTAGGGCACAGGCCCAGACTCACTGACCTGCCTATTGGCCTCTTGGCCCTAAACCACCTAATGACGATGCTAACCACAGGATTCATAACTGGAGACATTTTTACCTCTCAAGGGGGGTTTTGGAATGATATTACATGTAAATCAGTTATCTACTTGAACAGGTCGATGAGGGGGCTCTCCATGTGTATCACCTGCCATCTGAGTGTCCTTCAGGCTATCACGCTCAGCCCCAGAAGCTCTTGTTTGGCAAAGTTAAAACACCAATCCTCATGTCACAGCCTGTGTTCCCTTCTTTTATTTTGGCTGTTATATATATCCATTAGCAGTCACCTTTTTATCTCCATCGCTGCTAGTACCAATTTAACTTCAGAAAATATTTTGTATGTCAGTAAGTCCTGTTCTCTTTTACCCATGAACTATTTACTCCGGCACATAGTTTTCATATTATTGACCATCAGAGATATGTTCTTTATAGGAATTGTGTCCCTCTCAGGTGGATACATAGTGATTCTCTTGTGCAGGCATAAGAAAAAGTCCCAGCATCTTCACAGGAACAAAGTTTCTCCAAAAGCATCCCCAGAACAAAGGGCCACTCGGACCATTCTGCTGCTTATGTGTCTCTTTGTAGTTATCACCAGTTTGGACATTATATCTTCCTCAAGAATTGTGTTGAATAATAATCCAGTAATATATTGTATTCAAATTTTTGTGGTCCATGGCTATGCCACAGTCAGTCCTTTAGTATTCATTACTACTGAAAAACATATCATTAATATTTTGAGACTCATGTGTGGAAGAAAATAA

>Cavia_porcellus_intact_V1R603

ATGATTTCAACATCTAGAATCCTGGGAATTGCTTTTCTCTTTCAAATTGGGCTTAGTATCATGGGGAATGCCATTCTCTTTGTAGGCTACACTTACATCTTCATTGCGAATCCCAAGCAAAGGCCCATGTGTCTAATAGTAACCCACTTGTCGGTGGTCCACCTGTTATTAGTCTGTACCGCAGGAGTCCCTGGAGTGGCAGAAATTCTGGGTTACAGAGTTCTCATCAATGATATTATGTGTAAGATAAATACTTATCTGCACAGAGTGTCGCGTGCCCTCTCCATCTGCTTGACAAGCCTCTTAAGCATTCTTCAGACCATCACCATTAGCCCTGCAAGTTCCTACTGGGCAGTATTCAAAACCAAAAACTTATTGTACATCATTTATTCCTTCTTCTTTGTTTGGATCCTTAATCTACTTATATGTTCAAACATGATTATCTCAATGACAGCCCCCAAGAGTGGTAATATATCCAAAGTCATTGCCAAATCTTGTTTTATTTTGTCAATGAATGATTTCATTAAATGGTTTTTTCTCATGTTCTTGGTCCTGCGAGATATTACCTTCCTGGGTTCTATGGGCTGGACCAGTGGGTACATGACTCTCCACCTCTACAGACACCACAAGCGAACACAGCACCTTCGCAGGTCAACCACACCTTTTAAAATGAACCATGAGATGAGAGCAACTAAAAAGATCATTATTTTAACTCTGTGCTTCATCTCTTTGTATGGGATTAATATGATTCTTTCCATTAATATAGGGTACTTTTCAAAAACTGAACCTGTGATTTTGCAGATTGAGCAGTTAGTTGCGGCGAGCTTTGCTTTACTAAGCCCTGTAGTGTTTCTCAGCAACAACAGCCAACTTCACATATTTTGGGCATCTGTCTTTGAGAAAATAAGGAATTTACAAAATAGAGGAACAAATGCCATGCAAATGCCATGCAAATGCTGA

>Cavia_porcellus_intact_V1R604

ATGAAGAAGAATGAAATCAGCCAACTTTCCAGGTTTATCTACATACGAAACATATTTCTCTTGGAAGTCATCATTGGGATCATAGCCAACACTGCCCTACTTCTCTTTCATGTTCTCATAATACTTGTGGAGCACAAACTCAAGCTCATTGATCTGACTATTGGTCACTTGGCCCTGATCCATGTAGTAATGCTTCTAACTGTGGCTTTCATAGCCATGGACATTTTTGAGTTTCAGTATTTGGGGGATGACATCACATGTAAATGTGTTATCTATTTGTATAGATTGATGAGGGGTCTTTCCATCTGTACCACCTGCATGCTAAGTGTCCTCCAGGCCATCACCCTCAGCCCCAGAAGCTCCTGTCTGGCAAAGTTCAAACAGAAGTCCCTGCATCAGAATCTGTGTTGTTTTCTATTTTTATGGATCTTCAATATACTCATCAGTGTTCATTTCTTAATCTCCATTATTGCCACTCCCAATATGACCTCACACAGTTTTATGTTTGTCACTCAGTCCTGCTCCCTTTTGCCCAGTAGTTATTTACTCAAATACATATCTATCTCACTTCTGAGTTTACAGCACGTGACCTTTATAGGCTTCATGGCACTTTCAAGTGGGTACATGGTGCTTCTCTTGCATAGGCATAAAAGGCAGTCCCAGCATCTTCACAGCACCAGCCTGTCTCCAAAAGCATCTGCAGAACAAAGGGCCACCCACACTATTTTGTTGCTTATGAGTTTCTTTATCATTATGTACATTTTGGACAGTGTTTTGTCTACCACCTCTGCAATGCTATGGGATCGTGACCCAATTCGTCATTTTGTTCATATGTTTGTTGGCAATGGATATGCCATAGTTAGTCCTTTGGTACTAAGTAGTACTGCAAAAAGAGTGATCAACTTCATAACACCCATGTAG

>Cavia_porcellus_intact_V1R605

ATGAATATAAACAGCACATTTTCTAGTGTCATTGGCATCCAACACTTGGTTTTCTTTGAAATCACCATTGGGATCATTGCCAACATGTTCCTGCTTATCTTACATGTCCTCACATTCCTTTTGGAGCACAAGTCTAAGCCTATAGATCTGACTATTGGTCACTTGGCTCTTATTCACATAGTGATGCTCATAACTGTGAGTTTCATAGCTATAGACACTTTCGGATATTGGACTTTCAAGGATGTAATCTCATGTACATCTATCATGTATTTGAATAGTTTGATGCGGGACCTCTCCATCTGCAGCACCTGTCTGCTGAGTGTCCTCCAGGCCATCACTGTCAGCCCCAGAAGCTCCTGTGTGGCAAAACTCAAGCGGAAATCTCTCCATCACAACCTGTATGGCTTTCTCTTTTTTTGGGTCTTCAATATGTTCAATAGTGGTCGAATCTTAATTTCCACTGTTGCCACTCCCACTGTGACCTCACATGGTCTTATATTTCTTAGTAAATCCTGCTCCCTTTTGCCCATTAATCCTTTCCTCAAGTATATATTTTTTTCACTAATGACTTTCAACTACGTGTCCTGTATAGGGCTCATGGTTCTCTCAAGTGGGTACATGGTGATTCTCCTATGCAAACATAAAAAACAATCCCAGTATCTACATAGCACCAGCCTGTCTCCAAAAGCATCCCCAGAAAAAAGGGCTATATGGACTATTTTGTTGCTCATGAGTTTTTTTATCATTTTTTACTCTCTGGACTGTGTTTTCCATTCCATGTACTCAATCATGAGGAGCCATGACCAAATTCGCCACTGTGTCCTGATGCTAGTTGGCAATGGCTATGCCACAATCTGTCCTTTGGTGCTCATCAGTAGTGAAAGACAGTTTATCAAGAGCTTCATATTCATATTCAGGATAGACAGTAAATGA

>Cavia_porcellus_intact_V1R606

ATGAAGATGAATAAAAATGGCAGATTTTCCAATTTTATTGATGTTCAGCACATATTTTTCTTTGAAATAATCATTGGAGTCATGGCCAACACTTTTTTGCTTCTCTGTCATGTCCTCATATTTCTTCTGGAGCACAAGGCAAAGCCCACTGATCTGACCATTGGTCACTTGAGCCTTATCCATATAGCAATGCTTTTAATTGCAGGTTTTATAGCGATAGATCTATTCGGGTTTCAGCACTTGGGGGATGACACCACATGTAAATGTGTTATCTATTTGCACAGGTTAATGAGGGGCCTCTCTATTTGTACCACTTGCCTGCTGAGTGTCCTCCAGGCCATCACCCTCAGCCCCAGAACCTGCTGTTTGACTAAATTCAAACACAAATCCCTGCAACAGAACCTGTGTTGTTTTCTCTTTTTATGGGTCTTCAATATGCTCATCAATGGTCGTTTCTTAATCTCCACTGTTGCCACTCCCAATGTGACCTCACACAATCTTATTGTTGTCACTCAGTCTTGCTCCATTTTGCCCATTACTTCCTTCCTCAAGTACATATCTTTGTCACTGATGGTTTTCCAGCACATGACATTTATAGGGCTCATGGCACTCTCAAGTGGGTACATGGTGATTCTCTTGTGCAAACATAAAAGACAATTGCAGCATCTTCACTGCACCAAACTATCTCCAAAATCATCCCCAGAAAAAAGGGCCACCCAGACTATTTTGTTGCTCATGAGTTTCTTCACTGTCATGTATGTTTTGGACAGTATTATTGAATATACCTCGGCAATGTTGTGGAACCATGACCCAATCAGTCGTTGTATCCAGATGCTGATTGGCAATGGCTATGCCACAGTCAGTCCCTTGGTGCTCATCAGTACTGAAAGACGATTGATTAAGTGTTTTTTATAA

>Cavia_porcellus_intact_V1R607

ATGAATATAAACAGCACATTTTCTAGTGTCATCAGCATCCAACACTTGGTTTTCTTTGAAATCACCATTGGGATCATTGCCAACGTGTTCCTGCTTATCTTACATGTCCTCACATTCCTTTTGGAGCACAAGTCTAAGCCTATAGATCTGACTATTGGTCACTTGGCTCTTATTCACATAGTGATGCTCACAACTGTGAGTTTCATAGCTATAGACACTTTTGGATATTGGACTTTCAAGGATGTAATCTCATGTACATCTGTCATATATTTGAATAGTTTGATGCGGGACCTCTCCATCTGCAGCACCTGTCTGCTGAGTGTCCTCCAGGCCATCACTGTCAGCCCCAGAAGCTCCTGTGTGGCAAAACTCAAGCGGAAATCTCTGCATCACAACCTGTATGGCTTTCTCTTTTTTTGGGTCTTCAATATGTTCAATAGTGGTCGAATCTTAATTTCCACTGTTGCCACTCCCACTGTGACCTCACATGGTGTTATATTTTTTAGTAAATCCTGCTCCCTTTTGCCCATTAATCCTTTCCTCAAGTATATGTTTTTTTCACTAATGACTTTCAACTACGTGTCCTGTATAGGGCTCATGGTTCTCTCAAGTGGGTACATGGTGATTCTCTTGTGCAAACATAAAAAACAATCCCAGTATCTACATAGCACCAGCCTGTCTCCAAAAGCATCCCCAGAAAAAAGGGCTATATGGACTATTTTGTTGCTCATGAGTTTTTTTATTGTTTTTTACTCTCTGGACTGTGTTTTCCATTCCATGTACTCAATCATGAGGAGCCATGACCAAATTCGCCACTGTGTCCTGATGCTAGTTGGCAATGGCTATGCCACAATCTGTCCTTTGGTGCTCATCAGTAGTGAAAGACAGTTTATCAAGTGCTTCATATTCATATCCAGGATAGACAGTAAATGA

>Cavia_porcellus_intact_V1R608

ATGAAGATGAATGAAATCAGCCAACTTTCTAGGTTTATCTACATACGAAACATATTTCTCTTGGAAGTCATCATTGGAATCATAGCCAACACTGCCCTACTTCTCTTTCATGTTCTCACAATCCTTGTGGAGCACAAGCTCAAGCCCATTGACCTGACTATTTGTCACTTGGCCATGATTCATGTAGTAATGCTTCTAACTGTGGCTTTCATAGCCATGGACATTTTTGAGTTTCAGTATTTAGGGGATGACATCACATGTAAATGTGTTATCTATTTGTATGGATTGATGAGGGGTCTTTCTATCTGTACCACCTGCATGCTAAGTGTCCTCCAGGCCATCACCCTCAGCCCCAGAAGCTCCTGTCTGGCAAAGTTCAAACAGAAATCCCTGCATCAGAATCTGTGTTGCTTTCTATTTTTATGGATCTTCAATATACTCATCAGTGTTCGTTTTTTAATCTCCACTATTGCCACTCCCAATATGACCTCACGCAGTTTTATGTTTGTCACTCAATCCTGCTCCCTTTTGCCCAGTAGTTACTTACTCAAATACATATCTATCTCACTTCTGAGTTTCCAGCACGTGACCTTTATAGGCTTCATGGCACTTTCAAGTGGGTACATGGTGCTTCTCTTGTATAGGCATAAAAGGCAGTCCCAGCATCTTCACAGCACCAGCCTGTCTCCAAAAGCATCTGCAGAACGAAGAGCCACCCACACTATTTTGTTACTCATGAGTTTCTTTATCGTCATGTACATTTTGGACAGTGTTTTGGCCTCCACCTCTGCAATGCTATCGGATCGTGACCCAATTCGTCATTTTGTTCAGATGTTTGTTGGCAATGGATATGCCACAGTTAGTCCTTTGGTACTCATTAGTACTGCAAAAAGAGTGATCAACTTCATAAAACCCATGTAG

>Cavia_porcellus_intact_V1R609

ATGAAGATGAATAAAAATGGCAGATTTTCCAATTTTATTGATGTTCAGCACGTATTTTTCTTTGAAATAATCATTGGAGTCACGGCCAACACTTTTTTGCTTCTCTATCATGTCCTCATATTTCTTCTGGAGCACAAGCCAAAGCCCACTGATCTGACCATTGGTCACTTGAGCCTTATCCATATAGTAATGCTTTTAATTGCAGGTTTTATAGCGATAGATATATTCGGGTTTCAGCACTTGGGGGATGACACCACATGTAAATGTGTTATCTATTTGTACAGGTTAATGAGGGGACTCTCTCTCTGTACCACTTGCCTGCTGAGTATCCTCCAGGCAATCACCCTCAGCCCCAGAAGTTCCTGTTTGACTAAATTCAAACAAAAATCCCTGCAACATACCCTGTGTTGTTTTCTCTTTTTATGGGTCTTCAATATGCTCATCAATGTTCGTTTTTTAATCTCCACTGTTGCCATTCCCAATGTGACCTCACACGATCTTATGGTTGTCACTCAGTCTTGCTCCATTTTGTCCATTACTTCCTTCCTGAAGTACATATCTTCGTCATTGATGGTTTTCCAGTACATGATGTTTATAGGGCTCATGGCACTCTCAAGTGGGTACATGGTGATTCTCTTATGCCAGCATAAAAGACAATTGCAGCATCTTCACCGCACCAAACTATCTCCAAAATCATCCCCAGAAAAAAGGGCCACCCAGACTATTTTGTTGCTCATGAGTTTCTTCACTGTCATGTATGTTTTGGACAGTATTATTGAATATGCCTCAGCTATGTTGTGGAACCATGACCCAATCAGTCGTTGTGTCCAGATGCTGATTGGGAATGGCTATGCCACAGTCAGTCCCTTGGTGCTCATCAGTACTGAAAGACGATTAAGTGCTTTATATAAATGTAGGGGCAAACATAGGAATTTTTTTCTTTTGTTTCACAGAGTAATCCCTCTGACAATGGGCAATTGA

>Cavia_porcellus_intact_V1R610

ATGAATAAAAATGGCAAATTTTCCATGTTTATTGATGTACAACACGTGTTTTTCTTTCAAGTCACCACTGGGGTCATGGCCAACACTTTTTTGCTTCTCTTCCAAGTCTTCATATTTTTTCTGGAGCACAAACCAAAGCCCACTGATGTGACCATTGGTCATTTGGCCCTTATCCACATAGTGATGCTCCTATCTGCGGGATTCATAACTATCGACATATTTGGGTTTCAGGACTTGGGAAACAGGATCACATGTAAATGTGTTATCTATTTATACAGATTGATGAGGGGCCTCTCTATCTGTACCACGTGCCTACTGAGTGTCCTCCAGGCCATCACCCTCAGCCCCAGAGCCTGTCCTTTGGCAAAGTTCAAACAGAAATCCCTGCATCAGAATCTGTATTGCTTTCTCTTTTTATGGATCTTCAATATGCTCATCAATGGTCGTTTCTTGATCTCCACTGTTACCACTCCCAATGTGACCTCACACAGTCTCATGTTTGTCACTAGGTCCTGCTCTCTTTTGCCCATTAGTTCCTTTCTCAAGTACATGTCTTTGTCACTGATGATTTTCCAGCATATATTGTTTATAGGGCTCATGGCACTTTCCAGTGGGTACATGGTGATTCTCTTGTACAAGCATAAAAGACAATCACAGCATCTTCACTGCACCAAACTGTCTCCAAAAGTATCCCCAGAACAAAGGGCCACGTGGACTATTTTGTTGCTCATGAGTTTCTTCATTGTCATGTACATTTTGGACAGCGTTATTGAGTACACCTCAGCAATGTTGTGGAACCATGACCCAATCCGACATTGTGTCCACATGCTGTTTGGCAGTGGGTATGCTACAGTCAGTCCCTTGGTGCTCATCGGTACTGAAAGACGATTGATCAAGTGCTTTATATTCACTGGAGGGAAACAAAGTAATTTTTTTTTGTTTCATGATAGGTAA

>Cavia_porcellus_intact_V1R611

ATGAATGAAAACAATACACTTTACAGGTTTATTGGTCTGCATATCATGTTTTTCTTAGAAGTCAGCATTGGAATTATAGCCAATACTGTCCTGCTTCTCTTTCGTGTCCTTAAATTTTTTCTTGAGCACAGGCTCAAATCCACCAACCTGATTACTGGGCACCTGGCACTCATACATATAATGATGCTACTAGTCTTTAGTTTCATAGCCAGAGACATGTTTGGGTTTAAGAACTTGGGAGGTGTCATCACATGTAAATCCATTATCTATTTGCACAGACTGATGAGGGGCCTTTCCATCTCTACCACATGCTTGCTGAGTGTCCTGCAGGCCATCACCCTAAGCCCCAGAAGCTCCTGTCTGGCAAAGTTCAAATACAGATCCTCACATCACAGCCTTTTTTGTTTTCTTTTTTTATGGATCTTTAATATGTTCATCAGCAGTCGTTTCTTATTTGTCACTGGTATCACTGCTAATGTGACTTCACATAACCTCATGTTTCTCACTGAATCCTGCTCTCTTTGGCCCATCAGTTACTTATTCAAGGACCTGTATTTCTCACTGACAACCATCCGTGATATTTTCTGTATAGGGCTCATGGTACTCTCGAGTGGTTACATGGTAATTCTCTTGTTCATGCATAAAAAACAGTCCCGGAATCTTCACAGCACCAGTCTGTCTTCAAAAGCATCTCCAGAACAAAGGGCCACCTGGACTATTTTGTTGCTTATGAGTTTCTTTACAGTCATGTACTTTTTTGACTATGCTATTGCCTCTATTTCTGGAATGTTATGGAAAAGTGACCCAATTCGTCACTGTATCCAGATGCTGGTGGGCAATGGCTATGCCACAGTAAGTCCTTTGGTGCTAGTCAGCACTGAAAAACAAATAATCACTTTCTTAATATCTATATGGAAGAAAGACAATAGGTGTTTGATTATTAAGTGA

>Cavia_porcellus_intact_V1R612_1

ATGTATAAATACAACATACTTTCAAGATTTATTGGTGTACAATACATAGTCTTTTTTGAAGTCAGCATTGGAGTCATAGCCAACATGGTACTGCTACTCTTTCATGTCCTAAAATTCCTTCTGGAGCACAGGCCCAAGCCTACTGATCTGATTATTGGTCACCTGGCTCTTATACATGCTGGGATGCTTCTAACTTTGGGCTTCATAGCTATAGATATATTTGGGTTTCAGAAGTTGGAGGATGACATCGCATGTAAATGTGTTATCTATTTGCACAGATTGATGAGGGGCCTCTCTGTCTGTACCACCTGCCTGCTGAGTGTCTGCCAGGCCATCACCCTCAGCCCCAGAAGCTCCTGTTTAGCAAAGTTCAAACAGAAATCCCTACAGCAGAACCTGTATTGCTTTGTCTTCATATGGGCCTTCAATATGCTCATCAATGGTCATTTCTTAATCTCCACTGTTACCACTTCCAATGTGACCTTAGGCAGTCTCATGTTTGTCACTCGATCCTGCTCCTTTTTGCCTATTAGTTCCTCTCTGAAATACACATCTATCTCACTGCTGAGTTTCCAGCACATGACCTTTATAGGGCTCATGGCGCTCTCAAGTGGGTACATGGTGATTCTCTTGTGCAGGCATAAAAGACAGTCCCAGCATCTTCACAGCACCAGTCTGTCTCCAAAAGCATCTGCAGAACAAAGGGCCACCAGGATTATTTTGGTGCTCATGAATTTATTTATTGTCATGTACATTTTGGACAACATTATTTCCTCTACTTCTTACATGTTGTGGAACTTTGACCAAATTCGTCTTTGCATCCAGATTCTGGTTGGCAATGGCTATGCCACAATCAGTCCTTTAGTGCTCATGAGAACTGAAAGAGTGATGACATTTTTTAGATCCAAATAG

>Cavia_porcellus_intact_V1R612_2

ATGAAGATGAATAAAGACAACATACTTTCAAGTTATATTGATGTACAATACATATTCTTTTTTGAAGTCAGCATTGGAATCACAGCCAACGTTGTACTGCTTCTCTTTCATGTCTTAAAATTCCTCCTGGAACACAGACCCAGTCCTACTGATCTGATCATTGGTCACTTGGCTCTTATACATGCTGTGATGCTTCTAACTGCGGGCTTCATAGCTATAGACATATTTGGGTTTCAGAAGTTGGGGGATGACATCGCATGTAAATGTGTTATCTATCTGCACAGACTGATGAGAGGCCTCTCTGTCTGTACCACCTGCCTGATGAGTGTCTTCCAGGCCATCACCCTTAGCCCCAGAAGCTCCTGTTTAGCAAAGTTCAAACAGAAATCCCTACAGCAGAACCTGTATTGCTTTCTTGTCTTATGGGCCTTCAATATGCTCATCAGTAGTCGTTTCTTAATCTCCACTGTCATCACTCCCAATGTGACTTCAAATAGTCTCATGTTTGTCACTCGATCCTGCTCCATTTTGCCAATTAGTTCCTCTCTCAAATATATCTCTATCTCACTGCTGAGTTTCCAACACCTGACCTTTATAGGGCTCATGGCACTCTCAAGTGGGTACATGGTGATTCTCTTGTGCAGGCATAAAAGACAGTCCCAGCATCTTCACAGCACCAGTCTGTCTCCAAAAGCATCTGCAGAACAAAGAGCCACCCAGACTATTTTGGTGCTCATGAATTTATTTATTGTCATGTACATTTTGGACAGCATTATTTCCTCTACTTCTGACATGTTGTGGAACTTTGACCAAATTCGTTTTGCATCCAGCTTCTGGTTGGCAATGGCTATGCAACGATCAGTCCTTTGGTGCTCATGA

>Cavia_porcellus_intact_V1R613

ATGAATAAAAATGGCAAATTTTCCATGTTTATTGATATACAACACGTGTTTTTCTTTCAAGTCACCACTGGGGTCATGGCCAACACTTTTTTGCTTCTCTTTCATGTCTTCATATTTCTTCTGGAGCACAAACCGAAGCCCACTGATCTGACCATTGGTCATTTGGCCCTTATCCACATAGTGATGCTCCTATCTGCAGGTTTCATAACTATAGACATATTTGGGTTTCAGGACTTGGGAGATAGGATCACATGTAAATGTGTTATCTATTTGCACAGATTGATGAGGGGCCTCTCCATCTGTACCACGTGCCTACTGAGTGTCCTCCAGGCCATCACCCTCAGCCCCAGAGCCTGTCGTTTGGCAAAGCTCAAACAGAAATCCCTGCATCGGAACCTGTATTGCTTCCTCTTTTTATGGATCTTCAATATGCTCATCAATGGTCGTTTCTTGATCTCCCCTGTTACCACTCCCAATGTGACCTCACACAGTCTCATGTTTGTCACCAGGTCCTGCTCTCTTTTGCCCATCAGTTCCTTCCTCAAGTACATGTCTTTGTCACTGATGATTTTCCAGCATATATTGTTTATAGGGCTCATGGCACTTTCCAGTGGGTACATGGTGATTCTCTTGTACAAGCATAAAAGACAATCACAGCATCTTCACTGCACCAAACTGTCTCCAAAAGTATCCCCAGAACAAAGGGCCACGTGGACTATTTTGTTGCTCATGAGTTTCTTCATTGTCATGTACATTTTGGACAGTGTTATTGAGTATACCTCAGCAATGTTGTGGAACCATGACCCAACCCGTCATTGCGTCCACATGTTGTTTGGCAGTGGATATGCTACAGTCAGTCCCTTCGTGCTCATTGGTACTGAAAGACGATTGATCAAGTGCTTTATATTCACTGGAGGGAAACAAAGTAAATTTTTTTTGTTTCATGATAGGTAA

>Cavia_porcellus_intact_V1R614

ATGAATATGAGCAAAAACAGTAGATTTACTGGTGTCATTGACGTACATCATATGGTTTTTTTTGAAGCCAGCATTGGAATCATTGCCAACACAGTTCTCTTTCTCTTTCATGTCCTTCCATTCCTTCTGCAGCACAGGCACAGGCCTACTGATCTGACCATTGGTCACTTGGCCCTTATCCACATAGTCTTGCTCATAACAGAGGGTTTCCTAGCTATAGACATTTTGGGATATTGGCACTTGGGAGATGACATCACATGTACGTCTGTTGTCTATTTGAATAGGTTGATGCAGAACATCTCCATCTGTACCACCTGCCTGCTGAGTGTCCTCCAGGCCATCACCCTCAGCCCCAGAACCTCCTGTTTGGCAAAGTTCAAACAGAAATCCTTGCATCAGGTCCTGTGTTGCTTTCTGTTTTTGTGGGTCTTGAATATGCTCATCAGTAGTCGTTTCTTAATTTCCACTGTTGCCTCTTCCAATGTGACCTCACATGGTCTTATGTTTCTTACTAAATCCTGCTCCCTTTGGTCTATTAGTTCTTTTCTCAAGTATATGTTTTTCTCACTGATGGTTTTCCACTACATGTCTTGGATAGGGCTCATGGTGCTCTCAAGTGGGTATATGGTGATTCTCTTACGCAGACATAAAAGACAATCCCAGCATCTTCACAGCACCAGTCTGTCTTCAAAAGCATCCCCAGAAGAAAGGGCCACCCGCACGATTTTGTTACTCATGAGTTTCTTTATTATTATGTACTGCTTGGACTATGTTATATACTCCATGTCCTCAATCACATGGAAATATAACCCATTTCATCATTGTGTCCTGATGCTGGTTAGCAATGCCTATGCCACAGTCAGTCCTTTGGTGCTCATCAGTAGTAAAAGACGAATGATCAAGTGA

>Cavia_porcellus_intact_V1R615

ATGTCATCCATGGATCTGAAATTAGTAATCCTGTTCCTTTTCCCTGTTGTCATTGGAAGCCTGGGGAATCTCATTCTTTTATATCACTTTCTCTGCCTTTACTGCAGGGGAGGCAGGTCCAGGTCCATGGATGTGATTCTCATGCAACTAACTGTGGCCAACTCCTTGTTCATTCTTTCCAGAGGAATCCCACAGATCCTGGAAGCTTTTGAGATAGAAGTCTTCATTACTACCCTTGGATGCAAACTCCTTTTCTATGTTTACAGAGTGGCTAGAGGTGCATCTTTCAGCATCACCTGCCTGCTGAGTGTCTTTCAGGAAATCATCATCAGCCCCAAAAGCTCCAAGTTTGCAAAACTGAAAGCTAAAACCCTAAAATACATTGGACCCTGTGCCATAATTTGCTGGGTCTTGCAAATGCTGCTAAACATCAGAGTTCCTATGCTTGTAAGAGAGAAAAGGAACAGTGAAAACATCTCAAACACTATAGATTTTCATTATTGCTCAGTGATGAGTTCTGACAAAGAAAAATCCTCCATCTTTGTGGTACTGGAATTATCTCATGATGTTTTGTGCTTGAGACTTATGATCTGGAGCACTGGCTCCATGGTTTTGATTCTGAACAGGCACAAGCAGCGAACACAGCACATTCACAGACAAAACAGCTCCTCAAGATCTTCCCCTGAGACCAGAGCTTCTCAAAGCATCCTCCTCCTAGTCTGTGCCTTTGTGTCCTTTCATGCCCTATCTTTTATCACAAATCTTTGGTTTTCTCTTTATTACAGATATTCTTGGTGGCTGGTTAAAACTTCTGACTTAACCTATTGGTGTTTCCCTACTGTTAGTCCCTTCATTTTCATGACACGTGAGCAGTGTGTGTGCAGGTCCATGTGGAAAAAGTGA

>Cavia_porcellus_intact_V1R616

ATGACATCTCGGGACATGGTAATAGGAATTGCCTTTCTGATTCAGACAGTCACTGGAATCCTAGCTAATATTGTGCTTTTGTTCCATTATCTCTTTCTCTACTTCACTGGTTACAAGTTCAGGCCTTCGGATCTAATAGCTGAGCACTTGAATGTCGCAAACACACTGATCATGCTCTCTAAGGGAATCTCACAGACAGTACAAGCTTTTGGAATACAGTATTTCTCTCCAGGTAATAGATGTTCATTTGTTTTGTACATCTACAGAGTGGCCCGGGGTGTGTCAGTTAGTGCCACTTGTCTGCTGAGTGTCTTCCAGACCATTAAGATAAGCCCCATAAACTCCATTTGGCAGGATCTTAAAGTTAAACTTTCCAAACGTATTGGCTTCTTCACTTTCCTTAGCTGGATTCTCCATATATTGGTTAATTGTATTTTTCCATTTTATAGTGTCATGAAATATAGCAGTAAAAATATCACAAAGAGAAAATATTTTGCGCACTGCTTTTCTGGATCTCAAGACAAAACCGCAGAGTTATTGTATGCAGCCTTTGTGTTATTCCCTGAAGTTTCATTTTCTGGGCTCATGATCTGGTCCAGTGGCTCCATGATTTTCATTTTGCACAGGCACAAGCAGCAGGTCCAGCACATCCGCAGGACAAATGAATCCCACAAGTCATCCCCTGTGTCCAGGGCCACACAAAATGTCCTTGTCCTTGTGTGCACCTTTGTGTCTTTCTACACCCTCTCCTCATTCTTCTATGTTTGTTCCATCAATTTGAATGTACCTAGTTGGTGGCTCATAAACACCTCTGATCTAATTTCCACCTTGTTCCCCACTGTCAGCCCTTTCGTTCTAATGAGTTGTAACTCCACTGCGGCCAGACTCTGGTTCAGATTATTAAATGTTTTCAGAAGCAGGTAG

>Cavia_porcellus_intact_V1R617

ATGAAACCCTTGGGCTTGCTCATTGGAATGGTTTTTCTGATTCAGACAGTCACTGGATTCTTAGCTAACATTGTCCTTTTGTTCCACTATCTCTCTCTGTACTTCACTGCGTATAAGTTAAGGCCTACAGATCTAATAGCAGAACACTTAACTATTGCCAACACACTGATCATGCTGTCAAGGGGAACCCCGCAGACAATGCAAGGCTTTGGGCTAAAGTATTTCTGTCAAGTTAGTAGATGCACACTTCTTTTATATGTCTACAGAGTGGCAAGGAGTGTATCAGTTAGCACAACCTGCATGCTGAGTGTCTTCCAGACTATTAAGATTAGCCCCATGAATTCCAGCTGGAAGGAGCTTAAAGCTAAACTTCCCAAGAACATTGGCTTGCTGACTTGTTTCAGTTGGATTCTCCATATATTGATAAATTGTATTTTTCTATTTTATGACATCATCAAGTATACCAGCAAAAATATCACAGAGTTAAAAGATTTTGGATACTGTTCTTGTATTCTTAGAGATGAAATCATAGAAATATTATACACAGCATTTGTCTTGTTGCCTGAAGGTTTATGTTCTGGCCTCATGATCTGGTCCAGTGGTTCAATGATTTTCTTTTTGCATAGGCACAAGCACTGTATGCAATATATTCATAAGACAAAGGATTTCCACAAAACTTGCCCTGAGACCAGGGCCACCCACAGCATCTTCATCCTTGTATGCACATTTATTTCTTTTTACACCCTATCCTCATTCTTTTATGCTTGCATTGCAAATTTCAGAAATCCTAGTTGGTGGCTCATGAAAACCTCTGCATTCTTTGCCACCTGCTTCCCCACTGTCAGCCCATTAGTACTAATGAGCTGCAACTCTACTGCAGCCAGACTCTGGATCAAACTATCAGGTTTTTTCAGAAGCATGTAA

>Cavia_porcellus_intact_V1R618

ATGAAACCTAGGAGCTTGCTCATTGGAATGGTTTTTCTGATTCAAACAGTCACTGGAATCTTAGCTAACATTGTCCTTCTGTTCCACTATCTCTCTCTGTGCTTCACTGCATATAAGTTAAGGCCTACAGATCTAATAGCAGAACACTTAACTATAGCCAACACACTGATCATGCTGTCAAAGGGAATCCCACAGACAATGCAAGGCTTTGGGCTAAAGTATTTCTGTCAAGTTAGTAGATGCACACTTCTTTTATATGTCTACAGAGTGGCAAGGGGTGTCTCAGTTAGCACAACCTGCCTGCTGAGTGTCTTCCAGACTATTAAGATCAGCCCTATGAACTCCATTTGGAAGGAGCTTAAAGGTAAACATCCCAAGAACATTGGTTTATTGATTTTTTTCAGTTGGATTCTCTATACGCTGGTAAATTGTATTTTTATATTTTATGGCATCATCAAATATACCAGCATAAATATCACAGAGTTAGAAGACTTTGGATACTGTTCTTGTATTTTTAGGGATGAAATCATAGAAGCATTATATACAGCATTTGTCTTGTTCCCTGAAGTTTCATGTTCTGTGCTTATGATCTGGTCCAGTGGCTCCATGATTTACTTTTTGCACCAGCACAAGCAGCGGATGCAATATATTCATAAGACAAATGATTTCCAGAAAACTTGCCCTGAGACCAGGGCCACCCACAGCATCTTCATCCTTGTATGCACCTTTGTTTCTTTTTACACCCTCTCCTCATTCTTTTATGCTTGCATTGCAAATTTCAGAAATCCTAGTTGGTGGCTCATGAACACCTCCGCACTGTTTGCCACCTGCTTCCCCACGGTCAGCCCATTCGTTCTAATGAGCTGCAACTCTACTGCAGCCAGACTCCGGATCAAATTATCAGGTTTTTTCAGCAGCATGTAA

>Cavia_porcellus_intact_V1R620

ATGGATCTGAAATTAGTAATCCTCTTCCTTTTCCCAGTTGTGATTGGAAGCCTGGGGAATCTCTCACTTCTATGTCATTCTGTCTTCCTTTACTGCAGTGGACACAGGTCAAGATCCATAGATGTGATTGTCAGGCATCTGACTGTGGCTAACACCCTGTTCCTTCTCTCCAGAGGAATCCCACAGATCATGGCAGCATTTGGGAAGCAAGACTTCCTAAATAACGTTGGATGCAAACTAGTTTTCTATCTTCAGGTAACAGCAAAGGGTGTATCTGTCAGCACCACCTGCATGCTGAGTGTCTTCCAAGCCATCACCATTAGCCCGAGGAGTTGTGGGTGGGCAGAGCTGAAATTGAAAGCTGCAAAGCACATTGGGCGAAGTGTCATCATGTTCTGGGTCCTGCACATGCTGCTAAACATCAGAGTTCCTATTATTGTGAGTGACAAAAGGAACAATACAAACATTATGAACATCATAGATTTTCAGTATTGTTCTGTTATGATATCTAGCAAAGAAAAAACGACTACCTTTGTGGCACTGACTTTATCCCATGACATTTTGTGTTTGAAACTTATGATCTGGAGCAGCAGCTCCATGATTTTCATTCTGTACAGGCACAAGAAGCGAACACAGCATATTCATCATTCTAGCATCTCATCAAGAGCTTCCCCTGAGACCAGAGCCTCTCAAAGCATCCTCATCCTGGTCTGTACTTTTGTATCTTTCTATGCCCTGTCTTCCCTCATGTACGTTTGGTTTTCTCTTTATATCAAAGCTGCTTGGTGGCTGGTTAAAACTTCTGCCTTAACCAATGCTGGTTTCCCTACTGTTAGTCCATTCATTCTCATGACTCGTGAACAGTTTGTTTGTGAGTCCACGAGGAAGAAGTGA

>Cavia_porcellus_intact_V1R621

ATGGATTTGAAATTTGTAATCCTCTTCTTTTTCCCAGTTGTCATTGGAATCCTGGGGAATCTATCCCTTTTACATCATTCTATTTTCACTTACTTCAGTGGACACCCGACCAGGCCAACAGACTTTATTCTCAGGCACCTGATAGTGGCCAATCTCTTGGTCATTCTCTTCCAAGGGATACCAGAGACCATGGCAGCACTTGGGATGGAAGACTTCCTTGATAATTTTGGATGCAAACTTGTTTTTTATGTTTTAACGGTGGGCAATGGTGTGTCTTTCAGCAACACCTGCCTGATGAGTGTCTTCCAGGCCATCACCATCAGCCCCCGGAGGTCCAGGTGGGCAGAATTGAAAGTGAGAGTCCTAAAGTGCATTAGGCACTGTCCCAGCATTTGCTGGGTCCTGCACCTGCTGGCAAACATTAGAGTTCCTATGCTTCTGAATGAAAAAAGGCGCAATGACAACATCACAAACTCTATAGATTATCAATACTGTTCAGCTGTGATTTCTAGCAAAGAAAAAAACTCCATCTTGGTGGCATTGTCATTATCACATGACATTTTCTGTTTGGAACTTATGATCTGGAGCAGTGGCTCCATGGTTTTCATTTTGTATCAGCACAGGCAGCGAACTCAGTACATTCACAGACACAACAGCTCATCACGATCTTCCCCTGAGACCAGAGCTTCGCAAAGCATCCTCATGCTTGTCTGTGCCTTTGTATTATTCCATGCCCTGACTTCTATAATGACTGTTTGGTTTTATCTTTATGACAAAGCTGCTTGGTGGCTGGTTAAAACTTCTGCCCTAACCCATGTTTATTTTCCAACTATTACTCCTTTTATTTTCATGACTCGCGAACAGGGTATCTGCAGACCCATGTGGAAGAAGTGA

>Cavia_porcellus_intact_V1R622

ATGACATCTGTGGATCTAAAATTTTTCACCCTCCTACTTTTCCCCATTTGCATTGGAAGCCTGGGGAATCTCTCACTTTTATGCCAATATGTCTTCATTTACTTCAGTGGAAGCAGGTCTAGGCCAACAGATTTTATTCTCACAAACCTGACAGTGGCCAACCTGTTAGTCATTCTCTCCCGAGGAATCCCAGACACCATGTCAGCTCTTGGGATGAAAGACTTTCTTAATAATTTTGGGTGCAAACTTGTTTTCTATGTTTTAATGGTGGGCAAGGGTGTGTCTTTCAGCACCACCTGCTTGCTGAGTGTCTTCCAGGCCATCACCATCAGCCCCAGGAGCACCAGGTGGGCAGAATTGAAAGTGAAAGCCCTAAAATACATTGGGCACTGTACCATCCTTTTCTGGGTCCTGCACATGCTGGCAAACATCAGAGTTCCTATGCTTGTGAGTGAAAAAAGCAACAATGAAAATTTAACAAACACTATAGATTATCAATACTGTTCAGCTACAACTACTCCCAAGGACAAAAATACCATTTTTGCATCATTGTCTTTATCACATGATATTTTGTGTTTGAAATTTATGATCTGGAGCAGTGGCTCCATGGTTTTCATTCTATACAGGCACAAGCAGCGAACAAAGCACATTCACAGATACAACAGTTCCTCGAGAACTTCCCCCGAAACCAGAGCTTCTCAAAGCATCTTCATCTTGGTCTGTGCCTTTGTTTCCTTTTATGCCCTGTCTTCCATCACATACATTTGGTTTTCTATTTATGACAGATCTGCTTGGTGGTTGGTTAAAACTTCTGCCTTAACCAATGCTTGTTTCCCTACTGCTAGTCCATTTATTCTCATGACTCGTGAACACTGTGTATGCAGGTGCATGTGGAAGAAGTGA

>Cavia_porcellus_intact_V1R623

ATGACATCTGTGGATCTAAAATTTTTCACCCTCCTACTTTTCCCCATTTGCATTGGAAGCCTGGGGAATCTCTCACTTTTATGCCAATATGTCTTCATTTACTTCAGTGGAAGCAGGTCTAGGCCAACAGATTTTATTCTCACAAACCTGACAGTGGCCAACCTGTTAGTCATTCTCTCCCGAGGAATCCCAGACACCATGTCAGCTCTTGGGATGAAAGACTTTCTTAATAATTTTGGGTGCAAACTTGTTTTCTATGTTTTAATGGTGGGCAAGGGTGTGTCTTTCAGCACCACCTGCCTGCTGAGTGTCTTCCAGGCCATCACCATCAGCCCCAGGAGCACCAGGTGGACAGAATTGAAAGTGAAAGCCATAAAATACATTGGGCACTGTACCATCCTTTTCTGGGTCCTGCACATGCTGGCAAACATCAGAGTTCCTATGCTTGTGAGTGAAAAAACCAACAATGAAAATTTAACAAACACTATAAATTATCTATACTGTTCAGCTACAACTACTACCCAGGACAAAAATACCATTTTTGCATCATTGTCTTTATCACATGATATTTTGTGGTTGAAACTTATGATCTGGAGCAGTGGCTCCATGGTTTTCATTCTATACAGGCACAAGCAGCGAACAAAGCACATTCACAGATACAACAGTTCCTCGAGAACTTCCCCTGAAACCAGAGCTTCTCAAAGCATCTTCATCTTGGTCTGTGCCTTTGTTTCCTTTTATGCCCTGTCTTCCATCACATACATTTGGTTTTCTATTTATGACAGATCTGCTTGGTGGTTGGTTAAAACTTCTGCCTTAACCAATGCTTGTTTCCCTACTGCTAGTCCATTTATTCTCATGACTCGTGAACACTGTGTATGCAGGTGCATGTGGAAGAAGTGA

>Cavia_porcellus_intact_V1R624

ATGGATTTGAAATTTGGGATAATATTTCTTTTCCTGGTTGTCATTGGAACCATGGGCAATTTCTCACTGTTATGTTATTATATTATCCTCCACTTCAATGGATACAGGTCAAGGTCAACAGATTTGTTTCTCAGACACCTGATTGTGGCCAACTCCTTAGTTATTCTCTCCAGAGGGATCCAAGAGGTCATGGTGGCTCTTGGGAAGGATTACGTCCTCGACAATCTGGGATGTGAGTTTGTTTTTTATCTTCACAGGGTGGGCAGGGGTGTGTCCATTGACTCCACCTGTCTTTTAAGTGTCTTCCAGGCCATCATCATTAGCCCCAGGGACTACATCTGGGCAAAACTGAAACAGAAAGCCGTAAAACTCATGGGCCCTTCTATCATCCTCTGCTGGGTCCTGCACCTGCTGCTAGCTAGTAGAACTTTTGTGCTTATCACTAATAAAGGGAACAAGAAAAACTTCTCAAGAACTATATATTTCCAGCATTGTTCAGTGATGTGTCCTGGCAATGACACAGGCACGGTTTTTGCAGCAGTGACGTTATCCCATGACATTTTGTGTTTAAATCTCATGATATGGGCCAGTGGCTCCATGGTGTTCATTCTTCACAGGCACAAGCAGCGGGTCCACCACATCCACAGACACAGTTCAGGTAAATCTCCTGCTGAGACCAGAGCTTCTCAAAACATCCTTGCTTTGGTGAGCACCTTTGTGTTCTTCTACACCCTGTCTTCTACCCTCCATGTTTGCTTTGCTCTTACTGAGAAAACTGCTTTGTGGCTGTTCATAACTATCCTAATAAGTGCCTGCTTCCCCACCATTAGCCCCTTTATTCTCTTGAGTCGTGAATGCAGGGGAACTAGGCTTACCTGGAAGAAGTGA

>Cavia_porcellus_intact_V1R625

ATGACATCTGGGAACATGGTATTAGGAACTGCCTTTCTGATTCAGACAGTCACTGGAATCCTAGCTAATATTGTGCTTCTGTTCCATTATCTCTTTCTCTGCTTCACTGGTTACAAGTTCAGGCCTACGGATCTAATAGCTAAGCACTTGACTGTTGCAAACACACTGATCATGCTCTCTAAAGGAATCTCACAGACAGTACAAGCTTTTGGAATACAGTATTTCTCTCCAGGTAATAGATGCTCATTTGTTTTGTACATCTACAGAGTGGCCCGGGGTGTGTCAGTTAGTGCCACTTGTCTGCTGAGTGTCTTCCAGACCATTAAGATCAGCCCCATAAACTCCATTTGGCAGGATCTTAAAGTTAAACTTCACAAGCATATTGGCTTCTGGACTTTCCTCAGCTGGATTCTCCATATATTAGTTAATTGTATTTTTCCATTTTATAATCTCATAAAATATAGCAGTAAAAATATCACAAAGAGAAAAGATTTTGGGTACTGCTCTTCTGGACCTCAAGACAAAACTACAGAGTCATTGTATGCAGCATTTGTGTTATTCCCTGAAGTTTCATGGTCTGGGCTCATGATCTGGTCCAGTGGCTCCATGATTTTCATTTTGCACAGGCACAAGCAGCAGGTCCAGCACATCCGCAGGACAAATGAATCCCACAAGTCATCCCCGGAGTCCAGGGCCACACAAAGTGTCCTTGTCCTCGTGTGCACCTTTGTGTCTTTTTATACCCTCTCCTCATTCTTCTATGTTTGTTTCATCAATTTCAGTGTACCTACTTGGTGGCTCATAAACACCTCTGATCTGATTTCCACCTGGTTCCCCACTGTCAGCCCTTTCGTTCTAATGAGTTGTAACTCCACTGCGGCCAGACTCTGGTTCAGATTATTAAATTTTTTCAGAAGCAGGTAA

>Cavia_porcellus_intact_V1R626

ATGGCTGCCAGTGAAGTGGTTACAGGAGTGATCTTCTTATCACAGATTTTGATTGGAGTTCTGGGCAATTCCTCTCTTCTCTATCATTATCTCTTCCTTTACTTCACTGGGTGCAGATTAAAGTGCACAGATCTTATTCTTAAGCACTTGATTGTTGCCAACCTCCTAACTCTCTTGTGTAGAGGAGTGCCCCACACCATGGAAGCATTTGGTTGGCAAGTTTCCCTTGGTAATGTGGGCTGCAAGCTACTCTTCTATTTCCACAGAGTGGGTAGGGGAGGGACCATCGGCATCATCTGTTTCCTGAATGTTTTCCAGGCCATCACCATCAGCCCAAGGAACTCCAAATGGGCAGAGCTTAAACTGAAGGCTTCCAAGTATTTTGGCTCAACCTTGTGCCTCATCTGGGTCCTGTACAGCCTTGTAAATGTTATTTTTCTTATGTACATGACTGGAATTTGGAACAAGGAAAATCACACAGGCCTAAAATATTATGGGTACTGTTCTAGTGTTCGTCATGACAAAACTTCAGAGTCATTACATGCAGCCCTGCTGTTCCTCCCTGATGCTTTATGTGTGGGGCTCATGATCTGCACCAGCAGCTCCATGGTTTCTGTCCTGTACAGGCACAGGCAGAAGATGAGACATATCCACAGGATTAATGTAAGCCCCAGATCTTCTCCTGAGTCCAGAGCAACCAAACACGTCCTTCTTTTGGTCCTCACCTATGTCTGTTTTTACACTCTCTCCTGCATCTTTCAAGTTTATTTGTCTCTTATTTATAATCCCACCTCTTTACTGGTGAATATATCTCTAATAGTTGCTGGGTTTTTCCCAGTTGTCAGCCCTGTTCTGCTCTTGAGATGTGAATCCAGAGTATCCATGTTCTGCTTTGCCTGTGTAAGGAATGGAAAATCCCCTATTCATATGACAAATTCGTAA

>Cavia_porcellus_intact_V1R627

ATGGGTAGGATGGCAGCCCTTGAAATGGTCATAGGAGTGATCCTCTTTTCACAGACTGTGATGGGGGTTTTGGGCAATTCCTCTCTTCTCTACCAGTATCTATTCCTTTACTTCATTGGGTGCAGGTTAAAGTGCACAGACCTGATTCTTAATCACTTGATTTTTGCCAACATCTTAACCCTCCTGTGTAGAGGAGTACCCCACACCATGGAAGCTTTTGGATGGCAAGTTTCCCTTGGTAATGTGGGGTGCAAAATACTCTTCTACCTACATAGAGTGGGCAGAGGAGGGTCCATCTGCACCATCTGTTTCCTGAGTGTCTTCCAGGCCATCAGCATCAGCCCCAGGAATTCCAAATGGGCAGAGCTTAAAATGAAGGCTTCCAAGTATGTTGACTTCACCTTGTGCCTTATCTGGGTCCTGTACAGCCTTGTAAATGTTATTTTTCTTAAGTATGTAACTGGAAATTGGAAAAACGAAAATCACACAAGCCTCAAATATCATGGGTACTGTTCTAGTATTCGTCATGACAAAATTTCAGAGTCATTATATGCAGCCCTGCTGTCCCTCCCTGATGCTTTGTGTGTGGGGCTCATGCTCTGCTCCAGCATCTCCATGGTTTCCATGCTGTACAGGCACAGGCGGCAGATGAGACAGATCCACAGGACTAATGCTAGCCCCAGATCTTGTCCTGAGTCCAGAGCAACCAAACACATCCTTCTCCTGATCATCTCCTATGTCTGTTTTTACACACTCTCCTGCATATTTCAAGTTTGGTCGTCTTTTACATATAATCCCACCTTTTTCCTGTTGAAAATATCTGCAATAGTTTCTGGGTGTTTCCCAGCTATCAGCCCTCTTCTGCTCTTGAGGTGTGAATCCAGAGAATCCAGGTTCTGTTTTGCCTGTATAAGGAATGGAAAAGCACCTATTTACATGACAAATGGGTAA

>Cavia_porcellus_intact_V1R628

ATGGCAGCCAGTGAAGTGGTTGCAGGAGTGATCTTCTTATCACAGATTTTGATTGGAGTTCTGGGCAATTCCTCTCTTCTCTATCATTATCTCTTCCTTTACTTCACTGGGTGCAGATTAAAGTGCACAGATCTTACTCTTAAGCACTTGATTGTTGCCAACCTCCTAACTCTCCTGTGTAGAGGAGTGTCCCACACCATGGAAGCATTTGGTTGGCAAGTTTCCCTTGGTAATGTGGGCTGCAAGCTACTCTTCTATTTCCACAGAGTGGGTAGGGGAGGGACCATCGGCATCATCTGTTTCCTGAGTGTTTTCCAGGCCATCACCATCAGCCCAAGGAACTCCAAATGGGCAGAGCTTAAACTGAAGGCTTCCAAGTATTTTGGCTCAACCTTGTGCCTCATCTGGGTCCTGTACAGCCTTGTAAATGTTATTTTTCTTATGTACATGACTGGAACTTGGAACAAGGAAAATCACACAGGCCTAAAATATTATGGGTACTGTTCTAGTGTTCGTCACGACAAAACTTCAGAGTCATTACATGCAGCCCTGCTGTCCCTCCCTGATGCTTTATGTGTGGGGCTCATGATCGGCACCAGCAGCTCTATGGTTTCTGTCCTGTACAGGCACAGGCAGAAGATGAGACATATCCACAAGACTAATGTGAGCCCCAGATCTTCTCCTGAGTCCAGAGCAACAAAACACGTCCTTCTTTTGGTCAGCTCCTATGTCTGTTTTTACACTCTCTCCTGCATCTTTCAAGTTTGTTTGTCTCTTATTTATAATCCCACCTCTTTCCTGGTGAACATATCTTCAATAGTTGCTGGGTTTTTCCCAGCTGTCAGCCCTCTTCTGCTCTTGAGATGTGAATCCAGAGTATCCATGTTCTGCTTTGCCTGTGTAAGGAATGGAAAAGCCCCTATTCATATGACAAATTCATAA

>Cavia_porcellus_intact_V1R629

ATGGGTAGGATGGCAGCCCTTGAAGTGGTCATAGGAGTGATCCTCTTTTCACAGACTGTGATGGGGGTTTTGGGCAATTCCTCTCTTCTCTACCAGTATCTATTCCTTTACTTCATTGGGTGCAGGTTAAAGTGCACAGACCTGATTCTTAATCACTTGATTTTTGCCAACCTCTTAACCCTCCTGTGTAGAGGAGTACCCCACACCATGGAAGCTTTTGGCTGGCAAGTTTCCCTTGGTAATGTGGGGTGCAAAATACTCTGCTACCTACAAAGAGTGGGCAGGGGAGGGTCCATCTGCACCATCTGTTTCCTGAGTGTCTTCCAGGCCATCAGCATCAGCCCCAGGAATTCCAAATGGGCAGAGCTTAAAATGAAGGCTTCCAAGTATGTTGACTTCACCTTGTGCCTTATCTGGGTCCTGTACAGCCTTATAAATGTTAGTTTTCTTAAGTATGTAACTGGAAATTGGAAAAACGAAAATCACACAAGCCTCAAATATCATGGATACTGTTCTAGTATTCGTCATGACAAAATTGCAGAGTCATTATATGCAGCCCTGCTGTCCCTCCCTGATGCTTTGTGTGTGGGGCTCATGCTCTGCTCCAGCATCTCCATGGTTTCCATGCTGTACAGGCACAGGCGGCAGATGAGACAGATCCACAGGACTAATGCTAGCCCCAGATCTTGTCCTGAATCCAGAGCAACCAAACACATCCTTCTCCTGGTCATCTCCTATGTCTGTTTTTACACACTCTCCTGCATCTTTCAAGTTTGGTTGTCTTTTACATATAATCCCACCTTTTTCCTGTTGAAAATATCTGCAATAGTTTCTGGGTGTTTCCCAGCTATCAGCCCTCTTCTGCTCTTGAGGTGTGAATCCAGAGCATCCAGGTTCTGTTTTGCCTGTATAAGGAATGGAAAAGCACCTATTTACATGACAAATGGGTAA

>Cavia_porcellus_intact_V1R630

ATGGCTGCCAGTGAAGTGGTTACAGGAGTGATCTTCTTATCACAGATTTTGATTGGAGTTCTGGGCAATTCCTCTCTTCTCTATCATTATCTCTTCCTTTACTTCACTGGGTGCAGATTAAAGTGCACAGATCTTATTCTTAAGCACTTGATTGTTGCCAACCTCCTAACTCTCTTGTGTAGAGGAGTGCCCCACACCATGGAAGCATTTGGTTGGCAAGTTTCCCTTGGTAATGTGGGCTGCAAGCTACTCTTCTATTTCCACAGAGTGGGTAGGGGAGGGACCATCGGCATCATCTGTTTCCTGAATGTTTTCCAGGCCATCACCATCAGCCCAAGGAACTCCAAATGGGCAGAGCTTAAACTGAAGGCTTCCAAGTATTTTGGCTTAACCTTGTGCCTCATCTGGGTCCTGTACAGCCTTGTAAATGTTATTTTTCTTATGTACATGACTGGAATTTGGAACAAGGAAAATCACACAGGCCTAAAATATTATGGGTACTGTTCTAGTGTTCGTCACGACAAAACTTCAGAGTCATTACATGCAGCCCTGCTGTCCCTCCCTGATGCTTTATGTGTGGGGCTCATGATTGGCACCAGCAGCTCCATGGTTTCTGTCCTGTACAGGCACAGGCAGAAGATGAGACATATCCACAAGACTAACGTGAGCCCCAGATCTTCTCCTGAGTCCAGAGCAACAAAACACGTCCTTCTTTTGGTCCTCACCTATGTCTGTTTTTACACTCTCTCCTGCATCTTTCAAGTTTGTTTGTCTCTTATTTATAATCCCACCTCTTTCCTGGTGAACATATCTTCAATAGTTGCTGGGTTTTTCCCAGCTGTCAGCCCTCTTCTGCTCTTGAGATGTGAATCCAGAGTATCCAAGTTCTGCTTTGCCTGTGTAAGGAATGGAAAAGCCCCTATTCATATGACAAATTCGTAA

>Cavia_porcellus_intact_V1R631

ATGGGTAGGATGGCAGCCCTTGAAGTGGTCATAGGCGTGATCTTCTTATCACAGACTGTGATGGGGGTTTTGGGCAATTCCTCTCTTCTCTACCAGTATCTATTCCTTTACTTCATTGGGTGCAAGTTAAAGTGCACAGACCTGATTCTTAATCACTTGATTTTTGCCAATCTCTTAACTCTCCTGTGTAGAGGAGTACCCCACACCATGGAAGCTTTTGGCTGGCAAGTTTCCCTTGGTAATGTGGGGTGCAAACTACTCTTCTACCTACACAGAGTGGGCAGGGGAGGGTCCATCTGCACCATCTGTTTCCTGAGTGTCTTCCAGGCCATCAGCATCAGCCCCAGGAATTCCAAATGGGCAGAGCTTAAAATGAAGGCTTCCAAGTATGTTGACTTCACCTTGTGCCTTATCTGGGTCCTGTACAGCCTTGTAAATGTTGTTTTTCTTAAGTATGTAACTGGAAATTGGAAAAACGAAAATCACACAAGCCTCAAATATCATGGGTACTGTTCTAGTATTCGTCATGACAAAATTTCAGAGTCATTATATGCAGCCCTGCTGTCCCTCCCTGATGCTTTGTGTGTGGGGCTCATGCTCTGCTCCAGCATCTCCATGGTTTCCATGCTGTACAGGCACAAGCGGCAGATGAGACAGATCCACAGGACTAATGCTAGCCCCAGATCTTGTCCTGAGTCCAGAGCAACCAAACACATCCTTCTCCTGGTCATCTCCTATGTCTGTTTTTACACACTCTCTTGCATCTTTCAAGTTTGGTTGTCTTTTACATATAATCCCACCTTTTTCCTGTTGAACATATCTGCAATACTTTCTGGGTGTTTCCCAGCTATCAGCCCTCTTCTGCTCTTGAGGTGTGAATCCAGAGCATCCAGGTTCTGA

>Cavia_porcellus_intact_V1R632

ATGGCAGCCAGTGAAGTGGTTGTAGGAGTGATCTTCTTATCACAGACTGTGATTGGGGTTCTTGGCAATTTGTCTCTTCTTTACCAGTATTTCTTTCTTTACTTCACGGGGTGCAGGTTAAAGTGCACAGACCTGATTCTTAAGCACTTGATTGTTGCCAATCTCTTAACTCTCCTGTGTAGAGGAGTACCCCACACCATAGAAGCATTTGGTTGGCAAGTTTCCCTTGGTAACATGGGGTGCAAACTACTCATCTATCTACACAGAGTGGGCAGGGGAGGGTCCATTGGCACCATCTGTTTCCTGAGTGTCTTTCAGGCCATCACCATCAGCCCTAAGAACTACAAGTGGGCAGAGATTAAAATGAAGGCTTCCAAGTATGCTGGCTCCACTTTGTGCCTCATCTGGGTCCTGTACAGCCTTGTAAATGTTGTTTTTCTTATGTACATTACTGGAAATTCCACCAACAAAAATCACACAGGTCTAAAAAGTCATGGATACTGTTCCAGTATTCGTCACGATAAAACCGCAGATTTATTATATATAGCCTTGTTGTCATTCCCTGATGCTTTATGTTTGGGGCTCATGCTCTGCACCAGCAGCTCCATGGTTTCCATCCTGTACAAGCACAGGCAGCAGATGAGAGACATCCACAGGACTAATGTCAGCCTTGCATCTTCTCCAGAGTCCAGAGCGACGAAACACATCCTTTTCTTGGTCAGCTTCTATGTCTGTTTTTACACACTATCCTGCATCTTTCAAGCCTTTATGTCTTTTACGTATAATCCCAACCTATTCCTAGTGTACTCATCTGCAATAGTTGCTGGGTGTTTCCCAGCTGTCAGCCCTCTTCTGTTCTTGAGGTGTGAATCTAGAGTATCCAGGTTCTGCTTTGACTGTATAAGGAATGGAAAAGCTCCTTTTAATATGACAAATGGGTAA

>Cavia_porcellus_intact_V1R633

ATGTCAGCCAATGAAGTTGTTGTAGGTGTCATCTTCTTATCACAAACTGTGATTGGGGTTCTGGGCAATTCCTCTCTTCTGTACCATTATCTCTTCCTTTACTTCACTGGGTGCAGGTTAAAGTGTACAGACCTGATTCTTAATCACTTGATTTTTGCCAACTTCTTAACTCTCCTGTGTAGAGGAGTACCCCACACCATGGAAGCATTTGGTTGGAAAGTTTTCCTTGGTAATGTGGGGTGCAAACTACTCTTCTACCTACACAGAATGGGTAGAGGAGGGTCCATTGGAACCATCTGTTTCCTGAGTGTCTTCCAGGCCATCACCATCAGCCCCAGGAACTCCAAGTTGGCAGAGTTTAAAATGAAGGTTTCCAAGTATGCTGGCTCCACTTTGTGCCTCATCTGGGTCCTGTACAGCCTTGTAAATGTTATTTTTCTTATGTACATGACTGGGAATTCAATGAAGGAAAATCACACAGGCCTAAAATATCATGCATACTGTTCCAGTGTTCGTCCCGACAAAACTGCAGAGTCATTATATGCAGCCCTGCTGTCCATCCCTGATGCTTTGTGTGTGGGGCTCATGCTCTGCACCAGCAGCTCCATGGTTTCTGTCCTGTACAGGCACAGGCAGCAGATGCGATGGATCCACAGGACTAATGTGAACCCTGGATCTTCTCCTGAGTCCAGAGCAACCAAACACATTCTTCTCCTGGTCAGCTCCTATGTTTGTTTTTACACACTCTCCTGCATCTTTCAAGTTTTGTTTTCTCTTACTTATAATCCCAACATTTTCCTGGTGAACATATGTGCCATAGTTGCTGGGTTTTTCCCATCTGTCAGCCCTCTGCTGCTGTTGAAGTGTGAATGCAGAGTATCCAGGTTTTGCTTTGCCTGTATAAGGAATGGAAAATCACCTATGTATATGACAAATGGGTAA

>Cavia_porcellus_intact_V1R635

ATGCCTTCCAAGGATTTGGCAATAGGAATAATGTTTTTGTTACAGACTGTGATTGGAATTCATGGAAATTTTTCTCTTCTTTATTATTATTTTTTCCTTCACCATATGGAAAGTAGAATGAAGCCCATAGATCTGATTCTCAAGCATCTTCTTATAGCTAATTCTTTAGTTATTCTTACAAATGGACTATCCAGGACAATGGTAGCCTTTGGGTTGAAATATTTATTCAATGAACTTATTTGCAAGCTTAACTGGTTTATGCTCAGAGTGGGCAGGGCTATGTCAATTTCTACCATGTGCTTCTTGAGTTTCTTCCAGAACATCACACTCAGCCCCATGAACTCCTGTTGGAATAATCTTAAAATCAAAGATTCCAAGTACATTGACTTCTCCATGTCTCTCTGCTGGCTGCTACACTTGGGGCTAAATTGCATTTTCCCTCTGTATGTGCTGCATGCATTGGGAAATTCAGAAAGCAAAAACATCACAAGGAAAAGACATCTGGGAATTTGTTCTGTCGTAGATTATGGAACAACCATGGGCTCAGTCTATATTGCATTAGTAGTATCTCCAGAGATTTCTTTTATTATGCTCACAGTATGGGCCAGTGGCTCAATGATTCTTGTCCTGTACAGACATAAAGCGCAGGTTAAGCACATTTATAGTAGTAAAGTTTCCTCCAGATCCCCTGAGTCAAGAGCCACCAAAAGCATCCTTCTGCTGGTGAGCACCTTTGTATCATTTTACACCATCTCCTCCATTTTTAACATTTATACTGCTCTTTTTTATAATCTCAGTTGGTGGTTGATGAGTATTTCTGACCTAACTTCTGTGTGTTTTCCAATGATCAGCCCATTTCTTATCATGAGTCAGAATTCCTCTATATCTGTGTTCCAGTTTGTCTGGATTAGAAATACAAAATCCTCTATTATCAAAAATATATAA

>Cavia_porcellus_intact_V1R636_1

ATGAGGCTGGGTCAAAGAAATGATAGTCACCATACTTTAAACGAAATTTTAGAAGAAAATGACAAAATTCCTTCCAGGGATTTGGCGATAGGGATAATGTTTTTGTCACAGACTGTGATTGGAATTCTGGGAAATTTTTCTCTTCTTTATCGTTATTTTTTCCTTCACCAAATGGAAAGCAAAATGAGGCCCATATATTTGATTCTCAGGCATTTGTTTATAGCTAATTCTTTGACTATTCTTTCAAAAGGACTTCCCCAAACAATGGTAGCCTTTGGGTTGAAGCACTTGTTTAATGATTTTTGTTGCAAACTTAACTTGTATGTGCTCAGAGTTGGCAGAGCTATGTCAGTTTCTACCATGTGCCTCTTGAGTTTCTTCCAGTATATCACTCTTAGCCCCATGAACTCCTGTTGGAAGAATCTTAAAATCAAAGCTGCCAAGTACATTGACTTTTCCATGTCTCTCTGCTGGTTGCTACATATAGGGATAAACTCTATTTTTCCTCTGTATGTGCTGCATATATCTAAAAATCCAGAAAGCAGAAACAACACAAGTATAAGATATATGGAAATCTGTTCCATTTTAGATTATGGGACAAACATGGGCCCAGTCTATATTGCATTAGTAGTGTCCCCAGAGGTTTCTTTTATTGTGATCACAATATGGGCCTGTGGCTCAATGATTCTTCTCCTGTACAGACATAAAATGCAGGTTAAGCACATTCATAGCTCTAATGTTTCCTCCAGATCACCTGAGTCGAGAGCCACCAAAAGCATCCTTTTCCTGGTGAGCACCTTTGTATTATTTTACATCATCTCCTCCATCTTTTACATTTTTGTTGCTCTATTTTATAATTTAAGTTGGTGGTTGAAGAACATTTCTGGCATAATTTCTGTGTGTTTCCCAGTGATCAGCCCCTTTCTTATCATGAAACAGAATTCCTCTATATCTATGTTCTGGTTTGTCTGGATAAGAAACACAAAATTTCCCTTTCTCATGAAAAATACATAA

>Cavia_porcellus_intact_V1R636_2

ATGAGTCTGGTTCAAAGAAATGATTGTCATCACATTTTACAGGAAATATTAGAAGGAAATGGCAAAATCCCTCCCAGGGATTTGGCAGGAGGAATAATATTTTTGTCACAAACTGTGATTGGAATTCTGGGAAATTTTTCTCTTCTCTACCATTATTTTTTCCTTCACAATATGGTTATTAAAATAAGGCCTATATATTTGATTCTCAAGCATTTGCTTATGGCTAATTGTTTGGTTATTTTCACCATTGGACTTTCCCAGACAGTGGTGGCATTTGGGTTCAAACATTTATTCAATGAATTTAGTTGCAAACTTAGTTTGTATGTGCTCAGAGTAGGTAAGGCTATGTCAATTTCTACTATGTTTTTCTTGAGTTTCTTCCAGAATATCACACTCAGCCCAATGAACTCCTGTTGGAAGAATCTCAAAATCAAAGCTCCCAAGTACATTGACTGCTCCATGGCTTTCTGCTGGCTTCTTCACATGGCAGTAAATTGCATTTTTCCTCTGTTTATGCTGCATGTGTCTAAAAATTCAGGAGGCAGAAACATCACAAGAAAAAGATATCTGGGATTCTGCTCTGTTGTAGATTATGGGACAGCCATGAACTTAGTCTATATTGCATTAGTAGTATCCCCAGAGGTTTTGTTTATTGTGCTCACAATATGGGCCTGTGGCTCGATGATTCTTGTTCTGTGCAGACATAAGCAGCAGGTTAAGCATATTCATTGCACTAAAGTTTCCTCCAGATCTCATGAGTCGAGAGCCACCAAAAAGCATTCTTTTCCTAGTGAGCGCTTTTGTATCATTTTACATCATCTCTCCATCTTTTCCATTTTAATGCTCTTTTTTATGAATCAGTGA

>Cavia_porcellus_intact_V1R637

ATGAGCCTGATTCAAAGAAATTATTGTCATCACATTTTAAAGGAAATTTTAGAAAGAAATGACAAAATCCCTTCTAGGGATTTGGTGATAGGAATAATGTTTTTGTTACAGACTGTGGTTGGAATTCTTGGAAATTTTTCTCTTCTTTGCCATTATTTTTTCCTTCACCATATGGAAAGTAGAATGAAGCCCATAGATCTGATCCTCAAGCATATGTTTATAGCTAATTCTTTAATTATTCTTACAAAAGGACTTTCCCAGACGATGGTAGCCTTTGGGTTGAAACATTTATTCAATGAACTTAGTTGCAAGCTTAATTTGTTTGTGCTCAGAGTGGGCAGGGCTATGTCAATTTCTACCATGTGCTTCTTGAGTTCCTCCCAGAATATCACACTTAGCCCCATGAACTCCTTTTGGAAGAATCTTAAAATCAAAGCTGCCAAGTACATTGACTTCTCCATGTCTCTCTGCTGGTTGCTATACATGGGGGTAAATTGCATTTTCCCCCTGTATATGTTGCACCTATCAGGAAATTCAGAAAGCAGAAACATCACAAGAAAAAGACATCTGGGAATTTGTTCTGTGGTAGATTATGGAACAGCCATGGGCTCAGTCTATATTGCATTAGTAGTATTCCCAGAGGTTTCTTTTATTGTGCTCACATTACAGGCCAGTTGTTCAATTATTCTTGTCCTGTACAGACATAAAGAGCAGGTTAAGCATATTCATAGCTCTAAAGTTTCTTCCAGATCTCTTGAGTCAAGAGCCATGAAAAGCATCCTTCTCCTCATGAGCACCTTTGTATCATTTTACATCATCTCCTCCATATTTTCAATTTTTATGGCCATTTATATTAATCTCAGTTGGTGGTTGAAGAGTATTTCTGACTTAATTTCTGTGTGTTTCCCAATGATCAGCCCCTTTCTTGTCATGAGCCAGAATTCCTCTATATCCATGTTCCGGTTAGTCTGGATAAGAAACACAAATATACCTCTTATCGAAAATATATAA

>Cavia_porcellus_intact_V1R638

ATGACAAAAATAGAACCTGTTTCTTTGGTAATAGGAGTAATATTATTGTCACAGACTATGGTTGGAGTTATGGGAAATTTTTTTCTTCTTTACCATTATCTTTTCATTTACCACACAAAAAGCAAGTTGAGATGCATAGAGTTGATTCTCAAGCACATCCTCATAGCCAACTCCTTACTGATTCTTTCTACAGGACTGCCTCAGACAATGGTAGCCTTTGGACTGAAAAATTCTCTCAATGACTTTGTCTGCAAAATTATCTTTTATGTTGAGAGAGTGGGCAGGGGCATGTCAATTGGAACCATCTGCCTCTTGAGTATTTTCCAAACTGTCACAATCAGCCCCATGAATGCCTGCTGCAAAGACCTTAAAATTAAAGCTCCAAAGTATGTAGAATTTTCCATATATCTCTGCTGGTTTGTGCACATGGTGGTAAATTTCATTTTTCCTCTCTATCTGTTATATGTCTCGGGAAGATTGTCTAGCAAAAACATCACAAAGGAAAGAAAAATAAGATTTTGTACTCACACAGATCTTGGGACAATCCTAGGCCCAATCTATGTAGTGTTAGTAGCATTCCCTGAAGTTGCTTGCTCTGTTCTCATGATCTCTGCAAGCAGCTATATGATTTTTACTCTGTATCAACACAAGCAGAGGGTCCAACACATTCACAGGACCAATGTCTTCTTCAGATCCCCTGAGTCCAGAGCCACCAAAAGCATCCTCCTTCTGGTGGGCACTTTTGTATCTTTTTACAGCCTGTCCTCCCTCTTTAACATTTCTATCGCTCTTTTTCCTCAGGCATATGGGAGGCTGGTAATAATTTCTGATATAGTTTCTGTGTGTTTTCCAACTATATCTCCCTTTCTGTTAATCAGCCAGGACTCTTCTATATCCAGGCCCTGCCTTATCTGGATAAGGAATGCACTCTCCATTTACAAGAAATAA

>Cavia_porcellus_intact_V1R639

ATGACACACATAGAACCCGTCAATTTGGTAATAGGAATGATCTTATTTTCACAGATCACGGTTGGAGTCATGGGAAATTTTTTCCTTCTTTACCATTATATTTTCATTTACCACACTAAAAGCAAGTTGAGATGCATAGATTTGATACTCAAGCACATTCTCATAGCCAATTCCTTATTTATTCTTTCTAAAGGACTGCCTCAGACAATGGTAGCATTTGGACTAAAAAATTCTCTCAGTGATTTTGTCTGCAAATTTTTCTTTTATGTTGAGAGAGTGGGCAGGGGTGTGTCAATTGGAACCATCTGCCTCTTGAGTATCTTCCAGAGGATCACAATCAGCCCCATGAACTCCTGCTGGAAAGACCTTAAAATAAAAGCACCAAAGTGTATCGGATTTTCCATTTACCTCTGCTGGTTTGTGCACATGGTGGTAAATTTCATTTTTCCTCTTTATCTGTTGTATATGTCTGGAAGATGGTCTAGCAGAAATATCACAAAGGAAAGAAAAATAATATTCTGTACTTACACAGATCCTGGGACAATCCTGGGCTCAATCTATATAGCGTTAGTAGTATTCCCTGAAGTTGCTTGTTCTGTGCTCATGATCTGTGCAAGCAGTTCCATGGTTTTTAATCTGTACCAACACAAGAAGAAGGTTCAACACATTCACAGGACAAATGTCTTCTCTAGATCCCCTGAGTCCAGAGCCACCAAAAGCATCCTCTTTCTGGTGGGCACTTTTGTATCATTTTACAGCCTGTCCTCCCTTTTTAACATTTCTATTGCTCTTTTTCCTGAGGTATATAGGAGGCTGGTGAGTATTTCTGAGATAATTTCTGTGTGTTTTCCAACTATGAGCCCATTTCTGCTGATGAGCCAGGACTCTTCTATACTCAGGCCCTGCTTTGTTATGTTAAGGAACGCTCTCCCCACTTATAAGAAATAA

>Cavia_porcellus_intact_V1R640

ATGATCTTATTTTCACAGACTATGGTTGGAGTCATGGGAAATTTTTTTCTTCTTTATCATTATCTTTTCATTTACCATACTAAAAGCAAGTTGAGATGTATAGATTTGATTCTCAAGCACACACTGATAGCCAACTCCTTACTTATTCTTTCTAAAGGACTGCCTCAGATAATAGTAGACTTTGGACTAAAAAGTCCTGTCAATGATTTTGTCTGCAAACTTATCTTTTATATTGAATGCGTGGGCAGGGGCATGTCTGTTGGAACCATCTGTCTGTTGAGTATCTTCCAGACGATCACAATCAGCCCCATAAACTCCTGCTGGAAAGACCTTAAAATAAAAACCTCAAAGTATATCAGCTTTTCCATTTGCCTATGCTGGCTTCTGCACGTGGTGGTAAATTTCATTTTGCCTCTCTATACATTGTTTGTGTCTGGAAAATGGTATAGCAAAAACATCACAAAGGAGATAAAAATGAGATTCTGTTCCTTTGTAGGTCATGGGACAATCCCAGGCTCAATCTATATAGCGTTGGTAGCATTCCCTGAAGTTGCTTGTTCTGTGCTCATGATCTGTGCAAGTGGCTCCACGATTTTTACTCTGTACCAACACAAGCAGAGGGTCCGACACATTCATAGGACAAATGTCTTGTCCAGAACCCCTGAGTCCAGAGCCACCAAAAGCATCCTCCTTCTGGTGGGCACTTTTGTATCTTTTTACAGCCTGTCCTCCCTCTTTAACATTTCTATCACCCTTTTTCCTCAGGAATATTGGTTGCTGATGAGCATTTCTGATATAATTTCTCTGTGTTTTCCAACTATGAGCCCCTTTCTGCTAATGAGCCAGGACTCTTTTACATCCAGGCCCTGCTTTGTTTGGATAAGGAACGCCCTCCCCACTTATAAGAAATGA

>Cavia_porcellus_intact_V1R641

ATGAGAATGAATCCTGGTGATTTCATCTTGAGGCTACTTTTCCTGGTTCAGACTGGGATTGGAGTGGTGGGAAATTCCTTTCTTCTCTCAATCTATGCCCCTACATCCTGTACTGGCCGTGTACCAAGGCCCACACACCTGATTCTCACTCACATGGCTGTGGCCAACTTCTTGGTTCTGCTCTTCAAGGGGATTCCACACATGATGCTAATCTGGGGAATAACACCCACTCTGGGAAATGCAGGATGCAAACTTGTCTATTATATCCACAGAGTGGCCCGGGGCCTTTCTCTCTGCACCACTTGCCTCCTGAGCAACTTTCAGGCCATCACCATCAGCCCCAGACCAGGAGGGATGATAGGCCTTAACCACCAAGCTTGGAAGAGCATCAGTTTCTCCTGTGTTCTGTGCTGGATCTTCAACTTGTTGATGAATATCTTTATTCCTATAAATATGGAGGGCCTTCAACATGGCCACAACTCTACCAAGCTACTTGACTATGGTCTATGTTCTTCCAGAGATTCTGAAACAAGTACTATAAGATACACATTTCTAATGACACTGGCAGATGTTGTGTTCCTGGCACTCATGGCTGGGGCCAGTGCCTACATGGTGCTTCTCCTACATACACATCAGCAGACAGTGAAGCACATTTACAGCACCAACAAATCCCATAGATTCTCCCCTGAGACAAAAGCCACTCAAACTATCCTGATTCTAGCAAGCACCTTCATTGTGTTTTATTTAACCAATTCTTTTCTTACCATTTATAGTACTCTCATTTTAAAATCTCGCCTCTGGCTGCAGCATACAAATGCATTTCTGGCAGCCTGTTATTCCACCATCAGCCCCTTGATACTGATGCTGCGAGATCCTCGAACATCAAACTGCTGCTCTTAA

>Cavia_porcellus_intact_V1R642

ATGTTTCCCAGTGCAGTATATTTTGGCTTTTTCCTCATATCACAGGTTTGTTTTGGTGTCCTTTCAAACTCCTCAGTGTTTATGGCATTGATGCATACCTTATTATCTCAACTTCATCTTATGAGACCTATAGACTTCATTATGGTTCACCTGACAGTGGTGAATAATTTGACTATCATAGTCACACTTATACCATATATCATGATATCCTTTGGAATAAGACACTTTTTGGATGATACTGGATGCAAAGTAATTTTGTATGCATATAGAGTTACCCGTGGTGTTTCCATCTGTACTACCTCACTTATGAGTACATTTCAAGCCATCACTATCAGCTCTGTTAATTCTAAGTGGGCACGGTTGAAGCCTAAACTCTCTGCAATGATTATGCCTTCTTTGCTTATCTTCTGGATCAACAACTTGCTTATTTATATCAATGTTATTCCGCATGTAAGAGCAATTGGAAATTTTTCTCTTGTTGGTCCTGGGTATTTTCACGCATATTGTCAAACTGGACAGATGAAAACCATGGATTCATGGTTATATATAATTGTCATATTAGCCCAAGATCTTGTGTTCTTGACCCTCATGCTCTGCACCAGCCTCTACATGGTGAGTCTCCTATACCGACACCACAGGATAGCCAAATATGTCCATAGATGCTCCTCCCAGACACCTGCAGAAATCAGAGCAACACAAAACATTGTTTTGGTGGTGTGTTGCTTTGCTTTTTTCTATTGCTCAAATAACTTTTTCACTTTTTACTCTTTGTCTATACATGAGAAAATTCCTGCTTTAGAGGCAATTAGTGCAGTGTTATCATCATGTTTTCCAATCATCAGTCCTTTCTTTTTGATGAGAAATAATAAATTATTTTCTAAATATTTCTGTTTTACAGTGATGAGGTTTATCTGCCCTCACACTCTCTTTAGTGGCTAA

>Cavia_porcellus_intact_V1R643

ATGAAAACAAGGAGAAAATTTTCCATTGTTTCTAATATAGAAAACACAGTTTTCTTTGAAGTCACCATTGGAATCATAGCCAACACTGTCCTGCTACTGTTCCACATCCTCATATTTCTCCTCAAGCAAAGGCCCACACCTCTTGACCTGACTATTGGTCAGTTGGCTCTAATTCACCTGGTAATGCTGGTAACTGTGGGCCTTATAGCTACAGACACTTTTGGGGTTCGTGACTGGGGGAATGACTTCCCATGTAAATCAGTTATCTATATATATAAGTTAATGAGGGGCCTTTCTATCTGTACTACTTGTGTAATGAGCATCCTACAGGCTGTCACTCTCAGTCCCAGAAACTCTTGCTTGGCAAAATTCAAACATAAATCTATACATCATTACCCATGTTGCCTTATCTTCATATGGGTCTTCAATATGCTCCTGAATGGTCGTTTCTTAGTCTCCATTGGTGCCACCCCCAATGTATCCTCACACAGTCTTCTGTTTGTCTCTGAATCCTGCTCTCAGTGGCCCATTAATTATTTGTTCAGGTACATATTTTTCTCATTGGTGAACATTCAGGATATCTCCTTTATAGGGCTGATGGCTCTCTCAAGTGGATACATGGTGAGCCTGTTGTGCAGGCATAAGAGGCAGTTCCAGCATCTTCACAGCATCAGCTTTTCTCCAAAAGTATCCCCAGAAGGAAGGGCCACCCGAACCATTCTGCTGCTTATGGAGTTCTTTATGATCATGTACTTTTTGGACTGTGTGTCCTTCTTTTCCTCTGGAATACTGTGGAAAAATGATCCAATTAAGCATTTTATTCAGATGCTTGTGGGCAATGGCTATGCCGCAATCTGTCCTTTTGTGCTAATAAGCACCGAAAAACGAATCATCAGGTGGTTAAAATTCAGATGCTAA

>Cavia_porcellus_intact_V1R644

ATGATGTCAAAGAAAGAGAGAACATTTTCCAGGTTTCTTGATGTAAGAAGCACAGTTTTCTTTGAAATCACCATCGGGATCACTGCCAACACTGTCCTACTTCTGTTCCACATCCTCACATTTCTCCTCAAGCAAAGGCCAAAGCCCCTTGACCTGACTATCACTCAGTTGTCCCTGATCCACCTGGTGATGCTGGTAACCATGGGATTCATAGCTACAGACACTTTTGAGTCTCAGCGTTGGGAGAATGTCCTCACATGCAAATTGGTTATCTACATGCAAAGATTGATGAGGGCCCTCTCCATCTGTACCACCTGTCTGCTAAGTGTCCTCCAGGCTATCACCCTAAGCCCCAGAAATTCTTGTTTGGCAAAATTCAAACAAAAATTGTCACATCATTATCCATGTTGCCTTGGATTTTTATGGGTCTTCAATATGCTCCTGAATGGTCGTTTCTTAGTCTCCATTGGTGCCACCCCCAATGTGACCTCACAGAGTCTTGTGTTTGTCACTGAATCCTGCTCTCAGTGGCCTATTAATTACTTATTCAGGTACATATTTTTCTCATTGGGCAATGTTCAAGATATCTCCTTTATAGGACTGATGACTCTATCCAGTGGATACATGGTGAGTCTTTTGTGGAGGCATAAGAGGCAGTTCCAACATCTTCACAGCATCAGCCTTTCTCCAAAGGCATCCCCAGAAGAAAAGGCCACTCGAACCATTCTGCTGCTCATGGGGTTTTTTATGCTTATGTACATTTTGGACTGTGTTACTTTCTCCTCACGTTCAATATTGTGGAACAATGACCCAATCCATCTTTGTGTCCACATGTTTGTGGGCAATGCCTATGCAACAATCTGTCCTTTTGTGCTACTTAGCACTGAAAAATGA

>Cavia_porcellus_intact_V1R645

ATGATGGTGAAGAAAAAGAGAAAACTTTTTAGGTATACTATCATAAGAAGCATAGTGTTCTCTGAAGTCACCATTGGAATTGCAGCCAATACCATCTTGCTTCTGTTTCACATCTCCATATTTCTCTTCAAGCACAGGCCCAAGCCCCTTGATCTCACTATTGCTCAGTTGGCCCTGATCCACCTGGTGATGCTGACAACTGTGAGCTTCATAGCTACAGATACTTTTGGGTTTCAGAGTTGGGATGGCCTCACATGTAAATTGGTTATCTATGTGTACAGGTTGATGAGGGCCCTCTCTATCTGTACCACCTGCCTGCTGAGTGTCCTCCAGGCCATCATCCTATGCCCCAGAAGCTCTTGCCTGGCAAAATTCAAACAAAAATCCTCACATCATTACCCAAGTTGCCTTGGATTTTTATGGGTTTTCAATATGCTTCTGAACGGTCATTTCTTAGTCTCCATTGGTGCCACATCCAATGTGATGTCACACAATCTTGTGTTTGTCACTGAATCCTGTTCTCTGTGGTCTATTAATTACTTTTTCAAGTACATATTTCTTTCATTGGTGAACATTCAAGATGTCTCCTTTATAGGGCTGATGACTCTTTCAAGTGGATACATGGTGAGTCTCTTGTGCAGGCATAAGAGGCAATTTCAGCATCTTCACAGTACCAGCCTTTGCCCAAATGCATCCCCAGAAGAAAGGGCCACCCGGACCATTTTGCTGCTCATGGTGTTTTTTATGCTCATGTACATTTTGGACTGTGTTTCTTTCTCCTCGCTTGCAATATTGTGGAACAATGACCCACTTCATCTTTGTGTTCTGATGGTTGTGAGCAATGGATATGCCACAATCTGTCCTTTTGTGCTAATGACCTCTGAAAAAAGGATAGCTGAGTCCTTAAAATCCAGGTGGAAAAGATAA

>Cavia_porcellus_intact_V1R646

ATGGTGAAGAAAAAGGGAAAAATTTCCAGATTTTTTGATATAAGAAGCACAGTTTTCTTTGAAATCACCATTGGGATCATAGCCAACACTGTCCTCCTTCTGTTCCACATTCTCCAGTTTCTATTCAAACACAAACCCAAGCCCCTGGACCTAACTATTGCTCAGTTGGCCCTAATCCACTTGGTGATGCTGGTAACCATGGGTTTCATAGCTACAGACACTTTTGGGTTTCAGGGTTGGGAAGATGGCCTCACATGTAAAGTAATTATTTACATGCAAAAGTTGATGAGAGCCCTCTCGATCTGTACCACGTGCCTGTTGAGCATCCTCCAGGCCATCACCCTAAGCCCCAGAAACTCTACTTTGGCAAAATTCAAACATAAAGCCACATATCGCTATCCATGTTGCCTTGTGTGTTTATGGGTCTTCAATATGCTCCTGAATGGTCGTTTCTTAGTCTCTATTGGTGCCACTCCCAATGTGACCTCACATAGTCTCATATTTGTCACTGAATCCTGCTCTCAGTGGCCTATTAGTTACTTGTTCAGGTACATATTTTTCTCACTGGCAAATATTCAGGATATTTCCTTTATAGGACTGATGGCTTTCTCAAGTGGATACATGGTGAGCCTGTTAAGCAGGCATAAGAGGCAGATCCAGCATCTTCACAGCACCAGCTTTTCTTCAAAGAGCTCCCCTGAAGGAAGGGCTACCCAGACCATTCTGTTGCTCATGGGGTTCTTTATGCTCATGTACTTCTTGGACTGTGTTACTTTCTCCTCCCGTGCAATATTGTGGAATAATGACCCAATTAGTCTCTGTGTACACATGCTTTTGGGCAATGGCTATGCCACAGTCTGTCCTTTTGTCCTAATGAGAACTGAAAAACGAATGATTGGGTGCTTAACATTCTGGTGGAAAAGATAA

>Cavia_porcellus_intact_V1R647

ATGATGATGAAGAAAAAGAAAACATTTTCCAAGTTTTTTGATGTGATAATCACCATTTTCTTTGAATTCACCATTGGAATCATAGCTAACACTGTCCTGCTTCTGTTCCACATCCTCACATTTCTTTGCAAGCACAGGCCTAAGCCCCTGGACCTGACTATTGCTCAGTTGGCCCTGATCCACCTGGTGATGCTGGTAACTGTGTGCTTCATAGCTACAGACACTTTTGGATTTGGGTACTGGTGGAACAACCTCATGTGTAAATCTGTTATCTATGTATATAGGTTGATGAGGGCCCTGTCTATCTGTACCTCTTGCCTGCTGAGCATCCTTCAGGCCATCACTCTAAGCCCAAGAAATTCTTGTTTGGCAAAATTCAAACATATATCTACACATCACTATCCGTGTTGCCTTGTCTTCTTATGGGTCTTCAATATGCTCCTGAATATTCGTTTCTTACTTTCCATTGGTGCCACCCCCAATGTGACCTCACACAGTCTCATGTTTGTCTCTGAATCGTGCTCTCTGTGGCCTGTTAATTCCTTGTTCAGGTACGTATTTTTATCATTGTTGAACATTCAGGATATCTCCTTTATAGGGCTGATGGCTCTGTCAAGTGGATATATGGTGAGTCTTTTGTGCAGGCATAAGAGGCAAATCCAGCATCTTCACAGCACCAGCCATTCTCCAAAGTCATCCCCAGAAGGAAGGGCCACCCAGACCATTCTGCTGCTCATGGGGTTCTTTATGTTCATGTACTTATTGGACTGGGTTGTTTTCTCTTCCAGTGCAATATTGTGGAGTAATGACCCAGTCAGTCTTTGTGTCCAGATGCTTGTGGGCAATGGTTATGCCACAGTCTGTCCTTTTATTCTAATGAGCACTGAGAAACGCATGGCTGAATGTTTAATAGTCAGGTGGGCAAGAAAATGA

>Cavia_porcellus_intact_V1R648

ATGATGGTGAAGAAAAAGAGAACATTTTTCAGGTTTTTTGATGTAAGAATCACCATTTTCTTTGAAATCACCATTGGAATCATAGCCAACACTGTCCTCCTTCTGTTCCACATCCTCACATTTCTTCTCCAGAACAGGCCTAAACCCCTTGACCTGACTATTGCTCAGTTGGCGCTGATCCACCTGGTGATGCTGATGACCATGAACTTCATAACTACAGACACTTTAGGATTTCAGGGTTGGGAAGATGGCCTCACATGTAAAGTGATTATCTATGTGCACAGGTTGATGAGAGCCCTCTCCATCTGTACCACCTGCCTGCTGAGTGTCCTCCAGGCCATCACTCTAAGCCCTAGAAATTGTTGTTTGGCAAAATTCAAACATATATCCACACATCACTATCCATGTTGCCTTGTGGGTTTATGGGTCTTCAATATGCTCCTGAATGGTCGTTTCTTAGTCTCCATTGGTGTCACCCCCAATGTGACCTCACACAGTCTCATATTTGTCACTGAATCCTGTTCTCTGTGGCCTATTAATTACTTATTCAGATACATATTTTTATCATTGGTTAACATTCTGGATATCTCCTTTATAGGACTGATGGCTCTTTCAAGTGGATATATGGTGAGCCTGTTGTGCAGGCATAAAAGACAGTTCCAACATCTTCACAGCACTAGACTTTCCCCTAAGACATCCCCAGAAGAAAGAGCCACCCAGACCATTCTGTTGCTCACGGTTTTCTTTATGCTTATGTATTTCTTGGACTGTGCTACTTTCTTCTCCCGTTCAATATTGTGGAACCATAACCCAGTTAATCTTTGTGTTCACATGCTTGTGGGCAATGGCTATGCAACTGTCTGTCCTTTTGTTCTAATGAGCACTGAAAAACGAATGATTGAGTGTTTACCATTCAGGTGTAAAAGACAATGA

>Cavia_porcellus_intact_V1R649

ATGCTTTGCATTGACCTCTTGCAGAGAATAATCTTCTTTTCTCTTACTGGACTTGGGATTTGGGGGAACACTTTTCTATTTGTGAGATATGTATATACTTTTGTAATGAGTCCTGAGAAAAAACCCATAGATTTTATCATCATTCACCTGGTTTTTTCAAATGCAATCATTATTTGTAGCACAGGGATTAGAGATATAGCCCCAGTTTTCCGTTTCAGAAACTTCCTAGGTAGTGCTGGCTGTAAAACTGTGGTTTACCTGGGGAGGGTGGCCCGGGGCCTGTCCATCTGCAGCACCTGTCTCCTCAGCGTGGTCCAGGCCATGACCATCAGTCCCAGCACCACCCTGTGGGGAAAGCTCAAGCCATGGACTGCATGGCAAGTTCTTCCCTATCTTCTCCTCTGCTGGGTCATTAATCTTCCCATAAGCTCCAACTTGCTGTTCTATGTCACAGCACTTGGTGGGGTGAACAGATCTACAAGCCGAGCATATGGTGGGCACTGTTTTTTGCTGGCATCCAGACACACAGTCCGGTGGCTTTTCCTCTGTCTCATGGCTCTGCGGGATGTCCTCTTCCAGGGTGTCATGGGCTGGAGCAGCGGGCACATGGCTTTCCGCCTGTATGAGCATCACAAGCGTGTGCTTTATCTGCACAGCTCCAGGTTGGTGGTGAGTTCCAGTCCAGAAATCAGAGCTACTGTAAATATTCTTGTCCTCATGACCTGTTTCCTTTTCTTTTTTTGGACAGATTTCATGTTCTCCTTCTACCTAGGGTTCATGGTGGCCCATGGAGTTGCACTCCTCTATGTTAAATTCTTTCTAGAACTTGGTTATGCGGTCCTCAGTCCCTTTGTGCTGAGGAGCAGGGATGGGTGTGGGGCGAAGGCCCAGAATGCTCACTGA

>Cavia_porcellus_intact_V1R650

ATGGTTTTGAACCTTATCAAGAGAATCATCTTCCTCTCTCCAACTGTGCTTGGGATTCTGGGGAATGTCACAGTGTTTCTGAATTACATTCTCCTACTTGGAGGAACCGAGATGAAATCTGTACACCTTATTCTCATCCACCTGGCTTTTACAAATATCATCATGCTTTTTGCAAAACGGGTTCCTAAGACAATAGTAGCTCTTGAGCAGAGAAACTTCCTCCATGACACAGGCTGCAAGATTGTTGTTTACCTGGAGCGGGTGGCCCGGGGCCTCTCCATCTGCACCAGCAGCCTCCTCACAGTGACACAAGCCGGCACCATGAGTCCCGCAGATTCGGTGTGGAGGAGACTCAGGCTAAAGTCACCAAGGCACATTCTCTCCATATTCATCTTGTTTTGGATTCTCAATTCCTTTATAAGCATGAACTTACTGCTTTCTGTCATAAACATCAACAGTGTGAACATATCACAAATTACAAAAGGTGACAAGTATTGCTATTTTCTACCTGAAGGCTGGATAATAAGATGGACATTTCTCACTCTGATGGTTCTACGGGATGCTGTGTTTCTGGTCATCATGGGTGGGTCCAGTGGCTACCTGGTGTTTCTTCTCCGCAGGCACCACCAGCGTGTTCTCCACCTGCAGAGCTCCAAGTTCCTCTACAAAACTCCCCCTGAGATCAGAGCTGCTCAAAGCGTTCTCTTTCTGATGCTCTGTTTTCTTGTCTTTTATTGGGCAGATTGTGCTTTTTCCATGTTGTTTAGCTCCTCTCTCAATATAGACTCCATGGTGTTACATGTCCAAGAATATGTGACCCTTGGTTATGCCATGCTGAGCCCATTTGTGCTGATTCACAGAGATGGACAGCTGGGCGCATGTTTTCATCATCTGAAAGACAGAACATCACACAGAAAGTGTCTGATTGATCTTCTCTGCTAA

>Cavia_porcellus_intact_V1R651

ATGCGATCGAATATTGTCAAGGGAGCAATCTCTGTGGCGCTAACTGGAGTTGGCACTATGGGGAATATCTTTGTTTTTATGAAGTACATGCTCATATTTGCAGGCAGTGAGATGAAACCTATACATCTCATTCTCATCCATTTGGCTGTTACAAATACCATGACTCTGCTTTCCAAGGGAGCGCCAGATACCTTAGCAGTTTTTGGTGTGAGGAACTTCCTAGATGACACAGGGTGTAAGATTGTTGTTTATACGCAGCGGGTGGCCCGGGGCCTCTCCATCTGCACCACTGGTCTCCTCACAGTGGTCCAGGCCATCACCATGAGTCCCAGAACCTCGGGGTGGAGGAAGCTCCAGCCCAGGTCTGCATGGCACCTCCTTCCCTTGTGTCTTTTCTTTTGGATTCTCAATTCCTCAGTAGGCATGAACTTACTCCTTTATATCACAAGTATAAACAAGAATACATCAAAAGGGAGCATGAACCGTAGTCCCTGTTTTTTTCAACCAGAAAACCCAAAAGTGAATTGGATATTTCTCATGGCCATGGTAGGACGAGACATCGTGTTTTTGGCTGCCATGGGCACAGCCAGTGGTCACATCGTCTTTCTTCTCCGCAAGCACCACCACCGTGCTCTCTGCCTACAGATCTCCAAGTCACTCCACAAAGCTGCTCCCGAGGTCAGGGCCTCTAGAAGTGTGCTCCTTCTGATGCTCTGCTTTGTTTTATTCTATTGGACAGATTGTGTCTTTTCTGTATTTACTAATTCTTTCCTTGAGAATAATTTCATTTTATTAGATGCTTGGAATTTTGTGACCCTCGGTTATGCTGTTGTCAGCCCATTTGTACTGATTCACAGAGATGTACACCTGACCAAGTTCAGGCGAGCTCTGTGGAACACAGCAAGCACTGAAAAGCTGCGTATTCCTTGA

>Cavia_porcellus_intact_V1R652

ATGCCATCTAACACTGTCAGGGGAGCAATCTCTGCCTCGCTAACTGGATTTGGCACTATGGGGAACATCTTTGTTTTTGTGAAGTGTGTGCTCATATTTCGAGGCAGTGAGATGAAACCTATACATCTCATTCTCATCCATTTGGCTGTTACAAATACCATGACTCTGCTTTCCAAGGGAGCTCAAGAGACCTTAACTGCTTTGGGTGTGCGAAACGTCCTAGATGACACAGGCTGTAAGACTGTTGTTTACTTGTCAAGGATGGCCAGGGGCCTCTCCATCTGCACCACTGGTCTCCTCACAGTGGTCCAGGCCATCACCATGAGTCGCAGACACTCAGGGTGGAGGAAGCTCCAGCCCAGGTCTTCATGGCACCTCCTTCCCATGTGTCTTTTCTTTTGGATTCTCAACTCCTCATTAAGCCTGAACTTACTCCTTTACATCACAAGTACAAGCAAAAATTCATCAAAAGGGAATATGAACCGTAGTCCCTGTCTTTTACAACCAGAAAACCTAAAAGTGAAATGGATATTTGTTGCAGCCATGGTAGTAAGAGATGTCATGTTTTTGGCCACCATGGGCACAGCCAGTGTGCACATCATCTTTCTTCTCCACAAGCACCACCAGCGTGCTATCTGCCTACAGATCTCCAAGTCACTCCACAAAGCTGCCCCTGAAGTCAGAGCCTCTAAAAGTGTGCTCCTTCTGATGCTCTGCTTTGTTTTCTTCTATTGGACAGACTTTGTTTTTTCTCTGTTTATTAATTCCTTCCCTGAGAATTCCATTTTAGTAGATATTTGGAATTTTATGACCCTTGGTTATGCGATTGTCAGCCCATTAGTTCTGATTCACAGAGATGGACACCTGACCGAGTGCAGGCTAGCTCTGTGGAACAGAGCGGGCACTGAAAAGCTGTGTATTCTTTGGCCCTTTCCTTAG

>Cavia_porcellus_intact_V1R653

ATGGTTTTGAACCTTGTCAAGAGAATCATCTTCCTCTCTCCAACTGTGCTTGGGATTCTGGGGAATGTCACAGTGTTTCTGAATTACGTTCTCCTACTTGGAGGAACTGAGATGAAATCTGTACACCTTATTCTCATCCACCTGGCTTTTACAAATATCATTTTTGCAAAACGGGTTCCTAAGACAATAGTAGCTCTTGAGCAGAGAAACTTCCTCCATGACACAGGCTGCAAGATTGTTGTTTACCTGGAGCGGGTGGCCCGGGGCCTCTCCCTCTGCACCAGCAGCCTCCTCACAGTGACACAAGCCGGCACCATGAGTCCCGCAGATTCAGTGTGGAGGAGACTCAAGCTAAAGTCACCAAGGCACATTCTCTCCATATTCATCTTGTTTTGGATTCTCAATTCCTTTATAAGCATGAATTTACTGCTTTCTGTCATAAACATCAACAGTGTGAACATATCACAAATTACAAAAGGTGACAAGTATTGCTATTTTCTACGTGAAGGATGGATAATAAGATGGACATTTCTCACTCTGATGGTTCTACGGGATGCTGTGTTTCTGGGCATCATGGGTGGGTCCAGTGGCTACCTGGTGTTTCTTCTCCGCAGGCACCACCAGCGTGTTCTCCACCTGCAGAGCTCCAAGTTCCTCTACAAAACTCCCCCTGAGATCAGAGCTGCTCAAAGCGTTCTCTTTCTGATGCTCTGTTTTCTTGTCTTTTATTGGGCAGATTGTGCTTTTTCCATGTTGTTTAGCTCCTCTCTCAATATAGACTCCATGGTGTTACATGTCCAAGAATATGTGACCCTTGGTTATGCCATGCTGAGCCCATTTGTGCTGATTCACAGAGATGGACAGCTGGGCGCATGTTTTCATCATCTGAAAGACAGAACATCACACAGAAAGTGTCTGATTGATCTTCTCTGCTAA

>Cavia_porcellus_intact_V1R654

ATGCTTTGCATTGACCTCTTGCAGGGAATAATCTTCTTTTCTCTTACTGGACTTGGGATTTGGGGGAACACTTTTCTATTTGTGAGATATGTATATACTTTTGTCATGAGTTCTGAGAAAAAAGTCATAGATTTTATCATCATTCACCTGGTTTTTTCAAATGCAATCATTATATATAGTAACGGGATTAGAGATATAGCCCCAGTTTTCCGTTTCAGAAACTTCCTAGGTAGTGCTGGCTGTAAAACTGTGGTTTACCTGGGGAGGGTGGCCCGGGGCCTGTCCATCTGCACCACCTGTCTCCTCAGCGTGGTCCAGGCCATGACCATCAGTCCCAGCACCACCCTGTGGGGAAAGCTCAAGCCTCGGACTGCATGGCAAGTTCTTCCCTATCTTCTCCTCTGCTGGGTCATTAATCTTCCCATAAGCTCCAACTTGCTGTTCTATGTCACAGCAGTTGGTGGGGTGAACAGATCTACAATCCGAGCATATGGTGGGCACTGTTTTTTGCTGGCATCCAGACACACAGTCCGGTGGCTTTTCCTCTGTCTCATGGCTCTGCGGGATGTCCTCTTCCAGGGTGTCATGGGCTGGAGCAGCGGGCACATGGCTTTCCGCCTGTATGAGCATCACAAGCGTGTGCTTTATCTGCACAGCTCCAGGTTGGTGGTGAGTTCCAGCCCAGAAATCAGAGCTACTGTAAATATTCTTGTCCTCATGACCTGTTTCCTTTTCTTTTTTTGGACAGATTTCATGTTCTCCTTCTACCTAGGGTTCATGGTGGCCCATGGAGTTGCACTCCTCTATGTTAAATTCTTTCTAGAACTTGGTTATGCGGTCCTCAGTCCCTTTGTGCTGAGGAGCAGGGATGGGTGTGGGGCGAAGGCCCAGAATGCTCACTGA

>Cavia_porcellus_intact_V1R655

ATGGCTTTAAACCTTGTCAAGGGAATCATCTTCCTCTCTCCAACTGTGCTTGGCATTCTGGGGAATGTTACTGTGTTTGTGAATTATGTTCTCCTCCTTGGAGGCACCGAGATGAAATCTGTACAACTTATTCTCATTCACTTGGCTTTTACAAATATCATCATACTTTTTGCAAAAGGGATTCCTAAGACAATATCAGCTTTTGGGCAGGGACTCTTCCTCCATGACACAGGCTGCAAGATTGTTGTTTACCTGGAGCGGGTGGCCCGGGGCCTCTCCATCTGCACCAGCAGCCTCCTCACAGTGACACAAGCCGTCACCATGAGTCCCACAGACTCCATGTGGAGGAGGCTCAAGCTGAAGTCACCAAGGCACATGCTTTCCATATTCATCTTGTTTTGGATTCTCAATTCCTTAATAAGCATGAACTTACCACTTTCTATCATAAGTATCAACAGTGTGAACATATCACAAATTACTAAAGGTGACAAGTATTGCTATTTTCTACCTGAAGGATGGATAATAAGATGGACATTTCTCCTTCTGATGGTTCTACGGGATGCTGTGTTTCTGGGCATCATGGGTGGGTCCAGTGGCTACCTGGTGTTTCTTCTCTGCAGGCATCACCAGCGTGTTCTCCACCTGCAGAGCTCCAAGTTCCTCTACAAAACTCCCCCTGAGATCAGAGCTGCTCAAAGCGTTCTCTTTCTGATGCTCTGTTTTCTTGTCTTTTATTGGGCAGATTGTGCTTTTTCCATGTCGTTTAGCTCCTCTCTCAATATTGATTCTGTGGTGTTAAATATTCAAGAATATGTAAGTCTTGGTTATGCCATGCTGAGCCCATTCGTGCTGATTCACAGAGATGAACAGCTGGGTACATGTTGTCACAGTCAAGAGGACAGCAAAACCCACAGGAACTGTTTTACTGATTTTTTCTGTAACTAA

>Cavia_porcellus_intact_V1R656

ATGCCATCTAACACTGTCAGGGGAGCAATCTCTGCCTCGCTAACTGGACTTGGCACTATGGGGAACATCTTTGTTTTTGTGAAGTATGTGCTCATATTTGGAGGCAGTGAGATGAAACCTATACATCTCATTCTCATCCATTTGGCTGTTACAAATACCATGACTCTGCTTTCCAAGGGAGCGCCAGAGACCTTAACAGCTTTGGATGTGCGAAACTTCCTTGATGACACAGGCTGTAAGACTGTTGTTTACTTGTCAAGGATGGCCCGGGGCCTCTCCATCTGCACCACTGGTCTCCTCACAGTGGTCCAGGCCATCACCATGAGTCGCAGACACTCAGGGTGGAGGAAGCTCCAGCCCAGGTCTTCATGGCACCTCCTTCCCTTGTGTCTTTTCTTTTGGATTCTCAATTCCTCCATAAGCCTGAACTTACTCCTTTACATCACAAGTACAAGCAAAAATTCATCAAAAGGGAATATGAACCGTAGTCCCTGTCTTTTACAACCAGAAAACCTAAAAGTGAAATGGACATTTGTTGCAGCCATGGTAGTAAGAGATGTCATGTTTTTGGCCACCATGGGCACAGCCAGTGTGCACATCATCTTTCTTCTCCACAAGCACCACCAGCGTGCTCTCTGCCTACAGATCTCCAAGTTACTCCACAAAGCTGCCCCTGAAGTCAGAGCCTCTAAAAGTGTGCTCCTTCTGATGCTCTGCTTTGTTTTCTTCTATTGGACAGATTTTGTTTTTTGTCTCTTTATTAATTCCTTCCATGATAATTCCATTTTAGAAGATATTTGGAATTTTATGACCCTTGGTTATGCGATTGTCAGCCCATTAGTTCTGATTCACAGAGATGGACACCTGACCGAGTGCAGGCGAGCTCTGTGGAACACAGGGGGCACTGAAAAGCTGTGTATTCCTTGGCCCTTTCCTTAG

>Cavia_porcellus_intact_V1R657

ATGCCATCTAACATTGTCAAGGGAGCAATCTCTGTGTCGCTAACTGGAGTTGGCACTATGGGTAACATCTTTGTTTTTGTGAAGTACATGCTCGTATTTGCAGGAAGTGAGATGAAACCTATACATTTCATTCTCGTCCATTTGGCTGTTACAAATACCATGACTCTGCTTTCCAAGGGAGTGCCAGGTACCTTAGCAGTTTTCGGTGTGAGGAACTTCCTAGATGACACAGGGTGTAAGATTGTTGTTTACATGCAGCGGGTGGCCCGGGGCCTCTCCATCTGCACCACTGGTCTCCTCACAATGGTCCAGGCCATCACCATGAGTCCCAGAACCTCGGGGTGGAGGAAACTCTGGCCTATGTCTGCGTGGCACCTCCTTCCCTTGTGTCTTTTCTTTTGGATTCTCAATTCCTCAGTAGGCATGAACTTACTCCTTTATATCACAAGTATAAACAAGAACACATCAAAAGGGAGCATGAACCGTAGTCCCTGTTTTTTTCAACCAGAAAACTCAAAAGTGAAATGGATATTTCTCATGGCCATGGTAGGACGAGACATCGTGTTTTTGGCTGCCATGGGCACAGCCAGTGGTCACATCATCTTTCTTCTCCGCAAGCACCACCAGCGTGCTCTCTGCCTACAGATCTCCAAGTCACTCCACAAAGCTGCCCCTGAGATCAGAGCCTCTAAAAGTGTGATCCTTCTGATGCTCTGCTTTGTTTTGTTCTATTGGACAGATTGTGTCTTTTCTGTATTTAATCATTCTTTCCTTGAGAATAATTTCATTTTATTAGATGTTTGGAATTTTGTGACCCTCGGTTATGCTATTGTCAGCCCATTTGTGCTGATTCACAGAGATGTACACCTGACCAAGTTCAGGCGAGCTCTGTGGAACACAGCAAGCACTGAAAAGCTGCGTGTTCCTTCATCCTTTCCTTGGGTAAGTTCGAAGTAG

>Cavia_porcellus_intact_V1R658

ATGGCTTTAAACCTTGTCAAGGGAATCATCTTCCTCTCTCCAACTGTGCTTGGCATTCTGGGGAATGTTACTGTGTTTGTGAATTATGTTCTCCTCCTTGGAGGCACCGAGATGAAATCTGTACAACTTATTCTCATTCACTTGGCTTTTACAAATATCATCATACTTTTTGCAAAAGGGATTCCTAAGACAGTATCAGCTTTTGGGCAGGGACTCTTCCTCCATGACACAGGCTGCAAGATTATTGTTTACCTGGAGCGGGTGGCCCGGGGCCTCTCCATCTGCACCAGCAGCCTCCTCACAGTGACACAAGCCGGCACCATGAGTCCCACAGACTCCATGTGGAGGAGGCTCAAGCTGAAGTCACCAAGGCACATCCTTTCCATATTCATCTGGTTTTGGATTCTCAATTCCTTAATAAGCATGAACTTACCGCTTTCTATCATAAGCATCAACAGTGTGAACATATCACAAATTACTAAAGATGACAAGTATTGCTATTTTGTACCTGAAGGATGGATAATAAGATGGACATTTCTCATGCTGATGGTTCTACGGGATGCTGTGTTTCTGGGCATCATGGGTGGGTCCAGTGGCTACCTGGTATTTCTTCTCCGCAGGCACCACCAGCGTGTTCTCCACCTGCAGAGCTCCAAGTTCCTCTACAAAACTCCCCCTGAGATCAGAGCTGCTCAAAGCGTTCTCTTTCTGATGCTCTGTTTTCTTGTCTTTTATTGGGCAGATTGTGCTTTTTCCATGTTGTTTAGCTCCTCTCTCAATATTAATTCTGTGATGTTAAATATTCAAGAATATGTAACCCTTGGTTATGCCATGCTGAGCCCATTTGTGCTGATTCACAGAGATGAACAGCTGGGTACATGTTGTCACAGTCAAGAGGACAGCAAAACACACAAGAACTGTTTTACTGATTTTTTCTGTAACTAA

>Cavia_porcellus_intact_V1R659

ATGGTTTTGAACCTTGTCAAGGGAATCCTCTTCCTCTCTCCAACTGTGGTTGGCATTCTGGGGAATGTCACTGTGTTTGTGAATAATGTTCTCCTTGCAGGCACGGAGATGAAATCTGTATATCTTATTCTCATCCACTTGGCTTTTACAAATATCATCATGCTTTTTGCAAAAGGGATTCCTAAGACAATAGCAGCTCTTGGGCAGAGAAACTTCCTCCATGACAAAGGCTGCAAGATTCTTGTTTACCTGCAGCGGGTGGCCCGGGGCCTCCCTATCTGCAACAGCAGCCTCCTCACGATGACCCAAGCTGTCACCATGAGTCCCACAGACTCGGTGTGGAGGAGGCTCAAGCTAAAGTCACCAAGGGACATCCTTCTCATATTTGTCTTGTTTTGGATTCTCAATTCCTTAATAAGCATGAACTTATCACTTTCTGTCATAAACATCAACAGTGTGAACATATCACAAATTGCTAAAGGTGACAAGTATTGCTATTTTCTACCTGAAAACTGGAAAATAAGATGGGCATTTCTCACTCTGATGGTTCTACGGGATGCTGTGTTTCAGGGCATCATGGGTGGGTCCAGTGGCTACCTGGTGTTTCTTCTCCGCAGGCACCACCAGTGTGTTCTCCACCTGCAGAGCTCCAAGTTCCTCTACAAAACTCCCCCTGAGATCAGAGCTGCTCAAAGCGTTCTCCTTCTGATGCTCTGTTTTCTTGTCTTTTATTGGGCAGATTGTGCTTTTTCTATTTTGTTTAGCTCCTCTCTCAATATAGATTCCATGGTGTTAAATATTCAAGAATATGTGACCCTTGGTTATGCCATGCTGAGCCCATTTGTGCTGATTTGCAGAGATGGAAAGCGGGGCGCATGGTTTTATCAGTTGAAAGACAGAAAAACACACAGAAAGTGTCTGTTTGATTGA

>Cavia_porcellus_intact_V1R660

ATGCATGTGCTGGATCCATTTTTCCCTATTAGGGTAGAAATGACATCTAACACTGCCAAGGGAGCAATCTCTGCCTGGCTTACTGGACTTGGGACTATGGGGAACATCTTTGTTTTTGTGAAGTACGTGCATGTATTTGCAAGCCGTGAGATGAAACCTATATATCTCATTCTCATCCATTTGGCTGTTACAAATATCATGACTCTGCTTTCCCAGGGAGTGCCAGGTACCTTAGCAGTTTTTGGTGTGAGGAACTTCCTAGATGACACATGCTGTAAGAGTGTTGTTTACTTGTCAAGAATAGCCCGAGGCCTCTCCATCTGCACCACAGGCCTCCTCACGGTGGTCCAGGCCATCACCATGAGTCCCAGACAGTCGGGATGGAGGAAGCTTCAGCCCAGTTCTGCATGGCACCTCCTTCCCTTGTGTCTTTTCTTTTGGATTCTCAATTCCTCAATAGGCATGAACTTACTCCTTTATATCACAAGCATAAGCAAAAATTCATCAAAAGGGAATATGAACCATATTCCCTGTCTTTTTCAACCAGAAAACCCAAAAGTGAAATGGTCATTTCTTGCAGCCATGGTAGTACGAGACATCATGTTTTTGGCCACTATGGGCACTGCCAGTGTCCACATCATCTTTCTTCTCCACAAACACCACCAGCGTGCTCTCTGCTTGCAGATCTCCAAGTCACTCCACAAAGCTGCCCCTGAGGTCAGAGCCTCTAAAAGTGTGCTCCTTCTGATGCTCTGCTTTGTTTTCTTCTATTGTACAGATTTTGTTTTTTCTTTGTTTGTTAATTCTTTCCGGGAGAATAATTCCTTTTTATTATATATTTGGAATTTTATGACCCTCGGTTATGCGATTGTCAGCCCATTTGTGCTGATTCACAGAGAAGGACACCTGACTGAGTGCAGGTGA

>Cavia_porcellus_intact_V1R661

ATGCCAGCTAACATTGTCGAGGGAACAATCTCTGCCTCGCTAACTGGACTTGGCACTACAGGGAACATCTTTGTGTTGGGGAAGTACGTGCTCATATTTGCAGGCAAAGAGCAGAAATCTATATACCTTATTCTCGTCCATTTGGCTGTTACAAACACCATGACTCTGCTTTCCAAGGGAGTGCCAGGTACCTTAGCAGCTTTGGGTGTGAGAAACCTCCTAGATGACACAGGCTGCAAGATTGTTGTTTATATGGAGAGGGTGTCCCGGGGCCTCTCCATCTGTACCAGCAGCTTCCTCACGGTGGTCCAGGCGGTCACCATCTGTCCCAGACACTCAGGATGGAGGAGGCTCAAGCCAAGGTCAGCACGCTATGTCCTCCTTGTCCTTCTCTATTTTTGGAGTCTCAACTCTTTGATAAGCATGAGCTTACTCCACTCCATCACAAATATCAGCACCAACTCATCAAAAGTTAGTAACAGTGAAAGGTACTGCTATTTTCTTCCAGAAAGTAAGAAGGTAAAGTGGATTTTTCTCACCTGCATGGTGCTGCGGGATGCAGGGTTTCAGGGCATCATGGGTGCAGCCAGCGGCTACATGGTGCGGGTTCTCCACAAACACCACCAGCAGATTCTTCATCTGCAGAATGCCAAGTTCCTCTACACAGCTCCTCCTGAAATCAAAGCTGCCCAAAGCATTCTCCTTCTGATGCTTTGCTTTATTGTGTTTTATTGGATAGACTGTCTCCTTTCTCTGATTATAAGTTGCTCCTTAGTGACTCTTTCCACTGTCATAAATGTTAAAGAATTTCTCACCCTTGGTTATGCAGTTTTTAGTCCATTTATTTTGATTCACAGAGAGGGACATCTGGGTCATTGTGGGGGCACTCGTGGGACACACAGAAGCTGA

>Cavia_porcellus_intact_V1R662_1

ATGCATGTACAGAATCCACTCTTACCTGTTATGTTAGAAATGCCAGCTAACATTGTCAAGGGAGCAATCTCTGCCTCACTAACTGGAGTTGGCACTATAGGGAATGTCTTTGTTTTTGTGAAGTACATGCTCGTGGTTGGAGGCAGTGAGATGAAACCTATACATCTCATTCTCATCCATTTGGCTGTTACAAATGCCATGAATCTGCTTTCCAAGGGAGTGCCAAGGACCTTAGCAGCTTTGGGTGTGAGAAACTTCCTAGATGACACAGGCTGCAAGATTGTTGTTTATATGGAGAGGGTGGCTTGGGGCCTTTCCATGTGTACCACCAGTCTCCTCACGGTGGTCCAGGCGGTCACCATCTGTCCCAGACACTCAGGATGGAAGAGGAGTAAGCCACGGTTTGCACGCTATGTCCTCCTTGTGCTTCTCTCTTTTTGGAGTCTAAACTCCTTGATAGCCACGAACTTACTCTACTCCATCACAAATATCAGCACGAACACATCAAAAGTTAGTAACACTGACAGTTACTGTTTTTTTCTAAGAGAAAGTAAGAAGATAAAGTTGATTTTTCTCATCTGCATGGTGCTGCGGGATGCGGGGTTTCAAGGCATCATGTGTGGGGCCAGCAGCTACATGGTGTGGGTTCTCCACAAACATCACCAGCAGGTTCTTTATCTGCAGAATGCCAAGTTCCTCTACACAGCTCCTCCTGAAATCAAAGCTGCCCAAAGCATTCTCCTTCTGATGCTTTGTTTTCTTGTGTTTTACTGGATAGATTGTCTCCTTTCTCTGTTTATAAGTTCCTCATAA

>Cavia_porcellus_intact_V1R662_2

ATGCATGTGTGGAATCCATTCTTGCCTGTTAGGTTAGAAATGCCAGCTAACATTGTCGAGGGAACAATCTCTGCCTCGCTAACTGGACTTGGCACTACAGGGAACATCTTTGTGTTGGGGAAGTACGTGCTCATATTTGCAGGCAAAGAGCAGAAATCTATATACCTTATTCTCGTCCATTTGGCTGTTACAAACACCATGACTCTGCTTTCCAAGGGAGTGCCAGGTACCTTAGCAGCTTTGGGTGTGAGAAACCTCCTAGATGACACAGGCTGCAAGATTGTTGTTTATATGGAGAGGGTGTCCCGGGGCCTCTCCATCTGTACCAGCAGCCTCCTCACGGTGGTCCAGGCGGTCACCATCTGTCCCAGACACTCAGGATGGAGGAGGCTCAAGCCAAGGTCAGCACGCTATGTCCTCCTTGTCCTTCTCTATTTTTGGAGTCTCAACTCTTTGATAAGCATGAATCTACTCCACTCCATCACAAATATCAGCCCCAACTCATCAAAGGTTAGTAACAGTGAAAGGTACTGCTATTTTCTTCCAGAAAGTAAGAAGGTCAAGTGGATTTTTCTCACCTGCATGGTGCTGCGGGATGCAGGGTTTCAGGGCATCATGGGTGCAGCCAGCGGCTACATGGTGCGGGTTCTCCACAAACACCACCAGCAGATTCTTCATCTGCAGAATGCCAAGTTCCTCTACACAGCTCCTCCTGAAATCAAAGCTGCCCAAAGCATTCTCCTTCTGATGCTTTGCTTTATTGTGTTTTATTGGATAGACTGTCTCCTTTCTCTGATTATAAGTTGCTCCTTAGTGACTCTTTCCACTGTCATAAATGTTAAAAAAATTCTCACCCTTGGTTATGCAGTTCTTAGTCCATTTATTTTGATTCACAGAGAGGGACACCTGGGTCATTGTGGGGGCACTCGTGGGACACACAGAAGCTGA

>Cavia_porcellus_intact_V1R663

ATGCACGTGCTGGATCTATTTTTGCCTATTAGGGTAGAAATGACATCTAACACTGTCAAGGGAGCAATCTCTGCCTGGCTTACTGGACTTGGGACTATGGGGAACATTTTTGTTTTTGTGAAGTACATGCATGTATTTGCAAGCCGTGAGATGAAACCTATACATCTCATTCTCATCCATTTGGCTGTTACAAATATCATGACTCTGCTTTCCCAGGGAGTGCCAGGTACCTTAGCCGTTTTTGGTGTGAGGAACTTCCTAGATGACACAGGCTGTAAGAGTGTTGTTTACTTGTCAAGAATAGCCCGGGGCCTCTCCATCTGCACCACTGGCCTCCTCACGGTGGTCCAGGCCATCACCATGAGTCCCAGACAGTGGGGATGGAGGAAGCTTCAGCCCAGTTCTGCATGGCACCTCCTTCCCTTGTGTCTTTTCTTTTGGGTTCTCAATTCCTCAATAGGCATGAACTTACTCCTTTATATCACAAGTATAAGCAAAAATTCATCAAAAGGGAATATGAACCACCTTCCCTGTCTTTTTCAACCAGAAAACCCAAAAGTGAAATGGACATTTCTCGCGGCCATGATAGTACGAGACATCGTGTTTTTGGCCACTATGGGCACTTCCAGTGTCCACATCATCTTTCTTCTCCACAAACACCACCAGCGTGCTCTCTGCCTACAGATCTCCAAGTCACTCCACAAAGCTGCCCCTGAGGTCAGAGCCTCTAAAAGTGTGCTCCTTCTGATGCTCTGCTTTGTTTTCTTCTATTGTACAGATTTTGTTTTTTCTTTGTTTGTTGATTCTTTCCAGGAGAGTAATTCCTTTTTATTATATATTTGGAATTTTATGACGCTCGGTTATGCGATTGTCAGCCCATTTGTGCTGATTCACAGAGAAGGACACCTGACCGAGTGCAGGCGAGCTCTGTGGAACACAAGGGACACTGAAAAGTTGGGTATTCCTTGGCCCTTTCCTTAG

>Cavia_porcellus_intact_V1R664

ATGCATGTGTGGAATCCATTCTTGCCTGTTAGGTTAGAAATGCCAGCTAACATTGTCGAGGGAACAATCTCTGCCTCGCTAACTGGACTTGGCACTACAGGGAACATCTTTGTGTTGGGGAAGTACGTGCTCATATTTGCAGGCAAAGAGCAGAAATCTATATACCTTATTCTCGTCCATTTGGCTGTTACAAACACCATGACTCTGCTTTCCAAGGGAGTGCCAGGTACCTTAGCAGCTTTGGGTGTGAGAAACCTCCTAGATGACACAGGCTGCAAGATTGTTGTTTATATGGAGAGGGTGTCCCGGGGCCTCTCCATCTGTACCAGCAGCCTCCTCACGGTGGTCCAGGCGGTCACCATCTGTCCCAGACACTCAGGATGGAGGAGGCTCAAGCCAAGGTCAGCACGCTATGTCCTCCTTGTCCTTCTCTATTTTTGGAGTCTCAACTCTTTGATAAGCATGAATCTACTCCACTCCATCACAAATATCAGCCCCAACTCATCAAAGGTTAGTAACAGTGAAAGGTACTGCTATTTTCTTCCAGAAAGTAAGAAGGTCAAGTGGATTTTTCTCACCTGCATGGTGCTGCGGGATGCAGGGTTTCAGGGCATCATGGGTGCAGCCAGCGGCTACATGGTGCGGGTTCTCCACAAACACCACCAGCAGATTCTTCATCTGCAGAATGCCAAGTTCCTCTACACAGCTCCTCCTGAAATCAAAGCTGCCCAAAGCATTCTCCTTCTGATGCTTTGCTTTATTGTGTTTTATTGGATAGACTGTCTCCTTTCTCTGATTATAAGTTGCTCCTTAGTGACTCTTTCCACTGCCGTAAATGTTAAAGAATTTCTCACACTTGGTTACGCAGTTCTTAGTCCATTTATTTTGATTCACAGAGAGGGACATCTGGGTCATTGTGGGGGCACTTGTGGGACACACAGAAGCTGA

>Cavia_porcellus_intact_V1R665

ATGCTTCGGGTGAACATCGTCCTGGGGATACTCTTCGTTTCTCTTGCTGGACCTGGAATTGGGGGGAATATTCTTGTATTTGTAAGACATGTATATAATTTTGTTTTGATTCCAAAGAAAAAACCCATAGATTTTATCATCATTCACCTGGTTTTTTCAAATGCAATCATTATTTGTAGCACAGGGCTTAGAGATATAGTCCTAATTTTCCATTTCAGAAACTTCCTAGGTAGTGCTGGCTGTAAAACTGTGGTTTACCTGGGGAGGGTGGCCCGAGGCCTGTCCATCTGCACCACCTGTCTCCTCAGCGTGGTCCAGGCAGTCACCATCAGTCCCAGGACCACCCTGTGGGGAAAGCTCAAGCCGCGGACTGCATGGCAAGTTCTTCCCTATCTTCTCCTCTGCTGGGTCATTAATCTTCCATTAAGCTCCAACTTGCTGCTCTACATCACAGCAGTTGGCAGCGTGAACAGATCTGAGATTGGAACCTATGTCAGACACTGTTTTTTGCTGTCATCCAGACCCACAGTCTGGTGGCTTTTCCTCTGTCTCATGGCTCTGCGGGATGTCCTCTTCCAGGGTGTCATGGGCTGGAGCAGCGGGCACATGGCTTTCCGCCTGTATGAGCATCACAAGCGTGTGCTCTATCTGCACAGCTCCAGGTTGGTGGTGAGTTCCAGCCCAGAAATCAGAGCTACCATAAATATTCTAGTCCTCATGACCTGTTTCCTTTTCTTTTTTTGGACAGATTTCATGTTCTCCTTCTACATAGGGTCCACGGTAGCCCGTGAAATTACACTCCTGTATGTTCAATTATTTCTAGAACTCGGTTATGCGGTTCTCAGCCCATTTGTGCTGATGAGCAGGGATGTTGCTGTCACTAAACCCTGA

>Cavia_porcellus_intact_V1R666

ATGAGAGCAATGGTTTTGGACCTTGTCAAAGGAACAATCCTAGCCTTTCTGGCCGGACTTGGCACTGCAGGGAACATCTTTGTTTTTGTAAATCATATACTCATGCTTAGGGGTACTGAGAAGAAGGTTGTGCACCTTATTCTCATCCATTTGGCCTTTGCAAATATCATAATGCTTCTGTCCAAGGGAATGCCAAGGACGATGGCAGATGTTGGTGTGAGAAACTTTCTGGATGACACCAGCTGTAAGATCGTGTGCTACCTGGAGCGGGTGGCCCGGAGCCTCTCCATCTGCACCAGCGGTCTCCTCACGGTGGTCCAGGCTGTCACTATGAGTCCCAGAAGCTCTCGGTGGAGGAGGCTCCGGCCCAGGTCTGCATGGTTGGTTCTTTCTTTGTTTCCCTTCCTGTGGATATTCAGTTTTTTGACAAACATCAGTTTACTCCTTTACATTACAAGTACTAGTGTGAACACATCACAAATGAGTGAGAGTGACTACTGTTATTTTCGGCCCAAAAATCAGAAGGTAAGATGGATCATTCTCAGTAGCATGGTCCTACGGGATGCTGTGTTTCAGGGTCTCACGTGTGGGCCGAGCGCCTACTTGGTGCTTCTTCTCCGTAGGCACCACCAGCATGTTCTCCACCTGCAGAGCTCCAAGTTCCTGTACAAAACTCCCACTGAGGTCAGAGCTGCCCGAAGTGTTCTCCTTCTGATGCTCTGTTTCCTCTTCTTTTATTGGGCAGATTGTGTTTTTGCTCTACTTGTGAATTCCTTCTTGAAGAGTAATCCTATGTTAAATATTCGAGAGTTTCTCACCCTTGGTTATGCGATTCTCAGCCCATTTGTGCTGATTCACAGAGATGGACATGTGGAGGACTGCTGGCCCTCTAGGTGGGAGAGCAGGACTTGCAGAAAATATTTATTCCCTGCATCCTTTAAAGTAAGTAAGTTTGAAATAACGCTTACTTTCATTGTGCTCTATGTAGTAAAAACTCAAATGAGAGTAGCTCACTTGAGACAAAAATACAAGTGA

>Cavia_porcellus_intact_V1R667

ATGATTTGGAGTAGCTACATCAAGTCAGCAATCGCTCTTCCCCTTTTGGGACCAGGAATCATAGTCAGCACTCTTATATTTGTGAGATATGTGTATACTTTTGTCAAAGTACCTGAGAAAAGACCTATAAATTCCATTCTTATACACTTGGTTTTCTCAAACGTGGTCATAATTTCTGTTGGAGGTATTGGAATCATTTCAACAGTTTTTTGTTTCAGAAACTTCCCAGGTAATGTAGGATCTAAAACTATGGTTTACCTGGGGCGGGTGGCACGGGGCCTCTCCATCTGTACCACCTGTCTCCTCAGCATGGTCCAGGCCATTACCATCAGTCCCAGGAACAGCCTGTGCAGAAAGCTCAAGCCGCAGACTGCATGGCAAGTTCTTCCCCATGTTCTCCTCTTTTGGGTTTGTAATTTTTTGATAAGTTCCAACTTGCTTTACTATGTCACAATAATCAATAGCACTAATTGTTCTGGAATTGGAATGTATATTAGCAAGTGCTACATGCTACCATCTCAGCAGATGGTTAGGTGGCTTTTTCTTTCTCTCATGACTCTTCGAGATATTACCTTCCAGAGTCTCATGAGCTGGAGCAGTGTGGATATGGCCGTCCATCTGCACAAACATCACAAGCGTGTCTTTTACCTGCATAGCTCCAAGTTTAGAAACAATTCCAGCCCAGAAATCACAGCTATCCAAAATACTCTCATTCTCATGACCTGTTTCCTTTTCTTTTATTGGGCAGATTTCATTTTCTCCTTCTATATAGGGTCAATCACAACACCTCATACAACAATAGTAAATATTAAATTGTTTCTAGAACTTGGTTTTGCCATTCTCAGCCCATACGTGCTGATTAGCAGGGATATCCACGTGGTTAATTGCTGGCATTGTCACTGA

>Cavia_porcellus_intact_V1R668

ATGAATAAAAAGTGGAAATTTTCCACGTATACTGATATAAGAAACGCAATATTCTTTGAAGTCATCATTGGGATCACAGCGAACACTGTCCTGCTTCTATTCCACATCTTCACATTTCTTTGCAAGCACAGGCCCAAGCCCCTTGATATGGCTATTAGTCAGTTGGCCCTGATTCACATGGTGTTGCTGGTAACCATGGCTGTCATAGCTACAGACATTTTTGGGTTTGAGGACTGGTGGAATGATCTCTTGTGTAAGTCAGTTATTTATGTAAACAGGTTGATGAGGGCCCTCTCCATCTGTACCACCTGCCTTTTGAGTGTCCTCCAGGCCATCACCCTCAGCCACAGAAACTCTTGCTTGGCAACATTCAAGCAGAAATCCACACATCACTACCCGTGCTGCCTTGTCTTCTTATGGATCTTCAATCTGCTCCTGAATAGTCGCTACTTAGTCTCTATTGGTGCCACTCCCAATGAGACTTCACAGAGACTCATGTTTGTCACTGAATCCTGCTCTCAGTGGCCTATCAGTTACTTGTTCAGATACATATTTCTCTCCTTGGTGAATATTCAGGATATCTCTTTTATAGTGTTGATGGCTCTCTCAGGTGGATACATGGTGAGTCTCTTATGCAGGCATAAGAGGCAGTTCCAACACCTTCACAGCACCAGTCTTTCTCCAAAGGCATCCCCAGAAGAAAAGGCTACTCAGACCATTCTGCTGCTCATGGGTTTCTTTATAATCATGTACTTATTGGACTGTGTTACTTTCTCATCCCTTGGAGTGTTGTGGAAAAATGATCCAATTCATCATTGTGTCCAGATGCTTGTGGGAAATGCTTATGCAACGATTTGTCCTTTTGTGCTAATGAGCACTGAAAAGCGAATGATTAAATGTTTAACTTTCAGGTATAAAATATAG

>Cavia_porcellus_intact_V1R669

ATGAATCTAAAGAGAAAAATTTCCAGTTTCACTGATATAAGAAACACAGTGTTCTTTGAAATCACCATTGGAATCACTGCCAACACTATCCTGCTTCTGTTCCACATCCTCGTATTTCTCCTAAAGTACAGGCCCAAGCCTCTTGACCTGCCTGTTGGGCAGTTAGCCTTAACTCACTTGGTGTTGCTGGTAGCCATGGGTTTCATAGCGAGGAACACTTTTGAGTCTCAAGGTTGGAAGGATGGCCTCATGTGTAAAGTGGTTATCTATTTACAAAAGTTGATGAGAGCCCTCTCCATCTGTACCACCTGCCTGCTGAGCATCCTCCAGGCCATCATGCTTAGTCCCAGAAATTCTTGTTTGGAAAAATTCAAACATAAGTCCACACACTACTACCCATATTGTCTTGTGTTCCTGTGGATCTTCAATATGCTTCTGAATGGTCGTTTCTTGGTCTCTATTGGTGCCACCCCCAATGTGACCTCACACAGTCTCATATTTGTCACTGAGTCCTGCTCTCAGTGGCCTATTAGTCTTTTGTTCAGATACATATTTTTTGCCTTGGTGAACATTCAGGATATCTCCTTTATTGGACTGATGGCTCTCTCAAGTGGATATATAGTGAGTCTCTTGTGCAGGCATAAGAGGAAGTTCCAACATCTTCACAGCACCCGCCATTCTCCAAAAGTATCCCCAGAAGAAAGGGCCACCCGGACTATTCTTCTCCTTATGAGTATCTTCACATTAATGTACTTTTTGGACTGTGTTACTTTCTCCTCGCTTGCAATACTATGGAAAAATGACCCAGTTCATCATTTTGTCCAGATGCTTGTGGGCAATGCCTATGCCACAATCTGTCCATTTTTGCTAATGAGCACTGAAACACGAATGATTCAGTGCTTAACATCAAAGTGGAAAAGATAG

>Cavia_porcellus_intact_V1R670

ATGATGAAAACAAGGAGAAAATTTTCCATTTTTCCTCAAATAGAAAACACAGTTTTCTTTGAAGTCACAATTGGAATCATAGCCAACACTGTCCTGCTACTGTTCCACATCCTCATATTTCTCTTCAAGCAAAGGCCCACACCTCTTGACCTGACTATCGGTCAGTTGGCTCTAATTCACCTGGTAATGCTGGTAACTGTGGGCATTATAGCTACAGACACTTTTGGGGTTCGTGACTGGGGGAGTGACTTCCCATGTAAATCAGTTATCTATGTATATAGGTTAATGAGGGGCCTTTCTATCTGCAATACTTGTGTCATGAGCATCATCCAGGCTGTCACTCTCAGTCCCAGAAACTCTTACATGGCAAAATTCAAACATAAATCTATACATCACTACCCATGTTGCCTTATCTTCATATGGGTCTTCAATATGCTCCTGAATGGTCGTTTCTTAGTCTCCATTGGTGCCACCCCCAATGTGACCTCACACAATCTTGTGTTTGTCTCTGAATCCTGCTCTCAGTGGCCCATTAATTATTTGTTCAGGTACATATTTTTCTCATTGGTGAACATTCAAGATATCTCCTTTATAGGGCTTATGGCTCTCTCAAGTGGATACATGGTGAGCCTGTTGTGCAGGCATAAGAAGCAATTCCAGCATCTTCACAGCACCAGCTTTTCTTCAAAAGTGACCCCAGAAGGAAGGGCCACCCGAACCATTCTGCTGCTTATGGGGTTCTTTATGATCATGTACTTTTTGGACTGTGTGTCCTTCTTTTCCTCTGGAGTATTGTGGAAAAATGATCCAATTAAGCATTTAATTCAGATGCTTTTGGGCAATGGCTATGCCACAATCTGTCCTTTTGTGCTAATAAGCACTGAAAAACGAATCATCAGGTGGTTAAAATTCAGATGCTAA

>Cavia_porcellus_intact_V1R671

ATGAAGATGAATAAAAAGAGAAAAATTTCCAGTTTCACTGACATAAGAAACACAGTGTTCTTTGAAATCACCATTGGAATCGCTGCCAACACCATCCTGCTTCTGTTCCACATCCTCACATTTCTCCTAAAGCACAGGACCAAGCCTCTTGACCTGCCTGTTGGGCAGTTAGCCTTAATTCACCTGGTGTTGCTTGTAGCCATGGGTTTCATAGCTAAGGACACTTTTGAGTCTCAAGGTTGGAAGGATGGCCTCGTGTGTAAAGTGGTTATCTATTTACAAAGATTGATGAGAGCCCTCTCCATCTGTACCACCTGCCTGCTGAGCATCCTCCAGGCCATCATGCTTAGTCCCAGAAACTCTTGTTTGGAAAAATTCAAACATAAGTACACACATTACTATCCATATTTTCTTGTGTTCCTATGGATGTTCAATATGGTTCTGAATGGTCGTTTCTTAGTCTCTATTGATGCCACCCCAAATGTGACCTCACACAGTCTCATATTTGTCACTGAATCCTGCTCTCAGTGGCCTATTAGTCGTTTGTTCAGATACATATTTTTTGCCTTGGTGAACATTCAGGATATCTCCTTTATTGGACTGATGGCTCTCTCAAGTGGATATATAGTGAGTCTCTTGTGCAGGCATAAGAGGCAGTTCCAACATCTTCACAGCACCAGCCATTCTCCACAGGGATCCCCAGAAGAAAGGGCCACCCGGACTATTCTTCTCCTTATGAGTATCTTCACATTAACGTACATTTTGGACTGTGTTACTTGCTCCTTGCTTGCAATACTATGGAAAAATGACCCAGTTCATCATTTTGTCCAGATGCTTGCGGGCAATGCCTATGCCACAGTCTGTCCATTTTTGCTAATGAGCACTGAAAAACGAATAATTCAATGCTTAACATCGAAGTGGAAAAGGTAA

>Cavia_porcellus_intact_V1R672

ATGAAAGCAAGGAGAAAATTTTCCATTTTTTCTCATATAGAAAACACAGTTTTCTTTGAAGTCACCATTGGAATCATAGCCAACACTGTCCTGCTACTGTTCCACATCCTCATATTTGTCCTCAAGCAAAGGCCCACACCTCTTGACCTGACTATCGGTCAGTTGGCTCTAATTCACCTGGTAATGCTGGTAACTGTGGGGTTTATAGCTACAGACACTTTTGGGGTTCGTGACTGGGGGAGTGACTTCCCATGTAAATCAGTTATCTATGTATATAGGTTAATGAGGGGCCTTTCTATCTGCACTACTTGTGTCATGAGCATCATCCAGGCTATCACTCTCAGTCCCAGAAACTCTTGCTTGGCAAAATTCAAACATAAATCTATACATCACTACCCATGTTGCCTTATCTTCATATGGGTCTTCAATATGCTCCTGAATGGTCGTTTCTTAGTTTCCATTGGTGCCACACCCAACGTGACCTCACACAGTCTTGTGTTTGTCTCTGAATCCTGCTCTCAGTGGCCCATTAATTATTTGTTCAGGTACATATTTTTCTCATTGGTGAACATTCAGGATATCTCCTTTATAGGGCTGATGGCTCTGTCAAGTGGATACATGGTGAGCCTGTTGTGCAGGCATAAGAAGCAGTTCCAAAATCTTCACAGCACCAGCTTTACTCCAAAAGTGTCCCCAGAAGGAAGGGCCATGCGAACCATTCTGCTGCTTATGGGGTTCTTTATGATCATGTACTTTTTGGACTGTGTATCCTTCTTTTCCTCTGGAGTATTGTGGAAAAATGATCCAATTAAGCATTTTATTCAGATACTTGTGGGCAATGGCTATGCCACAATCTGTCCTTTTGTGCTAATAAGCACTGAAAAACGAATCATCAGGTGGTTAAAATTCAGATGCTAA

>Cavia_porcellus_intact_V1R673

ATGAAGATGAATAAAAAGTGGAAATTCTCCAAGTATACTGATATAAGAAATGCAATATTCTTTGAAGTCATCATTGGGATCACAGCGAACACTGTCCTGCTTCTATTCCACATCTTCACATTTCTTTGCAAGCACAGGCCCAAGCCCCTCGATATGGCTATTAGTCAGTTGGCCCTGATTCACCTGGTGTTGCTGGTAACCATGGCTGTCATAGCTACAGACATTTTTGGGTTTGAGGACTGGTGGAATGATCTCTTGTGTAAATCAGTTATTTATGTAAACAGGTTGATGAGGGCCCTCTCCATCTGTACCACCTGCCTTTTGAGTGTCCTCCAGGCCATCACCCTCAGCAACAGAAACTCTTGCTTGGCAACATTGAAGCACAAATCCGCGCATCACTACCCATGCTGCCTTGTCCTCTTTTGGGTCTTCAATCTGCTCCTGAATGGTCGCTACTTAATTTCTATTGGTGCCACTCCCAATGAGACTTCACAGAGTCTCATGTTTGTCACTGAATCCTGCTCTCAGTGGCCTATCAGTTACTTGTTCAGGTACATATTTCTCTCCTTGGTGAATATTCAGGATATCTCTTTTATAGTGTTGATGGCCTTCTCAGGTGGATATATGGTAAGACTGTTATGCAGGCATAAGAGGCAGTTCCAACACCTTCACAGCACCAGCCTTTCTCCAAAGGCATCCCCAGAAGAAAAGGCTACTCAGACCATTCTGCTGCTCATGGGTTTCTTTATAATCATGTACTTGTTGGACTGTGTTACTTTCTCATCCCTTGGAGTGTTGTGGAAAGATGATCCCATTCATCATTGTGTCCTGATGCTTGTGGGAAATGCTTATGCAACAATTTGTCCTTTTGTGCTAATGAGCACTGAAAAGCGAATGATTAAATGTTTAACTTTCAGGTGTGAAATATAG

>Cavia_porcellus_intact_V1R674

ATGAAAGCAAGGAGAACATTTTCCATTTTTTCTCATATAGAAAAAACAGTTTTCTTTGAAGTCACCATTGGAATCATAGCCAACACTGTCCTGCTACTGTTCCACATCCTCATGTTTCTCCTCAAGCAAAGGCCCACACCTCTTGACCTGACTATCGGTCAGTTGGCTCTAATTCACCTGGTAATGCTGGTAACTGTGGGCCTTATAGCTACAGACACTTTTGGGGTTCGTGACTGGGGGAGTGACTTCCCGTGTAAATCAGTTATCTATATATATAAGTTAATGAGGGGCCTTTCTATCTGTACTACTTGTGTCATGAGCGTCTTCCAGGCTGTCACTCTCAGTCCCAGAAACTCTTGCTTGGCCAAATTCAAACATAAATCTATACATCACTACCCATGTTGCCTTATCTTCATATGGGTCTTCAATATGCTCCTGAATGGCCGTTTCTTAGTTTCCATTGGTGCCACCCCCAATGTGACCTCACACAATCTTGTGTTTGTCTCTGAATCCTGCTCTCAGTGGCCCATTAATTATTTGTTCAGGTACATATTTTTCTCATTGGTGAACATTCAAGATATCTCCTTTATAGGGTTGATGGTTCTCTCAAGTGGATACATGGTGAGCCTGTTGTGCAGGCATAAGAGGCAGTTCCAGCATCTTCACAGCATCAGCTTTTCTCCAAAAGTATCCCCAGAAGGAAGGGCCACCCGAACCATTCTTCTGCTCATGGGGTTCTTTATGATCATGTACTTTTTGGACTGTGTGTCCTTCTTTTCCTCTGGAATACTGTGGAAAAGTGATCCAATTAAGCATTTTATTCAGATGCTTGTGGGCAATGGCTATGCCACAATCTGTCCTTTTGTGCTAATAAGCACTGAAAAACGAATCATCAGGTGGTTAAAATTCAGATGCTAA

>Cavia_porcellus_intact_V1R675

ATGAATAAAAAGAGAAAAATTTCCAGGTACACTGACATAAGAAACACTGTGTTCTTTGAAATCACCATTGGAATCACTGCCAACACCATCCTGCTTCTGTTCCACATCCTCACATTTCTCCTAAAGCACAGGCCCAAGCCTCTTGACCTGCCTATTGGGCAGTTAACCTTAATTCACCTGGTGTTGCTTGTAGCCATGGGTTTCATAGCTAAGGACACATTTGAGTCTCAAGGTTGGAAGGATGGCCTCATGTGTAAAGTGGTTATCTATTTACAAAGGTTGATGAGAGCCCTCTCCATCTGTACCACCTGCCTGCTGAGCATCCTCCAGGCCATCATGCTTAGTCCCAGAAACTCTTGTTTGGAAAAATTCAAACATAAGTCCACACATTACTATCCATATTGTCTTGTGTTCCTGTGGATCTTCAATATGCTTCTGAATGGTCGTTTCTTAGTCTCTATTGGTGCCACCCCCAATGTGACCTCACACAGTCTCATATTTGTCACTGAATCCTGTTCTCAGTGGCCTATTAATCATTTGTTCAGATACATATTTTTTGCCTTGGTGAACATTCAGGATATCTCCTTTATTGGACTGATGGCTCTCTCAAGTGGATATATAGTGAGTCTCTTGTGCAGGCATAAGAGGCGGTTCCAACATCTTCACAGCACCAGCCATTCTCCACAGGTATCCCCAGAAGAAAGGGCCACCCGGACTATTCTTCTCCTTATGAGTATCTTCACATTAATGTACTTTTTGGACTGTGTTACTTTCTCCTTGCTTGCAATACTATGGAAAAATGACCCAGTTCATCATTTTGTTCAGATGCTTGTAGGCAATGCCTATGCCACAATCTGTCCATTTTTGCTAATGAGCACTGAAAAACGAATAATTCAATGCTTAACATCGAAGTGGAAAATATAG

>Cavia_porcellus_intact_V1R676

ATGTTACTCAACAGCATGGTGATCAGAATCTTCTTTGTTGTTCAGACAGGAATTGGAATCCTGGGAAACATTTTGCTCCTTTATTGTTATGTCTTCATTCCCTACCCTGAGCGAAGGCTAAAACACATGGATTTGATTCTCAAAAATTTAATTTTGGCAAACTGTTTGGTTCTTCTCTCCAAAGGAGTCCCTCACACAATATTAACCTTGGGGCTGCAATTCTCTATGGGTGATGTTGGGTGTAAATTGGTCTTCTATCTTCACAGGGTGGCCCGGGGTGTGACTCTTGGTACTACCTGTATCTTGAGTGGCTTCCAGTCCATCACAATCAGTCCTAATTGCTGCCAGTGGATAAGATTCAAGGTCAAATCTCTCAAGTGCACAAGATTTTCCATTACCTTCTGCTGGATTCTTCAACTGATGGTAAATAGTATTTTTCCTTTGTATATGAGAGGTGCAAAGGAAAACAACAGCTACACACATGATAGAGACCTTGGCTACTGCTCCAGTATGAGTAGTGAGAGAGTGACATCCTTATTGCACTCTGTGATGTTGTCTTCCATTGATGTGTTGTCTTTGGGGTTTATGGTGTGGAGCAGTGTCTTTATGATGCTCATTTTGTACAGACACAAGCAAAATGTCCTACATATTCACAGCAAGAACCTCTCTTCCAAATCCTCTGCTGAGACCAGAGCCACACAGACCATTTTAATTCTATTAATCACATTTTTCTCTGTATACACCCTATCTTCATTCTTTACTTCTTACATATCTCACTTGGATAAACCCTGTCAGTGGCTGGTAGACACGAGTGCACTTTTAGCTGCCAGCTTCTCATGTTTCAATCCATTTATACTCATCTGCAGAGACCCACATTTCACCATACTTTTTTTCGCACACTGCCCAAAGATGAACAATTTTGGTAAGCTGATCATGTACTCTTAA

>Cavia_porcellus_intact_V1R677

ATGGATGGATTGGTTTTTGCCGATTTAAATTGGGCTATTGTCTTCCTCACTCAGACTAGTATTGGAATCCTTGGAAATTCTTTTCTGTTTTGCCTCTATAACTTTTCTCTGCTCACTGCACGAGGGTTGAGACTCATAGATGTGATTCTCATACAGCTATTCCTAGCTAACAACTTGGTACTGTTCTCTAAAGGGATTCCACAGACAATTGCAGCTTTTGGATTGAAAGATTTCCTGGAGGAGACTGGATGTAAAATTGTCTTCTACTTGCACAGAGTGGCTAGAGGCGTTACCCTCAGCACCACTTGCCTACTCAGTGGCTTCCAAGCCATGACAATTTGCCCCACTATTTCAGGATACATGGAGAGCAAAATTAAATCACTGAAGTGTCTTGCCTTCTGCTGTTTTCTCTGTTGGATCCTGAACCTCCTGATAAATATCCATAATATAATGAATGTAAGTGGACCAAACAACAGCAGGAACATGAGTATACACAACATGTATAGATACTGCTCTGCTCCAGTCGCTAAGGGTTTTACATTCACACTACTAGCAATGCTTTATGTCCTTACTGATTGGACCTTTTTGGGTCTCATGGCCTGGGCCAGTGGTTCCATGGTCCTTCTGTTGCACAGACACAGACAAAGAGTCCAGCACCTTCACAGCCAGAGTCTCTCCCCCAGAACTTCTCATGAGGCCAGAGCTGAGTGCACTGTGCTAATCCTGGTGAGTATGTTTGTCTCCTTTTACTTTCTGGCTGCTTCTTTATCATTGTGGAAAACCCAGGCCCTGAGCCCAAGCCCCCGGCTCATGAGCATCTCTGTGCTGCTGTCCTTGGGCTTCCCCACATTCAGCCCCTTTGTGTTTCTCTTCAGTGATACTCATATCTACCAATTCTGTTTTGTCATATGGGCGAGAAAAACAAATATTTCAACTTTTGTCTCTGGGGTTTGA

>Cavia_porcellus_intact_V1R678

ATGGATGGATTGGTTTTTGCCAATTTAAATTGGGCTATTGTCTGCCTCATTGAGACTAGTGTTGGAATCCTTGGAAATTCTTTTCTGTTTTGCATGTATAGCTATTCTCTGCTCACTGCACGAGGGTTGAGGTGCATAGATGTGATCCTCATACAACTATTTCTAGCTAACGACTTGGTACTGTTCTCTAAAGGGATTCCTCAGACAATGGCAGCTTTTGGACTGAAAGATTTCTTGGAGGAGACTGGATGTAAAGTTGTCTTCTACTTGCACAGAGTGGCTAGAGGCATTACCCTCAGCACCACTTGCCTACTCAGTGGCTTCCAAGCTATGACAATTTGCCCCACTTTGCCCCATGCTCCAGGATGCATGGAGAGCAAAATTAAATCACTGAAGTGTCTTGCCTTCTGCTGTTTTCTCTGTTGGGCTATGAACCTCCTGATAAATACCCATAATATAATGAATGTAAGTGGACCAAACAACAGCAGGAACATGAGTATACAAAACATATATAGATACTGCTCCGCTCCAGTCGCTAAGGGACTTACATTCACACTACTAGCAATGCTTTATATCCTTACTGATTGGGCCTTTTTGGGTCTCATGGCCTGGGCCAGTGGTTCCATGGTCCTTCTATTGCACAGACACAGACAACGAGTCCAGCACCTTCACAGCCGTAGTCTCTCCCCCAGAACTTCCCATGAGGCCAGAGCTGGGTGCACTGTGCTAATCCTGGTGAGTATATTTGTCTCCTTTTACTTTCTGGCTGCTCTTTTATCATTTTGGATAACCCAGGCCCTGAGCCCAAGCCCCTGGCTCATGAACACCTCCGTGCTGCTGTCCTTGGGCTTCCCCACATTCAGCCCCTTTGTGTTTCTCTTCAGTGATACTCATATCTATCAATTATGTTTTGTCACATGGACAAGAAAAACAAATATTTAA

>Cavia_porcellus_intact_V1R679

ATGGATGTGAGATTTGCAATCCTCTTTCTATTCCCTGTTGTCATTGGAGGCCTGGGGAATCTCTCACTGCTATGTTATTCAGTCTGCATTTACTGCAGTGGGCGCAGGTCCAGATCCACAGATCTGATTGTCAGGCAACTGACTGTGGCCAACTGCTTGTTCATATTTTCCCGAGGACTCCCACAGACCATGGCAGTGCTTGGGATGGAAGACTTCCTTACTAATGTTGGATGCAAACTGTTTTTCTATGTTCACGGTGTGGCTAGAGGTGTTTCTTTCAACACTACCTGTCTGCTGAGTGTCTTCCAGGCCGTCTCTGTTAGCCCCAGAAGCTTCAGGTGGACAGAGCAGAAGGTAAAAGTTCTAAAGTGCATTGGCCCTTGTACCATCATTTGTTGGGTCATACACATGCTGCTAAACATCAGAATTCCTATGCTTGTGAGTGACAAAAGGAACAATGAAAATATCACAAACACTTTAAATTTTCATTACTGTTCAGCTGTCATTACTAGCAAAGACAAAGGCATATTTGTGCCATTGTCATTATCATACGATGTTTTGTGTTTGACACTTATGATCTGGAGCAGTGGCTCCATGGTTTTCATTCTATACAGGCACAAGCAGCAAACACAACACATTCACAGAAACAACATCTCAACAAGATCTTCCCCTGAGACCAGAGCCTCTCAAAGCATCATCATCCTGGTCTGTACCTTTGTGTCTTTCTATGCCCTGTCTTACATCATGATTGTTTGGTTCTCTCTTTATGATCGAAGTGCTTGGTGGCTAGTTGAAATTGCAGGCTTAACAAGTGCTTGTTTCCCTACTGCTAGTCCATTCATTCTCATGACTCGTGAGCAGTGTGTCCGCAGGTCCATGTGTAAGAAGTGA

>Cavia_porcellus_intact_V1R680

ATGGGCTCTGAGAGTGTGTATATTGTAATAATCTTCAGTTTTCAGACTCTCATTGGAACTCTGGGGAATCTCTTTCTCTTAGGTAAATATATATTTCTTTATCTCAGTGGACATAAAACAAAACCCATAGATTTGATTATTATGCATCTGATTGTGGCCAACTTATTGGTCATTCTCTTTAGAGGGGTCCCAGAGATTTTGGCAGCTTATGGGTTGGAAGACTTCCTCAGTGACTTTGGATGCAAGTTTGTTTACTATATTAGCAGGGTAGGCAGAGGTGTAGCCTTTGGTAGCACTTGTTTCCTGAGTGTCTTCCAGGCCATCACCATCAGCCCCAACAATTGTAGGTGGACTGAGCTCAAATTGAAATTTCCTAAGTACATAAACACCTTAACTACCCTACTCTGGGTCCTGCATTTGTTGCTGAATATTTGTTTCTCTGTGAGCTTGACTATGAAATTGAGAAACAAAAATGTTACTAGTAAGATAGACTTTGCTTACTGTTCAGATCCGAATACCAAGAGAGGGATATACACGGGGCATGCAGTGTTAACTGCCATATTTGATGTTTCATGCATGGGGCTCATGCTCTTAGCCTGTGGTTCCATGATTTTCATTTTATTTATGCACAAGCGACGGATTCAGCACATCCATAGGTCCAACCACTACTTCAGGTCCTCTGCTGAGACCAGAGCCACCCACAGCATCCTTATCCTGGTGAGCATCTTTATGTTTTTCTATTTCATGTCTTGTATCATGCAAATATATGTGACTTTTTTTCATCATTCGAGAATCTTGCTGTTTACACTTGATGCTTTCATAAGTGCATGTTTCTCAACTGTCTGTCCTTTTGTATTCATGAATTGTGGTCACTGTGTATCTGGAATATGCTGTTTCTATGGTCAAAGAAACAGAGTCACCTAA

>Cavia_porcellus_intact_V1R681

ATGGGCTCTGAGAGTGTGTATATTGTAATAATCTTCAGTTTTCAGACTCTCATTGGAACTCTGGGGAATCTCTTTCTCTTAGGTAAATACATATTTCTTTATCTCAGTGGACATAAAACAAAACCCACAGATTTGATTATTATGCATCTGATTGTGGCCAACTTATTGGTCATTCTCTTTAGAGGGGTCCCAGAGATTTTGGCAGCTTATGGGTTGGAAGACTTCCTCAGTGACTTTGGATGCAAGTTTGTTTACTATATTAGCAGGGTAGGCAGAGGTGTAGCCTTTGGTAGCACTTGTTTCCTGAGTGTCTTCCAGGCCATCACCATCTGCCCCAACAATTGCAGGTGGACTGAGCTCAAATTGAAATTTCCTAAGTACATAAACACCTTAACTATCCTACTCTGGGTCCTGCATTTGTTGCTGAATATTTGTTTCTTGGTGAGCTTGACTACGAAATTGAGAACCAAAAATGTTACTAGTAAGATAGACTTTGCTTATTGTTCAGATCCGAATACCAAGAGAGGGATATACACGGGGCATGCAGTGTTAACTGCCATATTTGATGTTTCATGCATGGGGCTCATGCTCTTAGCCTGTGGTTCCATGATTTTCATTTTATTTATGCACAAGCGACGGATTCAGCACATCCATAGGTCCAACCACTCCTTCAGGTCCTCTGCTGAGACCAGAGCCACCCACAGCATCCTTATCCTGGTGAGCATCTTTATGTTTTTCAATTTCCTGTCTTGTGTCATGCAAATATATGTGACTTTTTTTCATCATTCGAGAATCTTGCTGTTTACCCTTGATGCTTTCATAAGTGCATGTTTCTCAACTGTCTGTCCTTTTGTATTCATGAATTGTGGTCACTGTGTATCTGGAATATGCTGCTTCTATGGTTAA

>Cavia_porcellus_intact_V1R682

ATGGGCTCTGAGAGTGTGTATATTGTAATAATCTTCAGTTTTCAGACTCTCATTGGAACTCTGGGGAATCTCTTTCTCTTAGGTAAATATATATTTCTTTATCTCAGTGGACATAAAACAAAACCCACAGATATGATTATTATGCATCTGATTGTGGCCAACTTATTGGTCATTCTCTTTAGAGGGGTCCCAGAGATTTTGGCAGCTTATGGGTTGGAAGACTTCCTCAGTGACTTTGGATGCAAGTTTGTTTACTATATTAGCAGGGTAGGCAGAGGTGTAGCCTTTGGTAGCACTTGTTTCCTGAGTGTCTTCCAGGCCATCACCATCAGCCCCAGCAATTGCAGGTGGACTGAGCTCAAATTGAAATTTCCTAAGTACATAAACACCTTAACTACCCTACTCTGGGTCCTGCATTTGTTGCTGAATATTTGTTTTTCTGTGAGCTTGACTATGAAATTGAGAAACAAAAATGTTACTAGTAAGATAGACTTTGCTTACTGTTCAGATCCGAATACCAAGAGAGGGATATACACGGGGCATGCAGTGTTAACTGCCATATTTGATGTTTCATGCATGGGGCTCATGCTCTTAGCCTGTGGTTCCATGATTTTCATTTTATTTATGCACAAGCGACGGATTCAGCACATCCATAGGTCCAACCACTCCTTCAGGTCCTCTGCTGAGACCAGAGCCACCCACAGCATCCTTATCCTGGTGAGCATCTTTATGTTTTTCTATTTCCTGTCTTGTATCATGCAAATATATGTGACTTTTTTTCATCATTTGAGAATCTTGCTGTTTACCCTTGATGCTTTCATAAGTGCATGTTTCTCAACTGTCTGTCCTTTTGTATTCATGAATTGTGGTCACTGTGTATCTGGAATATGCTGTTTCTATGGTCAAAGAAACAGAGTCACCTAA

>Cavia_porcellus_intact_V1R683

ATGGATTTGAAATTTGGGATATTTCTTATACAGATTGTCATTGGAACTATCGGCAATTTTTCACTCTTGTGTTATTATATCTTCCTATACTTCAATGGATACAGGCCAAGGTCAACAGATTTGTTTCTCAGACATCTGATTGTGGCCAACTCCTTAGATATTCTCTCCAGAGGGATCCCAGAGTCCAAGGCAGCTTTTGAAAAGAAACATTTCCTTGATGATCTAGGATGTAAGTGTCTTTTCTATCTTCACAGGGTAGGCAGGGGTGTGTCTATCACCTCCACCTGCCTCTTGAGTGTGTTCCAAGCCATCACCATCAGCCCCAGGGACTACATCTGTTCACAACTGAAACTGAAAGCCATAAAATACATGCACCCTTTTATCATCCTCTGCTGGGTCCTGCACTTGCTGCTAGCTAGTAGAACTCTTGAGCGTATCATTGATAAAGGGAACAAGAAAAACTTCTCCAGAACTATAGATTTCCAGCATTGTTCAGCGATAATTCCTAACAATGACTCAGGCATGGTTTTTGCCGCAGTGACATTATCCCATGACATTTTGTGTTTACAGCTCATGATCGGGGCCAGTGGTTTCATGGTTTTCATTCTGCAAAGGCACAAGCAGCAGGTCCACCATATCCACAGACACAGCTCAGGTAGATCTTCCGCTGAAACCAGAGCTTCTCAAAGCATCCTTGCTTTGGTTAGTGCCTTCGTGTTCTTCTACACTGTGTCATCTACCCTTCACGCTTGCTTAGCTCTTAATGATAAAACTGCTTTGTGGCTGTTGAATGCCACTGTCCTAATCAGTGCTGGTTTCCCCACCATCAGCCCCATCATTCTCTTGAGTCGGGAATGCAGCTTAACCAGGCTCATCTCGAAGAAGTGA

>Cavia_porcellus_intact_V1R684

ATGAACCCCTTGGGCTTGCTCATTGGAATGTTTTTTCTGATTCAGACAGTCACTGGAATCTTAGCTAACATTGTCCTTCTGTTCCACTATCTCTCTCTGTGCTTCACTGCATATAAGTTAAGGCCTACAGATCTAATAGCAGAGCACTTAACTATAGCCAACACACTGATCATGATGTCAAGGGGAATCCCAAAGACAATGCAAGTCTTTGGGCTAAAGTATTTCTGTCAATTTAGTAGATGCACACTTCTGTTATATGTCTACAGAGTGGCTAGGGGTGTATCCATTAGCACAACCTGCCTACTGAGTGTCTTCCAGACTATTAAGATTAGCCCCATGAACTCCAGCTGGAAGGCGCTTAAAGCTAAACTTCCCAAGAACATTGGCTTGCTGATTTTTTTCAGTTGGATTCTCCATAAACTGATAAATTGTATTATTCTATTTTATGACATCATCAAATACACCAGCAGAAATATCACAGAGTTAAAAGATTTTGGATATTGTTCTTGTATCTTTAGAGATGAAATCATAGAAAACTTGTACACAACATTTGTTTTATTGCCCGAAGGTTTATATTCTGGCCTCATGATCTGGTCCAGTGGTTCAATGATTTTCTTTTTGCACAGGCACAAGAAGCGGATGCAATACATTCATAAGACAAATGATTTCCACAAAACTTCCCCTGAGACCAGGGCCACCCACAGCATCTTCATCCTTATATGCACTTTTGTTTCTTTTTACATGCTCTCCTCATTCTTTCATGCTTGCATTGCAAATTTCAGAAATCCCAGTTGGTGGCTCATGAACACCTCTGCACTGTTGGCCACCTGCTTCCCCACTGTCAGCCCATTCGTTCTAATGAGCTGCAACTCTACTGCAGCCAGGCTCTGGATCAGATTATCAGGTTTTTTCAGAAGCATGTAG

>Cavia_porcellus_intact_V1R685

ATGACATCTATGGATATGAAATTTGTAATCCTCTTCCTTTTCCCAGTTGTCATTGGAAGCCTGGGTAATTTCTCACTTTTATATCATTATATCTTCATTTCCTTCAGTGAACAAAGAACCATGCCAACGTACTTTATTCTCAGGCACCTGACAGTGGCTAATCTCTTGGTCATTCTCTCCCGAGGAATCCCAGAGACCATGGCAGCACTTGGGATAGAAGACTTCCTCAATAATTTTTGGTGCAAACTCATTTTCTATGTTTTAATGGTGGGGAAGGGTGTGTCTTTCAGCACCACCTGCCTGCTGAGTGTCTTCCAGGCCATCACCATCAGCCCCAGGAGCACCAGGTGGGTAGAGCTGAAAGTGAGAGCACTAAAGTGCATTAGGTCCTTTCCCAGCATTTTCTGGGTCCTGCACATGCTGGCAAACATCAGAGTTCCTATGCTTGTGACTGACAAAAGGCACAATAAAAACATCACAAACACTATAGATTATCAATACTGTTCAGCTGCAATCTCTAGCAAAGAAATAAACTACATCTTTATGGCATTGTCATTATCACATGATATTTTCTGTTTGGAACTTATGATGTGGAGTAGTAGCTCCATGGTTTTCATTCTGTACCAGCACAAGCAGCAAACTCAGTATATTCACAGATACAAAAGTTCATCAGTATCTTCCCCTGAAACCAGAGCTGTTCAAAGCATCCTCCTCCTGGTCTGTGCTTTTGTTTCCTTCTATGTCCTGTCTTCCATTATGAATGTTTGGTTTTCTCTTTTTGACAGATCTGCTTGGTGGCTGGTTAAGACTTCTGCTTTAACCCATGTTTATTTTCCTACTATTAGTTCTTTTATTTTCATGACTCATGACCAGTGTGTATGCAAGCCAATGTGGAAGAAGTAA

>Cavia_porcellus_intact_V1R686

ATGAATGTGAACAAGGAATTGTCCAATAATATTAACATCAGAATCACATTTTATTTTCAGTTTATGATTGGGGTCTCAGGCAATGCCATCCTTCTTCTCTTCCACATCGTTGTGTTTGTTGTAGGGCACAGGCCCAGACTCACTGACCTGCCTATTGGCCTCTTGGCCCTAAACCACCTAATGACAATGCTAACCACAGGATTCATAACTGAAGACATTTTTACCTCTCAAGGGGGGTTTTGGAATGATATTACATGTCAATCAGTTATCTACTTGAACAGGTCGATGAGGGGGCTCTCCATGTGTATCACCTGCCATCTGAGTGTCCTTCAGGCTATCACGCTCAGCCCCAGAAGCTCTTGTTTGGCAAAGTTAAAACACCAATCCTCATGTCACAGCCTGTGTTCCCTTCTTTTCTTTTGGCTGTTATATATATCCATTAGCAGTCACCTTTTTATCTCCATCGCTGCTAGTACCAATTTAACTTCAGAAAATATTTTGTATGTCAGTAAGTCCTGTTCTCTTTTACCCATGAACTATTTACTCCGGCACATAGTTTTCATATTATTGACCATCAGAGATATGTTCTTTATAGGAATTGTGTCCCTCTCAGGTGGATACATAGTGATTCTCTTGTGCAGGCATAAGAAAAAGTCCCAGCATCTTCACAGGAACAAAGTTTCTCCAAAAGCATCCCCAGAACAAAGGGCCACTCGGACCATTCTGCTGCTTATGTGTCTCTTTGTAGTTATCACCAGTTTGGACATTATATCTTCCTCAAGAATTGTGTTGAATAATAATCCAGTAATATATTGTATTCAAATTTTTGTGGCCCATGGCTATGCCACAGTCAGTCCTTTAGTATTCATTACTACTGAAAAACGTATCATTAATATTTTGAGACTCATGTGTGGAAGAAAATAA

>Cavia_porcellus_intact_V1R687

ATGGGCTCTGAGAGTGTGTATATTGTAATAATCTTCAGTTTTCAGACTCTCATTGGAACTCTGGGGAATCTCTTTCTCTTAGGTAAATATATATTTCTTTATCTCAGTGGACATAAAACAAAACCTACAGATTTGATTATTATGCATCTGATTGTGGCCAACTTATTGGTCATTCTCTTTAGAGGGGTCCCAGAGATTTTGGCAGCTTATGGGTTGGAAGACTTCCTCAGTGACTTTGGATGCAAGTTTGTTTACTATATTAGCAGGGTAGGCAGAGGTGTAGCCTTTGGTAGCACTTGTTTCCTGAGTGTCTTCCAGGCCATCACCATCAGCCCCAGCAATTGCAGGTGGACTGAGCTCAAATTGAAATTTCCTAAGTACATAAACACCTTAACTATCCTACTCTGGGTCCTGCATTTGTTGCTGAATATTTGTTTTTCTGTGAGCTTGACTATGAAATTGAGAAACAAAAATGTTACTAGTAAGATAGACTTTGCTTACTGTTCAGATCCGAATACCAAGAGAGGGATATACACGGGGCATGCAGTGTTAACTGCCATATTTGATGTTTCATGCATGGGGCTCATGCTCTTAGCCTGTGGTTCCATGATTTTCATTTTATTTATGCACAAGCGACGGATTCAGCACATCCATAGGTCCAACCACTCCTTCAGGTCCTCTGCTGAGACCAGAGCCACGCACAGCATCCTTATCCTGGTGAGCATCTTTATGTTTTTCTATTTCCTGTCTTGTATCATGCAAATATATGTGACTTTTTTTCATCATTCGAGAATCTTGCTGTTTACCCTTGATGCTTTCATAAGTGCATGTTTCTCAACTGTCTGTCCTTTTGTATTCATGAATTGTGGTCACTGTGTATCTGGAATATGCTGTTTCTATGGTCAATGA

>Cavia_porcellus_intact_V1R688

ATGGGCTCTGAGAGTGTGTATATTGTAATAATCTTCAGTTTTCAGACTCTCATTGGAACTCTGGGGAATCTCTTTCTCTTAGGTAAATACATATTTCTTTATCTCAGTGGACATAAAACAAAACCCACAGATTTGATTATTATGCATCTGATTGTGGCCAACTTATTGGTCATTCTCTTTAGAGGGGTCCCAGAGATTTTGGCAGCTTATGGGTTGGAAGACTTCCTCAGTGACTTTGGATGCAAGTTTGTTTACTATATTAGCAGGGTAGGCAGAGGTGTAGCCTTTGGTAGCACTTGTTTCCTGAGTGTCTTCCAGGCCATCACCATCAGCCCCAGCAATTGCAGGTGGACTGAGCTCAAATTGAAATTTCCTAAGTACATAAACACCTTAACTACCCTACTCTGGGTCCTGCATTTGTTGCTGAATATTAGTTTTTCTGTGAGCTTGACTATGAAATTGAGAAACAAAAATGTTACTAGTAAGATAGACTTTGCTTACTGTTCAGATCCGAATACCAAGAGAGGGATATACACGGGGCATGCAGTGTTAACTGCCATATTTGATGTTTCATGCATGGGGCTCATGCTCTTAGCCTGTGGTTCCATAATTTTCATTTTATTTATGCACAAGCGACGGATTCAGCACATCCATAGGTCCAACCACTCCTTCAGGTCCTCTGCTGAGACCAGAGCCACCCACAGCATCCTTATCCTGGTGAGCATCTTTATGTTTTTCTATTTCCTGTCTTGTATCATGCAAATATATGTGACTTTTTTTCATCATTCGAGAATCTTGCTGTTTACCCTTGATGCTTTCATAAGTGCATGTTTCTCAACTGTCTGTCCTTTTGTATTCATGAATTGTGGTCACTGTGTATCTGGAATATGCTGTTTCTATGGTCAAGGAAACAGAGTCACCTAA

>Chinchilla_lanigera_intact_ancV1R

ATGAAGCTCTCTGCAGACCTGCTTGAGATCATCACTTGTGCCATCCTGATCCTTGTGAGCTTTGTAGGAAATGTATGTTTATTTTATTCTACAAGGAAGTGTATCACTGGACGTTTACAGACATCGTTTCTTCTAATTTTCAGCCTTGTGTTTGTCCACCTTATTAAGAACTTGGTGGTAAATGTCATGAAAATTGTTTATTCTTCTGGCATCATGGTGGATTCAGCCGGCTGCAAAGTTCTGCACTTCACGGCAGCCCTGACAACATCACTGGCCATCTGGTTCATGTTATACTTCGCATTGTTCTACCACCTGAAACTTTACCAAACTGTCTACCCCTTGAGAGGTGCTGCAAACCTAGACCAGCAGAAACATTGCTTGAAGGGAATTTCTGCTCTTTGGGTGGCTGCTGTGGCTGTGTACATACCAGTTTTAATTTATACTAGAAAACCAGAATACCTGAATGCAGGAAATGATACAGACTCCTTGTCTACGAAGAGGATTTACATAGATTGCATAATTGGCTTTGGAAACAAGCAGGTAGAGTTTTACTATGGGAAAATATTTTTAGTTCTGATTGATGTTCTTCCCTTAGCCGTCTTAGTCTTTGTCTGTTTCTGGATGTCTTTACTCCTTTCATCAAAGAAGAAGATGACATATGGTGACATCTGGATTGGAGATGATGATTCAGAAGTTGAAATCCTTAGAGGGGCCAGGTTCAGTATTTTATTAATGTTACTGATCACTCCACTTTGGATTTCTCACTTTATCTTAGTCTGTTTCTTGAGCAGCGTGGCCACGTGCATGTTTATCCCAGCTGTGCTCACAGCCCTGTCGTCGGGCTTCTCTGCTCTCAGTCCTGTGCTGCTGATGCTAGTTCACTACAGAGTGAGGCTGGTGTCATTCTGTGGTGCCAAAGTGGAAAATCCCACACCACAGCCAACCAATGTTATTCTTTCTCCATATGCTTAA

>Chinchilla_lanigera_intact_V1R1_1

ATGACTGCTGGTGATTTCATCTTGAAGCTACTTTTCCCAATCCAGACTGGGATTGGGGTGGTGGGGAATACCCTTCTTCTCTCAGCCTATGCTCCCACATCCTGTACTGCCCATGCACCGAGGCCCACACACCTGATTCTCACCCACATGGCTGTGGCCAACTTCTTGGTTCTTCTCTTCAAGGGGATTCCCCATATGATGTTAATCTGGGGAATAACACCCATCCTGGGAAACACGGGTTGCAAACTTGTGTATTATATCCACAGAGTGGCCCGGGGCCTTTCTCTCTGCACCACTTGCCTCCTGAGCAACTTTCAGGCCATCACCATCAGTCCCAGGCCTGGAGGGATGATAGGCCTTAAAGACCGAGCTTGGAAGAATATCAGTTTCTCCTGTATTCTTTGTTGGATCTTCAACTTGCTGATGAATACTTTTATACCTATAAATATAGAGGGCCTTCAACATAGTCACAATTCTACCATGGTACGGGATTATGGTCTATGTTCTTCCAGAAATTCTGAAACATCTACTCCAAAATATGCATTTCTAATGACACTGGCAGATGTTTTGTTCCTGGGACTCATGGCTTGGGCCAGTGCGTACATGGTGCTTCTCCTATATAGACATCAGCAGACAGTGAAGCATATTCACACCACCCACAACTCCCACAGGTTCTCCCCTGAGGCGAAAGCCACTCAAACCATCCTGCTTCTAGCGAGCACCTTCATTTTGTTTTATTTAATCAATTCTTTCCTTGCCATTTATAATACTGTCTCTTTTAAACCTCACCTCTGGCTGCAGCATACAACAATGTTTCTGGCAGCCTGTTATCCCACCATCAGCCCCTTGATACTGACGCTTCAACATCCTCAAGCACCAAATTGCTGCTCTTAA

>Chinchilla_lanigera_intact_V1R1_2

ATGTGCTCTGCCCAACTGAATTTGGGGATTATCTTCCTTACGCAGACAGGACTTGGCCTGACGGGCAACTTCTCCCTGCTCTGTCTGTATAACTTCATTTTGCTTACTGGACATCACTTGAGACCTATAGATTTGATTCTTAATCAACTGGTTTTGACCAATTTGGTAGTTCTTTTCTCCAAAGGTGTCCCTCAGACATTGGCAGCTATGGGACTGGAACATTTCCTGGATGATGCTGGGTGTAAACTTGTCTTCTACTTTTACAGAGTGAGCACTGGGGTGTCCTTCAGCACTTTCTGCCTCTTCAGTGGCTTCCAGGCCATTAAGCTCAACCCCAGTATTTGTAGGTGGATGGAGCGCAAGATCAGATCCCTAAGGTTTACTGGCTTCTGCTGTTTCCTGTGCTGGATCCCACATCTCTCAATAAATTCATTTCTTCCTTTAATAGTGAATGGTCCATTGAGTAAGAAAAATCTCAGCATAGAAAATAATTATGACTATTGTTCTGGGATAATGCCACAAGGATATATCTCATTATATACAATCTTGTATTTTTTCCCTAACTTAATGAGTCTCATCTTCATGACCTGGGCCAGCGGCTCGATGGTCCTGGTCCTGCACAGACACAGGCAGCAGGTCCAACATATCTACAGCCACAGCCTCTCCCCCAGACCTTCCCACGAAGCCAGAGCCACACGCACCATCCTGATCCTGGTGAGCTCCTTTGTCACCTTTTATTCAGTCTATATTATTTTGACCATCTGGATGACTCTAATCACAAATCAAGGCCAATGGACACTGAATAGCTCCATGCTTGTGGCCTCATGTTTCCCAGCATTCAGCCCCTTTGTGCTCATCGTCAGTGACTCCAGGATCTCACAGTGCCGGATCGCCTGCAGAGAGCGGGCAAAAGTTTTTTCCTAA

>Chinchilla_lanigera_intact_V1R1_3

ATGGATGGAATAGCTTTTGCTAATTTAAATTGGGCTATGGTCTTCCTCACTCAGACTAGTGTTGGAATCCTTGGAAATTCTTTACTCTTTTGCCTGTATAACTTTTCTCTATTCACTGCACAAGTGTTCAGACTCATAGATGCGATTCTCATACAGCTATTCCTAGCTAACAACTTGGTACTGTTCTCTAAAGGGATTCCTCAGACAATGGCTGCTTTTGGATTGAAAGACTTCCTGGGTGAGACTGGATGTAAGCTTGTCTTCTACTTGCACAGAGTGGCCAGAGGTGTGACCCTCAGCACCACTTGCCTACTCAGTGGCTTCCAAGCCATTACAATTTGCTCCACATTTTCAGGGTTGTCAGAGACCAAAATCAGGTCTCCAAAGTATCTTGGCTTCTGCTGTTTTCTCTGCTGGATCCTGAACCTCCTGATAAATACGCATACTATTATGAATGTAACGGGACCAAAGAACAGCAGGAACATGAGTAGACAAAACATGTACAGATACTGCTCTGCTCCTGTCACTAACAGTTTGATATTCACGCTACTAGCAACCATTTACGTCCTTACAGATTGGACTTTCTTGGGTGTCATGGTCTGGGCCAGTGGTTCCATGGTCCTTCTGCTGCACAGACACAAACAGCGTGTCCAGCACATTCACAGCCCAAGTCTCTCCCCCAGAACTTCCCACGAGGCCAGAGCCAGGTGCACTGTGCTGATCCTGGTGAGCATGTTTGTCTCCTTTTATGCTCTGGCTGGCATTTTATCCTTTTGGATTACCCAGACCCCAAGCCCAAGCCCCTGGCTCCTGAGCACCTCTGCACTGCTGTCGTCGGGCTTCCCCACGTTCAGCCCCTTTGTGTTTCTCTTCAGTGACACTCATGTCTCTCAATTCTGTGTTCTGTCATGGACAAGAAAAACAAATATTCCACCACTGTTCTCTGGGCTTTGA

>Chinchilla_lanigera_intact_V1R1_4

ATGAGTTCTGCTAATGTGGACATGGGGATTATCTTCCTCATTCAGACAAGCACTGGTATCCTGGGAAACTCTACGCTCCTTTGCTTTTATAGCTTCCTTCTCTTTTCTGGACACACGGTGAGACCCACAGACTTGATTCTCGGTCAACTGGTTTTAGCCAACTCCATGGTTCTTTTCACCAGAGGAGTCCCTCAGACAGTGGCGTGCTTTGGATGGAAACCTTTCCTGGATGATGTTGGCTGTAAACTCGCCTTCTATCTTCACAGAGTGGGCAGGGGAGTTTCCCTCAGCACCACCTGTCTCCTGAGTGTCTTCCAGGCCTTGAAACTCTGCACTAATTATTTTCAGAGCTTTGGAATCCAAATACAATCCCCAAGGTGCATCAGTTTCTGCTGTGTCCTTTTGTGGGTTCTCCATCTCTTCATAAATATAATAGTTCCCTTGAACATGACTCAGTTAGTCGACAGCAAAAATGTAAGTGTGATGAGAAAAAACTTTGGCTATTGCTCCGCCCCCGTTCTGGATAAACTTACAGCTGGACTTCACGCAGTCTTGTTCTCTTTATTCGACGTGATGACTTTGGGTATCATGTGTTGGGCCAGCGGCTCCATGGTCCTGGCCCTGCACAGGCACAGGCAGCGGGTCCAACACATCCACAGCCACAGCCTCTCCTCCAGACCTTCTCATGAGGCCAGGGCCACACGCACCATCCTGATCCTGGTGAGCTCCTTCATCTTGTTTAATTCTATTGCCTCCATTTTCACCTTTTGTTTCTCTTTTGTGAATCCAGGCCACCGGGTGGTGCAAACCTCTTTGTTAGCAAGCTTATGCTTCCCAACACTTAGCTCTTTCCTGCTCATCAGCACCGACTCCCGAATCTCACAGTTCTGCTTCTCCTGCTGGCAAAGACAGCAGTACATTTGCTAA

>Chinchilla_lanigera_intact_V1R1_5

ATGACTGCTGGTGATTTCATCTTGAAGCTACTTTTCCTGGCCCAGACTGGGGTTGGGGTGGCAGGGAATACCCTTCTTCTCTCAGTCTATGCCCCTACATCCTCTACTGCCCATGCACCGAGGCCCACGCACCTGATTCTCACCCACATGGCTGTGGCCAACTTCTTGGTTCTTTTCTTCAAGGGGATTCCCCACACGATGTTAATCTGGGGAATAACACCCATCCTGGGAAACACGGGATGCAGACTTGTGTACTATATCCACAGAGTGGCCCGGGGCCTTTGTCTCTGCACCACTTGCCTCCTGAGCAACTTCCAGGCCATCACCATCAGCCCGAGATCTGCAAGGAGGATGGGGCTCAAAGACCGAGCTCATAAGGACGTCAGTTTCTCCTGTGCTCTGTGTTGGATCCTGAACTTGCTGATAAATACCTTCATTCCTGTGCATATTGAGGGTCTTCAGCATATTCACAATTCTACCAAGATACAAAGCTATGGACTATGCTCTTCTAAAAGTCCTAGAACAACTGATGCAAAATTACCAATTATCCTGACATTGCCAGATGTTGTGTTCCTAGTACTCATGGCCCGGGCAAGTGTCTTCATGGTACTTCTCCTATACAGACACCGCCAGCAAGTGAAACATATTCGAACTCAAGGGAATTTCCACAAAGTTTCCCCGGAGGCAAAAGCCACTCAAACCATCCTGCTTCTAGCGAGCACCTTCATTTTATTTTACTCCAGCAATTCCATCCTCACAATTTATAATGTTGTTGTTTTAAAATTTTACCTCTGGCTGCAGCACACTATTAGCTTTCTAGCAGCCTGCTATCCCACACTTGGCCCCCTGATACTGATGCTTCAAGATCCTCAAGCACCAACTTGCTGCTCTTAA

>Chinchilla_lanigera_intact_V1R1_6

ATGGTTGCAGGAAACTTAGACTTGGGGGTGATCTTCCTTGCTCAGACTGGGATTGGCATCTTTGGAAATTCTTTCCTCCTTTGTTTTTACAGCTTCTGTCTGCTCACTGGATACAAGTTGAGACACACAGACCTAATCCTCAACCAACTGGTCTTAGCCAACTCCTTGGTCCTGCTCTCCAAAGGGATATCGCAAACAATAGTGGCTTTTGGGTGGAAGTATTTCCTGGACAGTGTGGGATGTAAACTTGTCTTCTATTTTCACAGAGTAGGCACAGGGGTTTCCTTCAGCACTGTCTGCCTGCTCAATGGCTTCCAGGCCATTAAGCTCAACCCCAGTATTTGTAGATGGATGGAGCTCAAGATTAGATCCCTAAGGTTCATTGGCTTCTGCTGTTTCCTGTGCTGGATTCAGAATCTCTCAATAAATTCATCTATTGTTATAGTAGTGAATGGTCCATTGAGTTGCAAAAACATTACTGTGAGAGCCAGTTGTGGACACTGTTCCTGGACAATGATAGAGAGATATAGCTCATTATATGCATTCTTATATTTCTCCCTTGACTTCATCAGTGTGGGCTTCATGATATGGGCCAGCGGCTCCATGGTCCTGGCCCTGCACAGGCACAGGCAGCGGGTCCAACACATCCACAGCCACAGCCTCTCCTCCAGACCTTCTCATGAGGCCAGGGCCACACGCACCATCCTGATCCTGGTGAGCTCCTTTGTTACCTTTTACTCAGTCTATATTATTTTGACCATCTGGTCAACTCTAGTTTCAAATCGTGGCCAGTGGACAGTGAACAGCTCCGTGCTTGTGGCTTCATGTTTCCCAGCATTCAGCCCCTTTGTGCTCATCATCAGTGACTCCAGAATCTCTCAGTTCTTCATCACCTGCAGGAGAGGACAACTCGTTCTTCCTAAGTAG

>Chinchilla_lanigera_intact_V1R1_7

ATGGATGGAATAGTTTTTGCTAACTTAAATTGGGCTATGGTCTTCCTCACTCAGACTAGTATTGGAATCCTTGGAAATTCGTTACTCTTTTGCCTGTATAACTTTTCTCTATTCACTGCACAAGTGTTCAGACTCATAGATGCGATTCTCATACAGCTATTCCTAGCTAACAACTTGGTGCTGTTCTCTAAAGGGATTCCTCAGACAATGGCTGCTTTTGGATTGAAAGACTTCCTGGGTGAGACTGGATGTAAGCTTGTCTTCTACTTGCACAGAGTGGCCAGAGGTGTGACCCTCAGCACCACTTGCCTACTCAGTGGCTTCCAAGCCATTACAATTTGCTCCACATTTTCAGGGTTGTCAGAGACCAAAATCAGGTCTCCAAAGTATCTTGGCTTCTGCTGTTTTCTCTGCTGGATCCTGAACCTCCTGATAAATACGCGTACTATTATGAATGTAATGGGACCAAAGAACAGCAGGAACATGAGTAGACAAAGCATGTACAGATACTGCTCTGCTCCTGTCACTAACGGTTTGATATTCACGCTACTAGCAACCATTTACATCCTTACAGATTGGACTTTCTTGGGTGTCATGGTCTGGGCCAGTGGTTCCATGGTCCTTCTGCTGCACAGACACAAACAGCGTGTCCAGCACATTCACAGCCCAAGTCTCTCCCCCAGAACTTCCCACGAGGCCAGAGCCAGGTGCACTGTGCTGATCCTGGTGAGCATATTTGTCTCCTTTTATGCTCTGGCTGGCATTTTATCCTTTTGGATTACCCAGACCCCGAGCCCAAGCCCCTGGCTCCTGAGCACCTCTGCGCTGCTGTCGTCAGGCTTCCCCACGTTCAGCCCCTTTGTGTTTCTCTTCAGTGACACTCATGTTTCTCAATTCTGTGTTCTGTCATGGACAAGAAAAACAAATATTCCACCACTGTTCTCTGGGCTTTGA

>Chinchilla_lanigera_intact_V1R2_1

ATGACAATGATAGATCTGGAATTTGTAATCCTTTTCTTTTTCCCCATTGTCATTGGAAGCCTGGGAAATGTTTCACTTTTATGTCATTCTATATTCTGTTACTTCAGTGGATGCAGGTCCAGGTTCACAGATGTGATGCTCAGGCACCTGACTGTGGCCAACTCCTTGTATATTCTCTCCAGAGGAATCCCAGAGACCATGGCAGCTCTTGGGATGGAAAACGTCCTTAATAATGTTGAATGCAAACTTGTTTTCTATGTTCACAGGGTGTCTAGAGGTGTGTCTTTCAGCACCACCTGCCCGCTGAGTGTCTTCCAGGCCATCACCATCAGCCCCAGGAGCTCCAGGTGGGCAGAGTGGAAAGTGAAAGCCCTAAAGTGCATTGGGCCCTGTCCCATCATTTGCTGGGTCCTGCACATGCTGCTAAACATCAGAGTTCCCATGCTTGTGAGTGACAGAAAGAACAACAAAAACATCACAAACACTATAGATTTTCAGTTCTGTTCAGCTATGAGTCCAGACAAAGACAAACACTCCATTTTTGTGGCATTGACATTGTCATATGACATTTTGTGTTCAACACTTATGATCTGGAGCAGTGGCTCCATGGTTTTCATGCTGTACAGGCACAAGCAGCAAACAGAGCATATTCACAGATACAACATCTCATCAAGATCTTCTGCTGAGACCAGAGCTTCTCAAAGCATCCCCATCCTGGTCTGTGCCTTTGTGTCCATCTATGCCCTGTCTTCCATCATGTACGTTTGGTTTTCTCTTTATAACACAGCAGTTTGGTGGCTGGTTAAAACTGCTGCCTTAACCGATGTGTGTTTTTCTGCTGCTAGTCCCTTCATTCTTATGACTCGTGAGCAGTGTGTGAGCAGGCCCCTGTGGAAGAGGTGA

>Chinchilla_lanigera_intact_V1R2_10

ATGACATCTATGGATCTGAAATTTGTAATCCTCTTAATCTTCCCAATTGTTATTGGAAGCCTGGGTAATTTCTCCCTTTTATGTCATTTTATCTTCCTTTACTTCAGTGGATGCAGCACCAGATCCACAGATTTGATTCTCAGGCACCTGACAGTGGCCAACCTCTTGGTCATTCTCTCCAGAGGAATCCCAGAGACCATGGCAGCAGCTGGGGTGGAAGACTTCCTCAGTAATGTTGGATGCAAACTAGTTTTCTACGTTCTAATAGTGGGCAAGGGTGTGTCTTTCAGCGCCACCTGCCTCCTGAGTGTCTGCCAGGCCATCACCATCAGCCCCAGGAGCTCCAGGTTGGCAGAGCTGAAAGTGAAAGCCCTAAAGTACATTGGGCCCTGTTGCATCCTTTTGTGGCTGCTGCACATGTTGTTAAACTTCAGAGTTCCTATGCTTGTGAGTGACAAATGGAGCAGTAGAAACATGGCAAACATTATAGATTATCAATACTGTTCAGCTATAATTTCTGGCAAAGGTAATACCTGGTTTTTTACAGTATTGTCACTGTCACACGATATTTTGTGTTTGAAACTTATGATCTGGAGCAGTGGCTCCATGGTTTTCATGCTGTATAGGCACAAGCAGCGAACACAGCATATTCACAGACACAAAATCTCATCAAGATCTTCTGCTGAGACCAGAGCTTCTCAAAGCATCCTCATCCTGGTCTGTGCCTTTGTATCCTTTTATACCCTGACTTCCTTCACATATTTTTTGTTTTCTGTTTATGACAAAAGTGCTTGGTGGTTGGTTAACACTTCTGCCTTAACCATTGTTTGTTTCCCGACTGCTAGTCCCTTCATTCTCATGACCCGTGAACAGTGTGTATGCAGGCCCCTGTGGAGGAAGTGA

>Chinchilla_lanigera_intact_V1R2_11

ATGATGGATCTGGAATTTGTCATCCCTTTCTTTTTCCCCATTGTCATTGGAAGCCTGGGAAATGTCTCACTTTTATGTCATTCTATCTTCCGTTACTTCAGTGGATGCAGGTCCGGGTTCACAGATGTGATTCTCAGGTACCTGACTGTGGCCAACTCCTTGTGCATTGTTTGCAGAGGAATCCCAGAGACCATGGCGGCACTTGGGATGGAAAACATCCTTAATAATGTTGGAGGCAAACTTGTTTTCTATGTTCACAGGGTGGCTAGAGGTGTGTCTTTCAGCACCACCTGCCTGCTGAGTGTCTTCCAGGCCATCACCATCAGCCCCAGGAGCTCCAGGTGGGCAGAGCTGAAAGTGAAAGCCCTAAAGTGCATTAGGCCCTGTCCCATCATTTTCTGGATCCTGCACATGCTGCTGAACATCAGAGTTCCTGTGCTTGTGAGTGACAAAAGGAACAACAAAAACATCACAGACACTATAGATTTTCGGTACTCTTCAGCTATGAGTCCTGACAAAGACAGACACTCCATTTTTGTGGCATTGACATTATCACATGACATTTTGTGTTTGAAAGCTATGATCTGGAGCAGTGGCTCCATGGTTTTCATTCTGTACAGGCACAAGCAGCGAACTCAGCATATTCATGGACACAAAATCCCATCAAGATCTTCCCCTGAGACTAGAGCTTCTCAAAGCATCCTCATCCTGGTCTGTGCCTTTGTATCCATCTATGCCCTGTCTTCCATCATGTATGTTTGGTTTTCTTTTTATGATAGAGCAGCCTGGTGGCTGATTAAAACTTCCGCCTTAATTGATGTTTGTTTCCCTGCTGCTAGTCCCTTCATTCTCATGACTTGTGAACAGTGGGCATGCAGGCCCAAGTGGAAGAAGTGA

>Chinchilla_lanigera_intact_V1R2_12

ATGTCATCTATGGATCTGAAACTCCTCACCCTCTTCCTCTTTCTCATTGTCATTGGAAGCCTAGGAAATCTCTCCCTTTTACGGCATTCTATCTGCCTTTATTTCAGTGGATGCAGGTTGAAGTTCACAGATGTGATTCTCAGGCACCTGACAGTGGCCAACCTCTTGGTCATTCTCTCCAGAGGAATCCCAGAAATCATGGCAGCTTTTGGGGTGGAAGACTTTCTCAATAGTATTGGATGCAAACTGGTTTTCTATGTTCAAACAGTGGGCAGGGGTGTGTCTTTCAGCACCACCTGTCTGCTGAGTGTCTTCCAGGCCATCACCATCAGCCCCAGGAGCTCCAGGTGGGCAGAGCTGAAAGGGAAAGCCCTAAAGTTCATTAGACCCTATCCAAATATTTTCTGGGCACTGTACATGCTGGTAAACATCAGAGTTCCTATGCTTGTGAGTGACAAAAGGAGCAAGACAAACATCACTAACACTATAGATTACCAGTACTGTTCAGCTATGATTTCTAGCAAAGACAAAAACTCCATTTATGTGGCACTCAGATTATCACATGACATTCTGTGTTTGAAACTTATGATCTGGAGCAGTGGCTCCATGGTTTTCATGCTGTACAGGCACAAGCAGCAAATGCAACATATTGACCGACCCAAAATCTCATCAAGGTCTTCCCCTGAGGTCAGAGTTTCTCAAAGAATCCTCATCCTGGTCTGTGCCTTTGTGTCCTTCTATGCTCTGTCCTCCATCATGTACGTTTGGTTCTCTTTTTATGACAAAGGTGCTTGGTGGCTGGTTAAAACTTCTGCCTTGATACATGTTTGTTTCCCGACTGCTAGTCCCTTCATTCTCATCACTCGAGAACAGTGTGTACACAGGCCCATGTGGAAGAGGTGA

>Chinchilla_lanigera_intact_V1R2_13

ATGTTGATACTAATCCTGCACAATAGAAGAATGACTACTATGGATCTGAAATTTGTGATCCTTTTCCTTTTCCCCATTGTCATTGGAAGCCTGGGAAATTTCTCCCTTTTATGGCATTCTATCTTTCTTTATTTCAGTGGATGTAGGTCCAGGTCCACAGATTTGATTCTTAGGCACCTGACAATGGCCAACCTCTTGGTCATTCTCTCCAGAGGAATCCCAGAGGCCATGGCAGCGCTTGGGAAGGAAGACTTCCTCAATAATGTTGGATGCAAACTTGTTTTCTATGTTCAAATGGTGGGCAAGGGTGTGTCTTTCAGCACCACCTGCCTGCTGAGTGTCTTCCAGGCCATCACCATCAATCCCAGGAGCTCCAAATGGGCAGTGCTGAAAGTGAAATCCCTAAAGTGCATTGGGCCCTGTCCCATCATTTTCTGGGTCCTGCACATGCTGCTAAACTTCAGAGTTCCTATGCTTGTAAGTGACAAAAGGAAGAACACAAACATCACAAACACTATAGATTATCAGTACTGTTCAGCTATACATTCTAGCAAAGAGAAAAACAATATTTATATGGCATTGACATTATCACATGACATTTTGTGTTTGAAACTTATGATCTTTAGCAGTGGCTCTATGATTTTCATTCTGTACAGGCACAAGCAGCAAACTCAGCATATTTACAGACACAAAATCCCATCAAGATCTTCCCCTGAGACCAGAGTTTCCCAAAAAATCCTCATCCTGGTCTGTGCCTTTGTATCTTTTTATGTCCTGACTTCCATCATGAATGTTTGGCTGTCTCTTCATGACAAAACTGCTTGGTGGCTGATTAAAACTTCTGCCTTAATTCACATCTGTTTCCCTACTGCTAGTCCCTTCATTCTCATGACTCGTGAACAGTGTGTATGCAGGCCCATGTGGAAGAAGTGA

>Chinchilla_lanigera_intact_V1R2_14

ATGTCAGCTATGGATCTGAAATTTGTAATCTTCTTACTTTTCCCAATTGTCTTTGGAAGTCTGGGAAATGTTTCACTTTTATGTCAGTCTGTCTTCCTTTACTTCAGTGGATGCAGGTCCAGGTCTACAGATGTGATTCTCAGGCACCTGACTGTGGCCAACTTCTTGTACATTCTGTGCAGGGGAATCCCAGAGACCATGGCAGCTCTTGGGATGGAAGACTTCCTCAATAATGTTGGATGCAAACTAGTTTTCTATCTTCAAGCAGTAGGAAAGGGTATGTCCTTCAGCACCACCTGCCTGCTGAGTGTCTTCCAGGCCATCACCATCAGCCCCAGGAGCTCCAGGTGGGCAGAGCTGAAAGTGAAAGCCCTAAACTGCATCGGACCCTGTACCATCATTTGCTGGGTCCTGCACATGCTGCTAAACATCAGAATTCCTATATTGGTGAGTGACAAAAAGAGCAACAAAAACGTCACAAACATTATAGATTTTCAGTACTGTTTAATTATGAGTCCTGACAAAGACACAAACTCCATTTTTGCAGCCTTGTCATTATCACACGACATTTTGTGTTTGAAACTTATGATCTGGAGCAGTGGCTCCATGGTTTTCATTCTGTACAGGCACAAGCACCAAACTCAGCATATTCACAGACACAACATCTCACCAAGATCTTCCGCTGAGACAAGAGCTTCTCAAAGCATCCTCACGCTGGTCTGTACCTTTGTGTCCTTCTATGCCCTGTCTTCAGTCATTTATGTCTGGTTTTCTCTTTATGACAGAGCTGCTTGGTGGCTGGTTAAAACTTCTACCTTAACCAGTGCTTGTTTCCCTGCTGCTAGTCCCTTCATTCTCATGGCCCGTGAACAGTGTGCGTGCAGGCCCCTGTGGAGGAAGTGA

>Chinchilla_lanigera_intact_V1R2_15

ATGGCATCTATGGATCTGAAATTTGTAATCCTCTTCCTTTTTCCCATTGTCATTGGAAGCCTGGGAAATTTCTTCCATTTTTGGCATTCTACCTGCCTTTATTTCAGTGGATGCAGGTCCAGATTCATAGACTTCATTCTCAGGCACTTGTCAGTGGCCAACCTCTTGGTCATTCTCTCCAGAGGAATCCCAGAGACGATGGCAGCACTTGGGATGGAAGAATTCCTTAATAATGTGGGATGCAAACTAGTTTTCTATGTTCAAATGCTGGGCAAGGGTGTGTCTTTCAGTAACACCTGTCTGCTGAGTGTCTTCCAGGCCATCACCATCAGCCCCAGGAGCTCCAGGTGGGCAGAGCTGAAAGTGAAAGCCCTAAAGTGCATCGGGCCCTGTCCCATCATTTGCTGGGTCCTACATATGCTGCTAAATATCAGAGTTCCTATGCTTGTGAGTGACAAAAGGAACAACAAAAACATCACAAACACTATAGATTATCAATACTGCACGGCTATGAGTCCTGACAAAGACAAAAACTCCATTTTCGTGGCATTGACCTTATCACATGACATTTTGTGTTTGAAACTTATGATCTGGAGCAGTGGCTCCATGGCTTTCATTCTGTACAGGCACAAGCAGCGAACTCAGCATATTCACAGGCACATCTCATCAAGATCTTCCCCTGAGACAAGAGCTTCTCAAAGCATCCTCATGCTGGTCTGTACCTTTGTATCCTTATATGCACTGACTTCCATCATGAATGTTTGGTTCTCTTTTTATAGCAAACCTGCTTGGTGGCTGGTTAAAACTTCTGCCTTAATTCACGTTTGTTTCCCGACCATTAGTCCATTTATTCTCATGACTCGAGAACAGCTTGTATGCAGGCTCATGTGGAAGAAGTGA

>Chinchilla_lanigera_intact_V1R2_16

ATGACATCTATGGATCTGAAATTTGTAATCCTCTTCCTTTTTCCCATTGTCATAGGAAGCCTGGGAAATTTCTCCCTTTTATGGCATTCTATCTGCCTTTATTTCAGTGGGTGCAGGTTTAGGTCCACAGACTTTATTCTCAGGCACCTGACAGTGGCCAACCTCTTGGTCATTCTCTCCAGAGGAATCCCAGAGACCATGGCAGCACTTGGGATAGAAGACTTCCTCAATAATGTGGGATGCAAAATGGTTTTCTATCTTCAAGCAGTAGGAAAGGGTGTGTCTTTCAGCACCACCTGCCTGCTGAGTGTCTTCCAGGCCATCACCATCAGCCCCAGGAGCTCCAGGTGGGCAGAGCTGAAAGTGAAAGCCCTAAAGTGCATTGGGCCCTGTCCTGTCATTTGCTGGGTCCTGCACATGCTGGTAAACATTAGAGTTCCTATGCTTGTGAGTGACAAAATGAACAATACAAACATCATAAACACTATAGATTACCAGTATTGTTCAGCTATGATTTCTAGCAAAGACAAAATCTCCATTTTTGCAGCATTGAGATTATCACATGACATTTTGTGTTTGAAACTTATGATCTGGAGCAGTGGCTCCATGGTTTTCGTTCTGTACAGGCACAAGCAGCGAACTCTGCATATTCACAGGCACAAAATCCCGTCAAGATCTTCCGCTGAGACAAGAGCTTCTCAAAGCATCCTCACGTTGGTCTGTGTGTTTGTATCCTTCTATGTCCTGTCTTCCATCTTGAATGTTTGGTTTTCTCTTTATTATGAAGCTTCTTGGTGGCTTGTTAAAATTTCTGCCTTAATCTATGCTTGTTTTCCAACTCTTAGTCCCTTCCTTCTCATGACTCATGAACAGTGTGTATGCAGGTTCATGTGGAAGAAGTAA

>Chinchilla_lanigera_intact_V1R2_2

ATGGCCTCTGGAGCTGTTTATATTGTAATAATTTTCCTTGTTCAGATTTTCATTGGAACCCTGGGGAACCTCTCTCTCTTAGGTAAATATTTATTTCTTTTCCTCACTGGATATAAGACAAGATCCATAGATTTGATTGTTATGCATCTGACTGTGGCCAACTTCTTGGTGATTCTCTCTAGAGGGATCCCAGAGACCATGGCAGCTTATGGGTTGAAAGGCTTCCTCAGTGATTTTGGGTGCAAATTTGTTTTCTATGTTAGCAGAGTGAGTAGGGGTGTAGTCTTTGGCACCACCTGCCTCCTGGGTGTCTTCCAGGCCGTCATCATCAGCCCCAGCAACTCTAGGTGGGCAGAGTTGAAGATGAAGTTTCCCCAGTATATAAGCACCTTCACTGTCCTCTGCTGGGTCCTGCACTTGGTGTTGAACATTAGTTTCTCTGTGAGTTTGACTAGCAGTTCGAAGAAGAAAAACCTCACAAATAAAATAGACTTTGCTTACTGTTCAAATCCACATACCATGAAAGGGATATACGCAGGATATGCAGTGTATACCACCATCATTGATGTTTTGTGCATGGGGCTAATGCTCTCAGCCTGTGGCTCTATGCTTTTCATCTTGTGTATGCACAAGCGGCGGGTCCAGCACATGCGTAGGACCAGCAACTCCTTCAGATCCTCTCCTGAGACCAGAGCCATTCACAGCATCCTTCTCCTGGTGAGCATCTTTGAATTTTTCTATTTCCTGTCTTGTATCATACAAATATATGTGACTTTTTTTAGTCATTCAAGAATCTTACTGTTCACTCTTGGTGCTTTCATAAATGCATGTTTCTCAACTGTATATCCCTTTGTATTCCTAATTCATGGTCATTATGTATCTGGAATTTGCTGCATCTAG

>Chinchilla_lanigera_intact_V1R2_3

ATGGATTTGAAATTTGGGATAATATTTCTTTTCCAGATTGTCATTGGAACCATGGGGAATTTCTCACTCTTATATAATTATATGTTTCTCCACTTCAGTGGATACAGGCCAAGGCCAACAGATTTATTTCTCAGACACCTGATTGTGGCCAATTCCTTAGTCATTCTCTCCAGAGGGATGCCAGAGACCATGGAAGTTTTTGGGAAGGAGCACTTCCTTGATGATCTTGGATGCAAATTTGTTTTCTATCTTCACAGGGTGGGCAGGGGTGTGTCCATTGACACCACCTGCCTATTGAGTGTCTTCCAGGCCATCACCCTCAGCCCAGGGAACTCCATCTGGGCACACCTGAAAATGAAAGCCCTGAAATACAAGGGTCCATCTATCATCCTCTGCTGGGTGCTGCACCTGCTGTTAACTATTAGACTTCCTGTTCTTATCACTGATAAAAGAAACAAGAAAAACTTCTCAAAAACTATAGATTTTCAACATTGTTCAGTGATGCTCCCTGAGAATAACACAGGCACAGTTTTTGCAGCAATGACATTATCCCATGACATTTTGTGTTTAAATCTCATGATCTGGGCCAGTGGTTCCATGGTTCTTATTCTGTACCGGCACAAGCAGCGGGTCCAGCATATCCACAGACAAAGCTCATCTAGATCTTCCCCTGAGACCAGAGCTTCTCAAAACATCCTTGCTCTGGTGAGTGCCTTTGTGTTCTTCTACACCCTGTCCTCCACCCTTCATGCATGCTTTACTCTTGTTGATAAAAGAGCTTTGTGGCTAATCAGCAGCACTCCCATAATCAGTGCTGGTTTCCCTACTATCAGCCCCTTTATTCTCATGAGTCATGAATGCTCTGTGTCCAGGCTCTTCCGGAAGAAGTGA

>Chinchilla_lanigera_intact_V1R2_4

ATGACATCTTTGGATCTAAAATTTGTAATCCTCTTCCTTTTCCCAGTTGTCACTGGAAGCCTGGGAAATGTCTCACTCTTATGTCGTTTTATCTTCCTTTACTTCAGTGGATGTCAGTCCAGGGTCACAGATGTGATTCTCAGGCACCTGACTGTGGCCAACTCCTTGTATATTCTCTCCAGAGGAATCCCAGAGACCATGGCAGCTCTTGGGATGGAAGACTTCCTCAATAATATTGGATGCAAGCTGGTTTTCTATCTTCAAGCAGTAGGAAGGGGTGTGTCTTTCAGCACCACCTGCCTGCTGAGTGTCTTCCAGGCCATCACCATCAGCCCCAGGAGCTCCAGGTGGGCAGAGCTGAAAGTGAAAGCCCTAAAGTGCATTGGTCCCTGTCCCGCCATTTGCTGGGTCCTGCACATGCTGCTAAACATCAGAGTTCCCATGCATGTGAGTGACAAAAGGAACAACAAAAACATCACAAACACTATAGATTTTCAGTACTGTTCAGCTATGAATTCTGCCAAAGTTGAAAACTCCATTTTTGCAGCACTGTCATTATCACACAACATTTTGTGTTTGAAACTTATGATCTGGAGCAGTGGCTCCATGGTTTTCATTCTGTACAGGCACAAGCAGCAAACTCAGCACATTCACAGACACATCTCATCAAGATCTTCCGCTGAGACCAGAGCTTCTCAAAGCATCCTCCTCCTGGTCTGTACCTTTGTGTCTGTCTATGCCCTGTCTTCTATCATGTATGTCTGGTTTACTCTTTATGACAAAACTGCTTGGTGGCTGGTTAAAACCTCTGCCTTAACCAATGCTTGTTTCCCTGTTGCTAGTCCCTTCATTCTCATGACTCGTGAGCAGATTGTGTGCAGGCCCCTGTGGAGGAAGTGA

>Chinchilla_lanigera_intact_V1R2_5

ATGGCCTCCAGGGTTGTGTATATTTTAATAATCTTCCTTGTTCAGATTTTCATTGGAACACTGGGGAATCTCTCTCTCTTAGGTCAATATTTATTTCTTTACCTCAGTAGATATAAGACAAGATCAATAGATTTGATTATTATGCATCTGATTGTGGCCAACTTCTTGGTCATTCTCTCTAGAGGGATCCCAGAGACCATGGCAGCTTATGGGTTGGAAGACTTCCTCAGTGATTTTGGATGCAAATTTGTTTTCTATGTTAGCAGGGTGGGTAGGGGTGTAGCTTTTGGCAGCACCTGCCTCCTGAGTGTCTTCCAGGCCATCACCATCAGCCCCAGCAGCTCCAGGTGGGCAGAACTGAAAGTGAAATTTCCCAAGTATATAAGCACCTTCACTGTCCTCTGCTGGGTCCTGCACTTGGTGCTGAACATTAGTTTCTCTGTGAGCTTGACTAGCAGATTGAAAAACAAAAACATCACAAATAAAATAGACTTTGCTTACTGTTCAGATCCAAGAACCACGAAAGGGATATACACTGCACATGCAGTGTATATCGCCATCTTTGATGTTTTGTGCATGGGTCTAATGCTCTCAGCCTGTGGCTCTATGCTTTTCATCTTGTTTATGCACAAGCGACGGGTCCAATACATGCATAGGACCAGCAACTTCTTCAGATCCTCTCCTGAGACCAGAGCCACTCAAAGCATCCTTATCCTGGTAATCATCTTTGTATTTTTCTATTTCCTGTCTTGTATCATGCAAGTATATGTGACGTTTTTTAGTCATTCAAGAATCTTTCTGTTCACTCTTGATGCTTTTCTAAGTGCATGTTTCTCTACTGTTTGTCCCTTTGTATTCATGATTTGTGGCCACTATGTAAATGGAATCTGCTGTGTCTACTCTAAAAGGAACACAGTCATCTAA

>Chinchilla_lanigera_intact_V1R2_6

ATGGTTGCTACCAATGTGCATATTGGAGCAGCATTCCTTGTCCAGGTCGCCACTGGAACCCTGGGAAATTTATTTCTCTTAAGTCAGTATTTATTTCATTACTTCAGTGGATGCCGGTCAAGGGCCACAGATCTGATCCTTAGGCACCTGACAGTGGCCAATCTCTTGGTGATTCTCTCCAGAGGAATCCCAGAGACCATGGCAGCTTTTGGGTTTGAAGACTTCCTCAGCGATCTTGGATGTAAACTCGTCTTCTACGTGCACAGGGTGGGCAGAGGTGTGTCTATGAGCAGCACCTGCCTCTTGAGTGTCTTCCAAGCCACCACCATCAGTCCCAGGAGCTCCAAGTGGACAGAGCTTAATGTAGAAGCTAAGAAATACACGAGCATCTCCACTGTCCTGTGCTGGCTCCTGCACATGCTGCTGAATGTCACTTTTCCTGCCACTGTGACTGCCAAGTGGAGAAACAAAAACGTTACAAGTAAATTAGACTTTGCATACTGTTCTGGGCCAGGGTATGAGAAAGTTTTGTACTCCATAACCACCGCGCTGTTCTCCATCAGTGATACTTTGTGTATGGGGCTCATGATCTGCACCAGCAGCTCCATGGTTTCCACACTATACAAGCACAAGAAGCAGGTCCAGCACATCTACAAGCTCAATGTTGGCTCCAGGTCCTCTCCTGTGACCAGAGCCACCGACGGCATCCTTGTCCTGGTGTGCACATATGTACATTTTTACACCATCAGCTGCATCATCCAGGTGTATCTGGCTGTGTTTCAGCATCCCAGCACCTTATTACTAACCACTGCTGCCTTGATTAATACATGTTTTCCAACACTTTGTCCCTTTGTGCTCATGAGCCATGACCACAGTCTGTCCAAAATCTTTCGTTTCTATTGTAGAAGGAACACAGTCATCTAA

>Chinchilla_lanigera_intact_V1R2_7

ATGAATTTGATTTTTGGAATGATCTTCCTTTCCCTGCTTGTCATTGGATCCCTGGGGAACTGCACACTGCTACACCATTATGCTTTCCTTTATTTCCGTGGATGTAGATTAAGGTCCACAGATGTGATCCTCAGACAGCTGACCATAGCCAACCTCTTACTCATTCTCTGCAGAGGAATCCTGGAGGTCATGTCAGATTTTGGATTGAAACACTTCCTCAATGATGCTGGATGCAAACTCGCTTTCTATGTTCACAGGGTGGCTAGAGGTGTGTCTCTCAGCACCACCTGCCTGCTGAGTGTCTTCCAGGCCATCACCATCAGCCCCTGGAACTCCAGGTGGGCAGAGCTGAAAGGGAAGGTCCTGAAGCACATTGGTCCCTTTAACATTTTCTGCTGGGTCCTGCACATGTTTCTAAACATCAGAATTCCCATGCTTATGACCGCCCAAAGGAAAAAGGAAAACATGACAAAAGCTATGGATTTTCAGTACTGCTCAACTAGGTCTCATGACAAAGATCCAGGCTTAGTGTTTGCAGCATTAATATTTTCCCATGATATTTTGTGTTTGGAGCTCATGATCTGGGCCAGTGGCTCCATGGTTTTCATTCTGTACAAGCACAAGCAGCGGGTCCACCATATCCAGAGACACAATATCTCCTCTAGATCTTCCCCTGAGACCAGAGCCTCTCAAAGTATTCTTGCCCTAGTGAGTGCCTTTGTATCTTTCTATACTCTGTCTTCTGTTGCTCAAATTTTGTTCTCTCTTTATGGTAATGTTACTTGGTGGCTGATCAAAACATCTGCCTTAATCAATACTTGTTTCCCCACTGTGAGCCCCTTTATTCTCATAAATCGTGAACGTTCTGTATCCTGGCTCACCTCAAAGAAGTGA

>Chinchilla_lanigera_intact_V1R2_8

ATGACCTCCGTGGATTTTACAATTGGAATAATTCTCCTTTTCCAGATAATCTTTGGAATCCTGGGAAATTTCGCACTCTTATACCATTATATTTTCCTTTACTTCAATACTTATAAGGCAAGGTCCATAGATTTGATTCTCAGGCACCTGACAGTGGCCAACTCCTTGTTCATTATCTTCAGAGGAATCCCAGAGACCATGGCAGCTTTTGGGTTGAAACACTTTCTCAGTAGTGTTGGGTGCAAAATCATTTTCTACGTTCACAGGGTGGCTAGAGGTGTGTCTCTCAGCACCACCTGCCTGCTGAGTGTCTTCCAGGCCATCACCATCAGCCCCAGGAGCTCCAGGTGGGCAGAGCTGAAAAGGAAGGCCCTAAAGTGCATTGGTCCCCTTAATGTCTTCTGCTGGGTCCTGCACATGTTCCTAAATATCAGAATTCTCATGCTTATGACCGCCCAAAGGAAAAAGGAAAACATGACAAAAGCTATGGATTTTCAGTACTGCTCAGCTAGGCCTCATGACAATGACCCAGGATCAGTGTTTGCAGTATTAGTATTCTCCAATGACATTTTGTGTTTGGAGCTCATGATCTGGGCCAGTGGTTCCATGGTTTTCATTCTGTACAAGCACAAGCAGTGGGTACATCACATCCACAAGCATAATATCTCATCAAGAGCTTCCCCAGAGACCAGAGCCTGTCGAAGTATTCTTACTCTGGTGAGTGCCTTTGTGTCCTTCTATAGCCTGTCTTGCATTATTCATATTTTATTTTCTCTTTATGATAATACTACCTGGTGGCTCATCAAGACTTCTGCCTTAATAAATTCTTGTTTCCCCACTGCCAGCCCCTTTATTCTAATAAGTCGTGAACACTGTGTAACCAGGCTCATCTGA

>Chinchilla_lanigera_intact_V1R2_9

ATGAGCCTTGCTCAGAGAAATGATTGTCAGCATAATTTAAAGGAAATTTTAGAACGAAATGACAAAATAGCTTCCAGGGATTTGGCATTAGGAATACTTTTTTTGTCACAGACTGTGATTGGAATTCTGGGAAATTTTTCTCTTCTATACCATTATGTTTTCCTTCACCATAGACAAAGCAGATGGAGATCCATAGATTTGATTCTCATGCACTTGTGTATAGCTAATTCTTTAGTTATTCTTTCAAATGGATCTTCCCAGACAATAGTAGCCTTTGGGTCGAAATATTTATTCAATGAATTTGGTTGCCATGTTACCTTGTACGTCCTCAGAGTGGGCAGGACTATGTCAATTTGTACTATATGTCTCTTGAGTGTCTTCCAGACTATTATAATCAGCCCCACGAACTCCTGTTGGAAAAATCTTAAAGTAAAAGCTGCAAAGTACACTGGCTTCTGCATGTCCCTCTGCTGGTTGCTACACATGGGAGTAAATTTCATTTTTCCTCTGTATACACTACATGCATTTGGGAAAGCAGAAGGCAGAAACATCACAAAGGAAAGGCATGTGGGATTCTGTGCTTTTGTAGATTATGGGACAACCACGAGCTACATCTATATTGCAGTAGTAGTATCGCCTGAAGTGGCTTTTATTGTGCTAACGATATGGGCCAGTGGTTCTACTATTTTTGTCCTCTACAGACATAAACAGCAGGTTAAGCACATTCATACCAAGAATGTTTCCTTCAGATCCCCTGAGTTGAGAGCCACCAAAAGCATCCTTCTCCTGGTGAGCACCTTTGTATCATTTTACATCATCTCTTCCATCTTTCACATTTTCGTTGCCCTTTTTTATAGTCTAAGTTGGTGGTTGGTGAATATCTCTCGCCTCATTTCTCTGTGTTTTCCAACTATCAGCCCCTTTCTGGTCATGAGCCAGGATTCCTCTATATCATTGTTCTGGTTTGTCCAGATAAGAAACACAAAAATGCCTCTTCTCATCAGAAATATATAA

>Chinchilla_lanigera_intact_V1R3_1

ATGAGCCCTGTGGATTTGAAATTTGGAGTGGTCTTCCTTGTCCAGGTTGTCCTTGGAACTCTGGGGAATTTAGCACTCTTGTATCATTATTGTATCCTTTACTTCAGTGGATGCAGATCCAGGTCCACAGATGTGATCCTCAGGCACCTGACTATAGCCAACTCCATTGTTATTCTCTCCAGAGGAATCCCAGAGACCATGGCAGCTTCTGGGTTGAAAGACTTCCTCAGTGATGTTGGATGCAAACTCGTTTTCTATGTGCACAGGGTGGGCAGGGGTGTGTCTATCAGCACCACCTGCCTGCTGAGTGTCTTCCAGGCCATCACGATCAGCCCCAGGAGCTCCAGGTGGGCAGAGCTGAAGGTGAAAGCCCTGAAGTGCAGCGGCTTGTCTAACGGCCTCTGCTGGGTCCTGCACCTGCTGCTAAATGTCCGAGTTGCTATGCTTGTGACCGACAGATGGAACAACAGAAACATTACAAAAGCTATTGATTTTCAGTACTGTTCAGGCACCCTGCCTGACAAAAACGCAGGTTACTTGTTTGGAGCGTTGACACACTTTCATGACGTTTTGTGTATGGAATTTATAATCTGGGCCAGTGGCTCCATGGTGTTTAACCTGTACAGGCACAAGCAGCGGGTCCACCATATTCACAGCCAAAATAGCTCCTCAGGATCTTCCCCCGAGAGCAGAGCCACTCAAAGCATCCTCGTCCTGGTGAGTGCCTTTGTCTCTTTCTATGCTGTATCCTCCGCCATCCATGTTTGGTTTTCTCTCCATGGTGAAACTGCTTGGTGGCTGGCCAGAATTTCTGCCTTCATCAATGCTTGCTTCTCCGAGGCCAGCCCCTTTATTCTCATGAGCCGTGAGCGCCGTGTTTCCAGGCTCATTTGGAAGACGTGA

>Chinchilla_lanigera_intact_V1R3_2

ATGAGGCACCACAGGAATTGTCTCCATTTTTATGAACGATATGTGCTTTACTGGAGGCACTGGGAAGAAATCTGTGCACCTTATTCTCCGCTGTTTTGTTTTCACAGATATCAACCTGCTTCTTTCCAAGCGAATTCCAAGAACAACAGCAGCTTTGGAGTGAGAAACTTCCTGGATGACATAGGCTGTAAGGTTGTGTGTCACCTGCAGAGGATGGCTCTGGCCTCTCCATCTGCACCAGTTGTGTCCTCACCATGGTGCAGGCCATCAGCATCCATTCCAGACACTCAGACTGGAAGAGGCTCCAGCTCAGGTCTGCATGGCAGGTCCTCCCCCTGCTTCTTTCCTGTGGGGACTCAATGCTATGCTAAGCATGAACTTACTCTGTATCATCTCAGGTACCAACATGAACCCAATTTATTTACCCATAAATTAGGAAAGCTGACAAATATTGTAATTTTCTACCAAAAATCAGACAGCAAACTGGATTTTCTTCTCTCTCCTGGCCCTGAGAGACACTGTGTTTCAGGTCTCATGCTTGGGGCCAGTGTCTACATAGTGTTTCTTTGCCAGAAGCAGCACCAGTGTGTCCTCCACCTGCAGAAGTCCAAAGTGGTCTACAAAACTCCCCCTGAGATCAAAGCTATCCAAAGTGTGCTCCTTCTGATGCTTTGTTTTCTTTTCTTTTCTTGGACAGATTGTACCACATCTTTACATTTAATTTTGTACTTCAACTATGACTCCATAACGGTAAATCTTCAAGAATTTCTGACCTTTGCTTATGTAATTCTCAGTCCTTTTGTGTGGATTCGCAGGGATGGATGCTGGCTAAATGCTGGCATACTCTGCAAGGTGGAAAGACATTGA

>Chinchilla_lanigera_intact_V1R3_3

ATGAGCCCTGTGGATTTGAAATTTGGAGTGGTCTTCCTTGTCCAGGTTGTCCTTGGAACTCTGGGGAATTTAGCACTCTTGTATCATTATTGTATCCTTTACTTCAGTGGATGCAGGTCCAGGTCCACAGATGTGATCCTCAGGCACCTGACTGTAGCCAACTCCATTGTTATTCTCTCCAGAGGGATCCCAGAGACCATGGCAGCTTTTGGGTTGAAAGACTTCCTCAGTGATGTTGGATGCAAACTCGTTTTCTATGTGCACAGGGTGGGCAGGGGGGTGTCTATCAGCACCACCTGCCTGCTGAGTGTCTTCCAGGCCATCACGATCAGCCCCAGGAGCTCCAGGTGGGCAGAGCTGAAGGTGAAAGCCCTGAAGTGCAGCGGCTTGTCTAACGGCCTCTGCTGGGTCCTGCACCTGCTGCTAAATGTCCGAGTTGCTATGCTTGTGACTGACAGATGGAACAACAGAAACATCACAAAAGCTGTATCTTTTCAGTACTGTTCAGCTGCGCTTCCCACCAAAAGTACAGGGTTCGATTTTGGTGCATTCACCTGCTTTCATGACATTTCATGCTTGGCTCTCATGGTCTGGGCCAGCGGCTCCATGGTTTTTACTCTATATAGGCACGAGCGGCAGGTCCACCATATCCACAGACACAGCAGCGCGTCGAGACCTTCGCCTGGGACCAGAGCTTCTCAGAGCATCCTCGTCCTGGTGAGTGCGTTCATACTCTTCTACACCCTGTCTTCTGTCGTTTATGTCTTGTTTTCTTTTTATCATGAAGGTGCTTGGTGGCTGGTCCAAACTTCTGCCTTTGCCAATGCTTGTTTCCCCACGACCAGCCCTTTTATTCTCATGAGTCGTGAAGGTGGTGCATGTAGGCTCATTTGGAAGAAATGA

>Chinchilla_lanigera_intact_V1R3_4

ATGCGTTCAAACAATTTAGCAATAGGAATAATCTTCTTGTCTCAGATAATCATCGGTATCCTGGGAAATTTCTTCCTTCTTTACCATCATTTTTTCCTTGTCCACAAGGGAAATAGTTTAAGGTCCACAGATGTGATTCTGAAACACTTTTTTGTAGCCAACCTCTTTGTTCTTTTTTCCAGAGGAGTTCCACACACAATGGTAGTCTTTGGGTGGAAACAATTCTTCAGTGATTTTGTGTGCAAACTTCTCTCCTATATTGAGAGAGTGGGCAGGGTTGTGTCAATTGGTACCATCTGCATCTTGAGTTTTGTCCAGAACATCATGATCAGCCCCATGAACTCTTGCTGGAAAGATGTTAAAGGTAGAGTTCTAAAGTACATTGGCTTGTCCCTTAGACTCTGTTGGATCCTAAATATGGTGTTCAATTTAATTATTCCTCTGTACTCAGGGTACTTGTCTGGAAAAGGATATGGTGAAAACATCAGAAAGAAAGTAAATGTAGGTTACTGTTGTCTTGTTGACTACGGGGTGGTCCTTGGCTCAATCTATATATCATTTGTAGTTGTACCTGAAGTTTGTTTTTCTTTGCTTATTATCTGGACCAGTGGCTCTATGATTTTCATCCTGTACAGACACAAGCAGCAGGTTCAACACATTCACACCAGTAATGTCTCTCTCAGATCCCCTGAGTCCAGAGCCACACAGAGCATCCTTCTATTGGTGAGCACGTTCATATCTTTTTACACTGTCTCCACCATCTTTCACACTTCTATGACTGTACTTCCTAGACTCGGTTCATGGTTGCTGGGTACTTCTAAAGTAATTACTGTGTGTTTTCCAACCATCAGTCCCTTTCTGATCATGAGTCAGGATCCCTCTATATTGAGGCACGGTTTCACCTACACAAGAAACACACAACCTTTCTAA

>Chinchilla_lanigera_intact_V1R3_5

ATGGCCTCCACGGATATTAAAATTGGCATAGTCTTCATTGTGCAGGTGGTCATTGGGACTGTGGGGAATTTCTCTCTCTTCAGTCTGGACATATTCCTGTACTACAGTGGCTACAAGACAAGGTCCACAGATTTGATTCTCAGGCACCAAACTGTGGCCAACTTCTTGGTCATTCTATCTAAAGGAATCCCAGAGACCATGGCAAGATTTGGGTTGGAGCACTTCCTCAGTGATTTTGGATGCAAATTAGTTTTCTATGTTCATGTCTTAAGCAGAGGTGTGTGCTTTGGCAACACCTGCCTCCTGAGTGTCTTCCAGGCCATCATCATCAGCCCCAGAGAATCGAAGTGGGCACAATTTAAAGAGAAAGTTCCCAAATACACAAGAAATTCCACTACTCTGTGTTGGGTTCTGCAGATGTTGCTGAGCTCCAGATTTGCAGCGACTATAACTAGCAAATTGAAAACCAGAAACATCACAAACAAAATAGACTTCGCATACTGCTCCAATGTACAATCCGTTGAACACTTATATTCTGTAAGTATAGCGTTGATCTCCATCAGTGATATTCTATGTATGGGGCTCATGCTCTTTGCCAGTGGCTCCATAGTTTGCATCCTGCACAGGCACAAGCACCGGGTCCAACACATCCATAGGACTAATAGCTCTGCCCGATCCTCTCCCGAGACCAGAGCCACTCACAGTGTCCTTGTCCTTGTGAGTGTCTTTGTGTGTTTCTACATCCCCTCTGCCATCATGCACATGTATCAGGTTTTTTCTGGAAAGCACAGCTCCTTCCTGCACACTCTAGGAGCCTTCATAAACGGATGTTTCCCAGCCATCTGCCCCTTCTTGCTCCTGAGCCGTGGTCACAGTGTATCCCAAGTCAGCTGCATCTGCTGTGCAAGGAACGCAGAGCCAGAGCCATCTCATCACAGCAGGAAAACGTGTGCACCGCCTGCTTTTTAG

>Chinchilla_lanigera_intact_V1R4_1

ATGGCCTCCATGGATGTAAAAATTGGCATAGCCTTCATTGTGCAGGTGGTCATTGGAACTGTGGGGAATTTCTCTTTCTTTAGTCAGGACATATTCCTGTGCTACAGTGGATACAAGAAAAGGTCCACAGACTTGATTCTCAGACACCAAACTGTGGCCAACTTCTTGGTCATTCTCTCTAAAGGAATCCCAGAGACCATGGCAAGATTTGGGTTGGAGCACTTCCTCAGTGATTTTGGATGCAAATTTGTTTTCTATGTTCACGTCTTAAGCAGAGGTGTATCCTTTGGCAGCACCTGCCTCCTGAGTGTCTTCCAGGCCATCACCATCAGCCCCAGAGACTGGAAATGGGCACAGTTTAAAAGGAAAGTTCCCAAGTACACAAGCATTTCCACTGCCATGTGCTGGGTGCTGCATTTGTTGCTGAGCTTCGGTTTTGCTGTGAGTATAACTAGCAAATTGAAAACCAGAAACATCACAGATAAAATGGACTTTGCATACTGCTCTGATGTACATCCCATTAGAGGGTTATATTCCTTAAGTATAGCATTGATCTCCATCAGTGATATTCTGTGTATGGGGCTCATGCTCTTTGCCAGTGGCTCCATGGTTTGCATCCTGTACAGGCACAAGCAGCAGGTCCAACACATCCATAGGTCTAAGAGCTCCTCCCGATCCTCTCCGGAGAGCAGGGCCACTCACAGTGTCCTTGTCCTTGTGAGTGTCTTTGTGTTTTTCTACACCCCCTCTGCCATCATGCACATGTATCTGACTTTTTCTGGAAAGCCCAGCTCCTTCCTGCACACTCTAGGAGCCTTCATAAATGGATGTTTCCCAACCATCTGCCCCTTCTTGCTCCTGAGCTGCGGTCACAGTGTATCCCGAGTCAGCTGCATCAGCTGTGCAAGAAATACAGAGCCAGAGCCATCTCATCACAGCAGAAAAAATCTGTGCACCACCTGCTTTTCAGTATTGAGCTACGGGCGGACTCCACTGCCAAGGACTGTAAAAATGCAGGGGACATAA

>Chinchilla_lanigera_intact_V1R4_10

ATGGGTTTTAACTTTGTTAAAGGAGTAATTTTCTTCTTTCTAACTGGACTTGGCATGACGGGAAACATCTTTGTTTTTATGAATTGTACATGCTTTATTGGAGGCACTAAGATGAAAAGTACGCCCTTGATTCTCACCCATTTGGTTTTTACAAATATTATCCTGCTTCTTTCCAAGGGAATTCCAAGAACAATAGCAGCTTTTGGTTTCAGAAACTTCCTCAGTGACATTGGCTGTAAGGTTGTTGTTTATATGGAGAGAGTGGCCCGGGGCCTCTCCATCTGCACCAGCAGTCTCCTCACTGTGGTCCAGGCCATCACCATGAGTCCCAGACACTCAGCATGGCGGAGGGCCCAGCCCAGGTCTGCATGGCACGTCCTCCCCTTGCTCCTCTCCTTGTGGGGCCTCAATGCTATGGTAAGCATGAACTTACTCTATATCGTCTCGAGTACTAATATGAACTCATCACAAATTAGTAAAAGTGACGTCTATTGTGGTTTTCTACCAAAAAGTCAGAAAGTGAACTGGATTTTCCTCACTCTGCTGGCCCTGAGAGACACTGTGTTCCAAGGTCTAATGGGTGGGGCCAGTGTCTACCTGGTGACTCTTCTCCACAAGCACCACCAGCGGGTTCTCCACCTGCAGAAGTCCAAAGTGGTCTGCAAAACTCCCCGTGAGATCAAAGCTGCCCAAAATGTGCTCCTTCTGATGCTTTGTTTTCTTTTCTTTTATTGGACAGATTGTTTCACATCTTTATATTTAGCTTTCTCCTTCAAGAGTGACTCCATAACAATAAATCTTCAAGAATTTCTGACTTTCGGTTATGCAATCCTCAGTCCTTTTGTGCTGATTCACAGGGATGGATTCCAGGCTAAGTGCTGGCATGCTCTGCAGGGTGGGAAGATATTGAGAAATTGTCTATTGCATTCTCCTTTCTATAGGTGGAAATGA

>Chinchilla_lanigera_intact_V1R4_11

ATGAATTCAAAGGATAATTGGAGTAACCTTATCCGGAAAATAACGTTCTTTTCTCTTACTGGAACTGGCACTGCAGGGAATTCCTTTTTGTTTGTGAAACATTTGTACACTTTAGGTGTGGAGACTAAGAAAAAAGGCACAGACTTTATTGTAATTCAACTGGCATTCGCTAATGCGATGACACTCTTGGCTTCTGGAATTGCACATATAAGATCACCTTTTCATTTTCATTACTTCCTAGGTGATGTTGGTTGTAAAGTTGTGATTTTTCTGGGAAGAGTGTCCCGGGGCCTTTCCCTCTGCACAACCTGTCTTCTCAGTGTGGTCCAGGCCATCACCATCAGTCCCCTGAACACCTCATGGACAAAGCTCAAACCCCAGAAAAAATGGCAAGTTCTTTCTTGTCTCCTCTTATTTTGGTTCTTGAATTTCCTGATAAGCTCCAACTTGCTCTACTACATCAGAGCAGTAAATAGCATCAACAGATCTGTTATTAGAATACATGTTGGGTATTGCTACATGATGCCATCTAGGCAGATAGTCAGGTGGCTTTTCCTGTCTCTCATGACTCTTCGAGACATCATCTTCCAGAGTTTCATGGGCTGTAGCAGTGGATACATGGCTGTATATCTGTATGAGCATCACAAGCGTGTCCTCTACTTACACAGCTCCAGGTCTGCAAATGAGAGCTACTCTAGGTCTGCAAACCAGAGCCCAGAAATCAGAGCTACTCTAAGTATTCTCATTCTCATGACCTATTTCCTTTTTTTTTATTGGGCAGATTTTGTTTTTTCCTTCTACACAGGCTCAACCGTGACACATGAGTTCACAATACTAAATATTAAAATATTTCTAGGACTTGGTTATGCAATTCTCAGCCCCATTGTGCTGATCAGCAGAGATGCCCACATGGTTAAATGTTGGCACAGACACAGAGAATCTGCAGAAGCTGCCTTTTACACTAACTCCTTAGGAAACTGA

>Chinchilla_lanigera_intact_V1R4_12

ATGACTTGTTGCTTTATCAGTGCAATAATTTGTGCCTTTCTAACTGGACTTGGTATTTTAGGTAACATGCTAATTTTTGTGATTTATATGTGCAATTTGGGGCGGGACACCAAGAACAAATTAATACAGCTCATTCTTATCCATCTAATTTTCACAAATATCTTAATGCTTGTTTCCAAAGGAGTGCCAAAGACAATAGCAGCTTTTGGCATGAGAAACTTCCTAGATGACCTAGGCTGTAAGATCATCTGTTACCTGGAGAGGGTGGCCCGGGGCCTCTCCATCTGCACCAGTGCTCTCCTCACGGTGGTCCAGGCCATCACCATCAGTCCCAGACACCCTGGGTGGAGGAAACCCAAACCCAGGTCTGCATGGTGCATCCTCCTTCTGCTTCTCTCTTTTTGGGGTCTCAACTCTTTGATAGCCATGAATTTACTCTATTCCATCTCAAATATCAGCATGAATTCATCACAAATTAATCACAGTGAGAGCTATTGTTATTTACTACCACAGAGTAAGAAAGTAAAGTGGATTTTTCTCACCTGCATGGTGCTACGGGATGTGGGGTTTCAGGGCATCATGGGTGGGGCCAGTGGCTACATGGTGTGTGTTCTCCACAAACACCACCAGCAGGTTCTTCATTTGCAGAACTCCAAGTTCCTCTACACAGCTCCTCCTGAAATCAAAGCTGCTCAGAGTGTTCTCCTTCTGATGCTTGGTTTTCTTTTGTTTTATTGGATAGATTGTCTTCTTTCTCTCTATATAAATTCCTCTTTAAGTACTCAGTCCACTGTCACAAATGCTCGAGCATTTCTCACTGTTGGTTATGCAGTTCTTAGTCCATTTGTATTGATTCACAGAGAGGGACACCTGGGTCATTGTTGGAGCACTTGTGAGACAGAAAGACACTGA

>Chinchilla_lanigera_intact_V1R4_13

ATGGCTTCCATGGATTTGGCAATGGCAGTGGTCTACTTCTCGCAGACTCTCATTGGAACACTGGGGAATTTCTCTCTTCTTTGCCACTATACCTGCCTTCATTTCACTCAGTGCCAATTAAGATCCACAGATCTGATTCTGAAGCACCTGTTTGTAGCCAACTCCTTGGTCATTCTGTTTAGAGGAGTTCCTGACACAATGGCAGCATTTGGTTTGAAAGATTTCCTCAACGACCTCGGATGCAAACTTGTTTTCTATTTCCACAGAGTAGGCAGAGGTGTGTCCATTGGCAGCACCTGCCTCTTGAGTGTCTTCCAGGTGATCACGATCAGCCCTAGGAGCTCCAGGTGGACAGAGTTTAAGTTAACAGCCAACAAGTACATAAGCATCTTCAGTATCCTGTGTTGGATCCTCCACATGCTGTTAAACACAATTATTCTTCAGTATATTACTGACAGATGGCGCCATAAAAACATCACCTACAGAAAAGAGTTTGGATACTGTTCTCGTGTTTACCAACACTCTTGGAAGGTCCTGCATGCAGCACTGCTGTCATTGCCTGATGCTTTGTGTGTGGGGCTCATGCTCTGGACCAGCAGCTCCTCAGTTTCCATCCTGCACAGACACAAGCTGAGAATGTGGCATATTCATAAGACCAACATGCCCAGTAGAGTCTCCCCTGAAACTCAGGCCACTAAAACCATCATTCTCCTCGTCAGCACCTTCATCTATTTTTATACCCTCTCCTCCATTTTCCAAGCGTTTTTCACTTTGTATAACAATCCCAACAGGTTCCTCGTGAATGTTGCTTCTGTAGTCACCGGGGGTTACCCAACTGTCAGCCCTTTTCTGCTCATGAATCATTACCCAAGCGTATCCTGTCTCTCTCTTGACTGTACAAGGAAAACAAAACCTGTCATGTCATGA

>Chinchilla_lanigera_intact_V1R4_14

ATGAGTTGGAGTAACCTCATCCAGAGAATAATTTTCCTCTCTCTTAGTGGACTTGGAAGTATTGGAAACATCCTTATATTCATAAAACATATACATGTTTTTGTTATGATTACTGAGAAAAGACCCATAGACTCTATCCTCATCCACTTGGCCTTTTCAAACACAATCATTATATGTAGCACAGGGATCAGAGGTACAACCACAATTTTTTATTTTAGAAACTTCCTAGGTAGTGTTGGATGTAAAATTGTGATTTATTTTGAAAGGGTGGCTCGTGGTCTTTCCATCTGTACCACCTGTCTCCTCAGTGTGGTCCAGGTGGTCACTATCAGTCCCAGGACCATCCTGTGGAGAAAGTTCAAACCACAGACTACAGGGCAAGTTCTTCTATGTCTCCTCCTATTTTGGATCTTTAATTTCCTAATAAGTTCTAACTTGCTCCACTACATCACAGCAGTCAGAAGCATGAATCAATCTGGGATTAGAATATATATTGGCCATTGTTATATGCTACCATCTAGGCAAATAGTTAGATGGATTTTCCTCTCTTTCATGGCTGTTCGGGATCTCATCTTCCAGAGTCTCATGGGCTGGAGCAGTGGGTACATGGCTATCCGTCTGTACAAACATCACAAGCGTGTTCTCTACCTGTATAGCTCTAGGTTTGCAAACAATTCCAGCCCTGAAATCAGAGCTACTCAAAGAATTCTCATTCTCATGATCTGCTTCCTTTACTTTTACTGGGCTGACTTCATTTTCTCCTTCTACATGGGATCTATCTTGCCAGATGGTTCCACAACATTAAATATTAAAATATTTTTAGTACTTGGTTATGCAGGTGTCAGCCCCTTTGTCCTGATGAGCAGGGCTGTCCATGTTGCTAAATACAGGAATGCACACTGA

>Chinchilla_lanigera_intact_V1R4_15

ATGACAACCAGTGAAGGGGTCATAGGAGTGATCTTCTTATTACAGACTGTGGTTGGGGTTCTCGGCAATTCCTCTCTTCTTTACCACTATCTGTCCCTTTACTTCACTGGGTGCAGACTAAAGTGCACAGACCTGATTCTTAAGCACGTGATTGTAGCCAACCTCCTAACTCTCCTAGGTAGAGGAGTGCCCCACACAATAGCAGCTTTTGGTTGGCAAGTTTCCCTTGGTGATGTGGGGTGCAAACTACTGTTCTATCTGCAAAGAATAAGCAGGGGTGGGACTATTGGCAGCACCTGTTTCCTGAGTGTCTTCCAGGCCATCACCATCAGCCCCAGGAACTCCAGGTGGGCAGAGCTTAAAGTGAAGGCTCCCAAGTATGTTGGCTCCTCCTTGTGCCTGATCTGGGTCCTGTACAGCCTTGTCAATGTTATTTTTCTTATGTACACCACTGGGAACTGGAGCAAGAAGAACCTCACAAGCCTCAAATATCATGGGTACTGTTCCAGTATTCGTCATGACAAAACCACAGAGTCATTATACGCAGCCCTGCTGTCCCTCCCTGATGTTTTGTGTTTGGGGCTCATGCTCTGGACCAGCAGCTCCATGGTTTCCATCCTGTACAGGCACAAGCAGCAGATGAGACACATCCACAGGACTAATGTCAGCCCCGGATCTTCTCCTGAGTCCAGAGCGACCAAAACCATCCTTCTCCTGGTCAGCACCTACGTCTGTTTTTACACACTCTCCTGCATCTTTCAAGTTTCCTTGCTATTTATTTATAATCCCACCTGGTTCCTGGTGAACATATCTGCAATAGTTGCTGGCTTTTTCCCAACTGTCAGCCCTTTTCTGATCCTGAGCTGTGATGCCAATATATGCGCATTTTGTTTTGACAACATAAGAAATAGAAAATCTCCTACTCGTATGAGGAATGGGTAA

>Chinchilla_lanigera_intact_V1R4_16

ATGATCTTGCACCTTGTCAAGGGAATAGTCCTAGCCTTTCTGACTGGACTTGGAGCTGCAGGGAACATCGTTGTTTTTGTCAGTCATATACTCATGTTTGGGGGCATGGAGAAGAAAGCTATACATCTTATTCTCATCCACTTGGCCTTCACAAATATCATAATGCTTCTTTCTAAGGGAGTGCCAAGGACAATCACAGCTTTTGGCATGAGAAACTTCCTAGATGACATAGGCTGCAAGATGGTGTGCTACCTGGAGCGGGTGGCCCGGGGCCTCTCTGTCTGCACTAGCAGTCTCCTCACTGTGGTGCAGGCCATCAGTATGAGTCCCAGACACTCCAGGTGGAGGAGGCTCCAGCCCAGGTCTGCGTGGTTAATTCTTCCTTTGTTTCCCTTCCTGTGGATATTCAGTTTTTCAACAAATGTCAACTTACCCCTCTACATTACAAGTGCTAGTGTGAACACATCGCAGATTAGCAAGAGTGACTACTATTGTTATTTTCAGCCAGAAAGTCAGAAGGTAAGATGGATCATTCTCAGTAGCATGGTCTTTTGGGATGCTGTGTCTCAGTGTATCACGTGTGGAGCCAGTGCCTACATAGTGTTTCTTCTCCATAAGCACCACCGGCGTGTTCTCCGCCTGCAGAGGTCCAAGTTCCTCTACAAAACTCCCCCCGAGATCAAAGCTGCCCAAAGTGTTCTCCTTCTGATGCTTTGTTTCCTCTTCTTTTATTGGGCAGATTGTGTTTTTTCTCTGCTTGTGAATTGTTTCTTGGAGAACAATCCTATATTAAATATTCGAGAGTTTCTTACCCTTGGTTATGCGATTCTCAGCCCCCTTGTGCTGATTCACAGAGACGGACATGCAGTTGACTGCTGGTCCACTCAATGGGAGAGAGGGACTTTCAGAAAATATTTATTCATTCTATTCTTTCAATAA

>Chinchilla_lanigera_intact_V1R4_17

ATGACCTCCAAAAATTTTCCAATGGCACTAGTCTGCTTATCTTACACTATCATTGGAATACTGGGGAATTTTTCTTTACTTTATCACTATATTTTTCTTTATGTCACCAAATGCAGAATAAGAGCCACAGATCAGATTATCAAGCACCTGGTTTTAGCTAATTCCTTGACCATTCTGTTTAGAGGAGTTCCTGAAACACTGGCAGCATTTGGTTTGAAAGATTTCCTCAGTGATGTTGGATGCAAACTTGTTTTCTATGTTCATAGAGTAGGCAGAGGCGTGTCCATTGGTAGCACCTGCTTCTTGAGTGTTTTGCAGGTGATTACAATCAGCCCTAGGAACTCCAAGTGGGCAGATATTAAGCTAACAGCCACCAAGTATGTAGGCATCTTTAATACCCTGTGTTGGACACTTCACATGCTGTTAAACATAGTTGTTCTTATGTATATGACTGACAACTGGAGTAATAAAAACAACACATACAAAAAAGAATATGGATACTGTTCTGGTATTCGTCAACAAAAAGCTAAGACAGTCCTATATGCAGCAATGCTGTCTGTCCCTGATGTTTTTTGTATGGGGCTCATGCTCTGGACCAGCAGCTCCTTGGTTTCCATCCTGTACAGGCACAAGCAAAGAATGCAGCATATTCATAAGACCAACATCACATATAGATCCTCCCTTGAGAACAGAGCTACTAGAACTATCATTCTGCTGGTCAGTACCTTTGTCTATTTTTATACCCTATCCTCCATTTTTCAAGCTTTTTTTATTCTCTATAATAATCCTATCAGGTTCCTACAAAACATGGCTGCGATACTCACTGGGCTTTACCCAACTATCAGCCCTTTCGTGCTCATGAAACATTATCCAAGAATATCCAGTCTATTGTTCCTTGCTTGTGTAAGCAAAAAAAAAATCCTGTAA

>Chinchilla_lanigera_intact_V1R4_18

ATGGTTTTTGTGGATATGGTGATGGGAATAGCCATCCTCACTCAGACTGGAATTGGGCTGGTGGGAAATTCTTCCTTCCTTTTCCTTTACATTTACCGCCTTTCCAGGAAACATCGATTGAGACCAGTAGCTCATATAATCAGCCATCTTGCACTGGCCAATACATTGTGGATTCTTTGTGGAGAAATCCCTCAGACAATGGCAGCCTTTGGCCTTCAGTATTTCCTGGATGATGTTGGATGTAAACTTGTCTTTTACTGTAACAGATTGGCCTGGGGAAATTCTCTCACCACCACCTGTCTTCTAGGTGGTGTCCAGGCCTTAAGTATCAACCCAACTAACGTGAGAAGGTCAGAACTCAAAACCAAATCGATACAACATATTACTTCTACCTGTATCCTGAGTTGGGTGTTTCATTTGCTGGTGAATATCGTTGTTCCCGTGACAGTGACTGGTCAAAAAAATAGCAGAAATAACACTGTGAATTCAAATCTGAAATATTGCTCTCGTCTTTTCTTTGATACAATTACAGAATCAGTATGTTTACTGTTTTTCTCCTCTATTGATATTTTTTGCTTGGGATTCATGATATGGGCTGGTGGCAGCATGATACTTTTCCTTCATAGGCACAAACAGCAAGTTAGATATATTCATAGCCTTAGACAATCTCCTCACATATCCCTGGAGAGCAGGGCCACAAAATCTGTCCTGCTCCTTATGAGCACTTTTGTCTTATTTTATTCTCTCTCTTCCACTTTCGAAGCTTGTATTTTTCTTTCTGACAATCCACAGTCATGGCTTGTGAACACCGGTGTGATTTTAACATCTTGTTTTCCAACCATCAGCCCCTTTTTGCTCTTAAAAAGCAACGTCTGTATCTTCCATTTCTAA

>Chinchilla_lanigera_intact_V1R4_19

ATGTATTTCCACATTATCAACAGAACAATCTGGACCTTTCTAACTGGACTTGGCATATTAAGTAACACATTAGTTTTTATACTCTATGTGTGCAATTTTGGGGGAGCCATCAGGAACAAATCAATACAGCTGCTTCTCATCCATTTACTTTTCACAAATATCTTGATGCTTGTTTCCAAAGTAGTGCCAAAGACAATAGCAGCTTTTGGTGTGAGAAACTTCCTAGATGACGTAGGCTGTAAGACTGTGTGTTACCTGGAGAGGGTGGCTCGTGGCCTCTCCATCTGTACGAGTGGTCTCCTCACTGTGGTCCAGGCCATCACCATCAGTCCCAGAGACTCTGGGTGGAGGAGACTCAAGCTCAGATCTCCATGCTATGTCCTCCTTTTGCTTTTTTCTTTTTGGGGCCTCAACACTTTGATAAGCATGAACTTACTCCATTCCATCACCAATATCAGGATGAACTCATCCCAAGTTAGTAACAGTGAGGGCTATTGCTTTTTTCTACTAGAAAATAAGAAGGTAAAGTGGATTTTTCTCACTGGCATGGTCCTACAGGATGTGGGCTTTCAAGGCATCATGGGTGTGGCCAGTGGCTACATGGTGTGTGTTCTCTACAAACACCACCAGCACGTTCTTTATTTGCAGAACTCCAAGTTCCTCTACACAGCTCCTCCTGAAATCAAAGCTGCTCAAAGTGTTCTCCTTCTGATGCTTTGTTTTCTTTTCTTTTATTGGCTAGACTGTCTTTTTTCTCTATTTATAAGTTCCTCATTAGTTACTCAGTTTACTGTCATAAATGCTGGAGAATTTCTTGCTGTTGGTTATGCCATTCTTAGTCCATTCATACTGATTCACAGGGAGGGACACCTCGGTCATTGTTGCAGCACCCGTAGGGCAGGAAACCACTAA

>Chinchilla_lanigera_intact_V1R4_2

ATGGTATTGAACATGATCAAAATAACAATATTCATCCTTCTAACTGGACTGGGTATGGTGGGGAATATATTTGTTTTTGTAAATTATATGTGCGTGTCCTGTATGGATCCTGGAAAGAAACCTATACGTCTTATTCTCATCCATTTGACTATTACAAATGTCATAATGCTTTTTTCCAAAGGAATGCCAAAGACGGTAGCAGAATTTGGCTTGGAAAACTTCCTCAATGACACAGGCTGTAAGATTGTTGCTTGTCTGGAGCGAGTGGCCCGGGGCCTCTCCATCTGCACCAGCAGTCTCCTCACCGTGGTCCAGGCCATCACCCTGAGTCCCAGACACTCCAGGTGGAGGAGGCTTAAGCTAAAGAATCCATGGCACATTCTTCTCTTCTTTCTCTTCTTTTGGATACTCAACCTCTTGATAAGCATAAACTTGATCCGTTCTATCCTGAGTAGAAGCAAGAACACATCACAAAGTAGTAAAAGTGACAAGTATTGTTACTTTCTACTAAAAATTCGGGGGGAAAACTGGCTTTTTCTCATTTTCATGGCTCTGAGAGATACCATGTTTCTGGTTGTCATGGGCGGGGCCAGTGTCTACATGGTGCTGCTTCTGCACACACACCACCAGCGTGTTCTCCACCTGCAGAACAAGTTCCTCCGTAAAGCTCCTCCCGAGATCAGAGCTGCTCAGAGTGTTCTCCTTCTGATGCTGAGTTTTCTTTTCTTTTACTGGACAGATTGTGCCATTTCTTTATATGTAACTCTCTCCTTAGAGAAAGATTTCCTAGCAGTGAGTGTACGAGAATTTCTGACCCTTGGTTATACAATTGTCAGCCCATTTGTGCTGATTCACAGAGATGGACACCTGGCTGAATACTGGCATGCTTAG

>Chinchilla_lanigera_intact_V1R4_20

ATGGCCTCCAGGGATTTGGCAATGGCAGTGGTCTACTTCTCGCAGACTCTCATGGGTACACTGGGAAATTTCTCTCTTGTTTGCCACTATACTTGCCTTCATTTTGCTCAGTGCCGATTAACATTCACAGATCAAATTCTCCAGCACCTGTTTGTAGCCAACTCCTTGGTCCTTCTGTTTAGAGGAGTTCCTGAAACAATGGCAGCATTTGGCTTGAAAGATTTCCTCAATGACCTCGGATGCAAACTTGTTTTCTATGTTCACAGAGTAGGCAGAGGTGTGTCCATTGGCAGCACCTGCCTCTTGAGTGTCTTCCAGGTGATCACGATCAGCCCTGGGAACTCCAGGTGGGCAGAGCTTAAACTAACAGTCCACAAGTACATAAATATTTTCATTATCTTCTTTTGGATCCTCCACTTGCTGTTAAACACCATTGTTCTTCCGAATGTTACTGACAAATTGCACCATAAAAATATCACATACAATAAAGAGTTTGGATACTGTTCTCGTGTTTCCCAACAAAACTCTAGGAAAGTCCTTCATGCAGCACTGCTGTCATTGCCTGATGCTTTGTGTGTGGGGCTCATGCTCTGGACCAGCAGCTCCTTGATTTCCATCCTGTACAGACACAAGCTGAGAATGCGGCATATTCATAAGACCAACATCCCCAGTAGAGTCTCCCCTGAAACTAGAGCCACCAAAACCATCATTCTCCTGGTCAGCACCTTTGTCTATTTTTACACCCTCTCCTCCATTTTCCAAGCTTTTTTCACTTTGTATAACAATCCTAACAGGTTCCTCGTGAATGTTGCTGCAGTAATCACCGGGGGTTACCCAACTGTCAGCCCTTTTCTGTTCATGAACCATTACTCAAGCATATCCTCTCTCGCTCCTGACTGTGTGAGGAATAAAAAACTTGTAATGTTATGA

>Chinchilla_lanigera_intact_V1R4_21

ATGCTTTGGATGAACCTCATTCAGGGAATAATCTTCCTTTCTCTTACTGGACCTGGAATTTGGGGGAACATCTTCATATTCATAAGACATTTATATATTTTTGCTTTGATCCCTGAGAAAAAACCTATAGGTTTTATTATCATTCACCTGGCTTTTTCAAATGCAGTCATTATTTGTACCACAGGAATCAGAGACATAGTGCCAGCTTTCTACTTCAGAAACTTCCTAGGTAGTGTTGGTTGTAAAGCTGTGGTTTACCTGGGACGCATGGCCCGGGGCCTCTCCATCTGCACCACCTGTCTCCTCAGCGTGGTCCAGGCCGTCACCATCAGTCCCAGGGCCACGCTGTGGGGAAAGCTCAAGCCACAGACTGCATGGCAAGTTCTTCCCTATTTCTTCCTTTTCTGGATTTCTAATCTTCTGCTAAGCTCCAACTTGCTCCACTACATCACAGCAGTCAGTAGCATGAACAGATCTGAAATTGGAATGTACGCTGGGCACTGTTACTTGCTATCATCCAGACAAACAGTCCGCTGGCTTTTCCTCTCTCTCATGGCTCTTCGGGATGTCCTCTTTCAGGGACTCATGGGCTGGAGCAGTGGACTCATGGCTTTCCGCCTGTACGAACATCACCAGCGCGTGCTCTACCTGCACAGCTCCCGGCTGGCAGTGAATTCCAGCCCCGAAATCAGAGCTACACTAAGTACTGTCATTCTCATGACCTGTTTCCTTTTCTTTTTTTGGACAGATTTCATTTTTTCCTTCTACATAGGTTCCACTGTGAGCAATGAAACCACACTCCTGTATATTAAAATATTTCTAGAGCTCGGCTATGCGGTTCTCAGCCCCTTTGTGCTGATGAGCAGGGATGCTAGTGGCGCTAAACGCTGGCGAGCACAGTGA

>Chinchilla_lanigera_intact_V1R4_22

ATGGCCTCCAAGGAGGCAGCAATGGGAGTGGTTTTCTTATCACAGACTGTGTGTGGAGCCCTTGGAAATTTCTTTCTTCTTCGCCATTATCTCTTACTTTCCTTCACTGGACACCAGTTAAAGTCCACAGATTTAATTCTCCAGCATTTGATGGTTGCCAATTCTTTATCCCTGCTCTCTAGAGGAGTCCCCCAGACAATGACAGCTTTTGGCTTGGAAGATTTCCTCGATGATCTTGGATGCAAAGCTGTGTTATATCTTCATAGAGTGGGCAGGGGTGTGTCCGTCAGCATCACCTGCCTCTTGAGTGGCTTCCAGGCCATCACCATCGGCCCCAGAAACTTCAGGTGGGCAAAGCTTAAAGTAAAAGCCCCCAAGTATACTGGCTTCTGTATTTTCATGTGTTGGACCCTGCAAATACTGGTAAATATAATTTCTCCCATATATGTAACTGCAAAATGGAGCAATAGAAACATTACACTGCAAAAGGATTTGGGATTCTGTTCTGCAAATCATGCTGATACAGTCACTCTACAGCTATATGCAGTGCTGCTATTGTTTCCTGATGTGGCCAGTTTGAGCCTCATGATCTACACCAGCATCTCTATGCTCTTCATTCTGTATAGGCACAGGCAGCAGGTCCAATACATTCACAGGATCAATGTCTCCTCCAAGTCCTCCACTGAGTCCAGAGCTACCCGCAGGATTCTGGCTCTTGTGAATACCTTTGTGTCATCTTATACCCTCTCCTCCATCTTCCAAGTCTGTGTTTCTAATTCTCAGAAACCCAGTTGGTGGTTGCTGAACATCGGTGCATTTAGCTCTCTGTGTTTCCCAGCTTTCTGCCCCTTTGTGCTGATGATCCATAACTCACAGTGTCCAGCTCTGTGTCTCGGTAAGAAAAAAAAAAGTGTTTATTTCTTTTCTTAA

>Chinchilla_lanigera_intact_V1R4_23

ATGTTTCTGGGTGACAAGCTTTTTGGATTTGTCCTCATATCTGAAATTTTCCTTGGGTTAATAGGGAACTTATTGGTCTTCATATTATATATGTTCACCTTCTTAATTAATCCTCATCTGAAAAAGCCCATAGATCTGATTTTCACACATCTGACACTGGTCAATGTTTTGACCATCATGTTCAGGTTGCTACCGGATATCATGTCCTCCTTTGGATTAAGACAGTTTTTGGACAATGTTGGTTGCAAAATATTTTTGTATGCATACAGAGTTACCCGGGGCCTTTCCATCTGTACTACCTCTCTGCTGAGTGTATTTCAGGCCATCACTATCAGTCCCAGTAATTCTCAGTGGGAATGGGTAAAATCTAAACTTTCTAAGTGGATTTTTCCCTTGTTCCTATTCTTCTGGATCATTAACATGCTCACTTACATCCCCATAATTGAAACTGTAAGAGCCAATAGGAATTTCACTGCTGTTGGTTCTGTGTTTTCTCAAGCATACTGTCATGTTAATCACTATGGATATGTCATTACAGCATCATTTCTATGTATGACATTGACTCGAGATCTTCTGTTCGTGGTTCTCATGATCTGGAGCAGCCTGTACATGGTGAACCTCCTCTACAGACACCGCCAAAGAACCCACCATGTCCACAGCCTATGTCACTCTTCCCAGTTATCTCCTGAACACAAAGCCACTCACAGCATCCTTTTGCTGGTAAGTTGCTTTGTGTTCTTTTATGGTTCAAACAACATCATCATGTTTTGTTTGTTTTTTAGACCCAAGAAAATCCCAGGACTGGACAGGATCAATGGAGTTTTATCATCATGGTATCCAGCCATCTGTCCTTTTATCTTAATGAAAAATAAGAAAATTATCTCCAAATTTAATTTGCCTTTTTAA

>Chinchilla_lanigera_intact_V1R4_24

ATGAGCCTCGACACTCAGAGGAACGACTGTTATAGCAATTTACATGACATTTTCAAAGGAAATGACAAAATAACTTCCAAAGATTTGGCAGTAGGAATAATCTTCTCCTCACAGACTATCATTGGTGTCACAGCCAATTTCTTCCTTCTTTGTCATTATTTTTTCAATTTCCAGCATGGAAGGAGGTTAAGATCCACAGATGTGATTCTGAAGCACCTTTGTCTGGCCAACTCCTTAGTTCTTCTCTCTGGAGGAGTTCCTCAAATAATGGCAGCATTTGGGTTGAAACATATATTCAGTGATATTGTTTGTAAATGTCTCTTGTATATTGAAAGAGTGGGCAGGGGTGTTTCAATTGGTACTATCTGCCTCTTGAGTGTCTTCCAAAACATCATCATCAGCCCCATGAATTCTTGTTGGAAGGATATTAAACGTAAAACTCAAGAATATATTGGCTTCTCCATTCACCTTTGCTGGATCTTAAATGTGTTGGTCAGTTTAATTATTCCTATGTATGCATGGTATTCATCTGTGAAAGGTTATGACAGAATCATCAAAAAGAAAGTAAATATAGGATACTGTTGTCTTGTTGATTATGGGGTAGTCATTGGCTCAGTCTATATAGCATTAGTAGTTGTACCTGAAGTTTGTTTTTCTCTGCTTATAGTGTGGGCCAGTGGTTCTATGATTTCCATCCTGTACAGACACAAGCAAAGGGTTCAACACATTCATATCACTAATGTTTCCTTAAGATCCCCTGAATCCAGAGCCACACAAAGCATCCTTTTCCTGGTGAGCACCTTCATATCTTTTTACACTCTCTCCACCATTATTCGAATTTCTATTGCTTTATCTTACAATCCCAGTTCGGGGTTGGTGAGTGCTTCTGATCTAATTTCTTTGTGTTTTCCAACTATCAGCCCCTTTCTGAGCATAAGCCAATGCTCCTCTATGTTTAGGCACTGCTTCGCCTGCACAAAGAACACAAAACCTCTCCAACTCTTTCAGAAATGTACGACTTGTATATTGTGGCAGTACTTTCTGTTGTTTACTCTTCACTTCTTTTTATAA

>Chinchilla_lanigera_intact_V1R4_25

ATGGCTTTCAACCTTGTTAAAGGAGTCATTTTCTTCTTTCTAACCGGACTTGGCACGGTGATGAACATCTCTGTTTTTATGAATTATATATGCATCATTGGAAGCACTGAGAAGAAATCTATGCACCTTATTCTCATCCATTTGGTTTTTACAAATACCATACTGCTTCTTTCCAAGGGAATTCCAAGGACAATAGCAACTTTTGGCTTGAGAAACTTCCTGGACAACATAGGTTGTAAGATTGTTGTTTACCTGGAGCGGGTGGCCCGGGGCCTCTCCATCTGCACCAGCAGTCTCCTCACTGTGGTTCAGGCCATCACCATTAGTCCCAGACATTGTGGATGGAGGAAGCTCAACCCCAAATCAGCATGGCACATTTTTTCCTTGTTTCTACTATTTTGGATTCTGAATTCCTTGATAAGCATAAACTTGTTCTATATCATCACAAATATCAACCTGAACTCATCAAAAACAGGTGAAAATGACTCCTATTGTTTTCTACCAAGAAGCCAGAAAATTAACTGGATTTTTCTCACTCTCTTCACCCTGAGAGACACCATGTGTCAGGGTGTCATGGGTGGATCTAGTGTCTACATGGTGTTTCTTCTCCACAGGCACCATCAGCGTGTTCTCCACCTGCAGCTCTCCAAGTTCCTCTACAAAACTCCACCTGAGGTCAAAGCTGCTCAAAGTGTTCTTCTTCTGATGTTTTCTTTTCTTTTTTTTTATTGGACAGATTGTTTCCTATCTTTATATTTAATTTTCGCCTTCAAGAATGAATCCATAACAAACAATCTTCAAGAATTTCTGATACTTGGTTATGCAATACTCAGCCCATTTGTGCTGATTCACAGATATGAACACCGGAGTAAATGCTGGCATGCTTAA

>Chinchilla_lanigera_intact_V1R4_26

ATGTCCTTCAGAGATTTGGCAATAGCAGTAATGTCCTTCTCACAGACTACAGTTGGAATCCTGGGGAATTTCTCTCTTCTTTACTACTATCTAGTCCTTGGCCAGAACAGATGCACACTGAAGTCCACACATCTGATTCTCAGGCACCTGATTATAGCCAACTCCATGATCATTCTATCTAAGTCACTTCCTCAGACAATGACAGCTTTTGGCCTGAAACATTTCCTGAATGACTTTGGCTGCAAAATTCTTTTGTATTTTCAGAGGGTGGGCAGAGGTGTGTCAATTGGTACCACCTGCCTCTTGAGTATCTTCCAGACCATCATGATCAGCCCCATGAACTCTTGTTGGATGGATCTTAAAGTAAAAGCCCCAAGGTACGTTGGCTTCTCCACTGCCTTCTGCTGGATCCTATATATGGTTGTAAATTTAATTTTTCCTATGTATGTGTATGGCAAATGGAACAGCCAAAACATGACAAAGAAAAGGGACTTGGGATACTGTTCTACTGTCGGTGGTGACAAAATAGTAGATTCACTATACACAGTGTTGTTTGTATTCCCTGAAGTTTTATTTTCTGTGCTCATAATCTGGGGCAGCAGCTCCATGGTTTTCATTCTGTATAGGCACAAACAGCGGGCTCAACATATTCACAGCAGTAATGTTTCTCTTAAATCCTCCCCTGAGTCCAGAGCCACTCATAGCATCCTTGTTCTGCTGTGCACCTTTGTGATGTTCTTTTTTCTCTCTTCCATATTAAATGTTTGTGTTGCTCTCTTTTATAATCCCAGTTGGTGGTTGGTGAACATCAGTGCCCTCCTTTCTGTGTCCTTTCCTACTGTCAGCCCCTTTGTTCTCATGAACCGAGCCTCCACTGTACCCAGGTTCTGCTTAGGGAATATAAAAATCCACTAA

>Chinchilla_lanigera_intact_V1R4_27

ATGAATAACAGAATGTTTTCCAGGGATATAGCAATAGGAATAATGTTCTTATCACAGACTACAGTTGGAATTCTTGGTAATTTCTCTGTTCTTTACTATTATTTAGTCCTTTACTACCAAGACTGCACATTAAGGTCCACAGACATGATTCTGAAGCACCTTTTTATAGCCAACTTTTTCATCATCCTCTCTCATGGAGTTCCCCATACAATGGCAGCTTTTGGGTTGAAACATTCCTTCAATGATTTCAGCTGCAAAATCCTTTTGTACATTCACAGACTGGGCAGAGGTGTGTCCACTGCGATGACATGCCTCTTGAGTGTCTTCCAGACTATCATGATCAGCCCCATGAACTCCTGTTGGAAGGACCTTAAAGTAAAAGCCCCAAGGTACACTGAATCTTCCATTTCCCTCTGCTGGATCCTGTATGTGGTGATAAGTTTCATTTTTCCTATACATGCATATGTCAAACAGAATAGCAAAAATATGACAAAGAGCAGATATTTTGGATTCTGTTCTACTGAAGGTAATAACATTATTTCCTCAATATATTTAGTATTATTTATATTCCCTGAAGTTTTATTTTCTGTGCTCATAATTTGCAGCAGTTGTTCCGTGATCATCTTTCTCTATAAGCATAAGCAGCAGGTTCAATATATTCGCACTGTGGCTTCCCGCAGAGCCTCCCCTGAGTCCAGAGCTACACACAGCATCTTTGTCCTGGTGTGCACATTTGTGATTTTTAACACTGTTTCCTCCATATTGTATATTTATGTTGCTTTCATGCATCATCCCAGTGTGTGGTTGGTGAACATCAGTGCCCTCCTTTCTGTGTCCTTTCCCACGGTCAGCCCCTTTGTTTTCATGAGGAGACACTCTACTGTACTGAGGCTCTGCTTTGTCTGGATAAGAAATCCAAGAATTGGTCAATCATAA

>Chinchilla_lanigera_intact_V1R4_28

ATGAGTCCTGAAAATTTGGCAATGGGGATTTTGTTCCTAACACAGACTGGAGTGGGAATCTCAGGCAATTTTTTACTTCTATTTTATTACTCTTTCTTTGTTTTCACTGGAAAGAGTCTGACGCTCAAAGACCTGCTCATACAGCACTTGACTTTTGCCAACTTCTGGGTGATTACCTCAAGGGGAATCCCTCAAACAATGGCAGAGTTTGGCATGAAATATTTCCTGGGTGACACTGGATGTAAACTTATTCTCTACCTTTATCGAGTAGCCCGGGGGATTTCCCTGTACACCACGTGCTGCCTGAGTTGCTTCCAAGCCGTCACAATCAGTTCCAGCAACAGGTGGAGGACACTTAAGCAAAGAGCCACCAAGTACTTTGGCCCATCCTGTTCACTCAGCTGGCTGGTGCATCCGCTTCTAAACATGCTGATTTCCATGAGGACAGTTGGCCCAAGAAACAATAAAAATAGCAGTAAGACATTTAATTTTGGTTACTGCTCAGGGTTTGTTACCGATACCATGGCAACCGCGATCTATGCGCTCATACTCTGCTGCACAGATGGTCTCTGTTTGGGTCTCATGGCCTGGGCCAGTGGCTCCATGGTGAGCATCCTCTACGGGCACAAGAGGCAGGTTCGGTACATCCACAGTGCCCACCGCTCCCCTCGAGTCTCCCCGGAGCCCAGAGCCACCCAGACCATCCTGCTCCTGGTGTGCACCTTTGTCACTTTCTACGCCCTGTCTTCTATCCTACTACTTCACTCAGCAATGTTTGGAAATACAAACGTATGGGCTATGAATGTCTTTACATTCATAGAGACACGTTTTCCCACCCTTTGCCCCTTTGTTCTAATCAGTAACAGCAACTCTGTTTCTAGGCTGTTGTCCCTGCTGTGTGAACAGGTGATTTTCTCCGAACAGTGCACAAGTTCCAGTGATGTACATTCTGTGATGTGTTGGTATATTGCTTCATTCATTAGGTAA

>Chinchilla_lanigera_intact_V1R4_29

ATGAGCTATGCTACTCAGAGGAATGACTGTTATAGCAATTTAAGTGACATTTTAAAAGAAAATGGCAAAATAACTTCCAAAGATTTGGCAGTAGGAATAATCTTCTTGTCTCAGACTATCATTGGTGTCATGGGAAATTTCTTTCTTCTTTGCCATTATATTTTTGTTTTCCATCCTGGAAGGAGATTAATGTCTACAGATGTGATTCTGAAGCACATTTTCATAGCCAACTCATTAGTTCTTCTTTCTAGAGGAGTTCCTCAAACAATGGGAGCCTTTGGAATGAAGCATTTCTTCAATGATATTGTCTGTAAATGTGTCTCGTATTTAGAAAGAGTAGGCAGGGGTGTGTCAATTTGTACCATCTGCCTCTTGAGTGTCTTCCAAAACATCATCATTAGCCCCATGAAATCTTGTTGGAAAGACATTAAAGGTAAAGCTCAAGACTATATGTGCTTCTCCGTTGGCCTGTGCTGGATCTTAAACATGGTGGTCATTTTAATTATTCCTCTGTATTCATTGTATTTGTCTGCAGAAGGTTATGACAGACACATGACAAAGAAAATATACATAGGATACTGTTGTGTTGCTGACCATAGATTACTCATGGGCTCAATCTATATAGCATTAGTAGTTTTTCCTGAAGTTTGTTTTTCTCTGCTTATAGTCTGGGCCAGTGGTTCTACGATTTTCACACTTTACAGACACAAGCAAAGGGTCCAACACATTCATATCACTAATGTTTCCTTAAGATCTCCTGAGTCCAGAGCCACACAAAGCATCCTTTTTCTGGTGATCACCTTCATATGTTTTTACACTCTGTCTTCTATCTTTCGAATTTCTACAACTGTGGTTAATGATCGCAATTCATTGTTGGTGAGCACTGGAGATGTAATTGCTATGTGTTTTCCAACTATCAGCCCCTTTCTTATTATGGGCCAACATTCTTCTATATTAATACGCTGTTTTTCCTGCAGAAGAAACACACAACCTCTCTAA

>Chinchilla_lanigera_intact_V1R4_3

ATGAGTCCTGTTACTCAGAGGAACGATTGTTATAGCCATTTACAAGAAATTTTAAAGGAAAATGACAAAATAACTTCCAAAGATTTGGCAGTAGGAATAATCTTCTTGTCACAGATGATCACTGGTGTCATGGGCAATTTCTTTCTTCTTTGCAACAATATTTTAATTTTCCACCCTGAAAGGAGGTTAAGAGCCACAGATGTGATTCTGAAGCACCTTTGTCTAGCCAACGCCTTAGTTCTTCTTTCTGGAGGAGTTCCTCAAACAATGGCAGCATTTGGGTTGAAACATTTCTTCATTGATATTGTTTGTAAATGTCTCTTGTACACTGAGAGAGTGGGCAGGGGTGTGTCAATTGGTACCATGTGCCTCTTGAGTGTCTTCCAAAACATCATCATCAGCCCTATGAACTCCTGTTGGAAAGATTTTAAAAGTAAAACTCAAGAGTATATTGCATTCTCTATTCCCTTCTGCTGGGTCCTAAACATGGTGGTCATTTTAATTATTCCTCTGTATGCACTGTATTTGTCTGCAGAAGGTTATGACAGACACATGACAAAGAAAATATATGTAGGATACTGTTGTGTTGTTGACTATAGAGTACTCATGGGCTCCATCTATATAGCATTAGTAGTTTTCCCTGAAGTTTGTTTTTCTCTGCTTACAGTCTGGGCCAGTGGCTCTATGATTTTCATCCTGTACAGACACAAGCAAAGGGTCCAACACATTCATATCACTAATGTTTCCTTAAGATCTCCTGAGTCCAGAGCCACACAAAGCATCCTTTTTCTGGCTATCACCTTCATATCTTTTTACACTCTGTCTTCAATCTTTCGAGTTTCTATTATTGTAGTTAATGATCACAGTTCATGGTTGGGTAGCACTGCTGATGTAATTTCTTTGTGTTTTCCAGCAATCAGCCCCTTTCTGATCATGAGCAAACTTTCCTCTATGTTTATACACTGCTTTGCAGGCATAAGAAGCAGAAAACTTTTCTAA

>Chinchilla_lanigera_intact_V1R4_30

ATGAGCCTTGCTCAGAGAAATGATTGTCAGCATAATTCAAAAGAAATTTTAGAAGGAAATGACAAAATAGCTTGCAGGGATTTGGCACTAGGAATACTCTTTTTGTCACAGACTGTGATTGGAATTCTGGGAAATTTTTCTCTTCTATACCATTATGTTTTCCTTCACCATAGGCAAAGCAGATTGAGATTTATAGATTTGATTCTCATGCACTTGTGTATAGCTAATTCTTTAGTTATTCTTTCAAATGGATCTTCCCAGACAATAGTAGCCTTTGGGTCGAAATATTTATTCAATGAATTTGGTTGCCATGTTACCTTGTACGTCTTCAGAGTGGGCACAACGATGTCAATTTGTAGTGTATGTTTCTTGAGTGTCTTCCAGACAATTATAATCAGCCCCACTAACTCCTGTTGGAAATATCATAAAATAAAATCTCCAAAATACATTGGCTTCATCATATCTTTCTGCTGGTTGCTACATATGGGAGTAAATTTCATTTTTCCTCTGTATATGTTACATGCATCTGGGAAACTAGAAAGCAGAAACATCACAAAGGAAAGACATCTGGGATTCTGTGCTGTTGTAGATTATGGGACTACCATGAACTCAGTCTATATTGCATTAGTAGTATTGCCTGAGGTTTCTCTTATTGTGCTAACAATATGGGCCAGTGGTTCTACTATTTTTGTCCTGTACAGACATAAACAGCAGGTTAAGCACATTCATTGCACTAATGTTTCCCTCAGATCCCCTGAGTTGAGAGCCACCAAAAGCATCCTTCTCCTGGCAAGCACATTTGTAACATTTTACATCATCTCCTCCATCTTTCACAGTTTCATTGCCCTTTTTTATAACCTCAGTTGGTGGTTGGTGAATATCTCTTGCCTCATTTCTCTGTGTTTTCCAACTATCAGCCCCTTTCTGGTCATGAGTCAGGATTCCTCTATATCCGTGTTCTGGTTTGTCTGGATAAGAAACACGAAACATCTCTTTTCAGCAGAAATATATAATTGTATATTTTCTCGATCCTTTGTGTTGTGTACTCCTCAATTCATTATGAAAGGCACTACCTAA

>Chinchilla_lanigera_intact_V1R4_31

ATGGCATCTAGGGACTCGGCAATAGGAATGACTTTTCTGACTCAGACAGTCACTGGGATCCTAGCTAATGTTGTCCTTCTTTTCCATTATCTCTCTCTCTGCTTCACTGGATACAAGTTAAGGCCAACAGATCTAATAGCTGAGCACTTGACTATAGCAAACACGGTGATCATGCTGTCTAAGAGAGTCTCACAGACAGTGCAATCATTTGGGATAAAGTATTTCGCTCAGGGTATTAGATGCCTGCTTCCTTTGTGTGTCTACAGAGTGGCCAGAGCTGTGTCAGTCAGTACAACCTGCCTCCTGAGCGTCTTTCAGACCGTGAAGATCAGCCCCATGAGCTCCAGCCGGAAGCAGCTTAAAGCTAACCTTCCCAAGCACATTGGCTTGTCTATTTTCCTCAGTTGGAGGCTCAATATGTTGGTAAATTGTTTTTTTCCCTTTTATAGCATCAACAAATATAGCAGCAAAAACATCACAAAGGAAAAAAAAGGATTCTGTTCTGCTGTCTTTAGAGACAAAATCGTAGATGTATTGTATACAACATTAGTGTTATTCCCTGAAGTTTCATGCTCTGGACTCATGATCTGGTCCAGCGGCTCCATGATTTTCATCCTGCACAGGCACAAGCAGCAGGTCCAACACATCCGAAGCACAACTGTTTCCCATAAATCATCCCCTGAGTCCAGGGCCACCCAAAGTGTCCTTGTCCTTGTGTGCAGCTTTGTGTGTTTTTACACCCTCTCCTCCTTCTTTTATGCTTATCTTGCACATTTCAATATTCCTAATAGTTGGCTCATGAACACCTCTGATCTGATTTCCACCTGCTTCCCTGCTGTCAGCCCTTTCATTCTAATGAGCTGCAACTCCACTGCGTCCAGACTCTGGTTCAAATTGTTAAATGTTTTCAGAAGCATATAA

>Chinchilla_lanigera_intact_V1R4_32

ATGAATCCTGTTGATTTTATCCTTGGGCTACTTTTCCCAATCCAGACTGGGGTTGGGGTGGCAGGGAACACCCTTCTTCTCTCAGTCTATGCCCCCACATCCTGTACTGCCCATGCACCGAGGCCCACGCACCTGATTCTCACCCACATGGCTGTGGCCAACTTCTTGGTTCTTCTCTTCAAGGGGATTCCCCATACGATGTTAATCTGGGGAATAACACCCATCCTGGGAAACACGGGATGCAGACTTGTGTACTATATCCACAGAGTGGCCCGGGGCCTTTGTCTCTGCACCACTTGCCTCCTGAGCAACTTCCAGGCCATCACCATCAGCCCGAGATCTGGAAGGAGGATGGGGCTCAAAGACCAAGCTCGTAAGAACATCAGTTTCTCCTGTGCTCTGTGTTGGATCTTCAACTTGCTGATAAATACCTTCATTCCTATAGATATTACGGGCCTGAAACATACCTACAATTCCACCAAGGTATGGAAATATAGTCTATGCTCTTCTAAAAATTCTGAAACACCTTCAAAATATACATTTCTATTGACCTTCCCAGATGTTGTGTTCCTGGGACTCATGGCTGGGGCCAGTGTCTATATGGTGCTTCTCCTATATAGACACCAGCAGACAGTAAAGCATATTCACACCACCAACAACTCCCACAGATTCTCCCCTGAGACAAAAGCCACTCAAACCATTCTGCTTCTAGCAAGCACCTTTGTATTGTTTTATTTAATCAATTCATTGATTACAATTTACCTTTCAAGTTTTTTAAAACCTCACACCGGGCTGCAGCATATCACTACTTTTCTGGCAGCCTGCTATCCCACCATCAGCCCACTGATATTGATGCTTTGA

>Chinchilla_lanigera_intact_V1R4_33

ATGTATTTCCGCATTATCAACACAATCTGGACTTTTCTAACTGGACTTGGCATATTAGGTAACACATTAGTTTTCATGTTTTATGTGTGTAATTTTGGGGAAGACAACAAGAACAAATCAATACAGCTCATTCTTATCCACTTAGATATCACAAATATGTTAATGCTTGTTACCCAAGGAATACCAAAGACAATACCAGTTTTTGGTGTGAGAAACTTCCTGGATGACATAGGCTGTAAGATTGTGTGTTACCTGCATAGGGTGGCCCGGGGCCTCTCCATCTGCACCAGCGCTCTCCTTACGGTGGTCCAGGCCATCACCATCAGTCCCAGACATTCCATGTGGAGGACGCTCAAGCCCAGGTCTTCATGGTACATCCTCCTTCTGCTTCTCTCTTTTTGGGGTCTCAACTCTTTGATAGCCATGAACTTACTCTATTCTATCACAAATGTCAGCATGAACTCATCAGAGCTTAGTAACAGTGAAAGATATTGTTATTTACTACCTGAAAGTGAGAAGACAAAGTGGATTTTCCTCACCTGCATGGTGCTACGGGATGCGGTGTTTCAGGGCGTCATGGGTGTGGCCAGTGGCTACATGGTGTGGGTTCTCTACAAACACCACCAGCAGGTTCTTCATCTGCAGAACTCCAAGTTCCTCTACACAGCTCCTCCTGAAATCAAAGCTGCTCAGAGTGTTCTCCTTCTGATGCTTGGTTTTCTTTTGTTTTATTGGATAGATTGTCTCCTTTCTCTATTTATAAGTTCCTCCTTAGTTACTCAGTCCACTGTCATAAGTGCTCGAGAATTTCTTGCTGTTGGTTATGCGGTTCTTAGTCCATTTGTACTGATTCACAGAGAGGGACACTTGGATCACTGCTGGAGAACTTGTGAGACAGAAAGACACTGA

>Chinchilla_lanigera_intact_V1R4_34

ATGAGTCATATTTCTCAAAGGAATGATTGTTATACTGATTTAAATGAAAATTTAAAAGGAAATGACAAAATAGCTGCCAATGATTTGGCAGTAGGAATAATCTTCATGTCACAGACTGTCATTGGTGTTGTGGGCAATTTCTTTCTTCTTTGCCATTATACTTTTGTTTTCCACACTGGAAGGAGGTTAAGGTACACAGATGTGATTCTGAAGCACCTTTTTGTAGCCAATGCTTTAGTTCTTCTTTCTAGAGGCATTCCTCACACAGTGGCAGCCTTTGGATTGAAACATTTCTTCAATGATTTTAAGTGTAAATGTCTCTTGTATATTGAGAGAGTGGGCAGGGGTGTATCTATTGGTACCGTGTGCCTCTTGAGTGTTTTCCAAAACATCATCATCAGCCCCATGGACTCCTGTTGGAAAGATGTTAAAGGTAAAGCTCAAAAGTATATCGGGTTCTCCCTTGGCTTCTGCTGGATCCTAAATGTGTTGGGCAGTTTAATTTTCCCTCTGTATGCATGGTATTCATCTGTAAAAGGTTATGACAGAATCATCAAAAAGAAAGTAAATATAGGATACTGTTGTCTTGTTGAGTATGGGGTAGTCATTCCCTCAATCTATATAGCATTAATAGTTTTTCCTGAAGTTTGTTTTTCTCTGCTTATAGTCTGGGCCAGTGGCTCTATGATTTCCATCCTGTACAGACACAAGCAAAGGGTCCAACACATTCATATTGCTAATGCTTCCTTAAGAGCTCCTGAGTCCAAAGCCACACACAGCATCCTTTTCCTGGTGAGCACCTTCATATCATTTTATACTCTCTCCACCATTATTCGAATTTCTGTTACTTTTGCTTATAATCTCAGTTTGTGGTTAGTGAGTGCTTGTGAGCTAATATCTGTATCTCAGTTTGTGGTTAGTGAGTGCTTGTGA

>Chinchilla_lanigera_intact_V1R4_35

ATGATTTGGAACCTTGTCAAGGGAATTATCTTCTTTTTTCCAACTGTACTTGGCATTCTGGGGAACGTCTTTGTGTTTGTGAATTACATTCTCTTCCTTGGAGGAACCGAGATGAAATCTATACACTTTATTCTCATCCACTTGGCTTTTGCAAATAGCATCATGCTTTTTGCAGAATGGGTTCCTAAGACAATAGCAGCTCTTGGGCAGCAAAACTTCCTTCATGACACAGGCTGTAAGATTGTTATTTACATGGTGAGGGTGGCCCGCGGTCTGTCCATCTGCACTAGCGGTCTCCTCACGGTGGTCCAGGCTGTTACCATCAGTCCCACAGACTCAATGTGGAGGAGGCTCAAGCTAAAGTCACCATGGCATATCCTTCCCATATTTGTCTTCTTTTGGATTCTCAATTCCTTAATAAGCATGAACTTACCATTTTCTGTCATAAGCATCAACAGTGTGAACATATCACAAATTAGTAAAGGTGACAAGTATTGCTATTTTCTACCTGAAGGCTGGATAATCAGATGGACATTTCTCACTCTGATGGTCCTACGTGATGCTGTGTTTCAGGCCATCATGAGTGGGGCCAGTGGATACCTGATATTTGCTCTCCACAAGCACCACCAACATGTTCTCTACCTGCAGAGCTCCAACGTCCTCTACAGAGCTCCCCCTGAGATCAAAGCTGCTCAAAGTGTGCTCCTTCTGATGCTTTGTTTTCTTGTCTTTTATTGGTCAGATTGTGCTTTTTCCATGTGTTTTAGTTATTCTCTCAATATTGATTCTGTGATATTAAATATTCACAAATATTTGACACTTGGTTATGCAATTCTGAGCCCATTTGTGCTGATTCACAGAGATGGACGCCTGGGTGCATGTTTTCACAGTCAAGAGGACAGAAAAGCACACAGAAATTGTCGGTCTTATTGA

>Chinchilla_lanigera_intact_V1R4_36

ATGGCATCTAGGGACTTGGCGATAGGAATGACTTTTCTGACTCAGACAGTCACTGGGATCCTAGCTAACATTGTTCTTCTTTTCCATTATCTCTCCCTCTGCTTCTCCGGATACATGTTAAGGCCCACAGATCTAATAGCTGAGCACTTGACTATAGCAAACACATTGATCATGCTGTCTAAAGGAGTCTCACAGACAGTGCAAGCCTTTGGGATAAAGTATTTCTCTCAAGGTATTATATGCACAGTTCTTTTTTATGTCTACAGAGTGGCCAGAGGTGTGTCAGTCAGTACAACCTGCCTCCTGAGTGTCTTCCAGACTGTGAAGATCAGCCCCATGAACTCCAGTTGGAAGCAGCTTAAAGCTAAACTTCCCAAGCACATCAGCTTCTCTATTTTCCTCATTTGGAGTCTCTATACGTTGGTAAATGGTATTTTTCCATTTTATAGAAGCCACAAATATAGTGGCAAAAATATCACAAAGAAAAAAGAATATGGATTCTGTTCTACAGGATTTCGAGACAAAATCATAGAGGCACTGTATGCAGCATTCATATTATTCCCTGAAGTTTCATTTTCTGGGATCATGATCTGGTCCAGTGGCTCCATGATTTTCATTTTGCACAGGCACAAGCAGCAGGTCCAATACATCCTTAGAACAAATGTTTCCCACAAATCATCCCCTGAGTCCAGGGCCACCCAAAGTGTCCTTGTCCTCGTATGCACCTTTGTGTCATTTTACACCCTCTCCTCCATCTTTTATGCTTGTGTTTTGAATTTCAATATTCCTAGTTGGTGGCTCATGAACACCTCTGACTTGATTTCCACCTGCTTCCCTGCTGTAAGCCCTTTTGTTCTAATGAGCTGCAACTCCACTGCATCCAGACTCTGGTTCAAATTCTCAAGTTTTGTCAGAAGCATGCAAAGTGCATTCTTTTGCACAAAGGTGAGTGGTTGA

>Chinchilla_lanigera_intact_V1R4_37

ATGGGGTCAGAGGATCATCCAATGCATTCAATATTCAGGATAATATTCTTTGCTCTTATTGGACCAGGCATTGGAGGAAATGCCCTTTTGTTTGTGAAGCATGTGTCCCTGTCCATCATGGGGCTTCAGAAAAAGCCCGTAGACCTTATTCTCATTCAACTGGCCTTTGCTAATGTGCTGACACTTTGTGTTGCGAGAATTACAGAAAGAAGATCACCTTTTCATTCTGATAACTTCCTAAGTGATATTGGATGTAAAATTGTGGTTTACATGGAGAGGGTGGCCTGGGGTCTCTCCATCTGCACCACCTGTCTTCTCAGCATGGTCCAGGCCACCACCATCAGTCCCAAGACCATCCTCTGGAGAAAGCTTAAACCACAGACTACACGGCAAGTCCTTTCCTGTCTTCTCCTCTGCTGGATCATTAATCTTCTGCTAAGCTCCAATTTGCTCCACTACATCACAGCAGTCAGTAGCATGAACAGATCTGAAGTTGGAGTGTATGCTGGACCTTGTTACTTACTATCATCTGGACAGACAGTCCGCTGGCTTTTCCTCTCTCTCATGGCTCTTCGGGATGTCCTCTTTCAGGGTCTCATGGGCTGGAGCAGCGGACACATGGCTTTCCGCCTGTACGAACATCACCAGCGTGTGCTCTACTTGCACAGCTCCCGACTGGCAGTGAATTCCAGCCCCGAAATCAGAGCTACACTGAATATACTCATTCTCATGGCCTGTTTCCTTTTGTTTTATTTGGCAGATTTCATTTTCTCCTTTTACATAGGGTCCATGGTAACCCGTGACTTCACCATACTAAATATTAAAATATTTCTAGGACTTGGTTATGCAGTTCTTAGTCCCTTTGTGCTGATAAGCAAGGATGTCTACCAGGTTACATGCTGGCATAGTGACTGA

>Chinchilla_lanigera_intact_V1R4_38

ATGATTCCAGAGCACTTAGTAATGGGTATTTTCTTCTTCTCCCAGACAACTGTGGGAATTTTAGGCAATTGGTCACTTCTGTTTCATTATTTCTTCTCTGTATATACGGGAAAGAATTTGACACCCACAGACCAGATTATAACGCACTTGACCTTTGCCAATTCCTTGGCTATTATGTCAAGAGGAATTCCTCAGACAATGAGACAATTTGGGTCGAAATATTTACTGGATGACACTGGATGTAAACTTACTCTATACTTGAACCGCATATCCCGAGGGATTTCCCTGAACACCACCTGCCTCCTGAGTTGCTTCCAAGCACTCACAATCAGCCCCAGAAGCGGCAAGTGGGTGGCATGGAAACACAGAACCACCAAGTACATCAGCCCCTGCTGTTCCCTCGGCTGGCTCATACATCTGCTTCTCAACATCATGATTACAATGCGAGTGACTGGTTATCCCAACAGCGAGAATTTCACTAACAGATTTCATTATGGGTTCTGCTCAGGGTTTGTTTCCGGCACCATAACAACTCCACTATACGTGTCCCTGCTCTGCTGCACAGATGGTCTCTGTTTGGGTCTCATGGCCTGGGCCAGTGGCTCCATGGTGAGCATCCTCTATGGGCACAAGAGGCAGGTTCGGTATATCCACAGTGCCCACCGCTCCCCTCGAGTCTCCCCGGAGGCCAGAGCCACCCAGACCATCCTGCTCCTGGTGTGCACCTTTGTGACCTGCTACTCCCTCTCTTCCACCTTGGTAGTGTATGTAACTTTCTTTGAGAATCCGAGGCTATGGGTGATAAACATCTTTGCATTTCTAGAAACATGTTTCCCCATGTTTTGCCCCTTTGTCCTCATCAATAATAACTCTACTTCCAGGTCCTATTTTTCCTGCTGGGGGAGTAGGTAG

>Chinchilla_lanigera_intact_V1R4_39

ATGGCCTTCAGGGATTTGGCAATGGCAATGGTCTACTTCTCTCAGACTCTCATTGGAACAGTGGGGAATGTCCTTCTTCTTTACCACTGCAGCTGTCTTCATTTCACTAAGTCCAAGTTAAGGCCCACAGATCTGATTCTCAAGCACCTGCTTGTAGCCAACTCCTTGGTCATTCTGTTTAGAGGAGTTCCTGAAACAATGGCAGCATTTGGCCTGAAAGATTTTCTAAGTGACCTGGGATGCAAACTTGTTCTCTATGTTCACAGAGTAGGCAGAGGTGTGTCCATTGGCAGCACCTGCCTCTTGAGTGTTTTCCAGGTGATCACGATCAGCCCTAGGAACTCCAGGTGGACAGATCTTAAGTTAACAGCCCCTGAGTATGTAAGCATCATCAGTATCCTCTGTTGGATCCTCCACATGCTGTTAAACACAATTGTTCTTATGTATGTTACTGACAGACGGTGCCATAAAAACATCACATACAGAAAAGAGTTTGAATACTGTTCTACTATTCGCCAACAAAAATCTAGGACAATCCTGCATGCAGCACTGCTGTTCCTCCCTGATGCATTGTGTGTGGGGCTCATGCTCTGGACCAGCAGCTCCATGATTTCTATTCTGTACAGGCACAAGCTGAGAATGCAACATGTTCGCAAGACCAACTTCACACAGAGATCTTCCCCTGAGACCAAGGCCACCAAAACCATCATTCTCCTGGTCAGCACCTTCGTCTGTTTTTATTCCCTTTCATCCATTTTCCAAGCTTTGTTCACTTTGTATAACAATCCTGACAGGTTCCTCGTGAACGTTGCTGCAGTAGTCACTGGCGGTTACCCAACTGTCAGCCCTTTTCTGCTCATCAAACATTTCCCATGCATATCTAATCTCACTGACTGTGTAAGAAATAAGAAAACCTATAATGTTAAGAGGATCATGCAACTTTCTTAA

>Chinchilla_lanigera_intact_V1R4_4

ATGGCATCTAAGGACCTGGCAATGGGAATGACTTTTCTGACTCAGACAGTCACTGGGATCCTAGCTAATGTTGTTCTTCTGTTCCACTATTTCTCTCTCTGCTTCACTGGATACAGGTTAAGGCCCACAGATCTAATAGCTGAGCACTTGACTATAGCAAACACAGTGATCATGCTGTCTAAGGGAGTCTCACAGACAGTGCAAGCCTTTGGGGTAAAGTATTTCTCTCAAATTACTAGATGCACAGTTCTTTTGTATGTCTACAGAGTGGCCAGAGGTGTGTCAGTCAGTACAACCTGCCTCCTCAGTGTCTTTCAGACCGTGAAGATCAGCCCCATGAGCTCCAGATGGAAGCAGCTTAAAGCTAAACTTCCCAAGCACATTGGCTTGTCTGTTTTCCTTTGTTGGAGTCTCAATATGTTAGTAAATTGTATCTTTCCCTTTTATAGCATCAACAAATACAGCAGCAAAAATACCACAAAAATAAAATATTTTAGATACTGCTCCTCAGGATTTCCAGACAAAATCACAGAAGCATTGTATGCAGCATTCGTATTATTCCCTGAAGTTTCATGTTCTGGGATCATGATCTGGTCCAGCAGCTCCATGATTTTCATTCTGCACAGGCACAAGCAGCAGGTCCAACACATCCGAAGGACAAACGTTTCCCACAAATCATCCCCTGAGTCCAGGGCCACCCAAAGTGTCCTTGTCCTCGTGTGCAGCTTTGTGTGTTTTTACACCCTCTCCTCCTGCTTTTATGCTTCTGTTGTGAATTTCAGTATTCCTAGTTGGTGGCTCCTGAACACCTCTGCCCTGATTTCCACCTGCTTCCCTGCTGTCAGCCCTTTTGTTCTAATGAGCTGCAACTCCACTGCGTCCAGACTCTGGTTCAAATTCTCAGGTTTTGTCAGAAGGAGGTAA

>Chinchilla_lanigera_intact_V1R4_40

ATGGCATCTAAGGACTTGGCAATAGGAATGACTTTTCTGACTCAGACAGTCACTGGGATCCTAGCTAACGTTGTTCTTCTGTTCCATTATCTCTCTCTCTGCTTCACTGGATACAAGTTAAGGCCCACAGATCTGATAGCTGAGCACTTGACTATAGCAAACACATTGATCATGCTGTCTAAAGGAGTCTTACAGACAGTGCAAGCATTTAGGATTAAGTATTTCTCTCAAGTTATTAGATGCACAATTCTTTTGTATGTCTACAGAGCAGCCAGAGGTGTGTCAGTCAGTACAACCTGTCTCCTGAGTGTCTTCCAGACCGTGAAGATCAGCCCCATGAACTCCATTTGGAAGCAGCTTAAAGTTAAACTTCCCAAGCACATTGGCTTCTCTATTTTCTTCATTTGGAGTCTCTATATGTTGGTAAATTGTTTTTTTCCATTTTATAACATCAGCAAATATAGCAGCAAAAATATCACAAAGATAAAAGATTTTGGATACTGCTCTGCAGGACTTCGAGACAAAATCGTCGATGCATTGTACGCAACATTTGTGTTATTCCCTGAAGTTTCATGTTCTGGGATCATGATCTGGTCCAGTGGCTCCATGATTTTCATTTTGCACAGGCACAAGCAGCAGGTCCAACACATCCGTAGAACAAATGTTTCCCACAAATCATCCCCTGAGTTCAGGGCCACCCAAAGTGTCCTTGTCCTCGTGTGCACCTTTGTGTGTTTTTACACACTCTCCTCCTTCTTTTATGCTTGCGTTGTGAACTTCAGTATTCCTAGTTGGTGGCTCATGAACACCTCTGCCCTAGTTTCTGTCTGCTTTCCCACTGTCAGCCCTTTCATCCTAATGAGCTGCAACTCCACTGCATCCAGACTCTGGTTCAAAATCTCAAATTTCTTTAGAAACATAAAATGCACCACTTTTCACAAGGTTAAGTGA

>Chinchilla_lanigera_intact_V1R4_5

ATGACAAAAACAGAACCCATGTATTTGGCATTAGGAATGATCTTCTTGTTGCAGACTATTGTTGGAGTCCTGGGCAATCTTTTTCTTCCTTACCATTATCTTTTCCTTTACCATAGTAAAAGCAAGTTGAGATACATAGATTTGATTCTCAGGCACATATTTATAGCCAACTCCTTACTTATTCTTTCTAAAGGACCTCCCCAGATAATTGTAGCCTTTGGACTGAAACATTCTTTCAACGATTTTGTCTGCAAACTTATCTTGTATGTTGAAAGAGTGGGCAGGGGTATGTCGATCGGCACCATCTGCCTCTTGAGTATCTTCCAGACTATCATGATCAGCCCCACAAACTCCTGCTGGAAAAATCTTAAAATAAAAGCTCCAAAGTATCTTGGCTTATCCATTTGCCTCTGCTGGTTTCAACATACAGTGGTAAATTTCATCTTTCCCATTTATCTGCTGTCTATGTCTGCAAAACGGTATAGCAGAAACATCACAAAGAGAAGAGAAATGAGACTCTGTCCTCTTGTAGATCACGGGAAAATCCTGGGCTCAGTCTATATGGCATTAGTAGCATTCCCTGAAGTTATTTTTTCCATACTTATGATCTGTGCAAGTGGCTCCATGATTTTTACTCTGTACCAACACAAGCAGCGGGTCCAACACCTTCACAGGAAAAATGTTTTCTCCAGATCCCCTGAGTCCAGAGCCACCAAAAGCATCTTTCTTCTAGTGGGCACTTTTGTATCATTTTACAGCATGTCCTCCATCTTTAACTTTTCCATTGCTTTTTTTCCTGAGATATATTGGTGGCTGGTGAGCATTTCTGATGTAATTTCTTTGTGTTTCCCAACTATGAGCCCCTTTCTGATAATGAGCCAGGATTCCTCTATATCCAGGCTCTGCTTTTTTTGGATAAGGAACACTCTCTCCCCTTATAAGAAATAA

>Chinchilla_lanigera_intact_V1R4_6

ATGGCCTTCAGGGATTTGGCAATGGCAATGGTCTACTTAGTTCAGACTCTCATTGCTACACTGGGGAATTTCTTTCTTCTGTGCCATTGTACCTGCCTTCATTTCACTAAGTGTCGATTGAGGTCCACAGATCTGATTCTCAAGCACCTGGTCATAGCCAACTCTTTGGTCATTCTGTTTAGAGGAATTCCTGAAACAATGGCAGCATTTGGCTTGAGAGATTTCCTCAGTGACCTGGGATGCAAACTTGTTTTCTATGTTCACAGAGTAGGCAGAGGTGTGTCCATCAGCAGTACCTGCCTCTTGAGTGTCTTCCAGGTGATCACAATCAGCCCTAGGAGCTCCAGGTGGGCAGAGCTTAAGCTAACAGCCCCCAAGCATGTAAGCACCTTCAGCATCCTCTGTTGGATCCTCCACATGCTGTTAAACACAATTGTTCTTATGTATGTTACTGACAGATGGGGCCATAAAAACATCACCTACAGAAAAGAGTTTGGATACTGTTCTACTATTCGCCAACAAAAGTCTAGGACAATCCTGCATGCTGCAGTAATGTTCCTCCCTGATGCATTGTGTGTGGGGCTCATGCTCTGGACCAGCAGCTCCATGATTTCTATCCTGTACAGGCACAAGCTAAGAATGCAACATGTTCACAAGTCCAACTTGACACACAGATCCTCCCCTGAGACCAGGGCCACCAAAACCATCATTCTCCTGGTCATCACCTTCGTCTATTTTTATTCCCTCTCTGCTATTTTCCAAGCTTTCTTCACTTTGTATAAAAATCCTAACAGTTTGCTTGTGAATGTTGCTGCTCTAGTCAGTGGGGGTTACCCAACAGTCAGCCCTTTTCTGCTCATGAAACATCACCCAAGCATATCCAGTCTCCTTGACTGTTTAAGGAATGCAAAAACTTGTAATGTTATGAGGATCATGTAA

>Chinchilla_lanigera_intact_V1R4_7

ATGTCACCCAACAGCCTGACAATCAGAATCATCTTTGTTGCTCAGACAGGAGTTGGAATCCTGGGAAATATTTTGCTCCTTTATCATTACGTCTTCATTTCCTACCCTGGACGAAAGCTAAAACAGGTGGATTTTATTCTCAAAAACTTAATTTTGGCCAACTGTTTGGTTCTTCTCTCCAGAGGAATTCCTCACACAATAATAGTCTTGGGGCTGCAATATTCTATGGGTGATATTGGGTGCAAACTGGTCTTCTATCTTCACAGGGTGGCCCGAGGTGTGACTCTTGGTACTACTTGTATCTTGAGTGGCTGCCAGGCCATCACAATCAGCCCTAGCCATCACCAGTGGGTGAAACTCAAGGCCAAAGTGCCCAAGTACATGAGATTTTCTGTTACCTTATGCTGGATCCTTCACATGCTGGCAAACAGTGTTTTTCCTATATTTATCACAGGTGCGAAGGAAAGCAGCAACCACACACATTACAGAGACCTTGGGTCTTGTTCCAGCACAAGTAGCGAGAGAGTGGCATCCTTATTGCACTCTGTGATATTGTCTTCCATTGATGTGTTGTCTTTGGGGTTTATGTTGTGGGTCAGTGTTTTCATGATGCTCATCTTGTATAGACATAAGCAGAACGTCCTACACATTCATAGCAAGAACCTCTCTTCCAAATTCTCTGCTGAGACCAGAGCCACCCAGACCATTTTGGTACTACTAATCACATTTTTCTCTACATACACCCTGTCTTCGTTCTTCACCTTTTACATGTCTTACTTTGATAGACCCCGTCGGTGGCTGGTAGACACCAGTGCACTGTTAGCTGCCAGCTTCTCCTGTGTCAGCCCATTTGTGCTCATCAGCAGGGACCCACATTTCTCTTTGCTCTGTTTTGCATGCTGCCCAAAATAA

>Chinchilla_lanigera_intact_V1R4_8

ATGTATTTCCACATTATCAGCACAATCTGGGCCATGCTAACGGGACTTGGCATATCAGGTAACACATCGGTTTTTGTGACTTATATGTGCAATTTGGGGCAGGACACCAAGAACAAATCAATACAGCTCATTCTTATGCACTTCATTTTCACAAATACCTTAATGCTTGTTTCCAAAGGAGTGCCAAAGACAATAGCAGCTTTTGGCGTGAGAAACTTCCTAGATGACATAGGCTGTAAGATCATCTGTTACCTGGAGAGGGTGGCCCGGGGCCTGTCCATCTGCACCAGTGCTCTCCTCACGGTGGTCCAGGCCGTCACCATCAGTCCCAGACGCTCTGGGTGGAGGAAACTCAAGCCCAGGTCTGCATGGTGCATCCTCCTTCTGCTTCTCTCTTTTTGGGGTCTCAACTGTTTGATAGCCACCAACTTACTCTATTCTATCACAAATGTCAGCATGAATTCATCACAGATTAATAACAGTGAGAGTTATTGTTATTTTCTACCAGAAAGTGAAAACGTAAAGTGGATTTTCCTCACCTGCATGGTGCTACGGGATGCGGTGTTTCAGGGCGTCATGAGTGGGGCCAGTGGCTACATGGTGTGTGTTCTCCACAAACACCACCAGCAGGTTCTTCATTTGCAGAACTCCAAGTTCCTCTACACAGCTCCTCCTGAAATCAAAGCTGCTCAGAGTGTTCTCCTACTGATGCTTGGTTTTCTTTTGTTTTACTGGATAGATTGTCTCCTTTCTCTATTTATAAGTTCCTCCATAGTTTTTCAGTCTACTGTCATAAGTGCTCGAGAATTTCTCGCTGTGGGTTATGCGGTTCTTAGTCCATTTGTACTGATTCACAGGGAAGGTCACCTGGGTCATTGTTGGAGCCCTTGTGGGACAGAGAGACAGTGA

>Chinchilla_lanigera_intact_V1R4_9

ATGGGTTTTAACTTTGTTAAAGGAGTAATTTTCTTCTTTCTAACTGGACTTGGCATGACAGGAAACATCTTTGTTTTTATGAATTGTACATGCTTTATTGGAGGCACTAAGATGAAAAGTACACACTTCATTCTCACCCATTTGGTTTTTACAAATATTATCCTGCTTCTTTCCAAGGGAATTCCAAGAACAATAGCAGCTTTTGGTTTCAGAAACTTCCTCAGTGACACTGGCTGTAAGGTTGTTGTTTATCTCGGGAGGGTGGCCCAGGGCCTCTCCATCTGCACCAGCAGTCTCCTCACTGTGGTCCAGGCCATCACCATGAGTCCCAGACACTCAGCGTGGCGGAGGGCCCAGCCCAGGTCTGCATGGCATGTCCTCCCCTTGCTCCTCTCCCTGTGGGGCCTCAATGCTATGGTAAGCATGAACTTACTCTATATCGTCTCGAGTTCTAATATGACCTCATCACAAATTAGTAAAAGTGACGTCTATTGTGATTTTCTACCAAAAAGTCAGAAAGTGAACTGGATTTTCCTCACTCTGCTGGCCCTGAGAGACACTGTGTTTCAAGGTCTAATGGGTGGGGCCAGTGTCTACCTGGTGACTCTTCTCCACAAGCACCACCAGCGTGTTCTCCACCTGCAGAAGTCCAAAGTGGTCTGCAAAACTCCCCATGAGATCAAAGCTGCCCAAAGTGTGCTCCTTCTGATGCTTTGTTTTCTTTTCTTTTATTGGACAGATTGTTTCACATCTTTATATTTAGCTTTCTCCTTCAAGAGTGACTCCATAACAATAAATCTTCAAGAATTTCTGACTTTCGGTTATGCAATCCTCAGTCCTTTTGTGCTGATTCACAGGGATGGGTTCCAGGCTAAGTGCTGGCATGCTCTGCAGGGTGGAAAGACATTGAGAAATTGTCTATTGCATTCTCCTTTCTATAGGTGGAAATGA

>Chinchilla_lanigera_intact_V1R48_1

ATGAATGTGAAGAATGAGTTGTTCAGTAATAATAACATCAGAATCACATTTTATTCTCAAGTTGTCATTGGGGTCTCAGGCAATGCCATCCTTCTTCTCTTCCACGTCATCATATTTCTTGCAGGGCACAAGCCCAGACTCACCGACCTGCCCGTTGTCCTCTTGGCTCTAAATCACCTAGTGATGCTGCTAATTGAAGGATTCATAACTGGAGACATTTTTACCTCCCAACCAGGGTTTTGGGATGACATCACATGTAAATCAGTTATCTACTTGAACAGATCCATGAGGGGCCTCTCCATTTGCATCACCTGCCAGCTGAGTGTCCTTCAGGCCATCACACTCAGCCCCAGAAGCTCTTGTTTGGCAAAGTTCAAACACAAATCTTCATATCATAACCTGCGTTCCCTTCTTTTCTTCTGGGTGTTCCATATATCCATTAGCAGTCACCTTTTTATCTCCATCACTGCTACTCCCAATTCAACTTCAGAAAATATTTTGTATGTCACTGAGTCTTGTTCTCTTTTACCCATGAGCTATTTACTCCAGCACATATTTTTCATATTATTGACCTTCAGGGATCTCTTCTTCATGGGGCTCGTGTCTCTCTCAGGTGGATACATAGTGACTCTCTTGTGCAGGCATAAGATGCAGTCGCAACATCTTCACAACAACAGACTTTCTCCAAAAGGATCCGCAGAACAAAGGGCCACTAGGACCATTCTGCTGCTTCTGTGTTTCTTTGTAGTCATGACCAGTTTGGACACTCTAACCTACTCAAGAATTGTGTTGAATAATAATCCAATAATTTATTGTATTCAAATTCTTATGGCCCATGGCTATGCAACAGTCAGTCCTTTAGTGTTCATCAGTACTGAAAAACGTATCATTGATATTTTGAGACTCATGTGTGGAAGAAAATAA

>Chinchilla_lanigera_intact_V1R48_2

ATGAATGTGAAGAACGAGTTGTTCAATAATATTAACATCAGAATCACATTTTATTCTCAAGTTGTCATTGGGGTCTCAGGCAATGCCATCCTCCTTCTCTTCCACGTCATCATATTCCTTGTAGGGCACAAGCCCAGACTCACCGACCTGCCCACTGTCCTCTTGGCTCTAAATCACCTAGTGATGCTGCTAATTGAAGGATTCATAACTGGAGACATTTTTACCTCTCAAGCAGGGTTTTGGAATGACATCACATGTAAGTCAGTAATCTATGTGTACGGGTTAATGAGGGGCCTCTCCATTTGCATCACCTGCCAGCTGAGTGTCCTTCAGGCCATCACACTCAGCCCCAGAAGCTCTTGCTTGGCAAAGTTCAAACACAAATCCTCATGTCACAACCTGCGTTCCCTTCTTTTCTTTTGGCTGTTATACGTATCTGTTAGCAGTCACCTGGTGTTCTCCATTGCTGCTACCCCCAATTTTACCTCAGAAAATATTTGGTATGTCACTGAGTCCTGTGCTTTTATTCCCATGAATTACTTCCTCCGGCACATACTTTCAACACTGCTGGCCTTCAGGGAAGCCTTCTCTGTAGGTGTCCTGGCCCTCTCGGGTGGGTACATAGTGACTCTCTTGTGCAGGCATAAGAAGCAGTCCCAGCATCTTCACAACAACAGACTTTCTCCAAAAGGATCCGCAGAACAAAGGGCCACTAGGACCATTCTGCTGCTTATGTGTTTCTTTGTAGTCATGACCAGTTTCGGTACTGTTACCTACTCAAGAATTGTGTTGAATAATAATCCAATATTTTACTGTATCCAAATTCTTATGGCCCATGGCTATGCCACAGTCAGTCCTTTGGTGTTCATCAGTACTGAAAAACGTATCATTAATTTTTTAACATTCGTATGTGGAAGACAATAA

>Chinchilla_lanigera_intact_V1R642_1

ATGGTTTATAAAATGTTTCACAATTCAGTAATTTTTTCCTTTTTTGTCATATTTGAGACTTGTTTTGGTGTCACTGCAAACTCATCACTCTTTGTGGCATTTATATACACCTTCTTAACTCAACCTAATCTTATGAGACCCATAGATTTCATTATAATTCACCTGACAGTGGTCAATAATTTGACGATCTTAGTCACACTGATACCATATATCAAGGCATCCTTTGGAGTGAGACAGTTTTTGGATGATACAGGCTGTCAGGTAATGTTGTATACAGGCAGAGTTACCCGGGGTGTTTCCATCTGTACTACCTCACTTGTGAGTATATTTCAAGCCATCACTATCAGTCCCCTTAATTCTAAGTGGGCATGGCTTAAGTCTAAACTCTCTGCAACAATAATGCCTTCTTTGCTTATCTTCTGGATCATCAACTTGCTTATTTTTATCCGTGTCATTGAAACCATAAGTGCAATTGGTAATTTTTCTCTTGTTGGCTATGGGTATTCTAATATTTACTGCCAAACAGGAGAGAAAACCATCAGATCTTCAGCCTCATTTATAAGCCTCATACTGAGCCATGATATCATCTTTTTGATCTTCATGCTGTCGACCAGCTTCTACATGGCCAGTTTCCTCTACAGACACCACAGGAGAGCACAGTATGTCCATAAACCCTCTTCCCAGACAGCTCCCGAAATCAAAGCAACTCAAAACATCCTTTTGCTGGTAAGTTTCTTTGTTTTCTTCTATTGTTCAAACAACTTTGTCACTTTGTTTACATTTTATTCACACAAGAAAATTCCTGCTTTGGAGGGGATTAGTGGGATGTTAGCATCATGCTATCCATCTATCAGTCCTCTGTTTTTTATGAAAAATAATAAAATGCTTTCCAATTTTTTCCATTTTACAATGAGGAGGTTTATGTGTCCTCAAACGGTTTTCAGTGGCTAA

>Chinchilla_lanigera_intact_V1R642_2

ATGTTTGATAATTCAGTAATTTTTTCATTTTTTGTCATATTTGAGACTTGTTTTGGTGTCACTGCAAACTCATCGCTGTTTGTGGCATTTATGTACACCTTCTTAACTCAACCTAATCTTATGAGACCCATAGATTTCATGATAATTCACCTGACAGTAGTCAATAATTCGACTATCTTAGTCACACTGATACCATATATCAAGGCATCCTTTGGAGTGAGACAGTTTTTGGATGATACAGGCTGTCAGGTAATGTTATATGTATACAGAGTTACCCGGGGTGTTTCCATCTGTACTACCTCACTTGTCAGTACATTTCAAGCCATCACTATCAGTCCCATTAATTCTAAGTGGGCATGGCTTAAGTCTAAACTCTCTGCAATGATTAAGCCTTCTTTGCTTATCTTCTGGATCATCAATTTGCTCATTTATATTCATGTTATTGAAAAAATAAGGGCAATTGGCAATTATTCTTTTGTTGGCCGTGGGTATTCTAATATTTACTGCCAAACAGGACGGATGACCGGCAACTCTTCATTCTCATTTATAAGCCTCATACTGACCCATGATATCATCTTTTTGATCCTCATGCTGTCGACCAGCCTCTACATGGTCTGTCTCCTCTACAGACATCATAGGAGATCCCAGTATGTCCATAAACCCTCTTTGTGGACAGCTCCCGAAATCAAAGCTACCCAAAACATTCTTTTGCTGGTGAGTTGCTTTGTTTTCTTCTATTGTTCAAACAACTTTTTCACTTTATTTACCTTTTACTCACATAAAAAAAGTCCTTCTTTGGGGGGGATTAGTGGGATGTTATCATCATGCTATCCAACTATCTGTCCTTTGTTTTTGATGAAAAATAATAAAATATTTTCCAAATTTTTCCATTTTACATAA

>Chinchilla_lanigera_intact_V1R642_3

ATGTCCCACAATGCAGTGTTTTTTGGTTTTTTCCTTATATTTCAGACCTGTATTGGTGTCATGGCAAACTCGTTACTGTTTGTGGCATTTATGTACACCTTCTTAACTCAACCTAATCTTACAAGGCCCATAGATTTCATTATGATTCACCTGACAGTGGTCAATAATTTGACTGTCATAGTCACACTGATACCATATATTAAGGCATCCTTTGGAGTGAGACAGTTTTTGGATGATACAGGCTGTCAGGTAATGTTGTATGCATACAGAGTTACCCGGGGTGTTTCCATCTGTACTACCTCACTTGTGAGTACATTTCAAGCCATCACTATCAGTCCCATTAATTCTAAGTGGGCATGGTTTAAGTCTAAACTCTCTACAATGATTAAACCTTCTTTGCTTGCCTTCTGGATCATCAATTTGCTCATTTATATTCGTGTTATTGAAACAATAAGAGCAACTGGAAATTTTTCTCTTGTTGGTCATGGGTATTCTCATGCATATTGCCAAACTGAACCGATGACACCTACCCAATTGTGGTCATATATAAGCATCATATTGACCCATGATCTCGTGTTTTTGATGCTCATGCTGTCAACCAGCCTCTACATGGTGAGTCTCCTCTACAGACACCACAGGAGAGTCCTGTATATTCGTAGCACCTCTTCACAGACGTCAATAGAAACCAAAGCAACTCGAAACATTCTTTTACTGGTGAGTTGCTTTGTTTTCTTCTATTGCTCAAACAACTTTGTCACTTTATATTCCATTTCTACACACAAGAAAATTCCCGCTTTAGAGGGAGTTAATGCAGTGCTAGCATCATGTTATCCAACTATCTGTCCTTTGTTTTTGATGAAAAATAATCAATTATTTTCCAAATTTTTCTTTTTTACAGTGTTGAGGTTTATCTGTCCTCAAACTGTTTTCAGTGGCTAA

>Chinchilla_lanigera_intact_V1R664

ATGCTATCTTATATTGTCAAAGGAACAATCTTTGCCTTTCTAACTGGACTTGGCACTATGGGGAACATCTTTGTTTTTGTGAATTATGTGGTCATACTTTCAGGCAATGAGAAGAAATCTATACACCTTATTCTAATGCATTTAACTGTTACAAATACTATAACTCTTCTTTCCAAGGGAATGCCAACAACAATAGCTGCTTTTGGTGTGAGAAACTTCCTAGATGACAAAGCCTGTAAGATTATATGTTACCTGGAACGGGTGGCCCGAGGCCTCTCCATCTGCACCAGTGCTCTTCTCACAGTGGTCCAGGCCATCACCATCAGTCCCATACACTCTGCGTGGAGGAGGCTCAAGCCCAGGTCTGCATGGTGCATCCTCCCTGTGATTCTCTTTCTCTGGGGTCTCAATTGTTTGATAGCCATAAATTTACTCTATTCCATCACAAATATCAGCATGAACTCATCACAGATTAGTAACCATGAAAGATACTGTAATTTTTTACCAGAAAGTAAAAAGGTAAAGTGGATTTTTCTCACTGGCATGGTCCTACGGGATGCGGTGTTTCAGGGCATCATGGGTGGGGCCAGTGGCTACATGGTGTGTGTTCTCTACAAACACCACCAGCAGGTTCTTCATTTGCAGAACTCCAAGCTCCTCCACACAGCTCCTCCTGAAATCAAAGCTGCTCAGAGTGTTCTCCTTCTGATGCTTTGTTTTCTTTTGTTTTACTGGATAGATTGTCTCCTTTCTCTATTTATAAGTTCTTCATTAGTTACTCAGTCCACTGTCATAAATGCTCGAGAATTTCTCACCCTTGGTTATGCAGTTCTTAGCCCATTTATACTGATTCACAGGGAGGGTCACCTGGGTCATTGTTGGAGCAGTTGTGGGACAGAAGGACACTGA

>Chinchilla_lanigera_intact_V1R90_1

ATGATGGTGAAGAAAAAGAGAAAGTTTTTCAAGATTTTTGATATGAGAAGTGCAGTTTTCTTTGAAATCACCGTTGGGATCTTAGCCAACAATGTCCTGCTTCTGTTCCACATCCTCACATTTCTCCTCAAGCACAGGCCCAAGCCCCTGGACCTCACTATCGCGCACCTGGCCCTGATCCACTTGGTGATGCTGGTAACCATGGGCTTTATAGCTGCAGACACATTTGAGTTTCAGGGTTGGTTGGATGGCCTCATGTGTAAATTGGTTATCTATGTGCACAGGTTGATGAGGTCCCTCTCCATCTGTACCACCTGCCTGCTGAGCATCCTCCAGGCCATCACCCTAAGCCCCAGAAACTCTTGTTTGGAAAAATTCAAACATAAATCCTCACATCACTACCCATGTTGCCTTGTGTTTTTATGGGTCTTCAATATTCTCCTGAATACTCGTTTCTTAGTCTCCATTGATGCCACCCCCAATGTGACCTCACACAGTCTCCTGTTTGTCACTGAATCTTGCTCTCAGTGGCGAATTAGTTACTTGTTCAGGAACATATTTTTGTCACTGGTGAATATTCAGGATATCTCCTTCATAGGGCTGATGGCTCTCTCAAGTGGATACATGGTGAGCCTGTTGTGCAGGCATAAGAGGCAGTTCCAGCACCTTCACAGCACCAATCTTTCTCCAAAAGCATCTCCAGAAGAAAGGGCCACTCAGAGCATCCTGCTTCTCATGGGTTTCTTTATGCTTATGTACTTTTTGGACTGTGTTGCTTTCTCATCCCGTGCAATATTGTGGCACAATGACCCAATTAATCTTTGTGTCCACATGCTTGTGGGCAATGGCTATGCCACAATCTGTCCATTTGTGCTAATGAGCACTGAAAAGCGAATGACTGAGTGCTTAACATCCAGGTGGAAAAGACTATGA

>Chinchilla_lanigera_intact_V1R90_10

ATGAAGATGATTAAAAAGAGAAAATTTTCCAGTTATACTGGCGTAAGAAACATTGTTTTCTCTGAAGTCTCCATTGGCATCGTAGCCAACACTGTCCTGCTTCTGTTCCACATTCTCACATTTCTCCTCAAGCACAGGCCCAAGCCCCTGGACCTGACCATCAGTCAGTTGGCCCTGATCCACCTGGTGATGCTGGGAACCATGGGCTTCATAGCTCCAGACACTTTTGGGTTTGAGGACTGGTGGAGTGACCTCATGTGTAAATTAGTTTTCTATGTAAACAGATTGATGAGGGCCCTCTCTCTCTGTACTACTTGCCTGCTGAGTGTTCTCCAGGCCATCATGCTCAGCCCTAGAAACTCTTGTCTGGCAATATTTAAGCATAAATCCACACACTATTACCCATGTTGCCTTGGCTTTTTATGGGTCTTCAATATGCTCCTGAATACTCGTTTCTTAGTCTCCATTGGGGCCACCCCAAATGTGACCTCACACAGTCTCATGTTTGTCACTGAATCCTGCTCTCAGTGGCCTATCAGTTACTTGTTCAGGTACATGTTTTTCTCATTAGCGAACGTTCAGGATGTCTCCTTTATAGGGCTGATGGCTCTCTCAAGTGGATACATGGTGAGTCTCTTGTCCAGGTATAAGAGGCTGCTCCAGCATCTTCACAGCACCAATCTTTCTCCAAAATCATCCCCAGAGGATAGGGCCACCCGGACCATTCTGGTACTCATGGGTTTCTTTACATTTATGTACTTTTTGGACTGTGTGATTTTCTCAGTCTCTGGGATATTGTGGAAGAATGATCCCATTCATCTTTGTGTCCACATGATTGTGGACAATGGTTATGCCGCAATCTGTCCTTTTGTCCTAATGAGTAATGAGAGACGAATGATGAGATGCTTAATATCTAGGTGGAAAAGATAG

>Chinchilla_lanigera_intact_V1R90_11

ATGAAGATGAATAAAAAGAGAAAATTTCCCAGTTATACTGCCTTAAGAAACACAGTTTTCTTTGAAATCACCATTGGGATCTCAGCCAACACTGTCCTACTTATGTTCCACATCCTCACATTTCTCCTCAAGCACAGGCCCAAGCCCCTGGACCTGACTATCGCTCAGTTGGCCCTAGTCCACCTGGTGATGCTGGTAACTGTGGGCTTTGTAGCTGCAGACACTTTTGGATTTGAGAACTGGGGGAATGACCTCATGTGTAAAACAGTTATCTATGCAAACAGGTTGACGAGGGCCCTCTCCATCTGTACCACCTGCCTGCTGAGCATCCTCCAGGCCATCACCCTAAGCCCAAGAAATTCTTGTCTGGCAAAATTCAAACATAAATCCTCACATTGCTACCCATGTGGGCTTGTCTTTCTATGGATCTTCAATATGCTCCTGAATGGTCGCTTCTTAGTCTCTATTGGTGCCACCCCCAATGTGACCTCTCACAGTTTCGTGTTTATCACTGAATCCTGCTGTCAGTGGCCCATTAGTTACTTGTTCAGGTACATATTTCTCTCACTGGTGAATGTTCAGGATATCTCCTTTATAGGGCTGATGGCTCTCTCAAGTGGGTACATGGTGAGTCTCTTATGCAGGCACAAGAGGCAGTTTCAGCACCTTCACAGCACCAGCCTTTCTCCAAAACTATCTCCAGAAGAAAGGGCCACCTGGACCATCCTTCTGTTCATGGGTTTCTTTATGCTCATGTACTTTTTGGACTGTATAATTTTCTTATCCTCTGGAATACTGTTGAAAAATGACCCAGTTCGTCATTCTGTACAGTTGCTTGTGGGCAGTGGGTATGCCACAATCTCTCCATTTGTGCTAATGAGCACTGAAAAACGGATGATCAGGTGCTGA

>Chinchilla_lanigera_intact_V1R90_12

ATGAAGATGAAAAAAAACAGCAAAATTTTCAGGTTTATTGACATACAATACATGTTTTTCTTTGAAGCCAGCATTGGGATCATAGCCAACACAATACTGCTTCTCTTTTACGTCCTGACATTTCTCCTGCAGCACAGACTCAAGCCCACTGACCTGACCATTGGCCACTTAGCTTTTATTCACATTGTAATGCTGGTAACTGTGGTTTTCATAACCATAGACATTTTTGGATATCAGGGTTTAGGGAATGACATCAAATGTAAGTCTGTTATATATTTGTACAGGGTGATGAGGGGCCTCTCCATCTGTACCACCTGCCTGCTGAGTGTCCTCCAGGCCATCACCCTCAGCCCCAAAAGCTCCTGTTTGGCAAAGTTCAAACAGAAATCCCTATATCAGAACTTATGTTGCTTTCTGTTTTTATGGGTCTTCAACATGCTTATGAGTGGTCGTTTCTTAATCTCCACTATTGCCACCCCTAACATGACTTCCCATAACCTTGTGTTTGTCACTCAATCCTGCTCCCTTTGGCCCATAAATTCCTTCATTAAATATATAACTTTCTCCTTGATGATTTTCCAGGAAATTCCTGTTATAGGACTAATGGGGCTCTCAAGTGTGTACATGGTGATTCTTTTGTACAGGCACAGAAGACAGTTCCAGCATCTTCACAGAACCAGCTTAGCTCCAAAAACATCCCCAGAGCAAAGGGCCACTCACACTATTTTGTTGCTCATGAGTGTCTTTATTGTCATTTATGTTTTGGACTCTGTTATCTCCTCCACATCTGGAGTTTTCTGGAAGCATAACCAGGTTCATCACTGCGTACAGATGCTGATTGGCAATGGCTATGCCACAGTCAGTCCGTTGGTGCTCATCAGTACTGAGAAAAGAATGATCAAGTGTTCAACATCTATGTTTTGGAATGATACTTAG

>Chinchilla_lanigera_intact_V1R90_13

ATGAAGATGATTAAAAACAGCAAAATTTTCAGGTTTATTGACATACAATATGTGTTTTTCTTTGAAGTCAGCATTGGGATCATAGCCAACACAATACTGCTTCTCTTTTACATCCTCACATTTCTTCTGCAGCACAGACTCAAGCCCACTGACCTGACCATTGGCCACTTAGCTTTTGTTCACATTGTGATGCTGGTAACTTTGGGCTTCATAGCCATAGACATTTTTGGATATCAGGGTTTAGGGGATGACATCGCATGTAAATCTGTTATATATTTGTACAGGGTGATGAGGGGCCTCTCCATCTGTACCACCTGCCTGCTGAGTGTCCTCCAGGCCATCACCCTCAGCCCCAGAAGCTGCTGTTTGGCAAAGTTCAAACAGAAATCCCTGCATCAGAACCTGTGCTGCTTTCTGTGCTTATGGGTCTTCAACATGCTTATAAGAGGTCGATACTTAATCTCCACTACTGCCACCCCTAACATGACTTCCCATAACCTTATGTTTGTCACTCATTCTTGCTCCCTTTGGCCCATTAGTTACGTCCTTAAGTATATAACTTCCTCCTTGATGATTTTCCAGGATATTTCTGTTGTAGGACTAATGGGGCTCTCAAGTGTGTACATGGTGATTCTTTTGTACAGGCACAGAAGACAGTCCCAGCATCTTCACAGAACCAGCTTAGCTCCAAAAGCATCCCCAGAGCAAAGGGCCACTCACACTATTTTGTTGCTCATGAGTGTCTTTGTTGTCATTTATGCTTTGGACTGTGTTATTTCCTCCACATCCGGAGTGTTCTGGAAACCTAACCAGATTCATCACTGTGTCCAGATGCTGATTGGCAATGGCTATGCCACAATCAGTCCTTTGGTACTCATCAGTACTGAAAAACGAATGATCAAATGTTTAACATCCATGTATTGGAAAAATGGTGAATGCTTGTTTATTGGTGATGACAGTTTCTCTGACAGTGGGAAATTATTTTAA

>Chinchilla_lanigera_intact_V1R90_14

ATGATGGTGAAAAGGAAGAGAACATTTTCCAGGTTTTTTGATGTGAGAAGCGCAGTTTTCTTTGAAATCACCATTGGGATCTCAGCCAACACTGTCCTGCTTCTGTTCCACAGCCTCACATTTCTCCTCAGGCACAGGCCCAAGCCCCTGGACCTCACTATCGCACACCTGGCCCTGATCCATCTGGTGATGCTGGTAACCATGGGCTTCATAGCTGCAGACACTTTTGAGTTTCAGGGTTGGTTGGATGGCCTCATGTGTAAATTGGTTATCTACATGCACAGGTTGATGAGGTCCCTCTCCATCTGTACCACCTGCCTACTGAGCGTCCTCCAGGCCATCACCCTAAGTCCCAGAAACTCTTGTTTGACAAAATTCAAACATAAATCCTCACATCACTACCCATGTTGCCTTGTGTTTTTATGGGTCTTCAATATGATCCTGAATACTCGTTTCTTAGTCTCCATTGATGCCACCCCCAATGTGACCTCACACAGTCTCGTGTTTGTCACTGAATCCTGCTCTCAGTGGCCAATTAGTTACTTGTTCAAGTACGTATTTTTGTCATTGGTGAACATTCAGGATATCTCCTTTATAGGGCTGATGGCTCTCTCAAGTGGATACATGGTGAGCCTGTTGTACAGGCATAAGAGGCAGTTCCAGCACCTTCACAGCACCTACCTTTCTCCAAAAGCATCCCCAGAAGAAAAAGCCACTTGGAGCATCCTGCAGCTCATGGGTTTCTTTATGCTCATGTACATTTCAGACTGTGCTACTTTCTCATCCCGTGCAACACTGTGGCATAATGAACTAATTAGCCTTTGTGTCCAGATGCTTGTGGGCAATGCTTATGCCACAATCTGTCCTTTTGTGCTGATGAGCACTGAAAAACGGATGACTGAGTGCTTAACATCCATGTGGAAAAGGCTATGA

>Chinchilla_lanigera_intact_V1R90_15

ATGAAGATGAATAAGAACAGCAAACTTTTCAGGTTCATTGACATACAATACGTGTTTTTCTTTGAAGTCAGCATTGGGATCATAGCCAACACAATACTGCTTCTCATTTATGTCCTGACATTTCTCCTGCAGCACAGACTCAAGCCTACTGATCTGATCATTGGACACTTAGCCTTTATTCACATTGTGATGCTAGTAACTTTGGGTTTAATAGCAATAGACATTTTTGGATTTCAGAGTTTAGGGGATGATATTACATGTAAATCTGTTCTATATTTGTACAGGGTGATGAGAGGCCTCTCCATCTGTACCACCTGCCTGCTGAGTGTCTTCCAGGCCATCACCCTCAGCCCCAGAAGCTCCTGTTTGGCAAAGTTCAAACAGAAATCCCTGCCTCAGAACCTGTGCTGCTTTCTGTGCTTATGGGTCTTCAATATGGTCATCAGTGCCCGTTTCTTAATCTCCACAATTGCCACCCTTAACGTGACTTCCCATAACCTTATGTTTGTCAGTCAGTCCTGCTCCCTTTGGCCCGTTAGTTACTTACTTAAGTATATAACTTTCTCCTTGATGATATTCCAGGAAATTTCTGTTATAGGGATAATGGGGCTCTCAAGTGTGTACATGGTGATTCTTTTGTACTGGCACAGAAGACAGTCCCAGCATCTTCACAGAACCAGCTTATCTCCAAAAGCATCCCCCGAACAAAGGGCCACTCACACTATTTTGTTACTCATGAGTGTCTTTATTGTCATTTATGCTTTGGACTGTGTTATCTCCTCCACATCTGGAGTGTTCTGGAAACCTAACCAAATTAATCTCTGTATTCAGATGCTAGTTGGAAATAGCTATGCCATGATCAGTCCTTTTGTGCTAATCAACACCAGAAAAGGAGTGATAAAATGTGTAACAACCACATTTTGGAAAGATAGTTAA

>Chinchilla_lanigera_intact_V1R90_16

ATGATGGTGAAGAAAAAAAGAAAGATTTTCATGACTTTTTATGTGAGACGCATATTTTTCTTTGAAATCACTGTCGGGATCACAGCCAACACTATCCTGCTTCTGTTCCACAGCCTCACATTTCTCCTCAAGCACAGGCCCAAGCCCCTGGACCTGACTGTTGCACACCTGGCCCTGATCCACCTGGTGATGCTGGTAACCATGGGCTTCGTAGCTGCAGATACTTTTGAGTTTCAGGGTTGGGTGGATGGCCTCACATGTAAATTGGTTATCTACGTGCACAGGTTGACGAGGTCCCTCTCCATCTGTACCACCTGCCTGCTGAGCATCCTCCAGGCCATCACCCTAAGCCCCAGAAACTCTTGTTTGGCAGAATTGAAACATAAATCCTCACATCACTACCCATGTTGCCTTGTGTTTCTATGGATCTTCAATATGATCCTGAATACTCGTTTCTTAGTCTCCATCGGGGCCACCCCCAATGTGACCTCACACAGTCTCCTGTTTGTCACTGAATCCTGCTCTCAGTGGTCTATTAGTCACTTATTCAGGTACATATTTTTGTCACTGGTGAATATTCAGGATATCTCCTTCATAGGGCTGATGGCTCTCTCAAGTGGATACATGGTGAGCCTGTTGTGCAGGCATAAGAGGCAGTTCCAGCACCTTCACAGCACCAATCTTTCTCCAAAAGCATCTCCCGAAGAAAGGGCCACTCAGAGCATCCTGCTTCTCATGGGTTTCTTTATGCTCATGTACTTTTTGGACTGTGTTACTTTCTCATCCCGTGCAATATTGTGGAACAATGACCCAATTAATCTTTGTGTCCAGATGCTTGTGGGCAGTGGTTATGCCACAGTCTGTCCTTTTGTGCTAATGAGCACTGAAAAACGGATGATTGAGTGCTTAACATCCAGGTGGAAAAAGTATAAATGCTCCAGAATTTGCTGA

>Chinchilla_lanigera_intact_V1R90_17

ATGAAGATGAATAAAAACAGCAGATTTTCTAGTTTTATTGATATACAACACATGTTTTTCTTTGAAGTCAGCCTTGGGGTTATGGCCAACACTGCCCTGCTTCTCTTTCATGTCCTCACATTCCTTCTGCAGCGCAAGCCCAAGACCACTGATCTGACCATCGGTCACTTGGCCCTTATCCACATAGTGATGCTCCTAACTGTGGGCTTCATAGCTATGGACATATTTGGGTTTCAGGACTTAGGGGATGGCATCACATGTAAATGTGTTTTCTATTTGTACAGGGTGATGAGGGGCCTCTCCATCTGTACCACCTGCATGCTGAGTGTCCTCCAGGCCATCACCCTCAGCCCCAGAAGCTCCTGTTTGGCAAAGTTCAAACAGAAATCTCTGCATCGGAACCTGTGTTGCTTTTGCTTTTTATGGGTCTTCAATATGCTCATCAGTGGTCGTTTCTTAATCTCCACTGTTGCCACTCTCAATGTGACCTCACACAGTCTCATGTTTGTCACTAAATCCTGCTCCCTTTTGCCTATCAGTTCCTTCCTCAAGTACATGTCCTTGTCACTGATGATTTCCCAGCATATATCGTTTATAGGGATCATGGCGCTCTCAAGTGGGTACATGGTGATTCTCTTGTGCAAGCATGAAAGACAATCCCAGCGTCTGCACTGCATCAGCCTAGCTCCAAAAACATCCCCAGAACAAAAGGCCACCTGGACTATTTTGTTGCTCATGAGTTTCTTTATTGTCATGTACATTTTGGACAGTGTTATTGAGTACACCTCGGCCATGTTGTGGAGCTATGACCCAGTTCGTCATTGTGTCCAGATGCTGATTGGCAATGGCTATGCCACAGTCAGTCCCCTGGTGCTCATCAGTACCGAAAGACGATTCATCAAGTGCTTTACACGCATGTGGGGGAAACATAGTAAATTTTTGTGTTTCATGATGGGTAATCTCTCTGACAACTGA

>Chinchilla_lanigera_intact_V1R90_18

ATGAATAAAAAGAGAAAAATTTCCAGTTATATTGACATAAGAAACACAGTTTTCTTTGAAATCACTGTCGGGATCACAGCCAACACTGTCCTGCTTCTGTTCCACATCGTCACATTTCTCCTCAAACACAGGCTCAAGCCTCTTGACGTGACTATTGCACAGTTGGCTCTAGTCCACTGGATGTTACTGGTAACCATGGGCTTCATAGCTACAGACACTTTTGGGTTTGAGAACTGGGGGAGGGACCTCATGTGTAAATCAGTTATCTATGTAAACAGGTTGACAAGGGCCCTCTCCATCTCTACCACCTGCCTGCTGAGTGTTTTTCAGGCTATCACCCTAAGCCCCAGAAACTCTTGTTTGGAAAAATTCAAACATAAATCCTCACATCACTACCCATGTTGCCTTATTTTTTTATGGGTCTTCAATATGCTCCTGAATATTCGCTTCTTAATCTCCATTGGTGCCACCCCCAATGTAACTTCACACAGTCTCGTGTTTGTCACTGAATCCTGCTCTCAGAGGCCTATTAGTTACTTGTTCAGGTACATATTTCTCTCACTGGTGAATGTTCAGGATATCTCCTTTATAGGGCTGATGGCTCTCTCAAGTGGATACATGGTGAGTCTCTTGTGCGGGCATAAGAGGCAGCTCCAGCATCTTCACAGTACTAATCTTTCACCAAAAATATCCCCAGGACAGAGGGCCACTCAGACAATTCTTTTGCTCACGAGTTTCTTTATGCTCATGTACTTTTTGGACTGTGTTACTTTCTCCTCCCTTGTAATATTGTGGAACAATGACCCAATTAATCTTTGTGTCCAGATGCTTGTGGGCAATGGCTATGCCACAATCTGTCCTTTTGTGCTGATGAGCACTGAGAAACGGATGATTGAGTGGTTAATATCCAGGTGGAAAAGACAGTAA

>Chinchilla_lanigera_intact_V1R90_19

ATGATGGTGAAGAAAAAGAGAAAATTTTCCCGGTTTTTTGATGTGAGAAGCACATTTTTCTTTGAAATCACCATCGGGATCACAGCCAACACTGTCCTGCTTTTGTTCCACATCCTCACATTTCTCCTCAAGCACAGGCCCAAGCCCCTGGATCTGACTATCGCACAGCTGGCCCTAGTCCACCTGGTGATGCTGGTAACCATGGGCTTCATAGCTGCAGACATTTTTGAGTTTCAGGGTTGGGAAGATGGTCTCACATGTAAATTGGTTATCTACGTGCAAAAGTTGATGAGGTCCCTCTCCATCTGTACCACCTGCCTGCTGAGCATCCTCCAGGCCATCACCCTAAGCCCCAGAAACTCTTGTTTGGCAAAGTTCAAACATAAATCCTCACGTCACTACCCACGTTGCCTTGTGTTTTTATGGGTCTTCAATATGATCCTGAATACTCGTTTCTTAGTCTCCATTGATGCCATCCCCAATGTGACCTCTTACAGTCTCGTGTTGGTCACTGAATCCTGCTCTCAGTGGCCTATTAGTTACTTGTTCAGGTATATATTTTTGTCACTGGTGAATATTCAGGATATCTCCTTTATAGGGCTGATGGCTCTCTCAAGTGGATACATGGTGCGCCTGTTATACAGGCATAAGAGGCAGTTACAGCACCTTCACAGCACCTATCTTTCTCCAAAAGTATCTCCAGAAGAAAGAGCCACTCGAAGCGTCCTGTTGCTCATGGGTTTCTTTATGCTCATGTACTTTTTGGACTGTGTTACTTTCTCATCCCGTGCAATATTGTGGAACAATGACCCAATTAATCTTTGTGTCCAGATGCTTGTGGGCAGTGGTTATGCCACAATCTGTCCTTTTGTGCTAATGAGCACTGAAAAACGTATGACTGAGTGCTTAAAATACAGATAG

>Chinchilla_lanigera_intact_V1R90_2

ATGAAGAAAGACAGCATACTTTCAAGTATCACTGACTTACAATACATGTTTTTCTTTGAAGTCAGCATTGGGGTCATGGCCAACGTGGCCCTGCTTCTCTTTCATGTTCTAAAATTCCTTCTAAAGCACAGGCCCAGGCCTACCGATCTGACCATTGGTCATCTGGCTCTTATACACACAGTGATGCTTCTAACTGTGGGCTTCATAGCTATGGACATGTTTGGGTTTCAGGACTTGGGGGATGACATCACATGTAAATCTGTTATATATTTGTACAGGGTGATGAGGGGCCTCTCCATCTGTACCACCTGCCTGCTGAGTGTCCTTCAGGCCATCACCCTCAGCCCCAGAAGCTCCTGTTTGGCAAAATTCAAACAGAAATCCCTGCATCAGAATCTGTGTTGCTTTTTCTTTTTATGGGTCCTCAATATGCTCACTACCGGTCGTTTCTTAATCTCCACTGTTGCCACTCCCAATGTGACCTCACACAGTCTCATGTTTGTCACTCGATCCTGCTCTCTTTTGCCCAGGAATTCCTCTCTCAAGTACATATCTATCTCACTGTGGGGTTTCCAGCAAATGTGCTTTATAGGGCTCATGGCACTTTCAAGTGGGTACAAGGTGCTTCTCTTGTGCAGACATGAAAGGCAGTCCCAGCATCTTCACAGCACCAGCCTGTCCCCAAAAGCATCTGCAGAACAAAGGGCCACCCGGACTATTTTGTTGCTCATGAGTTTCTTTATTGTCATGTACATTTTGGACAGTGTTATTGCCTCTACTTCTGAAATGTTGTGGAATTTTGACCAAATTCGTCATTGTGTCCAACTGCTAGTTGGCAATTGCTATGCCGCAGTCAGTCCTTTGGTGCTCATCAGTACTGAAAGAAGAGTGATCAGGTTTTTAGATCCATGTGGGGGAAAGATAGTAAATGTTTGTAAATTCAGCGATGGAAAATTTTTCTGGAAAGCAACAATGGATTTGAATTAA

>Chinchilla_lanigera_intact_V1R90_20

ATGGTGAAGAAAAAGGGAAAAATTTCCAGGTTTTTTGATGTGAGAAGCACATTTTTCTTGGAAATCACCATCGGGATCACAGCCAACACTGTCCTGCTTCTGTTCCACATCCTCACATTTCTCCTCAAGCACAGGCCCAAGCCCCTGGACCTCACTATCACACAGCTGGCCCTGATCCACCTCGTGTTGGTGGGAACCATGGGCTTTATAGCTACAGACATTTTTGAGTTTCAGGGTTCGTTGGATGGCCTCATGTGTAAATTGGTTATCTACGTGCAAAAGTTGATGAGGTCTCTCTCCATCTGTACCACCTGCCTACTGAGTGTCCTCCAGGCCATTACCTTAAGCCCCAGAAACTCGTGTTTAGCAAAGTTCAAACATAAATCCTCACATCACTACCCATGTTACCTTGTGTTTCTATGGGTATTCAATATGATCCTTAATACTCGTTTCTTAGTCTCCATTGATGCCATCCCCAATGTGACCTCACACAGTCTCGTGTTTGTCACTGAATCCTGCTCTCAAAGGCCTATTACTTACTTGTTCAGGTACATATTTCTCTCACTGGTGAATATTCAGGATATCTCCTTTATAGGGCTGATGGCTCTCTCAAGTGGATACATGGTGAGTCTCTTATGCAGGCATAAAAGGCAGTTCCAGCACCTTCACAGCACCAATCTTTCTCCAAAAGCATCTCCAGAAGAAAGGGCCACTCGGAGCATCCTGCTGCTCATGGGTTTCTTTATGCTCATGTACTTTTTGGACTGTGCTACTTTCTCATCCCGTGCAATATTGCAGAACAATGACCCAATTAATCTTTGTGTCCACATGCTTGTGGGCAACGGCTATGCCACAATCTGTCCATTTGTGCTGATGAGCTCTGAAAAACGGGTAATTGCGTGCTTAAGATACAGATGGAAAAGACTACGAATTTTCTAG

>Chinchilla_lanigera_intact_V1R90_21

ATGGAGATAAAGAAAAGCACCAAATTTTCCAGGTTTTTTGTCATTGGAAACATGTTTTTCTTTGAAGTCAGCCTTGGGATCGTGACCAACACTGTTCTGCTTCTCTTTCATGTCCTCACATTCTTTCTGCAGTACAGGCACAAGCCCATTGATCAGACCATTGGCCACTTGGCCCTGATCCACTTAGTGATGCTCCTAACTGTGGGATTCATGTTTATAGACATTTCTGGCTTTGAGGATTTCTGGAATGATGCCACATGTAAATGTGTTCTCTATTTGTACAGGGTGATGAGGGGCCTCTCCATCTCTACCACCTGCCTGCTGAGTGTGCTCCAGGCCATCACTCTTAACCCAGGAAACTCCTGTCTGGCAAAATTCAGACAGAAGCCCCTGCATCAGAACCTGTGTTGCTTTCTCTTTTTATGGGTTTTCAATATGATCGCCAGTGGTCGTTTCTTAATCTCCACTATTGCCACCCCTAATGTGTCCTCACAGAGTCTTATATTTGTCACGGAATCCTGCTCTCTTCGTCCCATCAGTTACTTAGTCAAGTACATATATTTCTCACTGGTGACCTTCAGGGATGTGTCCTTTATAGGGCTTATGATGTTCTCAAATGGGTACATGCTGATCCTCTTGTGCAGGCATAAAAAGCGGTCCCAGCATCTTCGTAGCACCAGCCTATCCCCAAAAGCATCTCCAGAACAAAGGGCCACCCAGACTATCCTGGTGCTCATGAGTTTCTTTATGATCATATACTTTTCGGACGGTGTTATTGCCTCCACTTCTGGAATCTTGTGGAAAAATGACCCCATTCGTTATTGTGTCCAAATGCTCTTGAGCAATGGCTATGCTACGTTCAGCCCCTTGGTGCTAATCAGTACTAAAAAACAGATGATCAAGGGCTTAGTATCCATGTGGGGAAGGTCAGTAAATGTCAGATTGTTCACTGATGAATATTTGCCCTGA

>Chinchilla_lanigera_intact_V1R90_22

ATGGTAATGAAAAAGGGAACATTTTTAAGGTTTTTTAATGTGAGAAACACAATTTTCTTTGAAATCACCCTTGGGATCTCAGCCAACACTGTCTTGTTTCTGTTACACATCCTCACACTTCTCCTCAAGCACAGGCCCAAGCCACTGGACCTCACTATTGCACCCCTGGCCCTGATCCATTTGGAGATGCTGGTAACTATGGGCTTCATAGCTGCAGACATTTTTGAGTTTCAGGGTTGGGGGGATGGCCTTGTGTGTAAATTGGTTATCTACGTGCACAGGTTGATGAGGTCCCTCTCCATCTGTACCACCTGCCTGCTGAGTGTTCTCCAGGCCATCACCCTAAGCCCCAGAAACTCTTGTTTGGAAAAACTTAAATATAAATCCTCACAATACCCATGTTGCTTTGTGTTTCTGTGGATCTTCAATTTGATCCTGAATACTCGTTTCTTAGTCTCCATTGGGGCCACCCCCAATGTAACCTCACACAGTCTCATGTTTGTCACTGAATCCTGCTCTCAGTGGCCTATTAGTTACTTGTTCGGGTACGTATTTTTGTCATTGGTGAGTATTCAGGATATCTCCTCTATAGTGGTGATGGTTCTCTCAAGTGGATACATGGTGAGCCTGTTGTGCAGGCATAAGAGGCAGTTCCAGCAACTTCATAGCACTAATCTTTGTCCAAAAGCATCCCCAGAAGAGGCCACTCGGAGCATCCTGCTGCTAATGGGTTTCTTTATGCTCATGTACTTTTTGGATTGTGTTACTTTCTTAACCCTTGCAATACTGTGGAAAAATGACCCAATTAATATTTGTGTGCAGATGCTTGTGTGTAATTGTTATGCCACAGTCTGTCCTTTTGTGCTAATGAGCACTGAAAAACGGATGATTGTATGTTTAAAATCCACGTGGAAAAGACTGTGA

>Chinchilla_lanigera_intact_V1R90_3

ATGCTGGTGAAGAGGAAGAGGAACTCTTTTATGTTTTTTGATGTGAGAAGTGCAGTTTTCTTTGAAATCACCCTTGGGATCTTAGCCAACACTGTCCTGCTTCTGTTCCACATCCTCACATTTCTCCTCAAGCACAGGCCCAAGCCCCTGGACCTCACTATCGCGCACCTGGCCCTGATCCACCTGGTGATGCTGGTAACCATGGGCTTCATAGCTGCAGACACATTTGAGTTTCAGGGTTGGGAGGATGGTCTCATGTGTAAATTGGTTATCTATGTGCACAGGTTGATGAGGTCCCTCTCCATCTGTACCACCTGCCTGCTGAGCGTCCTCCAGGCCATCACCCTAAGCCCCAGAAACTCTTGTTTGGAAAAATTCAAGCATAAATCCTCACACCAATACCCATGTTGTCTTGTGTTTTTATGGGTCTTCAATATTCTCCTGAATACTCGTTTCTTAGTCTCCATTGGTGCCACCCCCAATGTGACCTCACACAGTCTCGTTTTTGTCACTGAATCCTGCTCTCAGTGGCGAATTAGTTACTTGTTCAGGAACATATTTTTGTCACTGGTGAACATTCAGGATATCTCCTTTATAGGGCTGATGGCTCTTTCAAGTGGATACATGGTGAGCCTGTTGTACAGGCATAAGAGGCAGTTCCAGCATCTTCACAGCACCAATCTTTCTCCAAATGCATCTCCCGAAGAAAAGGCCACTCAGAGCATCCTGCTGCTCATGGGTTTCTTTATGCTCATGTATTTTTTGGACTGTGTTACTTTCTCATCCCGTGCAATATTATGGAAAAATGACCCAATTAATCTTTGTGTCCACATGCTTGTGGGCAATGGCTATGCCACAATCTGTCCTTTTGTGCTAATGAGCACTGAAAAGCGAATGACTGAGTGCTTAACATCCAGGTGGAAAAGACTATAA

>Chinchilla_lanigera_intact_V1R90_4

ATGAAGGTGAATAAAAACAGCAAATATTCCTGTTTCATTGGCATGCAGCACATGGTTTTCTTTGAAGTCAGCGTTGGGATCATTGCCAACATGGTTCTCTTTCTCTTTCATGTTCTCACATTCTTTCTGGGGCACAGGCCTAAGGCTACTGATCTGACAGTTGGTCACCTGGCCTTTATCCACATAGTGATGCTCCTGACTGTGGGTTTCATAGCTATAGACAATTTTGGGTATCAGGATTTGAATGATGACATCACATGTACATCTATTATCTATTTGAATAGATTGATGCGGGACCTCTCCATCTGTACCACCTGCCTGCTCAGTGTCCTCCAGGCCATCACCCTCAGCCCCAGAAGCTCCTGTTTGGCAAAATTCAAGCAGAAATCCTTTCATCAGAGCCTGTGTCGCTTTCTCTTTTTGTGGGTCCTCAATATGGTCATCAGTAGTCGTATGTTAATCTCCACTGTTGCCACTCCCAATGTGACCTCACACAGTCTCATGTTTCTTACTAAATCTTGCTCTCTTTTGCCCATTAATTCCTTCCTCAAGTACATGTTTTTTTCACTGCTGAGTTTCCAGCACATGTCCTGTATAGGGCTCATGATGCTCTCAAGTCTGTACATGGTGATTCTCTTGTACAAGCATAAAACGCAGTCTCAGCATCTTCACAACACCAGCCTGTCTCCAAAATCATCTGCAGAAGAAAGGGCCGCACGGACAGTTCTGTTGCTAATGAGTTTCTTTATTGTCATGTACACTTTGGACTCTGCTATCGGGTTGACTTCTTCAGTCATGTGGAACAGTGACCGCATTCATTATTGTGTGCTGATGCTGATTGGCAATGGCTATGCCACAGTCAGTCCCTTGGTGCTTATCAGTACAGAACGACGAATGATTAAGTGCTCCATATTCATGTGGTGGAAAGTGAATAAATGGTTTTATATTTCATGA

>Chinchilla_lanigera_intact_V1R90_5

ATGAGTAAAAAGAGAAAATTTTCCAGTTTTTCTGACATAAAAAACACAGTTTTCTTTGAAATCACCATCGGGATCACAGCCAACACTGTCCTGCTTCTGTTCCACATCCTCACATTTCTCCTCAGGCACAGGCCCAAGCCCCTGGACCTCACTATTGGTCAGTTGGCCCTGATCCACCTGGTGATGCTGGTATCAATGGCCTTCATAGCCACAGACACTTTTGGGTTTCAGGACTGGAGCGATGACCTCACATGTAAATCAGTTATTTACATAAACAGGTTGATGAGGGCCCTCTCCATCTGTACCACCTGCTTGCTGAGTGTCCTCCAGGCCATCACCCTCAGCCCCAGAAAATCTTGCTTGGCAAAATTCAAACATAAATCCTCACATTACTACCCTTGTTGCCTTGTGTTTTTATGGGCCTTCAATATTCTCCTGAATACTCGTTTCTTAATCTCCATTGGTGCCACCCCCAATGTGACCTCACACAGTCTCCTGTTTGTCACTGAATCCTGCTCTCAGTGGCCTATTAGTTACTTGTTCAGGTATGTATTTCTCTCACTGGTGAATGTTCAGGATATCTCCTTTATAGGGCTGATGGCTCTCTCAAGTGGATACATGGTGAGTCTCTTGTGCAAGCATAAGAGGCAGTTTCAGCATCTTCACAGCACCAGCCTTTCTCCAAAATCAGCCCCAGAAGAAAGGGCCACTCGGACCATTTTGCTGCTTATGGGTTTCTTTATGCTCATGTACTTTTTGGACTGTGTCACCTTTTCCTCCTCTGGAATATTGTGGAGAAATGAACCAATTAGCTATTGTGTCCGTATGCTTGTGGGCAATGGCTATGCCACAATCTGTCCTTTTGTGCTAATGAGCATTGAAAAGCGAATCATGAAGTGGTTAAAATTCAGGTGTTAA

>Chinchilla_lanigera_intact_V1R90_6

ATGAAGATGAATAAAAAGAGAAAATTTTCCAGTTATACTGACATAAGAAACATAGTTTTCTTTGAAGTCTCCATTGGAATCACAGCCAACACTGTCCTGCTTCTGTTCCACAGCCTCACATTTCTCCTCAGGCACAGGCCCAAGCCCCTGGACCTGACTATCGCTCAGTTGGCCCTGATCCACCTGGTGATGCTGGTAACTGTGGGCTTCATAGCTACAGACACTTTTGGGTTTGAGGGTTGGGGCAGCGATCTCATGTGTAAATCAGTTATCTATGTGAACAGGTTGATGAGGGCCCTCTCCATGTGTACCACCTGCCTCCTGAGCGTCCTCCAGGCCATCATGCTCAGTCCCAGAAATTCTTGTTTGGCAAAATTCAAACATAAATCCACACATTACTACCCATGTGGCCTTGTCTTTCTATGGATCTTCAATATGCTCCTGAGTGGTCGCTACTTATTCTCTGTGGGTGCCACCTCCAATGTGACCTCTCACAGTTCTGTGTATGTCACTGAATCCTGCTCTCAGTGGCCTATTAGTTATTCATTCAGGTACATATTTTTGTCACTGGTGAATATTCAGGATATCTCCTTCATAGGGCTGATGGCTCTCTCAAGTGGATACATGGTAAGTCTCTTATGCAGGCATAAGAGGCAGTTCCAGTACCTTCACAGCACCATCTTTTCTCCAAAATCATCCCCAGAAGAAAGGGCCACTAGGACTATTCTGCTGCTCATGAGTTTCTTTATGCTCATGTACTTTTTAGACTGTGTTATTTTCTTATCCTCTGGAATAGTGTGGGAAAATAGTCCAATTCATCATTGTGTCCAGATGCTTGTGGGCAGTGGGTATGCCACAATCTGTCCTTTTGTGCTAATGAGCACTGAAAAACGAATGATCAGGTGCTGA

>Chinchilla_lanigera_intact_V1R90_7

ATGAAGATGAATAAGAACAGCAAAATTTTCAAGTTTATTGACATACAATACATGTTTTTCTTTGAAGTCATCATTGGAATCATAGCTAATACAGTACTGCTTCTCTTTTATGTCTTCCTATTTCTCCTGGAGCACAGACTCAACCCTATCAATCTGACCATTGGCCACTTGGCCCTTATTCACATTGTGATGCTGGGAACTTGTTTCATAACAATAGATATTTTTGGATTTCAAGTTTTAGGAGATGACATTACATGTAAATTGGTTATATATTTGCACAGGGTGATGAGGAGCCTCTCCATCTGTACCACCTGCCTGCTGAGTGTCCTCCAGGCCATCACCCTCAGCCCCAGAAGCTGCTGTTTGGCAAAGTTCAAACAGAAATCCCTGCCCCAGAACCTGTGTTACTTTCTCTTCATATGGGTCCTCAATATGGTCATCAGTGCCCGTTTCTTAATCTCCACTATTGCCACCCCTAATGTGACTTCCCCAAACCTTATGTTTGTCACTCAGTCCTGCTCCCTTTGGCCCGTTAGTTATCTCCTTAAATATATAACTTTCTCCTTGATGATTTTGCAGGAAATTTCTGTTATAGGGCTCATGGGACTCTCAAGTGTGTACATGGTGCTTCTTTTGTACAGGCACAGAAGGCAGTCCCAGCACCTTCACAGTACCAAATTATCTCCAAAAGCATCCCCAGAGCAAAGGGCTACTCACACTATTTTACTGCTCATGAGTGTCTTTATTGTTATCTATGCTTTGGACTGTGTCATCTCCTCAACATCTGGAGTGTTCTGGAAACGTAACCAGATTCATCATTATATCCAAATGCTGGTTGGCAATGGCTATGCCACAATATGTCCTTTGGTGCTAATCAGTACTGAAAAAAGAATGATGAAGTGTTTAACATCCAGGTCTTAA

>Chinchilla_lanigera_intact_V1R90_8

ATGATGGTGAAGAAAAACAGAAAATTTTCCCAGATTTTTCATATGAGAAGCACAGTTTTCTTTGAAATCACAGTTGGAATCTCAGCCAACACTGTCCTGCTTCTGTTCCACATCCCCACATTTCTCCTCAAGCACAGGCCCAAGCCCCTTGGCCTCACTATTGCACACCTGGCCCTGATCCACCTCGTGATGCTGGGAACCATGGGCTTTATAGCCATAGACATTTTTGAGTTTCAGGGTTGGGAGGATGGCCTTGTGTGTAAACTGATTATCTATGTGTACAGGTTGATGAGGGCCCTCTCCATCTGTACCACCTGTGTGCTGAGTGTCCTCCAGGCCATCACCCTAAGCCCCAGAAACTCTTGTTTGGAAAAATTCAAACATAAATCCTCACATCACTACCCATGTTGCCTTGTGTTTCTATGGGTCTTCAATATGATCCTGAATACTCGTTTCTTAGTCTCCATTGGGGCTACCACCAATGTGACACCACATAGTCTCGTGTTTGTCACTGAATCCTGCTCTCAGTGGCCAGTTAGTTACTTGTTCAGGTACATATTTTTGTCACTGGTCAACATTCAGGATATCTCCTTTATAGGGCTGATGGCTCTCTCAAGTGGATACATGGTGAGCCTGTTGTGCAGGCATAAGAGGCAGATCCAGCAACTTCGTAGTACCAACCTTTCTCCCAAAGCATCCCCAGAAGAAAGGGCCACTCGGAGCATCCTGCTGCTCATGGGTTTCTTTATGCTCATGTACTTTTTAGACTGCGTTGCTTTCTCATCTCGTGCAATATTGCAGAACAATGACCCAATTTATCTTTGTGTCCAGATGCTTGTGGACAATGGTTATGCCACAATCTGTCCATTTGTGCTAATGAGCACTGAAAAACGGATGATTCAATGCTTAACATCCAGGTGGAAAAGACTATAA

>Chinchilla_lanigera_intact_V1R90_9

ATGGAGATAAAGAAAAGCACCAGATTTTCCAGTTTTGCTGTCATACAAAATATGTTTTTCTTTCAAGTCAGCCTTGGGATCACGGCAAATACTGTCCTGCTTCTCTTTCATGTCCTCACATTCCTTCTGCAGCACAAGCCCAAGTCCACTGATCTGATCATTGGTCACTTGGCCCTTATCCACATAGTGATGCTCCTAATTGTGGGTGTCATAGCTGTAGATATTGTTGGTTTTCCAGATTTATGGAATGACATCACATGTAAATGTGTTCTCTATCTGTACAGATTGATGAGGGGCCTCTCCATCTGTACCACCTGCCTGCTAAGTGTGCTCCAGGCCATCACCCTCAGCCCAAGAAGCTCCTATCTGACAAAGTTCAAACAGACATCCCTGCATCAGAACCTGTGTTGCTTTCTCTTTTTATGGGTTTTCAATATGATCATCAGTAGTCGTTTCTTAATCTCCACTATTGCCACCCCTAATGTGTCCTCACAGAGTCTTATGTTTGTCACTGAATCCTGCTCTCTTTGGCCCATCAGTTACTTAGTCAAGTACATATATTTCTCACTGGTGACCTTCAGGGATGTGTCCTTTATAGGGGTCATGGCACTCTCAAGTGTGTACATGGTGAGTCTCTTATGCAGGCATAAAAGGGAGTCCCAGCATCTTCACAGCACCAGCCAGTCTCCAAAAGCATCTCCAGAACAAAGGGCCACCCAGACCATTCTGTTGCTCATGAGTTTCTTTATGACCATGTATTTTTTGGATTGTGTTATTGCCTCTACTTCTGGAATGCTATGGAAGAATGACTCCATTCATTATCGCATCCAAATGTTCTTGGGCAATGGCTATGCTACCTTCAGCCCCTTGGTGCTAATCAGTACTGAAAAACGGATGATCAAGGGCTTAGTATCTATGTGGGGAAGGTCAGTAAATGTTTAA

>Cricetulus_griseus_intact_V1R1_1

ATGGATGCATGGGTTTCCATTAGCTTAAACTGGGGAATGGCGTTCCTCGTTGAGACCACTGCTGGAATCCTAGCCAATTGCTTCCTTTTTCATCTGTATAACTTTCCACTGTTCACTGCACAAGCGGTGAGACCCACAAACTTGATTCTCAATCAGCTGGTCCTATCGAACCATCTGGTTCTTTTCTCCAAAGGGATCCCCCAGACAGTGGCCACTTTTGGGTTGACAAGTTTCCTGGGGGAGTCTGGATGCAAACTTATCCTCTACTTAAACAGAGTGGCCAGAGGGGTCTCCCTCAGCACCACCTCCCTCCTCAGTGGCTTTCAGGCGATTAAGCTTCGCCCAAATACTTCTGCGTGGCTCAGCCTTCGAATTATATCCTCAAAATATATTGGCACCTGCTGTTTCCTTTGCTGGACGCTACAGCTCCTGCTCAACCTCCATGTTACTATGAAGGCAGGTAGTTCAAAATATAGCAAAAACCTGAGTGCTAAAGGAATCTACAGATACTGTTCCTCAACCATGTCTGACAGATTGACTTTCTTACTAAAAGCATTGATTTTATCCCTAAGTGATATTGTGTGTCTGGTCATCATGGCCTGGGCCAGTGGCTCCATGGTCCTTGTCCTGCATAAACACAAGCCTTTAGTCCAGCACATCCACAGCCACAGCCTCTCCTCCAGGCCTTCCCACGAGGACAGAGCCACACGAACCATCCTGATCCTGGTGAGCCTGTTTCTCTCCTTTTATTCTCTAGCTTCCCTCTTATCGTTTTGGATAACCCAGACTGTGAGCCCAAGCCCTTGGCTGTTCAACACCACTGTGCTGTTGTCACTGGGCTTCCCAGCACTCAGCCCCTTTGTGTTCAGTTTCAACAATATTTGTGTCCCTCAGTTCTGCTCTGCACTTTGGACAAAGAAAGCAAACCCTCCAACTTTGGTCTCTGTGGTTGGTGTTTCCTCCAGGTCATGTCAACTTTAA

>Cricetulus_griseus_intact_V1R1_2

ATGGATGCATGGGTTTCCATTAGCTTAAACTGGGGAATGGCGTTCCTCGTTGAGACCACTGCTGGAATCCTAGCCAATTGCTTCCTTTTTCATCTGTATAACTTTCCACTGTTCACTGCACAAGCGGTGAGACCCACAAACTTGATTCTCAATCAGCTGGTCCTATCGAACCATCTGGTTCTTTTCTCCAAAGGGATCCCCCAGACAGTGGCCACTTTTGGGTTGACAAGTTTCCTGGGGGAGTCTGGATGCAAACTTATCCTCTACTTAAACAGAGTGGCCAGAGGGGTCTCCCTCAGCACCACCTCCCTCCTCAGTGGCTTTCAGGCGATTAAGCTTTGCCCAAATACTTCTGCGTGGCTCAGCCTTCGAATTATATCCTCAAAATATATTGGCACCTGCTGTTTCCTTTGCTGGACGCTACAGCTCCTGCTCAACCTCCATGTTACTATGAAGGCAGGTAGTCCAAAATATAGCAAAAACCTGAGTGCTAAAGGAATCTACAGATACTGTTCCTCAGAACTGCATGAGAGATTGACTTTCTTAGTAACGGGAGTTATTTTATCCCTAAGTGATATTGTGTGTCTGGTCATCATGGCCTGGGCCAGTGGCTCCATGGTCCTTGTCCTGCATAAACACAAGCGTCGAGTCCAGTACATCCATAGCCACAGCCTCTCCCCCAAGCCTTCCCACGAGGACAGAGCCACACGAACCATCCTGATCCTGGTGAGCATGTTTCTCTCCTTTTACTCTCTAGCTTCCCTCTTATCGTTTTGGATAACCCAGACTGTGAGCCCAAGCCCTTGGCTGTTCAACACCACTGTGCTGTTGTCACTGGGCTTCCCAGCACTCAGCCCCTTTGTGTTCAGTTTCAACATTATTCGTGTCCCTCGGTTCTGCTCTGCACTTTGGACAAAGAAAGCAAACCGTCCAGCCATGGTCTCTGAGTTTTGA

>Cricetulus_griseus_intact_V1R1_3

ATGATGAGCGACTTCAGTTTGGAAGTGGGGATTATATTCTTTATCCAGACCGGAATGGGCATCATGGGAAACTCCTTGCTTCTCTGTCTTTACAGCATCACTTTGCTCAATGAACAAAAGGCAAGACCCACAGATCTGATCCTCAACCAACTGGTTTTTGCCAACAACTTAGTTCTCTTCTTCAGAGGGATCCCTGAGACAATGACAGCTTTTGGATGGAAGGATTTCTTGGGTGATGCTGAATGTCAACTTGTTTTATATCTTCACCGTGTTGCCAGGGGGGTTTCTCTTAACACCACCTGCCTTCTGAGTGGCTTCCAGGCCACCAAGCTTTGTTTTAGATTCTGTTGTTGTAAGCAGCTCTGTAAGAGATGCCCAAGATGTGTTGGTGTCTGTGGCTTCTACCTCTGGATCCTACAACTCTTGGTAAATGTGTATATTCCTATGGGAGTGACAGGCCCAAGGGGCAGTCAAAACCTAAGTATGAACACGAATTACAGATACTGTTCCTCACCTATCCCGAAAAAATACATAGTTTTATTAAATGCAGTCCTGTACTTGGCCATTGACGTTATATGTTTGGTGTTCATGGTTTTGGCTGGTGGATCTATGGTCCTTGTCCTGATCCGGCACAAGCAGCAGGTTCAACACATTCACAGCCCCAGCCTCTCCTTGAGACCTGCCCCAGAGGACAGAGCTACGTACACCATTCTGAGCCTGGTGACCATGTTTGTCTTCTTCTTTGGTCTCTCTTCCATTTCAACTCTTTGTGTTGCTCTCACCAGGAACCCAGACAAGGGGCTGATTAACGCTGCTGTATTCCTAGCTGCATGTTTCCCTGCATTCAGCCCCTTTCTGCTCCTCAGTTGTGACACCCATGTCTCTCGAATCTTTGATATGTGCAATAGAAAAACTCTCTCTCCTATTATGGGCAGATGTCTATCTTCCCTATGA

>Cricetulus_griseus_intact_V1R1_4

ATGGCTTTTCGTGATTTTATTCTTAGATTACTTTTCCTGGTACAGACTAGAGTTGGGGTATTGGGGAACTCCTTGGTTCTTGCAGTCTGTACCTCCATGTACTATACCAGCCTTGCACCACGGCCTACCCATCTCATATTCACCAATATGGCTGTGGGATATTTCTTGATTCTTCTCTTTAAGGGAATTCCCCACAGCATATATATCTGGGGAATAACACACATTCTGGGAAACCATGGGTGTAAAGTTGCCTTATATATTCACAAAGTGGGCCAGAGCCTCTCTCTATGTACCACTTGCCTCTTGACTAATTTTCAGGCAATACCCATCAGTCCCAGAGTAAATGGATGGATGGGGCTCAAAAGCCAAGCCTGGAGGATCAGTGTCTGTTGCTTTCTCTGTTGGATATCCAGCTTGCTGATAAAAATCTTTGACCTTGTACATACAGAAGGCTTTCAGCATATGAAGAATTCTACCAAGAAGCAGGATTATGAACTCTGCTCTTCTCAAGTGTCAAGTATATCTGAAGGCAGTTACAGAATACTGAGAACTCTCTCAGATTTTGTATTGATAGGATTCATGGTTGGAGCCAGTGTCTACATGGTTCTTCATCTCTATAGGCACCAGCAGAGGATGAGGCATTTCCACACATTCAGTATCTCCCACAGACTGTCCTCTGAGACTAAAGCCACTCAAAGCACTCTGCTTCTAAAGAACATCTTCATTTTGCTTTCTTTCACCAAATCTATCCTTATGCTTCACAATGCTGCTATTTTCAAGTTTTGCTACTGGTTCAGCATGTTACTACATTTCTGTCAACATGTGATCCCACTCTCAGGCCAATGGTACTGA

>Cricetulus_griseus_intact_V1R2_1

ATGATGGTGTTTCCTGATTTGCCATTTACAATTATTATATTATCCCAGAACTTTGTAGGAATCTTGGGCAATTGTTCACTTCTGTTTCATTATGTCATGTCTGTATTCACAAGGAAGAGTTTCATGCCCAAAGACAAGATTATAAAGCATGGGGCAATTGCCAACTTCTTGTATGTGATCTCAAGAAGCATGGATCAGATAATAGTTGAGCTTGGGCTAAAATGTTCCCTGGATGGCAATGCTTGTAAATTTCTTCTATACCTGTATAATGTAGCTCGGGGAGCTTCTCTTCATTCCTCAAGTCTCCTGATTTGCTTCCAAGCCATCACAGTGAACCCCAGCAACTTTAGATGGATGAAGTTTAAACACAGAGCTGCCAAGTACATTGTTCCCCCTTGTTCTCTCAGCTGGCTTGTACATCTACTTCTAAACAGCAGAATTATTATGAGTATGACTGCCCTTGGTACCAACATAAATTTCACTAAGAAATTCAATGGGAGCTCCTGCCTAACATTTTTTTCTGGCACCAATGCAGCCCTACTATATGTATTCTTGATGTGTTTCATTGACGTTCTCTATCTGTGCGTCATAGTCTGGTCTAGTATCTCCTTGCTGAGCTTCCTGTACAGGGTCAAGAAGCACATACAGTGTATCCACAGTGCCCAGAATTCCCTCAGAGTCTCACCTGGAGACAGAGCCACCGAATCCATCCTGAACCCAGTGTGCATCTATGTCACCTACTACTCAATGTCCTTCATCCTCACATTATACATAGTGCTCCGCGGCGAAATAAGGATGTGGCTGATGAGCTTAGTCGGACTTTTAGATAATTGTTTCCCTACTATTTCCCCTTCATAG

>Cricetulus_griseus_intact_V1R2_2

ATGGACACCAGGAATTTGGGAATAGGAATTATATTCTTGATAGAGAATACAGTTGGAATCCTGGGAAATGTCTGCCTTCTTTCCTACTACTTAGTAATTTATTACAAGAAACACAGCGTAAAACCCTTGGATTTAATTATCATGCATTTGATCATGGTTAATTTCTTGATCATTCTCTCTAAAGGAATGGGAAATACGATGACAGCATTTGAGCTGAAACATTTCTTCAATAATTGGAGTTACCAGCTTTTTATGTATGTGTTAAGAGTTTTCAGGAGTATGTCCATTGCTACCACTTTTCTCTTGAGTGTCTTCCAGGCCATTATCATCAGCCCTAGAAACTCCTGTTGGAATAATCTTAGATTCAAATCTCCAAAGGACATTGGTCTCTACATTTCTATCTGCTGGGTCTTGTTCATCATGGTAAATGTTCTTTTACCTTTGTATGTGTCCATAAAATTAGGGAGAAACATAACAAAAGAGACAGATTTTGAAAGAAATACTGTTGTAGATAATGACAAAATCACAGTTTCCTTATATATAGGTTTATTTGTATTTCCCGAACTTGTATTTTCTGTCCTCATCACCTGGTCCAGCAGCTCAATGATTGTCATTTTACTTAGGCACAAACATCGAGTTCAATACATTCACAGCAATTGTGCTTTCCACAGTAATATCTCAGAATCCAGAGCCACCCAGAGCATCCTTGCCAGAGTGTTCAGCTTTCTGGCTATTTATACCCTCTCTGCCATTTCACATGGTTGCAATGCTCTATTTTCTGGGCAAAGTTGGTGGCTGATGAAGATCACAATCATTATTTCTTTGTGTTTTCCCACTTTGAGCCCAATTTTGCTCATGAGTCAATCCTTCCATCTCTTCAGACTCTGCTCATTTTTGATAAAGGACAGATAA

>Cricetulus_griseus_intact_V1R2_3

ATGGACTTCTGGAATCTGGCAATCAGATTTATTTACTTATCACAAATTACAGCTGGAAGTCTGGGAAATTTCTTTCTAATTTTCTACTACCTATCCCTTTACTGGAGAAAACACAGAGTAAAGCCCACTGATTTGATTCTCATGCACCTAATGGCAGCCAATGCCTTGATCATTCTTTCCACAGGAGTGCCCCAAGCAATGGCAGATTGTGGATTTAAGCATTTCTTGAATGATTTTGGATGCAAGCTCCTGGTGTACATTCAAGGAATTGGTAGGAGTATGTCAATTGGAACCACCTGCCTCTTGAGTGTCTTCCAGGCCTTGACCATTAGTCCCAGGAAATCAGGTTGCAAGGATCAGAAAGTCAAAGTTGAGAAGAACATTCACTGCCACATCTCCCTCCTCTGGATCCTATACATATTGATAAATTTCATTTATTATGCATACACATTTGTCAAGAGAGATAACAAAAATGTGACAAGAAAACGAGATATTGGTTATTGCTCCATTGTAAGGTGGGATGAAATTGGCTCTTCAATCTATGTAGCACTGGTGGTTTGCCCTGAAGTGTTCTTTTCTGTGATCATGGCCTGGTCCAGTGGTTCCATGATTGAAATTCTCTACAGGCACAAACAGAGGGTTCAACACATCCACAGCACCCATGGTTCTAGCAAAAAGTCCCCTGAATCCAGAGTCACCCAGAATATTTTGATCCTTGTGTCTACCTTTCTAGCTTTTTATACTCTGTCCTCCATCTTAAAAGGCTACCTTACTCTTTTATATAATCATAATTGGTGGCTGGTGAACATTAATCGGATCACTTCTCTCTGTTTTCCATCATTTGTGCCCTTTGTTCTCATCTATCGGTACTCTATTGTACCCAGGTTGTTAGGTCTGGATAAGGAATAA

>Cricetulus_griseus_intact_V1R2_4

ATGTCCTTCTGGGCTCTGACCATCAAAATCATTTTCTTGTCACAAACTACAACTGGAATTCTGGGAAATTTTTCTCTTCTTTACTACTACATAGTCCATTATGGAAAAGGCACAATAAAGGCCACAGAGTTAATTCTCATGCACCTAATGGCAGCCAATGCCTTGACCATTCTCTCTGCAGGAGTGCCCCACACAATAGCAGCTTTTGGTTTGAAGCACTTTTTAAATGATTTTGGATGCAGATTACTTTTGTACATTCAAAGAGTTGGCCGCAGTGTGTCCATTGGCACCACCTGCCTCTTGAGTGTCTTTCAGGCCATGACCATCAGTCACACACAATTCTGTTGTAAGGATCTGAAAGTCAAAGCTTCAAAGTACATTGGCTTCTCTGTTGCCCTCCTCTGGATCTTTTACACGTTGATAAATTTCATTTACTTTGTGTACCTGTTTCTCAAGAGGAATGATAAAAATACGACAAGAAAACGGGATTTTGAATACTGCTCCATTGCAGGGCGCGATGAAATCAGTGACTCATTCTATGCAGCGTTGGGGGTGTTCCCTGAAATCTGCTTTTCTTTGCTTATGGCCTGGTCCAGTGGCTCCATGATTGTTATTCTATACACACACAAGAAGAGGGTTCAACATATCCTTAGCACCCATGGTTCCAGAAGAACCTCCCCTGAGTCCAGAGCCATCCAGAACATCCTGGTCCTGGTATTTATCTTTCTGGCTTTTTACACTCTCTCCTCTATCTTACAAGGCAGCATAGCTCTTTTGTATAATCACAGCTGGTGGCTGGTGAACATCACTCGCCTTACTGCTTTGTGTTTTCCCTCTTTTGCACCCTTGGTTCTTATGAATCATTACTGCAGACTACCTAGACTCAGTTTTGTCAGAGTAAGGAATATAAATCACTGA

>Cricetulus_griseus_intact_V1R3

ATGCAGGATCCTGTCACTCTGAATGGGACTGTCAGTCACTCCAGGGACTTGGCAGTAGGGATGATCTTCCTGTATCAGACCATAATTGGGTCCCTGGGGAACTGCCTTCTTTTCTGCTATTACAATTTCCATTGTTTCATGAGACGTACAGGCAGGTCTACGTATTTGATTGTCCAGCACCTGACCTTGGCCAATTTCTTGGTTATACTGACTAAAGGAGTACCACAGACGATGGCTGCTTTGGGGATGAAGGATTTCCTCAGCGATACAGGTTGCAAACTTGTCTTCTATGTCCACAGAGTGTCCAGGGGCATGTGCGTTGGTAGCACCTGCTTCTTGAGTGTCTTCCAGGCCATCACCGTCAGCCCCATGGTGTCCAGATGGAAGGAAGTGAAACGCCACATTTCTGAACGCATGGGCACCGCCCACATCTTGACCTCGTGTTGGCTGGTGAACATGCTGCTGTATGTCATTGTTCCGATGCGTGTCACCAGCCAACAGTATGGCATGGAGAAAATGAGTTTCGGATACTGTGGGGCAGTTAGTACCAGAATCACGTATCCTCTGCATCTCATATTGTGGTCCTCTTACGATGTGTTGTGTTTGGGGCTCATGGTCTGGTCCAGTGGCTTCATGATCTTTATCCTGCACAGGCACAAGCACCAGGTTCAACACATCCGCAGCACCAACTATTCCCTCAGGTCCTGTCCAGAGTCCAGGGCCACCCGAGGCATCCTTGCCTTAGTGAGCACTTTTGTACTGTCTTATGTCCTCTCCTCTGTCTTTTCATTAACCATGGCTCTTTCCATTGGTCCTGGTGTGTGGCTGGTGAAGACCTCTGCTCTAATGGCTGCTTGTTTTTCTGTAGCTGCTTATATGCCCGCCAGCCACAGGATCTCTGTGCTCCTCTCTGCCTGCTGTGGAAGGAGTGTATACAGCTCTAGGCTGGTCAGGTAG

>Cricetulus_griseus_intact_V1R4_1

ATGGACTTCTGGATTCTGGCAACGAGAATTATTTTCTTGTCAAAAACAGTAACTGGAATTCTGGGAAATCTGTCTCTAATTTTCTACTATCTAGTCCTGTGCTGCAGAGAAAACACATTAAAGCCCACAGATGTGATGCTAATGAACATAATGTCAGCCAATGTCTTGATCATTCTCTCTACTGGAGTGCCCCAAACAATGGCTGTTTGGGGATTTAAGCATTTCTTGAATGATTTTGGGTGCAAGCTCCTTTTGTTCATTCAAGGATTGGGTAGGAGTGTGTCCATTGGAACCACCTGCCTCTTGAGTGTCTTTCAGGCCTTGACCATCAGTCCCAGGAAGTCCTGTTGGAAGCGTCATAAAGTCAAGTTTGAGAAGTACATCAATCATCACATTTTGCTCATTTGGATCTTGTACTTGTTCCTAAATTTCATTTATTTGGCATACACACTTGCCAAAAGGAATAGCAAAAATGTGACAAGAGAACGAGATTTTGGATATTGTTTTACTGCAGGGTGGGATCAAATTGGCGTTTTACTGTATGCAGTGTTGGTGGTGTTGCCAGAAGTCTTCTTTTCTGTGCTCATGACCTGGTCCAGTGGATTCATGGTTTTCATTCTGTACAGACACAGGCAGAGGGTTCAACATATCCGTAGCACTCAGGGTTCCATCAGAAACTCCCTTGAATCTAGAGTCACCCAGTACATTCTTATCCTAGTGTCTACATTTTTGGCTTTTTATACTCTCTCCTCCATCTTAAGAGGCTACATTTCTCTTTTAAATAGTCAAAATTGGTGGCTGGTAAACATCAATCGCCTTACCTCTCTGTGTTTTCCATTAATTGGACCCTTTGTTCTCATGAATCATTACTCTATTATGTCCAGGCACTGTTTGGGCTTCATAAGGAATAAAAATAATTTAATTTCATTTTAA

>Cricetulus_griseus_intact_V1R4_10

ATGATTTTGAAGTTTATTAAGGAAATAATTTTCCTCTTCATGACTATGGTTGGTACTCTGGGGAACATTTCTGTTTCTATGAACTATATGTTCAGTTGGCGGGGAGGCTCTGAGAAGAAACCCATACACCTTATTCTCATGCACTTGGCTTTCACAAACACCATAATCCTTCTTGCAAAAGGATTGCCAAAGACAATGGCAGCTTTGGGTTTGAAAAATGTCCTTAATGACATAGGTTGTAAGATCATCATTTACCTGGCAAGGGTGGCCCGTGGCATCTCCATCTGTACCAGCAGTCTCCTCACTGTGGTCCAGGCCATCATCATCAGTCCCAGAGCATCCGGGTGGAGGAGGTTCAGACCAAAGTCTGCATGGCACATCCTTCCATTCTTTCTTTTCTTTTGGGTACTCAATGCTTTAATAAGTATGAACCTAATTCATTCCATCACAAGCACAAGCCTGAATATATCACAGCTTGAGAGTGAAAACAACTATTGCCATTTTATGCTAGAAAGTCAGAAAACAAAATGGATTGTTCTCCCTCTCATGGTCCTGAGAGATGCAGTGTTTCAGGGTGCCATGGGAGGGGCCAGTGGCTACATGGCACTTCTTCTCCACAAGCATCACCAGCATGTGCTCTACCTTCAGAACTCCAAGCTTCTCTACAGAATTCCCCCTGAGCTGAGAGCTGCTCAGAGTGTCCTCCTGCTGATGCTCTGTTTTGTTTTCTTCTATTGGACTGACTGTGCCTTTTCTCTGTTTTTAAGTCTCTCATTAGTAAACAATTCCTTGATTGTAAACATTCAAAAATTTCTAGCTCTTGGTTATGCAACTTTTAGCCCCCTTGTGTTGATTCACAGGGATGTGTGCTAG

>Cricetulus_griseus_intact_V1R4_11

ATGGACTTCTGGATTCTGGCACTCAGAATTATTTACTTTTCACAAACCACAATTGGAATTCTGGGAAATTTCTCCCTTATAATCTACTATCTGGTCCTTTACGGCAGAGAATGTATGCTCAAGCCCACAGATTTGATACTGCTGAATTTAATGGCAGCCAATTCCTTGGTCATTCTCTCGACAGGAGTGCCTCAAACAATGGCAGTTTTTGGGTGGAAGGAGATCTTGGATAATTTTGGTTGCAGGCTCCTAGCATACACGCAAGGATTTGGGCGGAGTGTGTCCATTGGCATCACCTGCCTCTTGAGTGTCTTCCAGGCCATGACAATCAGTCACAAGGTATCCTGCTGTAAAGGACAAAAAGCAAAAGGTGCAAAGTACGTTGGCTGCTCCATTTTCCTCCTCTGTGTCTTCTACACCTTGATAAATTTGGTTCTCTTTCTGCACACATTTTTCAAAGTTAATAGCAAAAACATAACAAGAAAACTAGATTTGGGATACTGCTCTATTGTGGGGAGTGATGGAATCACTGATTCGATCTATGCAACATTTGTAGTTTGTCCTGAAGTCTTCTTTTCTTTCCTCATAGCCTGGTCCAGTGGCTTCATGATCGACATTCTGTACAGACACAAGCAGAGGGTTCAACACATCCGCAGCACCCATGGTTCCAGAAGAACCTCCCCTGAAACCAGAGCCACCCAAAGCATCCTGATCCTGGTGTCTGCCTTTCTGGCTTTCTGTACTGTCTCCTCCATCTTACGGGGCTGCGTTAGTTTTTTGCGTGATCATAATTGGTTATTGGTGACCCTCAATCGCATTATATCTATGTGTTTTCCTTCTTTAGGACCCTTTGTTCTCATTAATCATTGCTCCATTGTGTCCACGATTACTTTGATCTGGATCAGGAGTAAAAATAATTTCATCTTATTTTCACTACATAAATGA

>Cricetulus_griseus_intact_V1R4_12

ATGAACCTCAAAGATTTGACGATAGGAGTAGTGTTCTTACTTCAGAGTACAGTTGGAATTGTGGGAAATTTCTCTCTTCTTTCCTGCTACCTCATCCATTACTATACTGAACAGACATTAAAGACCACAGATTTCATTCTCACACACATGTTCACAGCCAACATCTTGATCATGCTCTCTAAAGGATTTCTCAATACAATGAGAGCTTTTGGGATGAAAGGGTTCATCAATCATTTTGGCTGTGAATTTCTTTTGTATATTCAAAGACTAGGCAGGAGCATGTCCATTGTCACCACTTGCTTCTTGAGTGTTTTCCAGGCTATCACAATAAGGCCTGGGATCTCCTTTTGTTTTATCCCTGAAGTCAAAGCTTCAAAGCATATTGTTCTGCTCACCTCCCTCTGCTGGAAGCTCTACATGTCAGGAAATATGATTTTCCCTGTGTATATGTATACCCAGGGCAATAGCAACATCTTAGCACATGAGAGTGATATGAAATACTGCTCCATTTCAGGTAACGATGCCCTTGGAAGCTTATTATATACAGTATTGTTTGTTCTTCCTGAAATTTTGCTTTCTGCCATTATTGTTTGGTCTAGCTGTTCCATGGTTGTCATTTTGTACAGGCACAACAAAAGGGTTCAACATATCCGCAGCTCCAGTGTTTCCTTCAGAACGTCTCCTGAGTACAGAGCTACCCACCGCATACTGGCCTTTGTGTCAACCTTTATAGGTTTCCATGCCCTCTCTTCCATCTTACAAGGTTGCATTGCTCTCATACACAATCCTCATTGGTGGCTGCAAAATATTACTGCAATCATTTCTATGTGTTTTCCTACTCTGGGGCCCTTCTTAATGAGCCATGACTACACATTGCAAAGGTTGTGCTTTACTAAGTAA

>Cricetulus_griseus_intact_V1R4_13

ATGCATTTCTGGAATCTGGCCATCAAAATAATTTTCTTCTCACAAACTACAACTGGAATTCTGGGAAATTTCTCTCTGTTTTACTACTATTTAATCTTGTATGGAGAATCCAAATTAAAGACTATAGATTTGATTCACACACACCTATTAGCAGCCAACACCTTGATCATTTTTTCTCGAGGAATGCCCCACACAATGGCAGCTTTTGGTTTGAAGCAGTTTTTAAATGATTTTGGATGCAGATTACTTTTGTACATTGAAAGAGTTGGCCGCAGTGTGTCCATTGGCACCACCTGTCTCTTGAGTGTCTTCCAGGCCGTCATCATCAGTCACAAGGAATCCTGTTGCAAGGATCAAAAAGTGAAAGCTGCTAAGTACATTGGCAGCTCCATCGTCCTCCTCTGGATCTTGTACACGTTATTAAATTTCGTTTTGTTAGTGTACCCAATTATCAAAAGGTATAGTAATAATGTGACCAGAAAAAAAGATTTTGGATACTGCTCCACTGTTGGGAGGAATAAAATCAATGACTCACTCTATGCAGCATTGGTGGTCTGCCCTGAAATCTTCTTTTCTGTGCTCATGGCCTGGTCCAGTGGCTCCATGATTGTCATTCTGTACAAACACAAGCAGAGGGTTCAGCACATCCGTAGACCTTCTGGTTCCAGCAGAACCTCACCTGAGTCCAGAGCCACCCACAACATCCTGGTCCTGGTGTCTACCTTTCTGGGTTTTTATACTCTCTCCACTATCTTACAAGGTTGCATGGCTCTTATGTATAATTACAGTTGGTTGCTGGTGAACATCAGTCACCTCACTTCTCTGACTTTTCCTTGTTTTGCCCCCTTTATTCTTATGAATCATTACTCCACAATATCAAGACTCGGTTTTGTCTGGATAAGGAATGTGAACTCACTCATTCTTAAATGTGTAAATGATATAATTTTTTGTGGTATCCAGTTGTTTACTCACTCATATGCCTAA

>Cricetulus_griseus_intact_V1R4_14

ATGGCAGCTGGTGAAGTGGCCATAGGAGTGATCTTTTTATCACAGGTGGTGTTTGGAATATTAGGCAACTCCTTCTTAGCCTACCATTATCTGTTGGTTTACATTATGGGATACAGGTTAAGGTTCACAGACTGGATTTTACAACATTTGGTTGTAGCCAACTTCTTGACTCTCATATGTAAAGGAGTTCCTGAGACAATAGCAGATTTTGGTTTGAAAGACTTCCTTGATGATTTAGGTTGCAAATTTCTTCCCTATCTTCACAGAGTAGGGAGGGGTGTGTCCATCAGCAGCACCAGCTTCGTGGGAGTCTTTCAGGCCATCACCATCAACACTAAGTGCTCAAGATGGACAAAGGATAAAGTCAAGTCTCCATGTTTCATTGCTTCTTGTGTGTACATGAGCTGGATCCTGTCCCTCATAGCAAACATCGGTATTCCTATGAATATGACTGCAAGATGGAGCCAAAGAAACATGACAAACCTAAAAGAGTATGGATATTGTGCTGCTGTTTATGTTGACAAAACTAGTGACATCCTCTATGTAGCATTACTTTCAAGTCCCGATGTCTTCTTCATGGGCCTGATGCTGTGGTCCAGCGTTTCCATGGTTTACATCCTCTACAGACACAGGCAGAGAATGAAACACATTCAAAGGAGCCACTTGTCCCTCAGGTCCTCCCCTGAGACCAGAGCCACAAAGACCATTCTGCTCTTGGTGAGCACCTTTGTCTGCTTTTATGCAGTTTCTTGCCTCTTGCATGCTAGTTTGACTCTGATGAATGATCCAGGCTGGCTCCTGGTGAACATGGCTACAATTGTCTCTGGGTGTTTCCCAACTGTGAGTCCCTTTCTGCTGATGATTCATGACTCCCATGAAAACCCTTTCTGCTTTGCTCCTGCAAGGAATAGAAAACAATAA

>Cricetulus_griseus_intact_V1R4_15

ATGACATGGAATAACATTATCCAGACAATAATCTTCCTTTGTCTTATTGGACCTGGAATTGTGGGGAATATCCTAATGTTTGAGAGACATGTATACAATTCTGTCTTGGGGACTGAGAAAAAGCTTGTGGACCTTATCCTAATGCACTTGGGACTTTCTAATATGATCATTATTTGTACAACAGGGATCAGAGATATCGGTACAGTGTTTTATTTCAAAAACTTCCTTGGAGATTTTGGCTGTAAAGCTGTGGTTTTTCTGGCAAGGATGGCTCGGGGCCTTTCCATCTGTACCACCTGTCTTCTCAGTGTTGTCCAGGCTGTCACCATCAGTACCAGGACCACCATTTGGACAAAGCTAAAACCACAGTCCTCACAGCAAGTTCTTACCTATTTCTTGTTCTTTTGGATCTTTAATGTTCTCACAAGTTCCAACTTGTTGTACTATATAACAGCAGGCAGTGACAACAACAGATCTAAAGTTTCAGGGTATATTGGGTATTGCTATATGCTTCCATCCAAGCTCACAATTAAGTGGCTTTTCCTCTCGCTCATGGCTGTTCGTGATCTGATCTTTCAGAGTCTCATGGGCTGGAGCAGTGGATACATTTCTTTTTACCTGTATAGACATCACAAGCAAGTCCTCTACCTTCATAGCTGCAGGTTGGCAAATAGTTTCAGTGCAGAGATCAGAGCTGCACAGAGTGTTCTCCTTCTCATGGCCTGTTTCCTTTTCTTCTATTGGACAGACTTTGTTTTCTCCTTTTACACAGGTTCCATGGTGACTCATGATTCCGTTATACTAAATATTAAAACATGTCTAGTACTTGGTTATGCTGTTCTCAGTCCCTTTGTCCTGAGGAGCAGAGATGTCCCTGTTGCTAAATCCTTGTGTTCTCACTGA

>Cricetulus_griseus_intact_V1R4_16

ATGTCGTCATTAAATAATGTCTTTTATTTCCAAGCTGGACTTGGTGTTTTAGCCAATATGTTTCTCCTTTTTTACTGCACTTTCATAATCCTGTGTCACAGACCTAAGCTCATGGACCTGCTCTCCTGTCAGCTGCCCTTCATCCCCATAATGCTGGTCCTCACTGGAGGGGATATGTGGCTTACAGATGTTTTTGAATCATTCAACATTGAGAATGACATCAAATGTAAGGCAACTTTTTATATAAACAGGGTGATGAGAGGCCTCTCCATCTGCATCACCTGCCTCCTGAGTGTGTTCCAGGCTGTCACTATCAGTCCCAGTACCTCTTTTTTGGCAAACTTTAAACTTAAGCTAAAAAAATACATGACCTATGCCTTCTTCTACATTTGGTCTTTCAATGTGTCATTCAGTAGCATCCTGGTCTTCCATGTTCATGCTTTTGCCAATGTCAGTGAGCCCAATCAGATGAAGGTCACCAAATATTGCTCACTCTTCCCCATGAACTACATCATCAGGGCACTGATTTTAACGGTGACAACCTCCAGAGATATATTTCTTGTAGGAGTTATGCTGACCACAAGTGCATATATGGTGATTATCTTGTTCAGACATCAGAGGCAATGCAAATATCTTCACAATCCCATCAGCCACCTGAGAGTGTCCCCTGAGAAAAGGGCCACACAGACCATCTTGCTTCTGGTGGCTGTCTTTGTGGTCATGTACTGGGTGGACTTCATCATCTCATCCACCGCAGTCCTGTTATGGATGTACCACCCAGTCATCCTGACTGTTCAGAAGTTTGTGGTGAATGCCTATCCCACAATTACTCCTTTGGTTCAAATCAGTTCTGATAACAGAATAATCAACATGCTGAAAAACTTTCGGGCAAAGTGCCACCAGGTGTTTTAA

>Cricetulus_griseus_intact_V1R4_17

ATGTTTGCAATAAACTACATACTTTATTTCCAAGCTGGACTTGGAGTTCTAGCCAATATGTTTCTCCTTTTTTTCTACACTTTCATAATCCTAGTGCATAGATCTAACCTCATTGACCTGTTCTCCTGTCAACTGACCTTCATCCACACAGTACTGGTTCTCACTGGAGGGGATATTTGGCTTACAGATGTATTTGAGTCACTGAACATTGAGAATGACTTCAAATGTAAGACAACTTTTTACATGAACAGAGTCATGAGAGGCCTCTCCATCTGCACCACCTGCCTACTGAGTGTGTTCCAGGCTGTCACTATCAGTCCCAGTACTTCTTTGCTGGCAAAATTTAAATATAAACTAAAAAAATACATGATCTATGCTTTCTTCTATATGTGGTTTCTCAACTTGTCATTCAGTAGCAACCAGATCTTCTATGTTGGGGCTTTTACCAATGTGAGTGAGACCAACCAGATGAAGGTCACCAAACATTGCTCACTCTTCCCCATGAACTACATCATCAGGGTGCTGATTTTAACAGCAACAACCTCCAGGGATGTATTTCTTGTAGGAGTTATGCTGACCTCAAGTGCATACATGGTGATTATCTTATTCAGACATCAGAGGCAATGCAAATATCTTCACAGCCTCAGCCACTTGAAAACATCTCCTGAGAAAAGGGCCACTGTGACCATCTTGCTTCTGGTGGTTGTATTTGTGGTCATGTACTGGGTGGACTTCATCATCTCATCAACCGCAGTCATGTTATGGACGTACCACCCAGTCATCCTGACTGTTCAGAAGTTTGTGGTAAATGCCTATCCCACAATTACTCCTTTGGTACAAATCAGTTCTGATAACAGAATAATCAATATGCTGATAAACTTGTGCTCAAACTGCCACCAGGATTTTTTAAAAGTGTAA

>Cricetulus_griseus_intact_V1R4_18

ATGGACTTCAGGGGTGTGGCAATAGGAATAGTGTTCTTATTTCAGAGCACATTTGGAATTGTGGGGAATGTCTCTTTTCTTTCCTACTACCTCCTTCACTACTGCACTGCACAGAAGTTAATGACCAAGGATTTGATTCTCACACACCTGTTCACAGCCAATTCCTTGATCATTCTTTCTAAAGGAGTGCTAGAGATAACACATGCTTTTGAGATGAAATGGTTTGTCAGTGATATTGGCTGCAAATTTCTTTTGTATATTGAAAGACTTGGCAGGAACATGTCCATTGGCACCATCTGTGTTTTGAGTGTCTTTCAGGCCATCACCATCAGTCCCAGTGTCTCTCGTTGGAAGGATCTGAAAATAAAAGCTGCAAAGTATACTGGACTCTCCATTTCCCTCTGCTGGGTTCTGTGCATGGGAATAAATATGATTTACCCTGTGCATACATATGCAATGGAAAGCAGCAAAAACATGACACTTAAACAAAATTTGATACATTGCTCCACTCCAGGATATAATGCAGTCAGAGGTTCATTGTATATGGCTTTCTGTGTATTCCCTGAAATTTTGCTTTCTGTCATCATTGCCTGGATGGGTGGAGCCATGTTTATCACTTTGTACAGGCACAAGCAAAGAGTTCAACATATCCGAAGTACTCATGTTTCAAATATTACATCCCCTGTGTCCAGAGCCTCCCGGAGCATTGTTGTCCTGGTGTTCACTTTTCTAGGTTTTTATGCTCTCTCCTCCATCTTACAGGGTTGGAATGCTCTTGTATATAATAGTGGATGGTGGCTAATGAACATCACAGCTGTCATTTCTATGTGCTTTCCCACCTTAGGACCTTTTATTGTGAGTCATACCTCCACTTTATTCAAATTCCACTTTTTCTGCATGAGGAACCAAAACATCACATAA

>Cricetulus_griseus_intact_V1R4_19

ATGAACCTCAATGATTTGACGATAGGAATAGTGTTCTTACTTCAGAGTGCAGTTGGAATTGTGGGGAATTTCTCTGTTCTTTCCTGCTACCTCATCCATTACTACAATGAACAGACATTAAAGACCACAGATTTGATTCTCACACACATGTTCACAGCCAACATCTTGATCATTCTCTCTAAAGGATTACTCCATACAATGAGAGCTTTTGGGATGAAAGGGTTCATCACTCATTTTGGATGTGAATTACTTTTGTATATTCAAAGACTAGGCAGGAGCATGTCCATTGTCACCACCTGCTTCTTGAGTGTTTTCCAGGCTATCACAATCAACCCTGGGAACTCTTTTGGTTTGATCCTTAAAGTCAAAGCTCCAAAGCATATTGTTCTGCTCACCTCCCTCTTCTGGAAGCTGTACATGTCAGGAAATATGATTTTTCCTTTGTATATGTATACTAAGCAGAACAGGAACATCTTGGCACATGAGAGTGATATGAAATACTGCTCCATTTCAGGTAACGATGCCCTTGGAAGCTTATTATATACAGTGTTGTTTGTTCTTCCTGAAATTTTGCTTTCTGTCATCATTGTTTGGTCTAGCTGTTCCATGGTTGTCACTTTGTATAGGCACAACCAACGGGTTCAACATATCCGCAGCTCCAGTGTTTCCTTCAGAACATCTCCTGAGTACAGAGCCACCCACCGCATACTGACCTTTGTGTCAACCTTTATAGGTTTCCATGCCCTCTCTTCCATCTTACAAGGTTGCATTGCTCTCATACACAATCCTCATTTGTGGCTGCTGAATATTAATGCAATTATTTCTATGTGTTTTCCTACTTTGGGGCCCTTCTTAATGAGCCGTGACTACACATTGCAAAGATTGTGCTTTACTGAGTAA

>Cricetulus_griseus_intact_V1R4_2

ATGCTTCCAAGTAATACCATCTTGGGGGTCTGTCTGATATCTCAGTTATGTGTTGGTGTCACAGGGAACTCGTTACTGTTCATTTTGTACATATATACTTTCTTCTTTAAGCCTCAATTTAAGAAGTTGATCGATTCCATTTGCATGCACCTGACAATAGTTAATGTGCTGATGATCATATTTATGTTGATATCCCATATCATGTCATCCATTGGAGTACACAAATTTCTGGATGATGCTGGCTGTAGGGGAGTGCTATTTATATCCAGAGTCTCCCGGGGTATGTCCATCAGTACCACCTCTATTCTGAGCACATTTCAAGTCATCACCATCACTCCCAGTAATTCTAGGTGGGCGTGGCTTAAGCCTAAACTCTCCAAGTTGACTTTGTCATCCTTACTTTGCTCCTGGCTCATTAACCTGCTCATCTATGCATATATGGTTCCATTGGTAATAGCCAAAACCAATTCTACTCACTTTGGCAATGGATATTTGGATCCTTACTGTCAAAACAAGCACTTTGGGGACCAAAATTCAGGGTCATTTTTGATTGTCATTTTCATTTATGATTTCTTCTACGTGGCCATCATGATGTGGACCAGCCTTTACATGGTAATTGTCCTCTACAGACACCGTAAGAGAGTCCAGCATCTCCACAGCACAAGCCTGTCCAGCCAGCCATCTCCTGAACGCAGAGCCACTCACTACATCTTGTTGCTGGTAATCTGTTTTGTGTTCATTTATTGGTTAAACAATTTCATCACCCTTTCAGGAGTTTTTGTACAAGTAAAAATTCCAAACTTGGAGGGATTTAATGCCATTTTGGCAACATGTTACCCAACCATCTGCCCTTTTTTACTGATGAAGAATAATAAACTTGTTTTGCAATTCACTTCTTTTTCTGAAAGGAGGATGGCCTGTTTTCAAAGTGCACTCCGCGGCTGA

>Cricetulus_griseus_intact_V1R4_20

ATGTATTTCTGGACTCTGATCATCAAATTAATTTTTTTGTCACTAACTACAACTGGAATTCTGGGAAATTTCTCTGTGTTTTACTACTATCTGATCTGCTATGGAGATTGTAAATTAAAGACTGTAGATTTTATTCACGTGCACCTAATGGCAGGCAACACCCTGATCATTCTCTCTAAAGGAGTGCCCCACACGATGGCAGCTTTTGGTTTGAAGCAGTTTTTAAATGATATTCAATGCAGATTACTTTTGTACATTGAAAGAGTTGGCCGAAGTATGTCCATTGTCATCACCTGCTTCTTGAGTGTCTTCCAGGCCGTCACCATCAGTCAGAAGGAATCCTGTTGTAAGGATCAAAAAGTCAAAGCTGCTAAGTACATTGGCTGCACCCTGTCCCTTCTCTGGGTCTCGTACATATTGATACATTTCATTTTATTTGTGAATCCACTTATCAAAAGTTATAGTAACAATGTGACAGGAAAACAAGATTTTGGACACTGCTCTACTGAAGGGCGGGATGGAATCAATGACTCACTCTATGTAGCATTGGTGGTGTGCCCTGAAATCTGCTTTTCGTTGCTCATGGCCTGGTCTAGTGGCTCCATGATTGTCATTCTGTACAGACACAAGCAGAGGGTTCAGCACATCCGTAGAACTTGTGGTTCCAGCAGAACCTCCCCTGAGTCCAAAGCCACCCAGAACATTCTGGTCCTGGTGTCTACCTTTCTGGCTTTTTATACTGTCTCTGCTATCTTACAAGGCTGTGTGGCTCTTTTCTCTAATCCTAGTTGGTGGCTGGTGAATATTAAACACATCATTTCTCTGTGTTTTCCTTGTTTTGGACCCTTTGTTCTTATGAACCATTACTCCATGTTGCAAAGACTCAGTTTGGTCTGGATGAGGAATATAAACTCACTTATTCTTAAATAA

>Cricetulus_griseus_intact_V1R4_21

ATGGCAGCTGGTGAGGTCACAGTAGGAATGATCATTTTATCAGAGACTGCAGTTGGTATCCTGGGCAACTCCCTCTTAGCCTACCATTATCTGTCACTTTACTTCATTGGGAGCAGGATAAGGTTCACAGAGTGGATTCTACAGCATTTGGTTGTAGCCAACTTCTTAACGCTCATATGTAAAGGAGTTCCTGAGACAATGGCAGCCTTTGGTTTGAAAGACTTCCTGGATGATTTTGGGTGCAAACTTCTCCCCTATCTTCACAGAGTAGGTAGGGGTGTGTCCATCAGCAGCACCAGCTTCCTGGGTGTCTTTCAGGCCACCACCATCAGCTCTACGTGTCTAATATGGAAAAAGGATAAAGTCAAGTCTCCAAGTTACATTGCTTCTTGTGTGTACCTGAGCTGGATCCTGTCCCTCATAGCAAATATTGCTTTTCCTATGAACATGACTGCAAGATGGAGCCATAGAAACATGACAAGTCTAAAACAGTATGGATACTGTTCTGCTGTTTATCTTGACAAAACGAGTGACCTCCTCTATGCAGTATTACTCTCAGGTCCGGATGTCTTGTTCATGGGCCTGATGCTATGGTCCAGCGTTTCCATGCTCTACATCCTCTGCAGACATAAGCAGAGAATGAGATGCATTCATAGGAGCCAATTTTCCCTCAGGTCCTCCCCTGAGACCAGGGCCACAAAGACCATTCTGCTCTTGTTGAGCACCTTTATCTGCTTTTACACACTTTCTTCCCTCTTGCAGGCTAGTTTGGCTCTTTATGATCCAGATTGGCTCCTGGTGAAAATGGCTACAATTGCCTCTGGATGTTTCCCAACTGTGAGTCCCTTTCTGCTGATGAACCATGACTCCCATGAAAATCCTTTCTGCTTTGCTTATGCAAGGAGTAGAAAACAGCAGCCATGTGGGCAACAAGTAAATTGTCTGTTTCTAATTAATGTTCAATTTTGCCTCTAA

>Cricetulus_griseus_intact_V1R4_22

ATGAATATCTGGAACCTGGCAATCAGAATCATTTTCTTATCACAAACAACAACTGGAATTCTTGGAAATTTCTCTCTTTTGTTTTACTATCTAGTACTTTATTGTAGAGAACACACATTAAAGCATACAGATTTGATTCTCACACACTTAATGGCAGCCAATGCCTTGATCATTCTCTCTATAGCAGTGCCCCAAATAATGGCAGTTTGGGGATTTAAACATTTTGTGAATGATTTTGGATGTGTGTTCCTATTGTACATTCAAGGATTTGCTCGAAATGTGTCCATTGGTGCAACCTGCCTTTTGAGTGTCTTCCAGGCAATGACCATCAGTTCCAGGAAGTCATGTTGGAAGGATCATAAAGCCAAAACTACAAAGTGCATTCACTGTTCTGTTTCCCTTCTCTGGGTCTTCTACATGCTGATACGTTTCATATTCTTGATGAACATATTTATGGAAATGAATAGCAAAAACATGACAAGAAATCGAGATTTTGGATATTGCTCTACTGTAGGCTGGGATGAAATCATAAACTCACTCTATACAGCATTGGTGATGTGCCCTGAAATCTTTTTTGCTGTGCTCATCACCTGGTCCAGCGCCTCCATGATTGTCACCTTGTACAGACACAAGCAGAGTGTTCAACACATCAGAAGTTCTCATGGTTCCAGGAGATCCTCCCCTGAGTCCAGAGCCACCCAGAACATCCTGGTGCTGGTGTCTACTTTTCTAGCTTTTTATAGTCTCTCTACCATCTTGCGAGGCTGCATTGCTTTTTTGTATAATCACAATTGGTGGCTTGTGTACATCTCTCGAATTACTTCTCTTTGTTTTCCCTGTTTTGGACCCTTTGTTCTTATGAGACATCACTCAGTTTTGTCCATATTTAATCTGGAATGGTTAAGAAAACATTTTTCCTGA

>Cricetulus_griseus_intact_V1R4_23

ATGTCCTCATTAAAGAATGTCCTTTATTTCCAATCTGGACTTGGAGTCCTCGCCAATATATTTCTCCTTTTTTTCTACACTTTCATAATCATAGTGCATAAATCTAAGCCCATGGACCTGATCTCCTGTCAACTGACCTTCGTCCACATAGTGCTGGTTCTCACTGGAGGGGATATTTGGCTTACAGATGTATTTGAGTCACTGAACATTGAGAATGACTTCAAATGTAAGACAACTTTTTACATAAACAGAGTGATGAGAGGCCTCTCCATCTGCATCACCTGCCTCCTGAGTGTGATCCAGGCTGTCACTATCAGTCCCAGTACCTCTTTCCTGGCAAAATCTAAACATAAACTAAAAAAATACATAATCTATGCTTTCCTCTTTTTGTGGTTTTTCAACTTGTCATTCAGTAGCAACCGGATCTTCTATGTTGGGGCTTTTACTAATGTGAGTGAGACCAACCAGATGAAAGTCACCAAATATTGCTCACTCTACACCATGAACTCTGTCATCAGGGTACTGATTTTAACAGCAACAACCTCCAGAGATGTGTTTCTTGTAGGAGTTATGCTGACCACAAGTGCATACATGGTGATTATCTTGTTCAGACATCAGAGGCAATGCAAATATCTTCACAGCCTCAGCCACTTGAAAGCATCTCCTGAGAAAAGGGCCACTGTGACCATCTTGCTTCTGGTGGTTGTCTTCGTGGTCATGTACTGGGTGGACTTCATCATCTCATCCACTGCAGTCCTGTTATGGATGTACCACCCAGTCATCCTGACTGTTCAGAAGTTTGTAATGAATGCCTATCCCACAATCACTCCTTTGGTACAAATCAGTTCTGATAACAGAATTTTCAATATGTTGACAAACTTGTGGTCAAAGTGCCACCAGATTTTTAAAAAAGGGTAA

>Cricetulus_griseus_intact_V1R4_24

ATGTATATCTGGACTCTGATCATCAAATTAATTTTTTTGTCACTAACTACAACGGGAATTCTGGGAAATTTCTCTCTGTTTTACTACTATCTAATCTGCTATGGAGAATGCAAATTAAAGACTGTAGATTTGATTCACATTCACCTAATGGCAGCCAACACCTTGATCATTCTCTCTAAAGGAGTGCCCCACACGATGGCAGCTTTTGGTTTGAAGCAATTTTTAAATGATATTCAATGCAGATTACTTTTGTACATTGTAAGAGTTGGCCGAAGTATGTCCATTGTCATCACCTGCTTCTTGAGTGTCTTCCAGGCCGTCACCATCAGTCAGAAGGAATCCTGTTGTAAGGATCAAAAAGTCAAAGCTGCTAAGTACATTAGCTGCACCCTTTCCATTCTCTGGGTCTCATACATTTTGATACATTTCATTTTCTTTGTGAATCCACTCATGAAAAGTTATAGTATCAATATGACAAATAAACGAGATTTTGGACACTGCTCTACTGCAGGGCGGGATGGAATCAATGACTCACTCTATGCAGCATTGGTTATGTGCCCTGAAATCTTCTTTTCGTTGCTCATGGCCTGGTCTAGTGGCTCCATGATTGTCATTCTGTATAGACACAAGCAAAGAGTTCAGCACATCCGAAGCACTTGTGGTTCCAGCAGAACCTCCCCTGAGTCCAGAGCCACCCAGAACATCCTGGCGCTGGTGTCTACATTTCTGGCTTTTTATACTCTCTCTGCTATATTACAAGGCTGTGTGGCTCTTTTGTCTAATTCTAGTTGGTGGCTGGTGAACATCAATCATGTCACTTCTCTGTGTTTTCCTTGTTTTGGACCCTTTATTCTTATGAATCATTACTCCATGCCTAGTCTCAGCTTGGTCTGGATAAGGAATGTAAGCACACTCATTCTTAAGTATGTAAATGATATAATTCATATGGTATCCAGTTGTTTAGTCACCCATCTCCCTCACAAAGTCAGTGCAGAAAGTTAA

>Cricetulus_griseus_intact_V1R4_25

ATGGACTCTAGAAATTTAGGAACAGGAATAATGTTCTTGATACAGAATATTGTTGGAATTCTGGGAAATGTCTCTCTTCTTTCCTACTATCTAGTTATATATAAAGAGAAACACAAAGTAAAGACCTTGGATTTGATTCTCATCCATCTGATCATGGTTAACATCTTGATTATTCTCTCTAAAGGAATGGGCAACACAATGATAACTTTTGGGTTGAAACATTTCTTCAATGATTGGAGCTACCAAATTTTTATGTATGTTCAAAGAGTTTTCAGAAGCATGTCCATTGCCACAATCTGTCTCTTGAGTGTCTTCCAGGCCATCATCATCAGCCCTAGAAACTCTTGTTGGAAGAATCTTAGAGCCAACTCTCCCAAGGACATTGGTCTCTGCATTTCTCTCTGCTGGTTATTGTACATAATGGTAAATGTTCTGTTTCCTTTGTATATGTCCATAAAATTAAGAAAGAAAAACAGAACAAAAGAGACAGGTTTTGAACTGTATACTGTTGTAGGTCATGACAGAGTAACAGTCTCTTTATATACAGCTTTCTTTGTGTTTCCTGAACTTCTATTTTCTGTCCTGATCACCTGGTCTAGCATCTCAATGATTGTCTTTTTGTATAGGCATAAACAGAAAGTTCAACACATCCGAAGCGCTTGTGCTTTCCACAGTAACTCTCCTGAGTCCAGAGCCACCCAGAACATCCTTGTCCTTGTGTTTACCTTTTTGGCTTTTTATACCCTCTCTACCATCTCACATGGTTGCAGTGCTCTATTGTCTCATCAAAATTGGTGGCCAATGACAATCACAAGCATTATAAGTTTGTGTTTTCCCACTTTAAGCCCCTTTGTACTTATGAGTCAATCCTCCCCTCTCTTCAGACACTGCATTCTTTGGATAAAGGGTTCAGAGTCATCTAATGTTATTATAGACATTTAA

>Cricetulus_griseus_intact_V1R4_26

ATGGACTTCTGGAATCTGGCGACTAGAGTCATTTTCTTATCACAAATTACCACGGGAATTATGGGGAATTTCTCTCTAATTTTCTACTATCTAGTCCTTTACTACAGAGAAAGCACATTAAAACCCACAGATTTGATTCTTTTGAACCTAATGGCAGCCAATGCCTTGATCATTCTCTCAGCAGGAGTGCCCCAAACAATGGCAGTTTGGGGATTGAAGAAGTTCTTGAATGATTCCGGATGCAAGATCCTACTGTACATTCAAGGATTCAGTCGAAGTCTGTCCATTTGTACTACCTGCCTCTTGAGTGTCTTCCAGGTCATGACCATCAATCCCAGGAAATCCTGTTGGAAAGGTCATAAAGTCAGAACTGTAAAGTATATTGGCTGCTCTCTTTCCCTGCTCTGGGTCTTGTATATGTTGATAAATTTCATTTTCGTTATGTACCCATTTACCAAAATTAATAGCAAAAATGTGACAAGAAAAAGAGACTTTGGATACTGCTCTATTGGAGGACATGACGAAATCAGTGACTCCCTCTATGGAACATTGGCGATGTGCCCTGAATTCTTCTTTTCTCTGCTCATTGCCTGGTCCAGTGCCTCCATGATTGTCACTCTGTACAGACACAGGCAGAGGGTTCAATACATCTGTAGTTCTCATAGTTCCAGTAGAAAATTCCCTGAGTACAGGGCCACTCAAAACATTCTCTTCCTGGTGTCTACCTTTCTGGCTTTTTATACTGTCTCTACCATCTTGCGAAGCTGCATTGCTTTTTTGTATAACCACAACTGGTGGCTGGTGGACATCACTCACCTCACTTCCCTGTGTTTTCCCTCTTTTGGACCCTTCATTCTTAAGAGTCATTACTCTATTGTGTCAAGATTCACTTTGGCCTGGATAAGAAAAAAATTATCTCCTTATTTTATTATAAGTATGTAA

>Cricetulus_griseus_intact_V1R4_27

ATGTATTTCTGGGCTCTGACACTCAAAATAATTTTCTTATCACTAACTGCAATTGGAATTCTGGGAAATTTCTCTGTGTTTTACTACTATCTGTTCTGCTATGGAGATTGCAAATTAAAGGCTGTAGATTTGATTCACTGGCACCTAATGGCAGCCAACACCCTGATCATTCTCTCTAAAGGAGTGCCCCACACGATGGCAGCTTTTGGTTTGAAGCAGTTTTTAAATGATATTCAATGCAGATTAATTTTGTACATTGAAAGAGTTGGCTGTGGCTTTTCCATTGCCTCCACCTGCCTCTTGAGTGTCTTCCAGGTTATCAGCATCAGTCATAAGAAATCCTGTTGTAAGGATCAAAAATTCAAAGCTGCTAATTATATTGGCTGTTGCCTTTCCCTTCTCTGGGTCTCCTGCACATTAATACATTTCATTTTCTTTGTGTATCCAATTATAAAAAGGTATAGTATCAATGTGACAAGTAAACGAGATTTTGGACACTGCTCTACTGTAGGGCGGGATGGAATCAATGACTCACTCTATGTAGCATTGGTGGCGTGCCCTGAAATCTTCTTTTCGTTGCTCATGGCCTGGTCTAGTTGCTCTATGATTGTCATTCTGCACAGACACAAGAAGAGGGTTCAGCACATCCGCAGCACCCATGGTTCCATCAGAACCTCCCCTGAATCCAGAGTCACCCAGAACATCCTGGTGCTGGTGTCTAATTTTCTGGCTTTTTATACTCTGTCCTCTGTCTTACAAGGCTGTGTGGCTCTTTTGTCTAATCCTAGTTGGTGGCTAGTGAACATCACTCACCTCGTTTCTCTGTGTTTTCCTTGTTTTGGACCCTGTGTTCTTATGAATCGTTACTCCATAATGCCAAGTCTCAGTTTGGTCTGGATAAGGAACGTGACCACACTCATTCTTAAGTATGTGAATGATATAATTCTTATGGTATCCAGTTGTTTAGTCACCCATCTCCCTCACAAAGTCAGTACAGAAAGTTAA

>Cricetulus_griseus_intact_V1R4_28

ATGAAGTTGAATAACATTATCCATGCAATAATTGTCCTTTCTCTTGCTGGACCTGGAATTGTGGGAAATAGCCTAATATTTGTGAAGCACATATACATCTCTACCTTGGGGACTGAGAAAAGGCCCGTGAACCTTATCCTCATGCACTTGGCATTTTCTAATATGATCATTATTTGTACCACAGGGATCAGAGACATAGCCACAATGTTTTATTTCCAAAACTTCCTAGGAGATATTGGCTGTAAAGCTGTGGTTTATCTGGCAAGGATGGCTCGGGGCCTTTCCATCTGCACCACCTGTTTCCTCAGTGTGGTCCAGGCTGTCACCATCAGTCCCAGGACCACCATTTGGACAAAGCTAAAACCACAGAACTCATTGCAAGTTCTTCCCTTTCTCCTCCTCTTTTGGATGGCTAATGTTCTCATAAGCTCCAACTTGCTCTGCTACATCAAAGGAAGTGGTGGCTTGAACACTTCTATACCTGGAACATTCATTGGCCATTGCTATATGCTGCAATCAAGACATGTAATCAAGTGGCTATTCCTGTCTCTCATGGCTCTTCGTGATGTCATCTTTCAGAGTCTGATGGGCTGGAGCAGCGGGTCCATGGTGTTCCATCTTTATAAGCATCACAAGCGGGTCCTCTACCTTCACAGCTCCAGGTGTGAAAACAATTCCCCTCCAGAAATCAGAGCTACCTGGAGTGTTCTCATTCTAATGGCCTGCTTCCTTTTCTTTTATTGGGTCAATTTCATTCTCTCCTTCTACACTGGTTTCACAGCAACACATGATTCTATTTCACTAAATATTAAGACATTTTTAGAGCTTGGTTATGCTGGTTTTAGCCCCTATGTTCTGATCAACAGAGATGCCCGTGTTCCTAATATCTTGCATGCTCACTGA

>Cricetulus_griseus_intact_V1R4_29

ATGACTCCTGCAAAATTAGCCATGGGAATTTTCCTCTTCTCCCAGATTACAGTAGGCATGCTTGGCAATTCCTCAATACTGTTTTATCATGTCATTTTGATATTCACTGGAAAACATTTAATGCCCAAAGACCTGATCATAGGGCACTTGACTTTTGCCAACTGCTTGTCTATCATCTCAAGAGGAATTCCACAGACAATGTCAGATTTTGGATTTAAGTATTTCCTAGATGACATTGGATGTAAATTTATAATGTACATTTACCGAATAACAAGGGGGGTGTCCCTGTATGTCATGTGCCTCTTGAGTTGTTTCCAAGCTATTACAATCAGCCCCAACAACTCCAGGTGGATGAGGCTTAAACACAGAGCCACCAAGTTCATCAGTCCCTCCTGCTCACTCAGCTGGCTTGTGCACCTACTTCTAAACATCTCGACACCAGCAAAAGTGTCAGGCCCCATTTTCATCAACAATGCAACTAGCAGGATAAGTTATGGATACTGCTCATGGTTTGCTTCTGGTAACGTGGCAACGGCATTGTATTTGTTCTTACTGTGCTTCACGGATGGTCTGTGTCTGGGTCTCATGGCCTGCTCAAGTGTCTCCATGGTGACAATCCTCTACAGACACAAGAGACAAGTCAAGCATATCCATAGTGCTCAACACTGTCTAAAAGTTTCACCTGAGGACAGAGCTGCCCAAACTATCCTCACCCTGGTGTGCATATTTGTCATCTCTTACTCATTCTCTTCCATTATGGTCATCTTTATGAACTACTCCAAAGGTCAAATGCTGTGGGGCATAAGTGTATTTCTATTTGTAGAAATATGCTTTCCCATATTTTACCCTGTTGTTCTCATGTGCAATATTAAATATATTTCCAGCCTGTCTTTAACCTGCTGTGGTAAGAGGTAG

>Cricetulus_griseus_intact_V1R4_3

ATGGCTACCACAGATGTGGCTATTGGAGTGATCTTCTTAGCACAGACTGTCATTGGAATTCTGGGCAATTCCTACCTCTTCCACCATTATCTGTTGGGGTACAGGTTAAGGTCCACAGACTATATCCTGCAGCACTTAATTACAGCAAACTTCCTAAGTCTGCTGTGTACAGGAGTGCCCCAGACAATGGCAGCTTTCGGTATGAAAGACTTCCTCCGGGATATCGGGTGCAAACTAGTTTTCTATCTTCACAGGGTGGGGAGGACAGTATCCATCAGCAGCACAAGCTTCCTGAGTGTCTTCCAGGCCATTGCCATCAGTCCCATGGGCTCCACATGGGCAAAGTGTAAATTCCAAGCTCCCAGATACATTGGCTCTTCCCTATGTTTGACCTGGATCCTCTCCCTTCTTGCCAACGTTGCTTTTCCTGTGCACATGACTGGAAAATTTGGCAACGTAAACATCACAAGTCTAAAAGAGTTTGAATACTGTTCCGTTGTCCGTAACGACAAAGTCAGTGACATCCTCTATGCAGTATTACACTCAAGTCCTGATGTGTTTTTCGTGGGTCTGATGCTGTGTTCCAGCAGCTCCATGGTTTGCACCCTCTACAGACACAAGCAGAGAATGAAACATATTCATAGGAGCAACTTCTCCCTCGGGCCCTCCCCTGAGACCAGAGCCACAAAGACCATTCTGCTCTTGGTGAGCACCTTTGTCTGCTTTTACACAGTTTCCTGTCTCTTTCATATTCAGTTAGCTCTCATTTATCGTCCCACATGGCTCTTGGTAAAAGCTGCTGCAGTTGTCTCTGTGTGTTTCCCAACTGTGAGTCCCTTTCTGATGATGAGCCAAAACTCCTGTGCATCTTCACTCTGTTTTCAGTGTGCAAGGAATAGAAAGTACTAG

>Cricetulus_griseus_intact_V1R4_30

ATGGAAGCAAGCGACAAAGCCATAGCAGTGATGTTCTCAACCCAGACTTTGGTTGGAGTTCTGGGAAATCTTTCCCTTCTCTGCCGATACATGTTGTTTTGCTTCTCAGGGTTCAGTTTCAAGTCCACAGACTTGATCCTGTGGCACATGATTGTAGCCAACGTCTTAACTCTGCTGTGTAAAGGAGTGCCTCAGACAATGGTCGCTTTGGGTTTTAAAGACTTCCTTAATGATTTTGGATGCAAGCTCCTTGTCTATCTTCACAGAGTTGGGAGGGGTGTTTGCATGAGTAGCACCTGCTCCCTGAGTGTCTTCCAGGCCATTACTATTAGCCCTTGGAACTCCTTATGGGCAGAGCTTAAGGCTGAAGCTCCCAAGTACATTAGTTCTTCTTTGTGCCTTAGTTGGATCCTGTATGTGCTCATCAGCATTTTCTCTCTTATGTATATAAGTGCAAACTACAAGAACAACACAAAAAATGTAAAAGATTTGGGATACTGCTTTGCTGTTCGGCCTGACAATATCATCTATATACTGTATGCCATAATACAGTCAGTTCTTGATTTAATTTTCGTGGGTCTCATGCTCTGGGCCAGCAGCTCCATGTTGTTCACCCTGCACAGACATAAACAGAGGATGCAACACATGCCCAGGACTAATGTGTCCTCCAGACGCTCCACTGAGTCCAGAGCTACTGAAACCATCCTTCTCCTGGTGAGCACCTTTGTCTGCTCTTACACGCTCTCCTGCATGCTTACCCTTTCTGTGACACTCCTTTTGAATCCCAGCTGGTCTCTGGTAAACATGGCTGCAATAGTCGCAGGGAGTTTCCCAGCTATGAGTCCCTTCCTGCTCATGAGATGTCATAATGTTGCTCACAGCCCCTGCTTTTCTTGGATAAAGACTGAAAGAATGTCCATAGTCTTAAAACTAACCTGA

>Cricetulus_griseus_intact_V1R4_31

ATGGCTATTGAGAACTTGCCAATGGGGATTTTGTTCTTCTCTCAAACAACTGTTGGGATTCTTGGCAATTGGTCAGTTCTTCTTCCCTATGTCATGCCTGTACTCACTGGAAAAAATCTGATGCCCAAAGACCAGATTTTAAGGCATCTGTCTTTTGCCAATTCTTTGGTTATTATCTCAAGAGTAATTCCTCACATACTGGCACAGCTGGGCTTGCACAATCTCCTGGATGACCTTTTATGTAAACTGACTCTCTACAGTAACCGAGTGTCCAGGGGCATTTCCCTGCACTGCACCTGCCTCTTGAGTTGCTTCCAGGCAATCACAATCAGCCCCAGCAACTCCAGGTGCATGAAGCTTAAACACACAATCTCCAAGTACATGGTTCAGTCCTGCTCACTCAGCTGGCTTGTGCATCTGCTTCTAAACAGCAAAACAGCTATAGATGTGGTTGGATCTGGTACTAACAAAAACTTTACCATGAAAATCAAGTTGGGGTACTGCTCAGCGTTTCTTTATCACAATGCTGCAACTGTACTACATCTGATATTGATTCGTTTCACTGATGGGCTGTGTTTGGGTCTCATGGTCTGGACCAGTGTCTTCATGGTAGGCATCCTCTACAGGCACAGGAGTCAGCTACAGTACATCCACAGTGCCCAGCATTCCCTCAGAGTCTCCCCTGAAGACAGAGCCGCAAAAACCATCTTGATTCTTGTGTGCACCTTTGTCCTCTCCTACTCAATGTCTTTTATATTAGTTCTCTACACCACATTATTTAACAACCCAAAGCTGTGGGTAATCAGCATATTTACTTTCCTGGACACATGCTTCCCCACATTTTGCCCATTTATCCTCATCAGTAATAAGCAATCTGCTCCCAAGAGTCATTTTTCCTGCTGTAGGTGA

>Cricetulus_griseus_intact_V1R4_32

ATGCCCTCATTAAAAAATGTCCTTTATTTCCAAACTGGACTTGGAGTCTTCGCCAATATGTTTCTCCTTTTTTTCTACACTTTCATAATCCTAGTTCATAGATCTAAGCCCATGGACCTGATCTCCTGTCAACTGACCTTCATCCACATAGTTCTAGTCCTCACTGCAGGGGATATTGGGCTTTCAGAGGTATTTGAGTCACTGAACATTGAGAATGACTTCAAATGTAAGACAACTTTTTACATAAACAGAGTGATGAGAGGCCTCTCCATCTGCATCACCTGCCTCCTGAGTGTGTTCCAGGCTGTCACTATCAGTCCTAGTACATCTTTGCTGGCAAATTTTAAATATAAACTAAAAAAACATATGATCTATGCTTTCTTCTACCTTTGGACTTTCAATTTGTCATTCAGTAGTAGGTGGCTCTTCTATATTGGAGCTTTTACCAATGTGAGTGAGACCAACCAAATGAAGGTCACTAAATCCTGCTCACTCTTCCCCATGAACTCCATCATCAGAATACTGATTTTAACGGTGACAACCTCTAGAGATGTATTTCTTGTAGGAGTTATGCTGACCACAAGTGCATACATGGTGATTATTTTGTTCAGACATCAGAGGCAATGCAAATATCTTCACAGCCTCAGCCACCTGAGAGCGTCTCCTGAGAAAAAGGCCACTGTGACCATCTTGCTTCTGGTAGTTATCTTTGTGGTCATGTACTGGGTGGACTTCATCATCTCATGCACCGCAGTCCTGTTATGGATGTACCACCCAGTCATCCTGACTGTTCAGAAGTTTGTGATGAATGCCTATCCCACAATTACTCCTTTGGTACAAATCAGCTCTGATAACAGAATAATCAATATGCTGACAAACTTGTGGTCAAAGTGTCACAAGATTTTTTAA

>Cricetulus_griseus_intact_V1R4_33

ATGTATTTGTGGAATCTGACCATCAAAATAATTGTCTTGTCACTAACTACAATAGGAATTCTGGGAAATATCTCTGTGTTTTACTACTATTTAATCTGCTATGGAGACAGCAAATTAAAGACTGTAGATTTGATTCACGTGCACCTGATGGCAGCCAACACCCTGATCATTCTCTCTACAGGAGTGCCCCACACGATGGCAGCTTTTGGTTTGAAGCAGTTTTTAAATGATTTTGGATGCAGATTAATTTTGTACATTGTAAGAGTTGGCCGAAGTATGTCCATTGGCACCACCTGCCTCTTGAGCGTCTTTCAGGCCGTCACCATCAGTCAGAAGGAATCCTGTTGTAAGGATCAAAAAGTCAAAGCTGCTAAGTACATTGGCTGCACCCTTTCCTTTGTCTGGGTCTCATACATATTGATACATTTCATTTTCTTTGTGTATCCAATTATCAAAAGTTATAGTAACAATGTGATAAGTAAACGAGATTTTGGTCACTGCTCTACTGCAGGGCGGGATGGAATCAATGACTCACTCTATGCAGCATTGGTGGTGTGCCCTGAAATCTGCTTTTCGTTGCTCATGGCCTGGTCTAGTGGCTCCATGATCGTCATTCTGTACAGACACAAGCAGAGAGTTCAGCACATCCGGAGCACTTGTGTTTCCATCAGAAACTTCCCTGAATCCAGAGCCACCCATAACATTCTGGTCCTGGTGTCTACTTTTCTGGCTTTTTATACTCTCTCTGCTATCTTACAAGGCTGTGTGGCTCTTTTGTCTAATTCTAGTTGGTGGCTGGTAAACATCAGTCACCTCACTTCTCTGTGTTTTCCTTGTTTTGGACCCTTTATTCTTATGAATCATTACTCCATGCCAAGTCTCAGCTTGGTCTGGATAAGGAATGTGAACACCCTCATTCTTAAGTATGTAAATGATATAATTCTTATGGTATCCAGTTGTTTAGTCACCCATCTCCCTCACAAAGTAAGTACAGAAAGTTAA

>Cricetulus_griseus_intact_V1R4_34

ATGGACTTCAGGGGTGTGGCAATAGGAATAGTGTTCTTATTTCAGAGCACATTTGGAATTCTGGGGAATGTCTCTTTTCTTTCCTACTACCTCCTTCACTACTGCACTGCACAGAAGTTAATGACCAAGGATTTGATTCTCACACACCTGTTCACAGCCAATTCCTTAATCATTCTTTCTAAAGGAGTGCTAGAGATAACACATGCTTTTGAGATGAAATGGTTTACCAGTGATATTGGCTGCAAATTTCTTTTGTATATTCAAAGACTTGGCAGGAACATGTCCATTGGCACCATCTGTGTTTTGAGTGTCTTTCAGGCCATCACCATCAGTCCCAGTGTCTCTCGTTGGAAGGATCTGAAAATAAAAGCTGCAAAGTATACTGGACTCTCCATTTCCCTCTGCTGGGTTCTGTGCATGGGAATAAATATGATTTACCCTGTGCATACATATGCAATGGAAAGCAGCAAAAACATGACACTTAAACAAAATTTGATACATTGCTCCACTCCAGGATATAATGCAGTCAGAGGTTCATTGTATATGGCTTTCTGTGTAGTCCCTGAAATTTTGCTTTCTGTCATCATTGCCTGGATGGGTGGAGCCATGGTTATCACTTTGTACAGGCACAAGCAAAGAGTTCAACATATCCGAAGTACTCATGTTTCAAATATTACATCCCCTGTGTCCAGAGCCTCCCGGAGCATTGTTGTCCTGGTGTTCACTTTTCTAGGTTTTTATGCTCTCTCCTCCATCTTACAGGGTTGGAATGCTCTTGTATATAACAGTGGATGGTGGCTAATGAACATCACAGCTGTCATTTCTATGTGCTTTCCCACCTTAGGACCTTTTATTGTGAGTCATACCTCCACTTTATTCAAATTCCACTTTTTCTGCATGAGGAACCAAAATATCACATAA

>Cricetulus_griseus_intact_V1R4_35

ATGGACTTCCGGAGTCTGGCAATCAGAATGATTTTATTATCACAAACTACAGCTGGAATTCTGGGAAATTTCTCTCTAATTTTCTACTATCCAGTCCTTTGTTCCAGAGGATGTGTGCTCAAGCCCACAGATTTGATAGTGCTGAATTTAATGGCAGCCAATGCCTTGATCATTCTCTCTGCAGGAGTTCCACACACAATTGCAGCTTTTGGGGTGAAGCCGTTCTTGAACAATGTTGGCTGCAGACTCCTAATATGCATGCAGGCATTTGGCCGCAGTGTGTCCATTGGCACCATTTGTCTTTTGAGTGTCTTCCGGGCCATGAGCATCAGATTCAGAGAATCCTGTTGGAAGGACCATAAAGTCAGACCCGTAAATTACACTGCCTGCACCCTTTCCCTACTCTGGGTCTTCTACATGCTGATAAATTTTATTTTCTTTCTGTACCCACTTACCAAACTTAATAGTAAAAACACGACAAGAAAACGAGATTTTGGATACTGCTCAATTGTAGGGCGAAATGAAATCAGTGACTCCCTCTATGCAGCATTGGTGGTGTGCCCTGAAGTCCTGTTTTCTGTGCTCATTGCCTTGTCTAGTGGCTCCATGATTGTCACTTTGTACAGACACAAGCAGTCAGTTCAACACATCCGCAGCAAGCATGCTTCCAGCAGAACCTCCCCTGAGTCCAGAGCCACCCAGAATATCCTGGTCCTGGTGTCTAACTTTCTGGCTTTTTATACTCTCTCCTCAATTTTACAAGGCTGTATTGCTCTTTTGGATAATCATGACTGGTGGCTGACAAATATCACTCGCCTGACTTCTCTATGTTTTCCATCTTTTGGACCCTTTGTCCTCATGAATCATTACACTGTTGTGTCCAGGCTTTCTTTGATCTGGATCAGGAATAAAAAATAA

>Cricetulus_griseus_intact_V1R4_36

ATGACCCCTAATACTTTAGCAGGAACCCTATTTACAGTGCAAACTGTAGCTGGGATTTTAGCAAATTTCTCTCTTCTTCATCATTATGTGACCCTGTGTGTCACTGGGTTTAGGTTCAAGTCCACAGATGTCATAGTTCAGCACCTGACTGTAGCCAATGCCCTTATCATGCTTTCCAAAGGAATCCCACAGACCATGACAGCACTTGGAGTAAAATATTTCCTTCATGAGTTTGGATGTATCTCTGTTCTTTATATTTATAGAGTGGCCAGGGGTGTGTCAATTACTGCAACCTGCATACTCAGTGTCTTTCAGGCCATCAAGATGAGCTCCATGACCTCCAGCTGCAAGAAGCTTCCAACAAAAGCTCTGCAGGATGTTGGTGTCTCAATTTTCCTTTGTTGGATCCTATATATGTGTGTAAATTTAATTTTCCCACTCTACAGCCACAGCAGGTGGAAATTCAAAAACATCACACGGGATAGAGATCCTGCGTACTGTTCTGCCGGGCTTCAGAACAGAATCACAAATTCATTGTATATAGCTCTGGTTCTGTTCCCTGAAGTTTCATGCTCGGGACTCATGCTGTGGTCCAGTGGCACCATGGTGTGCATCCTGCACAGGCACAGGAGAAAGATGCAGTACATCCACAAGGCAAGGCTCTCCTCCAGACCCTCCCCCGAGGCCAGAGCCACCCAGAGCATCCTGGCCCTGGTGTGCACCTTCGTGTTTTTTTACACACTCTCATCCACCTTTTATGCTTACATTGCTGCCTTTAATATTTCCAGTAAGTGGCTAATGAACACCTCTGCCCTAATTTCTACCTGTTTTCCCACCATCAGCCCTTTGCTGTTAATGAACCGTGACGGCACTGTCACAAGGCTCTGGACCAAATTCAGAGAGTCTATCAGATTCATGTCACATTTGTCTTTCTTCACAAACTTCAGATTTCTACAAAGACCAGTGTGCAACTCAATAAGAAAGACAGAAGCTTCTTAA

>Cricetulus_griseus_intact_V1R4_37

ATGAAATCAGGAGATCTTGCCATTGGGATCTTCTTCCTGTCCCAGACTGCACTGGGGATTCTGGGGAACTTAGCATTGCTTTGCTGTTTTCTTATGTCTACCTTCAGTGTGAACAGGGTGAGGCCCACAGATGTCATTGTCAAACACCTGACCTGGGCCAACATTGTTGTTCTTCTCTGCAAAGGGATCCCTAAGACAATGGTTGCTTTTGATCAGACATATTCCTTAGATATCATTTCATGTAAACTTCTCTTCTATTTTCATAGAGTTGCCAGGGGAGTATCCATTGGTTGTACATCCCTGCTGAGTGTCTTTCAGGCCATAACCATCAGCCCCCACACTTCCAAGTGGGCACAGCTGAAAGTCAGACTCCACAGAATCATTGGTCCATCCCTGGGCCTGTGCTGGGCCTTTTACCTATTTTTAAATATCGTAATTATTGAGGGAGTGACTAATATGAGAAATAAAGGAAATCTTACAGAATTTAGAGATTTTTTATACTGTTTAATAATAAAACTTCCCAAACAAACTTCCATAGTACTTGCTATACTATTGGTTGCCAGTGATGTTATGTGTTTGGGACTCATGATATGGGCCAGTGGCTCCATGGTGTTGATCTTGTTCAAGCATAAACAGAGGGTCCAGCATATCCACAGATCCCTGTCCTCTAAATCATTTCCTGAAACCAAGGCCACACAAAGCATTCTTATCTTAGTGAGCAGCTTTGTGGTCCTTTATGTAACCTCTGCTACCTTAACCATGTATTTTCCTTTCCAGGATGGAGGAGGTACATGGGTGGCCAATGCCAATGTGGCCATGAGTGCCTCCTTCCCAGCAGTTTGCCCCTTTCTGCTAATAAGACACTATACGAGTAATTTTCATCCTTACAGTACTTGA

>Cricetulus_griseus_intact_V1R4_38

ATGTCCTCATTAAAGAATGTCCTTTATTTCCAAGCTGGACTTGGAGTCCTTGCCAATGTATTTCTCGTTTTTTTCTATACTTTCATAATTCTAGTTCATAAATCTAAGCTCATGGACCTGATCTCCTGTCAGCTGCCCTTCATCCACATAATGCTGGTCCTCACTGGAGGGGATATGTGGCTTACAGATATATTTCAGTCACTGAACAAGGCAAGTTTTTACATACACAGAATGATGAGAGGCCTCTCCATCTGCATCACCTGTCTCCTGAGTGTGTTTCAGGCTGTCACTGTAAGTCCCAGTACCTCTTTGCTGGCAAAATTTAAATATAAACTAAAAAAATACATGATCTATGCTTTCTTCTATATGTGGTTTCTCAACTTGTCATTCAGTACCAACCGGATCTTCTATGTTGGAGCTTTTACCAATGTCAGTGAGACCAACCAGATGAGGATCACCAAATATTGCTCACTCTTCCCCATGAACTACATCATCAGGGTGCTGATTTTAACAGCAACAACCTCCAGGGATGTATTTCTTGTAGGAGTTATGCTGACCACAAGTGCATACATGGTGATTACCTTGTTTAGACATCAGAGGCAGTGCAAATATCTTCACAACCCTGTCAGCCACCTGAGAGCATCCCCTGAGAAAAGGGCCACCCAGACCATCTTGCTGCTTGTGGTTTTATTTGTGGTCACATACTGGGTGGACTTCATCATCTCATCTACCTCAGGTCTATTATGGATGTACCACCCAGTCATCCTGACTGTTCAGAAGTTTGTGGTGAATGCCTATCCCACAATTACTCCTTTGGTCCAAATCAGTTCTGATAACAGAATAATCAATATGCTGAAAAATTTACGGTCAAAGTGCCACCAGATTTTTTTAAATGTGTATTTTTTTCTTGTTTAA

>Cricetulus_griseus_intact_V1R4_39

ATGTCCCCATTAAGGAATGTCCTTTATTTCCAAGCTGTTCTTGGACTCCTCGCCAATACGTTTCTTCTTTTTTTCTACACTTTCATAATCCTAGTGCATAAATCTAAGCTCATAGACCTGACCTCCTGTCAACTGACCTTTGTCCACATAGTGCTGCTCCTCGTTGGGGGGGAAATCGGGCTTACAGATATATTTGAGTCACTGAACAGTGAGAATGACTTCAAATGTAAGGCAACTTTTTACATACACAGAGTGATGAGAGGCCTCTCCATCTGCATCACCTGCCTCCTGAGTGTGTTCCAGGCTGTCACTATCAGTCCCAGTACCTCTTCACTGGCAAAATTTAAACATAAACTAAAAAAATACATGGTCTATTCTTTCTTATTTATTTGGGCTCTCAATTTGTCATTCAGTAGCATCATTGTCTTCTACGTTGGTGCTTTTACCAATGTGAGTGAGACCAACCAGATGAAGTCCACCAAATATTGCTCACTCTTCCCCATGGACTACATCATCAAGGCACTGATTTTAACAGAGACAATCATCAGAGACGTATTTCTTGTAGGAGTTATGATGACCTCAAGTGCATACATGGTGATTATCTTGTTCAGACATCAGAGGCAATGCAAGCATCTTCATAGCCTCAGTCACCTGAGAGCGTCCCCAGAGAAAAAGGCCACACTGACCATCTTGCTTCTGGTGGTTGTCTTTGTGGTCATGTACTGGGAAGACGTCATCATCTCATCCACCGCATTCCTGTCATGGATGTACCACCCAGTCATCCTGAGTGTTCAGAAGTTTGTGATGAATGCCTATCCCACAATTGCACCTTTGGTACAAATCAGTTCTGATTACAGAATAATCAATATGCTGACAACCTTGCAGTCAACGTGTCACCAGATTTTTAAATAA

>Cricetulus_griseus_intact_V1R4_4

ATGTTTTCAAGAGACCTAATTTCTGAATTCTTCTTCCTGTCAGAACTTTTCATTGGATTCCTGGGAAACTCACTGCTCTTCATGCTATACATGTACAGCTTCTTAATTCAGCCTCGTCTGAAGAAACCCATAGATATGATTTTCACACATCTGACGCTTGTCAACGTGCTGAGCATCGCCTTCCGGCTGCTGCCAGACGTCATGGCGTCCTTCGCGGTCAAGCTCCTTTTCCATGACGTTGGATGCAAGGCAGTTTTGTACGCATACAGTGTTACCAGGGGCCTTTCCATCTGCACTACGTCTCTGCTGAGTGTGTTTCAAGCCGTCACTGTCAGTTCTAATCATTCCAAGTGGGCGTGGCTTAAGTCCAAGCTTGAGCCCTGCATTTTTCCCTCACTCCTTCTCATCTGGATCATCAATGCCTTTCTCTATATTCCCATGCTTGAAAACGTAAAGGGCCAAATCAACTTCACTGTTGTGGATTCTAGATATTCCCAGACATACTGCCGAAGCAACCAGGTTCGCCATCACACCACCTTGTCACTTGTGACTGCGTTAACGATTAGAGACATCCTGTTCGTGCTTCTCATGATGGGGACCAGCCTCTACATGGTGACACTCCTGTTCAGACACAATAGGAGAACCCGGCATGTCCACAGTTCCAGAGTCTCTTCCCAGGCCTCTTCTGAAAAGAAAGCCACGCACAGCATCCTCCTGCTGGTGGGTTTCTTCATGTTTTTCTATTTCTCAAACACCTTTGTCACCTTCTATTCACTCCACGGACCTAAGAACAGCCAAGTATTGGATGTGATTAGTGGAGCTTTGTCTTCAGGCTACCCAATCATCTGCCCTTATGTTCTGATGAACAATAGGAAAATCATTTCCACATTCATTTCTTCCCTTTCAAACTTTGAATGTACCTTTTCTACAAGAGGCTGCCATGGCTAA

>Cricetulus_griseus_intact_V1R4_40

ATGTACTTCTGGACTCTGACCATGAAAATCATTTTCTTGTCACAAACTACAACTGGAATTGTGGGAAATTTCTCTCTTCTTTACTACTACATGGTCCATTATGGAAAAGGCACAATAAAGGCCACAGAGTTAATTCTCATGCATCTAATGGCAGCCAATGCCTTGACCATTCTCTCTGCAGGAGTGCCCCACACAATAGCAGCTTTTGGTTTGAAGCACTTTTTTAATGACTTTGGATGCAGATTAATTTTGTACATTCAAAGAGTTGGTCGTAGTGTGTCCATTGGCACCACCTGCCTCTTGAGTGTCTTTCAGGCCATGACCATCAGTCACACACAATTCTGTTGTAAGGATCTGAAAGTCAAAGCTTCAAAGTACATTGGCTTCTCTGTTGCCCTCCTCTGGATCTTTTACACGTTGATAAATTTCATTTACTTTGTGTACCTGTTTCTCAAGAGGAATGTTAAAAATACGACAATAAAACATGATTTTGGATACTGCTCCACTGCAGGGCGCGATGAAATCAGTGACTCATTCTATGCAGCGTTGGGGGTGTTCCCTGAAATCTGCTTTTCTTTGCTTATGGCCTGGTCCAGTGGCTCCATGATTGTTATTCTATACACACACAAGAAGAGGGTTCAACATATCCTTAGCACCCATGGTTCCAGAAGAACCTCCCCTGAGTCCAGAGCCATCCAGAACATCCTGGTCCTGGTATTTATCTTTCTGGCTTTTTACACTCTCTCCTCTATCTTACAAGGCTGCATAGCTCTTTTGTATAATCACAGCTGGTGGCTGGTGAACATCACTCGCCTTACTGCTTTGTGTTTTCCCTCTTTTGCACCCTTGGTTCTTATGAATCATTACTGCAGACTACCTAGACTCAGTTTTGTCAGAATACGGAATATAAATCACTGA

>Cricetulus_griseus_intact_V1R4_41

ATGGCCACCAGAGACTTTGCACTGGGGATCTTCTTCCTATCCCAGACTGTGCTGGGGATTTTGGGAAACTTAGCCTTGCTTTGTTGTTTTATCGTTGCTGACTTCTCTGGAATTAGGGCGAAGCCCACAGACCTGATTGTCAAACACCTGACCTGGGCCAACTTCATAGTTCTCTGCAGAGGAATCCCGCAGACCACTGCTGCTTTCAGTCAGAATTACCATCTAGATTATGTTTCATGTAAACTTGCCTTGTATTTTCATAGAGTTGGCAGAGGAGTGTCCCTTGGCTCCACATCCCTGCTGAGTGTCTTTCAGGCCATCACCATCAGCCCCAGTAATTCCAGATTGGCACAGCTCAAGGTCAGAGCCCCGAGGATTATTGCTCCATCCCTGGGTGTGTGCTGGGCCCTCCAGCTTTTGGTGTATATCTTCATTCCTTTATATACAACTGACATTTGGGGTGGAAGAAATGTTACTGGGATAAAAGACTTTGGATACTGTGTTATTATCAGCGACGGAGGACTAAGCATTACACTTATTGTCATCCTATTGTTATCCAATGATGTCATGTTTTTGGGACTGATGATGTGGGCCAGTTGCTATATGGTGTTTATCCTGCTCAAACACAAGCAGAGGGTCCAACACATCCACAAATCCCAGTTTCCTAGGGCATCCCCTGAGACCAGAGCAACCCAAAGCATCCTCATTCTAGTGAGCAGCTTTGCGTTCTTCTATGTAACTTCTATTGTCTTTACGTCTTACCTGGCTTTGCTTGAGGCAACCAATAGGTGGCTGTCCAATGCGTGTGTTGCTATGACTGCTTGCTTCCCAGCCTTCTGTCCCTTTCTGCTCATCAGACACTATGCTTCCCTTCTCAGGCTCTGCTGTACCTCTTATCCTCAGACAACACTCTGA

>Cricetulus_griseus_intact_V1R4_42

ATGGACCTCCAGGATTTGGCAGTAGGAACAGTGTTCTTGCTTCAGAGCACAGTTGGAATTGTGGTAAATTTATCTCTCCTTTCCTGCTACTTCATCCGTTACTATGTTGAACAGACATTAAAGACCACAGACTTGATTCTGACACACCTGCTCATGGCCAATTCCTTGATTATTCTTTCTAAAGGAATTCTAGAGACAGTAAGGGCTTTGGGGATGAAAGGGTTCTTCAGCGATTTTGGCTGCAAACTTCTCTTGTATATTCAAAGACTTGGCAGGAGCATGTCCATTGGCACCACCTGTCTCTTGAGTGTTTTCCAAGCCATCACCATCAGCCCGAGTGACTCCTGTTGGAACTGTCTTAAATTCAAGGCTCCAAAGCACATTGGCCTCTTCACTTCCCTCTGCTGGATGCTCTACATGTCAGTAAATATGATTTTCCCTGTGTATATGTCCTCCAAGGGGCACAGCAATAACCTCACATGTAAGAGTGATATGAAATACTGCTCCACTGCAGGTCATGATGGCCTCACAAGCTCATTATACATAGCGTTTTTTGTTCTCCCTGAAACTTTGCTTTCTGTCCTCATTATCTGGTCCAGCAGCTCCATGGTTGTCATTCTGTACAGGCACAAGCAACAGGTTCAACACATCCGGAGCACCAGTGTTTCCTCCAGAACGTCCCCTGAGTCCAGAGCCACCCAGAGCATCCTGGTCCTGGTGCTCACTTTTCTGGGTTTTTATGCCCTCTCCTCCATCTTACAAGGTTGTGTTGCTCTCATACATAATCCTGGCTGGTGGCTGATGAACATCACGGGCATCATTTCTATGTGTTTTCCTACTGTGAGCCCCTTTGTTGTGAGCCATGATGCCACTGTGCCAAGACTGTGCTATTCCTGA

>Cricetulus_griseus_intact_V1R4_43

ATGTATTTCTGGACTCTAATCATCAAAATAATTTTCTTATCACTAACTACAATAGGAATTCTTGGAAATTTCTCTGTGTTTCACTACTATCTGGTCTGCTATGCAGAGTGCAAATTAAAGACTGCAGATATGATTCATGTGCACCTGATGGCAGATAAAGGAGTGCCCAACACCATGGCAGCTTTTGGTTTGAAGCAGTTTTTAAATGATATTCAATGCAGATTACTTTTGTACATTGAAAGAATTGGCCGCAGTGTGTCCATTGGCTCCACCTGCCTCTTGAGTGTCTTCCAGGCTGTCACCATCAGTCAGAAGGAATACTGTTGTAAGGATCAAAAATTCAAAGCTGCTAAGTACATTGGCTGCTCCATTTCCCTTCTCTGGGTCTTGTACATATTGATACATTTCATTTTCTTTGTGAATCCACTCATCAAAAGGTATAGTAATAATGTGACAGGAAAACAAGATTTTGGACACTGCTCAATTGCAGGTCGGGACGGAATCAGTGACTCACTCTATGCAGCATTGGTGGTGTGCCCTGAAATCTGTTTGTCTTTGTTCATGGCCAGGTCTAGCGGCTCTATGATTGTCATTCTGTACAGACACAAGCAGAGGGTTCAGCACATCCGTAGCACCCATGGTTCCAGCAGGACCTCCCCTGAGTCCAAAGCCACCCAAAACATTCTGGCGCTGGTGTCTACCTTTCTGGCTTTTTATACTGTCTCTACTATCTTACAAGGCTGTGTGGCTCTTTTGTATAAGCCTAGTTGGTGGCTGGTGAACATCAATCGCGTCACTTCTCTGTGTTTTCCTTGTTTTGGACACTTTATTCTTATTAATCATTACTCCATGGTTTCAAGACTCAGTTTGGTCTGGATGAGGAATATAAACTCAGTTATTCTTAGATAA

>Cricetulus_griseus_intact_V1R4_44

ATGGCAGCCAGTGAGTTATCTTTAGGAGTGGTCTTCTTGTCTCTGACTATGATTGGACTACTGGGGAATTTCTTGCTTCTCTGTCACTACATGTTTCTTTACCTAATGAGGTACAGGATAAAGTCTACAGACTGGGTTCTGATTCACTTGGTTGTAGCCAGCATCTTAACTGTCCTGGGAAAAGGAGTGCCACACTCCATGGCAGCTTTTGGACTGAAGGACTTCCTCGATGATAATGGATGCAAATTGGTTTTCTCCTTTCACAGATTAGGTCGAGGTTTGTGTATTGGCTGCACCTCCTTCCTTAGTGTCTTCCAGGCCATCATCATCAGCCCCAGGGACTCCAGGTATTCAGAACTGAAAATAAAAGCACACAAGCACGGCTGTTATGCTCTGTACCTGAACTGGGCAATTCATTTCCTTATAAGCAGTATAAATCTTGTGCACATGAGGGCAAGATACGGAAATGCAAGCACAGCAAACCTGAAATCATTTTTATACTGCTATTCTGTCCGTCATGACCAAGCCAGTGACATTTTTTATGCAGCATTGCTGTCAGCTCCTGACATTTTTTTTCTGGGGCTCATGCTATGGGCCAGTGTCTTCATGATTCTCATCCTGTACAGGCACAAGCAAAGGATGCAACACATGCCCAAGATTAATACCTCCTCCAGATCCTCCCCCGAGTCCAGAGCCACTAAAACTATCCTTCTCCTGGTCAGTACCTTTGTCTCCTTTTACACAATCTCTTCCCTAGGTCAACTCATTGGTGCTATTGTGGATAATCCAAGCTGGTCAGTGGTGCATTTGACTGCAATGGCCTCTCTCTTTTTCCCCACTGTTTGTCCTTTTCTCCTCATGAGCCGTGATTCTCGGGTGTCCAGCTGCTGCCTGACCTTGAAAATAACTAGACATTTCCCTAAAGCATCTAATCAAGAGCAGAATTGA

>Cricetulus_griseus_intact_V1R4_45

ATGTATTTCTGGACTCTGACCATCAAAATAATTTTCTTATCAGTAACTACAATAGGAATTCTTGGAAATTTCTCTGTGTTTCACTACTATCTGGTCTGCAATGCAGACTGCAAATTAAAGACTGTAGATTTGATTCACATGCACCTGATGGCATCCAACACCCTGATCATTCTCTCCAAAGGAGTGCCCAACACCATGGCAGCTTTTGGGTTGAAACAATTTTTAAATGATTTTGGATGCAGATTAATTTTATACATTGAAAGAGTTGGCCGCAGTGTGTCCATTGGCTCCACCTGCCTCTTGAGTGTCTTCCAGGTGGTCACCATCAGTCATAAGGAATCCTGTTGTAAAGATCAAAAATTCAAATCTGCTAAGTGCATTGGTTGCACCCTTTCCCTTCTCTGGGTCTTGTACATAGTGATACATTTCATTTTATTTGTGAATCCACTTATCAAAAGTTATAGTAACAATGTGACAGGAAAACAAACTTTTGGACACTGCTCTATTTCAGGGCGGGATGGAAACAGTGACTCACTCTATGCAGCATTGGTGGTGTGCCCTGAAATCTGCTTTTCTTTGCTCATGGCCTGGTCTAGTGGCTCCATGATTGTCATTCTGTACAGACACAAGCAGAGGGTTCAGCACATCCGCAGCACCCATGGTTTCAGCAGAATCTCCCCTGAGTCCAAAGCCACCCAGAACATCCTGGTGCTGGTGTCTACCTTTCTGGCTTTTTATATGGTCTCCACTTTCTTCCAAGGCTGTGTGGCTCTTTTGTATAATCCCAGTTGGTGGCTGGTGAACATCACTAACCTATGTTCTCTGTGTTTTCCTTGTTTTGGACCCTTTGTTCTTATTAATCATTACTCCATGGTTTCAAGATTCAGTTGTGTCTGGATGAGAATATAA

>Cricetulus_griseus_intact_V1R4_46

ATGACATGGAATAACATTATCCAGACAATAATCTTCCTTTGTCTTATTGGACCTGGAATTGTAGGGAATATCCTAATGTTTGGGAGACATGTATACAATTCTGTCTTGGGGACTGAGAAAAAGCCTGTGGACCTTATCCTCATGCACTTGGGACTTTCTAATATGATCATTATTTGTACAACAGGGATCAGAGATATCGGTACAGTGTTTTATTTCAGAAACTTCCTTGGAGATTTTGGCTGTAAAGCTGTGGTTTTTCTGGCAAGGATGGCTCGGGGCCTTTCCATCTGTACCACCTGTCTTCTCAGTGTCGTCCAGGCTGTCACCATCTGTCCCAGGGCCACCATTTGGAGCAAGCTCAAACCACAGTCCTCACAGCAAGTTCTTACCTATGTCTTCCTCTTTTGGATCTTTAATGTTCTCACAAGTTCCAACTTGTTGTACTATATAACAGCAGGCAGTGACAACAACAGATCTAAAGTTCCAGAGTATATTGGGTATTGCTATATGCTTCCATCCAGGCTCGAAGTTAAGTGGCTTTTCCTCTCGCTCATGGCTGTTCGTGATCTGATCTTTCAGAGTCTCATGGGCTGGAGCAGTGGATACATTTCTTTTTACCTGTATAGACATCACAAGCAAGTCCTCTACCTTCATAGCTGCAGGTTGGCAAATAGTTTCAGTGCAGAGATCAGAGCTGCACAGAGTGTTCTCCTTCTCATGGCCTGTTTCCTTTTCTTCTATTGGACAGACTTTGTTTTCTCCTTTTACACAGGTTCCATGGTGACTCATGACTCCGTTATACTAAATATTAAAAAACGTCTAGTACTTGGTTATGCTGTTCTCAGTCCCTTTGTCCTGAGGAGCAGAGATGTGCCTCTTGCTAAATCCTTGTGTTTTCCCTGA

>Cricetulus_griseus_intact_V1R4_47

ATGGACTCCAGAAACTTGACTATAGGAATAGTGATCTCACTTCAGAGTACATTAGGAATTCTGGGAAATTTATCTTTTCTTTTTCACTATCTACTCATTTACTACACTGAACACACATTAAAGACTGTAGACTTAATTCTTATACATGTGTTCACAGCAAATTCCTTAATCATTCTCTCTAGAGGAGTACCCCAGTTAATGAGAGCTTTTAAGTGGAAAAGGTTCTTCAATGATGTTGAATGCAAACTCATTTTCTACGTTCACAGACTTGGCAGGAGCATGTCCATCACATCCATCTTCCTCTTGAGTGTCTTCCAGGTCATCACCATCAGCCCTAATGTCTCCTATTGTAAAGACCTTAAAACCAAATTAACAAAATATGTTCACCTTTCCATTTCCTTCCTCTGGATCCTGTACATGATAGTAAATATGGTTTTTCCCATGTACACATCTATGAAGAGTAATGTCGAAAATAAGACAAAAATGAGAGATTTTGAAATTTGTGACTCTCTCAGTCGAAACAAAATAGTAGATTCACTGTTCACAGCATTTTGGGTATTTCCTGAAGTCTTATTTTCTACACTCATTGTATGTTCCAGCGTCTCCATGATTGTCATACTCTATGGACACAAGAAGAGGGTTCAGTACATCCTCAGCACTCATTCGTCCACCAGAATTTCCCCTGAATCCAGAGCCACACAGAACATCCTGGCCTTGGTTTGCACCTTTCTAGCTTTCTATTCCATCTCCTCCTTTTTACAAGGCTACATCGCTCTTTCACGTAATTCCAATTCGTGGCTAAGGAATATCACAACCATGGTTTCTACTTGTTTTCCTACTTTATGTCCCTTTGTGGTGAGACATGATTCCATTATTTCCAGAGTTTACTTTTTCTGTTTGAGAAATATCAAAAGGAATTAA

>Cricetulus_griseus_intact_V1R4_48

ATGTTCTCCTTAAAGAATGTCCTTTATTTCCAAGCTGGACTTGGAGTCCTGGCCAATATTTTTCTCCTTTTTTTCTGCACTTTCATAATCCTAGTGCATAGATCTAAGCCCATGGACCTGATCTCCTGTCAACTGCCCTTCATCCACATAGTGCTGGTCCTCACTGGAGGGGATATTGGGCTTACAGATATATTTGAGTCACTGAACTTTGACAATGACTTCAAATGTAAGACAAGTTTTTACATAAACAGAATGATGAGAGGCCTCTCCATCTCCATCACCTGCCTTCTGAGTGTGTTCCAGGCTGTCACTATTAGTCCTAGTACCTCTTTGCTGGCAAAATTTAAATATAAACTAAAAAAATACATGATCTATGCTTTCTTCTATATGTGGTTTCTCAACTTGTCATTCAGTAGCAACCAGATCTTCTATGTTGGGGCTTTTACCAATGTGAGTGAGACCAACCAGATGAGGATCACCAAACATTGCTCACTCTTCCCCATGAACTACATCATCAGGGTGCTGATTTTAATAGCAACAACCTCCAGGGATGTATTTCTTGTAGGAGTTATGCTGACCTCAAGTGCATACATGGTCATTATCTTGTTCAGACATCAGAGGCAATGCAAATATCTTCACAGCCTCAGCCACCCAAGACTGTCCCCTGAAGAAAAGGCCACCTTGACCATCTTGCTATTGGTAGTTGTCTTTGTGGTCATGTACTGGGTGGACTTCATCATCTCATCCATTGCAGTCCTGTTATGGATGTACCACCCAGTCATCCTGACTGTTCAGAAGTTTGTGATAAATGCCTATCCCACAATTACTCCTTTGGTACAAATCAGTTCTGATAACAGAATAATCAATATGCTGACAAATTTGCAGTCAAAGTGTCACAAGATTTTTTTAAAAGGGTAA

>Cricetulus_griseus_intact_V1R4_49

ATGTTCCTCTCTGTTATGAAGATAAATAAGATATACACTCATGTCATCGTTAAGAACATTTGTTATTTCCAAATTGCCATTGGAATTTCAGCCAGCACCTTTCTTCTTTTGTTGCATGTCTTCACAGTCCTTCAAGAGTGCAGAATAAAGCCCACCGAGCTGATCACATGTCACTTGGCCCTCGTCCATATTATAATGCTACTTGTTGCATTAGATTTTTTGTCTCCAGACATGTTTGAGTCACTGAATTTATTCAATGATTTTAAATGCAAGACATTGTTTTACCTGAGCAGGGTGATGAGGGGCCTCTCGATCTGTATCACCTGCTTCCTGAGTGTGCTTCAGGCCACCACCATCAGCCCATACTCGGACTGGATGGTTAAAATCAAACACATACTTACAAATTACACCATCCATATTTTCTTGTTTTTCTGGTCCCTCAATTTGTCTCTCAGTAGTAATATCATCTTGCTCACTGTAGCTCATTCCAACACAAGTCAGACAAATCTGCTTATTGTCAGTAAATACTGCTCAATATCTCCAACTACCTCCATCATGAGGGGACTGTATTTCACTTTGACATTTTCCAGGGATGTCTTCTTTGTGGGACTCATGCTGTTCTCTAGTGCATACATGGTGCTTCTGTTGTTCTGGCATCATAGGCGATCACTGCATCTCCACAGAAGCAGCCTCTCGGCAAGACACTCCCCAGAGCAAAGAGCCACCTGGACTGTCCTGGTTCTGGTGAGTGTCTTTGTGGTCACATACTGGATGAACCTCATCATTTCATCCTGTTCAACATTGATCTGGAGGTATGACCCAGTCCTTCTGAGTCTTCAGAAACTTGTACTCAATGCCTATGCTACTGCATGTCCAGTGATACAAATGACTTTCCAGAAGAGAACACATGATATTGTGCGACTCATGTACTGGAAATGTTGTCAGTTTATTACAAGATAA

>Cricetulus_griseus_intact_V1R4_5

ATGTTTGCAATAAACTACATACTTTATTTCCAAGCTGGACTTGGAGTCCTGGCCAATATGTTTCTCCTTTTTTTCTACACTTTCATAATCCTAGTTCATAGATCTAAGCCCCTGGACCTGATATCCTGTCAACTGCCCTTCATCCACACAGTACTGGTTCTCACTGGAGGGGATACTTGGCTTACAGATGTATTTGAGTCACTGAACATTGAGAATGACTTCAAATGTAAGACAACTTTTTACATGAACAGAGTGATGAGAGGCCTTTCCATCTGCACCACCTCCCTCCTGAGTGTGTTCCAGGCTGTCACTATCAGTCCCAGTACCTCTTTGCTGGCAAAATATAAATATAAACTAAAAAAGTACATGATCTATGCTTTTTTCTATATGTGGTTTTTCAACTTGTCATTCAGTAGCAACCAGATTTTCTATGTTGGGGCTTTTACCAATGTCAGTGAGACCAACCAGATGAAGGTCACCAAATATTGCTCACTCTTCCCCATTAACTACATCATCAGGGTGCTGATTTTAACTGCAACAACCTCTAGAGATGTGTTTCTTGTAGGAGTTATGCTGACCTCAAGTTCATACATGGTGATTATCTTGTTCAGACATCAGAGGCAATGCAAATATCTTCACAGCCTCAGCCACTTGAAAGCATCTCCTGAGAAAAGGGCTACCCAGACCATCTTGCTTCTGGTAGTTGTCTTTGTGGTCATGTACTGGGTGGACATCATCATCTCATCCACTGCAGTCCTGTTATGGACGTACCACCCAGTCATCCTGACTGTTCAGAAGTTTGTGATGAATGTCTATCCCACAATTACTCCTTTGGTACAAATCAGTTCTGATAACAGAATAATCAATATGCTGATAAACTTGTGCTCAAACTGCCACCAGGATTTTTTAAAAGTGTAA

>Cricetulus_griseus_intact_V1R4_50

ATGGACCTCAAGGATTTTACAATAGGAATTGTGTTACTGCTTCAGAGCACAGTTGGAATTGTGGGAAATGTCTCTCTTCTTTACTGCTACCTAATCCATTACTACACTGAACAGACATTAAAGAACACAGATTTGATTCTTGCACACATGTTCACAGCAAACATCTTGATCATTCTTTCTAAAGGATTGCTCCATTCAACTAGAGCTTTTGGGATGAAAGGTTCCATCAATCATTTTGGCTGCGAATTTCTTTTGTATATTCAAAGACTAGGCAGAAGCATGTCCATTGTCACTGCGTGCCTCTTGAGTGTTTTCCAGGCTATCACAATCAGCCCTGGGAACAGTTTTTGTTTGCGCCTTAAAGTCAAAGCTCCAAAACATATTGTTCTATTCACCTCCCTCTCATGGATAATTTACATGTCAGGAAATATGATTTTTCCTGTTTATAAGTATGCTAAGGGGAACAGCAATAGACTGATACATGAGAATGATATGAAATATTGCTCAACTGTAGGTCATGATGATGTTACAAGCTCATTATATACAGTGGTTTTTGTTCTGCCTGAAATTTTGCTTTCTGTCATCATTGTTTGGTCTAGTTGCTCCATGGTTATCACTTTGTATAGGCACAACCAACGGGTTCAACATATCCACAGCTCCAGTGTTTCCTTCAGAACATCTCCAGAGAACAGAGCCACCTACCGCATACTGGCCTTTGTGTTAACCTTTATAGGTTTTCATTCCCTCTCTTCCATCTTGCAAGGTTGCAATGCTCTCACACAAAACCCTCATTGGTTGCTATTGAACATTACTGCAATCATTTCAATATCTTTTCCTACTTTGGGACCCTACTTCATGAGCCATGACTCCACATTTCAAAGGTTGTGCTTTCCATGA

>Cricetulus_griseus_intact_V1R4_51

ATGTCCCCATTAAGGAATGTCCTTTATTTCCAAGCTGTTCTTGGACTCCTCGCCAATATGTTTCTTCTTTTTTTCTACACTTTCATAATCCTATTTCATAAATCTAAGCTCATAGACCTGATCTCCTGTCAACTGACCTTTGTCCACATAGTGCTGCTCCTCGTTGGGGGGGAAATCGGGCTTACAGATATATTTGGGTCACTGAACATTGAGAATGACTTCAAATGTAAGGCAACTTTTTACATACACAGAGTGATGAGAGGACTCTCTATCTGCATCACCTGCCTCCTGAGTGTGATCCAGGCTGTCACTATCAGTCCCAGTACCTCTTCGCTGGCAAAATTTAAACATAAACTAAAAAAATACATGATCTCTGCTTTCTTATTTATTTGGACTCTCAATTTGTCATTCGGTAGCACCATTGTCTTCTACGTTGGGGCTCTTACCAATGTTAGTGAGACCAATCAGATGAAGTTCACCAAACATTGCTCACTCTTCCCCATGGACTACATCTCCACAGCACTGATTTTAACAGAGACAGTCATCAGAGACGTATTTCTTGTAGGAGTTATGCTGACCTCAAGTGCATACATGGTGATTTTCTTGTTCAGACATCACAGGCAATGCAAGCATCTTCATAGCCTCAGTCACCTGAGAGCGTCCCCAGAGAAAAAGGCCACACAAACCATCTTGCTTCTGGTGGCGTTCTATGTGGTCATGTACTGGGAAGATGTCATCATCTCATCCACTGCATTCCTGTCATGGATGTACCACCCAGTCATCCTGAGTGTTCAGAAGTTTGTGATGAATTCCTACCCCTCAATTGCTCCTTTGGTACAAATCAGTTCTGATTGCAGAATAATCAATATGCTGACAACCTTGAGGTCAAAGTGTCACCAGATTTTTAAATAA

>Cricetulus_griseus_intact_V1R4_52

ATGAAAATGGCTTCTGACAACTTGGCGGTGGGAATTTTCCTCTTCTCCGAGATTGCCGTGGGCATGCTTGGAAATTCCTTAATATTATTTTATCATATAATTTTGATAGTCACTGGAAAACATTTAATGAAAAAAGACCTGATCATAGAGCACTTGACTTTTGCCAACTGTTTGTCTATCATTTCAAGAGGAATTCCACAGACAATGTCAGATTTTGGATTTAAGTATTTCCTAGATGACACTGGATGTAAATTGATAATGTACATTTACCGACTATCAAGGGGGGTGTCCCTGTATGCCATGTGCCTACTAAGTTGCTTCCAAGCTATCACAATCAGCCCCAGTAACTCCAGGTGGATGAAGCTTAATCTCAGAGCCAACAAGTTCATTGGTCCCTCCTGCTTACTCAGCTGGCTTGTGAGCCTCCTTCTAAACATCTCGACTCCAGCAAGAGTGTCAGGCCCCATTTACAATAAAAATGCAACTAGTAGGATGAGTTATGGATACTGTTCATGGTTTGCTTCTGGTAATGTGGTAACGGCATTGTATATGTTCTTACTGTGCTTCACGGATGGTCTGTGTCTGGGTCTCATGGCCTGCTCAAGTGTCTCCATGGTGACAATCCTCTACAGACACAAGAGACAAGTCAAGCATATCCATAGTGCTCAACACTGTCTAAAAGTTTCACCTGAGGACAGAGCTGCCCAAACTATCCTCACCCTGGTGTGCATATTTGTCATCTCTTATTCATTCTCTTCCATTTTGGTCATCTTTACTACCTACTCCAAATTTCCAATGCTATGGGCAGTAAGTATATCTCTATCTCTAGAAATATGCTTTCCCATAGTTAGCCCCTTTGTTCTCATCAGCAATATTCGGGATAGTTCCAGCATATTTTTACCCTGCTGTCATAAGAGAAAGCTTCTTTCGAGTTTGACATGA

>Cricetulus_griseus_intact_V1R4_53

ATGTTCTCCTTAAAGAATGTCCTTTATTTCCAAGCTGGACTTGGAGTCCTGGCCAATATTTTTCTCCTTTTTTTCTGCACTTTCATAATCCTAGTGCATAGATCTAAGCCCATGGACCTGATCTCCTGTCAACTGCCCTTCATCCACATAGTGCTGGTCCTCACTGGAGGGGATATTGGGCTTACAGATATATTTGAGTCACTGAACTTTGACAATGACTTCAAATGTAAGGCAAGTTTTTACATACACAGAATGATGAGAGGCCTCTCCATCTGCATCACCTGTCTCCTGAGTGTGTTTCAGGCTGTCACTGTAAGTCCCAGTACCTCTTTGCTGGCAAAATTTAAATATAAACTAAAAAAATACATGATCTATGCTTTCTTCTATATGTGGTTTCTCAACTTGTCATTCAGTACCAACCGGATCTTCTATGTTGGAGCTTTTACCAATGTCAGTGAGACCAACCAGATGAGGATCACCAAATATTGCTCACTCTTCCCCATGAACTACATCATCAGGGTGCTGATTTTAACAGCAACAACCTCCAGGGATGTATTTCTTGTAGGAGTTATGCTGACCTCAAGTGCATACATGGTGATTATCTTGTTCAGACATCAGAGGCAATGCAAATATCTTCACAGCCTCAGCCACTTGAAAGCATCTCCTGAGAAAAGGGCCACCTTGACCATCTTGCTTCTGGTGGTTGTCTTTGTGGTCATGTACTGGGTGGACTTCATCATCTCATCCACTGCAGTCCTGTTATGGATGTACCACCCAGTCATCCTGACTGTTCAGAAGTTTGTGGTAAATGCCTATCCCACAATTACTCCTTTGGTACAAATTAGTTCTGATAACAGAATAATCAATATTCTGACAAACTTGCAGTCAAGGTTCCACAAGAATTTTAGAAAATGGTAA

>Cricetulus_griseus_intact_V1R4_54

ATGCAAGGTCCTAAGACTCAAACACCAGACCCAGACAGAAGGGCAGCCAGTGATGTGACCATGGGAATGATCTTCTTATCACAGACTGTGGTTGGAGTTTTGGGCAATTCCTGTCTCCTCTACTACTATGTGTTTCTTTACTTTACCCAAAGCAGATTAAGGTCCACAGACTGGGTTCTGATGCATTTGATTGTAGCAAATTTCTTAACTCTCTTATGTAAAGGAGTGCCCCAGACAATGGCAGGTTTTGGTTGGAAAGACTTCCTCGATGATTCTGGATGCAGAGGACTTTTCTATCTCCATAAAGTGGGCAGAAATGTGTCCTTTAGCAGTATCAGCTTCCTGAGTGCCTTCCAGGCCATCACCATCAGCCCTAAAGACTCTAAGTGGGCAGCACTTAAATTCAAAGCTCCCAAGTATGCTGTTTATTGGATAAACCTTAGTTGGATTCTGAACCTCCTTATAAATACAGTTTTTCTTACAAACATAAGTTCAAAACAGGGCAATGAAAATATCACAATGCTAAAAGACTTTGGGTACTGTTCTTCTGTTAATCTTGGGATAATGGATTTAGTTCTGCATGTTGTATACCTTTCATTCCCTGATGTTATCTGTATGTGGCTGATGTTATGGACCAGTGGCTCCATGGTGCTCATCCTGTACAGGCATAAGCAGAGGATGCAACATATACTCAGAGGCAAGGTCTCTCCTGCATCCTCACCTGAGACCAGAGCTACCCAAACCATCCTTCTAATGGTGAGCACGTTTATCTGCTTTTATCTGCTCTCCTGTATCTGTCAAATTTGTTTGGCTCTTGTTTATAATCCTTCCTGGTTCCTGGTGAATATGTCTGTTTTGATCTCTGGCTGTTTCCCAACTGTGAGTCCCTTTTTACTGATGAGAAGTAACTCCTGTGTGCTGACAGTCTGCTTTATGTGTGTCAGAATATAA

>Cricetulus_griseus_intact_V1R4_55

ATGACAGGCAGGAATCTGGCCGTAGGACTGATCTTCTTATCACAGACTACATTTGGACTTCTGGGTAATTCTTCTGTCTTCTACAATTACATACTGCTTTACTTCACAAGGTACAAGCTGAATTCCACTGACTGGATTCTGAACTATTTGGTTTTAGCCAACTTCTTAACTCTTCTGTGTAAAGGAGTGCCGCAGACCATGGCAGCTTTTGGGCTGAACACTTTCATAACTGATTTTGGATGCAAGCTCCTCTTCTACCTTCACAAAATAGGCAGGGGTACATGTATTTGCAGCAGCAGCTTCATGAGTGTCTTCCAGGCCATCACCATCAGCTGCAGGGACTCCAGAGGGGCAGAGTTTAAAAACCGAACATTCAAGCACAATGGCCTCTTCCTATGCTTTTGTTGCATTCTGAACATCCTCATGAGTATATATAATCTCCTCTACATGACTCAAAATTTGGGATATAGAAACATGACAAGCATTCAAAACTTTGGCTACTGCCCTCTTTATCTTGACAAAACCGGCCAAATGCTGCATGCTGTGTTCCTGCCCCTCCCTGATGCTGTGTGGGTGGGCCTCATGGTATGGGCCAGCATATTCACTGTTCTCAACTTGTACAGGCACAAGCAAAGAGTGCAACGTATGCCAAGGACCAAAAATTCCCCAAAATCTTCCATTGAGTCAAAGGCTATTAAAACCATCCTTCTTCAGTCAAGTACATTTGTTTTCTTTTATATAATTGCCTGTCTTATTCAACTGGTTCTGGTGTTCTTTCATAATCCTAGTTGGTTTCTGGTAAACACATCTATAGTCATCTCTGGGTGTTTCCCCACTGTGAGTCCCTTTCTTCTCATGACCCACTATTCCATAGCATCTAGTAAGTGTGTTCCCAGGAAGAGAAATTTATGA

>Cricetulus_griseus_intact_V1R4_56

ATGACCTCCAGAGACCTGACTATGGGGATCTTCTTCCTGTCCCAGACTATAACAGGAATGTTGGGAAACTCAGCCTTGCTTTGTTGTTTTATCATAGCTGACGTTTCTGGGATCACAGCGAAACCAAAAGACCTGATTGTCAAGCACCTGACCTGGGCAAATTTCATAGTTCTCTGCAAAGGAATCCCACAGACAATGAATGCTTTTAGTCAGACTTACTATCTAGATTATGTTTCATGTAAACTTGCCTTATACTTTCATAGAGTTGCCAGAGGAGTGTCTCTTGGCTCCACATCCATGCTGAGTGTCTTTCAGGCCGTCACCATAAGCCCAAGCAATTCCAAGTGGGCACGGCTCAAGACCAGAGCACCCCGGATCATTGGTCCTTCTCTAGGCCTGTGTTGGGCCCTCCAACTCTTGGTATATGTCTTCCTTCCTATACACACAACCGATATATGGGGGAGAAGAAATACTTCTGGGATAAAAGATTACGGATACTGTGCTGTTATAGATTATGGGAGACTAGTCAGTACACTTACTGTAGCGCTATTGGCATTACTTGATACGATCTTTTTGGGATTGATGATGTGGGCCAGTGTCTACATGGTGTTTATCCTGCTCAAACACAAGCAGAGAGTCCAACAGATCCACAGATCCCTGTCTCCTAGGTCATCCTCTGTGATGAGAGCAACACAAAGCATTCTTACCCTAGTGAGCAGCTTTCTGTTCTTCCATATAACCTCTATTGTCTTTACCTCCTATCTCTCTGTCTTTGCTGGGACCGGTAGGTGGCTGTCCAATGCAGGTGTGGCCATGGCTGCATGCTTCCCAACGTTCTGCCCCTTTCTGCTCATCAGACACCACACCTCCCTTTTCAGGCTCTGCTGTACCAGTTCTCATTAG

>Cricetulus_griseus_intact_V1R4_57

ATGATATCTACAGACCTGGCTATGGGGATCATCTTCCTGTCCCAGACTGCACTGGGAATTTTGGGGAACTCAGCCTTGCTTTGCTGTTTAATCATCAGCGAATTCTCTGTTAAGAGGGCCAGGCCTACAGACCTAATTGTCAAACACCTGACCAGTGCTAACTTCATGGTTCTGCTCTGTAAAGGAATCCCTCAGACATTGGCTGCTTTGGGTTGGAAATATTTTCTCAATACCATTGCATGTAAAATAGTTTTTTATTTTCATAGAGTTGCCAGAGGAGTATCCCTTGGGTCCACAGCCCTCTTAGGTTTCTTCCAGGCCATTATTATCAACCCTAGGAATGCCAAGTACAAGTATTTCAAAGCCACAGACCACAAGGTAATTGGGACTTCTCTCAGCCTGTGTTGGTCCCTGCAAATGATGGTAAATGGCTTCATTCCTGTGCTCATGACAGACTTGAGGGATGCAAAAAACGTCACTGGTTTTAGAGATTTGCTATACTGTGCTGTGCCCAAACCTAGCTATGCCTTCTATGTTATCTTATTGAGCTCCTCTGATGTCATGTGTTTGGTTCTCATGATATGGGCCTGTGGTTCCATGGTGCTTGTCCTGCTGAAGCATAAGCAGAGGCTCCAATATATGCATAGAGTTCTGTGTCCCAAATCATCTCCTGAGACAAGAGCCACCCAAACCATCCTTGTCCTAGTGAGCAGTTTTGTGTTCTTTTATACAGCATCTGGTATCTTAGTCATGTGCTTAACTTTAGTAAATGGAGCCACAAGGCATTTGGTGAATACCAGTGCAGCAACAGCTGCTTGCTTTCCGGCATTGTGTCCCTATCTTCTCATCAGACACTACACCCCAGTTTCCAGGCTCTGCTGTACTTGCTAA

>Cricetulus_griseus_intact_V1R4_58

ATGGACTCAAAGAATTATGCAATAGGAATAGTGTTCTTATCACAGACTGTGTTTGGAATTGTGGGAAATTTCTCCCTTGTTTCCCACTATCTGCTTTATTACTACAATCAAAGAAAACTGAAGCCTACCGATTTGATTCTCACACATTTGACCATTGCTAACTCCTTGATCATTCTCTCTGAAGGAATACCACAAACATTGTCAACTTTTGGGTTGATACAGTTCTTCAATGATTTTGGCTGTAGACTTGTTCTTTATACTCAAAGAGTTGGCAGAAGCATGTCCATAACCAACACCTGTCTTTTGAGTGCCTATCAGGCTATCATTATCAACCCAAGTAACTCATGTTGGAAGGATTTGAAAGGGAGAGCTCCTAAGTCTATTGGATTCTCCATTTCTCTTGGCTGGGTCATATACTTGATGATAAATTTTATTTTCCCTCTGTATATTCATATCAAAAATATTGGCAATAATATGACAGTAAAACGAGATTTTGAGTATTGTTCAACTCTGGGACGAGATGCACTTGTAGATTCACTTTATGCAGGGTTATTGATGCTTCCAGAAGTCATGTTTTCTGTGTTTATGGTATTCTCCAGTGGCTCCATGGTTGTCATTCTCTATAGACACAAGCAGCGGGTTCAACATATCCATACTCCCCATATTTCCACAAGAATGTCCCCTGAGTCCAGAGCCACCCAGAGAATCCTTCTACTTGTGTCTATCTTTCTAGTATTTTATACAGTCTCCTCCATTTTACAAAGCTTCATTGCTCTTTTGTATAACCCTAGTTGGTGCTTGGTCAAGATCAAAGCCATTATTTCTATGTGTTTTCCTGCTATTGGCCCCCTTATTATGAGATGCCACTCCACTACATTGCAATCCTGCTTTTATAGGTAA

>Cricetulus_griseus_intact_V1R4_59

ATGTTTTCAAGAGACCTAATTTCTGAATTCTTCTTCCTGTCAGAACTTTTCATTGGATTCCTGGGAAACTCACTGCTCTTCATGCTATACATGTACAGCTTCTTAATTCAGCCTCGTCTGAAGAAACCCATAGATATGATTTTCACACATCTGACGCTTGTCAACGTGCTGAGCATCGCCTTCCGGCTGCTGCCAGACGTCATGGCGTCCTTCGCGGTCAAGCTCCTTTTCCATGACGTTGGATGCAAGGCAGTTTTGTACGCATACAGTGTTACCCGGGGCCTTTCCATCTGCACCATCTTGCTACTGAGTGCAATCCAAGCCATCACTATCAGTTCTAATCATTCCAAGTGGGCGTGGCTTAAGTCCAAGCTTGAGTCCTGCATTTTCCCCTCACTCCTTCTCATCTGGATCATCAATGCCTTTCTCTATATTCCCATGGTTGAAAACGTAAAGGGCCAAATCAACTTCACTGTTGTGGATTCTAGATATTCCCAGACATACTGCCGAAGTAACCAGGTTCGCCATCACACCACCTTGTCACTTGCGACTGCATTAATGATTAGAGACATCCTGTTCGTGCTTCTCATGATGGGGACCAGCCTCTACATGGTGACACTCCTGTTCAGACACAATAGGAGAACCCGGCATGTCCACAGTTCCAGAGTCTCTTCCCAGGCCTCTTCTGAAAAGAAAGCCACGCACAGCATCCTCCTGCTGGTGGGTTTCTTCATGTTTTTCTATTTCTCAAACACCTTTGTCACCTTCTATTCACTCCACGGACCTAAGAACAGCCAAGTATTGGATGTGATTAGTGGAGCTTTGTCTTCAGGCTACCCAATCATCTGCCCTTATGTTCTGATGAACAATAGGAAAATCATTTCCACATTCATTTCTTCCCTTTCAAACTTTGAATGTACCTTTTCTACAAGAGGCTGCCATGGCTAA

>Cricetulus_griseus_intact_V1R4_6

ATGCTTCCAAGCAACACTGTCTTGATGGGTTTCTTGATATCCCAACTCTGCCTGGGTGTCATAGGGAACTCGTTGCTCTTCGTGCTGTATGTGTACAGCTTCTTGGTCAAAGCTCGCTCTAGCAGACCTATCGATCCCATTTTCATGCACCTGATGATAGTCAATATATTGACGATCCTATTTGCTATGATATCATATATCGTGTCATCCTTTGGAGTCAGACATTTTCTGGATGATGCTGGCTGTAAGGCAGTTTTATATGTGTTCAGAGTCACCCAGTGTCTGTCCATCAGCACCACCTCTATTCTGAGCACATTCCAAGCCATTACCATCACTCCCAGTTATTCAAAGTGGGCGTGGCTTAAGCCTAAACTGCCATTGTGGATGTTTATCTCCTTCCTGGTTTCCTGGTTCATCAACACGCTCATATATGTGTACATCATTGAGACTGTGATAGCAAAGATCAACGACACTGACGTTGATTACGGATATTCTCATGCTTACTGTAAAAGACTACCTAATACCCGCCATCAAGGATTATTCCTGAGTGTTATCACAGTAGGAGACATCTTCTTTATGACCATCATGATATGGACCAGCCTGTACATGGTGACTCTCCTTCACAGACACCGCAGGATAACCCAGAATCTCCACGGCCCAAGCCTCTCCAGTCAGCCGTCTCCTGAACGCAGAGCCACTCACAGCATCTTATGGCTGGTGAGCTGCTTTGTGCTCTTTTACTTGCTGAACAATTTCATCACCCTTAGTGGGTTTTATGCTCGTGAAAAAAGGCCAGCATTGGGGGCCATCGTTGTAATTGTGTCGTCCGTCTACCCCACTCTCTGCCCATTTTTACTAATGAGCAATAATAGAATAATTTCACGGTTTATTTCTTCTTTATCAACCCTGAGAATTACCTGTTTTCAAAGAACATTTGGTGACTGA

>Cricetulus_griseus_intact_V1R4_60

ATGGAGTCCAGAAACTTAGCTATAGGAATATTGATCTCACTTCAGAGTGCACTTGGAATTCTGGGAAATGTATCTTTTCTTTTCCAGTATCTACTTGTTTACTACAATGAACACACATTAAAGACTGTAGACTTAATTCTTATACATGTTTTCACATCAAATTCCTTAATCATTCTCTCTACAGGAGTGCCTCAGATAATGAGAATGTTTGAGTGGAAAAGGTTCTTCAATGATGTTGAATGCAAACTTATTTTATATATTCAGAGACTTTGCAGGAGCCTGTCCATCACCTCCACCTGCCTCTTGAGTGTTTTCCAGGCCATCACCATCAGTCCTAATGTCTCCTATTGTAAAGAACTGAAAATCCAATTACCAAAATATGTTCACCTCTCCATTTCCTTCCTCTGGATCCTGTACATGATAGTGAATATGGTTTTCCCCATGTATACGGCTACCAAAAGTAATAGCAAAAACAAGACAAAAATGAGAAATTTTGAATTTTGTCCTGCTCTCAATCAAGACAAGATAGTAGAGTCACTGTATACAGTATTTTGGGTGTTCCCTGAAGTCTTATTTTCCATACTCATTGTTTGTTCCAGCATCTCCATGATTGTCATACTCTATGAACACAAGAAGAGGGTTCAGTGCATCCTCAGCACTCATGCATCCACCAGAATTTCCCCTGAATCCAGAGCCACACAGAACATCCTGGTGTTGGTTTGCACCTTTTTAGCTTTTTACACCATCTCCTCCATTTTGCAAGGCTACATGGCTCTTTCTTTTAATCTCAATTCATGGCTTGTGAATATCACAGGCATCATTTCTATGTGTTTTCCTACTTTAAGCCCCTTTGTGATGAGTCATGAATCTATTATTTCTGGATTTTGCTTTTCCTGTATAAGGAATAGGAAAGGGAAATAA

>Cricetulus_griseus_intact_V1R4_61

ATGGAGTCCGGAGACTTGGTTATAGAAATAGTGATCTCACTTCAGAGTGCACTTGGAATTCTGGGAAATGTATCTTTTCTTGTCTACTATCTACTCGTTTACTACAATAAACACACATTAAAGACTGTAGACTTAATTCTTACACATGTGTTCACATCAAATTCCTTAATCATTCTCTCTACAGGCTTGCCTCAGATAATGAGAGCTTTTGAGTGGAAAAGGTTCTTCAATGATGTTGAATGCAAACTTATTTTATATATTCAGAGACTTGGCAGGAGCATGTCCATCACCTCCACCTGCCTCTTGAGTGTCTTCCAGGCCATCACTATCAGTCCTAGTGTATCCTATTATAAAGATTTTCAAATAAAATTCCCAAAATATGTTCACCTCTCCATTTCCTCCCTCTGGATCCTGTATGTGATAGTGAATATGTTTTTCCCCATGTATACATCTACTAAAAGTAATAGCGAAAATAAGACAAAAAAGACATATTTTGAATTTTGTCCAGGTTTCAGTCATGACAAAATAGTAGAGACAATGTATACAGCATTTTGGGTGTTCCCTGAAGTCTTATTTTCTATACTCATTGTATGTTCCAGCATCTCCATGATTGTCATACTCTATGGACACAAGAAAAGGGTTCAGTACATCCTCAGCACTCAGGCCTCCACCAGAATTTCCCCTGAATCCAGAGCCACACAGAACATCCTGGTCTTGGTTTGCACCTTTCTAGCCTTTAATACCATCTCCTCTATTTTACTAGGCTACATAACCCTTTATTTTAATCCCAATTTGTGGCTTAGAAATATCACAACAATTATTTCTATGTGTTTTCCTTCTTTAAGTCCCTTTGTGACGAGTCATGATTCTATTATTTCTGGATTTTGCTTTTCCTTTATAAGGAATATCAAAAGGAAATAA

>Cricetulus_griseus_intact_V1R4_62

ATGGAGTCCAGGAACGTGGCAATAGGAGTAGTGCTCTCACTTCAAAGTGTACTTGGAATTCTGGGAAACGTCTCTCTCCTCCTATACTATCTACTCCTTTACTACAATGAAGGCACATTAAAGACCATAGATTTCATTCTTACACAGATATTTACCGCCAACTCCTTGATTATTCTCTCCAAAGGAACGCTGCAGATAACAGGCGCCTTTGGGTGGAATCAGTTCTTTAATGATGTTGGATGCAAATTTATTTTATATATGGTCAGACTCGGCAGAAGCATGTCCATCACCACCACCTGCCTCTTGAGTGTCTTCCAGGCCATCACCATCAGCCCCAGGAACTCCTGTTGGAAGAATATCATGGCCAAAAACCCATGGTTCATGAGCTTCTCCATTTCCCTCTGCTGGATACTACACATGGTGATGAATATGATTTTCCCTACATATCCATCCACCAGGAGGAATAGCAAAAATATGACACAAAAAAGAAGTTCTGAATTCTGCCCCTCCTCTCCAGGACGTGATGCTATAAGACAATCACTATACATGGTATTTTGGGTGTTACCAGAAGCCTTGTTTTCGGTGATCCTGGTCGGTTCCAGCAGCTTCATGATTGTCATACTGTATGGACACAAGAAGCGTGTTCAACATATCCATAGTTCTCATTCTTACCCCAGAAACTGCCCTGAGTCCAGAGCCACCCAGAACATTCTAGTCCTCGTGTGCACATTTCTGGGTTTTTACTCACTCTCTTCTGTCTTACAAGGCTGCCTTACTCTTTCACATAATCCTAGTTGGTGGCTAATGAACACCACAGCCATTATTTCTGTGTTTTCCTACTTTAGGCCCCTTTCTCATGAACCGTGA

>Cricetulus_griseus_intact_V1R4_63

ATGATATCAACAGACCTGGTCATGGGGATCATCTTTGTGTCCCAGACTACACTGGGAATGATGGGGAACTCAGCCTTGCTTTGCTGTTTCATCATCAATGAATTCTCTGGGATCAAAGCCAGGCCTACAGACCTTATTGTCAAAAGCCTGACCTGTGCTAACTTCATGGTTCTGCTCTGTAAAGGAATCCCTCAGACAATGGCTGCTTTTGGTTGGACATATTTTTTAGATACCATTGCATGTAAACTTGTCTTTTACTTTCATAGAGTTGCCAGAGGAGTATCTCTTGGGTCCACAGCCCTCTTAAGTTTCTTCCAAGCCATCATGATCACCCCAAGGAATGCCAAGTACAAACACTTCAAAGCAAGAGCCTACAATGTCATTGGACCTTCTGTCAACATGTGTTGGTCCCTGCAAATGGTGTTAAATGCCTTCATTCCTGTGCTAGTGACAGACCTGGGGGATGGAAAAAACTGCACTGATTTTCGTGATTTGCTATACTGTGCTGTGGCCAAACCTCACAATCTAACCTCTGCCTTATATGTTATCTTATTGGGCTCCTCTGATGTCATGTGTTTGGTTCTCATGATATGGGCCAGTGGTTCCATGGTGCTCGTCCTGCTGAAGCACAAGCAGAGGGTCCAATACATGCATAGAGTTCTGTGTCCCAAATCATCTCCCGAGACCAGAGCCACCCAAACCATCCTTGTCCTAGTCAGCAGTTTTGTGCTCTTTTATACAGCATCTGGCATCTTAACCATGTGCTTAACTTTTGTAAACGGAACCTCTCGGGGGCTGGTCCATGCCAGTGTAGCCACAGCTGCTTGCTTTCCAGCATTGTGTCCCTTTCTTCTCATCAAACACCACACCCTAGTTACCAGGCTCTGCTAA

>Cricetulus_griseus_intact_V1R4_64

ATGAAGTTAGAAATGGTTTTGAGTATTAGTGAAATGACAATTTTCCTTCTTCTAACTGGACTTGGCACTGTGGCAAATGTTTCCGTTTTTGTGAATTACATTTGCACTTTTATGGGTTCTGGGGAGAAATCTATACACCTTATTCTCATCCACCTGGCTTTTACAAATATTACAATGCTTTTTTCCAAGGGTGTGCAAACGCCAATTACAGCTTTAGGTTTGAGACACTTCCTAGGTGATGTTGGCTGTCACATCATTGTTTACCTGGAAAGGGTGGCTCGGGGCCTCTCCATCTGCACCAGCAGTCTCCTCACAGTGGTCCAAGCCATCATCATGAGCCCCAGAACCTCCTGGTGGGGGAGGCTAAGACTGAGGTCTGTAGAATGTGTGCTTCTCTCACTGCTCTTCTTTTGGATTCTCAATTCCTTGATCAGCATGAATCTCCTCTATTCCATCAGAAACAGAGGTACAAACATTTTACAACAGAGTAAAAATAATAAATATTGTTATATCAAAAAAGAAAGTCAGAAAGTAAACAACATGTTTCTTGCCCTCATGGTCCTGAGAGATGCAGTGTTTCAGAGTGCCATGGGAGGGGCCAGTGGCTACATGGTACTTCTTCTCCACAAGCATCACCAGCATGTCCTCTACCTTCAGAACTCCAAGCTTCTCTACAGAACTCCCCCTGAGCTGAGAGCTGCTCAGAGTGTCCTCCTGCTGATGCTCTGTTTTCTTTTCTTCTATTTGGCTGATTGTGCTGTTTCTTTATATTTATCCTTCTCTTTGAAGAATGACTCCTTAACAATGCATTTGCAGCAATTTCTTACCCTTGGTTATGCAATTCTTAGCCCATTTATCCTGATTCACAGGGATGGACATCTGGCTGAATGTTGGCATGCTCAGTAA

>Cricetulus_griseus_intact_V1R4_65

ATGGTTTTGGACCCTGTCAAGGGCACAGTCTTCCTCTGTCTCACTGGGATTGGCATCATGGGCAACATCTTAGTTTTTGTGAGTTACGTGCACATGTTCCAAAGCACTGAGAAGAAACCTATACACCTCATTCTTGTTCACTTGGCACTCACAAATATTGGAATGCTTCTTTCAAAAGGGATGCCAAAGACAATAGAAGCCTTTAATTTTGGAAACTTCTTAGATGACACCAGTTGCAAAGTTGTTGTTTATCTGGCCAGAGTGTCCCGGGGCCTCTCCATCTGCACCAGTAGTTTTCTCACCATGGTCCAGGCCATCACCATCAGTCCCAGACACTCCCGGTGGCAGAGGCTCAAACTAAAAACTCCACAGCACATTCTTTCCTCGATGCTCTTCTTCTGGATACTCAATTCCTTGATCAGCATGAACTTACCATATTGCATTAAAAGTATCAACAGTGTGAACATAACACAGATTAAAAGGGGGGGCAACTATTGCTATTTTGTGCCAGAAAGTCAGATAACAAGATGGATTTTTCTTACCCTCATGGTCCTGAGAGATGCAGTGTTTCAGGGTGCCATGGGAGGGGCCAGTGGCTACATGGTACTTCTTCTCCACAAGCATCACCAGCATGTGCTCTACCTTCAGAACTCCAAGCTTCTCTACAGAACTCCCCCTGAGCTGAGAGCTGCTCAGAGTGTCCTCCTGCTGATGCTCTGTTTTCTTTTCTTCTACTGGGCAGATTGCTTTATTTCTTTGTATTTTACTTTCTTCATAGAGAATTATTCCAAAATACTATATGTTCCAGAGTTTCTAACCCTTGGTTATGCAGTTATCAGCCCCTTCATACTTATTCACAGAGATGAACATCTGACTAAATGTTGTCATACTCAGTAA

>Cricetulus_griseus_intact_V1R4_66

ATGCACTTTCCACATATGTCCTCATTAAAGAATGTCCTTTATTTCCAAGCTGGACTTGGAGTCATCACCAATATGTTTCTCCTTTTTATCTACACTTTCATAATCCTAGTACATAAATCTAAGCCCATGGACCTGATCTCCTGTCAACTGACCTTCATCCACATAGTGCTGATTCTCACTGGAGGGGGTATTGGGCTTACAGATGTATTTGAGTCACTGAACTCTGAGAATGACTTCAAATGTAAGACAACTTTTTACATAAACAGAGTGATGAGAGGCCTCTCCATCTGCATCACCTGCCTCCTGAGTGTGTTCCAGGCTGTCACTATCAGTCCCAGTACCTCTTTCCTGGCAAAATTTAAACTTAAACTAAAAAAATACATGATCTCTGCTTTCTTATTCATTTGGTCTTTCAATTTGTCATTCAGTAGTAGGTGGATCTTCTATGTTGGGGCTTTTACCAATATGAGTGAGACCAACCAGATGAAGGTCACCAAATATTGCTCTCTCTTCCCCGTGAACTATATCATCAGGGCACTGATTTTAACAGTGACAACCTCTAGAGATGTGTTTCTTGTAGGAGTTATGCTGACCACAAGTGCATACATGGTGATTATCTTGTTCAGACATCAGAGGCAATGCAAATATCTTCACAGCCTCAGCCGCCTGAGAGCATCCCCTGAGAAACAGGCCACTCTGACCATCTTGCTGCTGGTGGTTGTCTTTGTGGTCATGTACTGGGTGGACTTCATCATCTCATCCATTGAACTTTTGTTATGGATTTACCATCCAGCCATCCTGACTGTTCAGATGTTTGTGATGAATGTCTATCCTACGATTACTCCTTTGGTACAAATCAGTTCTGATAACAGAATAATCAATATGTTGAAAAAGCATGCATTAGGTATTTTTAAAAGATGA

>Cricetulus_griseus_intact_V1R4_67

ATGAAGATGAGTTGGAGATACCTCATCCAGAGATTAACTTTCTTTTCACTTAGTGGGCTTGGAGTTTTAGGGAACATGATCTTATTTGTGAGACATGTGTATACTTTTATTATGAGTCCTGAGAAAAAATATATAGATGTTATTCTCGTCCACCTGGCTTTTGTAAACACAATCATTATTCATTGCATAGGGGTCAGAAACATAGCCACAAATTTTTATTACAGAAACTTCCTAGGTGACGTTGGTTGTAAAACTATAATTTATCTAGAAAGGGTGGCTCGTGGCCTCTCCATCTGCACCACCTGTCTCCTCAGCATGGTCCAGGCTGTCACCATCAGTCCCAGGACCACCCTTTGCAGAAAGGTCAAACCACAGACTGCATGGCAAGTTCTTCCCTTTCTCCTCCTCTTTTGGATCTTTAATTCCCTGATAAGCTCTAACTTGCTCCACTATATCACAGCAGCCAGGAGCACGAACAAGTCTAATGTTGCGATGTATACTGGGCATTGCTATATGCTGCCATCCAGGCACATAGTTAAGTGGCTGTTTCTCTCTCTCATGGCTCTTCGTGATGTCATTTTTCAGAGTCTCATGGGCTGGAGCAGTGGATCTATGGCTCTCCATCTGTACAAACATCACATACGTGTCCTGTACCTTCGAAGCTCCAGGTTCCCAAATAATTACAGTCCTGAAATCAGAGCTACCCATAGTGTTCTCACTCTCATGACCTGTTTTCTTTTCTTTTATTGGGCAGATTTCATTTTCTCCTTCTATATAGGCTCCATAGTGACACATGACTCCACAATACTCAATATTAAAGCATTCTTAGTGCTTAGCTATGCTGGCCTCAGTCCCTTTGTCCTGATCATCAGGGATATCCATGTTGCTAAGCATTGCTGTGTTCTGAGAAATACAAAATTCCTTTTCACAACTAGTTCTTTATGA

>Cricetulus_griseus_intact_V1R4_68

ATGGACTTTTGGAATCTGGCAATAAGCTTTATTTACTTATCACAAACTACAGCTGGGATTCTGGGAAATTTCTCCCTAATTTTCTACTATCTAGTCATTTATTGCAGAGAAAACACATTAAAGCCCACAGATTGGATTCTCACGCACCTAATGGCAGCCAATGCCTTGATCATTCTCTCTACAGGAGTGCCTCAAACAATGGCAGTTTGGGGACTGAAGCATTTCTTGAATGACTTTGGATGTAAACTACTGTTGTACTTTCAAGGACTTGGTAGGAGTGTGTCCATTGGTACCACCTGCATCTTGAGTGTCTTCCAGGCTTTGACCATTAGTCCCAGGAAATCATGTTGGAAGCGTCATAAAGTCAAAGTTGAGAAGATCACTGGCTACCACGTCTCCCTCCTTTGGATCCTGTTCATCTTGATTTTTCCATATTCGTTTGTTAATTCGGGCAGCAAAAATGTGACCAGAAAACAAGATTTTGGTTACTGCTCCATTGTAGGATGGGATGGAACTGGCCCTTCACTCTATGCAGCACTGGTGGTGTGCCCTGAAGTCTTCATTTCTGTGCTCTTGGCGTGGTCAAGTGGATCCATGATTGTCATTCTGTACAGACACAAGCAGAGGGTTCAACACATCCGCAGCACTCATGGTTCCAGCACAAAATCTCCTGAATCCAGAGTCACAGAGAATATTCTTATCCTTGTGTTTACCTTTCTGGCTTTTCACACTCTTTCTTCCATCTTAAGAGGCTACATTATTCTTTTATATAATGACAATTGGTGGTTGGTGAACACCAATCGCATCATTTCTCTCTGTTTTCCATCATTTTTACCCTTTGTTCTCATATATCGTTACTCTATTGTACCCAAAATTTCTTTGGTCTGTATAAGAAATACAAATAATCTAATCTCATTTTAA

>Cricetulus_griseus_intact_V1R4_69

ATGCAGGATCCTCAGGCTGACAGCAGGACCCAACACCTTGGCAGCGTAGGAGTGATCTTCTTGTTACAGACTGTGGTTGGAGTTCCGGGCAATTCCTATCTCCTCCACCATTGCCTGTTGTTTCACCTCAGAGGGCTCAGGTTAAGGTCCACAGATTGGATTCTGATGCATTTGTTTGTAGCTAATATTTTAAGTTTGTTCTGTAAAGGGGTGCCCCAGACATTACGTGCATTTGGATGGAGAGACTTCCTTAATGATTTTGGATGCAAATGGTTTTTCTATCTCCACAGAGTGGGTAAAGGTATGTCCATAGTCAGCACTAGCCTTTTGAGTGTCTTCCAGGCCATCACCATCAACCCCAGGGACTCCAGGTGGGTACAGCTTAAAGGAAGAACTCACAAATACATTCGGTCTTCCGTGTACCTGAGCTGGATCCTGTACGCGTTTGCCAGCACTCTTAATCTTGTGTACGTGAGAGCAAAATACAGTAAGAACAACACAGCAAACCTAAAAGATCTAGGATACTGTGCTGCTGTTCGTCTTGATAAGACCAGTGACATACTGTATGCAACATTCCTGTCAGTTCCTGATTTTCTCTTTGTGGGGCTCATGGTGTGGGCCAGCATCTCCATGGTTCTCACTCTGCACAGACATAAGCAGAGGACGAAATACATACACCAGGTTAAGGATTCTTCCACATCCTCTCCTGAGTCCAGAGCCACTCAAACTGTCCTTCTCCTGGTGAGCACCTTTGTATTCTTTTACACACTCTCTTGCATATTGGTCATCTGGTTAACTTTTTTTACTAATCTCAGTTGGTTCCTGATGAGCGTGTCTGTGTTAATTGCTGGATGCTTCCCAGCTGTCAGCCCCTTCCTGCTCATGAGCCATTTCTCTGATACATCATGTTGTTTATGCTTTCTCTGCATAAGGAATGGCAATAAAAGTAACTATTTTAAGACAAACAAATAA

>Cricetulus_griseus_intact_V1R4_7

ATGAATATCTGGAATATGGCAATCAGAATCATTTTCTTATCACAAACTACAACAGGAATTCTTGGAAATTTCTCTCTTTTGTTTTACTATCTAGTCCTTTACTGTAGAGAACACACATTAAAGCCCACAGATTTGATTCTCACACACCTAATGGCAGCCAATGCCTTGATCATTCTCTCTGCAGCAGTGCCCCAAACAATGGCAGTTTGGGGATTTAAACATTTCTTGAATGATTTTAGGTGTGAGCTCCTATTGTATATTCAAGGATTTGCTAGAAATGTGTCCATTGGCAGCACCTGTCTTTTGAGTGTCTTCCAGGCAATGACCATCAGTTCCAGGAAGTCATGTTGGAAGGATCATAAAGCCAACGCTACAAAGTACATTCACTGTTCTGTTTCACTACTCTGGGTTTTCTACATGCTGATACGTTTTATTTTCTTGATGTACACATTTATCAAAATGAGTAGCAAAAACATGACAAGAAGTCAAGATTTTGGATATTGCTCTACTGTAGGCTGGGATGAAATCATAGAGTCACTCTATACAGCCTTGGTGATGTGCCCTGAATTCTTTTTTGCTGTGCTCATCACCTGGTCCAGCGCCTCCATGATTGTCACCCTGTACAGACACAAGCAGAGTGTTCAACACATCAGAAGTTCTCATGGTTCCAGGAGATCCTCCCCTGAGTCCAGAGCCACCCAGAACATCCTGGTGCTGGTGTCTACTTTTCTGGCTTTTTATAGTCTCTCTACCATCTTGCGAGGCTGCATTGCTTTTTTGTATAATCACAATTGGTGGCTTGTGTACATCTCGCACCTCACTTCTCTTTGTTTTCCCTGTTTTGGACCCTTTGTTCTTATGAGACATCACTTGGTTTTGTCCATATTTAATTTGGTATGGTTAAGAAAACATTTTTACTGA

>Cricetulus_griseus_intact_V1R4_70

ATGTCTGCTCATAATAAAGCCCTGAAAACCACTGAGGAAGCAGCTCTTCAGATGATCTTGAATTGCCAATTTGGGGTTGGGACTGTGGCCAACATCTTTCTGTTTGTCCATAATTTCTCTCCAGTCTTGACTGGCAATCGGATGAAGCCCACCCAGGAGATTCTCAGCCACATGGCTGTGGCCAATGCCTTCATCCTCCTCATCACTGTGTCTCCAAACAATATGATGGTTTTTGCTCCAAGGATTCCGCTTACTGACTTCAGATGTAAACTTGAGTTCTTCATTCGCCTGGTGGCTCGAAGCATAAACTTGTGCTCCACCTCTGTCCTGAGTACCTATCAGTTTGTCACTCTTGTTCCTGGTCATTGGGTTAGCCTCGTGCTCAGAGGAAGAGTGACACATTTGGTGAGTTATTCTTGTTATGGTTGTTGGTTGGTCAGTGTCATAAATAATGTCTACATTCCAATGACAGTCACTGGTCCACAGAAACCAGGCAATGACACTGTTCCTAGAAGCAAGTTGTTCTGTTCCACCTCTGGTTTCAGTGTAGGCATTGTCTTCTTGCGTTTTGCTCATGATGCCACGTTCATCAGCATCATGACCTGGACCAGTGTCTCCATGGTGATTCTCCTACGTAGACATCACCAGCGAACAAAGCACATCCTCACTCCCATTCAGGACCACAGAGTCCATGCGGAGACCAGAGCGGCCCACACCATCCTCACGCTGGTGGTCACATTTGTTAGCTTTTACCTCTTAAATTTTATTTGTATCATCTTTCACAACTTGTTAATAGACTCTCGTCTCTGGATGAGACATGTAGGTGAAGTTTTGGCCGTAAGCTTTCCCACTATCTCTCCTTTTCTGTTGATCTTGAGAGATCCTAAGGATCCTTGTTCTCTGCTCTTCCACCATTGA

>Cricetulus_griseus_intact_V1R4_71

ATGGACTCCAGAAACTTCACTACAGGAATAGTGATCTCACTTCAAAGTACACTGGGAATTCTGGGAAATGTATGTTTTCTTTTCCACTATCTACTCATTTCCTACCGTGGACACACATTAAAGACTGTAGACTTAATTCTTACACATGTGTTCACAGCAAATTCCTTAATCATTCTCTCCAGAGGAGTGCCCCAGATAATGAGAGCTTTTGAATGGAAACGGTTCTTCAATGATGTTGAATGCAAACTTATTTTCTATGTTCACAGACTTGGCAGGAGCATGTCCATCACCTCTACCTGCCTCTTGAGTGTCTTCCAGGCCATTACCATCAGTCCTAATGTCTCCTATTGTAAAGATCTTAAAGTCAAATTACCAAAGTATGTTCACCTCTCTATTTCCTTCCTCTGGATCCTGTACATGATAGTGAATATGGTTTTCCCCATGTATTCAGCTACTAAAATTAATAGCAAAAATAAGACAAAAATGAGAGATTTTGAATTTTGTGCCTCTCTCAGTCGTAACAAAATAGTAGATTCACTGTACACAGCATTTTGGGTGTTCCCTGAAGTCTTATTTTCTACACTCATTGTATGTTCCAGCATCTCCATGATTGTCATACTTTATGGACACAAGAAGAGGGTTCAGTACATTTTCAGCACTCAGGCCTCCACCAGATTTTCTCCTGAATCCAGAGCCACACAGCACATCCTGGTCTTGGTTTGCACCTTTCTAGCTTTTTACACCATCTCCTCAATTGTACAAGGCTATATGGCTCTTTCTCATAATTCTACTTCATGGCTAATGAATATCACAGCTATCATTTCTATGTGTTTTCCTACTTTATGCCCTTTTGTCATGAGTTATGATTCCACTGTTTCAAGGTTTTACCTTTTTTGGATAAGGAATATAATGATGAAATAA

>Cricetulus_griseus_intact_V1R4_72

ATGTCCTCATTAAAGAATGTCCTTTATTTCCAAGATGGACTTGGAGTTCTAGCCAATATGTTTCTCCTTTTTTTCTATACTTTCATAATCCTGTGTCACAGATCTAAGCTCATGGACCTGATCTCCTGTCAGCTGCCCTTCATCCACATAATGCTGGTCCTCACTAGAGGGGATATGTGGCTTACCGATGTATTTGAATCACTGAAGTTTGACAATGACTTCAAATGTAAGGCAACTTTTTACATACACAGAGTGATGAGAGGCCTCTCCATCTGCATCACCTGCCTCCTGAGTGTGATCCAGGCTGTCACTATCAGTCCCAGTACCTCTTTCTTGGCAAACTTTAAACTTAAGCTAAAAAAATACATGACCTATGCCTTCTTCTACATTTGGTCTTTCAATTTGTCATTCAGTATCAACCTGATCTTCTATGTTGGTGCTTTTACAAATTTGAGTGAGACTAACCAGATTAAGGTCACTAAATCCTGCTCAATCTTCCCCATGAACAATATCATCAGGGCACTGATTTTAACAGTGACAACCTCCAGAGATGTATTTCTTGTAGGAGTTATGCTGACCACAAGTATATACATGGTGATTATCTTGTTCAGACATCAGAGGCAATGCAAGCATCTTCATAGCCTTAGCCACCTGAGAGCATCCCCTGAGAAAAAGGCCACCCAGACCATCTTGCTGCTTGTGGTTTTATTTGTGATCTCGTACTGGGTGGACTTCATCATCTCATCCACTGCAGTCCTGTCATGGATGTACCACCCAGTCATCCTGACTGTTCAGAAGTTTGTGATGAATGCCTATCCCACAATTACTCCTTTGTTCCAAATCAGTTCTGATAACAGAATTTTCAATATGCTGATAAATTTTCAGTCAAAGTGTCGCAAGATTTGTTAA

>Cricetulus_griseus_intact_V1R4_73

ATGATTTTGAATTTTATTAAGAAAATAATTGTCCTCTTCATGACTATGGTTGGTACTCTGGGGAATATTTCTGTTTCTATGAACTATATGTTCAGTTGGTGGGCAGGCCCCAAGAAGAAACCCATACACCTTATTCTCATCCACTTGGCCTTTGCAAACATTATAATCCTTCTTGGAAAAGGATTGCCAAAGACAATGGCAGCATTTGGATTGAGAAACTTCCTAGATGACATAGGCTGTAAAACCCTTATTTACCTGGAAAGGGTGGCCCGTGGAGTCTCCATCTGCACCAGCAGTCTCCTCACTGTGGTCCAGGCCATCATCATCAGTCCCAGAGCATCTGGGTGGAGGAGGTTCAGACCAAAGTCTGCATGGCACATCCTTCCATTCTTTTCATTCTTTTGGGTGCTCAATTCTTTAATAAGTATGAACCTAATCCATTCCATCACAAGCACAAGCCTGAATACATCACAGCTTGAGAATGAAAACAACTATTGCCATTTTATGCTAGAAAGTCAGAAAACAAAGTGGATTGTTCTCCCTCTCATGGTCCTGAGAGATGCAGTGTTTCAGGGTGCCATGGGAGGGGCCAGTGGCTACATGGTACTTCTTCTCCACAAGCATCACCAGCATGTCCTCTACCTTCAGAACTCCAAGCTTCTCTACAGAACTCCCCCTGAGCTGAGAGCTGCTCAGAGTGTCCTCCTGCTGATGCTCTGTTTTGTTTTCTTCTATTGGACAGACTGTGCCTTTTCTCTGTTTTTAAGTCTCTCTTTAGGGAACAATTTCTTGATGACAAATGCTCAACATTTTCTGGCCCTTGGTTATGCAACTTTTAGCCCCCTTGTGTTGATTCACAGGGATGGACTTCTGACTGAGTGTTGGCATGCTCAGTGA

>Cricetulus_griseus_intact_V1R4_74

ATGGTTTTGAAGTTTATTAAGGAAATAATTTTCCTCTTGATGACTATGGTTGGTACTCTGGGGAATATTTCTGTTTCTATGAACTATATGTTCAGTTGGTGGGCAGGCCCCGAGAAGAAACCCATACACCTTATTCTCATCCACCTGGCTTTTACAAACATCATAATCCTTCTTGCAAAAGGATTTCCAAACACAGTAGCAGCATTTGGATTGAGAAACTTCTTAGATGACATAGGTTGTAAGACCCTTATTTACCTAGAGAGGGTGGCCCGTGGCCTTTCCATCTGTACCAGCAGTCTCCTCACTGTGGTCCAGGCCATCATCATCAGTCCCAGAGCATCTGGGTGGAGGAGATTCAGACCAAAGTCTGCATGGCACATCCTTCCATTCTTTTCATTCTTTTGGATGCTCAATGCTTTAATAAGTATGAACCTAATCCATTCCATCACAAGTATTAATTTGAATATATCGAAGCTTAAAAATGGCTACAACTATTGCCATTTTATGCTAGAAAGTCAGAAAACAAAGTGGATTGTTCTCCCTCTCATGGTCCTGAGAGATGCTGTGTTTCAGGGTGCCATGGGAGGGGCCAGTGGCTACATGGTACTTCTTCTCCACAAGCATCACCAGCATGTCCTCTACCTTCAGAACTCCAAGCTTCTCTACAGAACTCCCCCTGAGCTGAGAGCTGCTCAGAGTGTCCTCCTGCTGATGCTCTGTTTTGTTTTCTTCTATTGGACTGACTGTGCTTTCTCTCTATTTTTAAGTCTCTCTTTAGGGAACAATTCTGAGATGATAGTTATTAGAGAATTTCTTGTTCTTGGTTATGCAATCTTCAGCCCTCTTGTGCTGATTCACAGGGATGGACTTCTGGCTGAGTGTTCACAAAGTCAGTGGAAGAAATTGAGAAACTATCTCACTTATCTGTTTAATAAGCACAGATGA

>Cricetulus_griseus_intact_V1R4_75

ATGCCCAATAACAGAATGTATTTCTGGACTCTGATCATCAAAATAATTTTCTTATCACTAACTACAGTAGGAATTCTTGGAAATTTCTCCGTGTTTCATTACTATCTGGTCTGCTATGGACACTGCAAATTAAAGACTGTAGATTTGATTCATGTGCACCTGATGGCATCCAACACCCTGATCATTCTCTCTAAAGGAGTGCCCCACACGATGGCAGTTTTTGGTTTGAAGCACTTTTTAAATGATATTCACTGCAGATTAATTTTGTACATTGAAAGAGTTGGACGCAGAGTGTCCATTGGCTCCACCTGCCTCTTGAGTGTCTTCCAGGCTGTCACCATCAGTCAGAAGGAATCCTGTTGTAAGGATCAAAAATTCAAAGCTGCTAAGTACATTGGCTGCTCCATTTCCCTTCTCTGGGTCTTGTACATATTAATACATTTCATTTTCTTTGTGAATCCACTCATCAAAAGGTCTAGTAACAATGTGACAGGAAAACAAGATTTTGGGCACTGCTCTATTGCAGGGCAAGATGGAATCAATGAATCACTCTATGCAGCATTGGTGGTGTGCCCTGAAATCTGCTTTTCATTGCTCATGGTCTGGTCTAGTGGCTCCATGATTGTCATTCTGTACAGACACAAGCAGAGGGTTCAGCACATCAGTAGCACCCATGGTTCCAGCAGAACCTACCCTGAGTCCAAAGCCACCCAAAACATCCTGGCACTGGTGTCTACCTTTCTGGATTTTATACTGTCTCTGCTATCTTACAAGGCTGTGTGGCTCTTTTGTCTAATCCTAGTTGGTGGCTGGTGA

>Cricetulus_griseus_intact_V1R4_8

ATGATCATGCTTCCAAGTAATACCATCTTGGGGGTCTGTCTCATAACTCAGTTATGTGTTGGTGTCACAGGTAACTCATTACTGTTTATTTTATTCATATATACTTTCTTCTTTAAGCCTCATTTTAAGAAGTTGATTGATTCAATTTTCATGCACCTGACAATAGTTAATGTGCTGATGATCATATTCACATTGATATCCCCTATCATGTCGTCCTTTGGAGTACCCAAATTTCTGGATGATGCTGGCTGTAGGGCAGTGTTATTTATATCTTTGTCATCCTTACTTTGCTCCTGGCTCATTAACCTGCTCATCTATGCATATATGGTTCCAATGGTTATAGCCAAAACCAATTCTACTCACTTTGGCAATGGATATTTGGATCCTTACTGTCAAAACAAGCACTTTGGGAAACAAAATTCAGGGTCATTTTTGAGTGTCATTCTCATTTATGATCTCTTCTACGTGGCCATCATGATGTGGACCAGCCTGCTCATGGTAACTGTCCTCTACAGACACCGAAAGAGAGTCCAGCATCTCCACAGCACAAGCCTGTCCTGCCAGCCATCTCCTGAGGGCAGAGCCACTCACAGTATCTTGTTGATGGTGAGCTGTTTTGTCTTCTTTTATTGGTTGAACAATTTCATCACCCTTTCTGGTTTTTATGTACAAGTAAAAATTCCAAACTGGGAGGGAATTAATGCAATTTTGGGAGCATGCTACCCAACCATCTGCCCTTTTTTACTGATGAAGAATAATAAACTTGTTTTGCAATTCACTGCTTCCTTTTCTGAAAAGAAGATGGCCTGCTTTCAAAGTGCACTCGGTGGCTGA

>Cricetulus_griseus_intact_V1R4_9

ATGTCCTCATTAAAGAATGCCCTTTATTTCCTAACTGGACTTGGAGTCCTGGCCAATATGTTTCTCCTTTTTATCTACACTTTCATAATCCTAGTTCATAGATCTAAGCCCATGGACCTGATCTCCTGTCAACTGACCTTCATCCACATAGTGCTGGTCCTCACTGCAGGGGATATTGGGCTTTCAGAGGTATTTGAGTCACTAAACATTGAGAATGACTTCAAATGTAAGACAATTTTTTACATAAACAGACTGATGAGAGGCCTCTCCATCTGCATCACCTCCCTCCTGAGTGTGTTCCAGGCTGTCACTATTAGTCCCAGTACCTCTTTCCTGGCAAAATTTAAACATAAACTAAAAAAATACATGATCTATGCTTTCTTCTACCTTTGGTCTTTCAATTTGTCATTCAGTAGTAGGTGGCTCTTCTATGTTGGTGTTTTTGTCAATGTGAGTGAGACCAACCACATGAAGGTCAATAAATACTGCTCACTCTTGCCCATGAACTCTATCATTAGGGTACTGATTTCAATGGTGACAACCTCTAGAGATGTATTTCTTGTAGGAGTTATGCTGACCTCAAGTGCATACATGGTGATTATCTTGTTCAGACATCAGAGGCATTGCAAATATCTTCACAGCCTCAGCCGCCTGAGAGCGTCCCCTGAGAAAAAGGCCACACAGACCATCTTGCTGCTGGTGGTTGTCTTCGTGGTCATGTACTGGGTGGACTTCATCATCTCATCCACTGCAGTCCTGTTATGGATGTACCACCCAGTCATCCTGACTGTTCAGAAGTTTGTGATGAATGCCTATCCCACAATTACTCCTTTGGTACAAATCAGTTCTGATAACAGAATAATCAACATGCTGACAAACTTGTGGTCAAAGTGTATTGGATTAAGGTCTCTTTATTTCAGAGGATAG

>Cricetulus_griseus_intact_V1R54

ATGAATCAAATCAATAAACTGTCCCATAACACCAAGTTAAGAAACACCATTTATTCTGAAGCTGGAATTGGGATCTTAGGCAACTGCTTCCTTCTTCTCTTCCATATCCTCAGGCTCATCCGTGGGCAGAAGCCCAGACTCACTGACCTTCCCATTGCGGTCCTGGCCCTCATCCACCTGCTGATGCTGATAGTCATGAGTTTAGTAGCTACAGACATTTTAATGCCTTGGAGGAGGTGGAGTGATACCACATGCAAAGTTATTATATCCTTGTACAGGTTTTTTAGGAGCCTCTCTCTCTGTGCCTCTAGCCTGCTCAGCATCCTCCAGGCCATCACCCTCAGTCCCAGAACCTCCTGTCTAGCAAAGTTCAAATGTCAATCTGCACACTACATGCTAGGTTGTCTTCTTTTCCTCAGTGCCCTCTCTGCGTCCATTAGCAGTCACCTCGTGTCATACGTGACTGCGACCCCTAATTCAACCTCCTCTAGTCTTATATACCTTACTGAATCTTGCTCTCTTACACTCATGAGTTACTCTGTCCGGCATACGTTTTATATATTATTAACTGTCAGAGATGTCATCTTTGTAGGTCTCTTGGCCCTCTCCAGTGGGTACATGGTGATTTTCCTATGCAGACATAAGAAGCAGTCCCAGCATCTCCACAGCACCAGCCTTTCCCTTAAATCATCCCCAGAACAAAGAGCCACACGGACCATTTTGTACCTCATGAGTTTCTTTGTTGTCATGTACACTGTGGACAGCTGCATGGCCTACTTAAGAATAAATGGTGATCCTATGTTATATTGTATATCCATTCTCATAGGTCACAGCTATGCCACAGCCAGTCCTTTATTGGTTCTCAGTGCTGAAAAAAGTATAATTAACATTTTCAAATCCATGTATGGGAGGGCAGTAAACATGTTATTGCTCAAGAATGGGTAA

>Cricetulus_griseus_intact_V1R90_1

ATGTCCTTATTAAACATTGTCCTTTATTTCCAAGCTGGACTTGGAGTTCTAGCCAATATGTTTCTCCTGTTTTTCTACACTTTCATAATCCTGTGTCACAGACCTAAGCTCATAGACCTGATCTCCTGTCAACTGGTCTCTATCCACATAGTGCTACTGCTCACTGGAGGGGATACATGGCTTACAGACATATTTGAGTCACTGAACATTGAGAATGACTTCAAATGTAAGACAACTTTTTACATACACAGAGTGATGAGAGGACTCTCTATCTGCATCACCTGCCTCCTGAGTGTGATCCAGGCTGTCACTATCAGTCACAGTACCTCTTTGCTGGCAAAACTTAAACTTAAGTTAAAAAAATACATGACCTATGCCTTCTTCTACATTTGGTCTTTCAATTTGTCATTCAGTAGCAACCTGATCTTCTGTGTTGGTGCTTTTACCAATGTGAGTGAGACCAACCAGATTAAAGTCACTAAATCCTGCTCCATCTTCCCCATGAACAATATCATCAGGGCAGTGATTTTAACAGTGACAACCTCCAGAGATGTATTTCTTGTAGGAGTTATGCTGACCACAAGTGCATACATGGTGATTATCTTGTTCAGACATCAGAGGCAATGCAAGCATCTTCATAGCCTTAGCCACCTGAGAGTATCCCCTGAGAAAAAGGCCACCCAGACCATCTTGCTTCTGGTGGTTGTCTTTGTCATCATGTACTGGGTGGACTTCATCATCTCATCCACTGCAAGCCAGTTATGGATGTACAACCCAGTCACCCTGACTGTTCAGAAGTTTGTGATGAATGCCTATCCCACAATTACTCCTTTGGTACAAATCAGTTCTGATAGAAGAATAATCAATATGCTGAGAAACTTGTACTCAACATGCCATCAGATTTTTAAAAAAGTGTAA

>Cricetulus_griseus_intact_V1R90_2

ATGTCCCCATTAAGGAATGTCCTTTATTTCCAAGCTGTTCTTGGACTCCTCGCCAATATGTTTCTTCTTTTTTTCTACACTTTCATAATCCTATTTCATAAATCTAAGCTCATAGACCTGATCTCCTGTCAACTGACCTTTGTCCACATAGTGCTGCTCCTCGTTGGTGGGGAAATCGGGCTTACAGATGTATTTGAGTCACTGAACTTTGAGAATGACTTCAAATGTAAGGCAATGTTTTACATACACAGATTGATGAGAGGACTCTCTATCTGCATCACCTGCCTCCTGAGTGTGTTCCAGGCTGTCACTATCAGTCCCAGTACCTCTTCGCTGGCAAAATTTAAACATAAACTAAAAAAATACATGGTCTATTCTTTCTTATTTATTTGGGCTCTCAATTTGTCATTCGGTAGCACCATTATCTTCTACGTTGGGGCTTTTACCAATGTGAGTGAGACCAACCAGATGAAGTCCACGAAATATTGCTCACTCTTCCCCATGGACTACATCTCCACAGCACTGATTTTAACAGAGACAGTCATCAGAGATGTATTTCTTGTAGGAGTTATGATGACCTCAAGTGCTTACATGGTGATTTTCTTGTTCAGACATCACAGGCAGTGCAAGCATCTTCATAGCCTCAGTCACCTGAGAGCATCCCCAGAGAAAAGGGCCACACAAACCATCTTGCTTCTGGTGGCGTTCTATGTGATCATGTACTGGGAGGATGTCATCATCTCATCCACTGCATTCCTGTCATGGATGTACCACCCAGTCATCCTGACTGTTCAGAAGTTTGTGATGAATTCCTACCCCACAATTGCTCCTTTGGTACAAATCAGTTCTGATTACAGAATAATCAATATGCTGACAACCTTGTGGTCAAAGTGTCACCAGATTTTTAAACAATGA

>Cricetulus_griseus_intact_V1R90_3

ATGAACAGAAACAACCCACTGTACAATAACAATGGCATAAGAAATGCCTTTTTCTCTCAGATTGCCTTTGGGATCTTGGCCAATACCATCCTCCTGCTCTTCCTCGTGATGACACTCTTCCAGGAGCACAGGCACAAGCCGGCCAACATGATAACTGGCCTCTTGGCTCTAAGCCACATAGTGATGCTGTTGACCATGGCCTTCATGGCTACAGACATTTTGGGGTCCCAGAGTTTTTGGGAAGACTTCACATGTAGATCAGTTATTTCCCTGTACAGGCTGATGAGGAGCGTCTCCATCTGTGCTACGTGTCACCTGAGCATCCTCCAGGCCGTCATCCTCAGCCCCAGAAGTTCCTGTTTGTCCAAGTTCAAACACAAATCCTTACATCACAACTCCTGCTGCTTTCTTTCCCTGTGGAGCTTCTATATGTCCATTAGCGGTTACATGAGCTTCATTGTTGCCACCCCCAATGTGACTTCACACATTCTTATATTGATCACTAAATCCTGCTCTCTCTGGCTTTTTAGCGGCCTCATCAGACACTTACTTTGTGTACTGGCTGTCGTCCGAGATGCCGTTCTTGTAGGGCTCATGGCGCTTTCAAGTGTGTACGTGGTGGCTGTCTTGTGCAGGCATAAGAGGCAGTCACAGTACCTTCACAGTGCCAGCATTTCCCCCAGAGCATCTCCAGAGCAGAGGGCCATCTGTATCATCCTGGTGCTATTGAGTTTCTTTGTGGTCATGTACTGTTTGGACTGCATTGCCTTCTCCTTGAGAAGTATGTGGAATAATGACCCAACTCACCACTGTGTCCAGATGTTTGTGTCCAGTGGCTATGCCACACTCAGTCCTTTGGTGTTCATTAGCACTGAACAACATATAATTAACTTTTTAAAAACCATGCAGGGTGGACAATAA

>Cricetulus_griseus_intact_V1R94

ATGCATAAGAACGGCAGACCCTACACCAATTCCCATTTAACGAACATATTTTTCTCTGAAGCTGGCATTGGAATCTTAGCCAACAGCATCCTTCTTCTCTTTCACATCCTCAAGTTCATTTGTGGGCACAGGCCCAAACCCACTGACCTGCCCATTGGTCTCTTGGCCCTAATCCACCTACTTATGCTACTGACTTCGACAGTCACAGCTACCGACATTTTTATTTCTCGGAGGGGCTGGGATGACATCACATGTAAATTCCTTATCTACTTGTACAGAATTTTTAGGGGTCTCTCCCTGTGTACCACTAGCCTATTGAGTGCCCTCCAGGCTATCATCCTCAGTCCCAGAAGCTCCTGTTTAGCAAAGTTCAAGCATAAGTCCCCCTATCACATCTTACATGCCCTTCTGCTCCTGAATGTCTTCTATATGTTGATTAGCAGTCACCTCTTAGTATCTATTGTTGCCACACCCAACCTGACCGTATATAACTTTATGTATGTTACTCAGTCCTGCTCTATTCTACCTATGAGTTTCCTCATGCAAAGTACATTTACCACACTGCTGGCCCTCAGGGAACACTTTCTTATTAGTCTCATGGTCCTCTCCAGCGGGTACATGCTCATTCTCCTGTGCAGGCACAAGAAGCAGTCTCAACATCTTCACAGCACCAGCCTTTCTCCAAAAGCACCTCCGGAGCAAAGGGCCACCCGGTCCATCCTGCTGCTTGTGGGCTTCTTTGTGCTGATGTCCATCCTGGACAGCATTATCTCCTGCTTAAGAACTATGTTCCTGAATGATCCAACATCTTACTATATCCAACTCTTTGTGGTCCATATCTATGCCACAGTCAGTCCTTTTGTATTTATGAGCACTGAAAACCATATCGGTAACTTGTTGAGGTCCACATGTGAGAGGGTGGTAAATGTTTGA

>Dipodomys_ordii_intact_ancV1R

ATGAGGTTCTCCGCAGACATGCTCGAGATGACTGCCTGCGCCATTCTCATCCTCGTCAGCTTCGTGGGAAATACGTGTCTGTTTTATTCGACGAGGCGCTGTGCCACTGGACGTCCGCAGACATCGTTTCTTCTCATTTTCAGTCTTATCCTCGTCCACCTTATGAAGAACTTGGTGGTGAACGTCATGAAAATCGTGTATTCCTCTGGGATCCTGGTGGACGAAGTCGGCTGCAAAGTCCTCCATTTCACGGCGGCCCTGACCACCTCCTTGGCCATCTGGTTCATGCTGCACTTCGCTTTGTTCTACCTCCGGAAGCTTTACCAATTGGTGCACCCCTCCAGTGAGGTTGTACCCCAGGCCCAACAGAAACATTCCCTGGTGGGGATTTCTGCCCTCTGGGTGGCTGGTGTGGCTGTATACATCCCCATTTTAATTTATACAAGAAAACCAGAATACCCAAACATGGGAAACGATACAGACAGCTTGTCTATGCCCAGGATTTACATGGATTGCCTGATTCACTTTGGAGACAAGCAGGTAGAGTTTTACTATGGGAAAATATTTCTTGTTCTAGTTGATATTCTTCCTTTAGCCCTCTTAGTCTTGGTATGTTTCTGGATGTCTTTCCTCCTCTCAGAGAAAAGGAAGATGACATACGGTGACATCTGGATTGGAGAGGATGACTCAGAAACGGAAGTCCTCCGAGGGGCCAAGTTCAGCGTTGTGTTAATGCTGCTCATCACGCCGCTGTGGGTTTCCCACTTCATCTTAGTGTATTTCTTGAAGGACGTGGCAGCCTGGGTCTGCATCCCAGCCATCCTCACCGCCCTCTCTTCGGGTTTCTCGGCTCTCAGTCCTTTCCTGTTCATGTTGGTTAATTACAAAATGAAGCTGGTGTCTCTCTGTGGTGCCAGACAGGAAAAACCTCCACCACAGCCTGCAGATGCCATTCTCTCTCCGTATGCTTGA

>Dipodomys_ordii_intact_V1R1_1

ATGGCCGAAGCTGACATTTTCATGGGGGTTGCCTTCCTCACTCAGAGTGCAGCAGGGGTCTTGGGGAACTCCTGTCTGCTTTGTTTTTACAGCAATACCCTCCTCACTGGACAGATGATGAAGCCCATTGACCCCATCTTAATCCACCTGATCTTTGCCAACCACTTGGTTGTTTTATCCACTGGGATCCCTCACACGATGGCATTATTTGGATGGAAGTTCTTTCTAGATGACGCTGGATGTAAAGTTGTTTTGTATTTATTCAGAGTAGCTAGAGGGGTTTCTCTTAATGCCACCTGCCTTCTGAATGGCTTCCAGGTCTCAAAGCTTTGCACTAGAAATGCTTGGTGGAAGAACACTACTAGATTCCCCAAGTGCTTTGGATTCTGTGGTTCCCTTTTCTGGATCTTGCAGCTCCTGATAAATGTTTATGTACCTCTAAGAGTGATTGGCCCAAGACACAAACAAAATGTCACTCTGCACATGCGTCATAGATACTGCTCCTCAGGACCACCACAACACTCTGCAAGGTTATTACATGTTGTCTTGTTGATTTCCATTGATGTCACATGCTTGGTTTTCATGATGTGGGCCAGTGGCTTCATGGTCTTTGTCCTCCACAGACACAAGCAGAGTGTCTGGCACATTCGCAGCCACATTCCCTCCCGAAGGCCTGTCCATGAGACCAGGGCCACATGCACCATCCTCACCCTTGTGAGCATGTTCGTCTCCTTCTACTGCCTCTCTGCCATTTTCACTCTTTGTGTTGGTCTAAATACAAAACCAGACCTGTGGCTGGTGGACACGTCTGTGTTCCTGGATGTGTGTTTCTCAGTCCTGAGCCCTTTTGTGCTCATCAGCAGAGACACTCGTATCACTCAGGTGTTTTATTTCTGCACTAAAAGAAAATTATCAAGCTGA

>Dipodomys_ordii_intact_V1R1_10

ATGTCTCCTGCCAGCCTGGATATTGGGATTGTCTTCTTCACTCAGACAGCAATTGGCCTTGTGGGAAATGTCTCTCTCCTCTGTCTGTACAGCTTCATTTTGCTCTCCAGACATTTCTCCAGACCCAAAGATCTGATTCTCAATCAGTTGGTGTTGGCCAACTCTATGGTTCTTTCCTCTAAAGGCATAACTCAGACGATAACTACTTTGGGATGGAACTATGTCCTGGATGATAGGAGATGTAAACTTGTCTTCTACTTCTACAGAGTGGGCACAGGTGTGTCCTTCACCACGGTCTGCCTCTTCAATGGCTTCCAGGCCATTAAACTTAACCCAAGTATTTGCAGATGGATTGCATTCAAGATTAGATCTCTAAAGTTCATTGGGTCCTGCTGTTTTCTAAGCTGGACCCTCCACCTCCTGATAAATTCCTTCCTTCATTTAATCATACATGGTCCTTTGAGCAAGAACAATCTCAGCATGGGAACGCAGCATGGTTACTGTTCCTGGGATATGCCAGAGAGATACAGCTCGCTCTACACACTCTTATATTTCTCACCTGATTTGATGAGTTTGAGTTTCATGATCTGGGCCAGCAGCTCCATGGTGCTCGTCCTGCACAGACACAGACAGAGGCTGCGGCACATTCACAGCCTGACTCGCAGGCACCACCATGAGATCAGAGCCATGCGCACCATCCTCATCCTGGCCTGTTCCTTCGTCACCTTTTACGCTTCCTACGCCTTTCTGACCGTTTGGACCACTCTGGTTGGGCACCGTGGCCAGTGGGTGGTGAACACCTCTGTGCTCGTGGCCTCGTGTTTCCCTGCACTCAGCCCCTTCGTGCTCATCCTCACCGATACCCGCATCTCCCAGTTCTTCACCAGCAGAGTGAGGAAAATCGATGTTTCTTAA

>Dipodomys_ordii_intact_V1R1_2

ATGTCAGAAGCTCAGGTGGAGGTGGGGGTTGCCTTCCTCACTCAGACTGCAGCTGGGATTCTGGGGAACTCCTGTCTCCTCTGTTTTTACAGCTACACCCTCCTCACTGGACAGAAGGTAAGGCCCACAGACCCCATCCTCAGCCTTCTGGTCTTTGCCAACAACTTGGTTGTTTTCTCCTCTGGGATCCCTCAGACAATGGCAAGGTTTGGATGGAAGTACTTTCTGGATGATGCGGGATGTAAAGTTGTTTTGTATTTATTCAGAGTAGCTAGAGGGATTTCTCTTAATGCCACCTGCCTTCTGAGTGGCTTCCAGGTCTCAAAGCTTTGCAATAGAAACACTTGGTGGAAGATGACTACTAGATTCCCCAAGTGCTTTGGATTCTGTGGTTCCCTTTTCTGGATCCTGCAGCTCCTGGTAAATGTTTATGTGCCTCTGAGAGTGATTGGCCCAAGACACAAACAAAATGTCACTCTGCACATGCATTATATATACTGCTCCCCAGGCCCGTCAGAACCCTTTGCAACGTTATTACATGTCGTTTTATTCATTGCCATTGATGTTATGTGCTTGGTTTTCATGATGTGGGCCAGTAGCTCCATGGTCCTTTTCCTGCAGAGACACAAGCAGAGGGTCTGGTATATTCACAGCCACATTCCCTCCCGAAGACCTGACCATGAGGCCAGGGCCACACACACCATCCTGACCTTGGTGAGCATGTTCATCTCTTTCTACTGCCTCTCTGCCATTTTCACTCTTTGGCTTAGTCTAAGTAGGAAGCCAGGTCTCTGGCTGATGGACACATGTCAGTTCCTGGGTGCAGGTTTCTCAGTATTGAGCCCCTATGTGCTCATCAGCAGAGACACCCGCCTCATTCAGGTGTTTTATGTCTGCATTAAATAA

>Dipodomys_ordii_intact_V1R1_3

ATGTCCAAAGCCGACATTGAAATGAGGGTTGCCTTCCTCACACAGACTGGAGCTGGAGTCCTGGGGAACTCCTGTCTCCTTTGTTTTTACAGCTACACCCTCCTCACTAGACAGAAGGTGAGGCTTACAGACCCCATCCTCTGCCATCTGGTCTTTGCAAACTACGTGGTTGTTTTATCAACTGGGATTCCTGAGACGATGGCAGGAATTGGAGGGAAGTACTTTCTCGATGATGCCGCATGTAAAGTTGCTTTGTATTTCCGCAGAGTAGCCAGAGGGGTTTCTCTTAACACCACCTGCCTTCTGAGTGGTTTCCAGGTCTCAAAGCTTTGCACTAGAAATGTTTGGTGGAAGATCACTACTACATTCCCTAACTGCTTTGGAATCTGTGGTTTCCTTTTCTGGATTCTGCAGGTCCTGGTAAATGTTTATGTGCCTCTGAGAGTAATTGGCCCAACACACAGGCAAAATGTCACTCTGCACATGCATTATAAATACTGCTCCATAGGACCAACTTCACCAAAACCATTTGTATATTTATCACAAATAGTCTTATTCCTTTCCATTGATATTATGTGCTTGGTTTTCATGATGTGGACCAGTGGCTCCATGGTCCTTGTCCTGCACAGACACAAGCAGAGGGTCCAGCACATTCACAGCCACATTGCCTCTCGAAGACCTGACCATGAGGCCAGGGCCACACGTACCATCCTTACCCTGGTGAGCATGTTCGTCTCCTTCTACTGCCTCTCTGCCATGTTCACTCTTTGTGTTGGTTTACATATGAAACCAGACCTGTGGCTGGTGGACATGTCTGTGTTCCTGGATGCATGTTTCTCAGTACTCAGCCCTTTTGTGCTCATGAGCACTGACACTCGTATCACTCAGGTGTTTCATTCCTGCATTGAATAA

>Dipodomys_ordii_intact_V1R1_4

ATGAAAAAGATGACCGAAGCCAACATGCACTTGGGGGTTGCCTTCCTCACTCAGTCTACAGCAGGGTTGTTGGGAAACTCCTGTCTCTTCTGTTTTTACAACTACACCCTCCTCACTGGACAGAATGTGAGGCCCACAGACTTCATCCTCAGCCATCTTGTCTTTGCCAATAACTTGGTTATTTTATCCAAAGGGATTCCTCAGACAATAGCAGGTTTGGGATGGAAGTACTTCCTGGATGATGCCACATGTAAAGTTCTTTTGTATTTCCACAGAGTAGCCAGAGGGGTTTCTCTTAATGCCACCTGCCTTCTGAGTGGCTTCCAGGTCTCAAAGCTTTGCACCAGAAATGCTTGTTGGAAGATCACTACTAGATTCCCCAAGTGCTTTGAATTCTGTGGTTCCCTTTTCTGGGTCCTGCAGCTCCTGGTCAATGTTTATGTGCCTTTGAGAGTGATTGGCCCAAGACACAAACAAAATGTTACTCTGCACATGCGTTATAGATACTGCTCCACAGAAGCAGCCACAACAAAACCATTAGTACAATTGTCACTGATGATTTTACTTACTTCCATTGATGTTATGTGCTTAGTTTTCATGATGTGGGCCAGTGCCTCCATGGTCCTTGTCCTGCACAGACACAAGCAGAGGGTCCGTCACATTCACAGCCGCATTCCTTCCCGAAGACAAGATCACGAGGCCAAGGCCATATGGACCATCCTTATCCTGGTGAGCATGTTTGTCTCCTTCTACTGCCTCTCTGCCATTTTCACTCTTTATATTGGTTTAAATACAAAACCAGACCTGTGGCTGGTGGACACATCTGCGTTCCTGGGTGCATGTTTCTCATTTCTGAGCCCATTTGTGCTCATCAGCAGTGACACTCGCATCACTCAGGTGTTTCTTTCCTACACTGAATAA

>Dipodomys_ordii_intact_V1R1_5

ATGGAAGAGATGGCCGGAGCCGACATTGTAATGGGGGTGGCCTTCCTCACTCAGAGTGCAGCTGGGGTCCTGGGAAACTCCTGTCTCCTTTGTTTTTACAGCTACACCCTTCTCACTGGACAGAAGGTGAGGCCCACAGACCCCATCCTCTGCCATCTGGTCTTTGCCAACAACTTGGTTGTTTTGTCTGTTGGGATCCCTCAGACCATGGAAGGTTTTGGATGGAAGTACTTCCTGGATGATGTTGGATGTAAAGTTGTTTTTTATTTCTACAGAGTAGCCAGAGGGGTTTCTCTTAATGCCACCTGCCTTCTGGGTGGCTTCCAGGTCTCAAAGCTTTGCACTAGAAATGCTTGGTGGAAGATCACTAATAAATTCCCCAAGTGCTTTGGATTCTGTGGCTCTTTTCTCTGGATCCTGCATCTCCTGGTAAATGTTTCTGTGCCTCTGAGATTGATTGGCCCAAGACCCAAACAAAATGTCACTCTCCATATGCATTATAGATACTGTGCCATAGGAGCAGTCCCACCAAAGTCATTTGTACGTTTATCACAGATGGTCTTATTCTCTTCCATTGATGTTATGTGCTTGGTTTTCATGATGTGGGCCAGTGGCTCCATGGTCTTTGTTCTGCATAGACACAAGCAGAGTGTCCACCACATTCACAGCCAGATTCTCTCCCGAAGACCTGACCATGAGGCCAGGGCCACCCGCACCATCCTGTCCCTGGTGTGCATGTTCGTCTCCTTCTACTGCCTCTCTGCCATTTTCACTCTTTGTGTTGGTCTACATACGAAACCAGACTTGTGGCTTGTGGACATGGGTGTGTTCCTGGGTGCATGTTTCTCAGTCCTTAGTCCCTTTGTGCTCATCAGCAGTGACACTCGTATAACTCAGGTGTTTCACTCCTGCAGAGAATAA

>Dipodomys_ordii_intact_V1R1_6

ATGAAAAAGATGACCGAAGCCAACGTTCACATGTGGGTTGCCTTCCTCACTCAGACTACAGCAGGGGTGCTGGGGAACTCCAGTCTCTTTTGTTTTTACAACTACACCCTCCTCACTGGACAGAAGATGAGGCTCACAGACTTCATCCTCAGCCATCTGGTCTTTGCCAACAACTTGGTTATTTTATCCTCTGGGATCCCTCAGACAATGGCAGGATTTGGATGGAAGTACTTCCTGGATGATGGTGGATGTAAAGTTCTTTTGTATTTCCACAGAGTATCCAGAGGGGTTTGTCTTAATGCTACCTGCCTTCTGAGTGGCTTCCAGGTCTCAAAGCTTTGCACTAGAAATGCTTGGTGGAAGATCACCTCTAGATTCCCCAAGGGCTGTGGATTCTGTGGTTGCCTTTTCTGGATCCTGCAGCTCCTGGTCAATGTTTATGTGCCTTTGAGAGTGATTGGCCCAAGACACAAACAAAATGTTACTCTGCACATGCGTTATAGATACTGCTCCACAGAAGCAGCCCCTACAAAACCATTTGTACGTTTGTCACAGATGATCTTACTTACTTCCATTGATGTTATGTGCTTGGTTTTCATGACTTGGGCCAGTGCTTCCATGGTTCTTGTCCTGCACAGACACAAGCAGAGGGTCCATCACATTCACAGCCACATTGCCTCCCGAAGACCAGATCATGAGGCCAGGGCCACGGGGACCATCCTTACCTTGGTGTGCATGTTCGTCTCCTTCTACTGCCTCTCTGCCATTTTCACTCTTTATGTTGGTGTAAATACAAAACCAGACCTGTGGCTGGTGGACACGACTGTGTTCCTGGGTGCATGTTTCTCATTTCTGAGTCCCTTTGTGCTCATCAGCAGTGACACTCGCATCACTCAGGTGTTTCATTCCTACACTGAATAA

>Dipodomys_ordii_intact_V1R1_7

ATGGCCAAAGCCGACATTGAAATGGGGGTTGCCTTCCTCACACAGACTGCAGCTGGAGTCCTGGGGAACTCATGTCTCCTTTGTTTTTACAGCTACACCCTCCTCACTAGACAGAAGGTGAGGCCCACAGACCCCATCCTCTGCCATCTGGTCTTTGCAAACTACGTGGTTGTTTTATCAACTGGAATTCCTCATACGATGGCAGGAATTGGATGGAACTACTTTCTTGATGATGCCGCATGTAAAGTTGCTTTGTATTTCCGCAGAGTAGCCAGAGGGGTTTCTCTTAACACCACCTGCCTTCTGAGTGGTTTCCAGGTCTCAAAGCTTTGCACTAGAAATATTTGGTGGAAGATCACTACTACATTCCCCAACTGCTTTGGAATCTGTGGTTTCCTTTTCTGGATTCTGCAGCTCCTGGTAAATGTTTATGTGCCTCTGAGAGTGACTGGCCCAACACACAGGCAAAATGTCACTCTGCACATGCATTATAGATACTGCGCCATAGAACCAACTACACCAAAACCATTTGTGCGTTTGTCACAAATAGTCTTATTCCTTTCCATTGATATTATGTGCTTGGTTTTCATGATGTGGGCCAGTGGCACCATGGTCCTTCTCCTGCACAGGCACAAGCAGAGGGTCCAGCACATTCACAGCCACATTGCCTCCCGAAGACCTGACCATGAGGCCAGGGCCACACGCATCATCCTGACCCTGGTGAGCATGTTCGTCTCCTTCTACTGCCTCTCTGCCATGTTCACTGTTTTTGTTGGTCTACATATGAAACCAGACCTGTGGCTGGTGGACATGTCTATGTTCCTGTGTGCATGTTTCTCAGTACTCAGCTCCTTTGTGCTCATCAGCACTGACAATCGTATCACTCAGGTTTTTCATTCTTGCATTGAATAA

>Dipodomys_ordii_intact_V1R1_8

ATGGCCAAAGCCGACATTGATATGGGGATTGCCTTCCTCACACAGACTGTCGCTGGGGTCCTGGGGAACTCCTATCTCCTTTGTTTTTATAGCTACACCCTCCTTACTGGACAGAAGGTGAGGCTCACAGACCCCATCCTCAGCCATCTGGTCTTTGCCAACAATGTGGTTGTTTTTTCCACTGGGATCCCTCAGACCATGGAAGGATTTGGATGGAAGTACTTCCTGGATGATGCCGCATGTAAAGTTGTTTTGTATTTCCACAGAGTAGCCAGAGGGGTTTGTCTTAATGCCACCTGCCTTCTGAGTGGCTTGCAGGTCTCAAAGCTTTGCACTAGAAATGCTTGTTGGAAGATCACTACTAGATTCCCCAACTGCTTTGGAATCTGTGGTTTCCTTTTCTGGATCCTGCAGCTCCTGGTCAATGTTTCTGTGCCTCTGAGAGTGATTGGCCCAAGACACATACATAATGCCACTGTGCTCATGCATTATAGATACTGTGCCTTAGGACCAACTACACCAAAACCATTTGTACGTTTATCACAAATAGTCTTATTCCTTTCCATTGATATTATGTGCTTGGTTTTCATGATGTGGACCAGTGGCTCCATGGTCCTTGTCCTGCATAGACACAAGCAGAGGGTCCAGCACATTCATAGCTACATTGCTTCCCGAAGAACTGACCATGAGGCCAGGGCCACACGTACCATCCTGACCCTGGTGAGCATGTTTGTCTCCTTCTACTGCCTCTCTGCCATATTCACTCTTTTTGTGGGTCTACATATGAAACCAGACCTGTGGCTGATGGACACGTCTGTGTTCCTGGGTGCATGTTTCTCAGTACTCAGCCCCTTTGTGCTCATCAGCACTGACACTCGTATCACTCAGGTTTTTCATTCCTACACTGAATAA

>Dipodomys_ordii_intact_V1R1_9

ATGGCCAAAGCCGACATTGAAATCAGGGTTGCCTTCCTCACACAGACTGGAGCTGGTGTCCTGGGGAACTCCTGTCTCCTTTGTTTTTACAGCTACACCCTCCTCACTAGACAGAAGGTGAGGCCTACAGACCCCATCCTCTGCCATCTGGTCTTTGCAAACTACGTGGTTGTTTTATCAACTGGGATTCCTGAGACGATGGCAGGAATTGGATGGAAGTTCTTTCTCGATGATGCCGCATGTAAAGTTGCTTTGTATTTCTGCAGAGTAGCCAGAGGGGTTTCTCTTAACACCACCTGCCTTCTGAGTGGTTTCCAAGTCTCAAAGCTTTGCACTAGAAATGTTTGGTGGAAGATCACTACTACATTCCCTAACTGCTTTGGAATCTGTGGTTTCCTTTTCTGGATTCTGCAGCTCCTGGTAAATGTTTATGTGCCTCTGAGAGTAATTGGCCCAACACACAGGCGAAATATCACTCTGCACATGCATTATAGATACTGCGGCATAAGACCAACTACACCAAAACCATTTGTACGTTTATCACAAGTAGTCTTATACCTTTCCATTGATATTATGTGCTTGGTTTTCATGATGTGGACCAGTGGCTCCATGGTCCTTGTCCTGCACAGACACAAACAGAGGGTCCAGCACATTCACAGCCACATTGCCTCTGGAAGACCTGACCATGAGGCCAGGGCCACACGCACCATCCTGACCCTGGTGAGCATGTTTGTCTCTTTCTACTGCCTCTCTGCCACTTTCATTCTTTGTGTTGGTTTACATATGAAACCAGACCTGTGGCTGGTGGACATGTCTGTGTTCCTGGATGCATGTTTCTCAGTACTCAGCCCTTTTGTGCTCATGAGCACTGACACTCGTATCACTCAGGTGTTTCACTCCTGCATTGAATAA

>Dipodomys_ordii_intact_V1R2

ATGGTTTTGAAGCCACTTAAGGCACTGCCTTTCATCCTTCTAACAGGACTTGGGATTATAGGAAACATCTCTGTATTTGTGAATTATATGTGCAGTTTATGGGGAGGAACTAAGAAGAAATCTATACAATTGATTCTCATCCATGTGGTTCTCACAAATATCATAATACTTCTTTCCAAAGGATTGCTATGGACAATAGCAGCTTTGGGGTTGAGATCTTTTCTAGGTGTTATGGGCTGTAGAATTTTCGTTTATCTGGGGAGGGTGTCTCGGGGTCTGTCCATCTGCACCAGCAGTCTCCTCACACTGGTCCAGGCCAGCACCATCAGCCCCAGAGGTTCTGTGTGGAGGAGGCTCAAGCCAAGGTCTGCATGGCACATCCTTCTCTTGTTGCTCTTCTTTTGGATACTCAATTCTTTCATCAGCATGAACTTGTTAAATTCCATTGGAAATACCAGCTTAGTCAATTCTCAATTTGGTGGTAGTGAGTTCTATTGTTATACTATGCTAGGAAGTCAGAAAATCAACAGCATTTTCCTCACTTTCTTGGTTATGAGAGATGCTGTGTCCCAGAGTACTATGGGTGGGGCCAGTGTCTACATGATATTTCTTCTCTACAAGCATCACCAGTGTGTTCTATACCTTCAAAACTCCCAGTTTGTCTACAAAACTCCTCCTGAAATAAAAGCTGTTCAAAGTGTTCTTCTTCTGATGCTTTCTTTTATTTTATTTTATTTGATAGATTTAGTGATTTCTTTATATTTAACTATCTCCTTCGAACATATTTCCATACTAATAAATGTTCGAGAATTTCTGACCCTTGGTTATGCAATTTTCAGTCCATTTGTTCTGATTCACAGAGATGGACATCTGGCTGAATGGTGCCAAGTTCAGTGCAGAGATTTTAAACACTGA

>Dipodomys_ordii_intact_V1R3

ATGATTTGGAGTAACCTCATCCAAAGGATAATCCTCTATATGCTTACCGGTCCTGGAATTTTAGGAAACATCCTTGTACTTGTGAAATATGTATATATTTTAATTATGGGTCTTGAGAAACAAAAACCCATATACATTATCCTCATTCACTTGGTTTTCTCAAATACACTAATTATTTGTAACATAGGGATCAAAAGTATATTAACTACAGATCTTTGTTTCATAAACTTCCTAAGTGATGTTGGTTGCAAAACTGTGCTTTATATGGAAAAACTAGCTCGAGGCCTTTCTGTCTGCAACACCTGTCTCCTCACCATGTTCCAAGTCATTACCATCAGTCCCAGAATCACCCAGTGGAGAAAGCTCAAACCACAGACTGCATGGCAAGTTCTTCCCTATCTCCTCTTCTTTTGGGTCTTGAATGCTCTGATAAGCTCCTATTTGCTCTACTTCATGACAACATTCAAAAGCATGAATAGCTCCAGAACTGAAGTCTATGTTGGGTATTGTCATATGCATCAAACTAGCCATGTGTTTAGGTGGCTTTGCCTCTCCCTCATGGCTCTCCGGGATGTAATCTTTCAGAGTCTCATGGGCTGGAGCAGTGGGTACATGGTTTTCTACCTGTATAAACATTACAAGCGAGTCCACTACCTTCATAGCTCCAAATATGAAAACAATTCCACCCCAGAAATCAGAGTGGTTCTAAGTACTCTCATTCTCATGGTCTGTTTTCTTTGTTTTAATTGGACAAATTTAATCTCCTCCATCTACCTAAGTTCTGTCATGAAACATGACTGTACAATAATAAAGATCAATGCATTTCTAGTCTTTGGGTATGCTTGTCTCAGCCCCTTTATCCTGATCATCAGGAATGTACATGTCATGGAATGTTCACTGAAAAGTGCAAAAGTTACTTTGTATATCGATTCTTCCATGAGCCAAAGGTGTAATGCATGA

>Dipodomys_ordii_intact_V1R4_1

ATGGTTTTGAAGCCACTTAAGGCACTGCCTTTCATCCTTCTAACAGGACTTGGGATTATAGGAAACATCTCTGTATTTGTGAATTATATGTGCAGTTTATGGGGAGGAACTAAGAAGAAATCTATACAATTGATTCTCATCCATGTGGTTCTCACAAATATCATAATACTTCTTTCCAAAGGATTGCCAAGGACAATAGCAGCTTTGGGGTTGAGATATTTTCTAGATGCTATGGACTGTAGAATTATCGTTAACCTGGAGAGGGTGTCTCCGGGTCTGTCCATCTGCACCAGCAGTCTCCTCACACTGGTCCAGGCCAGCACCATCAGCCCCAGAGGTTCTGTGTGGAGGAGGCTCAAGCCAAGGTCTGCATGGCACATCCTTCTCTTGTTGCTCTTCTTTTGGATACTCAATTCTTTCATCAGCATGAACTTGTTAAATTCCATTGGAAATACCAGCTTAGTCAAATCTCAATTTGGTGGTAGTGAGTTCTATTGTTATACTATGCTAGGAAGTCAGAAAATCAACAGCATTTTCCTCACTTTCATGGCTATGAGAGATGCTGTGTCCCAGAGTACTATGGGTGGGGCCAGTGTCTACATGATATTTCTTCTCTACAAGCATCACCAGTGTGTTCTATACCTTCAAAACTCCCACTTTGTCTACAAAACTCCTCCTGAAATAAAAGCTGTTCAAAGTGTTCTTCTTCTGATGCTTTCTTTTATTTTATTTTATTTGATAGATTTAGTGATTTCTTTATATTTAACTATCTCCTTCGAACATATTTCCATACTAATAAATGTTCAAGAATTTCTGACCCTTGGTTATGCAATTTTCAGTCCATTTGTTCTGATTCACAGAGATGGACATCTGGCTGAATGGTGCCAAGTTCAGTGCAGAGATTTTAAACACAAAATATCTGAGTTTCATTCATCATTTAGCTAG

>Dipodomys_ordii_intact_V1R4_10

ATGATTTTGAATGTTGTCAAGGGAACCCTCTTCCTCTTTTTAACTGGAATTGGCATTGTAGGGAACATCATTGTTTTTGTGAATTTTATACATAGATTTTGGAGAGGAGTTCAAAAGAAGTCTACACACCTTATTCTCATCCATTTGGCTTTTGCAAACACCATAATACTTCTTTCCAAAGGATTACCAATGGCAATAGCAGCTTTTGGTTTGAGACACTTCCTGGATGACATAGGCTGTAAGGTAGTTATTTGTCTAGAGAGGGTGGCCCGGGACTTTTCCATCTGCACCACCAGTCTCCTCACTGTGGTCCATGCTATCATCATCAGTCCCAGAAGCTCTCTCTGCTGGAGATTCAAGCCAAAGACTCCAAAGAAAATTCTTCCCCTGTTTCTCTTCTTTTGGATCCTCACTTCCTTGAAATGCATCAACTTATTTCAACACATCGCAAACGTCAGAGAAAACACATCAGAAGCTGCTACAACTAACATTTATTGTTCTTTTCAACCAGGAAGCCAGAAAGCCAAATGGGTAGTTCTTAGTCTAATGGCCATGCACAGTGCCATGTTTCAGGGTGTCATGGGTGGGGCCAGTGGCTACATGCTATTTCTTCTCCACAAGCATCACCAGCGTGTTCTCTACCTTCAGACCTCTCACCTCCACAAAACTCTTCCTGAAATGAGAGCTGCTCACAGTGTTCTCCTTCTGATGTTCTGTTTTCTTTTCTTCTATTGGACAGACTGTGTTCTCTATCTATTTTTAAATTCTGTCTTAGAAACTAATTCTAAAATGGTAAACATTCATGACATTTTGACCATTGGGTATGCATGTCTCAGTCCCTTTGTGCTGATTCAGAGAGATGGAGGTCTGACCTGTTGCAATTTTCTTTCAAAAAGAAAGACATTGAAAATATCAGTTGCAGTGATTTGTCAGTGA

>Dipodomys_ordii_intact_V1R4_11

ATGTGGAGTAACATCATTCAGAGGATAATCTTCCTTTCTCTTACTGGACCTGGAATTGTGGGCAACATCCTTGTATTTGTGAGACATGTGTACACTTTTTTCACAGGTCCTGACAAAAAGCCTATAGACCTCATCCTCATCCACTTGACTTTTTCTAATGGGATCATTATCTGTAGCACAGTTATCAGAGACATAGCCATACTTTTCCCTTTCAGAAGCTTTATAGGCAATACAGGTTGTAAACTTTTGATTTATCTGGGAAGAGTGGCTCGAGGCCTTTCCATCTGCACCACCTGTTTCCTCAGTGTGGTCCAGGCCATCACCATCAGTCCCAGGACCACCCAGTGGAGAAAGCTCAAACCACAGACGGCATGGCAAGTTCTTCCCTATCTCCTCTTCTTCTGGATCTTTAATTCTCTGGTGAGTTTTAACTTGCTCCACTATATCAAAGCAGCCAGTAGCACAAACAGATCTGGAATTAAAAGGCATGCTCTGTATTGTTATATGCTACCATCTACGCAAATAGTTAGGTGGCTTTTCCTCTCCCTCATGGCTCTTCGGGATGTGATCTTTCAGAGTCTCATGGCCTGCAGCAGTGGCTACATGGCTTTCTATCTGTATAAACATCACAAGCGAGTCTTCTACCTTCATAGCTCCAGGTTGGCTAACAATTCCAGTCCAGAAATCAGAGCTACTCTAAGTACTCTCATTCTCATGACCTGTTTTCTGGTTTTTTATTGGGCTGATTTCCTTTTGTCTTTTTATATAGGTTCCACTATTACAAATGACTTCAAAATACTAAATATCAAAATCATTCTAGTACTTGGTTATGCCAGTTTCAGCCCCTTAGTCTTGATCCTTAGAGATAGTCATGCTGCTAAATATTCAGACTCTCACTAG

>Dipodomys_ordii_intact_V1R4_12

ATGGGAGATACCCATGTGCCTATGGGGGTGATCCTCTTAGCCCAGACTGTGGTTGGGGTTGTGGGTAATTTGTCTCTTCTCTCCCATTATCTATTCCTTTCCTGCATGGGGAACACGATGAGAGCCACAGACTTGATTCTTACACACTTAATTGTGGCCAACCTCCTAACTCTTCTGTTTAGGGGTGTTCCTCAAACCATGGTGGCTTGGGGCTGGAGAATTTTCTTCAATGATGTGGGGTGCAAAATGGTCTTCTATCTTTACCGAGTAGGCAGGGGGATGTCGATGGGTAGCACTTGCTCCCTCAGCATCTTCCTTGCTGTCAGCATCAGCCAGGGGGACTCCAGTTGGGCAGGGCTGAGGGGGAGAGCCCACAAGCACATTGTCTCCACCGTCTACCTCAGCTGGGGGGTCTTCCTCCTGGTGAGTATTGTTTTTCCCCTGTACATGACTGGACCCAAAGCGCATGACAACATGACGAGCTTTAAGTCCTATGGATACTGTTCTTCCGTGCGACACGATCCAGCTGCTGATGTGCTGTACATGGCCATACTCTCGGTGCCTGATGTTCTGTGTCTGGGGCTCATGCTCTGGGCCAGCACCTCCATGCTTTTTGTCCTGTACAGACACAAGCAGAAAATGCGACACATCCAGAGGACCAGTGTCTCCACCAGAGCCTCTCTTGAGTCCAGAGCCATCATAAGCGTCCTCCTCCTGGTGAGCGCCTTTGTCTCCTTTTATACCGTGTCCTTCATCTGTCATATTTGCATGTCTGTGATTTACAACCCCAGCCCTGCGCTACACCATATGGGGGCGTTTGCTATGGGCTGTTTCCCAGCCCTCAGCCCTTTCCTACTCATGAGCGGACACTCCACTCCCTGCAGCCTCCTCTTCACCTGCACAAAGAAGAAAGAGGTCCTCCCCTGA

>Dipodomys_ordii_intact_V1R4_13

ATGGGAGATGCCCATGTGTTTATGGGGGTCACCTTCTTAGCCCAGACTGTGGTTGGTTTTGTGGGTAATTTGTATCTTCTCTCTCATTGTCTACTCCTTTCCTGCATGAGGAACAGGATGAGAGCCACAGACTTGATTCTTAGGCACTTAATTGTGGCCAATTTCCTAAGTCTTCTGTGTAGGAGTGTTCCCCAAACCATGTTGGCTTGGGGCTGGAGAAATTTCTTCAATGATGTGGGGTGCAAAATGGTCTTCTATCTTCACCGAGTGGGCAGAGGGGTGTCGATCAGTAGCACTTGCTCCCTGAGCATCTTCCTTGCTGTCAGAATCAGCCAGGGGGACTCCAGGTGGGCAGGGCTGAAGGGGAGAGCCCACAAGCACATTGTCTCCACTGTCTACCTCAGCTGGGGGGTTTTCCTCCTGGTGAGCATTGTTAATCTCAAGTTCGAGACTGGACCCAAAGCACATGAAAACATGACAAGCTTAAAGGCCTATGGATGTTGCTCTTCCACGCATCAGGACCCAGCTGCTGATGTGCTCTACTTGGCCATGCTCTCAGTGCCTGATGTTCTGTGTCTGGGGCTCAGGCTCTGGGCCAGCATCTCCATGCTCTTTGTCCTGTACAGACACAAGCAGAGAATGCGACACATCCAGAAGATCAGCGTCTCCACCAGAGCCTCTCTCGAGTCCAGAGCTACCATAATCCTCCTCCTGGTGAGCACCTTTTTCTCCTTATATGCCATGTCCTGCATTTTTCAGATTTGCTTGTCTGTGATTGACAACCCCAGCCCTGTGTTGCACCATATGACGGTGTTTGTTATTGGCTGTTTCCCATCCCTCAGTCCTTTCCTGCTCATGAGTGGACACTCCACTCCCTGCAGCCTCCTCCTCACCTGCACAAAGAAGAAGAAAGTGGTCCTCCCCTGA

>Dipodomys_ordii_intact_V1R4_14

ATGTGGAGTAACATCATTCAGAGGATAATCTTCCTTTCTCTTACTGGACCTGGAATTGTGGGCAACATCCTTGTATGTGTGAGACATGTGTACAGTTTTTTCACAGGTCCTGACAAAAAACCCATAGACCTCATCCTTATCCACTTGACTTTTTCTAATGTCATCATTATTTGTAGCACTGGGGTAAGAGAGATAGCCACTGTTTTTTATGTCAGAAATTTTCTAGGTGATGTGGGTTGTAAACTTGTGCTTTATCTGGAAAGAGTAGCTCGAGGGCTTTCCATCTGCACCACCTGTTTGCTCAGCATGGTCCAGGCCATCACCATCAGTTCCAGGACCACCCAATGGAGAAAGCTCAAACCTACGACTGCATGGCAAGTTCTCCCCTATCTCCTTCTCTTTTGGATTTTAAATTCTCTGATAAGCTCCAACTTGCTCCACTATATCACAGCAGTCAATAATACGAATGGATCTGGACTTGGAATGTATGTTGTGTATTGTCATATGCTACCCTCTATGCAAATAGTTAGATGGCTTTTCCTCTCCCTCATGGCTCTCCGGGATGTGATCTTTCAGAGTCTCATGGGCTGCAGCAGTGGGTACATGGCTTTCTATCTGTATAACCATCACAAGAGAGTCCTTTATCTTCATAGCTCCAGGTTTGCAAACAATTCTAGCCCAGAAATCAGAGCTACTCTAAGTACCCTTGTTCTTATGGCCTGTTTCCTTGTCTTTTATTGGGCAGATTTCATTTTTTCCTTCTATACAGGTTCCGTCGAAACCTTTGACTCTATAATAATAAATATTAAAGTATTTCTAGTTCTTGCTTATGCTAGCCTCAGCCCTTTTGTTCTGGTCATCAGAGATATTCGTGTTGCCAAATACTGGTGTTCTTTCTGA

>Dipodomys_ordii_intact_V1R4_15

ATGGGAGATGCCCATGTGTCTATGGGGGTGATCCTCTTAGCCCAGACTGTGGTTGGTTTTGTGGGTAATTTGTCTCTTCTCTCCCATTGTCTATTCCTTTCCTGCATGGGGAACAGGATGAGAGACACAGATTTGTTTCTTAGGCACTTAATTGTGGCCAATTTCCTAAGTCTTCTGTGTAGGAGTGTTCCCCAAACAATGTTGGCTTGGGGCTGGAGAAATTTCTTCAATGATGTGGGGTGCAAAATGGTCTCCTATCTTCACCGAGTGGGCAGGGGGGTGTCGATCAGTAGCACTTGCTCCCTGAGCATCTTCCTTGCTGTCAGCATCAGCCAGGGGGACTCCAGATGGGCAGGGCTGAAGGGGAGAGCCCACAAGCACATTGTCTCCACTGTCTACCTCAGCTGGGGGGTTTTCCTCCTGGTGAGCATTGTTAATCTCAAGTTCACGACCAGACCCAAAGCACACGACAACATGACGAGCTCAAAGGCCTATGGATACTGTTCTTCCATGCATCAGGTCCCAGCTTATGATGTGCTCTACGTGGTCATGGTCTCAGTGCCTGATGTTCTGTGTCTGGGACTCATGCTCTGGGCCAGCAGCTCCATGCTATTAGTCCTGTACAGACACAAGCAGAGAATGCGACACATCCAGAAGATCAGCGTCTCCACCAGAGCCTCTCTCGAGTCCAGAGCTACCATAATCATCCTCCTCCTGGTGAGTGCCTTTATCTCCTTTTATACAATGTCCTGCATCTGTCAGATTTGCTTGTCTGTGATTTACAACCCCAGCCCTGTACTGCACCATATGACGGTGTTTGTTATGGGCTGTTTCCCATCCCTCAGTCCTTTCCTGCTCATGAGTGGACACTCCACTCCCTGCAGCCTCCTCTTCACCTGCACAAAGAAGAAGAAAGTGGTCCTACCCTGA

>Dipodomys_ordii_intact_V1R4_16

ATGTTCTCGGATGCATCAATATGGGGGCTTTTCCTCATAGCCCAGCTGTGTGTTGGTGTCATGGGGAATTCCCTGCTCTCCATGCTCTATCTGTACATGTTTTTATTCCAAAGTCATCTGAAGAAGCCCATCGATGCCATTTTCATGCACCTGACTGTGGTCAACATCCTGAATCTCACCTTCACAATGTTGCCAGACATCATAGCATGCTTCGGAATCCAGCAATTTCTGAACGATGTGGGCTGCAAGGTGGTGGTGTACTTCTTAAGAGTCACCCGGGGACTGTCCATCTGTACCACCTCACTCCTCAGTGCTTTTCAGGCCATCACCATCAGCAACAGCCATGCTCAATTGGTGTGTCTTAAATCTAAGCTTTCCATGTGGATTTTCCCTTCTTTCCTCTTCTTCTGGGTCATCAATATGGTCATCTATATCCCAGTCATTACAGCTATGAAAGGCAAAAGCAATTTCACTTTTGTTGGTCCCGGGATTTCCAATGCATATTGTCAGACTCCGCAGATAGAAGACAATAGATCATGGTATTTCATCTCTATTTTATTATTTCGAGATCTTGTGTTTGTTATCCTCATGATCACCACCAGCTTCTATATGGTGACCTTCCTGTACCGACACCATAAGACAGCCCAACACCTCCACAGCCCCAGGCTTCCTTCCCAGTTGGCACCTGAGAACAAGGCCACCCACACCATCTTACTGTTAGTCAGCTGTTTTGTGTTCTTCTATTGCTCCAACAACATCGTAAACTTTTATTCCTTTTACACACCAGTGAAAATCCCAACACTGGATGTGCTGATTTGTATTTTGTCATCATGCTACCCAACCGTATGTCCTTTTTTCCTAATGAAAAATAATAAAATTTTTTCTAAATATTCACTCTCCCTCTTGATGATAGGAAGTACTGGTTGTCATAAAGCATTCAACAGCTGA

>Dipodomys_ordii_intact_V1R4_17

ATGATTTGGAGTAACTTCATCCAGAAAGTACTTCTGCTGTCTCTGGCTGGACAAGGAATTATGGGGAACATCTTTGTTCTTGTGAGAACTGTATATATTTTTTCCATGGGCCCTGAGAAAAAACACTCAGACATAGTCCTTATTCACTTGGTTTTCTCAAATACATTACTTATTTGTAGCAGAGAGATCACAGATATAGCCACAGACCTTAATTACCTAAATTTTCTAGGTGATGTGGGTTGTAAAATTGTAGTTTATCTGGGAAGAGTGGCTCAAGGGCTTTCCATCTGTACCACCTGTCTCCTCAGCATGGTCCAGGCCATCACCATCAGTCCCAGAACCACCTTATGGGAAAAGCTCAAACCAAAGGCTGCATGGCAAGTTCTTCCCTATCTGCTCCTCTTTTGGATCTCTAATTCTCTGATAAGCTCAAACCTGCCATACTACATCACAGCAGTCAGAAGCAGAAACAGCTCTCGGATTGAAATATCTTATCCATATTGTTATATGTTACCATCTAAGCAAATAATTAAATGGCTTTTCCTCTCCCTCTTGACTCTCCGGGAGGTGATCTTTCAGAGTCTCATGGGCTGGAGCAGTGGGTACATGGCTTTCTATCTGTATAACCATCACAAGCAAGTCCTCTACCTTCATAGCTCCAGGTTTACAAACAATTCCAGCCCAGAAATCAGAGCCACTCTAAGTACTCTTGTTCTCATGACCTGTTTCCTTGTCTTTTATTGGGCAGATTTCATGTTCTCCTTCTATATAGGTTCCACCTTAAAACATGATTCCATGATTATAAATATTAAAGTATTTCTAGAACTTGGTTATGCCAGTCTAAGTCCCATTGTGTTGATCAGACGAAATGTCCATCTGGTCAGATGTTGA

>Dipodomys_ordii_intact_V1R4_18

ATGACACAAGAAAGTATTTCGAATCTTGTCAAGGGCACCATCTTCCTCTTTGTAACCACCCTTGGCATTGTAGGGAACATCATTGTTTTTGTGAATTTTGTATGTAGTCTTTGGAGAGGTGCTCAGAAGAAATCTGTACATCTTATTCTCATCCACTTGGCTTTTGCAAATACTATAATACTTCTTTCCAAAGGATTGCTAAGAGCAATAACAGCTTTTGGTTTAAGAAACTTCCTGGATGATATAGGCTGTAAGATAGTTGTTTATCTGGAGAGGGTGGCCCGGGACTTTTCCATCTGCACCAGCACTCTCCTCACTGTGGTCCAGGCCATCACCATCAGTCCCAGAGGCTCTGTGTGCTGGAGATTCAAGCCAAAGACTCCAAGGAAAATTCTTCCCGTGTTTCTCTTCTTTTGGATCCTTAGTTCCTCTAGTAGCATCAACTTGCTTCATTACATCACAAGTAGCAGGAAAAACATGTCAGAAGCTGCTAAAACTAACAATTATTGTTCTTTTCAACCAGAAAGTCAGGAAATAAAATGGATACTTCTTATTCTAATGGCCATACACAGTGTCATGTTTCAGGGTATCATGGGTGGGGCCAGTGGCTACATGCTATTTCTTCTCCACAAACATCACCAGCGTGTTCTCTACCTTCAGAGCTCTCACCTTCATAAAAGTCTTCCTGAAATGAGAGCTGCTCAGAGTGTTCTCCTTCTGATGTTCTGTTTTCTTTTCTTCTATTGGACAGATTACATTTTTTCTTTCTTTTCAAATTCTTTCTTACAGAATAATTCTATGATGTTAAATGTCCAAGAATTTTTGACCATCGGTTATGCATTTCTCAGTCCCTTTGTGCTGATTCACAGAGAGGGAAATCTGGATAGTTGCAAGCCTTGGAGGAGACAAAGGCATTAA

>Dipodomys_ordii_intact_V1R4_19

ATGGGAGATGCCCATGTGTCTATGGGGGTGATCCTCTTAGCCCAGACTGTGGTTGGGTTTGTAGGTAATTTGTCTCTTCTCTCCCATTATCTGTTCCTTTCCTGTATGGGGAACAGGATGAGAGCCACAGACTTGATTCTTAGGCACTTAATTGTGGCCAACTTCCTAAGTCTTCTGTGTAGAAGTATTCCCGAAACCATGGTGGCTTGGGGCTGGAGAAATTTCTTCAATGATGTGCAGTGCAAAATTGTCTTCTTTCTTTACCGAGTGAGCAGGGGGGTGTCGACCGGAAGCACTTGCTCCCTGAGCATCTTCCTTGCTGTCAGCATCAGCCAGCGGGACTCCAGGTGGGCAGGGCTGAAGTGGAGAGACCACAAACACATTGTCTTCACCCTCTACCTCAGCTGGGGGGTCTTCCTTCTGGTGAGCATTTCTTGTCTTATGTTCATGACCGGACCCAAAGCACACGACAACATGACGAGCTTAAAGGCCTATGTATACTGTTCTTCCATGCGTCAGGACCCAGCTGCTAATGTGCTCTACGTGGCCATGCTCGCAGTGCCTGATGTTCTGTGTCTGGGGCTCATGCTCTGGGCCAGCACCGCCATGCTCTTCATCCTGTACAGACACAAGCAGAGAATGCGACACATACAGAGAATCAGCGTCTCCACCAGAGCCTCTCTCCAGTCCAGAGCCACCATAATCCTCCTCCTCCTGTTGATCACCTTTGTCTCCTTTTATACCATGCCCTGCATCTGTCAGACTTGCTTGACTGTGATTTACAACCCCAGCCCTGTGCTGCACCATATGGTGGCGTTTGGTATGGGCTGTTTCCCATCCTTCAGCCCTTTCCTGCTCATGAGCGGACACTCCACTCCCTGCAACCACCCCTTCACCTGCACAAAGAAGAAGACAGTGATCCTCCCCTGA

>Dipodomys_ordii_intact_V1R4_2

ATGATTTTGAATGTTGTCAAGGGAACCCTCTTCCTCTTTCTAACTGCAATTGGCATTGTAGGGAACATCATTGTTTTTGTGAATTTTATATATAGATTTTGGAGAGGAACTCAGAAGAAGTCTACACACCTTATTCTCATCCATTTGGCTTTTGCAAACACCATAATACTTCTTTCCAAAGGATTACCAATGGCAATAGCAGCTTTTGGTTTGAGACACTTCCTGGATGATATAGGCTGTAAGGTAGTTATTTGTCTAGAGAGGGTGGCCCGGGACTTTTCCATCTGCACCAGCAGTCTCCTCACTGTGGTCCATGCTATCATCATCAGTCCCAGAAGCTCTCTCTGCTGGAGATTCAAGCCAAAGACTCCAAAGAAAATTCTTCCCCTGTTTCTCTTCTTTTGGATCCTCACTTCCTTGAAACCCATCAACTTATTTCAACACATCGCAAACGTCAGAGAAAACATATCAGAAGCTGCTAAAACTGACAATTATTGTTCTTTTCAACCAGAAAGTCAGAAAGCCAAATGGGTAGTTCTTAGTCTAATGGCCATGCACAGTGCCATGTTTCAGGGTGTCATGGGTGGGGCCAGTGGCTACATGCTATTTCTTCTCCACAAGCATCACCAGCGTGTTCTCTACCTACAGACCTCTCATCTCCACAAAACTCTTCCTGAAATGAGAGCTGCTCACAGTGTTCTCCTTCTGATGTTCTTTTTTGTTTTCTTCTATTGGACAGATTGTGTTCTCTCTATATTTTTAAATTCTGTCTTAGAAACTAATTCTGCAATGTTAAGCATTCATGGCATTTTGACCATTGGGTATGCATGTCTCAGTCCCTTTGTGCTGATTCAGAGAGATGGAGGTCTGACCTGTTGCAATTTTTTTTCAGGAAGAAAGACATTGAAAATATCAGTTGCAGTGATTTGTCAGTGA

>Dipodomys_ordii_intact_V1R4_20

ATGAAGTCTGAAGAGGTTTGGAGTAAAGTCATCCAGAAAATAGTCTTCTTTTCTCTCACTGGACCTGGAATGGTGGGGAACATCTTTATATTTGTGTTACATGTATGTACTTTTGTCATGGAACCTGACAAAAAACCCATAAATCTTATACTTGTCCACTTGGCTTTTTCAAATATGATCATTATTTGTAGCATAGGCATAAGAGAAATAGCAATCATTGCTTATTTCAGAAATTTGCTAGATGATCATAGTTGTAAACCTATGGTTTATCTGAGGAGGATGGCTCGAGGGCTCTCCATCTGTACCACCTGTCTCCTCAGTGTGGTCCAGGCCATCACCATCAGTCCCAGGACCACCCTGTGGAGAAAACTCAAACCACAGACTGCATGGCAAGTTCTTCCCTATCTCCTCCTCTTTTGGATCTCTAATTCTCTGATAAGCTCAAACCTGCTACACTACATCACAGCAGTCAATAACATGAATGGATCTGGGTTTGGAATGTACATTGGGTATTGTTATATGCTACCATCTAGACAAGAGGTTAGATGGCTTTTTCTCTCCCTCATGACTCTCCGGGATGTGATCTTTCAGAGTTTGATGGGCTGGAGCAGTGGGTACATGGCTTTCTATCTGTATAAACATCACAAGCGAGTCCTCTACCTTCATAGCTCCAAGTTTGCAAAAAATTCCAGCTCAGAAATCAGAGTCACTCTAAGTACTCTCATTCTTATGACCTGTTTCCTTGTCTTTTATTGGGCAGATTTAATGTTCTCCTTATATGCAGGTTCTACCTTGAGAAGTGATTTCATAACACTAAACATTAAAACATTTCTAGAACTTGGATATGCTGTTCTCAGCCCCTTGGTCCTGATCAACCGGCATGTCCATATTGTTAAATCCTGGAGGCTCACTGAGAAATACAGAAATCTATGCTTCTAA

>Dipodomys_ordii_intact_V1R4_21

ATGGCAGATGCCCATGTGTCTATGGGGGTGATCCTCTTAGCCCAGACTGTGGTTGGGTTTGTGGGTAATTTGTCTCTTCTCTCCCATTATCTATTCCTTTCCTGCATGGGGAACAGGATGAGAGCCACAGACTTGATTCTTAGGCACTTAATTTTGGCCAACTTCCTATGTCTTCTGTGTAGGGGTGTTCCCCAAACCATGGTGGCTTGGGGCTGGAGAAATTTCTTCAATGATATGGGGTGCAAAATGGTCTTCTATCTTTACCGAGTGGGCAGGGGGGTGGCAATAGGTAGCACTTGCTCCCTCAGCATCTTCCAGGCTGTGAGCATCAGCCAGGGGGACTCCAGGTGGGCAGGGCTGAAGGGGAGAGCCCACAAGCACATTGTGTCCACCGTCTACTTCAGCTGGGGGGTTTTCCCCCTGGTGAGCATTGTTTTTCCCATGTACATCACTGGATGCAGAGCACACAACAACATGACGATCTTAAAAGCCTTTGGATACTGTTCTTCTGTGCGTCATGATCCAACTGAGGATGTGCTTCACATGACCATGCTCTTGGTGCCTGATGTTCTGTGTTTGGGGCTCATGCTCTGGGCCAGCACCTCCATGCTTTTTGTCCTGTACAGACACAAGCAGAAAATGCGACACATCCAGAGGACCAGTGTTTTCACCAGAGCCTCTCCCGAGTCCAGAGCTACCATAAGCATCCTCCTCCTGGTGACTGCCTTTGTCTCCTTTTACGCTATGTCCTGCATCTCTCAGATTTGCTTGTCTGTGATTTACAACCCCAGCCCTGTGCTGTACCTTATAGCGGCATTTGCTATGGGCTGTTTCCCAGCCCTCAGCCCTTTCCTGCTCATGAGCAGACACTCCACTCCCTGCAGCCTCCTCTTCACCTGCACAAAGAAGAAGAAAGTGGTCCTCCCCTGA

>Dipodomys_ordii_intact_V1R4_22

ATGTCTTTGAGATTTTTCAAGGGAACATTCTCCTTTTTTATATCTGGACTTGGCATTGCAGGGAACACCTTTATTCTTTTGTATTATATGCACATGTTTAAAGGTTCCAAGAAGAAATATGTACACATTGTATTCATCCACTTGGCTTTTACTAACATCATAATGCTTCTTTCAAAAGTGATACCAACGACACTAGCAGCCTTTGGCATGGAGAACTTCTTGGAGGATATAGGCTGTATGATTTTTGTTTACCTAGAGAGAGTGACTCGAGGTCTTTGCATTTGCACCAGCAGTCTCCTCACCCTGGTCCAGGCCATCATCATCAGCCCCAGAGGCTCTGTGTGGAGGGAGCTAAAGCTTCGATCTGTATGGCACATCCTTCTCCCATTGCTGTTCTTTTGGATTCTCAATTCCTTGATAAGCATGAACTTACTATTCCACATAAAAAATATCAGCAGTATGAACATATCACAACCTAGTGTAAGTGACACCTATTGCTATTTTGTACCAGATAACTTGATAATTAGATGGATTTTTCTTATCCTCATGGTCTTGAGGGATGCTGTGTTTCAGGGTATCATGGGTGTGGCCAGTGGCTACATGGTATTTCTTCTCCACAAGCACCACCAGCGTGTTCTCTACCTGCAGAATTCCAACTTTCTCTACAAAACTTCTCCTGAGATGAAAGCTGCTTACAGTGTTCTCCTTCTCATGCTTTGTTTTCTTTTCTTTTATTGGACAGAATGTATTCTTTCTCTATTTTCAAATGCCTTCTTAGAGAATAATTACATGCTGGAAAATATTCAAGAATATTTGAGGGTTGGTTATGCAATTCTTGGTCCCTTCTTGCTGACTCCCAAGGATAGAAATCTGACTGAATGTTGGCATTGA

>Dipodomys_ordii_intact_V1R4_23

ATGTCATTGAGTTTTTTCAAGGGAACCATCTTCCTTTCTATATCTGTACTTGGCATTGTAGGGAACATCTTTATTTTTTTAAATTATATGCACATGTTTAAAAGTACCAAGAAGAAATCTATACACATTATATTCATCCACTTAGCTTTTACAAACATCATAATGCTTCTTTTAAAAGTGATACCAAAGACACTAGCAGCCTTTGGCATGGAGAACTTCTTAGAAGATATAGGCTGTATGATTTTTGTTTACCTAGAGAGAGTGACTCGAGGACTTTCCATCTGCACCAGCAGTCTCCTCACCCTGGTCCAGGCCATCACCATCAGCCCCAGAGACTCTGTGTGGAGAAAGCTAAAGATTCAGTCTGCATGGCACATCCTACCCCTGTTGCTGTTCTTTTGGATAATAAGTTCCTTGATAAGCATGAACTTACTATATCACATAAAAAATATCAGCAGTATGAACACATCACAAATTAGTAAACAGGACAACTATTGCTATTTTGTACCAGACAACTTAATAATGAGGTGGAGTTTTCTTGCCCTCATGGTCTTGAGGGATGCTGTGTTTCAGGGTATCATGGGTGTGGCCAGTGGTTACATGGTATTTCTTCTCCACAAGCACCACCAGCGTGTTCTCTACCTGCAGAACTCCAACTTTCTCTACAAAACATCTCCTGAGATGAAAGCTGCTCACAGTGTTCTCCTTCTCATGCTTTGTTTTCTTTTCATTTATTGGACAGAATGTATTCTTTCTCTACCTTTCAATTCCTTCTTAGAGAATAATTCCATGATGAAAAATATTCAAGAATATTTGAGGATTGGTTATGCAATTCTCAGTCCTTTTATGATGATTCCCAAAGATAGCAATCTCACTGGATGTTGGCATTCACATTAG

>Dipodomys_ordii_intact_V1R4_24

ATGTTCTCGGATGCATCAATATGGGGGCTTTTCCTCATAGCCCAGCTGTGTGTTGGTGTCATGGGGAACTCCCTGCTCTCCATGCTCTATCTGTACATGTTTTTACTCCAAAGTCATCTGAAGAAGCCCATCGATGCCATTTTCATGCACCTGACTGTGGTCAACATCCTGAACCTCACCTTCACAATGTTGCCAGACATCATAGCATGCTTCGGAATCCAGCGATTTCTGAATGATGTGGGCTGCAAGGTGGTGATGTACTTCTTCAGAGTCACCCGGGGACTGTCCATCTGTACCACCTCACTCCTCAGTGCTTTTCAGGCCATCACCATCAGCTCTAGCCATGCCCAGTGGGTGTGGCTTAAATCTAAGTCTTCAGTGTGGATTTTCCCTTCTTTCCTCTTCTTCTGGGTCATCAATATGGTCATCTATGTCCCAGTCATTACAGCTATGAAAGCCAAAAGCAATTTCACTTTGGTTGGTCACGGGATCTCCAATGCATATTGTGAGACTCGGCAGATAGAAGACAATAGATCATGGTATTTCATCTCTATCTTATTATTTCGAGATCTTGTGTTTGTTATCCTCATGATCACCACCAGCTTTTATATGGTGACTCTCCTGTACAGACACCACAAGACAGCCCGGCACCTCCACAGTCCCAGGCTTGCTTCCCAGTCAGCACCTGAGAACAAGGCCACCCACACCATCTTACTGTTAGTCAGCTGTTTCGTGTTCTTCTATTGCTCCAACAATATCGCAACCTTTTATTCCTTTTACACCACAGTGAAAATCCCAAAAATGGGCGTGATCATTTGTATTTTATCATCATGCTACCCAACCATGTGTCCTTTTTTCCTAATGAAGCATAATAAAATTTTTTCAAATGTATGCTCTCCCTCTCTTTGA

>Dipodomys_ordii_intact_V1R4_25

ATGTGGAGTAACATCATTCAGAGGATAATCTTCCTTTCTCTTACTGGACCTGGAATTGTGGGCAACATCCTTGTATGTGTGAGACATATGTACACTTTTTTCACAAATCCTGACAAAAAACCCATAGACCTCATCCTCATCCACTTGACTTTTTCTAATGTCATCATTATTTGTAGCACAGGGATAAGAGAGATAGCCACTGTTTTTTATGTCAGAAATTTTCTAGGTGATGTGGGCTGTAAACTTGTGCTTTATCTGGAAAGAGTAGCTCGAGGCCTTTCCATCTGCACCACCTGTTTGCTCAGCATGGTCCAGGCCATCACCATCAGTCCCAAGACCACACAATGGAGAAAGCTCAAACCTAAGACTGCATGGCAAGTTCTCCCCTATCTCCTCCTCTTTTGGATTTTAAATTCTCTGATAAGCTCCAACTTGCTCCACTATATCACAGCAGTCAATAACACGAATGGATCTGGACTTGGAATGTATGTTGTGTATTGTCATATGCTACCATCTATGCAAATAGTTAGATGGCTTTTCCTCTCCCTCATGGCTCTCCGGGATGTGATCTTTCAGAGTCTCATGGGCTGCAGTAGTGGGTACATGGCTTTCTATCTGTATAACCATCACAAAAGAGTCCTTTATCTTCATAGCTCCAGGTTTGCAAACAATTCTAGCCCAGAAATCAGAGCTACTCTAAGTACCCTTGTTCTTATGGCCTGTTTCCTTGTCTTTTATTGGGCAGATTTCATTTTTTCCTTCTATACAGGTTCCGTCGAAACCTTTGACTCTATAATAATAAATATTAAAGTATTTCTAGTTCTTGCTTATGCTAGCCTCAGCCCTTTTGTTCTGGTCATCAGAGATATTCGTGTTGCCAAATACTGGTGTTCTTTCTGA

>Dipodomys_ordii_intact_V1R4_26

ATGATTTTAAAACTTGTCAAGGGAACTATCTTCCTCTTTCTAACTGTACTTGGCATTGTAGGGAACATCATTGTTTTTGTGAATTTTATAAATAGATTTTGGAGAGGAGCTCAGAAGACCTCCACACACCTCATTCTCATCCATTTGGCTCTTTCAAATATCACAATGCTTCTTTCCAAAGGATTACCAATGGCAATAGCAGCTTTTGGTTCGAGACACTTCCTGGATAATATAGGCTGCAAGGTAGTTGTTTATCTGGAGAGGGTGGCCCGGGACCTTTCCATCTGTACCACCACTCTCCTCACTGTGGTCCAGGCCATCACTATCAGTTCCAGAAGCTCTCTCTGCTGGAGATTCAAGCCAAAGACTCCAAAGAAAATTCTACCCCTGTTTCTCTTCTTTTGGATCCTCACTTCCTTGAAAAGCATCAGCTTACTTCATTACATTGCAAAGGTCAGAGAAAACACATCAGAAGCTGCTAAAACTAACAATTATTGTTCTTTTCAACCAGAAAGTCAGAGAACCAAATCGGTAGTTCTTATTCTAATGGCCATGCACAGTGCCATGTTTCAGGGTGTCATGGGTGGGGCCAGTGGCTACATGCTATTTCTTCTCCACAAGCATCACCAGCGTGTTCTCTACCTTCAGACCTCTCACCTCCACAAAACTCTTCCTGAAATGAGAGCTGCTCACAGTGTTCTCATTCTGATGTTCTGTTTTCTTTTCTTCTATTGGACAGATTGTGTTCTCTCTCTACTTTCAAATTCTGTCTTAGAAACTAATTCTGCAATGTTAGACATTCAAGGCATTTTAATCATTGGTTATGCATTTCTCAGTCCCTTTGTGCTGATTCAGAGAGATGGAGTTCTGACCTGTTGCCATTTTCTTTGGGACAGAAAGTCACTGAAACTATATGTGGCAGTGATTTGTTAG

>Dipodomys_ordii_intact_V1R4_27

ATGGCAGATGCCCATGTTTCCATGGGGGTGATCCTCTTAGCCCAGACTGTGGTTGGTTTTGTGGGTAATTTGTCTCTTCTTTCCCATTATCTATTCCTTTCCTGCATGGGGAACAGGATGAGAGCCACAGACTTGATTATTAGGCACTTAATTGTGGCCAACTTCCTATGTCTTCTGTGTAGGGGTGTTCCCCAAACCATGGAGGCTTTGGGCTGGAGAAATTTCTTCAATGATATGGGGTGCAAAATGGTCTTCTATCTTTACCGAGTGGGCAGGGGTGTGGCGATAGGTAGCACTTGCTCCCTCAGCATCTTCCAGGCTGTGAGCATCAGCCAGGGGGACTCCAGTTGGGCAGGGCGGAAGGGGCGAGCCCACAAGCACATTGTCTCTACTGTCTACCTCAGCTGGGGGGTTTTCCCCCTTGTGAGCATTGTTTTTCCCATGTACATCACTGGATATACAGCACATGACAACATTACGATATTAAAGGCCTATGGATACTGTTCTTCTGTGCGTCATGATCCAGCTGAGGATGTGCTTTATGTAACCATGCTCTCTGTGCCTGATGTTCTGTGTTTGGGGCTCATGGTCTGGGCCAGCGCCTCCATCCTCTTCGTTCTGTACAGACACAAGCAGAGAATGGGACACATCCAGAGGACCAGTGTCTCCACCAGAGCCTCTCCCGTGTCCAGAGCTACCACAAGCATCCTCCTCCTGTTGAGCGCCTTTGTTTCCATTTACACTATGTCCTGCATCTGTCAGATTTGCTTGTCTGTGATTTACAACCCCAGCCCTGTGCTGTGCCTTATAGCGGCATTTGCTATGGGCTGTTTCCCAGCCCTCAGCCCATTCCTGCTCATGAGCAGACACTTCACTCCCTGCAGCCTCCTCTTCACCTGCACAAAGAAGAAGAAAGTGGTCCTCCCCTGA

>Dipodomys_ordii_intact_V1R4_28

ATGATTTGGAGTAACCTCATCCAAAGGATAATCCTCATTGTGCTTACTGGTCCTGGAATTTTAGGAAACATCTTTGTACTTGTGAAATATGTATATATTTTAATTATGGGTCTTGAGAAACAAAAACCCATATACGTTATCCTCATTCACTTGGTTTTCTCAAATACACTAATTATTTGTAACATAGGGATCAAAAGTATATTGACTACAGATCTTTGTTTCATAAACTTCCTAAGTGATGTTGGTTGTAAAACTGTGCTTTATATGGAAAAACTAGCTCGAGGCCTTTCCGTCTGCACCACCTGTCTCCTCACCATGTTCCAGGTCATTACCATCAGCCCCAGAACCACCCAGTGGAGAAAGCTCAAACCACAGACTGCATGGCAAGTTCTTCCCTATCTCCTCTTCTTTTGGGTCTTGAATGCTCTAATAGGCTCCTATTTGCTCTACTTCATGACAGCATCCAATAGCATGAACAGCTCCAGAAATGAAGTCTATGTTGGGTATTGTCATATGCATCCATCTAGCCATGTATTTAGGTGGCTTTGCCTCTCCCTCATGGCTCTCCGGGATGTGATCTTTCAAAGTCTCATGGGCTGGAGCAGTGGGTACATGATTTTCTATCTGTATAAACATTACAAGCGAGTCCTCTACCTTCATAGCTCCAAATATGAAAACAATTCCACCCCAGAAATCAGAGTGGTTCTAAATACTCTCATTCTCATGGTCTGTTTTCTTTGTTTTTATTGGACAAATTTAATCTCCTCCATCTACCTAAGTTCTGTCATGAAACATGACTGTACAATAATAAAGATCAATGTATTTCTAGTCTTTGGGTATGCTTGTCTCAGCCCCTTTATCCTGATCATCAGGGATGTACATGTCACTAAATACTGGAATGTTCACTGA

>Dipodomys_ordii_intact_V1R4_29

ATGATTTTGAATGTTGTCAAGGGAACCCTCTTCCTCTTTCTAACTGCAATTGGCATTGTAGGGAACATCATTGTTTTTGTGAATTTTATACATAGATTTTGGAGAGGAGCTCAGAAGAAGTCTACACACCTTATTCTCATCCATTTGGCTTTTGCAAATACCATAATACTTCTTTCCAAAGGATTATTAATGGCAATAGCAGCTTTTGGTTTGAGATACTTGCTGGATGATATAGGCTGTAAGGTAGTTGTTTTTCTACAGAGGGTGGCCCGGGACTTTTCCATCTGTACCACCACTCTCCTCACTGTGGTCCAGGCCATTACCATCAGTCCCAGAAGCTCTCTGTGCTGGAGATTTAAGCTAAAGACTCCAAGGAAAATTCTTCCCCTGTTTCTCTTCCTTTGGATCCTCACTTCCTTGAAAAGCATCAACGTGTTTCGACACACTGCAAATGTCAGAGCAAACATATCAGAAGCTGCTAAAACTAACAATTATTGTTCTTTTCAACCAGGAAATCAGAAAGCCAAATGGGTAGTTCTTAGTCTAATGGCCATGCACAGTGCCATGTTTCAGGGTGGCATGTGTGCGGCTGGTGGCTACATGCTATTTCTTCTCCACAAGCATCACCAGCGTGTTCTCTACCTTCAGACCTGTCACCTCCACAAAACTCTTCCTGAAATGAGAGCTGCTCACAGTGTTCTCCTTCTAATGTTCTGTTTTCTTTTCTTCTATTGGGCAGATTGTGTTCTCTATCTATTTTTAAATTCTGTCTTAGAAACTAATTCTGCAATGTTAAACATTCATGGCATTTTGACCATTGGGTATGCATGTCTCAGTCCCTTTGTGCTGATTCAGAGAGATGGAGGTCTGACCTGTTGCAATTTTCTTTCAGATAGAAAGACATTGAAAATATCAGTTGCAGTGATTTGTCAGTGA

>Dipodomys_ordii_intact_V1R4_3

ATGGGAGATGCCCATGTGTTTATGGGGGTGATCCTCTTAGCCCAGACTGTGGTTGGGTTTTTGGGTAATTTGTATCTTCTCTCCAATTGTCTATTCCTTTCCTGCATGGGGAACAGGATGAGAGCCACAGATTTAATTCTTAGGCACTTAATTGTGGCCAACTTCCTAAGTCTTCTGTGTAGGGGTGTTCCGCAAACCATGGTGGCTTGGGGCTGGAGAAATTTCTTCAATGATGTGGGGTGCAAAATGATCTTCTATCTTTACCGAGTGTGCAGGGGTGTGTCGATCGGTAGCACTTGCTCCCTGAGCATCTTCATTGCTGTCAGCATCAGACAGGGGGACTCCAGGTGGGCAGGGCTGAGGTGGCGAGCCCACAAGCACATTGCCTCAAATGTCTATCTCAGCTGGGGGGTCTTCCTCTTGGTGAGCATTGTTTTTCTCATGTACATGACCGGACCCAAAGCACGCTACAACATGACAAGCTTAAACGCCTATGGATACTGTTCTTCCGTGATTCACGACCCTGCTGCTGATGTGCTGTATGTGGCCATGCTCTCGGTGCCTGATGTTCTCTGTCTGGGGCTCATGCTCTGGGCCAGCACCTCCACGCTCTTTGTTCTGTACAGACACAAACAGAGAATGGGACATATGCAGAGGACCAGTGTCTCCACCAGAGCATTTCTCGAGTCCAGAGCTACCATAACCATCTTCCTACTGGTGATTACTTTTGTCTCCTTTTACACCATGTCCTGCATCTATCAGATTTCCTTGTCTGTGATTTACAACCCCAGCCCTGTGTTGCACCATATAGGGGTGTTTGCTATGAGCTGTTTCCCATCCCTCAGCCCTTTCCTGCTCATGAGCGGACATTCCACTTCCTGTAGCCTCTTCTTCACCTGCACAAAGAAGAAGAAAGTGGTCCTCCCCTGA

>Dipodomys_ordii_intact_V1R4_30

ATGTCTTTGAGTTTTTTCAAGGCAACCATCTTCCTTTCTATATCTGGACTTGGCATTGTAGGGAACACCTTTATTCTTTTGAATTATATGCACATGTTTAAAGGTTCCAAGAAGAAATCTGTACACATTGTACTCATCCACTTGGCTTTTACTAACATCATAATGCTTCTTTCGAAAGTGATACCAACGACAGTAGCAGCCTTTGGCATGGAGAACTTCTTAGAGGATATAGGTTGTGTGATGTCTGCTTACCTAGAGAGAGTGACTCGAGGACTTTCCATCTGCACCAGCAGTCTCCTTACCCTGGTCCAGGCCATCACCATCAGCCCCAGAGAATCTTTTTGGAGAAAGTTAAAGCTTCAATCTTCATGGCATATCCTTCTCCCATTGCTGTTCTTTTGGATTCTCAATTCCTTGATAAGCATGAACTTACTATTCCACATAAAAAATATCAGCAGTATGAACATATCACAACCTAGTGTAAGTGACACCTATTGCTATTTTGTACCAGATAACTTGATAATTAGATGGATTTTTCTTATCCTCATGGTCTTGAGGGATGCTGTGTTTCAGGGTATCTTGGGTGTGGCCAGTGGCTACATGGTATTTCTTCTCCACAAGCACCACCAGCGTGTTCTCTACCTGCAGAATTCCAACTTTCTCTACAAAACTTCTCCTGAGGTGAAAGCTGTTTACAGTGTTCTCTTTCTCATGCTTTGTTTTCTTTTCTTTTACTGGACAGAATGTGCTTTTTCTCTACTTTTAAATTCCTTAATAGAGAATAATTCCATGATGAAAAATATTCAAGAATTTCTGAAAGTTGGTTATGTAATTCTCTGTCCCTTTATACTGATTTCCAGAGATGGAAATCTGGCTCAATGTTAG

>Dipodomys_ordii_intact_V1R4_31

ATGTGGAGTAACATCATTCAGAGGATAATCTTCCTTTCTCTTACTGGACCTGGAATTGTGGGCAACATCCTTGTATGTGTGAGACATATGTACACTTTTTTCACAAATCCTGACAAAAAACCTATAGACCTCATCCTCATCCACTTGACTTTTTCTAATGTCATCATTATTTGTAGCACAGGGATAAGAGAGATAGCCACTGTTTTTTATGTCAGAAATTTTCTAGGTGATGTGGGCTGTAAACTTGTGCTTTATCTGGAAAGAGTGGCTCGAGGCCTTTCTATCTGCACTACCTGTTTGCTCAGCATGGTCCAGGCCATCACCATCAGTCCCAAGACCACACAGTGGAGAAAGCTCAAACCTAAGACTGCATGGCAAGTTCTCCCCTATCTCCTCCTCTTTTGGATTTTAAATTCTCTGATAAGCTCCAACTTGCTCCACTATATCACAGCAGTCAATAACATGAATGGATCTGGACTTGGAATGTATGTTGTGTATTGTCATATGCTACCATCTATGCAAATAGTTAGATGGCTTTTCCTCTCCCTCATGGCTCTCCGGGATGTGATCTTTCAGAGTCTCATGGGCTGCAGTAGTGGGTACATGGCTTTCTATCTGTATAATCATCATAAAAGAGTCCTTTATCTTCATAGCTCCAGGTTTGCAAACAATTCTAGCCCAGAAATCAGAGCTACTCTAAGTACCCTTGTTCTTATGGCCTGTTTCCTTGTCTTTTATTGGGCAGATTTCATTTTTTCCTTCTATGTAGGTTCTGGCATAACTTTTAATTCCATCATAATAAATATTAAAGTATTTCTAGTTCTTGCTTATGCTAGCCTCAGCCCCTTTGTTCTGGTCAGCAGAGATATTCGTGTTGCCAAATACTGGTGTTCTCACTGA

>Dipodomys_ordii_intact_V1R4_32

ATGTTCTCAGGTGCATCAATATGGGGGCTTTTCCTCATAGCCCAGCTGTGTGTTGGTGTCATGGGGAACTCCCTGCTCTCCATGCTCTATCTGTACATGTTTTTATTCCAACGTCATCTGAAGAAGCCCATCGATGCCATTTTCATGCACCTGACTGTGGTCAACATCCTGAACCTCACCTTCACAATGTTACCAGACATCATAGCATGCTTCAGAATCCAGCAATTTCTGGACAATGTGGGCTGTAAAGTGGTGGTGTACCTCTTCAGAGTCACCCGGGGACTGGCCATCAGTACCACCTCACTCCTCAGTGCTTTTCAAGCCATCACCATCAGCTCTAGCCATGCCCAGTGGGTGTGGCTTAAATCTAAGTCTTCAGTGTGGATTTTCCCTTCTTTCCTCTCCTTCTGGGTCATCAATATGGTCATCTACATCCCAGTCATTACAGCTATGAAAGCCAAAAGCAATTTCACATTGGTTGGTCCCGCGATGTACAATGCATATTGTGAGACTCGGCAGATAGAAGACAATAGATCATGGTATTTCATCTCTATCTTATTATTTCGAGATCTTGTGTTTGTTATCCTCCTGATCACCACCAGCCTCTATATGGTGAGCCTCCTGTACCGACACCACAAGACAGCCCGGCACCTCCATAGTCCCAGGCTTGCTTCCCAGTCAGCACCTGAGAACAAGGCCACCCACAACATCTTCCTGTTAGTCAGCAGTTTTGTGTTCTTCTATTGCTCCCACAACATCGCAAACTTTTATTCTTTTTACACACCAGTGAAAATCCCAACACTGGATGTGATCATTTGTATTTTATCATCATGCTACCCAACCGTTTGTCCTTTTTTCCTAATGAAGAATAATAAAGTTTTTCCAAATGTAAACTCTCCTTCTCGTTGA

>Dipodomys_ordii_intact_V1R4_33

ATGTCTTTGAGTTTTTTCAAGGGAACCATCTTCCTTTCTATATCTGGACTTGGCATTGTAGGGAACACCTTTATTCTTTTGAATTATATGCACATGTTTAAAGGTTCCAAGAAGATATCTGTACACATTGTATTCATCCACTTGGCTTTTACAAACATCATAATGCTTCTTTCAAAAGTGATACCAAAGACACTAGCAGCCTTTGGCTTGGAGAACTTCTTAGAGGATATAGGTTGTGTGATGTCTGTTTACCTAGAGAGAGTGACTCGAGGACTTTCCATCTGCACCAGCAGTCTCCTCACCCTGGTCCAGGCCATCACCATCAGCCCCAGAGACTCTGTGTGGAGGAACCTAAAGCTTCAATCTACATGGCACATCCTTCTCCCATTGCTGTTCTTTTGGATTCTCAATTTCCTGATAAGCATGTACTTACTATTCCACATAAAAAATATCAGCAGTATGAACATATCACAAACTAGTGTAAGTGACACCTATTGCTATTTTGTACCAGACAACTTGATAATGAAATGGATTTTTCTTACCCTCATGGTCTTGATGGATGCTGTGTTTCAGGGTATCTTGGGTGTGGCCAGTGGCTACATGATATTTCTTCTCTACAAGCACCACCAGCGTGTTCTCTACCTGCAGAACTCCAACTTTCTCTACAAAACTTCTCCTGAGATGAAAGCTGCTTACAGTGTTCTCCTTCTCGTGCTTTGTTTTCTTTTCTTTTATTGGACAGAATGTGCTTTTTCTGTACTTTTAAGTTCCTTCTTAGAGAATAATTCCATGATGCAAAACATTCAAGAATTTCTGAAAGTTGGTTATGCCATTCTCAGTCCCTTTATACTGATTTCCAGAGATGGAAACCTGGCTCAATGTTAG

>Dipodomys_ordii_intact_V1R4_34

ATGGGAGATGCTCATGTGTTTATGGGGGTGATCCTCTTAGGCCAGACTGTGGTTGGGTTTGTGGGTAATTTATCTATTTTCTCCCATTGTCTATTCCTTTCCAGCATGGGGAAAAGGATGATACCCACAGACTTGAGTATTAGGCACTTAATTGTGGCCAACTTCCTAAGTCTTCTGTGTAGGAGTGTTCCCCAAACCATGGTAGCTTGGGGCTGGAAAAATTTCTTCAATGATGTGCAGTGCAAAATGGTCTTCTATCTTCACCGAGTGGGCAGGGGGGTGTCGATTGTTAGCACTTGCTCGCTGAGCATCTTCCTTGCTGTCAGCATCAGCCAGGGGGACTCCAGGTGGGCAAGGCTGAAGGGGAGAGCCCACAAGCACATTGTTTCCACTGTCTACCTCAGCTGGGGGGCTTTCCTCCTGGTGAGCATTGTTAATCTCAAGTTCATGACTGGACCCAAAGCATACGAGAACATGACAAGCTTTAAGGCCTATGGATACTGTTCTTCCATGCGTCAGGACCCAGCTGCTGATGTGCTGTATGTGGCCATGCTCTCGTTGCCTGATGTTCTATGTCTGGGGCTCATGCTCTGGGCCAGCACCTCCATGCTTTTTATCCTGTACAGACACAAGCAGAGAATGGGACACATCCAGAGGATCAGCGTCTCCACCAGAGCCTCTCTCGAGTCCAGAGCTACCATAAGCGTCCTCCTCCTGTTGAGGGCCTTTGTCTCCTTTTATACCATATCCTGCATCTGTCAGATTTGCTTGACTGTGATTTACAACCCTAGCCCTGTGCTGCACTGCGGCGAATGCCTTCTCGGCCAGCGAGGAAGAACGACCACCACACCAGGAGTCTTCTCAGATCACGCTTTATTGGAGAGCCTTTGA

>Dipodomys_ordii_intact_V1R4_35

ATGGGAGATGCTCATGTGTTTATGGGGTTGATCCTCTTAGGCCAGACAGTGGTTGGGTTTGTGGGTAATTTGTCTCTTTTCTCCCATTGTCTATTCCTTTCCTGCATGGGGAACAGAATGAGAGCCACAGACTTGATTATTAGGCACTTAATTGTGGCCAACTTCCTATGTCTTCTGTGTAGGAGTGTTCCCCAAACCATGGTGGCTTGGGGCTGGAGAAATTTCTTCAATGATGTGCAGTGCAAAATGGTCTTCTATCTTCACCGAGTGGGCAGGGGGGTGTCGATCAGTAGCACTTGCTCCCTGAGCATCTTCCTTGCTGTCAGCATCAGCCAAGGGCACTCCAGGTGGGCAGGGCTGAAGGGGAGAGCCCACAAGCACATTGTTTCCACTGTCTACCTCAGCTGGGGGGTTTTCCTCCTGGTGAGCATTGTTAATCTCAAGTTCATGACTGGACCCAAAGCATACGAGAACATGACAAGCTTTAAGGCCTATGGATACTGTTCTTCCGTGCGTCACGGCCCAGCTGCTGATGTGGTGTATGTGGTCATGCTCTCGTTGCCTGATGTTCTATGTCTGGGGCTCATGCTCTTGGCCAGCACCTCCATGCTTTTTATCCTGTACAGACATAAGCAGAGAATGCGACACATCCAGAGGATCAGCGTCTCCACCAGAGCCTCTCTCGAGTCCAGAGCTACCATAATCATCCTCCTCCTGTTGAGCACCTTTGTCTCCTTTTATACCATGTCCTGCATCTGTCAGACTTGCTTGTCTGTGATTTACAACCCCAGCCCTGTGCTGCACCATATCTCGGTGTTTGCTATGGGCTGCTTTCCATCCCTCTGCCCTTTCCTGCTCATGAGTGGACACTCCACTCCCTGCAGCCTCCTCTTCACCTGCACAAAGAAGAAGAAAGTGGTCCTCCCCTGA

>Dipodomys_ordii_intact_V1R4_36

ATGACAAAAGAAAGTATTTCAAATCTTATCAAGGGCACCATCTTCCTCTTTGTAACCGCAGTTGGCATTGTAGGGAACATCATTGTTTTTGTGAATTTTGTATGTAGTTTTTGGAGAGGTGCTCAGAAGAAATCTATACACCTTATTCTCATCCACTTGGCTTTTGCAAATACCATAATACTTTTTTCCAAAGGATTGCTAAGAGCAATAACAGCTTTTGGTTTAAGAAACTTCCTGGATGATACAGGCTGTAAGATAGTTGTTTATCTGGAGAGGGTGGCCCGGGACTTTTCCATCTGCACCAGCGCTCTCCTCACTGTGGTCCAGGCCATCACCATCAGTCCCAGAAGCTCTCTGTGCTGGAGATTCAAGCCAAAGACTCCATGGAAAATTCTTCCCCTGTTTCTCTTCTTTTGGATCCTCAATTCCTTGAAAATCATCAACTTACTTCATTACATCACAAATAACAGGAAAAACATGTCAGAAGCTGCTAAAACTAACAATTATTGTTCTTTTCAACCAGAAAGTCAGGAAATAAAATGGATACTTCTTATTCTAATGGCCATACACAGTGTCATGTTTCAGGGTGTCATGGGTGGGGCCAGTGGCTACATGCTATTTCTTCTCCACAAGCATCACCAGCGTGTTCTCTACCTTCAGAGCTCTCACCTTCATAAAAGTCTTCCTGAAATGAGAGCTGCTCAGAGTGTTCTCCTTCTGATGTTCTGTTTTCTTTTCTTCTATTGGACAGATTACATTTTTTCTTTCTTTTCAAATTCTTTCTTACAGAATAATTCTATGATATTAAATGTCCAAGAATTTTTGACCATCGGTTATGCATTTCTCAGTCCCTTTGTGCTGATTCACAGAGAGGGAAATCTGGATAGTTGCAAGCCTTGGAGGAGACCAAGGCATTAA

>Dipodomys_ordii_intact_V1R4_37

ATGGTTTTGGCTCTTGTTAAGGGAGCTACCTTTTTCTTTGTGACTGGACTTGGCTTTGTGGGGAACATGTTTGTTTTGGTGAATTACCTATTCATGTTTGAAGGCACGCAGAAGAAATCTATACACTTTATATTTATCCACTTGACTTTTACAAACATTATGATCCTTCTCTTAAAAGGAATACCAAAGACAATAGCCTTAGATATGGGAAACTTCTTTGATGAAATAGGCTGTAAGATCATTGTTTACTTCACAAGAATGGCTCGAGGACTTTCCATCTGCACCAGCAGTCTCCTCACCCTGGTCCAGGCCATCATCATCAGCCCCAGAGACTCTGTGTGGAGAAAGCTAAAGCTTCAATCTGGATGGCACATCCTTCTCCCATTGCTGTTCTTTTGGATTCTCAATTCCTTGATAAGCATGAACTTACTATTCCACATAAAAAATATCAGTAGGAACACATCACAAATTAGCAAACATGACAACTATTGCTATTTTGTACCAGACAACTTGATAATGAGATGGAGTTTTCTTACCCTCATGGTCTTGAGGGATGCTGTGTTTCAGGGTATCCTGGGTGTGGCCAGTGGCTACATGGTATTTCTCCTCCATAAGCACCACCAGCGTGTTCTCTACCTTCAGAACTCCAACTTTCTCTGCAAAACTCCCCCTGAAATGAAAGCTACTCAAAATGTTTTATTTCTGATGCTTTGTTTTCTTTTCTTTTATTGGACAGATTGTTTTCTTTCTTTATATCTAACTTTGTCTTTAGAGAATGAATCCACAACGCTGGCTGTTCAAGAATTTCTGTCCCTTGGTTATGCACTTTTCAGTCCCTTTGTACTCATTCCCAGAGGTGGACATCTGGCTGAGTGTTGGCAATCTCAATAA

>Dipodomys_ordii_intact_V1R4_38

ATGGGGAACAGGATGAGAGCCAAAGACTTGATTCTTAGGCACTTAATTGTAGCCAACTTCCTAAGTCTTCTGTGTAGGGGTGTTCCCCAAACCATGGTGGCTTGGGGCTGGAGAATTTTCTTCAATGATGTGGGGTGCAAAATGGTCTTCTATCTTTTCCGAGTGAGCAGGGGGGTGTCGATGGGTAGCACTTGCTCCCTGAGCCTCTTCCTTGCTGTCAGCATCAGCCAGGGGGACTCCAGCTGGGCAAGGCTGAAGGGGAGAGCCCACAAGCACATTGTCTCCACCGTCTACCTCAGCTGGGGGGTCTCCTTCCTGTTGAGCATTATGTTTCTTATGTACATGACTGGACCCAAAGTGCATGACAACATGACGAGCTTAAAGTCCTATGGATACTGTTCTTCCATGTGTCACAATCCAGCTGCTGCTGTGCTGCACATGGCCATACTCTCGGGGCCTGATATTCTGTTTCTGGGGCTCATGCTCTGGGCCAGCACCTCCATGCTTTTTGTCCTGTACAGACACAAGCAGAAAATGCGGCACATCCCAAGGACCAGTGTCTCCACCAGAGCCTCTCTCCAGTCCAGATCCATCATAAACATCCTCCTCCTGGTGAGCGCCTTTGTCTTCTTTTATACGGTGTCCTTCATCTCTCAGATTTGCTTGTCTGTGATTTACAACCCCAGCCCTGTGCTGCACCATATGGCAGCATTTGCTATGGGCTGTTTCCCAGCCCTCAGCCCTTTCCTGCTCATGAGCGGACACTCCACTCCCTGCAGTGTCCTCTTCACCTGCACAAAGAAGAAACAGGTCTTCCCCTGA

>Dipodomys_ordii_intact_V1R4_39

ATGAGAGATGCCAATGTGTTTATGGGGGTGATCCTCTTAGCCCAGACTGTGGTTGGTTTTGTGGGTAATTTGTATCTTCTCTCCCATTACCTATTCCTTTCCTGCATGGGGAACAGGATGAGAGCCACAGACTTGATTCTTAGGCACTTAATTGTGGCCAACTTCCTAAGTCTTCTGTGTAGGAGTGTTCCCCAAACCATGGTGGCTTGGGGCTGGAGAAATTTCTTCAATGATGTGGGGTGTAAAATGGTCTTCTATCTTCACCGAGTGGGCAAGGGGGTGTCAATCAGTAGCACTTGTTCCCTGAGCATCTTCCTTGCTGTCAGCATCAGCCAGGGGGAATCCAGGTGGGCAGGGCTGAAGGGGAGAGCCCAGAAGCACATTGTCTCCACTGTCGACCTCAGCTGGGGGGTTTTCTTCCTGGTGAGCATTCCTAATCTGAAGTTCACGACCGGACCCAAAGCACACGACAACATGACAAGCTTAAAGACCTATGGATACTGTTATTCTACACATCAGGACCCAGCTGCTGGTGTGCTCTACGTGGCCATGATCTCAGTGCCTGATGTTCTGTGTCTGGGGCTCATGCTCTGGGCCAGCATCTCCATGTTGTTCATCCTGTACAGACACAAGCAGAGAATGCGACACATCCAGAAGATCAGCGTCTCCACCAGAGCCTCTCTGGAGTCCAAAGCTACCATAATCATCCTCCTCCTGGTGAGCGCCTTTATCTCCTTTTATACCATGTCTTGCATTTGTCAGATTTGCTTATCTGTGATTTACAACCCCAGCCCTGTACTGCACCATATGACAGTGTTTGTTATGGGTTGTTTCCCCTCCCTTAGTCCTTTCCTGCTCATGAGTGGACACTCCACTCCCTGCAGCCTCCTCTTCACCTGCACAAAGAAGAAGAAAGTGATCCTCCCCTGA

>Dipodomys_ordii_intact_V1R4_4

ATGATTTGGAGTAACCTCATCCAAAGGATAATCCTCTATATGCTTACCGGTCCTGGAATTTTAGGAAACATCCTTGTACTTGTGAAATATGTATATATTTTAATTATGGGTCTTGAGAAACAAAAACCCATATACATTATCCTCATTCACTTGGTTTTCTCAAATACACTAATTATTTGTAACTTAGGGATCAAAAGTATATTAACTACAGATCTTTGTTTCATAAACTTCCTAAGTGATGTTGGTTGCAAAACTGTGCTTTATATGGAAAAACTAGCTCGAGGCCTTTCTGTCTGCAACACCTGTCTCCTCACCATGTTCCAGGTCATTACCATCAGTCCCAGAACCACCCAGTGGAGAAAGCTCAAACCACAGACTGCATGGCAAGTTCTTCCCTATCTCCTCTTCTTTTGGGTCTTGAATGCTCTGATAAGCTCCTATTTGCTCTACTTCATGACAGCATTCAAAAGCATGAACAGCTCCAGAACTGGGTATTGTCATATGCATCAATCTAGCCATGTATTTAGGTGGCTTTGCCTCTCCCTCATGGCTCTCCGGGATGTGATCTTTCAGAGTCTCATGGGCTGGAGCAGTGGGTACATGGTTTTCTATCTGTATAAACATTACAAGCGAGTCCACTACCTTCATAGCTCCAAATGTAAAAACAATTCCACCCCAGAAATCAGAGTGGTTCTAAGTACTCTCATTCTCATGGTCTGTTTTCTTTGTTTTTATTGGACAAATTTAATCTCCTCCATCTACCTAAGTTCTGCTATGGAACACAACTGTACAATAATAAAGATCAATGTATTTATAGTCCTTGGGTATGCTTGTTTCAGCCCCTTTATCCTGATCATCAGGGATGCACATGTCACTAAATACTGGAATGTTCACTGA

>Dipodomys_ordii_intact_V1R4_40

ATGATTTTGAATGTTGTCAAGGGAACCCTCTTCCTCTTTCTAACTGCAATTGGCATTGTAGGGAACATCATTGTTTTTGTGAATTTTATATATAGATTTTGGAGAGGAACTCAGAAGAAGTCTACACACCTTATTCTCATCCATTTGGCTTTTGCAAACACCATAATACTTCTTTCCAAAGGATTACCAATGGCAATAGCAGCTTTTGGTTTGAGATACTTGCTGGATGATATAGGCTGTAAGTTAGTTGTTTTTCTACAGAGGGTGGCCCGGGAATTTTCCATCTGTACCACCACTCTCCTCACTGTGGTCCAGGCCATCACCATCAGTCCCAGAAGCTCTCTCTGCTGGAGATTTAAGCCAAAGACTCCAAGAAAAATTCTTCCCCTGTTTCTCTTCTTTTGGATCCTCACTTCCTTGAAACGCATCAACTTATTTCAACACATCGCAAACGTCAGAGCAAACACATCAGAAGCTGCTAAAACTGACAATTATTGTTATTTTCAACCAGAAAGTCAGAAAGCCAAATGGCTAGTCCTTATTCTAATGACCATGCACAGTGCCGTGTTTCAGGGTGTCATGGGTGGGGCCGGTGGCTACATGCTATTGCTTCTCCACAAGCATCACCAGCGTGTTCTCTACCTTCAGACCTCTCACCTCCACAAAACTCTTCCTGAAATGAGAGCTGCCCACAGTGTTCTCCTTCTGATGTTCTGTTTTCTTTTCTTCTATTGGACAGACTGTGTTCTCTATATATTTTTAAATTCTGTCTTAGAAATTAATTCTGCAATGTTAAATATTCATGGCATTTTGACCATTGGGTATGCATGTCTCAGTCCCTTTGTGCTGATTCAGAGAGATGGAGGTCTGACCTGTTGCAATTTTCTTTCAAAAAGAAAGACATTGAAAATATCAGTTGCAGTGATTTGTCAGTGA

>Dipodomys_ordii_intact_V1R4_41

ATGAAGATGAACACTGTTTCCATCCATGTTATCATTCAACAATTGTTTTTATTCCAAACCAGCATTGGGATCTCTGCCAACATCTTCCTTCTTTTCTTACATCTTTTCACATTTCTCCAAGATTTCAGAGTTAAACCCACTGACATAATCAGCTGTCATTTGGCCCTTGTCCATATGGTAACGCTTCTCATTGCACTGTGTCTTTCTTCTCCAGACATTTTTGAATCATTGAAATTGCAGAGTGACTTCAAATGCAAAGTGTTGATTTATATTCACAGAGTGACTAGGGCTATTTCCATCAGTACCACCTGCCTCCTGAGTGTGTTCCAGGCCATTACTATCAGCCCCAGAACCCCCTGGTTAGTGAGATTGAAACATAAATTTACAAACTATGTTATCTGCATAGTCACTTTTTTCTGGTCCTGTAATTTATCTTTTGGTAGTATCCTCATCTTCCATACTGCAGTATATTCTAACAAAAGCCAGAAGAATCTAATAAATATCAATGAACACTGTTCAATAGATCCTATCAACCCCATCATCATGGAAGTTATTTTCATTCTGACATTGTTCAGAGATGTCTTCCTGGTAGGACTTATGCTGCTGTCCAGTGCATACATGGTGATTCTGTTGTTCAGACATCAGAGGCAGTCACAGCACCTCCACAGCACCAGGCTGAACCCAAGAGTCTCCCCAGAACAAAGGGCCACAAAGACCATTCTGTTGATGGTGAGTTTCTTTGTGATCATATACTGGGCAGACATCATCATCACTATTATCTCCTTATTATGGAAACATGACAGAATTCTGTTTAGTGTCCAGAAACTTGTGCTCAATAACTATGCCACTGTTTGTCCTATAGTACAAACCACTTCCCATAAAATTATAAAATCTACTGTGTAA

>Dipodomys_ordii_intact_V1R4_42

ATGCCATTAACAATGTTCTTAGAGGAATCAATATGGAGGCCTTTCCTCATAGCCCAGCTGTGCATCGGTATCATGGGGAACTCCCTGCTCTCCATGCTCTATCTGTACATGTTTTCATTCCAACGTCATCTGAAGAAGCCCATCAATGCCATTTTCATACATCTGACTGTGGTCAACATCCTGAACCTCACCTTCACACTTGTGCCAAATATCATGGCATCCTTTGGAGTCCAACGTTTTCTGGATGATGTGGGCTGCAAGGTGGTGGTGTACCTCTTCAGAGTCACCCGGGGTCTGTCTATCTGTACCACCTCACTCCTCAGTGCGTTTCAGGCCATCACCATCAGCTCCAGCCGTGCTCAGTGCTTGTGGCTTAAATCTAAGTCTTCAGTGTGGATTTTCCCTTCTTTCTTGTTCTTCTGGGTTGTCAATATGCTCATCTATGTCTCAGTCATTAAGGTTATGAAAGCCAAAATCAATTTCACTCTTGTTGGCTCTGGGTTTTCCAATGTATATTGTCATACTCCACAGGCTGAAGACAATAGTTCAGGGTATTATATCTCTATCTTATTATTTCGAGATCTTTTGTTTGTAATCCTCATGATCATCACCAGCCTCTACATGGTGAGGCTTCTCTACCAACACCACCAGATAGCTCAGCACCTACATAGTTTTAGCCTTGCCACCCAGCCAGCACCCAAGAGCAAGGCCACCCACACCATCCTGCTGTTAGTCAGCTGTTTCATGTGTTTCTATTGCTTCAACAACATCACCAACTTCTACTACTTTTACACAGCAGTGAAAATCCCTAGCCTGGAGGTAATGGTTTGTATTCTATCAGCATGCTACCCAACCGTGTCCCCCTTTTTCCTCATGAAGAAAAATGAAATGGTTTCCCCATGTGCCCTGTCTCTTTCTTGGAGAACTATCAGTTGTCACAAAGCATTCAGTGGATAA

>Dipodomys_ordii_intact_V1R4_43

ATGATTTTGAATGTTGTCAAGGGAACCTTCTTCCTCTTTCTAACTGCAATTGGCATTGTAGGGAACATCATTGTTTTTGTGAATTTTATATATAGATTTTGGAGAGGAACTCAGAAGAAGTCTACACACCTTATTCTCATCCATTTGGCTTTTGCAAACACCATAATACTTCTTTCCAAAGGATTACCAATGGCAATAGCAGCTTTTGGTTTGAGATACTTGCTGGATGATATAGGCTGTAAGGTAGTTGTTTTTCTACAGAGGGTGGCCCGGGAATTTTCCATCTGTACCACCACTCTCCTCACTGTGGTCCAGGCTATTACCATCAGTCCCAGAAGCTCTCTCTGCTGGAGATTTAAGCCAAAGACTCCAAGAAAAATTCTTCCCCTGTTTCTCTTCTTTTGGATCCTCACTTCCTTGAAACCCATCAACTTATTTCAACACATCGCAAACGTCAGAGCAAACACATCAGAAGCTGCTAAAACTGACAATTATTGTTATTTTCAACCAGAAAGTCAGAAAGCCAAATGGCTACTCCTTATTCTAATGGCCATGCACAGTGCCATGTTTCAGGGTGTCATGGGTGGGGCCGGTGGCTACATGCTATTTCTTCTCCACAAGCATCACCAGCGTGTTCTCTACCTTCAGATATCTCACCTTCACAAAACTCTTCCTGAAATGAGAGCTGCCCACAGTGTTCTCCTTCTGATGTTATGTTTTCTTTTCTTCTATTGGACAGACTGTGTTCTCTATCTATTTTTAAATTCTGTCTTAGAAACTAATTCTGCAATGTTAAATATTCATGGCATTTTGACCATTGGGTATGCAAGTCTCAGTCCCTTTGTGCTGATTCAGAGAGATGGAGGTCTGACCTGTTGCAATTTTCTTTCAAAAAGAAAGACATTGAAAATATCAGTTGCAGTGATTTGTCAGTGA

>Dipodomys_ordii_intact_V1R4_44

ATGGCTGTAATTGCCATCTTGCCTTTTCAGATTATAGTTGGGACTCTGGCCAATGTCCTTCTCTTCTTCTACAATGTGTCTAGACCGAGGCCCACACAAGTGATTTTCTGTCACTTGGCTGTGGCCAATGTCTTACTTCTCACCACCGGGATTCCTCTCATCATGGTGGCTTTCGTTTCAAGACATCCCCGGTCTAGTCTTGGATGTCAGGTGGTCTACTACATTAACCATGTTGCTCACAGTGCCGTGCTGTGCTCCACCTGTGTTCTGAGCGCCTATCAGGCATTCACACTCATCCCTGGGAGTGGAAGAAGGGTGGTTATGATAGGGAGACAAGTCCTGGGGTCCACTGGTCATACCTGCTGTGCCTGCTGGGTGTACAGCACCCTAATAAGCACCTACCTTCCCAGTAGACTCACAGGTCCACAGTACAAGCACAACCACACCCATCCCCACAACCAGTGGTTCTGCTCATCCTTAGGCAGTACTGTGGGCTTTGTTTTCTTGTTGTTTGTCTCTAATGCCATATTTATTGGCCTTGTGGGCTGGGCCAGTGGGTCCGTGATTCTTCTCCTGCATAGACACCACAAGAGACAGCAGCAGATTCACACCTCCAGCCACTGTCACAGAGGCTCCCCAGAGACCAGGGCAGCTTACACTGTCTTGATGCTGGTTGTCACCTTCGTCTTTTTTCATGTGCTTAACTCTGCTTTTACTTCTTATATCACTTCTTATATGAGCTTTCGTCTGTGGGTGATGTATGTGTCTTACATTTTGAATTCGTGTTTTCCTACTTTCTCTCCTATGCTTCTCCTCCTCCGAGACCCTAGAATGCCTAGTTTGAGCTTTTGA

>Dipodomys_ordii_intact_V1R4_5

ATGGTTTGGAAGCTTCTCAGGCCACTGCTTTCCCTTCTTCTAACAGGACTTGGACTTTTAGGGAATATCTCTGTTTTTGTGAATTATATGTGCAGTTTAGGGGGAGGTGATGAGAAGAAGTCTGTACAATTGATCCTCATCTCTGTGGTTTTGACAAACATTATACTGCTGCTGTCCAAAGGATTGCCATGGACAATAGCAGCTTTGGGGTGGAAAACTTTTCTAGGTGTTTTAGGCTGTACAATTATCGTTTACCTGGAGAGGTTGTCTCGGGGTCTGTCCATTTGCACCAGCAGTCTCCTCACAGTGGTCCAGGCCAGCACCATCAGCCCCAGAAGTTCTGTGTGGAGGAGGCTCAAACCAAGGTCAGCATGGCACATCCTTCCCGTGTTGCTCTTCTTTTGGATACTCAATTCCTTCATTAGCATGAGCTTGTTAAATGTTATCATAATAACCAGCATGAATACATCTTACTTTATTAAGACAGACCTCTATTGTTTTACTGTACCAAGAAGTCAGAAAATAAGCAATATTTTTCTCACTTTCATGGCTGTGAGAGATGCTGTGTCTCTGGGGATCATGGCTGTGGCCAGTGGCTACATGGTATTTCTTCTCTACAAACACCACCAGCATGTTTTATATCTCCAAAACTCCAAGCTTCTCTATAAAACTCCTCCTGAGATGAAAGCTTCTCAAAGGGTTCTCCAACTAATGTTATGTTTCCTTTTCTTTTATCTGACAGATTGCTGGCTGTCTGTATATTTAATTGTCGCCACAGACCCTGTTTTCATACTAGCACTTGTTCAAGAATTTGTAACCCTTGGTTTTGCATTTCTCAGTCCGTTTTTACTGATTCACAGAAATGGATATCTGAATGAATGTTGGCGTGCTCGTTGA

>Dipodomys_ordii_intact_V1R4_6

ATGGGAGATGCCCATGTGTCTATGGGGGTGATCCTCTTAGCCCAGACTGTGGTTGGGTTTGTGGGTAATTTGTCTCTTCTCTCCCATTATCTATTCCTTTCCTGCATGGGGAACAGGATGAGAGCCACAGACTTGATTCTTAGGCACTTAATTGTGGCCAACTTCCTAAGTCTTCTGTGTAGGGGTGTTCCCCAAACCATGGTGGCTTGGGGCTGGAGAAATTTCTTCAATGATATGGGGTGCAAAATGGTCTTCTATCTTTACCGAGTGGGCAGGGGGGTGTCGATCGGTAGCACTTGCTCCCTCAGCATCTTCCAGGCTGTCAGCATCAGCCAGAGGGACTCCAAGTGGGCAGGGCTGAAGGAGAGAGCCCACAAGCACATTGTCTCCACCGTCTACCTCAGCTGGGGGGTCTTCCTCCTGGTGAGTATTGTTTTTCCCGTGTACATGACTAGACCCAAAGAGAACGATAACATGACGATCTTAAAGGCCTATGGATACTGTTCTTCCGTGCGTCACGATCTAGCTGTTGATGTGCTTTACGTGACCATGCTCTCAGTGCCTGATGTTCTGTGTTTGGGGCTCATGCTCTGGGCCAGCACTTCCATGCTTTTTGTCCTGTACAGACACAAGCAGAGAATGCGACACATACAGAGAACCAGTGTCTCCACTAGAGCCTCTCCCGAGTCCAGAGCTACCATAAGCATCCTCCTCCTGGTGAGCGCCTTTGTCTCCTTTTACACTGTGTCCTGCATCTGTCAGATTTGCTTGTCTGTGATTTACAACCCCAGCCCTGTGTTGCACCATATGGCGGCATTTGCTATGGGCTGTTTCCCAGCCCTCAGCCCTTTCCTGCTCATGAGCAGACACTCCACTCACTGCAGCCTCCTCTTCACCTGCACAAAGAAGAAGAAAGTGGTCCTCCCCTGA

>Dipodomys_ordii_intact_V1R4_7

ATGCTCTATCTATACATGTTTTCATTCCAGCGTCATCTGAAGAAGCCCATCAATGCCATATTCATACATTTGACTGTGGTCAACATCCTGAACATCACCTTCACACTTGTGCCAAATATCATGGCATCCTTTGGAGTACAACGTTTTCTGGACGATGTGGGCTGCAAGGCAGTGGTGTGCCTCTTCAGAGTCACCCAGGATCTGTCCATCTGTACCACCTCACTCCTCAGTGTGTTTCAGGACATCACCATCAGCTCCAGTCGTGCCCAGTGCGTGAGGCTTAAATCTAAGTCTTCAGTGTGGATTTTCCCTTCTTTCTTGTTCTTCTGGGTTGTCAATATGCTCATCTATGCCCCAGTAGTTAAGGTTATGAAAGCCAAAATCAATTTCATTCTCATTGGTTCTGAGTTTTCCAATGAATATGGTCATACTCCTCAGCTTGAAGACAATAGATCAGGGTATTATATGTCTATCTTATTATTTCCAGATCTTGTGTTTATAATCCTCATGATCATCACCAGCCTCTACATGATGAGGCTTCTCTACCAACACCACCAGATAGCTCAGCACCTACATAGTTTTAGCCTTGCCACCCAGCCAGCACCCAAGAGAAAGGCCACCCACACCATCCGGCTGTTGGTCAGCTGTTTCCTGTGTTTCTATTGCTTCAACAACATCACCAATTTCTATTACTTTTACACAGCAGTGAAAATCCCTACCTTGGAGGTGATGGTTTGTATTCTAGCAGCATGCTACCCGACCGTGTCCCCTTTTTTCCTCATGAAGAATAATAATATGCTTTCCTCATGTGCCCTGTCTCTTTCATGGAGAACTATCAGTTGTTACAAAGCATTCAGTGGCTGA

>Dipodomys_ordii_intact_V1R4_8

ATGGTTTTGAAATCCATTAAGGGGATAATTTTCCTCTTTGAAACTGCATTTGGCATTGTGGGGAACATTCTTGTTTCTGTGAATTACATGTGGTGTTTCCAAAAACCCACTGAGAAAAAATATATATACCCTATTCTCATCCATTTGGCTTTGGCAAATATCATAACACTTCTTTCAAAAGGGATGCCAAAAACAATAGCAGCTTTTGGTATGCAAAATTTCCTAGATGACATAGGCTGTAAGATGATTGCTTTCCTGGAAAGAATGGCCCATGGCCTTTCTGTCTGTACCAGCAGTCACCTCACAGTGATTCAAGCCATCTCGGTCAGTCCTAATGCCTCCAAGTGGAAAAGGTTCAAGCCAAGGTCTGCATGGCACATCTTCCCATTTATTTTCTTCTTTTGGATACTCAATTCCTTAGTATGTATTAACTTGCTCTATTCTGTCACAAGTAAAAGTCTGAACATATCACAACCTAACAATACTTATTATAGTTGCTATTTTCTACCCGACAACTTAATAAGGAGATGGATCTTTCTTACTTTCATGGTCTTGAGAGATGCTGTGTTTCAGGGTATCATGGGTGCAGCCAGCGGCTACATGGTATTTCTTCTCTACAAGCACCACCAGCGTGTTCTCTACCTTCACAATTCCAAGTTTCTCTACAAAACTCCCCCTGAGATGAAAGCTGCTGAAAGTGTTCTCCTCCTGATGTTCTGTTTTCTTTTCTTTTATTGGATAGATTGTCTTTTATCTGTCTTTTTGAGTTTTTCCCTAAAGAATAACTTCATGTTAAATACTCGAGAATTTCTGACTCTTGGTTACGCCATCTTCAGCCCAGTTGTACTAATTCACAGAGATGGCCATCTGGGTAAAGATTTGTATGCTCAGTGGAAGAGAAAGACATGGAGAGCATGTTTGTCTCCTTAA

>Dipodomys_ordii_intact_V1R4_9

ATGAATTGGAATAACTTCATTCAAAGAATAATCTTCTTTTTGCTTAATGTCCCTGGCATTATGGGGAACATTCTTATATTTGTAAGATATGTATATGTTTTTGTCACAGGTCCTGACAAAAAATCCATAGACCTTATCCTCATCCACTTGATGTTTTCAAATGCAATCATTATCTGTAGCTCAGGTCTCAGAGATATAGCCACTGTTTTTTATTTCAGAAATTTTGTGGATAATATTGGTTGTAAACTTTTGATTTATCTGGGAAGAGTGGCTCGGGGGCTTTCCATCTGCACCACCTGTCTCCTCAGTGTGGTCCAGGCCATCACCATCAGTCCCAGGACCACCCTGTGGAGAAAACTCAAACCACAGACTGCATGGCAAGTTCTTCCCTATCTCCTCCTCTTTTGGATCTCTAATTCTCTGATAAGCTCAAACCTGATACACTACATCACAGCAGTCAATAACATGAATGGATCTGGGTTTGGAATGTACATTGGATATTGTTATATGCTACCATCTAGACAAGAGATTAGATGGCTTTTCCTCTCCCTCATGACTCTCCGGGATGTGATCTTTCAGAGTCTCATGGGCTGGAGCAGTGGGTACATGGCTTTCTATCTGTATAAACATCACAAGAGAGTCCTTTATCTTCATAGCTCCAGGTTTACAAACAATTCCAGCCCAGAAATCAGAGCCACTCTAAGTACTCTTGTTCTCATGACCTGTTTCCTTGTCTTTTATTGGGCAGATTTCATGTTCTCCTTATATACAGGTTCTACCTTGAGAAGTGATTTCCTAACACAAAACATTAAAACATTTCTAGAACTTGGATATGCTGTTCTCAGCCCCTTGGTCCTGATCAGTCGGGATGTCCATATTACTAAATCCCGGAGGGCTCACTGA

>Dipodomys_ordii_intact_V1R41

ATGTTCTCGGGTGCATCAATATGGGAGCTTTTCCTCATAGCCCAGCTGTGTGTTGGTGTCATGGGGAACTCCCTGCTCTTCGTGCTCCATCTGTATACATTTTTATTCCAAAGTCATCTGAAAAATCCCATCAATGCCATCTTCATGCACCTGACTGTGGTCAACATCTTGAACCTCACCTTCACAATGTTGCCAGACATCATGGCATACTTCAGATTTGAGCGATTTCTGGACGATGTGGGCTGCAAGGCAGTGGTGTACCTCTACAGAGTCACCCGGGGACTGTCCATCTGTACCACCTCACTCCTCAGTGTGTTTCAGGCCATCACAATCAGCTCCAACTGTGCCCACTGGGTGTGGCTTAAATCTAAGTCTTCAGTGTGGATTTTCTCTTCTTTCCTCTTCTTCTATATCATCAATATGGTCATCTATTTCCCAATCACTGAGAGTCTGAGAGCCAAAAGCAATTTCACACTTGTAGGTTCTGGGTTTTCCAATGCATATTGTCAGACTCCGCGGATAGAAAACAATAGATCATGGTATTTCATCTCTATCTTATTATTTCGAGATCTTGTGTTTGTAATCCTCATGATCACCACCAGCCTCTATATGGTGACCCTCCTGTACCGACACCATAAGACAGCCCGGCACCTCCACAGTCCCAGGCTTGCTTCCCAGTCAGCACCTGAGAACAAAGCCATCCACACCATCTTACTGTTAGTCAGCTGTTTCGTGTTCTTTTATTGCTTCAACAACATCGCAAACTTTTATTCCTTTCACACAGCAGTGAAAATACCAACACTGGATGTGATCATTTGTATTGTATCATCATGCTACCCAACCGTGTGTCCTTTTTTCCTAATGAAGAATAATAAAATTCTTTCCAAATGTAAACTCTCCCTCTCGTTGATAGGAAGTACCGGTTGTCATAAAGCATTCAACGGCTGA

>Dipodomys_ordii_intact_V1R46

ATGGTTTGGAGGCTTCTCAAGCCACTGCTTTCCCTTCTTCTAACAGGACTTGGACTTTTAGGGAATCTCTCTGTTTTTGTGAATTATATGTGCAGTTTAGGGGGAGGTAATGAGAAGAAGTCTGTACAATTGATCCTCATCTCTGTGGTTTTGACAAACATTATACTGCTGCTGTCCAAAGGATTGCCATGGACAATAGTAGCTTTGGGGTTGAAATCTTTTCTAGGTGTTTTAGGCTGTACAATTATCGTTTACCTGGAGAGGTTGTCTCGGGGTCTGTCCATTTGCACCAGCAGTCTCCTCACAGTGGTCCAGGCCAGCACCATCAGCCCCAGAAGTTCTGTGTGGAGGAGGCTCAAGCCAAGGTCTGCATGGCACATCCTTCCCGTGTTGCTCTTCTTTTGGATACTCAATTCCTTCATTAGCATGAGCTTGTTAAATGTTATCATAATAACCAGCATGAATACATCTTACTTTATTAAGACTGACCTCTATTGTTTTTCTGTACCAAGAAGTCAGAAAATAAGCAATATTTTTCTCACTTTCATGGCTGTGAGAGATGCTGTGTCTCTGGGGATCATGGCTGTGGCCAGTGGTTACATGGTATTTCTTCTCTACAAACACCACCAGCATGTTTTATATCTTCAAACCTCCAAGCTTCTCTACAAAACTCCTCCTGAGATGAAAGCTGCTCAAAGGGTTCTCCAACTAATGTTTTGTTTCCTTTTCTTTTATCTGACAGATTGCTTGCTGTCTTTATATTTAATTGTCTCCACAGAGCCTGTTTTCATACTAGTACATGTTCAAGAATTTGTAACCCTTGGTTTTTCATTTCTCAGTCCGTTTTTACTGATTCACAGAAATGGATATCTGAGTGAATGTTGGCGGGCTCGTTGA

>Dipodomys_ordii_intact_V1R48

ATGCACAGAAACATCAACATAAGAAACACCTTTTTCTCTGAAGTTGGCTTTGGCATTTCAGCCAATGTCATCCTTCTCCTCTTCCACTTCCTCATATTTGTTCTCCGACTCAAGCCCAAATCCACTGACCTGCCTATTGGTCTCTTGGCCCTTGTACACTTAGCAATGCTGATAACCGCAGGGTACATAGCTATGGACATTTTTGGGTACAAGAATGATTGGGATGACATCACGTGTAAATGTCTTATCCTCATATACAGGTTGGTCAGGGGCCTGTCTGTCTGCCTCACCTGCCTGCTGAGTATCCTCCAGGCCATCACCCTCAGCCCCAGAAGCTCCTGTTTGGCAAAGTTCAAACATAAATCCCTACACCACAGCCTGTGTCTCCTCCTCCTCCTGTGGGTCTTCTATATATCCATTAGCAGTCACCTCTTAATCTCTATTATTGCTACCCCCAATCTGACCTCAGAAAATCTTATGTATGTCTCTGAGTCTTGCTCTCTTTCACCCTTGCCTCACCCCTACCAGCACGTATTTTCCAAACTGCTGACCTTCAGGGAAGTCTTGTGTATGGGGCTCATGGCTTTCGCCAGTGGGTACATGGTGCTTTTCTTATGCAGGCACAAGAGGCGTTCCCGGCATCTTCATAGGACCAGCCTCACCTCAAGGACTTCCCCAGAGCAAAGGGCCACCTGGACCATTCTGCTGCTCCTGAGTTTCTTTATGGCCATGTCCATTTTGGATAGCCTCGTCTCCTACTCAAGACTTACATTAAATGATGATCCAATATTTTATTGTATCCAGCTTCTCGAGGCCCATAGCTATGCGACAGTCAGCCCCCTGGTGTTCATCAGTATCGAAAAGCGTATAATTATCGTTTTGAGATCCATATGTAAGGAGAGAGTAAGTAGTTGA

>Dipodomys_ordii_intact_V1R90_1

ATGACAGCTACCCCAGGTGACACTGTGTTCAGGCTCCTCTTTGTGTCTAAGGTGTGTCTGGGTGTCCTGGGGAACTGTATCGTCTTCCTGCTGTACGCATACAGCTCTGTGTGCAAACCTCGGCTCCGGAAGCCCATTGCAATAGTTTTCATGCACCTGACCCTGGTCAACGCCTTGACCATCATTTTTGAGTCCATGCCCTTCATCGTCTCCTCGTATGGAGTCCTGTGTTTCTGGGATGATGCCATGTGTAAGGCAGTGCTGTTCCTGTTCAGGGTCATGCGGGGACTGTCCACCTGCACTACCACCTTCATGAGTGCTTTCCAGGCCCTCACCATCAGCCGCACCCCTGCCCAGAGGGCTTGGCTGAAAAGTCGACCCTCCACCTGCATCCTTCTTTCGCTGCTCTCCTTATGGATCTTGAATCCCATCATGTATTTCGAAATCATCACAAATTTGCAATCCAATTGCAATTCAACCATCATGAGCCTGGGATTTTCTCATCCCTACTGTCAAACGAACTTCATAAGGGCCACCTCAGTGCCCATTATTGGCTATTTTGGGCTGCTAGATGTTCTGTCCCTGGTCCTCATGGTGGCCCCCAGCCTCTACATGGTGACGCTTCTCTACCGACACCGCCGCAGAGCCCAGCACCTCCACAGTCCCAGCCTTGCCTCCCAGACAGCGCCTGAGAACACAGCCACCCACACCATCCTGATGCTGGTCAGCTGCTTCGTGTTCTTCTATTGCTCCAACAACATTGTGATTATGTATTCCTTGTGGACGCTGGAGAAATTGAACAAATCCAAGGGTGTGCTTCTGATGTTGTCCTTCAGCTACCCCACCCTCTGCCCGTTCTTGCTCATGAAGAATAACAGAATGGTTTCACGATGGTTCACTCACTTATGTTATACCAAGAATGATCATTTTAATAGGAAGTGA

>Dipodomys_ordii_intact_V1R90_2

ATGACAGCCACCCCAGGTGACACTGTGTTCAGGCTCCTCTTTGTGTCTAAGGTGTGTCTGGGTGTCCTGGGGAACTGTATCCTCTTCCTGCTGTATGCATACAGCTTCTTGTGCAAACCCCGGCTCCGGAAGCCCATTGCCATAGTTTTCATGCACCTGACCCTGGTCAACGCCTTGAGCATCATTTTTGAGTCCATGCCCTTCATCGTCTCCTCGTTTGGAGTCCTGTGTTTCTGGGATGATGCCATGTGTAAGGCTGTGTTGTTCCTGTTCAGGGTCACCCGGGGACTGTCCACCTGCACTACCACCTTCATGAGTGCCTTCCAGGCCCTCAACATCAGCCGCACCCCTGCCCAGTGGGCTTGGCTGAAAAGCCGATCCTCCACGTGCATCCTCCCCTCACTGCTCTCCTTCTGGATCTTCAGTTCCATCATGTATTTCCACATGATTGAATCTGTGCAATCCAATTGCAATTCAACCGTCATGAGACTGGGATTTTCTCATCCCTACTGTCAAGTGAAGTTTGGAATGGACAACCAAATGTCCCTTATCAGCTCCATTGTGCTGCGAGATACTCTGTTCTTGGTCCTCATGGTGGCTCCCAGCCTCTACATGGTGACGCTCCTCTACCGACACCGCCGCAGAGCCCGGCACCTCCACAGTCCCAGTCTTGCCTCCCAGCCGGCACCCGAGAACACAGCCACCCACACCATCCTGCTGCTGGTCAGCTGCTTCGTGTTCTTCTATTGCTCCAACAACATCGTGACTGTGTATTCCTTCTACACACCGGAGAAATTGAACAAATCCAAGGCTGTGCTTGTGATGTTGTCCTTCAGCTACCCCACCCTCTGCCCATTCTTGCTCATGAAGAATAACAGAATGGTTTCAAAATGGTTCACTCACTTATGTTATACCAAGAATGATCCATTGTAA

>Dipodomys_ordii_intact_V1R90_3

ATGACAACTACCCCAGGTGACACTGTTTTCAGGCTCCTCTTTGTGTCTAAGGTGTGTCTGGGCATCCTGGGGAACTGTATCGTCTTCCTGCTGTACGCGTACAGCTCTGTGTGCAAATCACGGCTCCGGAAGCCCATTGCCATAGTTTTCATGCATCTGACCCTGGTCAACGCCTTGACCATCATTTTTGAGTCCATGCCCTTCATCGTCTCCTCGTTTGGAGTCCTGTGTTTCTGGGATGATGCCACATGTAAAGCGATGCTGTTCCTGTTCAGGGTCATGCGGGGACTGTCCACCTGCACTACCACCTTCATGAGTGCTTTCCAGGCCCTCACCATCAGCCGCACCCCTGCCCAGAGGGCTTGGCTGAAAAGTCGACCCTCCACCTGCATCCTTCTTTCGCTGCTCTCCTTATGGATCTTGAATCCCATCGTGTATTTTGAAATCATCACAAATGTGCAATCAAATTGCAATTCCACCATCATGAGCATGGGATATTCTCATCCCTACTGTCAAACGAACTTCATTAGGGCCACCCCAATGCCCATTATCAGCTCTTTTGTGCTGCTAGATGCTCTGTTCGTGGTCCTCATGGTGGCCCCCAGCCTCTACATGGTGACACTCCTCTACCGACACCGCCGCAGAGCCCAGCACCTCCACAGTCCCATCCTTGACTCCCAGACAGCGCCCGAGAACACAGCCACTCACACAATCCTGCTGCTGGTCAGCTGCTTCGTGTTCTTCTATTGTTCCAACAACATCGTGATTATGTATTCCTTGTGGACGCTGGAGAAATTGAACAAATCCAAGGGTGTGCTTCTGATGTTGTCCTTCAGCTACCCCACTCTATGCCCGTTCTTACTCATGAAGAATAACAGAATGGTTTCGCGATGGTTCACTCACTTATGTTATACCAAGAATGATCCATTTTAA

>Dipodomys_ordii_intact_V1R90_4

ATGACAGCCACCGCAGGTGACACCGTGTTCAGGCTCCTCCTTGTGTCTAAGGTGTGTCTGGGTGTCCTGGGGAACTGTATCCTCTTCCTGCTCTATGCGTACAGCTCCGTGTGCAAGCCCCGGCTCCGGAAGCCCATCACCATGGTTTTCATGCACCTGACCCTGGTCAATGCCTTGACCATCATGTTCCATTCCATGCCTTACATCATCTCCTCATATGGAGTCCTGTGTTTCTGGGATGATGCCACGTGTAAGGCGGTTTTGTTCCTGTTCAGGGTCACTCGGGGACTGTCCACCTGCACTACCACCTTCATGAGCGCCTTTCAGGCCCTCACCATCAGCCGCACCCCTGCCCAGTTGGCTTGGCTGAAAAGCCGATCCTCAGCCTGCATCCTCCCCTCGCTGCTCTCCTTCTGGATCTTGAATCCCATCATGTATTTCAATGTCATCACAAATGTGGAATCCAATTGCAATTCCACAGTCATGAGCCTGGGATTTTCTCATCCCTACTGTCAAACAAAGTTTGGAAGGGGCAACCCAACGCCCATTATCAGCTCCATTGTGCTGCGAGATACTCTGTTCTTGGTCCTCATGGTGGCCCCCAGCCTCTACATGGTGACGCTCCTCTACCGACACCGCCACAGAGCCCGGCACCTCCAGAGTCCCAAACTTGCCTCCCAGCCGGCGCCCGAGAACACAGCCACCCACACCATCCTGCTGCTGGTCAGCTGCTTTGTGTTCTTCTATTGCTCCAACAACATCGTGATTGTGTATTCCTTGTACACGCTGGAGAAATTGAACAAATCCAAGGGTGTGCTACTGATGTTGTCCTTCAGCTACCCCACCCTCTGCCCATTCTTGCTCATGAAGAATAACAGAATGGTTACACGATGGATCACTCACTCATGTTATACCAAGAATGATCCATTGTGA

>Dipodomys_ordii_intact_V1R90_5

ATGACAGCCACCGCAGGCGACACTGTGTTCAGGCTCTTCTTTGTGTCTAAGGTGTGTCTGGGAGTCCTAGGGAACTGTGTACTCTTCCTGCTGTACGCGTACAGCTCCGTATGCAAACCCCGGCTCCGGAAGCCCATTGCCATAGTTTTCATGCACTTGACCCTGGTCAATGCCTTGAGCATCATTTTTGAGTCCATGCCCTTCATCGTCTCCTCATATGGAGTCCTGTGTTTCTGGGATGATGCTATGTGTAAGGCAATGCTGTTCCTGTTCAGGATCACCCGGGGACTATCCACCTGCACTACCACCTTCCTGAGTGCCTTCCAGGCCCTCAACATCAGCCCAACCCCTGCCCAGTGGGCTTGGCTGAAAAGCCGATCCTACACCTGCATCCTCCCCTCGCTGCTCTCCTTCTGGATCTTGAATCCCATCATGTATTTCAACATCATCACAAATGTGGAATCCAATTGCAATTCCACCATCATGAGCCTGGGATTTTCTCATCCCTACTGTCAAACGAAGTTTGGAAGGGACAACCCAACACCCATTATCAGCTCCATTGTGTTGCGAGATGCTCTGTGCCTGGTCCTCATGGTGGCCCCCAGCCTCTACATGGTGATGCTCCTCTACCGACACCACCGCAGAGTCCGGCACCTCCACAGTTCAAACCTTGTGTCCCAGCCAGCGCCTGAGAACACAGCCTCCCACACCATCCTGCTGCTGGTCTGCTGCTTCGTGTTCTTCCATTGCTCCAACAACATCGTGACTGTGTATTCTTTGTACACGCTGGAGAAATTGAACAAATCCAAGGGTGTGCTTGTGATGTTGTCCTTCAGCTACTCCACCCTCTTCCCGTTCTTGCTCATGAAGAATAACAGAATGGTTTCAGGATGGTTCACTCACTCATGTTATACCAAGAATAATCCATTGTAA

>Dipodomys_ordii_intact_V1R90_6

ATGAAGATGAATGCTGTTTCCATCCATATTATCATTCAGCAATTGTTTTTATTCCAAACCAGCACTGGGATCTCTGCCAACATCTTCCTTCTTTTCTTACAGCTTTTCACATTTCTCCAAGATTTCAGAGTTAAACCCACTGACATAATCAGCTGTCATTTGGCCCTTGTCCATATGGTAATGCTCCTCATTGCACTGTGTCTTTCTTCTCCAGACATTTTTGAATCATTGAAATTGCAGAGTGACTTCAAATGCAAAGTGTTGATTTATATTCACAGAGTGACTAGGGCTATTTCCATCAGTACCACCTGCCTCCTGAGTGTGTTCCAGGCCATTACTATCAGCCCCAGAACCCCCTGCTTAGTGAGATTGAAACATAAATTTACAAACTATGTTATCTGCATAGTCATTTTTTTCTGGTCCTATAATTTATCTTTTGGTAGTATCCTCATCTTCCATACTGTAGTATATTCTAACACAAGCCAGCAGAATCTAATAAATATCAATGAACAGTGTTCAATAGCTCCTATCAACCCCATCATCAAGGGACTAATTTTCATTCTGACATTGTTCAGAGATATCTTCCTGGTAGGACTTATGCTGCTGTCCAGTGCATACATGGTGATTCTGTTGTTCAGACATCAGAGACAGTCAAAGCACCTCCACAGCACCAGGCTGAACCCAAGAGTCTCCCCAGAGCAAAGGGCCACAAAGACCATTCTGTTGATGGTGAGTTTCTTTGTGATCATATACTGGGCAGACATCATCATCATCATCATCTCCTTATTATGGAAATATGACCGAGTTCTGTTTGGTGTCCAGAAACTTGTGCTCAATTTCTATGCCACTGTTTGTCCTATAGTACAAACCACTTCCCATAAAATTATTAAAGCTACTGTGTACAATATACAATGA

>Dipodomys_ordii_intact_V1R94

ATGATTAGAAAAGATCTGTACCAGAATACTGTTATCAGAAGCACCATTTTCTTTGAATATGCCATTGGAATCTTGGGCAATGCCATCCTCCTTCTCTTCCATATCCTCATCTTCCTTCTTGAGCACAATCCCAAGCCCACCGACCTGCCCATTGGGCTCTTGGCTCTCACCCACCTAGCGATGCTGATAACCGCAGCAATCATAGCTACAGACATTTTAATGTTCTGGGAGGGTTTTTGGGGTGACATCACATGTAAATCACTTGTCTACTTACACAGGTTTATCAGGGCCCTGTCTGTCTGCATCACCTGCCTGCTGAGCGTCCTCCAGGCCATCACCCTCAGCCCTAGAAACTCTTGGTTGGCAAAGTTCAAGAGTAAATCCCCACATCACTACCTGTGTTCTGTTCTTGTGCTGTGGGTCTTCTACATGTTAGCGAACAGTCACATCTTTTTGTCTGTCATTGGTACCCCCAATTTTACCTCCAGCAATCTTGTCTATGTATCTAAATCCTGCTCCGTTTGTCCCATTTCTCACACTCTCAGGCAGGTATTTTCTACACTGCTGACCTTACGGGAAGTCTTCCTCATGGGCCTCATGTTCCTCTCCAGTGGACACATGGCCCTTCTCCTGTGCAGGCACAAGAGGCGTTCCCGGCATCTTCACCGCACCAGCCTTTCTCCAAACGCTTCCCCAGAGCAAAGGGCCACCCGGACCATTCTGCTGCTCATGAGTTTCTTTTTGGTGATGTCCATTTTGTTCTACAGTATCCATGTCTCACGAATTATGTGGAATGATGACCCAATTTTATTTTATATCCTGATTCTTGTTGGCCATATTTATGCCACAGTCAGTCCACTGGTGTTGATCAGTGCTGATAAACGTGTGATCATCTTTGTGAGAACCATGTGTGGTAGAGAGTAA

>Ellobius_lutescens_intact_ancV1R

ATGAAGCTCTCTGCAGACGCGGTTGAGATCATCTCCTGTGCCGTTCTCATCTCTGTGAGCCTTGTAGGAAACACATGCTTATTCTATTCCACAAGCAAGTGCATCGCTGGGGGCCAGCAGACCCCCTTTCTTCTCATCATCAGCCTTGTTTTCGTCCACCTTATTAAAAACCTGGTGGTGAACATCCTCAAGATCGTTTACTCTTCTGGTGTCTTGCTGGATTCAGCGGGCTGCAAAGTTCTGCATTTCACTGCAGCCCTGACAACGTCCCTGGCCATCTGGTTCATGTTACACTTTGCATTGCTCTACCTCCGGAAGCTTTACCAAATTGTCCACTCCTTGAGTGTGGCTGCAAACCCGGATCCACAGAGGTACTCCTTGAAGGTGATTTCTGCACTTTGGGTGGCTGGTGTGGCGGTGTATATCCCAGTGTTGATATATACTAGAAAACCAGAACCCCCGAATTCCAGAAATGATACAGGCTCCTTGTCTACTAACAGAATGTACCTGGATTGCTTGACTGGTTTTGGAAATGACCAGGTAGAGCTCTACTATGGGAAAATATTTTTAGTTTTGGTGGATATTCTTCCTTTAGCTATCTTAATGTTTGTCTGTCTCTGGATGGCTCTCCTCCTTTTAGAGAAGAAAAAGATGACATATGGGGACATCTGGATTGGAGATGATGATTCAGAAACCGAAGTCCTTCGAGGGGCCAAGTTCAGTATCTTGTTAATGTTGCTGATCACTCCACTGTGGGTTTCTCACTTTGTCTTAGTCTGCTTCTTGAAGGACTTGGCAACGTGCGTCTTTATTCCAGCTGCTCTCACAGCCCTCTCCTCTGGCTTCTCCGCTCTCAGTCCTTTCCTGCTTATGCTGGTTAATTACAAAATGAAGCTGGTGTCCTTCTGTGGAGTCAAACAGGAAAAGTCCGCAACACAGCCTGCAGATGCCATTCTGTCTCCATATGCTTGA

>Ellobius_lutescens_intact_V1R1

ATGTACTCTGCAAATTTGAATATGGGGTTTATTTTCCTCATACAGACTGTCATTGGGATCATGGGAAATTCTTCCCTCTTCCTTCTGTACATCTTCCCTCTGCTTCCTGGAAATCACACGAGACCTATAGATTTGGTTCTCAGTCAACTACTTTTGGCCAACTTAATAGTTTTGTTGTCTAGGGGTATTCCACAGACATTGATAACATTGGGATGGAAATGTTTCCTGAGTGATACAAGTTGCAAACTTGTCTTCTACTGGTATAGATTTGGCACTGGGGTGTCAGTCAGCACTGTCTGTCTCTTTAATGGCTTCCAGGCCATTAAGCTCAAACCCAGAATGTGTAGGTGGATTGCACTCAAAATACAATCCCAAGAGTTCATTGGCTACTGCTGTCTCTTGACCTGGTTCTTTCATCTCCTCATGAATTTATTTCTTCCTTTTCTAGTGAATGGTCCATTGAATGAGAGAAATGCCAGTGTAGAAAGCAATGGTGGTTATTGCTCCTGGACAATACCTGCTGGATGTGTCTTCCTATGTAATCTCTCATACTTTTCCCCTGATATGATAAGTTTGGTTTTCATGTCCTGGGCTAGCATATTGGTGATTGTTGTGCTTCACAGACACAAGCAAAGAGTCCAGCACATTCACACAGACAGCTTCTCCTCTAGTGGTTCCCATGAAGACAGAGCCACATGTAGAATTCTGATCCTGGTGTGTTTCTTTACAGCCTTCTATTCTGTCTACATTAGTTTGACCCTTTGGATGATTCTTGCTGAAAAACATGGCCAGTGGGTAGTGAACAGCTCTGTCCTGCTGGCATCTTGTTTTCCAGCATTCAGCCCCTATGTGCTCATTCTCACTGACACCAGGGTCTCACGGGTCTGTTTTGCCTGCAGAACATGA

>Ellobius_lutescens_intact_V1R2

ATGGCTCCTGAGAATTTGTCAATGGGAATTTTGTTCTTTTTTCAGACAGCTGTGGGGATCTTTGGCAATTGGTCAATTCTTCTTCCTTATGTTGCATCTGTATTCACTGGAAAAAGTTTGATGCCCAAAGACCAGATTTTGAGGCATCTGATTTTAGCCAATTCCCTGGTTATCATCTCAAGAGTAATTCCTCAAATAATGGCACAGTTGGGCTTGCAATATCTCCTGGATGACCTGTTATGTAAACTTACTCTCTACAGTAACCGGGTTTCCCGGGGCATTTCCCTGCACTGCACCTGCCTCTTGAGCTGTTTCCAAGCAATCACAATCAGCTCCAGCAACTCCAGGTGGATGAAGCTGAAATACTCAGTCTCCAAGTACATGGTTCAGTCCTGCTCACTCAGCTGGCTTATTCATCTGCTTCTAAACAGCAAAACAGCTGTAGATGTGATTGGATCTGGTACTAACAAAAACTTCACCAAGAAAATCAAGTTGGGGTACTGCTCAGCATTTGTTTTTGGCAATTCTGTACCTGGGCTACATCTGTCCTTGCTGTGTTTCACTGATGGTCTGTGTTTGGGTCTCATGGTCTGGGCCAGTGTCTTCATGGTGAGAACCCTCCATAGGCACAAGAGTCAGCTACAGCATATCCACAGTGCCCAGCATTCCCTCAGAGTTTCCCCTGAAGACAGAGCCACAAAAACCATCTTGTTCCTTGTGTGTACCTTTGTCCTCTCCTACTCGATGTCCTTCATATTAGTTATCTATACTGTGGTATTTGACAATCCGAGGCTGTGGATAATCAGCATATTTACATTACTAGACACATGCTTCCCTATGTTTTGCCCCTTCATCCTCATCCATAATAACAAATCTGCCCTCAAGAATCATTTTCCCTGCTGTAGGAGAAGGTAA

>Ellobius_lutescens_intact_V1R4_1

ATGATTCTAGGTCACAGACCTAAGCCCACAGACATAATCTCCTTTCACCTGACCTTCATCCACATAGTGCTGCTCCTCACCGGAACTGATATTTGGCTTACAGGCATATTTGAGTCACTAAACATTGAGAATGACTTCAAATGTAAGACAACTTTTTACATGAGCAGGGTGATGAGAGGCCTCTCCATCTGCATCACCTGCCTTCTGAGTGTGTTCCAGGCTGTCACTATCAGTCCCAGTTCCTCTTTGTTGGAAAAATTTAAATATAAACTAAAAAAACACATGATCTATGCTCTCTTATTTATCTGGTCTTTCAACTTGTTATTCATTAGCAACCGAATTTTCTATGTTGGTTCTTTTACCAACATGAGTGAAACCAACCAGATGAAGATCACTGAATCCTGCTCCCTCTTCCCCATGAACTACATCATCAAGGGATTCATTTTAACAGTGGCAATCTCCAGGGATGTATTTCTCATAGGAGTTATGCTGACCACAAGTACATACATGGTGATCATCTTGTTCAGACATCAGAGGCAATGCCAACACCTTCACAGCCTTAGCCACTCGAGAGCATCTCCTGAGAAAAGGGCCACCCAGACCATCTTGCTGCTGGTGGTTTCCTTTGTGGTCATGTACTGGGTGGACTTCATCATCTCATCCGCTGCAGTCCTGTTATGGATGTACGACCCAGTCATCCTGAGTGTTCAGAAGATTGTGATGAATGTCTATCCCACAATTACTCCTCTGATACAAATCACTTCTGATAACAGAATAATCAATATGCTGAAACACATGCAGTCTGTGTGCCATCAGATTTTTAAAAAAGGATAA

>Ellobius_lutescens_intact_V1R4_10

ATGGTTTTGAAATTTATTAAGGAAACAATTTTTCTCTTAATGACTACGGTTGGCACTCTGGGAAACATTTCTGTTTCTGTGAATTATATGTTCAGTTGGTGGGGAGGCCCTGAGAAGAAACCCATACACCTTATTCTCATCCACTTGGCTTTTACAAACATCATAATCCTTCTTACAGAAGGATTTCCAAACACAATGGTAACTTTTGGTTTGAGAAACTTCCTAGATGCCATAGGATGTAAGATAATTGTTTACTTGGAGAGGGTGGCCCGTGGACTCTCCATCTGCACCAGCAGTCTCCTCACTGTGGTCCAGGCCATCATCATCAGTCCAGGAGCATCTGGGTGGAGGAGGCTTAGACCAAAGTCTGCATGGCACATCCTTCCATTCTTTCCATTATTTTGGATACTCAATGCTTTAATAGGTATGAACCTAATCCATTCCATCACAAGTACAAGCCTGAATATATCACAGTTTAAGAGTGAGAGTAACTGTTACTTTATGCTAGAAAGTCAGAAAACAAAATGGATTGTTCTCTCTCTCATGGTCCTGAGAGATGCTGTATTTCAAGGAGCCATGGGAGGGGCCAGTGTCTACATGGTATTTCTTCTCCACAAGCACCACCAGCATGTCCTCTACCTTCAGAATTCTGAGCTTGTCTACAGAACTCCCCCTGAACTGAGAGCTGCTCAGAGTGTCCTCCTTCTGATGCTCTGTTTTGTTTTCTTCTATTGGGCTGACTGTGCTTTTTCTCTATTTTTAAGTCTCTCTTCAGTGGACAACTCCTTGATGGTAAATATTCAAGAATTTATGACTCTTGGTTATGCAACATTTAGCCCTGTTGTGTTGATCCACAGGGATGGACTTCTAACTGAGTGTTGGCATGCTCAGTGGGAGAAATTGAGAAAATGTCTCTCTCCTTTATCTGTTCAATGA

>Ellobius_lutescens_intact_V1R4_11

ATGGACTTCTGGAATCTGGCAATCAGCATTATTTTCTTATCACAAACCACAACTGGAATCCTGGGAAATTTCTCTCTAATTTTCTACTATCTAGTCCTTTACTGCAGAAAATGTACATTAAAGCCCACAGATTTGATTTTGATGAATGTAATGGCAGCCAATGCCTTGATCATTCTCTCTACAGGAGTGCCCCAAACAATGGCAGTTTGGGGACTTAAACAATTCTTGAACGATTTTGGATGTAAGCTCCTATTGTACATTCAAGGTTGTGGTAGGAGTGTGTCCATTGGTACTATTTGCCTCTTGAGTGTCTTTCAGGCCTTGACCATTTGCCCCAGGAAATCCCACTTTAAGGATCATAAAGCCAAAGTTGAGAAGTACATCGGCTGCCACATTTCCCTCCTCTGGATCCTGTACATGTCCATAAATTTCATTTATTTTTCATACACAATTTTGAACAGGAACAGCAAAAATGTGTCACAAAAACGAGATTTTGGATACTGCTCCATTGTGGGGCAGGATGAAATTGTTGATTCACTCTATGCAGCATTGGTGGTTTGCCCTGAAGTCTTTCTTTCTGTGCTTATGGCCTGGTCCAGTGGCTTCATGACTGTCATTCTGTACAGACACAAGCAGAGGGTTCAGCACATCCGCAGCTCTCATGGTTCCAGTAGAATGTCGCCTGAGTCCAGAGTCACCCAGAACATTCTGATCCTTTTGTCTAACTTTTTGGTATTTTATACTCTCTCCTCCATCTTAAGAGGATACATTAGTCTTTTAAATAATAACAGTTGGTGGCTGGTGAACATGAATCGCCTGACTTCTCTGTGTTTTCCATCTTTTGGACCCCTTATTCTCATGAATCATTACTCCATTGTGTCCAGACTTAGTTTGGTCTGGATAAGGAAGAAAAAATATTCAATCTCATTTTAG

>Ellobius_lutescens_intact_V1R4_12

ATGGACTCCAGGAACTTGGCAGTAGGTATAGTGCTTTTACTTCAGAGTGCACTTGGAATTCTAGGAAACTTATCTTTTCTTTTCTACTACTTACTCATTTACTATCATGAACACAAGTTAAAGACTGTAGACTTAATTCTTGCACATGTGTTCACAGCCAACTCCTTGACCATTCTCTCTAAAGGAGTACCCCAAATATTGAAAGTTTTTTGGTGGAAATATTTCTTGAATGATGTTTCATGTGAACTTATTTTATATGTTCTCAGACTGAGCAGGAGCATGTCCATCAGTATCACCTGCATCTTGAGTATCTTCCAGGCCATTACTATCAGTCCTATTGACTCCTATTGGAAAGGTATTAAATTCAAAGTACCAAAATATGTTTGTTGTTCCATTTACATCCTCTGGATCCTGAACATGGTAGTAAATATGGTTTTCCCCATGTGTGCATCTACCAAAAGAAATAGCAAAAATAAGACACAAAAGAGAGATTTTGAATTCTGTTCCTATCCTGGTCATGACATAATAGTAGATTCACTGTTCATAGCATTTTGGGTGTTCCCTGAAGTCTTATTTTCTATACTCATTGTATGTTCCAGCATCTCCATGATTGTCATACTTTACGGCCACAAGAAGAGGGTTCAACATATACTCAGTACTCATACCTCACCAAGAATCTCTCCTGAATCCAGAGCCAGACAGATCATTTTGGTCTTGGTTTGCACCTTTATAGTTTTTTATACCCTCTCCTCCATTTTGCAAGGCTACATTGCTCTTTCTCATAATTTAAATTGGTGGCTATTAAATATCACAGTCATCATTTCTCTGTGTTTTCCTACTTTAAGCCCCTATATAATAAGTCATGATTCTATTATTTCCAGATTTTGCTTTTTTCTAGATTAA

>Ellobius_lutescens_intact_V1R4_2

ATGTCTGCTCATGACAAAACCCTGAAAACCACTGAGGAAACTGCTCTCCAGATCCTCTTGCTTTGCCAGATTGGGATTGGGATTGTGGCCAACATCCTTCTGTTTATCCATAATTTCTCTCCAGTCTTCACTGGTATTCGGCTGAAGCCCACACAGGTGATCCTTAGCCATGTGGCTGTGGCCAATGCATTGATTCTCCTCATCCCTGGGTTTCCAAACAATATGATGGCTTTTGCTCCAAGAAATCCTCTGACTGACCTCAAATGTAAACTTCATCAGTTTGTCACTCTTGTTCCTGGTACTTGGGGCAAGGTAATGGTCAGAGGAAAGGCCCCAAATTTAGTGAGTTATTCATGTTACAGTTGTTGGTTGCTCGGCATCTTAAATAATATCTACATTCCAATGAAAGTCACTGGTCCACAGAAAACAGGCAATGACACTGATTCTAAAAGCAAGATGTTCTGTTCTACCTCTGGTTTCAGTGTAGGCATTGTCTTCTTGCGCTTTGCCCATGATGCCGCTTTCATCAGCATCGTGGCCTGGACCAGCGTCTCCATGGTGCTTCTCCTATATAGACATCGCCAGCTAACACAGTGTATCCGCGCTCCCAATCAGGACCACAGAGGCCATGCTGAAACCAGAGCAGCCCACACCATCCTGTTGCTGGTAGTCACATTTGTTAGCTTTTACCTCCTAAATTTTGTTTGTATCATGTTTCAGACTGTTTTAATGGGTTCTTATTTCTGGTTGAGGCATGTAGGTGAGGTTTTGGTTGTTAGCTTCCCCACCATCTCTCCCTTACTGTTGCTCTCAAGAGATCCTAAGGATCCTTGTTCTTTGCTCTTCAAGCATTGA

>Ellobius_lutescens_intact_V1R4_3

ATGTCCTCATTAAGGATTGTCCTTCATTTACAAGCTGGACTTGGAGTCCTGGCCAATATGTTTCTCCTTGTTTTCTATACTTTCATAATTCTGTGTCACAGACCTAAGCCCATGGACATGATCTCCTGTCAACTGACCTTCATCCACATAGTGCTGCTCCTCACTGGAGGGGATATTTGGCTTTGGGACATATTTGATTCACTGAACATGGACAATGACTTCAAATGTAAGACAAGTTTTTACATAAACAGGGTGATGAGAGGCCTCTCCATCTGCATCACCTGCCTCCTGAGTGTGTTCCAGGCTGTCACTATCAGTCCCAGTTCCTCTCTGTTGGCAAAATTTAAATATAAACTAAAAAAACACACAATTTATGCTTTTTTACTTATTTGGTCTTTCAACTTGTTATTCAGTAGCAACCGAATCTTCTATGCCGGTGCTTTTACCAACGTGAGTGAGACCAACCAGATGAAGGTCACTAAATTCTGCTCACTCTTCTCCATGAACTACATTATCAGGGCACTGATTTTAACAGTGACAACCTCCAGAGATGTATTTCTTGTAGGAGTTATGCTGACCACAAGTGCATACATGGTGATTATCTTGTTCAGACACCAGAGGCAACACAAGCATCTTCACAGCCTCAGCAACCCGAGAGCATCTCTTGAGAAAAGGGCCACCCAGATCATCTTGCTGCTGGTGGTTTCCTTTGTGGTCATGTACTGGGTGGATTTCATCATCTCATCCACTGCAGTCCTGTTATGGATGTACGACCCAGTCATCCTGAGTGTTCAGAAGTTTGTGATGAATGTCTATCCCACAATTACTCCTCTGATACAAATCACTTCTGATAACAGAATAATCAATATGCTGAAACATATGCAGTCTGTGTGCCATCAGATTTTTAGAAAGGGATAA

>Ellobius_lutescens_intact_V1R4_4

ATGCTCTTGAAATATATTAAAGAAATAATTTTCTTCTTCATGACTGTGGTTGGCATGCTGGGAAACATTTCTGTTTCAGTGAACTACATGTTCAGTTGGTACAGAGGGCCTGAGAAGAAACCCATCCACCTTATTCTCATCCACCTGGCCTTTGCAAACTTCCTTATCCTTTTTTCAAAAGGATTGCCAAAGACAATGGCAGCATTTGGTTTTAGAAACTTCCTAGATGACATAGTATGTAAGATCCTCATTTACCTGCAGAGGGTGGCCCGTGGGGTCTCCATCTGCACCAGCAGTCTCCTCACTGTGGTCCAGGCTATCATCATCAGTCCCAGAGAATCTGGATGGAGAAGGCTCAGACCAAAGTCTGCATGGCACATACTTCTATTCTTTTCATTCTTTTGGATACTCAATGCTTTAATAAGTGTGAACCTAATTCACTCTGCCACAAGTAGAATCCTGAATATATCAGAGCTTAATAATGAAGATAACTATTGTTATGTTATGCTAGAAAGTCAGAAAATGATGTGGATTGTTCTCCCTCTCATGGTTCTGAGAGATGCTGTGTTTCAGGGAGCAATGGGAGGGGCCAGTGGCTACATGGTACTTCTTCTCCACAAGCACCACCAGCATGTCCTCTACCTTCAGAACTCCAAGCTTCTCTACAGAACTCCCCCTGAGCTGAGAGCTGCTCAGAGTGTCCTCCTTCTGATGCTCTGCTTTGTTTTCTTCTATTGGACTGACTGTGCTTTTTCTCTACTTTTAAGTAACTCTTTAAATAACAATTCCTTGATGGTAACTATTCAAAAATTTCTGGCCCTTGGTTATGCAACTTTTAGCCCCATTGTGTTGATTCACAGGGAAAGACTTCTGCCTGAGTGTTGGCATGCTCAGTGGGAAAAATTGAGAAAATGTCTCTTCTATTTTTCTATTAGATGA

>Ellobius_lutescens_intact_V1R4_5

ATGGTTTTGGATCCTGTCAAGGGCACAGTCTTCCTCTATCTCACTGGGGTTGGCACCATGGGAAATGTCTTAGTTTTCGTGAGTTACATGCACGTGTTCCAAAGCACTGAGAAGAAACCTATTCACCTCGTTCTTGCTCACTTGGCGCTCACAAACACCATAATGCTTCTTTCAAAGGGAATGCCGAAGACAATAGAAGCCTTTAATTTTAGAAATTTCTTAGATGACACCAGTTGTAAAGTTGTGGTTTACCTGGCAAGAGTGTCCCGGGGCCTCTCCATCTGCACCAGCAGTCTCCTCACTGTGGTGCAAGCCACCACCATCAGTCCCAGAGCCTCCAGGTGGCAGAGGCTCAACCTAAAGACTCCACAGCACATTCTTTCCTCGCTGCTCCTCTTTTGGATCCTCAATTCCTTGATCAGCATGAACTTACTGTATTACATTAAAAATATCAAGAGTGTGAACATAACACAGGGTGAAAGAGGCAACAACTATTGCTATTTTCTGCCAGAAAGCAGGATAACAAGATGGACTTTTCTGACACTTATGGTCCTGAGAGATGCTGTGTTTCAGGCAGCCATGGGAGCTGCCAGTGGCTACATGGTACTTCTTCTCCACAAGCACCACCAGCATGTCCTCTACCTTCAGGACTCTAAGTTTCTCTACAGAACTTCCCCTGAGCTGAGAGCTGCTCAGAGTGTCCTCCTTCTGATGTTATGTTTTCTTTTCTTCTATTGGGCAGATTGTTTTATTTCTTTATATTTTACTTTCTTCATAGAGAGTTATTCCAAAATTCTATATGTTTCAGAGTTTCTCACCCTTGGTTATGCAGTTATCAGCCCCTTCAAACGGATCCACAGAGATGAACATTTGACTAAATGTTGTCATACTCAGTAA

>Ellobius_lutescens_intact_V1R4_6

ATGGTTTTGGATCCAGTCAAGGGCACAGTCTTCCTCTATCTCACTGGGGTTGGCACCATGGGAAATGTCTTAGTTTTCGTGAGTTACATGCACGTGTTCCAAAGCACTGAGAAGAAACCTATTCACCTCGTTCTTGCTCACTTGGCGCTCACAAACACCATAATGCTTCTTTCAAAGGGAATGCCGAAGACAATAGAAGCCTTTAATTTTAGAAATTTCTTAGATGACACCAGTTGTAAAGTTGTGGTTTACCTGGCAAGAGTGTCCCGGGGCCTCTCCATCTGCACCAGCAGTCTCCTCACTGTGGTGCAAGCCACCACCATCAGTCCCAGAGCCTCCAGGTGGCAGAGGCTCAACCTAAAGACTCCACAGCACATTCTTTCCTCGCTGCTCCTCTTTTGGATCCTCAATTCCTTGATCAGCATGAACTTACTGTATTACATTAAAAATATCAAGAGTGTGAACATAACACAGGGTGAAAGAGGCAACAACTATTGCTATTTTCTGCCAGAAAGCAGGATAACAAGATGGACTTTTCTGACACTTATGGTCCTGAGAGATGCTGTGTTTCAGGCAGCCATGGGAGCTGCCAGTGGCTACATGGTACTTCTTCTCCACAAGCACCACCAGCATGTCCTCTACCTTCAGGACTCTAAGTTTCTCTACAGAACTTCCCCTGAGCTGAGAGCTGCTCAGAGTGTCCTCCTTCTGATGTTATGTTTTCTTTTCTTCTATTGGGCAGATTGTTTTATTTCTTTATATTTTACTTTCTTCATAGAGAGTTATTCCAAAATTCTATATGTTTCAGAGTTTCTCACCCTTGGTTATGCAGTTATCAGCCCCTTCAAACGGATCCACAGAGATGAACATTTGACTAAATGTTGTCATACTCAGTAA

>Ellobius_lutescens_intact_V1R4_7

ATGGCCACCAGAGACCTGACTGTGGGCTTCGTCTTCTTGTCTCAGACGGCACTGGGAATGTTGGGAAACTCAGTCTTCCTTTCTTGTTTTATCATTGCTGATTTCTCTGGAAACAGGGTGAAACCCACAGACATGATTGTCAAACACTTGACCATAGCCAACTTCATTGTTCTCTGCAAAGGAATCCCCCAGACAACAGCTGCTTTTAGTAAGACTTACCTTCTAGATTATGTTTCATGTAAACTTACTTTATATTTTCATAGAGTTGCCAGAGGAGTATCCCTTGGCTCCACATCCCTGCTGAGTGTCTTTCAGGCCATCACCATCAGCCCCAGTAATTCCAAATGGGCACAGCTCAAGGTCAGAGCCCCCAGGATCATTGGTCCTTCCCTGGGCCTGTGCTGGGCCCTCCAGATGTTGATATATGCCTTCATTCCCTTGTACACAACTGACATAAGGGCTGGAAGAAATGTTACTGGGATAAAATATTTTGGATACTGTGCTGTCATGAACCCTGGGAGATTAATTAACACACTTAATGCAATTCTATTGACATCCAATGATGTCATGTTTTTGGGACTGATGATGTGGGCCAGTGGCTATATGATGTTTATCCTGCACAAATACAAGCAGAGGGTCCAATACATCCATAGATCCCTGTCTTCTAAATCATCATCTGTGACCAGGGCCACCCAAAGCATCCTCACCCTAGCGAGCAGCTTTGTTCTCTTCTATGTACTCTCTATTGTCTTTACATCATACCTGTCTGTGATAGATGGGACCCTTCAGTGGCTGTCAAATACCAATTTAGCCATGGCTATGTGCTTTCCAGCATTCTGCCCCTTTCTACTGATCAGACACTATGCTTCCTTTTCCTGTCTCTGCTGTACCTGTTCTCACCAGACAACACACTGTGCTTGTGTAGTCAGATAA

>Ellobius_lutescens_intact_V1R4_8

ATGATATCAAGAGATCTGGCCATGGGGATCTTCTTCCTGTCCCAGACTTCGCTGGGCTTCCTGGGGAACTTAGCCTTACTTTGCTGTATTATTGTCTCTTCCTTCAATGGGATCAGGAGAAGGCCCACAGACCTGATTGTCAAACGCCTGACCTGTGCCAACATCTTGGTTCTTCTCTGCAAAGGAATCCCTCAGTCAATGGCTGCTTTTGGTCAAACATATTCTCTAGGTAACATTTCATGCAAACTGGTCTTTTATTTTTATAGAGTTGCTAGGGGAGTATCTCTTGGTTCCACAATCCTGTTGAGTGTCTTTCAGGCCATCACCATCAGTCCCAGCAATTCCAAGTGGGCACAGCTCAAGGTCAGAGCCCCCAGTATCATTAGTCCTTCCCTGGGCCTGTGCTGGGCCCTGGGTCTATCTACAAATAGCTTAATTATTGTGAGACTGAATGATATGAGGGCTAAAGGAAATCGTACTGAATTAAGACAATTTTTACACTGCTTAATAGTAAAAGTTAACAGACAAACTTCCACATTATATGCTATTCTATTGGCTTTCAATGATGTTATGTCTTTGGGACTCATGATATGGGCCAGTGGCTCCATGGTGCTGATATTGTTCAAACATAAACAGAGGGTCCAGCATATCCACAGATCCCTGTCTAATAAATCACTTCATGAAACCAAAGCCACACAAAGAATGCTTGTCCTTTTGAGCAGCTTTGTTGTCTTTTATGTAGCCTCTGTCATCTTAATGATGTATTTTTCTGTTCAGGATGGAGGAGATACATGGGTGACTCATGTCAATGTGGCCATCAATGCTTGCTTCCCAGCACTCAGCCCCTTTCTCCTCATTGGACAATACACCAGGAATTTTCATCCCTGCAGTACATGA

>Ellobius_lutescens_intact_V1R4_9

ATGGACTCCTGTAATCTAGCAATCAGAATCATTTTCTTATCACATATTACCACTGGAATTATGGGAAATTTTTATCTTATATTATACTATATAATACGTTACTATAAAGAATGCACATTGAAGCCCACAGATTTGATTCTCATGAACCTAATGGCAGCAAATGCATTGATCATTCTGTCTTCAGGAGTGCCCCAAACAATGGCAGTTTGGGGACTGAAGCAGTTCTTGAATGATTTTGGATGCGTGATCCTATTGTACATTCAAGGATTTAGTCGAAGTTTGTCCATTTGTACAACCTGCCTATTGAGTGTTTTCCAGGCCATGACCATGAGTCCCAGAAAATCTTGTTGGAAGAACCATAAAGTCAAAGTTGCTAAGAATATTGGCTGCTACATTGCCCTCCTCTGGGTCATGCATATGTTGATAAATTTCATTTTCTTTATCTACCCATTTACCAACATGAATAACAAAAATGTGACAAGAAAGCGAGAATTCGGGTACTGCTCTACTGCAGGGCATGATGAAATCAGTGATTCACTCTATGCAATATTGATAATGTGCCCTGAATTCTTCTTTTCTCTGATCATTGCCTGGTCCAGTGCCTCCATAATCATTTTTCTGTACAGACACAAGCAGAGGGTTCAGCACATCTGCAGTTCTTATGGTTCCAGGAGAAGCTTCCCTGAGTCCAGGGCCACCCAGAATATCCTTGTCCTACTTTCTACCTTTCTGGCTTTTTATACTCTCTCCACCATCTTGCGAAGCTGCGTTGGTCTTTTGTATAAGCACAATTTGTGGCTGGTGAATGTCACTCACCTCACAGCTCTATCTTTTCCCTCTTTTGGACCCTTTGTTCTTAAGAGTCATTACTCCATTGTGTTCAGATTTAGTTTTGCCTGGTTAAGAAAAAAGTCCCCTAATTTTATTATAACTATATAA

>Ellobius_lutescens_intact_V1R44

ATGCTCTTTGGTGGGCACAGACCTAAGCCCATTGATCTCTCCATTGATTTCTTGTCCCTAATCCAACTAACAATGCTCATAACTATGGGCCTCATAGCTGTGGACATGTTTGTATCTCAGGAGAGATGGGATTCCACCACATGCCAATCCCTTATCTATTTGCACAGGTTTTTGAGGGGCCTCTCCCTTCGTGCTACCTGCTTGCTGAATGTCCTTTGGGCCATCACACTCAGCCCTAGAAGCTCCTGTTTGGGAAAATTCAAACATAAATCTCCCTATCATGTCTCATGTGGCCTTCTTTTCCTCTGTATCCTCTATATGTCTCTTAGCAGTCCCTTCTTAGTATCAATCACTGCCACCCTCAATTTGACCTCAGAACACTTTATGTATGTTACTCAGTCTTGCTCACTTCTACCCATGAGCTACTCCCGACAAAGTGCAGTTTCCACACTGCTGGCCCTCAGGGAAGTCTTCCTGATCAGTTTCATGGCCCTCTCCAGCGGGTACATGCTGACTCTCCTGTGCAGGCACAAGAAGCTGTCCCAGAATCTTCACAGCACCAGCCTCTCTCCAAAAGCATCTCCAGAGCTAAGGGCCTCCCAAACCATCCTGCTGCTCATGAGCTTCTTTGTGGTTTTCTACATTTTGGACATTGTTATCTTCCACTCAAGAATGAAGTTCAAGGATGGCTCTTTATTCTACTGTATCCAGATTCTTGTGTCCCATAGCTATGCCACAGTCAGTCCTTTTGTGCTGATTAGTACTGAAAAGCGTATAATTAAGTTTTTGAGATCAATGTGTGGGAGGAAAATAAGTATTTGA

>Ellobius_lutescens_intact_V1R54

ATGAATAAAATCAATAAACTGTCCCGCAACACTAAGGTAAGAAACACCATTTTTTTCGAAGCTGGAATTGGAGTCGCAGGCAACAGCTTCCTTGTTCTCTTCCACATCCTCAGGCTCGTTCGTGGGCAGAGGTCCAGACTCACTGACCTGCCCATTGGTCTCTTGGCTCTAATCCACATACTGATGCTGATAGTCATGAGTTTACTAACTACAGACATTTTTATGCCTTGGAGGAGATGGGGTGACACCACATGCAAATTTATTATGTTCTCGTACAGGTATTTTAGGAGCCTCTCTCTCTGTGCCTCTAGCCTGCTCACCATCCTCCAGGCCATCACCCTCAGTCCCAGAAACTGCTGTCTGGCAAAGTTCAAGTGTAAATCTCCACGCTTCATGCTAGGTTGTCTTCTTTTCCTCAGTGTCGTCTATGCGTCCATTAGCTTTCCCCTCTTATCATACGTGACTGCGACCCCTAATGTGACCTCCTCTAGTCTTATATACCTCACCGAATCTTGCTCTCTTGTACCCATGAGCTACTTTGTCCGGCACACATTTTATATATTATTAGTTGTCAGAGATGCCATCTTTGTAGGTCTCATGGCCTTCTCCAGTGGGTACATGGTGACTTTCCTATGCAAACATAAGAAGCAGTCACAGATTCTCCACAGCACCAGGCTTTCTCTTAAAGCATTCCCAGAACAAAGAGCCACACAGACCATTCTGTACCTCATGAGTTTCTTTGTTGTGATGTACACCTTGGACAACTTCCTTGCCTACTTAAGACTCAGAAGTGATGATCCCGTGATTTATTGTATGTCGATTCTCATAGGTCATAGCTATGCATCGGTCAGTCCTTTTCTGGTCCTCAGCTCTGAAAGAAGTTTAATTAACGCTTTTAAATCCATGTACAAAAGGACAGTAAACATGTGA

>Ellobius_lutescens_intact_V1R90_1

ATGAACAGAAACAACCCACTCTACGATGACCATGGCATAAGAAATGCTTTTTTTTCTGAAATTGCTTTGGGGATCTCAGCCAATACCGTCCTTCTGCTCTTCCTCGTGGTCACGTTCTTTCGGGAGCACAGGCACAAGCCCACCAACTTGATAACCAGTCTCTTGGCTCTAAGCCACATAGTGATGCTGCTGACTATGGCCTTCATAGCTACAGACATTTTGGGGTCCCAGAGATTTTGGGATAACTTCACCTGTAGATCAGTCATTTCCTTGTACAGGGTGATGAGGAGCATCTCCATCTGTGTTACATGTCACCTGAGCATCCTCCAAGCCATCATCCTCATCCCCAGAAGTTCCTCATGGTCCAAGTTCAAACATAAATCCTTACTTCACAATTCCGGCTGCTTTCTTTCCCTGTGGACTTTCTATATGTCCATTAGTGGTTTCATGAATTCCATTGTTGCCACCCCCAATGTGACTTCGCATGTTCTTATATTGGTTACTAAATCCTGTTCTCTTTGGCTTTTCAGTGACTTCAGATACTTCCTTTTTGTACTGGCTGTCTTCCGGAATGCCATTCTTGTAGGGCTCATGGTGCTTTCAAGTGTGTACATGGTGACTGTCTTACGCAGGCATAAAAGGCATTCCCAGTACCTTCACAGCACCAGCATGTCCCCCAAAGCATCTCCAGAGCAGAGGGCCGTCCGTACCATTCTGCTGCTACTGAGTTTCTTTGTGGTCATGTACTGTTTAGACTGCATTGCCTCCTCCTCGAGAAATATGTGGAATAATGACCCAACTCACCGCTGTGTCCAGATGTTTGTGTTCAGTGGCTATGCCACACTCAGCCCTTTGGTGTTCATGAGCACTGAACAGCGTATAAGTAACTTTTTGAAAACCATGCAAGGTGAACAGTAG

>Ellobius_lutescens_intact_V1R90_2

ATGAATAAGATATACACTCATGTCATCATTAAGAACATTTGTTATTTCCAAATTTGCATTGGAATTTCAGCCAATACGTTACTTATTTTGCTGCATGTTATCATGGTCCTCCAAAACTGCAGAGCTAAGCCCACGGAGCTGATCACATGTCACTTGGCCCTTGTTCATATTGTGATGCTGCTCACTGCATTAGATTTTTGGTCTCCAAACATGTTTGAGTCACTGAATTTACAGAATGACTTCAAATGCAAGGCATTGTTTTACCTGAGCAGGGTTATGAGGGGCCTCTCCATCTGTATCACCTGCTTCCTGAGTGTGCTACAGGCCACCATCATCAGCCAAAGCACAGACTGGGTGGTTAAAACTAAACACACACTTACAAATTATATCAAGTATATTTTCTTGTTTTTCTGGTTCCTCAATTTGTCTTTCAGCAGTGATGTCATCTTGTTCACTGTAGCTTATTCCAATACAAGCCAGACAAATCCCCTTGTGATCAGTGAATACTGCTCGATATCTCCAAGTACCCCCATCATGAGGGGACTGTTTTTCATTTTGACATTTTCCAGGGATGTCTTCTTTGTGGGCCTCATGATGTTCTCTAGTGCATACATGGTGTTTCTGTTGTTCTGGCATCACAGGCAATCACTGCATCTCCACAGAAGCAGCCTCTCTGTAAGACACTCCCCAGAGCAAAGAGCCACCTGGACCATCCTGGCTCTGGTGAGTGTCTTTGTGGTCACATACTGGATGAACCTCATAATTTCATCTTACTCGACATTGCACTGGATGTATGACCCAGTCTTTCTGAGTCTTCAAAAACTTGTACTCAATGCCTATGCTACTGTGTGTCCAGTGATACAAATGACTTTCCAGAAGAGAATACACAATATTGTGCAATTTATGTACTGGAAATACAGTCACTTTATAACACAATAA

>Ellobius_talpinus_intact_ancV1R

ATGAAGCTCTCTGCAGACATGCTTGAGATCATCTCCTGTGCCGCTCTCATCTCTGTGAGCCTTGTAGGAAACACATGCTTATTCTATTCCACAAGCAAGTGCATCGCTGGGGGCCTGCAGACCTCCTTTCTTCTCATCATCAGCCTTGTTTTCGTCCACCTTATTAAAAACCTGGTGGTGAACACCCTCAAGATCGTTTACTCTTCTGGTATCTTGCTGGATTCAGCGGGCTGCAAAGTTCTGCATTTCACTGCAGCCCTGACAACGTCCCTGGCCATCTGGTTCATGTTACACTTTGCCTTGCTCTACCTCCGGAAGCTTTACCAAATTGTCTACTCCTTGAGTGGGGCTGCAAACCCGGATCCACAGAGGTACTCCTTGAAGGGGATTTCTGCACTTTGGGTGGCTGGTGTGGCGGTGTATATCCCAGTATTGATATATACTAGAAAACCAGGACCCCCGAATTCTGGAAATGATACAGGCTCCTTGCCTACTAACAGAATGTACCTGGGTTGCTTGACTGGTTTTGGAAATGACCAGGTAGAGCTCTACTATGGGAAAATATTTTTGGTTTTGGTGGATATTCTTCCTTTAGCTATCTTAGTGTTTGTCTGTCTCTGGATGGCTCTCCTCCTTTCAGAGAAGAAAAAGATGACATATGGGGACATCTGGATGGGAGATGATGATTCAGAAACCGAAGTCCTTCGAGGGGCCAAGTTCAGTATCTTGTTAATGTTGCTGATCACTCCACTGTGGGTTTCTCACTTTGTCTTAGTCTGCTTCTTGAAGGACTTGGCAACGTGCGTCTTTATTCCAGCTGCTCTCACAGCCCTCTCCTCTGGCTTCTCCGCTCTCAGTCCTTTCCTGCTTATGCTGGTTAATTACAAAATGAAGCTGGTGTCCTTCTGTGGAGTCAGACAGGAAAAGTCCACAACACAGCCTGCAGATGCCGTTCCGTCTCCATATGCTTGA

>Ellobius_talpinus_intact_V1R1

ATGGATGCATTAGTTCACATTGACTTAAACTGGGGAATGATGTTCTTCATTCAGACCATGGCTGGAATCCTAGCCAATTCCTTCCTCTTTCATCTGTATAACTTTCCATTGTTCACTGCACAAGTGGTGAGACCCATGAACTTGATTCTCAATCAGCTGGTCATATCAAACACTCTAGTTCTCTTCTCCAAAGGAATCCCTCAGACAGTGGCCACTTTTGGGTTGACATCGTTCCTGGGGGAGGCTGGGTGCAAACTTCTCTATTTGTACAGAGTGGCCAGAGGGGTCTCCCTCAGCACCACCTCCCTCCTCAGTGGCTTTCAGGCCATTAAGCTTCACCCCAATATTTTGGGGTGGCTGAACCTCAGAACTAGATCCTCAAAGTGCATTATCACCTGCTGTTTCCTTTGCTGGATCCCACAGCTTCTGCTCAATGTCCCTGTTTCTATGATCAAATCTGGTCCAAAGAATAGCAAAAACCTGATCACTAAAGGAATACACAGATACTGTTCCTCAACCATGCCTGAGAGGTTAACTTTCTTACTAAAAGCAGTGGTTCTATCCCTAAGTGATATTATGTGTCTGATCCTCATGACCTGGGCCAGTGGCTCCATGGTCCTTACCCTGCATAAACACAAGCATCGAGTCCAGCACATCCATAGCCACAGCCTCTCCCAAAGTCCTTCCCACAAGGACAGAGCCACACGAACCATCCTGATCCTGGTGACCATGTTTCTGTCCTTTTACTCTCTAGCTTCCGTCTTATCATTTTGTCTAACCCAGACTGTGAACCTGAGCCCTTGGCTGCTGAACACCTCTGTGCTGATGTCATTGAGCTTCCCAACACTCAGTCCCCTTGTGTTCAATTTCAGTAATATATGTGCCCCTCATTTCTGCTCTGTCTTTTGGGTAAAGAAAACAAACAGTCCAACTGTGGTCTCTGATGTTTGA

>Ellobius_talpinus_intact_V1R2

ATGGCTCCTGAGAATTTGTCAATGGGAATTTTGTTCTTTTTTCAGACAGCTGTGGGGATCTTTGGCAATTGGTCAATTCTTCTTCCTTATGTTGAATCTGTATTCACTGGAAAAAGTCTGATGCCCAAAGACCAGATTTTGAGGCATCTGATTTTAGCCAATTCCTTGGTTATCATCTCAAGAGTAATTCCTCAAATAATGGCACAGTTGGGCTTGCAATATCTCCTGGATGACCTGTTATGTAAACTTACTCTCTACAGTAACCGGGTGTCCCGGGGCATTTCCCTGCACTGCACCTGCCTCTTGAGCTGTTTCCAAGCAATCACAATCAGCCCCAGCAACTCCAGGTGGATGAAGCTGAAACACTCAGTCTCCAAGTACATGGTTCAGTCCTGCTCACTCAGTTGGATTGTTCATCTGCTTCTAAACAGCAAAACAGCTGTAGATGTGATTGGATCTGGTACTAACAAAAACTTCACCAAGAAAATCAAGTTGGGGTACTGCTCAGCATTTGTTTTTGGCAATTCTGTACCTGGGCTACATCTGTCCTTGCTGTGTTTCACTGATGGTCTGTGTTTGGGTCTCATGGTCTGGGCCAGTGTCTTTATGGTGAGTACCCTCTATAGGCACAAGAGTCAGCTACAGCACATCCACAGTGCCCAGCATTCCCTCAGAGTCTCCCCTGAAGACAGAGCCACAAAAACCATCTTGATCCTTGTGTGCACCTTTGTCCTCTCCTACTCAATGTCCTTCATATTAGTTATCTATACTGTGGTATTTGACAATCCAAGGCTGTGGATAATTAGCATATTTACATTACTAGACACGTGCTTCCCCACGTTTTGCCCCTTCATCCTCATCCATAATAACAAATCTGCCCTCAAGAATCATTTTCCCTGCTGTAGGAGAAGGTAG

>Ellobius_talpinus_intact_V1R3_1

ATGTTTCCAAGAGACCTAATTTCTGGATTCTTTCTCCTGTCAGAAGTTTTCATTGGATTCATGGGAAACTCGCTGCTCTTCATACTATACATGTACGCCTTCTTAATTCAGCCCCATCTGAAGAAGCCCATAGATATGATCTTCACACATCTGACACTTGTCAATGTTTTGAGCATTGTGTTCAGGCTGATACCAGATGTCATGGCATCCTTTGCAGTCAAGCTCCTTTTCCATGATGTGGGATGTAAGGCAGTTCTGTATGCATGCAGTACTACTTCTATCTGTACTACTTCTCTACTGAGTGCATTTCAAGCCATCACTGTCAGTCCTAATCATTCTAAGTGGGCATGGCTTAAATCCAAGCTTGAGTCCTGCATTTTTCCATCACTCCTCTTCATCTGGCTCATCAATACGTTTCTCTATATTCCAATGGTTGAAAACGTAAAAGGCCAAATCAACTTCACTGTTGTGGGTTCTAGATATCCCCAGACATATTGCCGAAGTAACCAGGTTCGCCATCACACCACCATGTCACTTGTAACTGCATTAACGATTAGAGACATCCTGTTTGTATTTCTCATGATATGGACCAGCCTCTACGTGGTGACCCTCCTGTTCAGACACAATAGGAGAACACAGCATGTCCACAGTTCCAGTGACTCTTCCCAGGCCTCTTCTGAAAAGAAAGCCACACACAGCATCCTTCTGCTTGTGGGTTTCTTTGTGTTTTTCTATTTCTCAAACACCTTTGTTACCTTCTATTCACTTCACAGACCTAAGAACAGCCCAGTATTGGATCTGATTAGTGGAGCTTTATCTTCAGGTTACCCAATCATCTGTCCTTATCTTCTGATGAACAATAGGAAAATTATTTCCAAATTAATTTCTTCCTTTTCAAACTTTGAATATTCCTTTTCTACAAGAGGCTGTCATGGCTAA

>Ellobius_talpinus_intact_V1R3_2

ATGTTTCCAAGAGACCTAATTTCTGGATTCTTTCTCCTGTCAGAAGTTTTCATTGGATTCATGGGAAACTCGCTGCTCTTCATACTATACATGTACGCCTTCTTAATTCAGCCCCATCTGAAGAAGCCCATAGATATGATCTTCACACATCTGACACTTGTCAATGTTTTGAGCATTGTGTTCAGGCTGATACCAGATGTCATGGCATCCTTTGCAGTCAAGCTCCTTTTCCATGATGTGGGATGTAAGGCAGTTCTGTATGCATGCAGTACTACTTCTATCTGTACTACTTCTCTACTGAGTGCATTTCAAGCCATCACTGTCAGTCCTAATCATTCTAAGTGGGCATGGCTTAAATCCAAGCTTGAGTCCTGCATTTTTCCATCACTCCTCTTCATCTGGCTCATCAATACGTTTCTCTATATTCCAATGGTTGAAAACGTAAAAGGCCAAATCAACTTCACTGTTGTGGGTTCTAGATATCCCCAGACATATTGCCGAAGTAACCAGGTTCGCCATCACACCACCATGTCACTTGTAACTGCATTAACGATTAGAGACATCCTGTTTGTATTTCTCATGATATGGACCAGCCTCTACGTGGTGACCCTCCTGTTCAGACACAATAGGAGAACACAGCATGTCCACAGTTCCAGTGACTCTTCCCAGGCCTCTTCTGAAAAGAAAGCCACACACAGCATCCTTCTGCTTGTGGGTTTCTTTGTGTTTTTCTATTTCTCAAACACCTTTGTTACCTTCTATTCATTCTACAGACCTAAGAAGAGCCCAATATTAGATCTGATTAGTGGAGCTTTATCTTCAGGCTATCCAATTGTCTGCTCTTATGTTTTGATGAACAATAGGAAAATTATTTCCAAATTCATTTCTTCCTTTTCAAACTTTTAA

>Ellobius_talpinus_intact_V1R4_1

ATGAAAATTTTATTAAAGGACAAAATGGACTTGAGGAATTTTGCAATAAGAGTAATGTTCTTAACACAAAGTACTGTTGGGATTCTGGGAAATGTCTCCCTTCTTTCCAATTATCTAATTATTTACTATAATGACCACACATTGAAGCCTACAGATTTGATCATCACACATTTGATCACAGCCAACTTTTTGATCATCCTCTCTAAAGGTATGCCCCATACAATAGCAGCTTTTGGGATGAAACAATTTTTCAATGATTTTATGTGCAAACTTTTCTTATATATTCAAAGACTTGGAAGAAGCATGTCTATGGGAACTACCTGCCTCTTGAGTGTTTACCAGGCCATCACCATCAGTCCCCAGAATTCCTTTTGGAAGAATATTAAATTCAAATCTCCAAATTACATTAGCTTCTCTATTTTTCTCTGCTGGATCCCATACAGTATCATAAATTTCATTTTCCCTGTGTTTGTTCATACCAAAAAAGGTCAGAAAAATACAACAGAGAAAAGAGATTTTACATTCTGTTCCACTTTGGGTCGTGACAAAATTGTAGACTTGCTGTACACAGCATTATTGGTATTCCCTGAAGTCCTGTTTTCTATACTCATCATCTGGTCTAGTGGCTTCATGATTGCCATTCTTTACAGGCACAAAAAGCAAATTCAGTGCCTCCGGAGAAATCAAGTTTCCCTCAAAACCTCTCCTGAGTCTAGAGCTACCCAGAGAATTCTGGTTCTTGTGTCCACTTTTGTAGCTTTTTACACCCTCTCTTCCATTTTACAAGGTTTCATTGCTGTTTTATATAAGGCCAACGGGTGGTTGGTAATTATAACAGGCTTCATTTCTATGTGTTTTCCCACTTTGTGCCCTTTTCTTGTGAGTCATGACTTCATTTTGCTCAGATTCTGCTTACTCTGGCTAAGAAATATCAAACCCCAATAA

>Ellobius_talpinus_intact_V1R4_10

ATGTCCTCATTGAAGAATGTCCTCAATTTCCAAGCTGGACTTGGAGCCCTAGCCAATATGTTTCTCCTTTTTTTCTACACTTTCATAATCCTAGGTCACAGACCTAAGCCCACGGACACGATTTCCTGTCAGTTGGCCTTCATCCACATAGTGCTGGTTCTCACTGGAGGGGATATTTGGCTTTGTGACATACTTGAGTCACTGAACATTGATAATGACTTCAAATGTAAGACAACTTTTTACGTAAACAGAGTGATGAGAGGCCTCTCAATCTGTGTCACCTGCCTCCTGAGTGTGTTCCAGGCTGTCACTATCAGTCCCAGTACCTCTATGTTGGCAAAATTTAAATATAAACTAAAAAAATATGTGATTTATGCTTTCTTATTTATTTGGTCTTTCAACTTGTCATTTGTTAGCAACCAGATCTTCTATTCTGGTGCTTTTACCAACGTGAGTGAGACCAACCAAATGAAGGTCACTAAATTCTGCTCACTCTTCTCCATGAACTACATCATCAGGGCACTGATTTTAACAGTGACAATCTCCAGAGACATATTTCTTGTAGGAGTTATGCTGATCACAAGTGCATATATGGTGATTATCTTGTTCAGACATCAGAGGCGATGCAAGCATCTTCACAGCCTCAGCCACCCAAGAGTATCCCCTGAGAAAAGGGCCACCCAGACCATCTTGCTGCTGGTGGTTTCCTTTGTGGTCATGTACTGGGTGGATTTCATCATTTCATCCACCGCAGTCCTGTTATGGATGTACGACCCAGTCATCCTGAGTGTCCAGAAGATTGTGATGAATGTCTATCCCACAATTACTCCTCTGGTACAAATCAGTTCTGATAATAGAATAATCAATATGCTGAAAAGCTTGAGGTCAAAATGTCACCATAGTTTTTAA

>Ellobius_talpinus_intact_V1R4_11

ATGGATTCCTGTAATCTGGCAATCAGAATCATTTTCTTATCACTTATTACCACTGGAATTATGGGAAACTTTTATCTTATATTATACTATATAATACGTTACTATAAAGAATGCACATTGAAGCCCACAGATTTGATTCTAATGAACCTAATGGCATCAAATTCATTGATCATTCTGTCTTCAGGAGTGCCCCAAACAATGGCAGTTTGGGGAGTGAAGCAGTTCTTGAATGATTTTGGATGCATGATCCTATTGTACATTCAAGGATTTAGTCGAAGTTTGTCCATTTGTACAACCTGCCTATTGAGTGTTTTCCAAGCCATGATCATCAGTCCTAGAAAATCTTGTTGGAAGAAACATAAAGTCAAAGTTGCTAAGAATATTGGCTGCTACATTGCCCTCCTCTGGGTCATGCATATGTTGATAAATTTCATTTTCTTTATGTACCCATTTACCAAAATGAATAACAAAAATGTGACAAGAAAACGAGAATTTGGGTACTGCTCTATTGCAGGGCATGATGAAATCAGTGATTCACTCTATGCAATATTGACAATGTGCCCTGAATTCTTCTTTTCTCTGCTCATTGCCTGGTCCAGTGCCTCCATAGTCATTTTTCTGTACAGACACAAGCAGAGGGTTCAGCACATCTGCAGTTCTTATGGTTCCAGGAGAAGCTTCCCTGAGTCCAGGGCCACCCAGAATATCCTTGTCCTACTTTCTACCTTTCTGGCTTTTTATACTGTCTCCACCATCTTGCGAAGCTGCATTGGTCTTTTGTATAAGCACAATTTGTGGCTGGTGAATGTCACTCACCTCACAGCTCTATCTTTTCCCTCTTTTGGACCCTTTGTTCTTAAGAGTCATTACTCCATTGTGTTCAGATTCAGTTTGGCCTGGTTAAGAAAAAAGTCTCCTAATTTTATTATAACTATATAA

>Ellobius_talpinus_intact_V1R4_12

ATGCTTATAAGTAATTCCGTCTTGGGGGTCTTTCTCGTATCTCAGTTATGTGTTGGTGTCATAGGGAACACATCACTGTTCATTTTATACATTTACATTTTCTTCTTTAAGCCTCATTTTAAGAAGTTGATAGATCTGTTTTTCATGCACCTGACAATAGCTAATACAGTGACGATCATATTCACGTTGATACCAGATATTGTGTCATCCTTTGGAGTACCCAATTTTCTGGATGATGTCAGTTGTAAGGTTGTTTCATGTATATACAGAATTTCCCTTGGTCTGTCCATCAGCACCACCTGTATTCTAAGCACATTTCAAGCTGTCACTGTGACTCCCAGTAATTCTCAGTGGGCTTGGCTTAAGCACAAACTATCAACGTGGACGTTTTCTTCCTTACTCTGCTCCTGGCTCATTAACCTGGCCATCTATGGATATATGGTTGAAATGGTAATAGCCAAAAACAATTCTACTCAAATTGGAAATGGATTTTCACGTGCTTACTGTCAAAACAGGAACTTTGGGAACCAAAATTCAGGATCATTTTTGAGTATCATATTCATGCACGATCTCTTTTATGTGGCCATCATGATGTGGACCAGCCTGTACATGGTAATTCTCCTCTACAGACACCGCAAGAGAGCCCAGTATCTCTGCAGCCCAAGCCTCTCCAGCCAGCAATCTCCTGAGCACAGAACCACTCGCAGCATCTTGTTGCTGGTGAGCTGTTTTGTGATCTTGTATTGGTTGAACAATTTCATCACCCTTTATGGATTTTATGCACAAACAAAAATTCCAAGATTGGAGGGAATTAATGCAATTTGGGCAACATGTTATCCAACCATCTGCCCTTTTTTAATAATGAAGAATAATAAACTTATTTTGCACTTCACTTCTTCCTTTTCGGCACTGAGAATGACCTGTTTTCAACGTGCACTTCATGGCTGA

>Ellobius_talpinus_intact_V1R4_2

ATGGACTCTAGGAATTTGGGATTAGGAATAATATTCTTGATAGAGAATACAGTTGGAATTCTGGGAAATGTCTCTCTCCTTTCCTACTACCTAGTTATTTATAATAAGAAACATAAAATAAAGCCCTTGGATTTAATTCTCATGCATCTCATTATGGTTAACCTCTCGATTATTCTTTCCAAAGGAATGGGCAACACAATGACAATTTTTAGCTTGAAACATTTCTTCAATGATTGGAGCTACCAACTTTTTATTTATGTGATAAGAGTTTTCAGGAGCATGTCCATTGCCACCATCTTCCTCCTGAGTGTCTTCCAGTCTATCATTATCAGTCCTAGAAACTCCTTTTGGAAAAACCTTAGAGTCAAATCTTCCAAGGATATTGGTCTCTGCATTTCTCTAAGCTGGGTCTTGTCCATCATGGTAAATGTTCTTTTACCTTTGTACATGTACATAAAATCAAGAAGGAAAAACATAACAAAAGAGATAGATTTTGAACACTATACTGTTGTAGGTAATGACAAAATCTCAGTCTCCTTATATATAGCTTTATCAGTGTTTCCTGAACTCTTATTTTCTGTCCTCATTACCTGTTCCAGCAGCTCAATGATTATCATTTTGTATAGGCACAAACAGAGAATTCAACACATACGCAACACCTGTGCTTTCCATAGTAACTCCCCAGAGTCTAGAGCCACTCAGAGCATACTTGTTCGAGTGTTCACCTTTCTGGTTATTTATACCCTCTCTACTGTCTCACATGGTTGCAGTGCTGTATTGTCTGGTCAAAATTGGTGGTTTGTGAAGATCACAGTCATTATAAGTTTGTGTTTTCCCAGTTTGAGCCCCTTTGTACTTATGAGTCAATCCTCCCCTCTCTGTAGACTGTTCTTTCTCTGGATAAAGGATACAGAATCATCTAATGTTATCATTACTATTTAA

>Ellobius_talpinus_intact_V1R4_3

ATGGGATTCTGGAACCTCGCAATCAGAATTATTTTTTTATCACAAACTACAACTGGAATTCTTGGAAATTTCTCTCTAATTTTCTACTATCTGCTCCTTTACTACAAAAAATACACATTAAAGCACACAGATTGGATTCTTGTGCACCTAATGGCAGCCAATGCCTTGATCATTCTCTCTGCAGGAGTGCCCCAAACAATAGCACTTTGGGGATTCAAATATTATTTGAATGATGTTGGATGCAAGCTCCTATCGTACTTTCAAGGGTCTTCTAGGAGTGTTTCCAATGGCACCACCTGCCTCTTGAATGTTTTTCAGGCCTTGACCATTAGTCCCAGGAATTCCTGTTGGAAGGATTATAAAATCAAAGTTGAAAAGAGGATTGGCTGCCACATTTCTCTCCTCTGGATACTGTACATGTTGGTAAATTCCATTCATTTTGCATACACGCTTGTCAACAAGAAAAGCAACAATGTGACAAGAAAATGGGATTTTGGATACTGCTGCACAGTAGGTTTGAATGAAATTGGTGGTTCACTCTACACAGCATTGGTCATGTGCCCTGAAGTCTTCTTTTCTGTGTTTATGGTCTGGTCTAGTGGATCCATGGTTCTCATTCTGTGTAAACACAAACAGAGGGTTCAACATATTCATGTTTCTTGCAGAACCTCCCCTGAGTCCAGAGCCACCCGGAACATTCTGATCTTAGTGTCTACCTTTCTGACTTTTTATACTCTCTCCTCCATCTTAAGAGGCTACACTGCTCTTTTGTATAATCAAAATTGGTGGCTCAAGAACATTAATGACTTCATTTCTCTCTGTTTCCCATCATTTGTACCCATTACCCTTATATATCATTACTCTATTTTATAA

>Ellobius_talpinus_intact_V1R4_4

ATGACGAACAGTGATGGGACCATGGGAATGATCTTCTTATTACAGACTGTACTTGGAGTTCTGGGCAATTCCTCTCTCCTCTACTACTATCTGTTTCTTTACTTTTCCCAAAGCAGTTTAAGGTCCACAGACTGGGTTCTGATGCATTTGATTGTAGCAAATGTCTTAACTCTCCTATGCAAAGGAGTGCCCCAGACAATGGCAGGTTTTGGTTGGAAAGACTTCCTCAATGATTGTGGATGCAAAGGACTTTTCTATCTTCACAAAGTGGGCAGGAGTATGTCCTTTAGCAGTATCAGTTTCCTGAGTGTCTTCCAAGCCATTACTATCAGCCCTAATGACTCAAAGTGGGCACCACTTAAATGCAAAGCTCCCAAGTATGTTGTTGCCTGCGTAAACCTTAGTTGGATTCTGAACCTTCTTATAAATACTGTTTTTCTTATAAACATAAGTTCAAAACAGGGCAATAAAAGCATCACAATGCTAAAAGACTTTGGGTACTGTTCTTCTGTTAATGTTGGGGAAATAGATAGAGTTCTGCATGTGGTTTACCTGTCATTCCCAGACATTATATGCATGTGGCTAATGCTATGGGCCAGTGGCTCCATGGTGCTCATCCTGCATAGGCACAAGCAGAGTACACAGCACATGTTTAGGAGCAAGGTCTCTCCTCAATTCTCACCACATACCAGAGCTACCCAAACCATCCTTCTCCTGATGAGCACCTTTATCTGCTTTTATCTGCTTTCCTACATCTGTCAAGTCTGTTTGGCTCTTATTTATAATCCTTCCTGGTTCCTGGTGAATATGTCTGTCTTCATTGCTGGCTGTTTCCCAACTGTGAGTCCCTTTTTGCTTATGTGCCGTAACTCCCAAGTGAAGACACTTTGTTTTACTTGTGTCAGGAACAGAGAAGCCCCATGA

>Ellobius_talpinus_intact_V1R4_5

ATGGAATCTGGCTATCATAATTATTATCTTTTCACAAACTGCAACTGGTATTCGGGAAATTTCTCTCTAATTTTCCACTATCTAGTCCTTTACTACAGAGAATACACATTAAAGCCCACAGATTTAATTCTGATGAATATAATGGCAGCCAATGCCTTGATTATTCTCTCTATAGGAGTGCCACAAACAATGGCAATTTTGGGATTTAAGCAATTCTTGAATGATTTTGGATGCAAGCTCCTATTTCACATTCAGGGATGTGGTAGGAGTGTGTCCATTGGTACCACTTGCCTCTTGAGTGTCTTTCAGGCCTTGACCATTTGCCCCAGGAAATACTGCTGGAAGGATCATAAAGTCAAAGTTGAGAAGTACATCATCTACCTCATTTCCCTCATCTGGTTCCTGAACATATTCATAAATTTCATTTATTTTTCATACATAATTTTCAACAGGAACAGCAAAAATATGTCACAAACACGAGATTTTGGATACTGCTCCACTGTAAGGAGGGATAAAATTGGTGATTCACTCTATGCAGCATTGGTGGTTTGCCCTGAAGTCTTTCTTTCTGTGCTCATGGCCTGGTCCAATGGCGTCATGATTGTCATTCTGTACAAACACAAGCAGAGGGTTCAACACATCCGCAGCTCTCCTGAGTTCAGAGTCACCCAGAACATTCTGATCATTTTGTCTAACTTTTTGGCTTTTTACACTGTCTCCTCCATGGTAAGAGGCTCCATTGCTCTTTTAAATAGTCACAACAGGTGGCTGGTGAATATCAGTCACCTGACTTCTCTGTGTTTTCCATCTTTTGGACCATTTGTTCTCATGAATCATTATTCTATTTTGTCCAGGCACTGTTTTGTCCTGATAAGGAATAAACATCATTGA

>Ellobius_talpinus_intact_V1R4_6

ATGGTTTTGGATCCTGTCCAGGGCACAGTCTTCCTCTATCTCACTGGGGTTGGCACCATGGGAAATGTCTTAGTTTTCGTGAGTTACATGCACGTGTTCCAAAGCACTGAGAAGAAACCTATTCACCTCGTTCTTGCTCACTTGGCGCTCACAAATACCATAATGCTTCTTTCAAAGGGAATGCCGAGGACACTAGAAGACTTTAATTTTAGAAATTTCTTAGATGACACCAGTTGTAAAGTTGTGGTTTACCTGGCAAGAGTGTCCCGGGGCCTCTCCATCGGCACCAGCAGTCTCCTCACTGTGGTGCAAGCCATCACCATCAGTCCCAGAGCCTCGTGGTGGCAGAGGCTCAACCTAAAGACTCCAGAGAACATTCTTTCCTCGCTGCTCCTCTTTTGGATCCTCAATTCCTTGATCAGCATGAACATAACACAGGGTGAAAGAGGCAACAACTATTGCTATTTTCTGCCAGAAAGCAGGGTAACGAGATGGATTTTTCTCACCCTTATGGTCCTGAGAGATGCTGTGTTTCATGGAGCCATGGGAGGGGCCAGTGGCTACATGGTACTTCTTCTCCCCAAGCACCACCAGCATGTCCTTTACCTTCAGAACTCCAAGCTTCTCTACAGAACTCCCCCTGAGCTGAGAGCTGCTCAGAGTGTCCTCCTTCTGATGTTATGTTTTCTTTTCTTCTATTTAACAGATTGTTTTATTTCTTTATATTTTACTTTCTTCATAGAGAGTTATTCCAAAATTCTGTATGTTTCAGAGTTTCTCACCCTTGGTTATGCAGTTATCAGCCCCTTCATACTGATCCATAGAGATGAAAATTTGACTAAATGTTGTCATACTCTGTAA

>Ellobius_talpinus_intact_V1R4_7

ATGGCCTCCGGAGATGTGGCTGTAGGATTCTTCTTTCTGGCCCAGACTCTACTAGGAATATTGGGGAACTCAGCCTTGCTTTTCTGTTTAATCATTGCTGACTTCTGTGGAAATAGGACGAAGAAGCCCACAGATCTGATTGTCAAACACTTAACCACAGCCAACTTCATTGTTCTCTGCAAAGGAATCCCTCAGACAACAACTGCTTTTAGTCAGACTCGCTATCTAGATTATACTTCATGTAGCCTTACCTTATATTTTCATAGGGTTGCCAGAGGAGTATCTCTTGGCTCCACATCCCTGATGAGTGTCTTTCAGGCCATCACCATCAGTCCCAGTAATTCCAAGTGGGCACAGCTCAAAGTCAGAGCTCCCAGGATCATTGGTCCTTCCCTGGGCCTGTGCTGGACCCTCCAGATATTGTTATATATCTTCATTCCTTGGTATACAACTGATATAAGGGGTCAAAGAAATATTACTGGGATAAAAGATCTTGGATATTGTGTTGTTATGAATCCTGGGAGACAAATCAGCACAGTTAATACAATCCTGCATATATTCAATGATGTCATCTTTTTGGGAATGATGATGTGGGCCAGTAGCTACATGGTGTTTATCCTGCTCAACCACAAGGAAAGGGTCCAACACCTCCACAGTTCTCTGTCTCCTAAGTCATCACCTGAGACCAGAGCAACCCAAAGCATCCTCATTCTAGTGAGCTGTTTTGTTCTCTTCTATATAGTCTCTATTGTCTTTACAGCTTACCTGTCTCTCCAAGATGTAACCACTAAGTGGGTGTTCAACACTGGTGTGGCTGTGGCAGCATGCTTCCCAGCTTTCTGCCCCTTTCTGCTCATCAGACACTACACTTCACTTTTCAGGATTTGCTGTACCTGTTCTTTCCAGACAGTAATCTATCCTACAGATGCCTAG

>Ellobius_talpinus_intact_V1R4_8

ATGCACTCAAAGAATTTGGCAATGGGAATAATATTCTTCTCACAAACTATAGTTGGAATTCTGGGAAATATATCTCTTCTTTACCACTATCTAGGTCTTTACTATAATGAACATAGGGAGAAGCCTGTAGATTTGATCCTCACCCATCTGTTCACTGCCAACTGCTTGATCATTCTTTCTAAAGGGGTGCCCCACACAATAGTAGCTTTTGGGATGAAGCTGTTCTTCAATGAGTTTATGTGTGAACTGTTTTTATATATTCAGAGACTTGGCAGAATCATGTCCATGGGAACTACCTGCCTCTTGAGTATCTATCAGGCCATCACAATCAGTCCCAAGAACTCCTTTTGGAAGAATCTTGAATTCAAATCATCAAACTACATTGCCTTCTCTATTTCCCTTGGCTGGGTCCTATACATCACTATAAATTTCATTTTCCCTGTGCTTGTGAATATCAAATCGAGTCACAAAAATACAACAGAGAAAAGAGATTTTGCATTTTGCTTCACTTGGGGTCATGATGAAATTGCAGAATCACTGTACACAGCATTGTTTGTTTTCCCTGAATTCTTGCTTTCTGTTCTCATCATCTGGTCCAGTGGCTCCATGATTGTCATTCTTTACAGACACAAGCAGAACACTCAACACATCCGGAGCACTCAAGATTCCCTAAGAACCTCCCCTGAATCTAGAGCCACCCAAAGCATCCTCATTCTGGTGTCCTCTTATGTAGTTTTTTATACCCTCTCTTCCATATTACAAGGTTTCATTGCAGTTTTACATAGACCCAGTTGGTGGCTGGTAAACATCACAGCTATTGTTTCTGTGTGTTTTCCCACTTTGAGTCCTTTTGTTGTGAGTCGTGATTTTATTGTACTCAAATTCTGCTTACTTTGTATAACTAATATTAAAAAAAGAGTAATTTGTTTATAG

>Ellobius_talpinus_intact_V1R4_9

ATGACAGACATCTGGAACCTCATAATTGGACTCATTTTCTTATCACAAACAACAATTGGAATTCTCGGAAATTTCTCACTTATGTTCTATTATGTAGTCCTTTACTATAGAGAATGTACATTAAAGCCCACAGATATTATTCTTTTCAATCTAACAACAGCCAATATCATGATCATTCTCTCTTCAGGATTGCCCCACACAATAACTCCTTTTGTGTTGAGACAGTTATTGAATTATTTTGGATGTAGATTAATTTTTTACATTGAAAGAGTTGGTCGGAGTTTGTCCATTGGAACCTCCTGCCTCTTGAGTGTCTTCCAGGCCATGATGATCAGTCCCAGGGAATACTGTTGGAAGGATCATAAAGTCAAAGTTGCCAGGTCCATTGGCTGCATCATTTCCCTACTCTGGCTTTTCTACATATTGATGCATTTCATTTTCTTTATCTACCCATTTATTGAAAGGAATGGCAAAAACATGACAAGAACACAAGATTTGGGATACTGTTTTATTGTACTGTACAATGAAATCAGTGATTTACTGTTTGTAGCATTGGTGATGTTCCCAGAAGTCCTTTTTTCTTTGCTCATCATCTGGTCCAGTGGCTTCATGATTGTCATCTTGTACAGACACAAGCAGAGGGTTCAGCACATCCGTAGAACACATAGTTCAAGCAGAAACTCCCCTGAGTCCAGAGCCACAAAGAATATCCTGGCACTGGTGTCTACCTTTCTTATTTTTTATACATTATCTTCCATCTTACAAGGCAGCATTGCTCTTTCATATGATCACAACAGGTGGCTGATGAACATCACTTCATTTACTTCTCTATGTTTTCCGTCTTTTGGGCCATTTGTTCTTATGAATCATTACTCCATTGTGTCCAGATTCAGTTTTGTCTGTTGCAGTAATAAAAATTATTGA

>Ellobius_talpinus_intact_V1R44

ATGAATAAAGCTAACACACTCCACATTAGCACAATCCTAAAAATCACCCTGTTCTCCGAAGTGAGTGTTGGGATCTCAGCTAATAGCATCCTCTTTCTTTTCCACCTCTGCATGCTCTTTGGTGGGCACAGACCTAAGCTCATTGATCTCTCCATTGATTTCTTGTCCCTAATCCAACTAACAATGCACATAACTATGGGCCTCATAGCCATGGACATGTTTATATCTCAGGAGAGATGGGATTCCACCACATGCCAATCCCTTATCTATTTGCACAGGTTTTTGAGGGGCCTCTCCCTTTGTGCTACCTGCCTGCTGAATGTCCTTTGGGCCATCACACTCAGCCCTAGAAGCTCCTGTTTGGGAAAACTCAAACATAAATCTCCCTATCATGCCTCATGTGGCCTTCTTTTCCTCTGTATCCTCTATATGTCTCTCAGCAGTCACTTCTTAGTATCAATCACTGCCACCCTCAATTTGACCTCAGAACACTTTATGTATGTTACTCAGTCTTGCTCACTTCTACCCATGAGCTACTCCCGACAAAGTGCAGTTTCCACACTGCTGGCCCTCAGGGAAGTCTTCCTGATCAGTCTCATGGCCCTCTCCAGCGGGTACATGCTGACTCTCCTGTGCAGGCACAAGAAGCTGTCCCAGAATCTTCACAGCACCAGCCTCTCTCCAAAAGCATCTCCAGAGCTAAGGGCCTCCCGGACCATCCTGCTGCTCATGAGCTTCTTTGTGGTTTTCTACATTTTGGACATTGTTATCTTCCACTCAAGAATGAAGTTCAAGGACGGTTCATTATTCTACTGTATCCAGATTCTTGTGTCCCATAGCTATGCCACAGTCAGTCCTTTTGTGCTGATTAGTACTGAAAAGCGTATAATTAAGTTTTGGAGATCAATGTGTGGGAGGACAATAAGTATTTGA

>Ellobius_talpinus_intact_V1R54

ATGAATAAAATCAATAAACTGTCCCGTAACACTAAGGTAAGAAACACCATTTTTTTCGAAGTTGGAATTGGAGTCGCAGGCAACAGCTTCCTTGTTCTCTTCCACATCCTCAGGCTCGTTCGTGGGCAGAGATCCAGACTCACTGACCTGCCCATTGGTCTCTTGGCTCTAATCCACATACTGATGCTGATAACCATGAGTTTACTAACTACAGACATTTTTATGCCTTGGAGGAGATGGAGTGACACCACATGCAAATTTATTATGTTCTCGTACAGGTATTTAAGGAGCCTCTCTCTCTGTGCCTCTAGCCTGCTCACCATCCTCCAGGCCATCACCCTCAGTCCCAGAAGCTCCTGTCTGGCAAAGTTCAAGTGTAAATCTCCACGCTTCATGCTAGGTTGCCTTCTTTTCCTCAGTGTCGTCTATGCATCCATTAGCTTTCCCCTCTTATCATACGTGACTGCGACCCCTAATTTGACCTCATCTAGTCTTATATACCTCACAGAATCTTGCTCTCTTGTACCCATGAGCTACTTTGTCCAGCACACATTTTATATATTATTAGTCATCAGAGATGCCATCTTTGTAGGTCTCATGGCCTTCTCCAGTGGGTACATGGTGACTTTCCTACGCAAACATAAGAAGCAGTCACAGCTTCTTCACAGCACCAGGCTTTCTCTTAAAGCATTCCCAGAACAAAGAGCCACACAGACCATTCTGTACCTCATGAGTTTCTTTGTTGTGATGTATACCTTGGACAACTTCCTTGCCTACTTAAGACTCAGAAGTGATGATCCCGTGATTTATTGTATGTCGATTCTCATAGGTCATAGCTATGCCACAGTCAGTCCTTTTCTGGTCCTCAGCTCTGAAAGAAGTTTAATTAACGCTTTTAAATCCATGTACGTGAGGACAGTAAACGTGATGGCTGAAGGATGCATGCCTAAGTTCTCTTAA

>Ellobius_talpinus_intact_V1R90

ATGGAGTTGTTAACATACAAAACACATTTTTTCTCTGAATTAGGCTTTGGGATTTCATTCAACAGCATCCTCCTCCTCTTCCATATCCTCGAGTTTCTTCTTGAACACAGGCCCCGGACCACTGACCTGTTCATCGGTCTCTTGGCCCTCATTCACCTTGGGATGCTAACAATCATGGGATTCACAGCTGTGGATACTTTCGCACCTCAGAATACATGGAATGACATCACGTGTCAATCCCTTGTCCACTTGTACAGGTTTTTGAGGGGCCTCTCTCTTTGTGCTACATGCCTGCTAAGCATCCTCCAGGCTGTCACCCTCAGCCCCAGAAGCTCCTGTTTGGCAAAGTTCAAGCATAAATCCCCACAGCACAACCTGTGTCTCCTTCTTGTGCTGTGGGCCTTCTACATGTCCTGTGGTGCTCACTTCTGGTTCTCCACTGTTGCTGGCTACAACATCACCTCACATGGGCTCTTATTTGTCACTAAATCCTGTGTTATTTTACCCATGAGCCATATCAGCAGGCACTTATTTACCATAATCAGGATATTTCGGGATGTGTCCTTTATAAGTCTCATGGCCCTCTCCAGCGGGTACATGCTGATTCTCCTGTGCAGGCACAAGAAGCAGGTCCAGCATCTTCACAGCACCAGCCTCTCTCCAAAAGCCTCTCCAGAGCAAAGGGCCTCCCGGACCATCCTGCTGCTCCTGAGCTTCTTTGCGCCGATGTACTGTTTGGACTGCATCATCTCTGCCTCCAGACTGATGCACAGCAGCAACCCGATCCACCACAGTGTTCAGATGATGGTCTCCAATAGCTTCGCCACCATCAGTCCTTTGTTGTTAATTTGTACTGAAAACCGAATTACTAACCTTTTGAAATCCTTTGTACGGGGAGATAGTAAATGTTTAATTATTGAGGCAAAGGTGGGTTCACTTAAGGAGCCAACATGA

>Ellobius_talpinus_intact_V1R94

ATGATAAGTCATTGTTGTACCCTTCTCCTCAGTCACTGTTTGTGCCCACATAAGGTGAATGAGAACAGCGGACTCCACACTAGTTCCAGCTTAAGGACCACCCTTCTCTCCGAAATTGGCATTGGGATCTTAGCCAACAGTATCCTTCTTCTCTTCCACATCCTCAAGTTCTTTTGTGGGCACAGGCCCAAACCTATTGACCTGCCCATTGGTCTTTTGGCTCTAATCCACCTACTGATGCTACTGGTCATGGCAGTCACAGCTACAGACATGTTTATTTCTGGGCAGGGATGGGATGACACCACATGTAAATTCCTTGTCTTCTTGTACAGAGTTTTTAAGGGTCTCTCTCTGTGTGCCACCAGCCTGTTGAGTGTCCTCCAGGCTATCATCCTCAGTCCCAGAAGCTCCTGTTTGGCAAAGTTCAAGCATAAATCTCCCTATTACATCTCATGTGCCCTTCTTCTCCTGAGTATTTTTTATATGTTCATTAGTAGTCACCTCTTAGTGTGCATTATTGCCACACTCAATTTGACCTTGAATAACTTTATATTTGTTACTCAGTCCTGCTCTATTCTACCCAAGAGTTATCTCATGCAAAGTACATTATCCACACTGCTGGCACTCAGGGAAGCCTTTCTTATTAGTCTCATGGCTCTCTCCAGCGGGTACATGGTGACTCTCCTGTGCAGACATAAGAAGCTGTCCCGGCATCTTCACAGCACCAGACTTTCTCCAAAAGCATCCCCAGAGATAAGGGCCACCTGGTCCGTCCTGCTGCTCTTGAGTGTCTTTGTGCTGATGTCCATCCTGGACAGCATCGTCTCCTGCTCAAGAGCTATGTTCCTGGATGACCCAACATTTTCTGCATTCAACTCTTAG

>Fukomys_damarensis_intact_ancV1R

ATGAAGCTCTCTGCAGACCTGCTAGAGATCATCACCTGTGCCATTCTAATCCTCGTGAGCTTTGTAGGAAACATATGCTTATTTTGTTCTACAAGGAAGTGTATCACTGGACGTTTACAGACATCATTTTTTCTAATTTTCAGCCTTGTGTTTGCCCACCTTATTAAGAACTTGGTGGTAAATGTCATGAAAATTGTTTATTCTTCTGGTATCATGTTGGATTCAGCAGGCTGCAAAGTTCTGCACTTCTTGGCAGCCCTGACAACTTCCCTGGCCATCTGGTTCATGTTATACTTTGCGTTGTTCTATCACCTGAGACTGTACCAACTTGTCTACCCCCAGAGCGATGCTGCGAACGTTGACCGTCAGAAGCATTCCTTGAAGGGGATTTCTGCACTTTGGGTGGCTGGTGTGGCTGTGTACATACCAGTTTTAATTTATACTAGAAAACCAGAAAACCTGAATGCAGGAAATGATACAGACGCCTTGTCTGCAACGAGGATTTACATGGATTGCTTAATTGTCTTTGGAAACCAGCAGGTAGAGTTTTACTATGGGAAAATATTTTTAGTTCTGATTGATATTCTTCCTTTAGCCATCTTAGTCTTTGTCTGTTTCTGGATGTCTTTCCTCCTTTCAACAAAAAAGAAGATGACATATGGTGACGTCTGGATTGGCGATGGTGATTCAGAAGTTGAGGTCCTTAGAGGAGCCAAGTTCAGTATTTTATTAGTGCTGCTGATCACCCCCCTGTGGATCTCCCACTTTGTCTTAGTCTGTTTCTTGAATGACTTGGCAGCATGGGTCTTCGTTCCAGCCGTGCTCACAGCCCTCTCTTCAGGCTTTTCTGCTCTCAGTCCTTTCCTGCTTCTGCTGGTTAATTACAAAGTGAAGTTGGTGTCATTCTGTGGTGCCAAAGAGGGAAAATCCACACCACAGCCTACAAATGCCATTCTTTCTCCATATGCTTAA

>Fukomys_damarensis_intact_V1R1_1

ATGGCTGTTGCTACTTTGGGAATGGGGGTTATCTTCCTTATTCAGATGGCAGTGGGACTCCTTGGAAACTTCTCCCTCCTCTGTCTTTATAGCTTCACTTTGCTCACGGGAAACAAAGTGAGACCCACCGACATGATTCTCAACCAGCTGGCCTTAGCCAATTCCTTTGTGCTTGTCTCTAGAGGGGTCCCTCAGATGACGGTAGCTTTTGGATCGAACTGTTTACTACATGAGGCTGGATGTAAACTTGTCTTCTACCTACACAGAGTGGCCAGAGGGGTAACGCTCAGCACTAGCTGTGTACTCAGTGGCTTCCAGGCCGTAAAACTTTGTCCCAATTTCTCTACATGGCTAGAGCTAAGAGTAAGATCCTCAAAGTGCATTGGGTTCACTTGCTTCCTCTGCTGGATCCTGCATCTGTTGGTAAACATCTTTGTTCCCCTCAAAGTGGTTGGTCAAATGTACAGCAAAAATTTAAGTGTGAAAATAAATTATGGATGCTGTTCCTCACTCAGTCCAGATAGAGCCGTACAGTTTCTAGTTGTATCCTTATTCTTTGCAGTTGGCTTTGTATGTTTTGCACTCATGGTCTGGACCAGTAGCTCCATGGTCCTGGTCCTGCACAGACACGGGCAGCGGGTCCAACACATCCACAGCCACAGCTGTTATCCCAGACCTTCCCACGAGGCCAGAGCCACACGGACCATTTTGATCCTTGTAATCTCCTTTTGTTTATTCTACTCATTCTCTTCCATTTTGCATGTTTGGATAACTCTATTTGCAATCCCAGGCCAGTGGCTGGTGAACATGTCTATGATTTTGTCTTCATGTTTCCCAACATTCAGTGCCTTTGTACTCATTCACAGTGATACTCGTTTCTCTGAGTTCTATTTTGCCTGCCAGACAAGGAAACAACTTTTCTTAGTTTGCAAATGA

>Fukomys_damarensis_intact_V1R1_2

ATGGCTGCAGGAAATTTGGATTTGGGAACTATCTTCCTTGCTCAGACTGCAATTGGGATCTTTGGAAATTGTTTCCTCTTTTGCTTTTACACCTTCTGTCTGCTCACTGGATACAACTTGAGACTCACAGACCTAATTCTCAACCAACTGGTTTTAGCCAACTCCCTAGTCCTAGTCTCCAAAGGGATATCGCAAACAACAGTGGCATTTCGATGGAAACATTTCCTGGGCGATGTTGGATGCAAACTTGTATTCTATCTTCACAGAGTGGGCACAGGGCTTTCTTTCAGCACTGTCTGCCTTCTTAATGGCTTCCAGGCAATTAAGCTCAACCCCAGTATTTGTAGATGGATGGAGCTCAAGATTCGGTCCCTAACATTCATTGGCTTCTGCTGCTTCCTGTGCTGGATTCCAAATCTCTCAATAAATTCGTGTATGATAGTAATAGTGAATGGTCCACTGAATTGCAAAAACATTACTGTGAGAACCACTTGTGGACCCTGTTCCTGGACAATGCCAGAGAGGCATGCCTTACTATACACACTCTTATATTTTTCCCTTGACATTCTCAGTGTGGCCTTCATTATCTGGGCCAGTGGCTCCATGGTCCTGGTCCTGCACAGACACAGGCAACAGGTCCAGCACATTCATGGCCACAGCCTCACCCCAGAGTCATCCCACGTAGCCAGAGCCACATGCACCATCATGATCCTGGTGAGCTCCTTTGTGACCTTTTATTCAGTCTATATTATTCTGACCATCTGGGCAACTCTAGTCTCAAATCGTGGCCAGTGGATAGTGAACAGCTCTGTGCTGGCGGCCTCGTGTTTCCCAGCATTCAGCCCCTTTGTGCTCATCTTCAGGGACTCCAGGATCTGTCATTTCTTCATGGTCTGCTGGGAGACACAAACTGTTCTTCATAAGCAGCTGACGTTGTGA

>Fukomys_damarensis_intact_V1R2_1

ATGGCCTCTGCGGACTGGCGATTTGGAGTGATCTTCCTTTTTGAGATCGGCATCGGAGCCTCGGGGAATTTAGCACTCTTGTGTCACTATTGTATCCTGTGCTGCAGGGGCTGCAGGCCCAGGTCCACCGATGTGATCCTCAGGCACCTGACTGTGGCCAACACCCTGGCCATTCTCTCCAGGGGAATCCCAGAGACCATGGCAGCGTTCGGGATGGAAGGCTTCCTCAGCGATGCCGGATGCAAGCCGGTGTTCTACGCTCACAGGGTGGGCAGGGGTGCGTCCATGGGCAGCACCTGCCTCCTGAGTGTCTTCCAGGCCGTCGGCATCGGCCCCAGGCGCTCCAGGGGGGCAGAGCTGAAAGCGAAGGCCCTAAACCACATTGGCTCGTCTAGCGCCCTCTGCTGGGGCCTGCACATGCCGCTCAGCGTCAGAGTTCCTGTGCTGGGGAGCGACACAGGGGTCAATGGAAACATCACCGAAACTGTTGATTTCCAGTGCTGTTCAGCTACGCTTCCTGACAAAGATAAAGCTTCTCTGTTTGGGGTGTTGACACTCTCCCACGACATTCTGTGTTTGGAGCTCATGGTCTGGGCCAGTGGCTCCACGGTTTTCATCCTGTTCAGGCACAAGCAGCGGGTCCACCATCTCCACAGCCACAAAATCTCATCACGACCTTCCCCTGAGACGAGAGCCACCCAAAGCATTGTCATCCTGGTCTGTGCCTTCGTGTCCTTCCATGCCCTGTCTTCCATCATTTACTTTTTGTTCCCCCTTCATTACGAAACTGCCTGGTGGCTGGTCAAGACCTCTGCTGTCAGCAATGCCTGTTTCCCCACTGCCGGCCCCCTGATTCTCCTGAGTCGCGAGCAGTGTGTTTCCAGGGTCATTTGGAAGAAGTAA

>Fukomys_damarensis_intact_V1R2_2

ATGACCACAGATTTGAAGTTCGGAATACTCTTTCTTTTCCAGATTGTGATTGGGACCACTGGGAATTTGTCACTCTTGTTTCATTATATCTCCCTTCAATTCAGTGGAAACAAGACAAGGTCCGCAGATGTGATCCTCAGGCACCTGACCATGGCCAACTCCTTGGTCATTCTCTCCAGGGGAATCCCAGAGACCATGGCCTCGTTTGGGGTGAAGCAGTTCCTCAGTGATGCTGGATGCAAGCTCGTGTTCTACGCTCACAATGTGGGTAAGGGTGTGTCCATAGACTCCACCTGCCTCCTGACTCTCTTCCAGGCCATCAGCATCAGCCCCAGGCGCTCTGTTTGGGCGGAGCTGAAAGTGAAAGCCCTGAGGTACATCAGTTCCTCTACTGTCTTCTGCTGGGTCCTGCAGATGCTGTTAAATATTAGACTTCCTATCATTTTGACCAACAACAACAGCAAGAGAAACATCACAAAAACCATAGACTTTCAATACTGCTCAGCTGGGCCTCATGACAGAGACCCAGGCTCAGTGTTTGCGGTGCTGAGACTCTCCCATGACGTTCTGTGTTTGGCGCTTATGATCTGGGCCAGTGGCTCCATGGTTTTCATCCTGTTCAGGCACAAGCAGCGGGTCCACCATCTCCACAGACACAGCTCATGCAGGTCTTCCCCCGAGACCAGAGCTTCTCAGAGCATCCTCATTCTGGTGAGCGCTTTTGTGTTCTTCTATACCCTGTCTTCCACCCTTCACGTTTGTATGGCTCTTACTCGTGGCTGTACTTGGTGGCTGGTCACAGCCTCTGCCTTCATCAATGCTTGTTTCCCCTTTGCCAGCCCCTTTATTCTCTTGAGTCGAGAACACTGGGCATCCAGGTTCATCTGGAAGAAGTAA

>Fukomys_damarensis_intact_V1R2_3

ATGGCCTCTGCGGACTGGCGATTTGGAGTGATCTTCCTTTTTGAGATCGGCATCGGAGCCTCGGGGAATTTAGCACTCTTGTGTCACTATTGTATCCTGTGCTGCAGGGGCTGCAGGCCCAGGTCCGCCGATGTGATCCTCAGGCACCTGACTGTGGCCAACACCCTGGCCATTCTCTCCAGGGGAATCCCAGAGACCATGGCAGCGTTCGGGATGGAAGGCTTCCTCAGCGATGCCGGATGCAAGCCGGTGTTCTACGCTCACAGGGTGGGCAGGGGTGCGTCCATGGGCAGCACCTGCCTCCTGAGTGTCTTCCAGGCAGTCGGCATCGGCCCCAGGCGCTCCAGGGGGGCAGAGCTGAAAGCGAAGGCCCTAAACCACATTGGCTCGTCTAGCGCCCTCTGCTGGGGCCTGCACATGCCGCTCAGCGTCAGAGTTCCTGTGCTGGGGAGCGACACAGGGGTCAATGGAAACATCACCGAAACTGTTGATTTCCAGTGCTGTTCAGCTACGCTTCCTGACAAAGATAAAGCTTCGCTGTTTGGGGTGTTGACACTCTCCCACGACATTCTGTGTTTGGAGCTCATGGTCTGGGCCAGTGGCTCCACGGTTTTCATCCTGTTCAGGCACAAGCAGCGGGTCCACCATCTCCACAGCCACAGCTCATGCAGGTCTTCCCCCGAGACCAGAGCTTCTCAGAGCATCCTTGTCCTGGTGAGCGCCTTCGTATCCTTCTATACCCTGTCTTCCACCCTTTATGTTTCGTTTTCCTTTTATTTTGAAGGTACTTGGTGGCTGCTCACGACCTCTGCTGTCAGCAAAGCCTGTTTCCCACTGCCAGCCCCTTCATTCTCGTGA

>Fukomys_damarensis_intact_V1R2_4

ATGACATTTATAGATCTGAAATTTGTAATCCTCTTCCTCTTCCAGACTGCCACTGGAGCTCTGGGAAATTTCTCACTTTTGTGTCATTCTATCTTCCTGTACTTCAGTGGATACAGGTCCAGGTCCACAGATGTGATCCTCAGGCACCTGACTGTGGCCAACACCCTGGTCATTCTGTCCAGGGGAATCCCGGAGACCATGGCAGCGTTTGGGGCAGAAGACTTCCTCAGTGATGCCGGATGCAAGCTGGTGTTCTATGTTCAGGCTGTGGGCAGGGGTGTGTCTTTCAGCACCACCTGTCTCCTGAGTGTCTTCCAGGCCATCACCATCAGCCCCAGGAGCTCAGGTTGGGCAGAGGTGAAATTGAAAGCCCTAAAATACATCTGGTCCTGTGCCATCGTTTCTTGGGTCATTCATATGTTGCTAAACATTAGAGTTCCTCTGCTTGTGAATGACAAAAGGAACAACAAAAACATCACAGACATTACCGATTTTCAATACTGTTCGGCTACGAGCTCTGACAAAGACCAAAACTCCATTTTTGCAGCACTGACATTATCACACAACATTCTGTGCTTGAAACTTATGATCTGGAGCAGTGGCTCCATGGTTTTCATTCTGCTCAGGCACAAGCAGAGGACACGGCATCTCCACAGACACAGCTCATGCAGGTCTTCCCCTGAGACCAGAGCCACTCAGAGCATCCTCATCCTGGTCTGTGCCTTCGTGTCCTTCTCTACCCTGTCTTCCATCTGGTATGTCTGGTTTATTCTTTATCGCAAAGCTGCTTGGTGGCTGGTTAAAGCTCATGCCTTAACCAGTGGTTGTTTCCCTACTACCAGTCCCTTCATTCTCATGACTCGAGAGCACTGTGCATGCAGGCCCATGTGGAAGACGTGA

>Fukomys_damarensis_intact_V1R3_1

ATGCCCTCTGTGAGTGTGTCTGTTGGAATAATCTTCCTTGGTCAGATTTTCATTGGAACCCTGGGGAATCTCTCTCTCTTAGGTAAATATTTATTTCTTTACTTCCGTGGATATAAGACAAGACCCATAGATTTGATTCTTGTGCATCTTACTGTGGCCAATATCTTGGTCATTCTCTCTAGAGGGATCCCAGAGACCTTGGCAGCTTATGGGTTGGAAGACTTCCTCAGTGATTTTGGATGCAAATGTGTTTTCTATGCTAGCAGGGTGGGCCGAGGTGTAGCCTTTGGCAGCACCTGTCTCCTGAGTGTCTTCCAGGCCATCACCATCAGCCCCAGCTACTTCAGGTGGGCAAAGCTGAAAGTGAAAGCTCCCAAGTATATAAGCACCTTCACCGCTCTCTGCTGGGTCCTGCACTTGGCGCTGAATACCAGTTTCTCTCTGACTTTGACTAGCAGATTGAAAACCAGAAACATCACAAATAAAATAGACTTTGCTTACTGTTCTGATCCACGTACCACAAAAGGGGTATACACAGGGCATGCAGTGTGTACTGCCATAGTTGATGTTCTGTGCATGGCGGTCATGCTCTTAGCCAGTGGCTCTATGCTTTTCATCTTATTTATGCATAAGAAGCAGGTCCGCCACATGCATAGAACCAGAGGATCCTCTAGATCCTCTTTTGAGACCAGAGCCACCCACACCATCCTTATCGTGATGAGCATTTTTGTATTTTTTTACTTCCTATCTTCTGTCATGCAAATATATGTGACTTTTTTTGGTAGGTCAATAATCTTACTGTTCTCTCTTACTTACTTCACGAGTGCGTGTTTCTCAACTGTCTGCCCCTTTGTGTTCATGACTGTGGCCAGTATGTAA

>Fukomys_damarensis_intact_V1R3_2

ATGGTTGTGAACATTTTTAAGGGAGTAGATTTTTTCTTTCTGTTAGGACTTGGTACTGTGGGGAGTGTCTTTCCTTTTATGAATTATGTGTGCTGTATTGTAGACACTTCAAAGAAATCTATGCACTTTATCCTCATCCATTTGGTTTTTACAAATATCATCCTAGTTCTTTCCAAAGGAATTCCAACAACAATAGCAAGTTTTGGTTTAAGCAACTTCCTAGACGATACAGGCTGTAAGATTGTGGTTTACTTGGAGAGGGTGGCCCGGGGCCTGTCCATCTGCACCAGTGCTCTGCTCACCATGGTCCAGGCCATCACCATCAGTCCCAGGCACTCCAAGTGGAGGAATCTTGGGCCCAGGACTGTCAAGCATGCGGTTCCCCTGCTTCTCTCCTTTTGGGGGCTCAATTCCATGCTAAGCATCAATTTACTCTATATTGTCACAAGTACCTACAGGAACTCATCACACAGCAGTGAAAATGACAACTATTGTGATTTTCTACCCCAAAGTCCGAAAATTAACTGGATTTTCCTCAGTCTCCTGGCCCTGAGAGACACTGTGTTTCAGGGCGTCTTGGGTGGGGCCAGTGGATACATGGTATTTCTTCTCCACAAGCACCACCAGCGGGTTCTCCACCTGCAGAGCTCCAGGTTTGTCTACAAAACTCCCCCTGAGATCAAAGCTGCTCAGAGTGTTCTCCTGCTGATGCTCTGTTTTCTTATCTTTTATTGGGCAGATTGTGTCACAACTTTGTATTTACATTTCTTCTCCAAGACTGAGTCTGTAACAATAAATCTTCAGGAATTTCTGACCATTGGTAATGCAGTTCTCAGCCCTTTTGTGCTGATTCACAGGGATGGACACCTGGCTGAATGCTGGCATGCTCTGGCAGGTAGAAAGATGACAAACTAG

>Fukomys_damarensis_intact_V1R3_3

ATGGCCTCCGCGGATGTTAACGTCGGCATAGTCTTCATTGTCCAGCTCGTCCTGGGAACCCTGGGCAATTTCTCTCTCTTAAGTCAGGACCTATTCCTGTACTTCAGTGGCTGCACGACAAGGCCCACAGACTCCATCCGCAGGCACCTGACCGTGGCCAACCTCTTGGTGATCCTCTCCAGAGGGATCGCAGAGACCATGGCAGAGTTTGGGTTGGAGCACTTCCTCGGGGATGCTGGATGCAAGCTCGTGTTCTCTGTTCACGCCCTGGGCCGAGGTGTAGCCTTTGGCAGCACCTGCCTCCTGAGTGTCTTCCAGGCCATCACCATCAGCCCCATGGACTGGAAGTGGGCACAGTTTAAAGGGAAAGCTCCCAAGTACAGGAGCACATTCACTACTCTGTGCTGGGTCCTGAACTTGCTGTTCACCTTCAGTTTTGTGACTATAACTAGCAAATTAGAAACCGTAAACATCACAAATAAAATAGACCTCACATACTGTTCCGATCCTCGTTCCATTAGAGGATCATATTCTGCAAGTGTATCCTTGGTCTGCATCAGTGATATCCTGTGCATGGGGCTCATGCTCCTAGCCAGTGGCTCCATGGTTTGCATCCTGTACAGGCACAAGCAGCAGGTCCGACACATCCGTAGTCCTCATGTCTCCTCTAGATCATCCCCTGAGACCAGAGCCACTCGCAGGGTATTAGTCCTTATGAACATCTTTGCATGTCTGTACATCACCTCTGCCACCATGCGAGTGTATCTGACTTTTTCTGAAAATCACAGCTTCTTCCTGCGCAGTCTAGGAACCTTCATAAATGGACTTTTCCCAACCATCTGCCCCTTCCTGCTCTTGAGCCATGGTCACAGCGTATCCTCGATCAGCTGTGTCTGCTCTGCAAGGAGCACAGAGCCACCTGATCACAGCAGGAGAAACGCGTGCACTGCCTGCTTTTCAGTATTGACCAAGGGATGGACTCCAGCCCCAAGGAATTCTCTGAAAATGCAGAGGACATAA

>Fukomys_damarensis_intact_V1R3_4

ATGGTTGTGAACATTTTTAAGGGAGTAAATTTTTTCTTTCTGTTAGGACTTGGTACCGTGGGGAACATCTTTCTTTTTATGAATTATGTGTGCTGTATTGGAGACACTGCAAAGAAATCTATGCACTTTATCCTCATCCATTTGGTTTTTACAAATATCATCCTAGTTCTTTCCAAGGGAATACCACGGACAATAGCAGGTTTTGGTGTGAGAAACTTCCTGGATGACACAGGCTGTAAGATGGTGTGTTACCTGCAGAGGGTGGCCCGGGGCCTGTCCATCTGTACCAGCGCTCTCCTCACCGTGGTCCAGGCCATCACCATCAGTCCCAGGCACTCCAGGTGGAGGAGGCTCGAGCCCAGGACTGTCTGGCATGTGCTTCCCCTGCTTCTCTCCTTTTGGGGGCTCAATTCCATGCTAAGCATCAATTTACTCTATATTGTCACAAGTACCTACAGGAACTCATCACACAGCAGTGAAAATGACAACTATTGTGATTTTCTACCCCAAAGTCCGAAAATTAACTGGATTTTCCTCAGTCTCCTGGCCCTGAGAGACATTGTGTTTCAGGGAATCATGGGTGGGGCCAGTGGCTACATGGTGTTTCTTCTCCACAAGCACCACCAGCGGGTTCTCCACCTGCAGAGCTCCAGGTTTGTCTACAAAACTCCCCCTGAGATCAAAGCTGCTCAGAGTGTTCTCCTGCTGATGCTCTGTTTTCTTATCTTTTATTGGTCAGATTGTGTCACATCTTTGAATTTACATTTCTTCTCCAAGACTGACTCTGTAACAATAAATCTTCAGGAATTTCTGACCATTGGTTATGCAGTTCTCAGCCCTTTTGTGCTGATTCACAGGGATGGACACCTGGCTGAATGCTGGCATGCTCTGGCAGGTAGAAAGATGACAAACTAG

>Fukomys_damarensis_intact_V1R3_5

ATGGCTTTGAACTTTGCTAAGAGAGTAATTATCTTCATTCTAAGTGGACTTGGCATCATACGGAACATCTTTGTTTTTATGAATTATATGTGCTTTATTAGAGACACTAAAAAGAAATTTATGCATCTTATTCTCATTCATTTGGTTTTCACAAATATCATCCTACTTCTTTCCAAAGGAATTCCAACAACAATAGCAACTTTTGGGTTAAGAAACTTCCTAGATGACAACACAGGCTGTAAGATTGTGGTTTATTTGGAGAGGATTTCCCGGGGCCTGTCCATCTGTACCAGCGCTCTCCTCACCGTGGTCCAGGCCAGCACCATCAGTCCCAGGCACTCCAGGTGGAGGAGGCTCGAGCCCAGGACTGTCTGGCATGTGCTTCCCCTGCTTCTCTCCTTTTGGGGGCTCAATTCCATGGTAAGCATCAATTTACTCTATATTGTCACAAGTACCTACAGGAACTCATCACACAGCAGTGAAAATGACAACTATTGTGATTTTCTACCCCAAAGTCCGAAAATTAACTGGATTTTCCTCAGTCTCCTGGCCCTGAGAGACACTGTGTTTCAGGGCATCATGGGTGGGGCCAGTGGCTACATGGTATTTCTTCTCCACAAGCACCATCAGCGGGTTCTCCACCTGCAGAGCTCCAGGTTTGTCTACAAAACTCCCCCTGAGATCAAAGCTGCTCAGAGTGTTCTCCTGCTGATGCTCTGTTTTCTTACCTTTTATTGGGCAGATTGTGTCACATCTTTGTATTTACATTTCTTCTCCAAGACTGACTCTGTAACAATAAATCTTCAGGAATTTCTGACCATTGGTTATGCAGTTCTCAGCCCTTTTGTGCTGATTCACAGGGACGGACACCTGGCTGAATGCTGGCATGCTCTGGCAGGTAGAAAGATGACAAACTAG

>Fukomys_damarensis_intact_V1R4_1

ATGATTTCAGTGGATATGGTGACGGCAATAGCCATCGTCACTCAAATTGGAATCGGGATGGTGGGGAATTCTTCCTTCCTTTTCCCTTACATTTATTCCCTTTCCAGGAAACATGGATTGAGACCAATAGCTCATATAATCAGCCATCTTGCACTGGCCAATACATTGTGGATTCTTTGTGGAGAAATCCCTCAGACAATGGCGGCCTTTGGGCTACAGTATTTCCTGGATGATGTTGGATGTAAACTTGTGTTCTATTTTCACAGAGTGGCCTGGGGAAATTCTCTCAGTACCACCTGTCTTCTAGGTGGCATCCAGGCCTTAAGTGTCAACCTAACAAACATGAGATGGTCAGAACTCAAATCCAAATCCATAAAACATATTAATTCTACTTGTATCCTGAGTTGGGTGTTTCATTTGCTGGTAAATATCGTTGTTCCTATGAGAGTGACTGGCCAAAAAAGCAGCAGAAATAATACTGTGAATTCAAATCTGAAATATTGTTCTCGTCTTTTCTTTGATACAACTACAGAATCAGTATGTTTATTTATTTTCTCCTCTATTGATATTATCTGTTTGGGATTCATGACATGGGCTGGTGGAACCATGATACTTTTCCTTCACAGACACAAACAGCAAGTCCAATATATTCGTAGCACTAGACAGTCTCCTCAAACATCCCCAGAGAGCAGGGCCACAAAATCTATCCTGCTGCTTGTGAGCACTTTTGTCTTATTTTATTCTCTCTCTTCCATTTTTGAAGCTTACATTTTTCTTTTCGACAACCCACAGTCATGGCTTGTGAACACCAGTGTAATTCTAACATCTTGCTTTCCAACTTTCAGCCCCTTTTTGCTCTTCAAAAGCAACATCTCTATCTTCCACTTCTGTTCTTCCTGCTGA

>Fukomys_damarensis_intact_V1R4_10

ATGACGGCCAGTGAAGTGGTGATGGGAGTGATCTTCTTATCACAGATTGTGCTCGGAGTTCTGGGCAATTGCTCTCTTCTCTACCGTTACCTGTTCCTTCACTTCACTGGGTGCAGGTTAAGGTCCACGGACCTGATTCTTAAGCACTTGATTGTAGCCAACCTCGTAACTCTCCTGTGTAGAGGGGTACCTGAGACAATGGCAGCTTTCAGTTGGCAAGTTTGCCTCAGCCACGCTGGGTGCAAGCTACTGTTCTATCTTCACAGACTAAGCAGGGGTGCGTCCATGGGCAGCACCTGTCTCCTGAGTGTCTCCTGGGCCATCACCATCAGCCCCAGGGACTCCAGGCCGGCAGAGCTTAAAGTGAAAGCGGCCAAGTATGTTGGCTCCTCCCTGTGCCTGATCTGGGTCCTGTGTGGCCTTGTAAATGTTATTTTCCTTACACACATGACTGGAAAATGGAGCAAGAAAAACCACACAAGCCTAAAAGACTTTGGGTACTGTACCAGTGTTCGTCAGGACAAAACCACTGAGTCGTTATACACAGTGCTGCTGTCCGTCCCTGAGGTTTCCTGTCTGGGGCTCATGCTGTGGACCAGCAGCTCCATGGTTTCCGTCCTGTACAGGCACAGGCAGCAGATGAGACACATCCACAGGACGAATGTCACCCCCCGGTCTTCTCCTGAGTCCAGAGCCACCAAAACCATCCTTCTCCTGGTCAGCACCTACATCTGTTTTTACGCTCTCTCCTGCATCTTTCAGGTTTGGTTGTCTCTTACTTATAATCCTCACTGGCTCCTGCTGAACACATCTGCCATTGTTGCTGGGCTTTTCCCAGCTGTCAGCCCCTTCCTGCTCCTGAGCCGTGACTCCAGTGTGTCCAGGCTCTCTGTTGCCTTTATTAGGAATAGAAAAGCTCCTGCTCATGTGACGAATGGATAA

>Fukomys_damarensis_intact_V1R4_11

ATGAAGATAGAAATGGATTACAACCTTGCCAACAGAACTGTTTATGTTTTTCTAGCTGGACATAGCCTTGTATGGAACATTTCTATTTTTGTGAATAATATGTACAGTGTTAGAGGAGGTATCAAAACCAAATCAATACACCTTATTATTATCCATTTGGCTTTTACAAACATCATAATGCTTGTTTCCAAAGGAGTGCCAAAGATAATACCAGCTTTTGGTGTGAGAAACTTCCTAGATGACACAGGCTGCAAGATTGTGGCTTATCTGGAGAGGGTGGCCCGGGGCCTGTCCATCTGCACCAGTGCTCTCCTCACAGTGGTCCAGGCTGCCGCCATTAGTCCCAGACACTCCAGGTGGAGGAGGCTCAAGCCCAGGGCTTCATGGCAGGTCTTTCCCCTGCTTCTCTTTTTTTGGATTCTCAATTCCTTGATAGACATAAATTTAGTATATTCCATCACAAATATCAGCATGAACATATCACAACTTAGGAAGAGTGACACCTACTGTTTTTATCTACGAGGAAGACAGAAAATGAAATGGCTTTTTCTCACTGTCATGGTCCTACGAGATGCTGTGTTTCAGGGCTTCATGGGTGGAGCCAGTGGCTACATGGTGTTTCTTCTCCACAAGCACCACCAGCGTGTTCTCCACCTGCAGAGCTGCAAGCTCCTCTACAAAACTCCTCCTGAGATCAAAGCAGCTCAAAGTGTTCTTCTTCTGATGCTCTGTTTTCTTTTCTTTTATTGGATAGATTGCGTTCTTGCCCTATACATAAGCTTTGAGAATCATTCCATGGTAATAAATATTCGAGAATTTCTGACCATTGGTTATGCAATTCTTAGTCCTTTTGTATTGATTCACAGAAAAGGACACCTGGCTAATTGCCGGCATGCTCATTAG

>Fukomys_damarensis_intact_V1R4_12

ATGAAGATAGAAATGGTTTACAACCTTGCTAACAGCACAATCTATGTTTTTCTGGCTAGACTTAGCATTATAGGGAACATTTTTGTTTTTGTGAATAATATGTACAATGTTAGAGGAGGTGCCAAAAGCAAATCAATACACTTTATTATTATCCATTTGGCTTTTACAAATATCATAACGGTTGTTTCCAAAGGAGTACCAAAGTTAATACCAGCTTTTGGTGTGAGAAACTTCCTGGGTGACACAGACTGCAAGATTGTGGCTTATCTGGAGAGGGTGGCACGGGGCCTGTCCATCTGCACCAGCGCTCTCCTCACAGTGGTCCAGGCTGCCACCATCAGTCCCAGACACTCCAGCTGGAGGAGGCTCAAGCCCAGAACTGTGTGGCATGTCCTTCCCCTATTTCTCTTCCTCTGGATTCTCAATTCCTTGATTGGCATGAACTTACTTCATTGCGTCAAAAGCTCCAGCATGAATATATCACAATTCAGTAAGCGTGACTATTGTTATTTGCAACCACAAAATCAAAAAATAAGATCACTTTTTGTTACCCTCATGGTAGTAAGGGATGCTGTGTTTCAGGGCGTCACGGGTGGGGCCAGTGACTACATGGTGTTTCTTCTCCACAAGCACCACCAGTGTGTTCTCCACCTGCAGAGCAGCAAGCTTCTCTACAAAACCCGTCCTGAGATCAAAGCTGCCCAAAATGTTCTTCTTCTGATGCTTTGTTTTCTTTTCTTTTATTGGATAGATTGTGTTCTTGCCCTATGTGTAAGTTTTGCGAATCTCTCCATGGTAATAAATATTAGAGAATTTCTGACCGTTGGTTATGCTGTTCTTAGTCCTTTGGTATTGATTCACAGAGAGGGACACCTGGCTGATGGCCGGCATGCTCATTAG

>Fukomys_damarensis_intact_V1R4_13

ATGGTTTTGAACATTATCAAAATAACAATATTCATCCTTCTAACTGGACTTGGCACAGTGGGAAATATGTTTGTTTTTGTAAATTATATGAGTATGTTCATTATGGAACCTAGGAAGAAATCTATACATCTTATTCTCATTCACTTAGCTTTTACAAATATCATCATGCTTCTCTCCAAGGGAGTACTAAAGACAATGGCAGTATTCAGCTCAGGAAACTTCCTCAGTGACACAAGCTGTAAGATTGGTTCATACCTGGAGAGGGTGGCCCGGGGCCTGTCCATCTGCACCATCGCTCTCCTCACAGGGGTGCAGGCCATCACCATCAGTCCGGGGCATTCCAGGTGGAGGAAGCTCAAACTAAAGGATCCATGGCACATTCTACCCTTGTTTTTCTTCTTCTGGATTCTCAACCCTTTGATAAGCATGAACCTGATCCGTTCTGTCCTGAGCAAGAACACATCACAAACTAGTAAAAGTGACCCGAATTGTTATTTTATGCTAAAACTTCAGGGAAAAAACTGGATTTTTCTAATTCTCATGGGTCTGAGAGATACTGTGTTTCTGGGTGTCATGGGTGGGGCCAGTGGCTACATGGTGTTTCTTCTCCACACACACCACCAGCGGGTTCTCTACCTGCAGAACAACAAGGTCTACAAAATTCCCCCTGAGCTCAAAGCCGTGCAGAGTGTTCTCCTTCTGATGCTGAGTTTTCATTTCTTTTATTGGACAGATTGTGCTATTTCTTTATATATAATTGCCTCCTCAGAGAAAGATTTCTTAGCAGTAAATGTTCACGAATTTCTGGGCCCTGCTTATGCGATTGTCAGTCCATTTGTGCTGATTCACAGAGGGACACCTGGCTGA

>Fukomys_damarensis_intact_V1R4_14

ATGGTTGTGAACCTTGTCAAGGGAACAATCTTCCTCTCTCCAACTGTACTTGGCATTCTGGGGAACGTCTTTGTGTTTGTGAATTACATGCTCATCCTTGGAGGCAGTGAGAAGAAATCTATACACTTTATTCTCCTCCACTTGGCTTTTACAAATATCATCATGCTTTTTGCAAAACGGATTCCTAAGACATTAGCAGCTTTTGGGCAGAAAATCTTCCTTGATGACACAGGCTGTAAGACTGTTGTTTACCTGGAGCGAGTGGCCCGGGGCCTGTCCATCTGCACCAGCGCTCTCCTCACGGTAGTCCAGGCCATCACCATCAGTCCCACAGACTCAGTGTGGAGGAGACTCAAGCTAAAGACTCCACAGCACACCCTTCTCATATTTGTCTTCTTTTGGATTCTCAATTCCTTAATAAGCATGAACTTACCATTTTCTATCATAAACATCAACAGTGTGAACATATCACAAATTAGTAAAGGTGACAAGTATTGTCATTTTCTCCCAGAAAGCTGGACAACAAGATGGACCTTTCTCACTCTCATGGTCCTGCGTGATGCTGTGTTTCAGTGCACCATGGGTGGAGCCAGTGTCTACCTGGTATTTGTTCTCCACAAGCACCACCAGCGGGTTCTCCACCTGCAGCAGGCCAAGCTCCTCTACAAAACTCCACCTGAGATCCAAGCTGCTCAAAGTGTCCTCCTTCTGATGCTGTGTTTTCTTTTCTTTTATTGGGCAGATTGTGCTTTTTCTCTATTTCTTAGTTCCTCTCTCAATCTTCATTCCATGATACTAAATATTCAAGAATTTCTGACCCTTGGTTATGCAATTCTGAGTCCATTTGTACTGATTCACAGAGATGGACAACTGAGTGGATGTCTTCACAGTCAGGAGGACAGAAAAATGCACAGAAATTATCTCACTGATCTTTTCTGTAAATAA

>Fukomys_damarensis_intact_V1R4_2

ATGAATTCAAAGGATGATTTGAACTTTATCCGGAAAATAGTGTTCATTTCTTTTACTGGAACTGGAATTGCAGGGAATTTGTTTTTGTTTGTGAAACATGTGTACACTGCAGGTTTGAAGACTGGGAAAAAACCCACAGACTTCATTGTAATCCAACTGGCATTCGCAAATGTAATTACACTCTGGATTTCTGGAATCTCACACATAAGATCACCTTTTAATTTTCATTACTTCTTAGGTAATGTTTCTTGTAAAATTGTGATTTTTCTGGGGAGGGTGGCCCGGGGCCTTTCCCTTTGCACAACCTGCTTTCTCAGTATAGTCCAGGCCATCACAATCAGTCCCCGGAATACCCCATGCCCCCTCAAACCACAGAAAAAATGTCAAGTTCTGTCCTGTCTCCTCCTTCTTTGGATCTTGAATTTTCTGATAAGCTCCAACTTGCTCCACTACATTAGAGCAGTGAATACCACTGCTATTAGAATGCATATTGGGTGTTGTTACATGATGTCATCTAGGCAAATAATCAGGTGGATTTTCCTTTCTCTCATGACTCTTCGGGACATCATCTTCCAGAGTCTGATGGGCTGGAGCAGTGGGTACATGGCTTTGCATCTGTATAATCATCACAAGCGTGTCCTCTACCTGCACAGCTCTAGGTTGGCAAACAACTCCAGCCCAGAAATCAGAGCTACTCTAAGTGTTCTCATTCTCATGGTCTGTTTCCTTTTCTTTTATTGGGCAGATTTTATTTTATCCTTCTACAGAGGCTCAACCATGACACTTGACTTTACAATGCTAAATATAAAAATATTTCTAGACCTTGGTTATGTAGTTCTCAGCCCCTTTGTGCTGATCAGCAGGGATGTCCACATGGTTAAATGTTGGCACAGACAGAGAATTTAG

>Fukomys_damarensis_intact_V1R4_3

ATGAAGTCAGAGGATTATCTGATTAATTTAACCTGCAAGGTAATCTTCCTTGCTCTTATTGGACCTGGCACTGGAGGGAATTCCTTTTTGATTGTGAAGCATGTGTACATGGCCATCACGGATTTTCAGAAAAAGCCCACAAATCTTATTCTCATCCAGCTGGCTTTTGCTAACATGCTGATAATCTGTGCTGTAGGAATTACAGATAGATCAGTTTTTCCTTTCAGTGGCTTCCTAGGTAGTGTTGGCTGTAAAACTGTGGTTTACCTGGGAAGGGTGGCCCGGGGCCTTTCCATCTGCACCTCCTGTCTCCTCAGCATGGTCCAGGCCGTCACCATCAGTCCCAGGACCATGCTGTGGGGCAATCTCAAACCACGAACCTCATGGCAAATTCTTCCCTATCTTCTCCTCTGTTGGATCATTAATTTTCTGATAAGCTCCAACTTGCTGCACTATGTCACATCAGTCAGCAGCATGAACAGATCTGTGATTGGAATGCATGCTGGTTATTGTTATTTCCTGCCATCCAGGCAAATCATCAGGTGGCTTTTCCTCTCTCTCATGGCTCTTCGGGACGTCATCTTCCAGTGTCTCATGGGCTGGAGCAGTGGGCACATGGCTTTCTGTCTGTATGAGCATCACAAGCTTGTGCTCTACCTGCACAGCTCCAGGATGGCAAAGAATTCCAGCCCAGAAATCAGAGCTACGCTAAGCACTCTCATTCTCATGACCTGCTTCCTTTTCTTTTATTGGGCAGATTTCATTTTCTCCTTCTACATAGGGTCCATGGTAACACATGACTTCACAATCCTAAATATTAAAAAATTTCTAGGACTGGGTTACACAGTTCTCAGCCCCTTGGTGCTTATCAGCAGAGATGCCCATATGGTAAAATGCTGGCACGGTCACTGA

>Fukomys_damarensis_intact_V1R4_4

ATGTTGCCTGTGATTCGGAAGATGCTTTGGATGAACCTCATCCAGAGAATAATTTTTCTTTCTCTTACGGGACCAGGAATTTTGGGGAACATCCTCATATTCATAAGACATGCATATGTTTTTGCTTTGATTCCTAAGAAAAATACCATAGATTTTATCATCATTCACCTGGCTTTTTCAAATGCAATCATTATTGGTACCACCAGAATCAGAGATATCTCCACAGTTTTCTACTTCAGAAACTTCCTTGGTAGTGTTGGCTGTAAAACTGTGGTTTACCTGGGAAGGGTGGCCCGGGGCCTTTCCATCTGCACCTCCTGTCTCCTAAGCATCGTCCAGGCCGTCACTATCAGCCCCAGGACCAACCTGTGGGGACAGCTCAAACCACAGACCACATGGCAAGTTCTTCCCTATTTCTTCCTGTTTTGGATCATTAATTTTCTGACAAGCTCCAACTTGCTGCACTCCATCACAGCAGTCAGCAGCATGAACAGATCTGTGATTGGAACGTATGCTGGGTATTGTTATTTGCTGCCATCCAGGCAAATCATCAGCTGGCTTTTCCTTTCTCTCATGGCTCTTCGGGACGTCATCTTCCAGAGTCTCATGGGCTGGAGCAGTGGGCACATGGCTTTCCGTCTGTATGAGCATCACAAGCATGTGCTCTACCTGCACAGCTCCAGGTTGGCAAAGAATTCCAGCCCAGAAATTAGAGCTATGCTAAGCACTCTCATTCTCATGACCTGCTTCCTTTTCTTTTTTTTGGACAGGTTTCATTTTTTCCTTATACATAGGTTCCACTGTGATGAATGA

>Fukomys_damarensis_intact_V1R4_5

ATGATTTTGAACCTTGTCAAGGGAACAATCCTAGCCTTTCTGACTGGACTCGGCACTGTAGGGAATATTTTTGTCTTTGTAAATCATATACTCATGTTTGAGGGCACTGAGAAGAAAGCTGTGCATCTTTTTCTCATCCACTTGGCTTTCACAAATATTATCATGCTTCTGTTCAAAGGAATGCCACAGACAATAGCAGCATTTGGTGTGAGAAACTTCCTAGAAGACACGGGCTGTAAGATTGTGGTTTATATGGAAAGGGTGGCCCGGGGCCTGTCCATCTGCACCAGTGCTCTCCTCACAGTGGTCCAGGCTGTCACCATCAGCCCCAGACACTCTGTGTGGAAGAAGCTAAAGCCTAGGTCTGCATGGTTAATTCTTCCTTCATTTTTCTTCCTGTGGATATACAATTTTTTGATGAATATCAACTTACTCCTTTACATGACAGGTACTAATGTGAACACATCACAAATTAGCACGAGTGACTACTATTGTTATTTTCAACCAGAAAGTCAGCAGATAAGATGGGTTATTCTCAGTATCATGGTCTTTCAGGATGCTATGTTTCAGGGTATCACATGTGGAGCCAGTGGCTACATGGTTTTTGTTCTCCACAAGCATCACCAACGTGTTCTCCACCTGCAGAGCTCCAAGTTCCTATACAAATCTTCCCCTGAGAGCAAAGCTGCTCAAAGCATTCTCCTCCTGATGCTTTGTTTCCTCTTCTTTTATTGGGCAGATTGTATTTCTTGTCTACTTTTTAATTCCTTCTTGAAGAATAATGTTGTACTAAATATTCGAGAATTTCTCGCCCTTGGTTATGCAACTCTCAGCCCATTTGTGCTGATTCACAGAGATGGACACATGTCTGAATGTTGGTGCATGCACTAG

>Fukomys_damarensis_intact_V1R4_6

ATGTTTCCAGGAGACACAGTGTTTGGCTTGGTCCTCTTATGTCAGATGTGCTTGGGCCTAATGGGAAACACATTCCTCTTCATGATCTACATATACAAGTCCTTGTCCCAACCTCACCTGAAGAAGCCTGTAGTTTCGATTTTCATGCACCTGACCTTGGTAAATATTTTGACCATCATATTTGCTTTGCTGCCACCTATCATGTTATCATTTGGAGTACAATATTTTCTGAACAATACTGGTTGTAAGACAGTTGTGTTCCTACAGAGAGTTACTCGAGGTCTTTCCATCTGTACTACCGCTCTCCTCAGTGCTTTTCAAGCTATCACAATTAGTTTCAATAATTCTAAATGGATGTGGCTTAAAGCTAAACTCCCTGTGTTGATTTTGCCCTCCTTCTTTTTCTTCTGGATCGTTAACATGCTCACTTATATCCCCATCATTAGCAGAATGGTAGCCATACACAATTTCACTTTTGTTGGCCATGGGTATTTTCATGCATACTGTCAAACCAGACCATTTGTAAGAAGCAAACCTGGCCCATATATAAGTGTCATATTGATCCGAGACCTCCTGTTTATGATCCTCATGCTCTGTAGTAGCCTGTGCATGGTGAGCCTCCTCTACAGATATCACAGGAGAGCCCAGCATATCCACAGCCCAAGATACTCTTTGCAGACAGCTGCTGCACTCAAAGCCACCCGAAGCATCCTTGTGCTTGTGAGTTGCTTTGTGCTTTTTTACTGCTTAAACAACCTCATGACTCTCTATTCATTTTATACGCCTAAAAAAATATTAGTATTGGAGGAAATTAACGCCATTTTATCATCATGCTACCCAACCTTCTGCCCATTTTTGCTGATTAAAAACAATAAAATTATTTCTCAGTACACCACTTCCTTTTCAATGATGAGAATTTCCTGTTGTTAA

>Fukomys_damarensis_intact_V1R4_7

ATGATCTGGAGTAATTTTGTCTGGAGAATAATCATTTTTTCTGTTGCTGGACTTGGCATCGGAGGGAATTTCTTTTTGTTTGCAAAGCATGTGTACGCTTCTGTCATGGGCCTCAAGAAGAAACCCATGGATTTTATTATCATCCAACTCACTCTTAGTAATGCAATAACACTCTGGGCCTCTGGAATATTAGATATAAGACTGCCTTTCCATTTTAATGACTACTCAGGTAGTGTTGTTTGTAAAATTGTGGTTTATCTGGGAAGGGTGGCCAGGGGCCTTTCCATCTGCACCTCCTGTCTCTTCAGCATGGTCCAGGCCATCACCATCAGTCCCAGAACCGTGCTCTGGGGCAAGCTCAAACCACAGTCCCCATGGCAAGTTCTTACGTCTCTTCTGCTCTTTTGGATCATTAACTTTCTGATAAGCTCCAACTTGCTGCACTATGTCACATCAGTCAGCAGCATGAACAGATCTGCAGTTGGAATGTATCTCAGGCACTGTTACATGATACGATCTAAGCAAATCATCAGGTGGCTTTTCCTCTCTCTCATGGCTCTTCGGGATGTCATCTTCCAGAGTCTCATGGGCTGGAGCAGTGGGCACATGGCTTTCTGTCTGTATGAGCATCGCAAGCGTGTGCTCTACCTGCACAGCTCCAGGTTGGCAAAGAATTCCAGCCCAGAAATCAGAGCTACCATAAGAATTCTCATTCTCATGATCTGCTTCCTTTTTTTTTATTGGGCTGATTTCATTTTCTCCTTCTACATAGGTTCTGTCTTAGCACCTGACTCCATGATATTGAATATTAAAGTATTTCTAGGCCTGGGCTATGCCAGTCTCAGCCCCTTTGTGTTGATCAGCAGAGATGTCAGCAAGGTTAAATATTGCCACAGTCACTGA

>Fukomys_damarensis_intact_V1R4_8

ATGGTTTTGAACCTTGTCAAAGGAGTTACTCTCTTTTTTCTAACTGGACTTGGCACCATGGGGAACATCTTTGTTTTTATGAATTATATATGCATTATTGGAGGCACTGAGAAGATATATATGCACCTTATTCTCATCCATTTGGTCTTTACAAATACCATACTGCTTCTTTCCAAGGGAATTCCAAGTACAATAGCAGCTTTTGGCTTGAGAATCTTCCTGGACATCATAGGTTGTAAGATTGTTATTTACCTGGAGAGGGTGGCCCGAGGTGTCTCCATCTGCACCAGCAGTCTCCTCACGGTGGTCCAGGCCATCACCATCAGCCCCAGACACTGTGTGTGGAAGAGGCTCCAGCCCAAGTCAGCATGGCACATTCTTCCCTTGCTTCTTTTTTTTTGGATTCTCAATTCCTTGTTAAGCATGAATGTATTCTCTGTGATCACAAATATTAACCTAAACTCATCACAAATAGATAAAAGTGACTTCTATTGTGGTTTACCACTAAAAAAACAGAAAATAAACTTCATTTTTGTGACTCTTATGGCCCTGAGAGACACTGCATTTCAGAGTCTTATGGGTGGATCCAGTGTGTACATTGTGTTTCTTCTCCACAGGCACCACCAGCGTGTCCTCTACCTGAAGCTCTCCAAGTTCTTCTACAAAACTCCACCTGAAATCAAAGCTGCTCAAAGTGTCCTTATTCTGATGCTTTCTTTTCTTTTCTTTTATTGGACAGATTGTGCCATATCTTTATACTTCATTGTCTCCTTCAAGAATGCCTCCACAATAGTAAATCTTCAAGTATTTCTGACTCTTGGTTATGCAGTGCTCTGCCCATTTGTGCTGATTCACAGAGATGGACACTTGGTTAAATGTTGTCATTCTCAGTAA

>Fukomys_damarensis_intact_V1R4_9

ATGACTCCCGGTGATTTTATCCTGAGGCTGCTCTTCCTGGCCCAGACTGGGATTGGGGTGGTGGGGAACATCTTTCTTCTCTCAGCTTACGCCCCCACATCCTGTGCCGGCCACGCACGGAGGCCCACACACCTGATTCTCTCCCACATGGCTACGGCCAACTTCTTGGTCCTTCTCTTCAAGGGGATCCCCCACATGATGTTCATCTGGGGGGTGACACCCACCCTGGGCAATGGGGGATGTAAACTCTTGCATTATGTCCCCAGAGTGGCCCGGGGCCTTTCTCTCTGCACCACTTGCCTCCTGAGCCACTTTCAGGCCATCACCATCAGCTCCAGAGCCGGAGGGCGGGTGGGGTCCAAAGACCGAGTTTGGAAGAACGTCAATTTCTCCTGTGTTCTGTGTTGGGTCTTTAACTTGCTGATAAATGTTTTCATTCCCATCCGTGTGGGGAGCCTTCCGCATAACCGCAGTTCTCCCACTGTACTGGACTATGTGCTATGCTCTTTTGAAAAGTCTGACACTTCTGTCCCAACGGGAGCATTGTTCTTGACATTCCCGGATGTTGTGTTCCTGGGGCTCATGGCCCGGGCCAGTGTGCACATGGTGCTTCTCTTGTACAGACACCACCTGCGAGTGAGGCACATCTACACCCACAGCAACTCCCACGGATTCTCCCCCGAGGCAAAAGCCACCCGAACCATCCTCCTTACAGCGGGCACCTTCCTTTTTGTTTATTCCATCAATTCTTTTCTTGTCCTTTATAAGAACGTCTTCTTCAAATCATGCCTGTGGCTGCAGCATATGACTGCTTTTGTGGTCACCTTTTACCCCACTGTCAGCCCGTTACTGGTGCTTGGAAATCACCGAGCACCAAATTGCTGCTCTGAAATGCCGAGAAAGTAA

>Fukomys_damarensis_intact_V1R48

ATGAATATAAACAACAAATTATTCAGTAATATTAACATGAGAATAACCTTTTATTCTCAAGTTGTCATTGGGGTCTCAAGCAATGCCTTCCTTCTTCTCTTCAATATCTTCATGTTCATTCTAGGGCACAGGCCCAGATTCACTCACCTGCCCATTAGCCTCTTGGCCCTAAGCCACATAGTGATTCTGCTAATTGAAGGATTCATAACTGCAGACATTTTTACAACTCAAGCAAGGTTTTGGGATGACATCACATGTAAATCAGTTATCTACTTGTACAGGTTAATGAGGGGACTCTCCATTTGTGTCACTGGCCAGCTGAGTGTCCTTCAGGCTATCACACTCAGTCCCAGGAGCTCTTATTTGGCAAAGTTCAAACATAAAACCTCATGTGACAACTTGTGTTTCCTTTTTTTCCTCTGGGTGTTTTACACATCCATTAGCAGTCATCTTGTAATCTCCATTGCTGCTACCCCAAATTTGACTTCACAAAACATTTTGTATATTACTGAATCCTGCTCTCTTTTATCCATGAATTATTTCCTGCAGCACATACTGTTAGCACTACTGACCTTCAGAGATACAATCTTTATTGGGATAGTGTCCCTTTCAGGTGGATATATAGTGATACTCTTGCGCAGGCATAAGAAGCAATCCCAGCATCTTCACAGCACCAAAATTTCTCTGAAAGCATCCCCAGAACAAAGGGCCATTAGGACCATTCTGCTGCTTATGTGTTTCTTTGTGATCATGACTGTTTTGGACATTATCTCCCACTCAAGAATTGTGCTGAATAATAATCCAATATTTTATTGTATCCAAATTCTTATGGCCCATGGTTATGCCACAGTCAGTCCTTTTGTGTTCATCACTACAGAAAAACGTATCATTAATTTTTTGAGAATCATGTGTGGAAGACAATAA

>Fukomys_damarensis_intact_V1R90_1

ATGAAGATGAATAAAAAAAGCAGATTTTCCAGTTTCATTGGCATACAACACATATTTTTCTTTGAAGTCAGCATTGGGATCATGGCCAATACTGTCCTGCTTCTCTTTCATGTCTTCACATTCATTCTGGAGCACAGGCCCAAACCCACTGACATGACCATTGGGCACTTGTCCCTTATTCACGTAGTGATGCTCCTAACTTCGGGCTTCATAGCTATAGACATTTTTGGGTATAAGGATTTTGTAAATGACATCACATGCAAATCTGTTCTCTATTTGTACAGATTGATGAGAGGCCTCTCTGTCTGTACCACCAGCCATCTGAGTGTGCTCCAGACCATCACCCTCAGCCCTAGAAGCTCCTATTTGGCAAAATTCAAACAAAACTCCCTGAATCAGAACCTGTATTGCTTTCTTTTTTTATGGGTCTTCAATGCGCTCATCAGTGGACCTTTTTCAAACTTCACTGTTGCCATTTCAAATGTGACCTCACATAGTTTTATATTTTTAACTCAATCTTGCTCCCTTTTGCCCATTAGATCCTTCATTAAGTACATATCTTTGTCACTGATGACTTTCCAGCACATGTCCTTTATAGGACTCATGGCCCTCTCAAGTGTGTACATGGTGAGTCTCTTGTGCAGACATAAAAGGCAGTCCCAGCATCTTTACAGCACAAGCCATTCTCCAAAAGCATCTGCAGAACAAAGGGCTACCAGGACTATTTTGTTGCTCATGAGTTTTTTTACTGCCATGTATATTTTAGACTGTGTTATCGAGTCCATCCCCACATGGCTGTGGAATCATGATCCAGTTCGTCATTGTGTCCACATGCTGATTGGCAGTGGTTACGCCACACTCAGTCCTTTGGTGCTTATCAGCACCGAAAGACGAATGATTAAGTGCTTTGTATTCATGTGGCAAAAACACAGTAAATGTCTGTTTAAATTTCATGATGGATAA

>Fukomys_damarensis_intact_V1R90_2

ATGCACAAGGAGGAATTCACAATTTCTGAAGAAGGAAGGCCTAGTGTTGATGAGATTGATGATGGTGGTGATATTTGGGATAAAGAACTTTTCTTCCTTTTCTTCTCAGACATTTTTTCTTTCTCTATTAAGGTAAATAAAAAGAAAAGCTTTGCCAGTCTTTCAGACATAAGAAACATGGTTTTCTTTGAAATCACCATTGGAGTCGTCGCCAACACTGTCCTGCTTCTGTTCCACATCCTCAAATTTCTTCTCAAGCAAAGACCCAAGCCCCTTGACCTGAGTATTGGTCATTTGGCCCTAATACACCTGGTGATGCTGGTAACTGTGGGCCTTGTAGCTACAGACACTTTTGGGTTTCAGGGTTGGGAGAAGGATCTCACATGTAAATTGGTTATCTATGTAAACAGGCTGATGAGGGCCCTCTCCCTCTGTACCACCTGCCTGCTGAGTGTCCTCCAGGCCATCGCCCTCAGCCCCAGAAACTCATGTTTAGCAAAGTTCAAGCACAGATCCACACATCACTACCCATGTTTCCTTGTCTTTTTATGGATCTTCACTATGCTCCTGAATGGTCGTTTGTTAGTCTTCATTGGTGCCACCCCCAATGTGACTTCACACAGTCTCATGTTTGTCACTGAGTCCTGCTCTCACTGGCCCATTAAGTCCTTGTTCAGGTACATATTTTTAACATTGGCAAACACTCAGGATGTCTCCTTTATAGGGCTGATGGCTCTCTCAAGTGGATACATGGTGACGCTCTTGTGCAGGCATAAGAGGCGGTTGCAGCATCTTCACAGCACCAGCCTTTATCCAAAAACATCCCCTGAAGAAAGGGCCACTCAGACCATTCTGCTGCTCATGGGTTTCTTTACTTTCATATACTTTTTGGATTGTATTACCTTCTCTTCCTCTGCAATATTGTGGAAAAATGACCCAATTCATCATTATGTCCAGATGCTTGTGGGCAATAGCTATGCCACAGGCTGTCCTTTGGTGCTAATGAGCACTGAAAAACGAATGACCAAGTGGTTAACATCCAGGTGGAAAAGACTCAATGTTTAG

>Fukomys_damarensis_intact_V1R90_3

ATGAAGATGAAAAAAATCAGCAGATTTTCTAGTTTCATTCACATACAGCACATGGTTTTCTTTGAAGTCAGCATTGGGATCATAGCCAACTTGATCTTGCTTCTCTTTTGTGTCCTCATAATACTTCTGGAGTGCAGAGCCAAGCCTGCTGATCTGACCATTGGTCATTTGGCCTTTATTCACACAATGTTGCTCTTAAATATGGTTTTCATAGCTATGGACATTTTTGGATATCAGGATTTGTGGGATGACATCACATGTACATCTGTTATCTATCTGAGTAGGTTGATGAGGGACCTCTCCGTCTGTACCACCTGCCTGCTGAGTGTCCTCCAGGCCATCACCCTCAGCCCCAGAAGCTCCTATTTGGCCCAGTTCAAATGGAAATCCCTGCTTCAGAACCCGTGTTACTTGCTCTTTCTTTGGGTTTTCAATATGATCATTAATGGTCGCATGTTAATCTCCACTGTTGCCACTCCCAATGTGACTTCAAACAGTGTTATGTTACTTACCACATCCTGTTCACTTTTGCCCATTAGTTCCTTTCTTAAGTACATGTTTTTCTCACTTATGACTTTCCAGCACATATTGTTTATAGGACTCATGGCAGTCTCCAGTGGGTACATGGTGAATCTCTTGTGCAAGCATAAAAGGCAGTCCCAGAATCTTCATAGCACCAGCCTGTCTCCAAGAGCATCTGCAGAACAAAGGGCTACCTGGACAATTTTATTGCTTATGAGTTTCTTTATTGTTATGTACACTTTGGACTGTGTTATTGGGTCCATCTTTTCGATCTTGTGGAATAATGACCCATTTCATCACTGTGTTCTGTTATTGGTTGGCAATGGCTATGCCACAGTCAATCCTTTGGTTCTCATCAGTAGTGAAAGACGAATGATGAGGTGTCTCATATTCATGTGGGGGAAAGATAGTAGATGA

>Fukomys_damarensis_intact_V1R90_4

ATGAATAAAAATAGCAAATTTTTCAGGTTTCTTGATGCACAATGCTTGTTTTTCTTTGAAGTCAGCACTGGAATCATAGCCAACATTATCCTGCTTCTTTTATATATGCTCATATTTCTTCTACAGCACAGGCCCAAGCCGATCGATTTGACCATTACCCATTTGCTCTTTATCCACATAATAATGCTGGTAACAGTGTGTTTGATAGCTTTAGACATTTTTGGGTATCAAGACTTGGGGAATGACATCACGTGTAAATGTGTTATCTATTTGCACAGGTTGATGAGGGGCCTCTCCATCTGTACCACCTGCCTGTTGAGTGTCCTCCAGGCCATTACCCTCAGCCCCAGAAGTTCCCATTTGGCAAAGTTCAAACAGAAATCCCTGCATCAGAACCTATATTGCCTTCTATTTTTATGGGTCATCAATATGCTCATCAGTGGTCGTTTCTTAATCTCCACTATTGCCACCCCTAATGGGACTTCCCATAAGCTTATGTTTGTCACTAAGTCCTGCTCCCTTTGGCCAATTAATTACTTACTTAAGTATATCACTTTCTCCTTGATGACTTTCCAGGAAATTTCTGTTATATCACTAATGGGGCTCTCAAGTGTGTACATGGTGATTCTTTTGTACAGGTACAAAAGACAGTTACAGTATCTTCACAGCACCAACTTGTCTCCAAAAGCATCCCCAGAACAAAGGGCCACTCAGACTATTCTGTTGCTCATGAGTGTCTTTATTGTCATTTATGCAATGGACTGTGTTATCACCTCCACATCTGGAGTGTTCTGGAAACATAACCAAATTCATCACTGTGTGCTGATGCTGATTGGGAATGGCTATGCCACAATCAGTCCTTTGGTGCTAATCAGTACTAAAAAACAAATGACTAAGTGCTCAGCATCCACATTTTGGAAGATAGTAAATGTTTGTTTATTTAGTGATGGAAAATTTCTCTGA

>Fukomys_damarensis_intact_V1R90_5
[truncated: 1,636,577 more chars]
