# Supplementary material for: Convergent reduction of V1R genes in subterranean rodents
Source: BMC Evol Biol. 2019 Aug 30;19:176. doi: 10.1186/s12862-019-1502-4 (PMC6717356; doi:10.1186/s12862-019-1502-4)
Supplement: Supplementary file 2 — Figure S1. The Bayesian tree based on the alignment of 1983 intact V1Rs. (PDF 3369 kb) [file 12862_2019_1502_MOESM2_ESM.pdf]

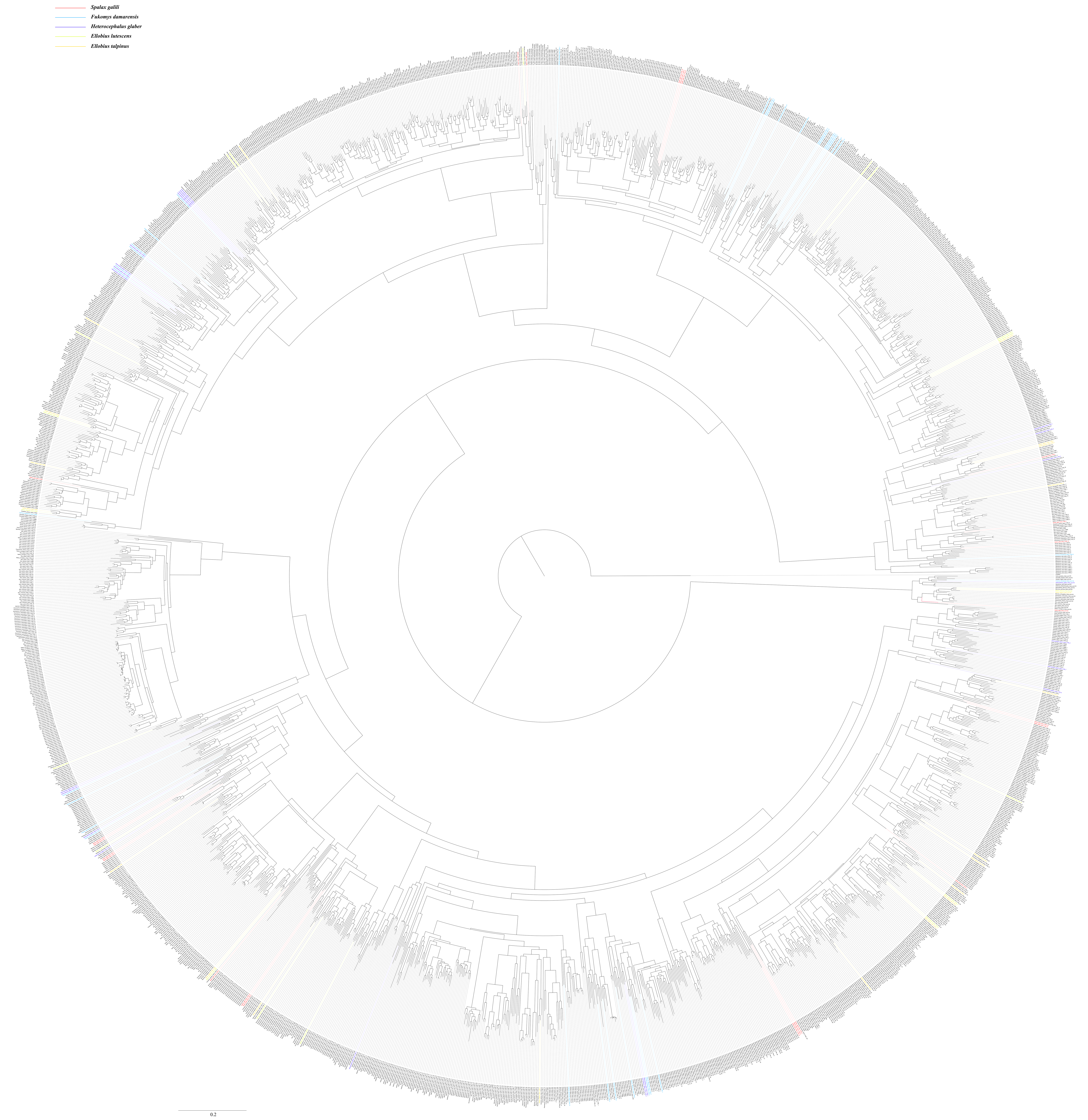

Figure S1. The Bayesian tree based on the alignment of 1983 intact *V1R*s.
